# Different Molecular Signatures in Magnetic Resonance Imaging-Staged Facioscapulohumeral Muscular Dystrophy Muscles

**DOI:** 10.1371/journal.pone.0038779

**Published:** 2012-06-13

**Authors:** Giorgio Tasca, Mario Pescatori, Mauro Monforte, Massimiliano Mirabella, Elisabetta Iannaccone, Roberto Frusciante, Tiziana Cubeddu, Francesco Laschena, Pierfrancesco Ottaviani, Enzo Ricci

**Affiliations:** 1 Don Carlo Gnocchi Onlus Foundation, Milan, Italy; 2 Department of Bioinformatics, Erasmus MC, Rotterdam, The Netherlands; 3 Crosslinks BV, Rotterdam, The Netherlands; 4 Porto Conte Ricerche Srl, Tramariglio, Alghero (SS), Italy; 5 Institute of Neurology, Catholic University School of Medicine, Rome, Italy; 6 Department of Radiology, Istituto Dermopatico dell’Immacolata IRCCS, Rome, Italy; IRCCS-Policlinico San Donato, Italy

## Abstract

**Background:**

Facioscapulohumeral muscular dystrophy (FSHD) is one of the most common muscular dystrophies and is characterized by a non-conventional genetic mechanism activated by pathogenic D4Z4 repeat contractions. By muscle Magnetic Resonance Imaging (MRI) we observed that T2-short tau inversion recovery (T2-STIR) sequences identify two different conditions in which each muscle can be found before the irreversible dystrophic alteration, marked as T1-weighted sequence hyperintensity, takes place. We studied these conditions in order to obtain further information on the molecular mechanisms involved in the selective wasting of single muscles or muscle groups in this disease.

**Methods:**

Histopathology, gene expression profiling and real time PCR were performed on biopsies from FSHD muscles with different MRI pattern (T1-weighted normal/T2-STIR normal and T1-weighted normal/T2-STIR hyperintense). Data were compared with those from inflammatory myopathies, dysferlinopathies and normal controls. In order to validate obtained results, two additional FSHD samples with different MRI pattern were analyzed.

**Results:**

Myopathic and inflammatory changes characterized T2-STIR hyperintense FSHD muscles, at variance with T2-STIR normal muscles. These two states could be easily distinguished from each other by their transcriptional profile. The comparison between T2-STIR hyperintense FSHD muscles and inflammatory myopathy muscles showed peculiar changes, although many alterations were shared among these conditions.

**Conclusions:**

At the single muscle level, different stages of the disease correspond to the two MRI patterns. T2-STIR hyperintense FSHD muscles are more similar to inflammatory myopathies than to T2-STIR normal FSHD muscles or other muscular dystrophies, and share with them upregulation of genes involved in innate and adaptive immunity. Our data suggest that selective inflammation, together with perturbation in biological processes such as neoangiogenesis, lipid metabolism and adipokine production, may contribute to the sequential bursts of muscle degeneration that involve individual muscles in an asynchronous manner in this disease.

## Introduction

Facioscapulohumeral muscular dystrophy (FSHD) is an inherited myopathy often characterized by asymmetric muscle involvement [1]. The clinical phenotype of FSHD patients can be heterogeneous, ranging from asymptomatic to wheelchair bound individuals, with wide intrafamilial variability [2]. Extramuscular manifestations may include abnormalities in the retinal vasculature [3].

FSHD is associated with a contraction in a macrosatellite repeat array in the 4q35 chromosomal region D4Z4 [4]. Alterations in the expression of several “candidate” genes, both *in cis* and *in trans* to the pathogenic 4q35 allele, have been proposed to act as major characters in carrying out muscle damage, but their disregulation in FSHD is still a controversial finding [5], [6]. Recent studies significantly improved the understanding of FSHD genetics and suggested a toxic role of a stabilized *DUX4* transcript transcribed from the contracted D4Z4 array [7], [8]. However, despite large progresses in the characterization of the genetic mechanism of the disease, many aspects of the FSHD pathophysiology and the sequence of molecular events associating a potentially cytotoxic lesion with the natural history of the disease are still incompletely described and deserve further investigation.

In muscle disease, microarray gene expression analyses have been widely used to characterize molecular aspects of muscle pathology [9]–[12]. By the use of this technology, several alterations have been described in FSHD muscle, linking the pathogenic process to altered angiogenesis, susceptibility to oxidative stress and abnormal muscle differentiation [13]–[15].

Muscle magnetic resonance imaging (MRI) has been lately introduced into clinical and research practice as a non-invasive and sensitive technique to evaluate the involvement of individual muscles in different myopathies [16], [17]. In FSHD [18], [19], together with muscles showing normal MRI signal and muscles showing abnormalities on T1-weighted (T1-W) MRI sequences (corresponding to areas of fatty fibrous replacement), different authors [20]–[23] have documented the presence of areas characterized by increased signal on T2- short tau inversion recovery (T2-STIR) sequences also in muscles not yet replaced by fat tissue, i.e. showing a normal signal on T1-W sequences. This type of alteration is present in other muscle diseases and reflects an increase in tissue water content usually thought to account for muscle oedema [20] and to underlie an ongoing inflammatory process.

To characterize the pathological changes associated with T2-STIR hyperintensity in FSHD muscle tissue, we performed histopathology and microarray analysis of a set of FSHD biopsies and compared T1-W normal/T2-STIR hyperintense muscle (T2-STIR +) with muscle showing absence of pathological signs on both T1-W and T2-STIR images (T2-STIR -) (Figure S1).

## Methods

### Ethics statement

The Ethics Committee of the Catholic University School of Medicine approved this study. Written informed consent was obtained from all patients.

### Patients and sample collection

Unrelated, genetically confirmed (D4Z4 EcoRI fragment <40Kb) FSHD patients who had undergone lower limb muscle MRI were considered as candidates for the study. MRI was performed as described elsewhere [21]. Patients who met the inclusion criteria (i.e. *i-* having at least one muscle showing hyperintensity on T2-STIR sequences, or *ii*- having normal T1-W and T2-STIR sequence signal on quadriceps muscle) were asked to undergo needle muscle biopsy. Exceptions to this procedure are constituted by sample 1, acquired from an open biopsy made for diagnostic purposes and sample 2, collected during orthopedic surgery. T2-STIR hyperintensity of the corresponding muscles had been previously documented for these samples as well. Twelve FSHD muscle biopsies were obtained from muscles with different MRI signal (6 T2-STIR + and 6 T2-STIR −). The samples were collected according to standard procedures and whenever possible, depending on the sample size, a fragment for histological analysis (frozen in isopentane cooled in liquid nitrogen) and a fragment for RNA extraction (immediately frozen in liquid nitrogen) were taken. Most of the samples underwent both histopathological and gene expression analysis (Table 1). Two of the samples, one T2-STIR + and one T2-STIR – were taken from different muscles of the same patient.

**Table 1:** Summary table of FSHD samples.

Sample	MRI signal	Muscle	Age	Biopsy	Histology	Gene expression
1	T1-W normal/T2-STIR hyperintense	biceps femoris	37	open	marked myopathic changes, endomysial and perimysial connective tissue +, adipocytes ++, endomysial and perivascular inflammatory infiltrates +++	yes
2*	T1-W normal/T2-STIR hyperintense	paravertebral	31	open	marked myopathic changes, endomysial and perimysial connective tissue ++, adipocytes +, necrotic fibers with invading macrophages ++, endomysial and perivascular inflammatory infiltrates +++	yes
3	T1-W normal/T2-STIR hyperintense	quadriceps	37	needle	moderate myopathic changes, endomysial inflammatory infiltrates +	not available
4	T1-W normal/T2-STIR hyperintense	quadriceps	38	needle	moderate myopathic changes, necrotic and regenerating fibers +, endomysial and perivascular inflammatory infiltrates +	not available
5	T1-W normal/T2-STIR hyperintense	quadriceps	20	needle	moderate/marked myopathic changes, interstitial oedema and slightly enlarged vessels, adipocytes +, necrotic fibers +, endomysial and perivascular inflammatory infiltrates ++	yes
6	T1-W normal/T2-STIR hyperintense	biceps femoris	50	needle	not available	yes
7	T1-W and T2-STIR normal	quadriceps	25	needle	mild myopathic changes, no inflammation	yes
8	T1-W and T2-STIR normal	quadriceps	33	needle	mild myopathic changes, no inflammation	not available
9	T1-W and T2-STIR normal	quadriceps	51	needle	mild myopathic changes, no inflammation	yes
10	T1-W and T2-STIR normal	quadriceps	36	needle	minimal myopathic changes, no inflammation	not available
11	T1-W and T2-STIR normal	quadriceps	26	needle	minimal myopathic changes, no inflammation	yes
12*	T1-W and T2-STIR normal	quadriceps	31	needle	mild myopathic changes, no inflammation	yes
T2-STIR + validation	T1-W normal/T2-STIR hyperintense	quadriceps	36	needle	not available	yes
T2-STIR – validation	T1-W and T2-STIR normal	quadriceps	36	needle	not available	yes

+  =  mild increase, ++  =  moderate increase, +++  =  marked increase. * samples from the same patient.

We also included in the study 7 normal controls (age range 18–58), 7 immunopathologically characterized inflammatory myopathies (IM) (2 dermatomyositis, DM, 2 polymyositis, PM, 1 necrotizing myopathy and 2 IM with nonspecific histopathological features) (age range 23–73) and 4 dysferlinopathies (LGMD2B) (age range 28–35). Non-FSHD samples were obtained for diagnostic purposes.

To provide a biological validation of our findings, two additional biopsies were performed in FSHD muscles showing different MRI patterns (T2-STIR + validation and T2-STIR – validation) and analyzed.

### Muscle histology

Eleven FSHD samples (5 T2-STIR + and 6 T2-STIR −) were available for histological analysis, which included hematoxylin-eosin and, whenever possible, routine diagnostic stainings (nicotinamide adenine dinucleotide dehydrogenase tetrazolium reductase, succinate dehydrogenase, cytochrome-C oxidase, modified Gomori trichrome, alkaline phosphatase, and periodic acid-Schiff). Immunohistochemistry protocol for inflammatory markers and antibodies used are described elsewhere [21].

### Gene expression and real time PCR

Eight FSHD samples (4 T2-STIR + and 4 T2-STIR −) were available for gene expression analysis. Total RNA was extracted by TriZol (TriZol reagent, Invitrogen, Carlsbad, CA, USA) and further purified using the RNAeasy mini kit following the RNA cleanup protocol as indicated by the manufacturer (Qiagen, Valencia, CA, USA). RNA purity and integrity were assessed by spectrophotometric analysis and agarose gel electrophoresis. We made use of the Illumina BeadChips technology to analyze the expression of >31,000 annotated genes with more than 47,000 probes using the HumanHT-12v4 Expression BeadChip (Illumina Inc., San Diego, CA, USA). Gene expression data were extracted and normalized using Illumina Genome Studio and analyzed using BRB-ArrayTools, developed by R. Simon and BRB-ArrayTools Development Team. To discriminate the gene expression variations that characterize T2-STIR + FSHD muscles with respect to T2-STIR – FSHD and IM muscles we computed the probability of genes being differentially expressed between the classes using the unequal variance *t* test as implemented in BRB-ArrayTools. A per gene estimate of false discovery rate was computed using the method of Benjamini and Hochberg [24] A MIAME compliant description of the experiment and full dataset have been submitted to the Gene Expression Omnibus public database (www.ncbi.nlm.nih.gov/geo, (GSE26852)). Methods used for gene set expression comparison and real time PCR are detailed in Supporting Information (Text S1).

### Power analysis

To define the minimum fold-change threshold we could confidently interpret as a true change given the size of our study sample, we made use of the SampleSize R package implemented in BRB-ArrayTools (Sample size determination in microarray experiments for class comparison and prognostic classification [25]).

## Results

### Muscle pathology

Routine histopathology displayed moderate to marked myopathic features in T2-STIR + FSHD muscles (Figure S2). Inflammatory infiltrates with CD8+ cell predominance in the endomysium and CD4+ cell predominance in the perimysium were present in all the samples obtained from T2-STIR + FSHD muscles (Figure S3). Further details about the immunocharacterization of inflammatory markers have been described in another paper [21]. In some cases the alterations also included mild increase in endomysial and perimysial connective tissue. Necrosis and myophagias were evident in sample 2, 4 and 5. Some T2-STIR + FSHD samples displayed the presence of adipocytes in perimysial areas. By contrast, samples obtained from 6 T2-STIR – FSHD muscles displayed only mild or minimal myopathic changes and no inflammation was evident on them. Histopathology results are summarized in Table 1.

### Gene expression

#### Comparison of T2-STIR hyperintense FSHD muscles transcriptional pattern with that of T2-STIR normal muscles

**Figure 1:**
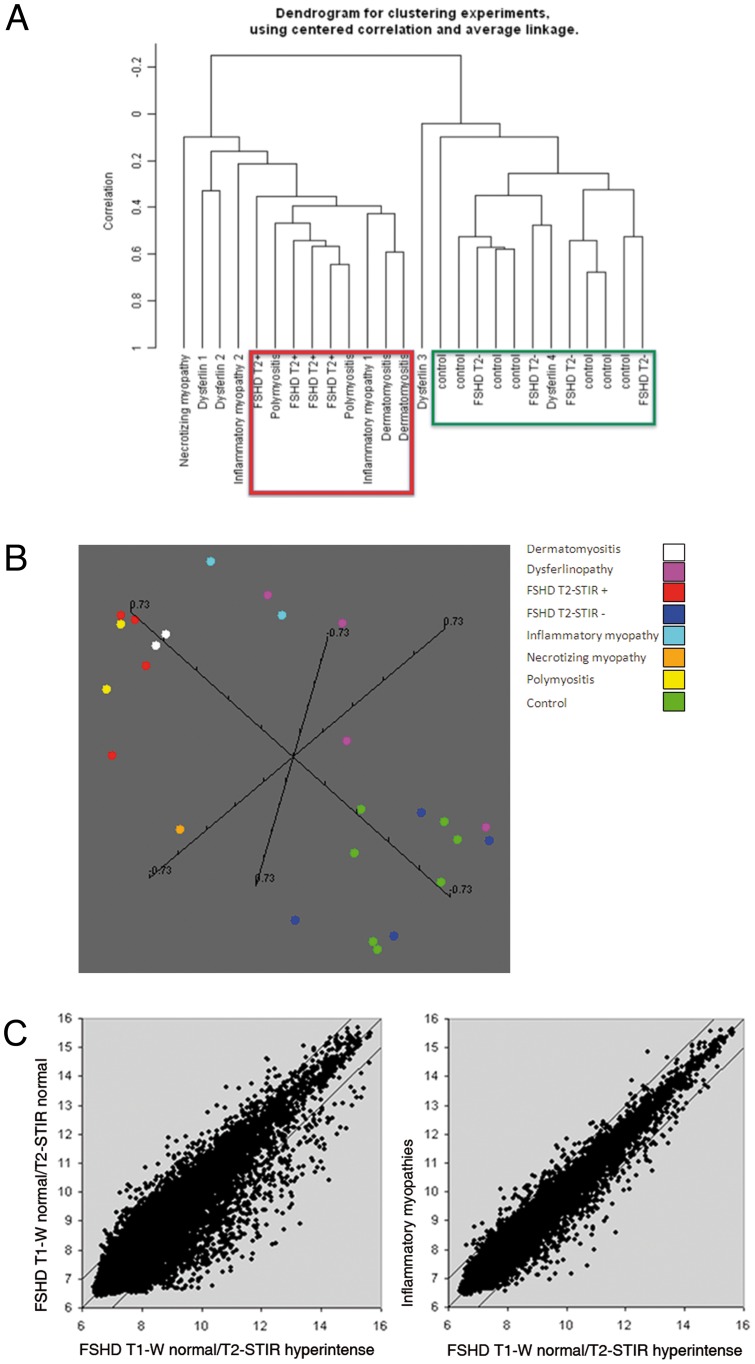
Class discovery graphical displays and scatter plot. (A) Unsupervised hierarchical clustering showing correlation relationships among samples. T2-STIR + FSHD samples are evidently separated from T2-STIR – FSHDs and group with IM (framed in red). T2-STIR – FSHDs show higher correlation with normal controls and one of the dysferlinopathies (framed in green). (B) Multidimensional scaling. Each sample is represented by a sphere in a 3D space. Samples with similar expression profile are shown close together. Note the peculiar and distinct spatial collocation of T2-STIR + FSHD samples clustering together with IM. (C) Scatter plot representation of average transcript expression levels in T2-STIR + FSHD muscles (*n* = 4) compared to T2-STIR – FSHD muscles (*n* = 4) or to inflammatory myopathies (*n* = 7).

To visualize the correlation relationships among our FSHD samples we made use of multidimensional scaling (MDS) analysis, by which samples are positioned in a 3D space on the basis of first three principal components of variability. Based on the expression level of all the 29045 probesets showing detection p-value greater than 0.05, FSHD samples formed two separate clusters corresponding to T2-STIR + and T2-STIR – muscles. A similar clustering pattern of the samples could be observed by Hierarchical clustering analysis (Figure 1A and 1B). FSHD samples, including the two biopsies taken from the same patient, were split into two subgroups according to their MRI pattern.

#### Transcripts differentially expressed between T2-STIR hyperintense and T2-STIR normal FSHD muscles

To describe at the molecular level the on-going process responsible for the observed differences between MRI patterns of FSHD muscle, we compared the expression profiles of T2-STIR + and T2-STIR – FSHD muscles. We first assessed the power of statistical analysis and estimated that our sample size of 4 samples per class was sufficient to confidently identify differences larger than 1log2, if taking into account the 75^th^ percentile of the variance distribution.

By using a random variance *t* test we identified 1948 probesets differentially expressed between the two classes with high statistical significance (p<0.005, 805 with p<0.001). These 1948 probesets, 1089 of which were upregulated, correspond to 1752 different transcripts. A table reporting all the significant probesets, ranked by fold change and tabulated along with between-classes p-value, within-class geometric mean, between-classes fold difference and links to NCBI annotations, is posted under Supporting Information (Table S1).

**Figure 2:**
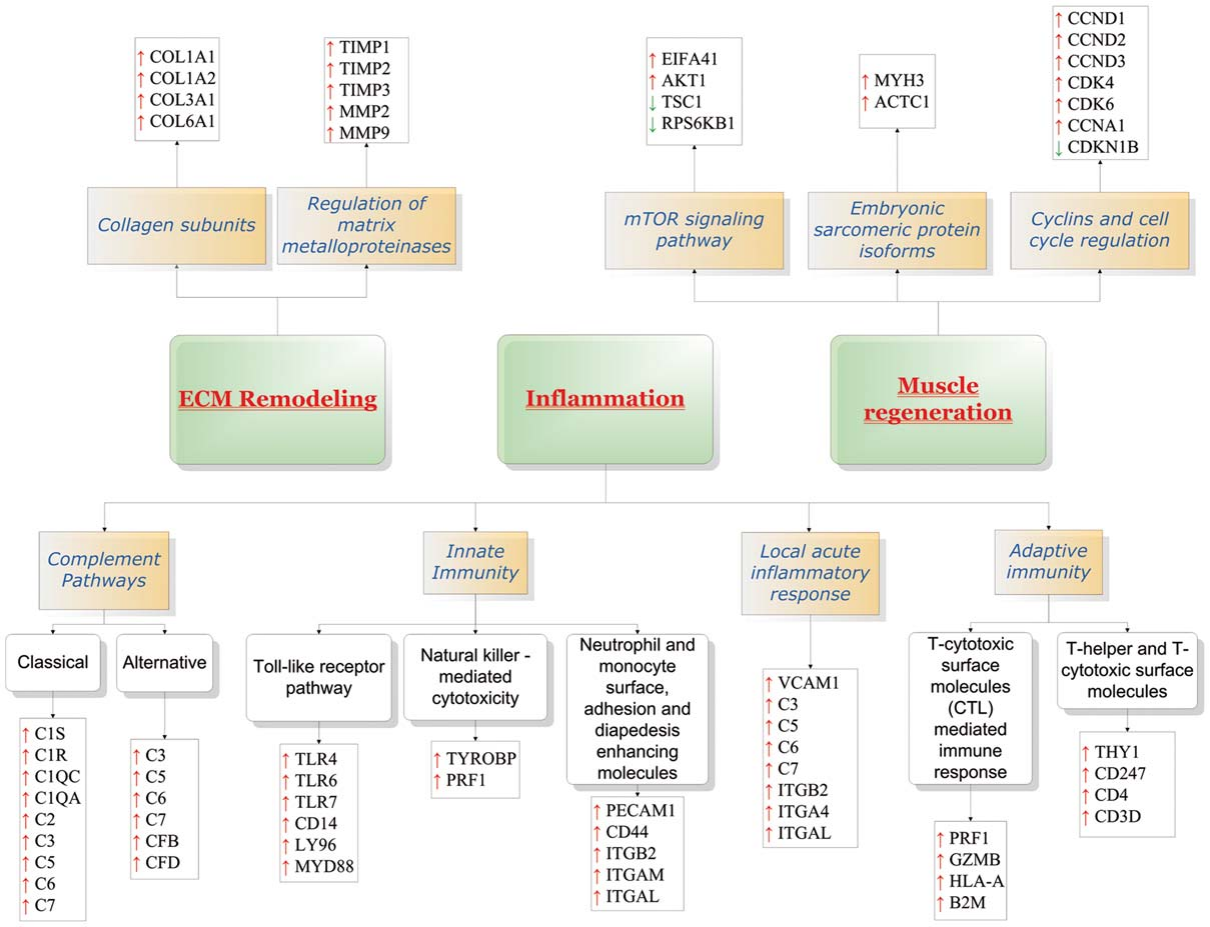
Schematic representation of the molecular pathways modulated in T2-STIR + FSHD samples.

We also tested the hypothesis that individual gene sets were statistically different between the classes by using the gene set class comparison tool implemented in BRB-ArrayTools to query the BioCarta, Gene Ontology, and KEGG databases (Table S2). As a result of this analysis, also integrated by additional available public information on gene function and pathways, a number of the modulated genes have been assigned to the following functional categories: i) Inflammation; ii) Extracellular matrix (ECM) remodeling; iii) Muscle regeneration (Figure 2).

#### Comparison of FSHD muscles transcriptional pattern with that of muscles from other myopathies and normal controls

We used MDS to plot our T2-STIR+ and T2-STIR- samples in a 3D gene expression space together with muscles from normal individuals and patients affected by different types of muscle diseases. As shown in Figure 1A, samples appeared grossly distributed in two large clusters. Samples showing the highest degree of similarity to T2-STIR + FSHD were inflammatory myopathies (IM), and in particular polymyositis (PM). Accordingly, a larger dispersion of the average expression ratios could be observed in a scatter plot analysis of T2-STIR+ vs T2-STIR- FSHD samples respect to T2-STIR+ vs IM (Figure 1C). Dysferlinopathy (DYSF) samples appeared broadly distributed, and distant from T2-STIR + FSHD. We also started characterizing the subtle differences between T2-STIR + FSHD and IM muscles. We identified a group of 286 probesets differentially expressed with a significance level of p<0.005 (Table S3). These 286 probe sets, 176 of which were upregulated, were representative of 229 different genes. The differences between T2-STIR + FSHD muscles and IM mainly mapped to the functional categories: i) Angiogenesis; ii) Adipocyte presence, glycerolipid metabolism and adipokine production (Table 2). Two tables reporting all the significant genes (table S3) and pathways (table S4) with summary statistics and links to annotations are posted under Supporting Information.

**Table 2:** Functional categories and genes modulated in T2-STIR + FSHD with respect to IM.

Functional category	Official Full Name	Official Symbol	Fold change	p-value
*Angiogenesis*	Angiogenin	ANG	2,3725	0,0041
	Angiopoietin-like 2	ANGPTL2	2,5002	0,0014
	CD44 molecule	CD44	2,4375	0,0035
	CD34 molecule	CD34	2,2935	0,0047
	CD47 molecule	CD47	1,6579	0,0013
	Filamin B beta	FLNB	2,2672	0,0056
	Von Willebrand factor	VWF	1,9606	0,0331
	Slit homolog 3	SLIT3	2,6961	0,0047
	Dedicator of cytokinesis 1	DOCK1	1,8760	0,0024
	Secreted frizzled-related protein 1	SFRP1	4,8032	0,0026
	Frizzled homolog 4	FZD4	2,1590	0,0051
	Frizzled homolog 7	FZD7	1,3833	0,0401
*Adipocyte presence, glycerolipid metabolism* *and adipokine production*	Cell death-inducing DFFA-like effector a	CIDEA	9,7264	0,0009
	Cell death-inducing DFFA-like effector c	CIDEC	8,5623	0,0038
	Perilipin 1	PLIN	5,6435	0,0017
	Glycerol-3-phosphate acyltransferase	GPAM	4,7128	0,0023
	Alcohol dehydrogenase 1A	ADH1A	4,1328	0,0016
	Adiponectin, C1Q and collagen domain containing	ADIPOQ	2,6172	0,0019
	C1q and tumor necrosis factor related protein 1	C1QTNF1	2,6181	0,0013
	Leptin	LEP	3,9142	0,0544

All of the cited genes were expressed to a higher extent in T2-STIR + FSHD muscles.

**Figure 3:**
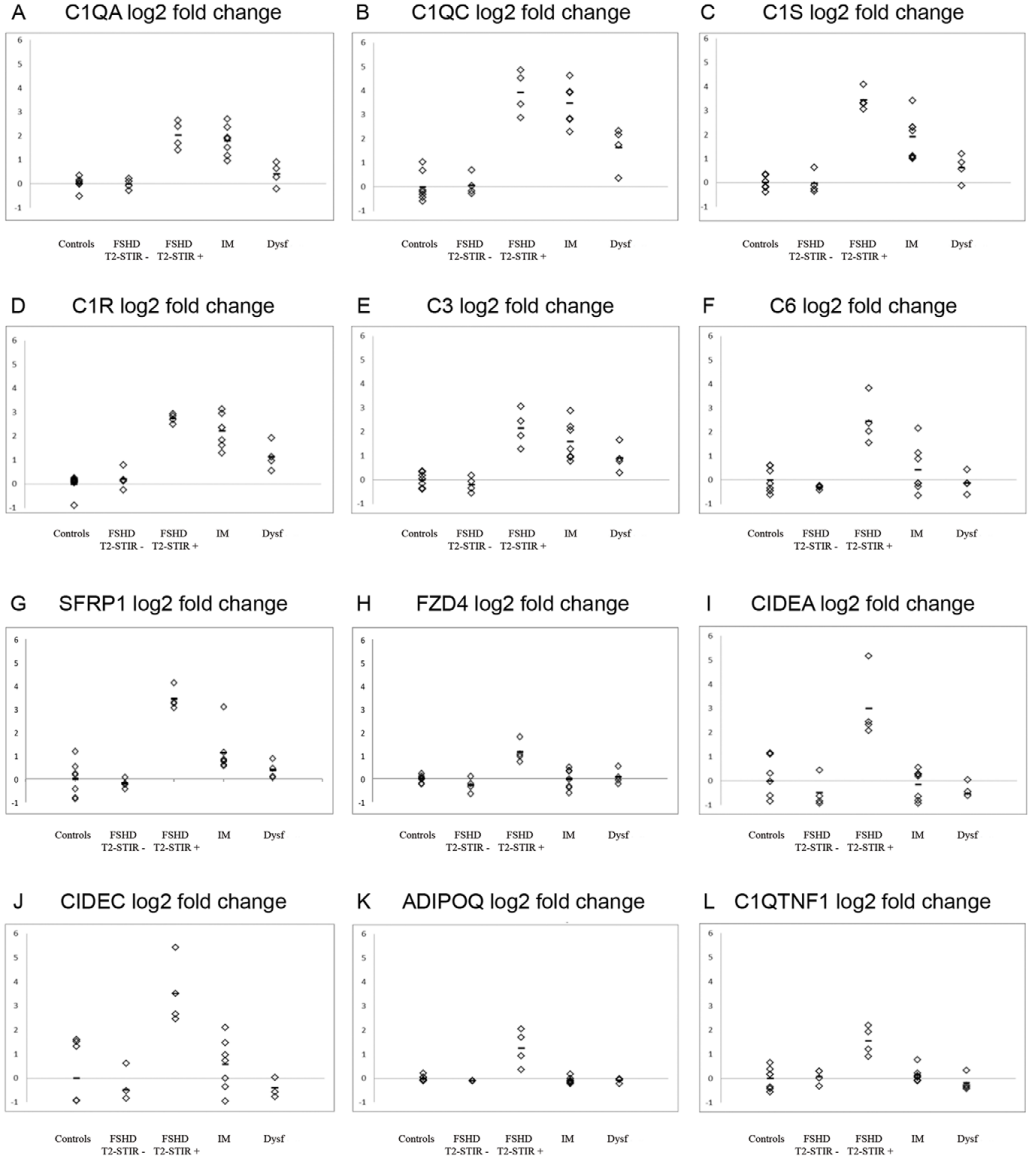
Expression levels of single genes across samples. Co-regulation of functionally related genes: (A–F) members of complement system, (G–H) co-regulated and selective induction in T2-STIR + FSHD muscles can be observed for SFRP1 and its receptor FZD4 and (I–L) for adipose tissue genes and inflammatory adipokines.

Further validating the consistency of the expression data within the identified functional categories, we observed co-regulation of a number of functionally related genes (Figure 3).

#### Real time PCR

**Figure 4:**
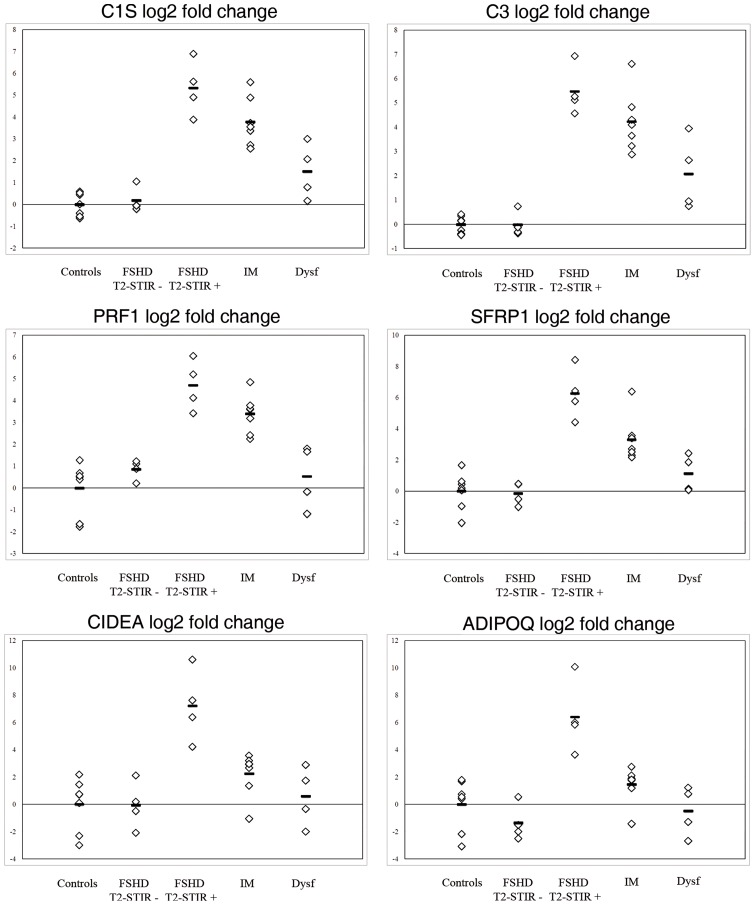
Real time PCR. Real time PCR analysis of selected gene expression. All the pathological samples display alterations consistent with the gene chip expression level. Changes in transcript abundance are expressed as log2 ratio to control mean.

We performed a technical validation of our microarray experiment and verified by real time PCR some of the gene expression changes identified by microarray analysis (*C1S, C3, PFR1, SFRP1, CIDEA, ADIPOQ*). All of the analyzed genes showed expression changes consistent with those estimated by gene chip analysis (Figure 4). The reported data have been obtained using *GAPDH* as endogenous control gene, although similar results have been obtained with *HPRT1* (not shown).

#### Validation

**Figure 5:**
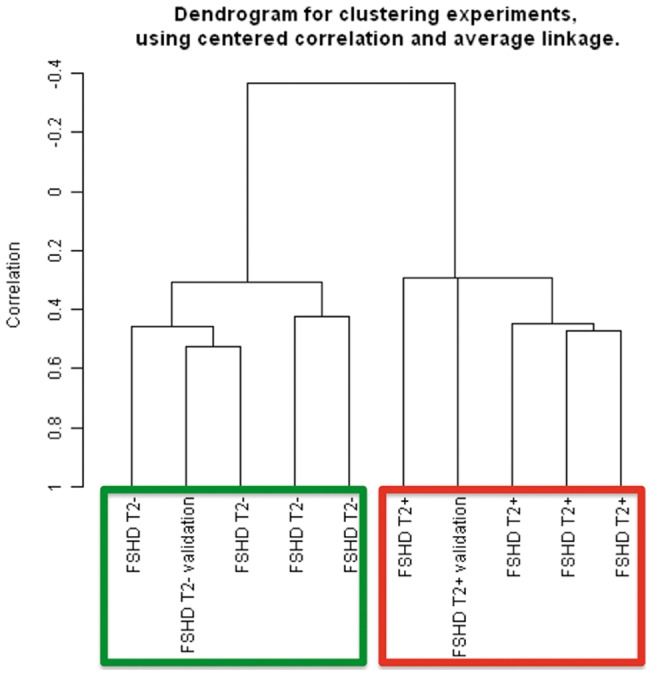
Unsupervised hierarchical clustering of the validation experiment.

To further validate at the biological level the consistency of obtained results we prospectively performed two additional biopsies, one in a FSHD T2-STIR + and one in a FSHD T2-STIR – muscle and generated expression profiles for these two muscle specimens. We normalized the two new arrays together with the overall set of FSHD arrays previously generated and performed a correlation analysis. As can be observed in Figure 5, the two validation samples also clustered according to their MRI pattern.

#### Expression of DUX4, DUX4-targets and 4q35 FSHD candidate genes

Based on the signal level of the two probesets interrogating *DUX4* present in the HumanHT-12v4 BeadChip, we observed no evidence of increased expression of this gene in our FSHD samples. We checked for disregulations in the expression levels of genes recently implicated in the *DUX4*-dependent transcriptional network [26]. By directly browsing the expression dataset, we observed overexpression of a number of *DUX4*-target genes in two out of four FSHD T2-STIR + samples (*ZSCAN4, PRAMEF1, PRAMEF 4–6, PRAMEF9, MBD3L2, DEFB103B*) (Figure S4).

Other genes previously suggested to play a role in FSHD pathogenesis like *PITX1* [27] as well as D4Z4 proximal genes (*FRG1, FRG2, ANT1, LRP2BP*) were expressed at comparable levels in T2-STIR + and T2-STIR – FSHD muscles (not shown).

## Discussion

Histopathological differences that emerge from the comparison of FSHD T2-STIR + with T2-STIR – muscles include myopathic alterations, increased endomysial spaces, presence of adipocytes in perimysial areas and scattered necrotic phenomena. All our T2-STIR + FSHD samples displayed inflammatory changes on immunohistochemistry [21]. These alterations could represent the beginning of a dystrophic process. At the molecular level, T2-STIR + FSHD muscle is different from T2-STIR – FSHD muscle. The differences described were consistent across different muscle types. We ruled out a major effect of two possible confounders, muscle type and genetic make up of the individual patients, because quadriceps muscle samples were positioned in two separate clusters according to their MRI pattern, as were the two muscles taken from the same individual. Of note, the majority of differentially expressed genes was upregulated in T2-STIR + FSHD muscles, suggesting that the transition from T2-STIR – to T2-STIR + is an “active” process. In T2-STIR + muscles we found a significant upregulation of genes involved in innate and adaptive immune response and in particular activation of classical and alternative complement pathways. Extrahepatic synthesis of complement factors by mesenchymal cells, such as endothelial cells or adipocytes, or inflammatory cells, such as macrophages, has been documented in inflammatory conditions [28]. Complement activation could enhance chemoattraction of inflammatory cells through the production of anaphilotoxins [29], or directly modulate both T-cell and B-cell mediated adaptive immunity [30]. Another innate immunity pathway induced in T2-STIR + FSHD muscles is the Toll-like receptor (TLR) pathway [31]. TLR expression has been demonstrated in inflammatory, epithelial and endothelial cells, and also in PM and DM muscle fibers [32]. Moreover, we found evidence of upregulation of endothelial and inflammatory cell adhesion molecules as well, likely contributing to immune cell diapedesis.

Together with innate immunity, adaptive immunity processes and in particular T-cell mediated immune responses were induced. Accordingly, we have described increased presence of activated circulating CD8+ T-cells in FSHD patients displaying T2-STIR hyperintense muscles [21]. Of note, together with CD8+ T-cell surface and co-stimulatory molecules we found an increased expression of genes involved in direct cell-mediated cytotoxicity, such as granzyme-B and perforin. Another expression study described a disregulation of chemokines from chromosome 4q in FSHD patients [33]. Interestingly, in line with a recent report, half of our T2-STIR + FSHD samples showed overexpression of *DEFB103B*, which is a *DUX4*-dependent gene involved in the modulation of both adaptive and innate immune responses, and PRAME family cancer testis antigens [26]. This adds further evidence to the fascinating perspective that bursts of *DUX4* expression may induce damage by triggering a sustained expression of genes causing a multifocal, inapposite immune response [26].

T2-STIR + FSHD samples appear more similar to IM, and in particular PM, while T2-STIR – muscles group with normal controls. Although many transcriptional alterations were shared between T2-STIR + FSHD and IM muscles, differences were present that mainly regarded the overexpression in T2-STIR + FSHD muscles of a number of proangiogenic factors and surface proteins involved in cell-cell adhesion and cell-ECM interactions (Table 2), consistently with previous findings [13]. To this regard, an interesting group of genes is constituted by *SFRP1, SFRP2, FZD4* (which are co-regulated in our dataset) and *FZD7*. SFRP1 and 2 (secreted Frizzled-related proteins 1 and 2) are soluble modulators, while FZD4 and 7 (Frizzled 4 and 7) are surface receptors, in the Wnt signaling pathway. Wnt/Frizzled signaling is involved in normal angiogenesis and neovessel formation after ischemic injury. In particular, SFRP1 acts through Frizzled 4 and 7 inducing vascular cell spreading [34] and proliferation [35] and also on mesenchymal stem cells promoting neoangiogenesis [36].

Several T2-STIR + FSHD upregulated genes can be ascribed to adipocyte metabolism. SFRP1 itself has proadipogenic activity [37]. *CIDEA* and *CIDEC* (cell death inducing DFFA-like effector A and C) encode proteins whose major localization is in white adipose tissue and hepatocytes. However, minor contents of *CIDEA* mRNA have been also detected in skeletal muscle [38] and macrophages [39]. *CIDEA* has an inhibitory action on the lipolytic process [40] and its expression in adipocytes is increased in human cancer cachexia [41]. Other upregulated genes are involved in glycerolipid metabolism (Table 2). Interestingly, in parallel with adipocyte genes, we found a T2-STIR + FSHD selective upregulation of genes encoding immunomodulatory adipokines (Figure 3). *ADIPOQ* encodes adiponectin, a cytokine important for macrophage recruitment that has been implicated in the pathogenesis of rheumatoid arthritis [42]. Synthesis of adiponectin by muscle cells under inflammatory conditions has been documented as well [43]. *C1QTNF1* encodes C1q and tumor necrosis factor related protein-1, another cytokine associated with a low-grade chronic inflammation status in adipose tissue [44]. We could speculate that adipose tissue is not only a bystander of the pathological process but may contribute to the maintenance of a chronic inflammatory milieu. A large number of the alterations described were not detectable in our T2-STIR – FSHD samples. Therefore, the modulation of these pathways constitutes a specific “molecular signature” of the disease in this particular pathological phase.

### Conclusions

In FSHD, different muscles of each patient can, at the same time, display different stages of the dystrophic process, characterized by peculiar and distinct histological and molecular alterations. Thus, even though the aim of our study was not to identify what distinguishes FSHD from normal muscle, it emerges that taking one “snapshot” of the pathologic process could be not enough to identify the process itself. This strict dependency on the timing and the muscle in which the “snapshot” is taken could have contributed to the diverse results obtained in different FSHD gene expression studies [14], [15], [27], [33]. This constitutes a substantial difference from, for instance, Duchenne muscular dystrophy, in which the molecular signature develops early and remains constant all over the disease course [12].

The early stages of involvement of each FSHD muscle, defined by the absence of T1-W hyperintensity, are identifiable through muscle MRI based on their T2-STIR normal or hyperintense signal. MRI with T2-STIR sequences, marking different phases of the disease at the single muscle level, is a useful and reliable tool in monitoring the progression of involvement of FSHD muscles and with this aim it should be taken into consideration in the future clinical trials in this disease.

The pronounced similarity with PM and DM of the T2-STIR + FSHD muscle transcriptional profile, characterized by a marked upregulation of genes involved in innate and adaptive immune response, suggests that a selective and multifocal inflammatory process may play an active role in the development of dystrophic changes and consequently in disease advancement at single muscle level. However, some aspects of our analysis may be limited in power due to the moderate sample size. Further studies are needed to clarify the exact significance of the specific biological processes active in T2-STIR + muscles in determining muscle damage, in a phase in which tissue modifications are still theoretically reversible.

## Supporting Information

Figure S1 **Early stages of involvement of FSHD muscles.** Images representative of T1-W normal/T2-STIR normal (T2-STIR -, star) and T1-W normal/T2-STIR hyperintense (T2-STIR +, arrowhead) muscles.
(TIF)

Figure S2 **Hematoxylin-eosin stainings of T2-STIR + (A, C) and T2-STIR – (B) FSHD muscles.** Note the presence, also evident at low magnification, of major myopathic changes, which include increase in fiber size variability, central nuclei, inflammatory infiltrates, increase in endomysial connective tissue in T2-STIR + muscles. T2-STIR – muscle only displays mild myopathic alterations. This differences are even more striking considering that (B) and (C) are samples from the same patient. (A) sample 1; (B) sample 12; (C) sample 2. Scale bar 100 μm.
(TIF)

Figure S3 **Immunofluorescence of T2-STIR + FSHD muscles with anti-CD8 (red, A) and anti-CD4 (red, B) antibodies.** CD8+ T-cells are the main component of endomysial inflammatory infiltrates, while CD4+ T-cells predominate in the perivascular regions. Muscle fibers are counterstained with FITC-conjugated phalloidin (green), nuclei are stained with DAPI (blue). (A) sample 3; (B) sample 5.
(TIF)

Figure S4 **Expression levels of** ***DUX4*** **-target genes.** Two out of four T2-STIR + FSHD samples express the highest levels of the *DUX4*-target genes *ZSCAN4, PRAMEF1, PRAMEF 4–6, PRAMEF9, MBD3L2* (A) and *DEFB103B* (B), which is also induced in inflammatory myopathies.
(TIF)

Table S1 **Class comparison of T2-STIR + vs. T2-STIR – FSHD muscles.**
(DOC)

Table S2 **BioCarta, Gene Ontology, and KEGG gene set expression comparison of T2-STIR + vs. T2-STIR – FSHD muscles.**
(DOC)

Table S3 **Class comparison of T2-STIR + FSHD vs. Inflammatory myopathy muscles.**
(DOC)

Table S4 **BioCarta, Gene Ontology, and KEGG gene set expression comparison of T2-STIR + FSHD vs. Inflammatory myopathy muscles.**
(DOC)

Text S1 **Supplementary methods: gene set expression comparison and real time PCR.**
(DOC)
