# Supplementary figures and images for: Different Molecular Signatures in Magnetic Resonance Imaging-Staged Facioscapulohumeral Muscular Dystrophy Muscles
Source: PLoS One. 2012 Jun 13;7(6):e38779. doi: 10.1371/journal.pone.0038779 (PMC3374833; doi:10.1371/journal.pone.0038779)

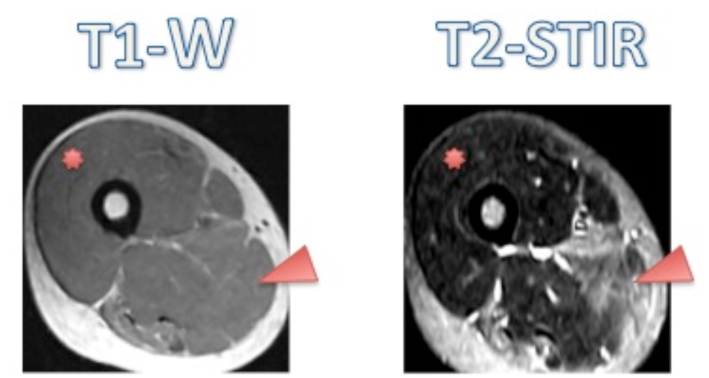

Supplement: Figure S1 — Early stages of involvement of FSHD muscles. Images representative of T1-W normal/T2-STIR normal (T2-STIR -, star) and T1-W normal/T2-STIR hyperintense (T2-STIR +, arrowhead) muscles. (TIF) [file pone.0038779.s001.tif]

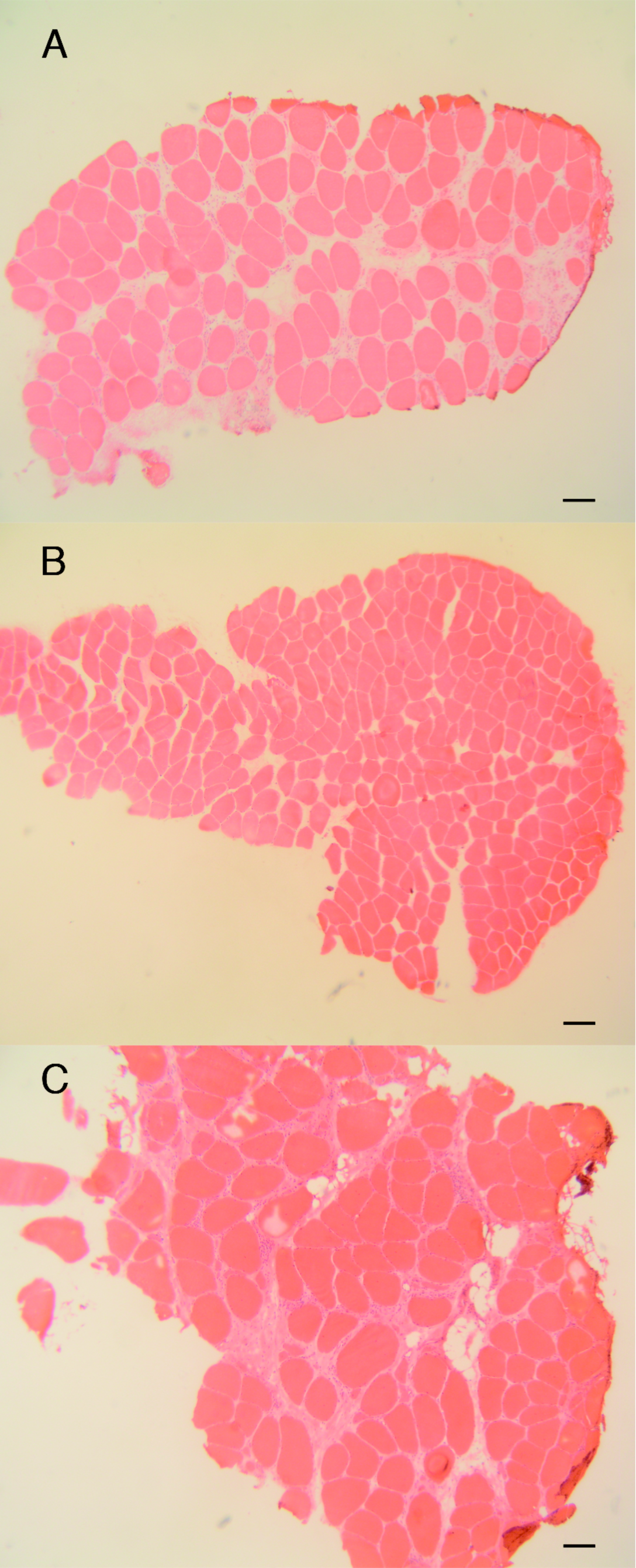

Supplement: Figure S2 — Hematoxylin-eosin stainings of T2-STIR + (A, C) and T2-STIR – (B) FSHD muscles. Note the presence, also evident at low magnification, of major myopathic changes, which include increase in fiber size variability, central nuclei, inflammatory infiltrates, increase in endomysial connective tissue in T2-STIR + muscles. T2-STIR – muscle only displays mild myopathic alterations. This differences are even more striking considering that (B) and (C) are samples from the same patient. (A) sample 1; (B) sample 12; (C) sample 2. Scale bar 100 μm. (TIF) [file pone.0038779.s002.tif]

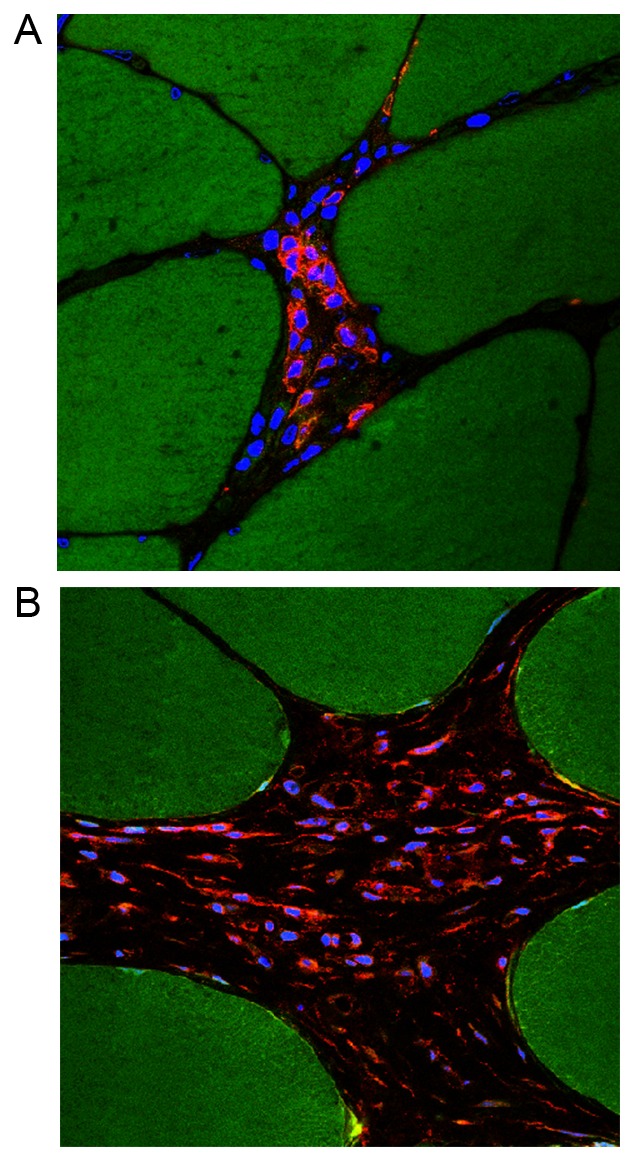

Supplement: Figure S3 — Immunofluorescence of T2-STIR + FSHD muscles with anti-CD8 (red, A) and anti-CD4 (red, B) antibodies. CD8+ T-cells are the main component of endomysial inflammatory infiltrates, while CD4+ T-cells predominate in the perivascular regions. Muscle fibers are counterstained with FITC-conjugated phalloidin (green), nuclei are stained with DAPI (blue). (A) sample 3; (B) sample 5. (TIF) [file pone.0038779.s003.tif]

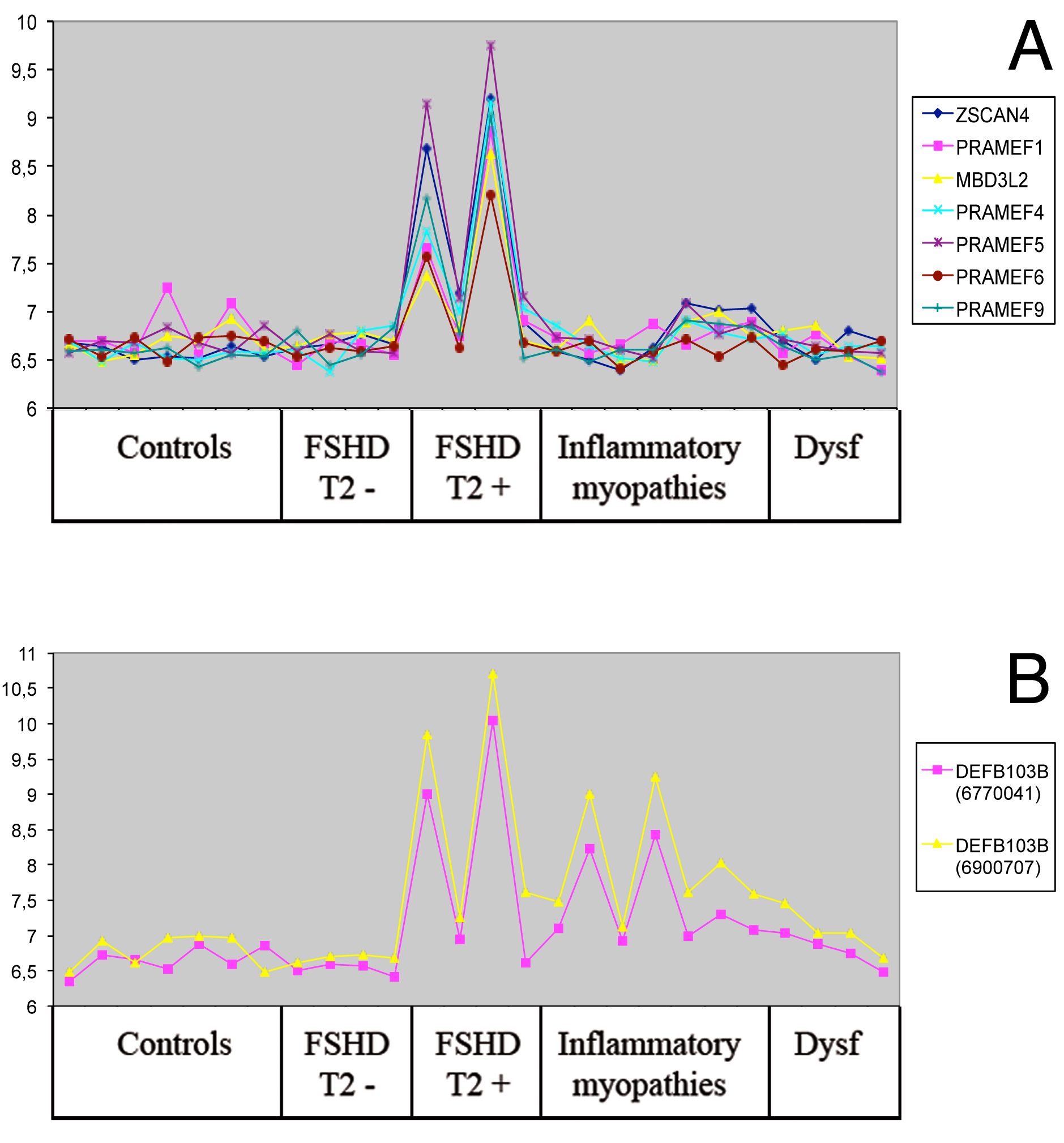

Supplement: Figure S4 — Expression levels of DUX4 -target genes. Two out of four T2-STIR + FSHD samples express the highest levels of the DUX4-target genes ZSCAN4, PRAMEF1, PRAMEF 4–6, PRAMEF9, MBD3L2 (A) and DEFB103B (B), which is also induced in inflammatory myopathies. (TIF) [file pone.0038779.s004.tif]
