# Supplementary material for: Different Molecular Signatures in Magnetic Resonance Imaging-Staged Facioscapulohumeral Muscular Dystrophy Muscles
Source: PLoS One. 2012 Jun 13;7(6):e38779. doi: 10.1371/journal.pone.0038779 (PMC3374833; doi:10.1371/journal.pone.0038779)
Supplement: Table S1 — Class comparison of T2-STIR + vs. T2-STIR – FSHD muscles. (DOC) [file pone.0038779.s005.doc]

**Table S1: Class Comparison FSHD T2-STIR + vs. T2-STIR -**

| **Parametric**  **p-value** | **FDR** | **Geom mean of intensities in FSHD**  **T2-STIR +** | **Geom mean of intensities in FSHD**  **T2-STIR -** | **Fold-change** | **Unique id** | **Gene symbol** | **DefinedGenelist** |
| --- | --- | --- | --- | --- | --- | --- | --- |
| 0.0013663 | 0.040869 | 5733.074674 | 299.9023899 | 19.116469 | 6960142 | [COL1A1](http://www.ncbi.nlm.nih.gov/entrez/query.fcgi?cmd=search&db=gene&term=COL1A1) | Cell Communication, ECM-receptor interaction, Focal adhesion, immunology |
| 0.0006911 | 0.030043 | 5278.90615 | 292.275119 | 18.061428 | 2140128 | [SCD](http://www.ncbi.nlm.nih.gov/entrez/query.fcgi?cmd=search&db=gene&term=SCD) | PPAR signaling pathway |
| 0.0021464 | 0.050338 | 3252.113608 | 203.6236635 | 15.971197 | 780309 | [CIDEC](http://www.ncbi.nlm.nih.gov/entrez/query.fcgi?cmd=search&db=gene&term=CIDEC) |  |
| 0.0002784 | 0.022708 | 3396.833962 | 234.4323801 | 14.489611 | 1440341 | [C1QC](http://www.ncbi.nlm.nih.gov/entrez/query.fcgi?cmd=search&db=gene&term=C1QC) | Complement and coagulation cascades |
| 0.0012699 | 0.039594 | 2000.054243 | 153.212884 | 13.054087 | 3060639 | [SFRP2](http://www.ncbi.nlm.nih.gov/entrez/query.fcgi?cmd=search&db=gene&term=SFRP2) | Wnt signaling pathway, development |
| 7.90E-06 | 0.012163 | 2619.070765 | 212.135307 | 12.346228 | 6960379 | [SFRP1](http://www.ncbi.nlm.nih.gov/entrez/query.fcgi?cmd=search&db=gene&term=SFRP1) | Wnt signaling pathway, development |
| 0.0005979 | 0.028704 | 1325.454379 | 108.376835 | 12.230053 | 4860152 | [PLA2G2A](http://www.ncbi.nlm.nih.gov/entrez/query.fcgi?cmd=search&db=gene&term=PLA2G2A) | Arachidonic acid metabolism, Fc epsilon RI signaling pathway, Glycerophospholipid metabolism, GnRH signaling pathway, Linoleic acid metabolism, Long-term depression, MAPK signaling pathway, VEGF signaling pathway, cell_signaling, signal_transduction, tsonc |
| 5.43E-05 | 0.015462 | 2005.03255 | 165.5125906 | 12.114079 | 6480204 | [THY1](http://www.ncbi.nlm.nih.gov/entrez/query.fcgi?cmd=search&db=gene&term=THY1) | T Cytotoxic Cell Surface Molecules, T Helper Cell Surface Molecules, Leukocyte transendothelial migration, immunology |

| 0.0002805 | 0.022708 | 3989.78426 | 349.4533369 | 11.417216 | 5690519 | [PLTP](http://www.ncbi.nlm.nih.gov/entrez/query.fcgi?cmd=search&db=gene&term=PLTP) | PPAR signaling pathway |
| --- | --- | --- | --- | --- | --- | --- | --- |
| 0.0019398 | 0.048328 | 7939.637087 | 697.7105334 | 11.379557 | 6060612 | [COL1A2](http://www.ncbi.nlm.nih.gov/entrez/query.fcgi?cmd=search&db=gene&term=COL1A2) | Cell Communication, ECM-receptor interaction, Focal adhesion, immunology |
| 0.0044321 | 0.070926 | 1687.55813 | 149.9285842 | 11.255747 | 10048 | [CIDEA](http://www.ncbi.nlm.nih.gov/entrez/query.fcgi?cmd=search&db=gene&term=CIDEA) |  |
| 0.0005713 | 0.028659 | 6726.950216 | 603.3221321 | 11.149848 | 6900408 | [EFEMP1](http://www.ncbi.nlm.nih.gov/entrez/query.fcgi?cmd=search&db=gene&term=EFEMP1) |  |
| 3.43E-05 | 0.014558 | 8396.28172 | 757.9367786 | 11.077813 | 7400707 | [C1S](http://www.ncbi.nlm.nih.gov/entrez/query.fcgi?cmd=search&db=gene&term=C1S) | Classical Complement Pathway, Complement Pathway, Complement and coagulation cascades, immunology |
| 5.36E-05 | 0.015414 | 3741.309351 | 339.1056498 | 11.032872 | 4900133 | [SRPX](http://www.ncbi.nlm.nih.gov/entrez/query.fcgi?cmd=search&db=gene&term=SRPX) |  |
| 0.000444 | 0.026228 | 3361.927991 | 317.2523116 | 10.597017 | 2370358 | [TYROBP](http://www.ncbi.nlm.nih.gov/entrez/query.fcgi?cmd=search&db=gene&term=TYROBP) | Natural killer cell mediated cytotoxicity, signal_transduction |
| 0.0005458 | 0.028008 | 4638.398107 | 459.8132357 | 10.08757 | 3890373 | [ITGB2](http://www.ncbi.nlm.nih.gov/entrez/query.fcgi?cmd=search&db=gene&term=ITGB2) | Adhesion and Diapedesis of Granulocytes, Adhesion and Diapedesis of Lymphocytes, Adhesion Molecules on Lymphocyte, B Lymphocyte Cell Surface Molecules, Cells and Molecules involved in local acute inflammatory response, CTL mediated immune response against target cells , Monocyte and its Surface Molecules, Neutrophil and Its Surface Molecules, T Cytotoxic Cell Surface Molecules, T Helper Cell Surface Molecules, Cell adhesion molecules (CAMs), Leukocyte transendothelial migration, Natural killer cell mediated cytotoxicity, Regulation of actin cytoskeleton, cell_signaling, immunology, metastasis |
| 0.0007602 | 0.031543 | 3697.0688 | 387.0507148 | 9.5518976 | 7200156 | [ITGB2](http://www.ncbi.nlm.nih.gov/entrez/query.fcgi?cmd=search&db=gene&term=ITGB2) | Adhesion and Diapedesis of Granulocytes, Adhesion and Diapedesis of Lymphocytes, Adhesion Molecules on Lymphocyte, B Lymphocyte Cell Surface Molecules, Cells and Molecules involved in local acute inflammatory response, CTL mediated immune response against target cells , Monocyte and its Surface Molecules, Neutrophil and Its Surface Molecules, T Cytotoxic Cell Surface Molecules, T Helper Cell Surface Molecules, Cell adhesion molecules (CAMs), Leukocyte transendothelial migration, Natural killer cell mediated cytotoxicity, Regulation of actin cytoskeleton, cell_signaling, immunology, metastasis |
| 0.0008018 | 0.032571 | 17009.55851 | 1798.807345 | 9.4560202 | 2120021 | [COL3A1](http://www.ncbi.nlm.nih.gov/entrez/query.fcgi?cmd=search&db=gene&term=COL3A1) | Cell Communication, ECM-receptor interaction, Focal adhesion, immunology |
| 7.15E-05 | 0.016325 | 1917.92826 | 204.145614 | 9.3949031 | 2680092 | [CD163](http://www.ncbi.nlm.nih.gov/entrez/query.fcgi?cmd=search&db=gene&term=CD163) |  |
| 0.000433 | 0.026228 | 8313.708645 | 911.3499031 | 9.1224113 | 7650433 | [TIMP1](http://www.ncbi.nlm.nih.gov/entrez/query.fcgi?cmd=search&db=gene&term=TIMP1) | Inhibition of Matrix Metalloproteinases, angiogenesis |
| 0.0003988 | 0.025361 | 5868.401481 | 649.3582013 | 9.0372332 | 730414 | [APOE](http://www.ncbi.nlm.nih.gov/entrez/query.fcgi?cmd=search&db=gene&term=APOE) | Alzheimer\'s disease, Neurodegenerative Disorders, immunology |
| 0.0003138 | 0.023475 | 2137.463946 | 239.2459848 | 8.9341685 | 2140121 | [BASP1](http://www.ncbi.nlm.nih.gov/entrez/query.fcgi?cmd=search&db=gene&term=BASP1) |  |
| 0.0014891 | 0.042622 | 14290.04355 | 1652.789113 | 8.6460175 | 4040671 | [COL1A2](http://www.ncbi.nlm.nih.gov/entrez/query.fcgi?cmd=search&db=gene&term=COL1A2) | Cell Communication, ECM-receptor interaction, Focal adhesion, immunology |
| 0.0002275 | 0.020993 | 3610.246984 | 429.6022214 | 8.4036972 | 3120370 | [CD68](http://www.ncbi.nlm.nih.gov/entrez/query.fcgi?cmd=search&db=gene&term=CD68) | angiogenesis, immunology, metastasis |
| 0.0006433 | 0.029369 | 15036.53429 | 1826.94559 | 8.2304226 | 270168 | [HLA-DRA](http://www.ncbi.nlm.nih.gov/entrez/query.fcgi?cmd=search&db=gene&term=HLA-DRA) | Activation of Csk by cAMP-dependent Protein Kinase Inhibits Signaling through the T Cell Receptor, Antigen Dependent B Cell Activation, Antigen Processing and Presentation, B Lymphocyte Cell Surface Molecules, Bystander B Cell Activation, Cytokines and Inflammatory Response, IL 5 Signaling Pathway, Lck and Fyn tyrosine kinases in initiation of TCR Activation, Th1/Th2 Differentiation, The Co-Stimulatory Signal During T-cell Activation, The Role of Eosinophils in the Chemokine Network of Allergy, Antigen processing and presentation, Cell adhesion molecules (CAMs), Hematopoietic cell lineage, Type I diabetes mellitus, immunology |
| 0.0004012 | 0.025383 | 8230.338431 | 1010.614531 | 8.1438948 | 6370369 | [CD14](http://www.ncbi.nlm.nih.gov/entrez/query.fcgi?cmd=search&db=gene&term=CD14) | Inactivation of Gsk3 by AKT causes accumulation of b-catenin in Alveolar Macrophages, Toll-Like Receptor Pathway, Hematopoietic cell lineage, MAPK signaling pathway, Regulation of actin cytoskeleton, Toll-like receptor signaling pathway, angiogenesis, immunology, metastasis, misc |
| 0.0005818 | 0.028704 | 3744.999863 | 461.3106635 | 8.1181732 | 520360 | [MS4A6A](http://www.ncbi.nlm.nih.gov/entrez/query.fcgi?cmd=search&db=gene&term=MS4A6A) |  |
| 0.0028231 | 0.057692 | 8158.107447 | 1010.96131 | 8.0696535 | 2490364 | [LUM](http://www.ncbi.nlm.nih.gov/entrez/query.fcgi?cmd=search&db=gene&term=LUM) |  |
| 0.0001744 | 0.019771 | 1721.32303 | 216.8667132 | 7.9372394 | 20593 | [CD163](http://www.ncbi.nlm.nih.gov/entrez/query.fcgi?cmd=search&db=gene&term=CD163) |  |
| 0.0010691 | 0.037055 | 16407.8741 | 2127.97667 | 7.7105517 | 2570564 | [HLA-DRA](http://www.ncbi.nlm.nih.gov/entrez/query.fcgi?cmd=search&db=gene&term=HLA-DRA) | Activation of Csk by cAMP-dependent Protein Kinase Inhibits Signaling through the T Cell Receptor, Antigen Dependent B Cell Activation, Antigen Processing and Presentation, B Lymphocyte Cell Surface Molecules, Bystander B Cell Activation, Cytokines and Inflammatory Response, IL 5 Signaling Pathway, Lck and Fyn tyrosine kinases in initiation of TCR Activation, Th1/Th2 Differentiation, The Co-Stimulatory Signal During T-cell Activation, The Role of Eosinophils in the Chemokine Network of Allergy, Antigen processing and presentation, Cell adhesion molecules (CAMs), Hematopoietic cell lineage, Type I diabetes mellitus, immunology |
| 0.0002365 | 0.021369 | 3435.996023 | 445.8449759 | 7.7067057 | 1070014 | [CSF1R](http://www.ncbi.nlm.nih.gov/entrez/query.fcgi?cmd=search&db=gene&term=CSF1R) | immunology, tsonc |
| 0.0001774 | 0.019818 | 1522.126925 | 197.7714431 | 7.6963939 | 2900390 | [VCAM1](http://www.ncbi.nlm.nih.gov/entrez/query.fcgi?cmd=search&db=gene&term=VCAM1) | Adhesion and Diapedesis of Lymphocytes, Cells and Molecules involved in local acute inflammatory response, Cell adhesion molecules (CAMs), Leukocyte transendothelial migration, angiogenesis, cell_signaling, immunology, metastasis |
| 0.0034365 | 0.062934 | 816.0839761 | 106.351629 | 7.6734506 | 3460070 | [SPP1](http://www.ncbi.nlm.nih.gov/entrez/query.fcgi?cmd=search&db=gene&term=SPP1) | Regulators of Bone Mineralization, Cell Communication, ECM-receptor interaction, Focal adhesion, immunology |
| 0.0016995 | 0.045404 | 1804.08566 | 235.1816849 | 7.6710296 | 5560195 | [LOC653879](http://www.ncbi.nlm.nih.gov/entrez/query.fcgi?cmd=search&db=gene&term=LOC653879) |  |
| 0.0026875 | 0.05636 | 1755.220704 | 230.8155588 | 7.6044298 | 3940392 | [KIAA0367](http://www.ncbi.nlm.nih.gov/entrez/query.fcgi?cmd=search&db=gene&term=KIAA0367) |  |
| 0.0003748 | 0.024685 | 2791.152086 | 367.9875327 | 7.5849094 | 6350161 | [LCP1](http://www.ncbi.nlm.nih.gov/entrez/query.fcgi?cmd=search&db=gene&term=LCP1) | immunology, misc |
| 0.0005067 | 0.02772 | 1668.826176 | 226.8262227 | 7.3572895 | 7000369 | [CD14](http://www.ncbi.nlm.nih.gov/entrez/query.fcgi?cmd=search&db=gene&term=CD14) | Inactivation of Gsk3 by AKT causes accumulation of b-catenin in Alveolar Macrophages, Toll-Like Receptor Pathway, Hematopoietic cell lineage, MAPK signaling pathway, Regulation of actin cytoskeleton, Toll-like receptor signaling pathway, angiogenesis, immunology, metastasis, misc |
| 6.10E-05 | 0.015962 | 2062.866334 | 280.8757899 | 7.3444078 | 1430278 | [CTSK](http://www.ncbi.nlm.nih.gov/entrez/query.fcgi?cmd=search&db=gene&term=CTSK) | immunology |
| 0.0005193 | 0.02772 | 1689.81749 | 231.7590188 | 7.2912696 | 2260129 | [MS4A6A](http://www.ncbi.nlm.nih.gov/entrez/query.fcgi?cmd=search&db=gene&term=MS4A6A) |  |
| 0.0021179 | 0.050014 | 2005.664866 | 280.5440095 | 7.1491987 | 1580088 | [HCST](http://www.ncbi.nlm.nih.gov/entrez/query.fcgi?cmd=search&db=gene&term=HCST) | Natural killer cell mediated cytotoxicity |
| 0.0005933 | 0.028704 | 6516.381514 | 915.1616312 | 7.1204706 | 5860152 | [CD44](http://www.ncbi.nlm.nih.gov/entrez/query.fcgi?cmd=search&db=gene&term=CD44) | Adhesion Molecules on Lymphocyte, Monocyte and its Surface Molecules, Neutrophil and Its Surface Molecules, ECM-receptor interaction, Hematopoietic cell lineage, cell_signaling, immunology, metastasis |
| 0.0003251 | 0.023725 | 3654.417161 | 513.4760768 | 7.1170154 | 4900731 | [HLA-DMB](http://www.ncbi.nlm.nih.gov/entrez/query.fcgi?cmd=search&db=gene&term=HLA-DMB) | Antigen processing and presentation, Cell adhesion molecules (CAMs), Type I diabetes mellitus, immunology |
| 0.0001268 | 0.018171 | 845.4636727 | 122.2317187 | 6.9168926 | 3370349 | [CFB](http://www.ncbi.nlm.nih.gov/entrez/query.fcgi?cmd=search&db=gene&term=CFB) | Alternative Complement Pathway, Complement Pathway, Complement and coagulation cascades |
| 0.0012404 | 0.039417 | 4455.191867 | 654.0404075 | 6.8117991 | 3370347 | [ADH1A](http://www.ncbi.nlm.nih.gov/entrez/query.fcgi?cmd=search&db=gene&term=ADH1A) | 1- and 2-Methylnaphthalene degradation, Bile acid biosynthesis, Fatty acid metabolism, Glycerolipid metabolism, Glycolysis / Gluconeogenesis, Metabolism of xenobiotics by cytochrome P450, Tyrosine metabolism |
| 0.0014309 | 0.041769 | 837.1879907 | 123.9309935 | 6.7552754 | 6520040 | [C6](http://www.ncbi.nlm.nih.gov/entrez/query.fcgi?cmd=search&db=gene&term=C6) | Alternative Complement Pathway, Cells and Molecules involved in local acute inflammatory response, Classical Complement Pathway, Complement Pathway, Lectin Induced Complement Pathway, Complement and coagulation cascades, immunology |
| 0.0031881 | 0.060634 | 8813.914686 | 1317.104785 | 6.6918857 | 1190039 | [HLA-DPA1](http://www.ncbi.nlm.nih.gov/entrez/query.fcgi?cmd=search&db=gene&term=HLA-DPA1) | Antigen processing and presentation, Cell adhesion molecules (CAMs), Type I diabetes mellitus, immunology |
| 0.0008755 | 0.033692 | 1202.642792 | 183.9790349 | 6.5368469 | 2230241 | [F13A1](http://www.ncbi.nlm.nih.gov/entrez/query.fcgi?cmd=search&db=gene&term=F13A1) | Fibrinolysis Pathway, Complement and coagulation cascades, immunology |
| 0.002925 | 0.058389 | 9277.892428 | 1425.495446 | 6.5085388 | 5310168 | [HLA-B](http://www.ncbi.nlm.nih.gov/entrez/query.fcgi?cmd=search&db=gene&term=HLA-B) | Antigen processing and presentation, Cell adhesion molecules (CAMs), Natural killer cell mediated cytotoxicity, Type I diabetes mellitus, immunology |
| 2.15E-05 | 0.013372 | 1808.409715 | 281.1808939 | 6.4314815 | 580739 | [FBLN1](http://www.ncbi.nlm.nih.gov/entrez/query.fcgi?cmd=search&db=gene&term=FBLN1) | immunology |
| 9.36E-05 | 0.017191 | 1934.791077 | 302.5409929 | 6.3951369 | 4860546 | [CTHRC1](http://www.ncbi.nlm.nih.gov/entrez/query.fcgi?cmd=search&db=gene&term=CTHRC1) |  |
| 0.0020614 | 0.049303 | 1557.580589 | 246.6053316 | 6.3160864 | 7570408 | [CCL5](http://www.ncbi.nlm.nih.gov/entrez/query.fcgi?cmd=search&db=gene&term=CCL5) | The Role of Eosinophils in the Chemokine Network of Allergy, Cytokine-cytokine receptor interaction, Epithelial cell signaling in Helicobacter pylori infection, Toll-like receptor signaling pathway |
| 0.0002389 | 0.021374 | 29232.61733 | 4634.307367 | 6.3078719 | 4060446 | [LOC649150](http://www.ncbi.nlm.nih.gov/entrez/query.fcgi?cmd=search&db=gene&term=LOC649150) |  |
| 4.87E-05 | 0.015375 | 1706.369775 | 270.7765443 | 6.3017636 | 5960762 | [PCDH18](http://www.ncbi.nlm.nih.gov/entrez/query.fcgi?cmd=search&db=gene&term=PCDH18) |  |
| 0.0009544 | 0.034956 | 1625.101782 | 259.1492269 | 6.2709112 | 4120553 | [WISP2](http://www.ncbi.nlm.nih.gov/entrez/query.fcgi?cmd=search&db=gene&term=WISP2) |  |
| 4.61E-05 | 0.015375 | 6527.315744 | 1044.127123 | 6.2514569 | 7510414 | [IGFBP4](http://www.ncbi.nlm.nih.gov/entrez/query.fcgi?cmd=search&db=gene&term=IGFBP4) | Ghrelin: Regulation of Food Intake and Energy Homeostasis |
| 0.0031644 | 0.060507 | 8144.772506 | 1305.726658 | 6.2377317 | 2470114 | [COL6A3](http://www.ncbi.nlm.nih.gov/entrez/query.fcgi?cmd=search&db=gene&term=COL6A3) | Cell Communication, ECM-receptor interaction, Focal adhesion |
| 0.0004315 | 0.02622 | 2139.994256 | 344.0380361 | 6.2202258 | 380424 | [CFH](http://www.ncbi.nlm.nih.gov/entrez/query.fcgi?cmd=search&db=gene&term=CFH) | Complement and coagulation cascades |
| 8.99E-05 | 0.017191 | 13532.45635 | 2177.188907 | 6.2155637 | 6520215 | [ANXA1](http://www.ncbi.nlm.nih.gov/entrez/query.fcgi?cmd=search&db=gene&term=ANXA1) | Corticosteroids and cardioprotection |
| 0.0032165 | 0.060896 | 1321.669246 | 213.5768487 | 6.1882608 | 830019 | [PPARG](http://www.ncbi.nlm.nih.gov/entrez/query.fcgi?cmd=search&db=gene&term=PPARG) | Basic mechanism of action of PPARa, PPARb(d) and PPARg and effects on gene expression, Nuclear Receptors in Lipid Metabolism and Toxicity, Role of PPAR-gamma Coactivators in Obesity and Thermogenesis, Visceral Fat Deposits and the Metabolic Syndrome, PPAR signaling pathway |
| 0.000127 | 0.018171 | 1381.803093 | 225.0581732 | 6.1397597 | 1260040 | [EFEMP1](http://www.ncbi.nlm.nih.gov/entrez/query.fcgi?cmd=search&db=gene&term=EFEMP1) |  |
| 5.20E-05 | 0.015414 | 17555.06067 | 2868.98468 | 6.1189106 | 4780615 | [ANXA2](http://www.ncbi.nlm.nih.gov/entrez/query.fcgi?cmd=search&db=gene&term=ANXA2) |  |
| 0.001797 | 0.046395 | 1435.937255 | 235.0201748 | 6.1098468 | 3850440 | [FCER1G](http://www.ncbi.nlm.nih.gov/entrez/query.fcgi?cmd=search&db=gene&term=FCER1G) | Fc Epsilon Receptor I Signaling in Mast Cells, Fc epsilon RI signaling pathway, Natural killer cell mediated cytotoxicity |
| 0.0002126 | 0.020598 | 976.4298858 | 161.0391436 | 6.0633077 | 990328 | [MS4A7](http://www.ncbi.nlm.nih.gov/entrez/query.fcgi?cmd=search&db=gene&term=MS4A7) |  |
| 0.0017292 | 0.045557 | 1923.872757 | 320.1643073 | 6.009017 | 1430280 | [CEBPA](http://www.ncbi.nlm.nih.gov/entrez/query.fcgi?cmd=search&db=gene&term=CEBPA) | Keratinocyte Differentiation, MAPKinase Signaling Pathway, gene_regulation, transcription |
| 0.0002002 | 0.020451 | 2035.227265 | 339.3142961 | 5.9980593 | 3800047 | [AIF1](http://www.ncbi.nlm.nih.gov/entrez/query.fcgi?cmd=search&db=gene&term=AIF1) | cell_signaling |
| 0.0010915 | 0.037385 | 1579.445213 | 263.8394338 | 5.986388 | 1770152 | [MS4A6A](http://www.ncbi.nlm.nih.gov/entrez/query.fcgi?cmd=search&db=gene&term=MS4A6A) |  |
| 0.0005964 | 0.028704 | 1079.821541 | 180.53436 | 5.9812522 | 2600164 | [THBS2](http://www.ncbi.nlm.nih.gov/entrez/query.fcgi?cmd=search&db=gene&term=THBS2) | Cell Communication, ECM-receptor interaction, Focal adhesion, TGF-beta signaling pathway, angiogenesis, immunology |
| 0.0015377 | 0.043264 | 2054.567993 | 346.1798817 | 5.9349723 | 6200064 | [CECR1](http://www.ncbi.nlm.nih.gov/entrez/query.fcgi?cmd=search&db=gene&term=CECR1) |  |
| 7.60E-06 | 0.012163 | 929.330263 | 157.7422524 | 5.8914479 | 4890292 | [TSHZ2](http://www.ncbi.nlm.nih.gov/entrez/query.fcgi?cmd=search&db=gene&term=TSHZ2) |  |
| 0.0040679 | 0.06788 | 1161.859179 | 199.7912184 | 5.8153666 | 4480035 | [LRRC17](http://www.ncbi.nlm.nih.gov/entrez/query.fcgi?cmd=search&db=gene&term=LRRC17) |  |
| 4.14E-05 | 0.014558 | 1174.143625 | 202.5539352 | 5.7966962 | 5260474 | [C1R](http://www.ncbi.nlm.nih.gov/entrez/query.fcgi?cmd=search&db=gene&term=C1R) | Classical Complement Pathway, Complement Pathway, Complement and coagulation cascades, immunology |
| 1.25E-05 | 0.012163 | 2111.310633 | 364.4626948 | 5.7929403 | 3180039 | [RGL1](http://www.ncbi.nlm.nih.gov/entrez/query.fcgi?cmd=search&db=gene&term=RGL1) |  |
| 0.0007777 | 0.03195 | 13209.09961 | 2289.282167 | 5.7699744 | 6250192 | [COL6A3](http://www.ncbi.nlm.nih.gov/entrez/query.fcgi?cmd=search&db=gene&term=COL6A3) | Cell Communication, ECM-receptor interaction, Focal adhesion |
| 0.0045058 | 0.071346 | 10991.87945 | 1905.80674 | 5.767573 | 1240070 | [CD74](http://www.ncbi.nlm.nih.gov/entrez/query.fcgi?cmd=search&db=gene&term=CD74) | Antigen Processing and Presentation, Antigen processing and presentation, immunology |
| 0.0001214 | 0.018034 | 2999.058446 | 520.5559161 | 5.7612609 | 6590341 | [RAB31](http://www.ncbi.nlm.nih.gov/entrez/query.fcgi?cmd=search&db=gene&term=RAB31) |  |
| 9.09E-05 | 0.017191 | 1776.202044 | 309.2738064 | 5.7431377 | 3170273 | [FER1L3](http://www.ncbi.nlm.nih.gov/entrez/query.fcgi?cmd=search&db=gene&term=FER1L3) |  |
| 0.0007203 | 0.030535 | 2158.10824 | 376.2098819 | 5.7364475 | 3360279 | [CFH](http://www.ncbi.nlm.nih.gov/entrez/query.fcgi?cmd=search&db=gene&term=CFH) | Complement and coagulation cascades |
| 0.0005871 | 0.028704 | 1378.245105 | 240.5168842 | 5.7303466 | 1300408 | [HCLS1](http://www.ncbi.nlm.nih.gov/entrez/query.fcgi?cmd=search&db=gene&term=HCLS1) | Tight junction |
| 0.0001293 | 0.01832 | 23436.54203 | 4094.561837 | 5.7238217 | 940735 | [MGP](http://www.ncbi.nlm.nih.gov/entrez/query.fcgi?cmd=search&db=gene&term=MGP) | immunology |
| 0.0023651 | 0.052605 | 583.2539706 | 102.0685393 | 5.7143364 | 4070241 | [GYG2](http://www.ncbi.nlm.nih.gov/entrez/query.fcgi?cmd=search&db=gene&term=GYG2) |  |
| 0.0020064 | 0.049013 | 1736.05354 | 305.148671 | 5.6892056 | 3520246 | [CXCL14](http://www.ncbi.nlm.nih.gov/entrez/query.fcgi?cmd=search&db=gene&term=CXCL14) | Cytokine-cytokine receptor interaction, Leukocyte transendothelial migration |
| 0.0001671 | 0.019753 | 5090.005601 | 901.649071 | 5.645218 | 730612 | [HTRA1](http://www.ncbi.nlm.nih.gov/entrez/query.fcgi?cmd=search&db=gene&term=HTRA1) |  |
| 0.0021321 | 0.050143 | 1034.63379 | 183.821973 | 5.6284555 | 620717 | [CCL5](http://www.ncbi.nlm.nih.gov/entrez/query.fcgi?cmd=search&db=gene&term=CCL5) | The Role of Eosinophils in the Chemokine Network of Allergy, Cytokine-cytokine receptor interaction, Epithelial cell signaling in Helicobacter pylori infection, Toll-like receptor signaling pathway |
| 0.0003009 | 0.023298 | 2437.776931 | 434.2793499 | 5.6133844 | 50192 | [DARC](http://www.ncbi.nlm.nih.gov/entrez/query.fcgi?cmd=search&db=gene&term=DARC) |  |
| 0.0008964 | 0.03399 | 1834.776 | 327.8538794 | 5.5963224 | 5810746 | [MATN2](http://www.ncbi.nlm.nih.gov/entrez/query.fcgi?cmd=search&db=gene&term=MATN2) |  |
| 0.0002342 | 0.021257 | 17828.44391 | 3187.108012 | 5.5939252 | 540681 | [S100A10](http://www.ncbi.nlm.nih.gov/entrez/query.fcgi?cmd=search&db=gene&term=S100A10) |  |
| 0.0002017 | 0.020451 | 7054.809461 | 1279.674431 | 5.5129721 | 2490195 | [S100A10](http://www.ncbi.nlm.nih.gov/entrez/query.fcgi?cmd=search&db=gene&term=S100A10) |  |
| 8.95E-05 | 0.017191 | 25946.44912 | 4744.574761 | 5.4686564 | 830593 | [VIM](http://www.ncbi.nlm.nih.gov/entrez/query.fcgi?cmd=search&db=gene&term=VIM) | Cell Communication, cell_signaling, metastasis |
| 0.0002575 | 0.022193 | 2023.829125 | 374.3191451 | 5.4066941 | 4120386 | [PCOLCE](http://www.ncbi.nlm.nih.gov/entrez/query.fcgi?cmd=search&db=gene&term=PCOLCE) |  |
| 6.42E-05 | 0.016179 | 1372.561073 | 255.7210994 | 5.3674142 | 5550408 | [TMED3](http://www.ncbi.nlm.nih.gov/entrez/query.fcgi?cmd=search&db=gene&term=TMED3) |  |
| 0.0009259 | 0.034346 | 9717.020608 | 1811.428289 | 5.3642867 | 4830685 | [FBLN1](http://www.ncbi.nlm.nih.gov/entrez/query.fcgi?cmd=search&db=gene&term=FBLN1) | immunology |
| 0.0001962 | 0.020451 | 16056.58172 | 3001.410102 | 5.3496794 | 1430487 | [MGP](http://www.ncbi.nlm.nih.gov/entrez/query.fcgi?cmd=search&db=gene&term=MGP) | immunology |
| 0.0020617 | 0.049303 | 1062.539924 | 199.9918333 | 5.3129166 | 5290608 | [RARRES2](http://www.ncbi.nlm.nih.gov/entrez/query.fcgi?cmd=search&db=gene&term=RARRES2) |  |
| 6.50E-05 | 0.016179 | 3568.085114 | 672.0614673 | 5.3091648 | 6840164 | [GPNMB](http://www.ncbi.nlm.nih.gov/entrez/query.fcgi?cmd=search&db=gene&term=GPNMB) |  |
| 0.0012579 | 0.039541 | 6697.282565 | 1267.165511 | 5.2852469 | 6290168 | [C10orf116](http://www.ncbi.nlm.nih.gov/entrez/query.fcgi?cmd=search&db=gene&term=C10orf116) |  |
| 0.0022653 | 0.051865 | 644.5502507 | 123.3338871 | 5.2260596 | 4900435 | [TNFSF13B](http://www.ncbi.nlm.nih.gov/entrez/query.fcgi?cmd=search&db=gene&term=TNFSF13B) | TACI and BCMA stimulation of B cell immune responses., Cytokine-cytokine receptor interaction |
| 0.0034102 | 0.062729 | 1170.227885 | 226.0455922 | 5.1769551 | 6580711 | [AEBP1](http://www.ncbi.nlm.nih.gov/entrez/query.fcgi?cmd=search&db=gene&term=AEBP1) |  |
| 0.0032702 | 0.061309 | 4909.670449 | 949.3673663 | 5.171518 | 2350066 | [HLA-A](http://www.ncbi.nlm.nih.gov/entrez/query.fcgi?cmd=search&db=gene&term=HLA-A) | immunology |
| 0.0001004 | 0.017673 | 3152.947427 | 609.903168 | 5.1695869 | 4560129 | [LGMN](http://www.ncbi.nlm.nih.gov/entrez/query.fcgi?cmd=search&db=gene&term=LGMN) | Antigen processing and presentation |
| 1.32E-05 | 0.012163 | 1159.22475 | 224.9345221 | 5.1536098 | 1780259 | [CCDC109B](http://www.ncbi.nlm.nih.gov/entrez/query.fcgi?cmd=search&db=gene&term=CCDC109B) |  |
| 6.68E-05 | 0.016179 | 7279.659069 | 1416.878307 | 5.1378153 | 1710369 | [RPL3](http://www.ncbi.nlm.nih.gov/entrez/query.fcgi?cmd=search&db=gene&term=RPL3) | Ribosome |
| 0.0012945 | 0.039905 | 604.5924068 | 118.0879946 | 5.1198465 | 4860494 | [C3](http://www.ncbi.nlm.nih.gov/entrez/query.fcgi?cmd=search&db=gene&term=C3) | Alternative Complement Pathway, Cells and Molecules involved in local acute inflammatory response, Classical Complement Pathway, Complement Pathway, Lectin Induced Complement Pathway, Complement and coagulation cascades, immunology |
| 0.0007755 | 0.03195 | 6756.97485 | 1323.430649 | 5.1056509 | 2450465 | [CYBRD1](http://www.ncbi.nlm.nih.gov/entrez/query.fcgi?cmd=search&db=gene&term=CYBRD1) |  |
| 0.0004131 | 0.025615 | 1825.821889 | 358.2787978 | 5.0960925 | 610152 | [DAB2](http://www.ncbi.nlm.nih.gov/entrez/query.fcgi?cmd=search&db=gene&term=DAB2) |  |
| 0.0016899 | 0.04531 | 3061.264416 | 602.8759201 | 5.0777686 | 4200025 | [OAF](http://www.ncbi.nlm.nih.gov/entrez/query.fcgi?cmd=search&db=gene&term=OAF) |  |
| 0.0013221 | 0.040294 | 1677.91103 | 331.513153 | 5.0613709 | 3450138 | [CTSC](http://www.ncbi.nlm.nih.gov/entrez/query.fcgi?cmd=search&db=gene&term=CTSC) | immunology |
| 0.0009089 | 0.034019 | 1741.234327 | 344.0770412 | 5.0605943 | 3930343 | [ALOX5AP](http://www.ncbi.nlm.nih.gov/entrez/query.fcgi?cmd=search&db=gene&term=ALOX5AP) | Eicosanoid Metabolism |
| 0.0007041 | 0.030252 | 1550.39555 | 306.7713252 | 5.0539129 | 1510538 | [EPB41L3](http://www.ncbi.nlm.nih.gov/entrez/query.fcgi?cmd=search&db=gene&term=EPB41L3) | Tight junction |
| 0.0001167 | 0.018034 | 879.6466558 | 174.3673259 | 5.0447907 | 3390551 | [C14orf78](http://www.ncbi.nlm.nih.gov/entrez/query.fcgi?cmd=search&db=gene&term=C14orf78) |  |
| 0.0006673 | 0.029864 | 1129.268355 | 224.7212531 | 5.0251961 | 70451 | [WAS](http://www.ncbi.nlm.nih.gov/entrez/query.fcgi?cmd=search&db=gene&term=WAS) | Adherens junction, Regulation of actin cytoskeleton, cell_signaling, immunology, signal_transduction |
| 6.38E-05 | 0.016179 | 2940.209506 | 586.2344571 | 5.0154157 | 3460520 | [CCND2](http://www.ncbi.nlm.nih.gov/entrez/query.fcgi?cmd=search&db=gene&term=CCND2) | Cyclins and Cell Cycle Regulation, Cell cycle, Focal adhesion, Jak-STAT signaling pathway, Wnt signaling pathway, cell_cycle |
| 0.0001372 | 0.018886 | 7400.861374 | 1484.935743 | 4.9839607 | 780270 | [TIMP2](http://www.ncbi.nlm.nih.gov/entrez/query.fcgi?cmd=search&db=gene&term=TIMP2) | Inhibition of Matrix Metalloproteinases, angiogenesis |
| 5.16E-05 | 0.015414 | 4994.114304 | 1009.86542 | 4.9453266 | 7570598 | [COL6A1](http://www.ncbi.nlm.nih.gov/entrez/query.fcgi?cmd=search&db=gene&term=COL6A1) | Cell Communication, ECM-receptor interaction, Focal adhesion |
| 0.0001403 | 0.018893 | 925.4827686 | 187.7617534 | 4.9290271 | 1820270 | [ATP8B4](http://www.ncbi.nlm.nih.gov/entrez/query.fcgi?cmd=search&db=gene&term=ATP8B4) |  |
| 0.0006918 | 0.030043 | 2460.659632 | 501.796456 | 4.9037007 | 6180554 | [SVEP1](http://www.ncbi.nlm.nih.gov/entrez/query.fcgi?cmd=search&db=gene&term=SVEP1) |  |
| 4.15E-05 | 0.014558 | 8390.243832 | 1714.085866 | 4.8948795 | 7050019 | [VIM](http://www.ncbi.nlm.nih.gov/entrez/query.fcgi?cmd=search&db=gene&term=VIM) | Cell Communication, cell_signaling, metastasis |
| 0.0006962 | 0.030046 | 2472.606538 | 505.9326993 | 4.8872242 | 1300452 | [LGMN](http://www.ncbi.nlm.nih.gov/entrez/query.fcgi?cmd=search&db=gene&term=LGMN) | Antigen processing and presentation |
| 1.12E-05 | 0.012163 | 777.1789388 | 161.2620102 | 4.8193554 | 5220093 | [HAVCR2](http://www.ncbi.nlm.nih.gov/entrez/query.fcgi?cmd=search&db=gene&term=HAVCR2) |  |
| 1.49E-05 | 0.012729 | 2521.361175 | 525.2584269 | 4.8002298 | 3450072 | [CLIC1](http://www.ncbi.nlm.nih.gov/entrez/query.fcgi?cmd=search&db=gene&term=CLIC1) |  |
| 0.0027721 | 0.057195 | 2014.805936 | 427.6198525 | 4.7116754 | 2350504 | [ECGF1](http://www.ncbi.nlm.nih.gov/entrez/query.fcgi?cmd=search&db=gene&term=ECGF1) | immunology, misc |
| 0.0021573 | 0.050404 | 16936.96835 | 3607.092805 | 4.6954623 | 5290270 | [S100A4](http://www.ncbi.nlm.nih.gov/entrez/query.fcgi?cmd=search&db=gene&term=S100A4) | immunology |
| 0.0013534 | 0.040636 | 4507.298302 | 964.5387142 | 4.6730092 | 3940435 | [EMP1](http://www.ncbi.nlm.nih.gov/entrez/query.fcgi?cmd=search&db=gene&term=EMP1) | tsonc |
| 0.0001462 | 0.019068 | 1149.176893 | 246.2594117 | 4.6665298 | 6280240 | [SH3BGRL3](http://www.ncbi.nlm.nih.gov/entrez/query.fcgi?cmd=search&db=gene&term=SH3BGRL3) |  |
| 0.0006337 | 0.029309 | 5521.239077 | 1187.175013 | 4.6507373 | 2370482 | [GSN](http://www.ncbi.nlm.nih.gov/entrez/query.fcgi?cmd=search&db=gene&term=GSN) | immunology |
| 0.0015558 | 0.04345 | 1341.305081 | 290.6596041 | 4.6146938 | 6860202 | [TKT](http://www.ncbi.nlm.nih.gov/entrez/query.fcgi?cmd=search&db=gene&term=TKT) | Carbon fixation, Pentose phosphate pathway, immunology |
| 0.0008542 | 0.033634 | 762.9295722 | 165.9932959 | 4.5961469 | 150072 | [IRF8](http://www.ncbi.nlm.nih.gov/entrez/query.fcgi?cmd=search&db=gene&term=IRF8) |  |
| 2.71E-05 | 0.014159 | 14830.77934 | 3243.982625 | 4.5717814 | 540075 | [NPC2](http://www.ncbi.nlm.nih.gov/entrez/query.fcgi?cmd=search&db=gene&term=NPC2) |  |
| 0.0045075 | 0.071346 | 1853.13158 | 408.0989727 | 4.5408876 | 1430170 | [FGL2](http://www.ncbi.nlm.nih.gov/entrez/query.fcgi?cmd=search&db=gene&term=FGL2) |  |
| 0.00018 | 0.019879 | 961.3016665 | 211.9883451 | 4.5346911 | 2140239 | [CMTM7](http://www.ncbi.nlm.nih.gov/entrez/query.fcgi?cmd=search&db=gene&term=CMTM7) |  |
| 0.0048623 | 0.073632 | 922.2539436 | 203.6960926 | 4.5275976 | 840678 | [ENPP2](http://www.ncbi.nlm.nih.gov/entrez/query.fcgi?cmd=search&db=gene&term=ENPP2) | Nicotinate and nicotinamide metabolism, Pantothenate and CoA biosynthesis, Purine metabolism, Riboflavin metabolism, Starch and sucrose metabolism |
| 0.0008758 | 0.033692 | 847.2670112 | 187.3486493 | 4.5224079 | 2750356 | [COL6A2](http://www.ncbi.nlm.nih.gov/entrez/query.fcgi?cmd=search&db=gene&term=COL6A2) | Cell Communication, ECM-receptor interaction, Focal adhesion |
| 0.0005385 | 0.028008 | 766.0165927 | 169.8841082 | 4.5090539 | 1940047 | [AIF1](http://www.ncbi.nlm.nih.gov/entrez/query.fcgi?cmd=search&db=gene&term=AIF1) | cell_signaling |
| 0.0011867 | 0.038641 | 741.6272418 | 164.9522003 | 4.496013 | 6280332 | [CXCL16](http://www.ncbi.nlm.nih.gov/entrez/query.fcgi?cmd=search&db=gene&term=CXCL16) |  |
| 0.0024207 | 0.053326 | 945.4714469 | 210.581536 | 4.4898117 | 3780315 | [CDO1](http://www.ncbi.nlm.nih.gov/entrez/query.fcgi?cmd=search&db=gene&term=CDO1) | Cysteine metabolism, Taurine and hypotaurine metabolism |
| 1.32E-05 | 0.012163 | 3019.849157 | 674.6269394 | 4.4763246 | 5360148 | [LIPA](http://www.ncbi.nlm.nih.gov/entrez/query.fcgi?cmd=search&db=gene&term=LIPA) | Alkaloid biosynthesis II, Bile acid biosynthesis, Glycerolipid metabolism |
| 0.0036627 | 0.064645 | 809.9761033 | 182.3565801 | 4.4417158 | 2650382 | [MYH8](http://www.ncbi.nlm.nih.gov/entrez/query.fcgi?cmd=search&db=gene&term=MYH8) |  |
| 0.0008938 | 0.033935 | 1595.60972 | 359.556132 | 4.4377208 | 2030309 | [SERPING1](http://www.ncbi.nlm.nih.gov/entrez/query.fcgi?cmd=search&db=gene&term=SERPING1) | Intrinsic Prothrombin Activation Pathway, Complement and coagulation cascades |
| 0.0036732 | 0.064645 | 2698.818605 | 609.6897277 | 4.4265443 | 2650220 | [FSTL1](http://www.ncbi.nlm.nih.gov/entrez/query.fcgi?cmd=search&db=gene&term=FSTL1) |  |
| 4.71E-05 | 0.015375 | 780.7634787 | 181.5709267 | 4.3000468 | 5050431 | [DCLK1](http://www.ncbi.nlm.nih.gov/entrez/query.fcgi?cmd=search&db=gene&term=DCLK1) |  |
| 0.0011782 | 0.038641 | 14996.714 | 3496.185827 | 4.2894499 | 3990458 | [S100A4](http://www.ncbi.nlm.nih.gov/entrez/query.fcgi?cmd=search&db=gene&term=S100A4) | immunology |
| 0.0003912 | 0.025241 | 531.6108121 | 124.5849525 | 4.2670547 | 5890414 | [FYB](http://www.ncbi.nlm.nih.gov/entrez/query.fcgi?cmd=search&db=gene&term=FYB) | cell_signaling, immunology, signal_transduction |
| 0.0007837 | 0.032019 | 1060.689367 | 249.4485448 | 4.2521369 | 70167 | [LY96](http://www.ncbi.nlm.nih.gov/entrez/query.fcgi?cmd=search&db=gene&term=LY96) | Inactivation of Gsk3 by AKT causes accumulation of b-catenin in Alveolar Macrophages, Toll-Like Receptor Pathway, Toll-like receptor signaling pathway |
| 0.0004256 | 0.02597 | 42130.27782 | 9933.948224 | 4.2410406 | 2100273 | [EEF1A1](http://www.ncbi.nlm.nih.gov/entrez/query.fcgi?cmd=search&db=gene&term=EEF1A1) | West Nile Virus, gene_regulation |
| 0.0002104 | 0.020598 | 7493.107225 | 1777.645054 | 4.2151875 | 5490019 | [GPX3](http://www.ncbi.nlm.nih.gov/entrez/query.fcgi?cmd=search&db=gene&term=GPX3) | Arachidonic acid metabolism, Glutathione metabolism, pharmacology |
| 0.0007359 | 0.030932 | 693.6555087 | 164.5650746 | 4.2150834 | 4610010 | [LILRB5](http://www.ncbi.nlm.nih.gov/entrez/query.fcgi?cmd=search&db=gene&term=LILRB5) |  |
| 9.31E-05 | 0.017191 | 34004.30072 | 8072.600052 | 4.2123108 | 5810328 | [FTL](http://www.ncbi.nlm.nih.gov/entrez/query.fcgi?cmd=search&db=gene&term=FTL) | immunology |
| 3.60E-06 | 0.012163 | 648.6145234 | 154.3504016 | 4.2022212 | 6040494 | [FBLN1](http://www.ncbi.nlm.nih.gov/entrez/query.fcgi?cmd=search&db=gene&term=FBLN1) | immunology |
| 0.0010939 | 0.03739 | 1016.067477 | 242.610207 | 4.1880657 | 4890270 | [LY6E](http://www.ncbi.nlm.nih.gov/entrez/query.fcgi?cmd=search&db=gene&term=LY6E) | cell_cycle, cell_signaling |
| 0.0013541 | 0.040636 | 3918.725141 | 940.9402157 | 4.1646909 | 4010296 | [RNASE1](http://www.ncbi.nlm.nih.gov/entrez/query.fcgi?cmd=search&db=gene&term=RNASE1) |  |
| 0.0009904 | 0.035745 | 672.6400281 | 161.6319498 | 4.1615536 | 4560717 | [MDK](http://www.ncbi.nlm.nih.gov/entrez/query.fcgi?cmd=search&db=gene&term=MDK) |  |
| 0.001504 | 0.04266 | 1974.278034 | 475.4509537 | 4.1524326 | 4070392 | [CYBA](http://www.ncbi.nlm.nih.gov/entrez/query.fcgi?cmd=search&db=gene&term=CYBA) | Leukocyte transendothelial migration, immunology |
| 0.000587 | 0.028704 | 546.5840516 | 132.5110371 | 4.1248191 | 2940685 | [C1QA](http://www.ncbi.nlm.nih.gov/entrez/query.fcgi?cmd=search&db=gene&term=C1QA) | Classical Complement Pathway, Complement Pathway, Complement and coagulation cascades, immunology |
| 0.0005249 | 0.02772 | 1169.375535 | 283.7265585 | 4.1214878 | 6580441 | [SLCO2B1](http://www.ncbi.nlm.nih.gov/entrez/query.fcgi?cmd=search&db=gene&term=SLCO2B1) |  |
| 0.0001004 | 0.017673 | 2954.427092 | 717.2633571 | 4.1190269 | 830278 | [C9orf19](http://www.ncbi.nlm.nih.gov/entrez/query.fcgi?cmd=search&db=gene&term=C9orf19) |  |
| 0.0028819 | 0.058288 | 2990.941251 | 730.5120271 | 4.094308 | 6590041 | [RNASE4](http://www.ncbi.nlm.nih.gov/entrez/query.fcgi?cmd=search&db=gene&term=RNASE4) |  |
| 9.23E-05 | 0.017191 | 3551.599309 | 869.1277244 | 4.0863951 | 5490431 | [SAT1](http://www.ncbi.nlm.nih.gov/entrez/query.fcgi?cmd=search&db=gene&term=SAT1) | Arginine and proline metabolism |
| 0.0003733 | 0.024685 | 5020.957193 | 1230.792057 | 4.0794521 | 1070451 | [ANXA5](http://www.ncbi.nlm.nih.gov/entrez/query.fcgi?cmd=search&db=gene&term=ANXA5) |  |
| 0.0001755 | 0.019771 | 729.0993568 | 178.7724435 | 4.0783654 | 1570092 | [TUBB6](http://www.ncbi.nlm.nih.gov/entrez/query.fcgi?cmd=search&db=gene&term=TUBB6) | Gap junction |
| 0.0008468 | 0.033518 | 811.6252793 | 199.7276702 | 4.0636597 | 6860687 | [PYCARD](http://www.ncbi.nlm.nih.gov/entrez/query.fcgi?cmd=search&db=gene&term=PYCARD) |  |
| 0.0002671 | 0.022554 | 7801.683308 | 1925.97961 | 4.0507611 | 3830131 | [TUBA1C](http://www.ncbi.nlm.nih.gov/entrez/query.fcgi?cmd=search&db=gene&term=TUBA1C) | Gap junction |
| 0.0005171 | 0.02772 | 2551.486794 | 630.6567358 | 4.0457616 | 5090156 | [NBL1](http://www.ncbi.nlm.nih.gov/entrez/query.fcgi?cmd=search&db=gene&term=NBL1) | tsonc |
| 9.63E-05 | 0.017266 | 952.9145507 | 236.4452952 | 4.0301692 | 1570348 | [CCND2](http://www.ncbi.nlm.nih.gov/entrez/query.fcgi?cmd=search&db=gene&term=CCND2) | Cyclins and Cell Cycle Regulation, Cell cycle, Focal adhesion, Jak-STAT signaling pathway, Wnt signaling pathway, cell_cycle |
| 0.0032574 | 0.061248 | 1224.684322 | 304.6867922 | 4.0194861 | 6350189 | [MGC4677](http://www.ncbi.nlm.nih.gov/entrez/query.fcgi?cmd=search&db=gene&term=MGC4677) |  |
| 9.24E-05 | 0.017191 | 5484.576511 | 1364.727766 | 4.0188063 | 7650358 | [TGFBI](http://www.ncbi.nlm.nih.gov/entrez/query.fcgi?cmd=search&db=gene&term=TGFBI) | cell_cycle, cell_signaling, immunology |
| 7.70E-05 | 0.016325 | 3064.260821 | 765.6439987 | 4.0022005 | 5820129 | [PABPC1](http://www.ncbi.nlm.nih.gov/entrez/query.fcgi?cmd=search&db=gene&term=PABPC1) | Regulation of eIF4e and p70 S6 Kinase |
| 0.001721 | 0.045525 | 2218.054234 | 555.2766065 | 3.9945033 | 4490577 | [TUBA1A](http://www.ncbi.nlm.nih.gov/entrez/query.fcgi?cmd=search&db=gene&term=TUBA1A) | Gap junction |
| 0.0003016 | 0.023298 | 1102.840514 | 276.3130011 | 3.9912726 | 1070544 | [EFHD2](http://www.ncbi.nlm.nih.gov/entrez/query.fcgi?cmd=search&db=gene&term=EFHD2) |  |
| 0.0008685 | 0.033692 | 6248.365717 | 1567.144091 | 3.9871035 | 4560020 | [CXCL12](http://www.ncbi.nlm.nih.gov/entrez/query.fcgi?cmd=search&db=gene&term=CXCL12) | CXCR4 Signaling Pathway, Pertussis toxin-insensitive CCR5 Signaling in Macrophage, Axon guidance, Cytokine-cytokine receptor interaction, Leukocyte transendothelial migration |
| 0.0003804 | 0.024885 | 1259.581245 | 316.0821136 | 3.9849811 | 3890551 | [CMTM3](http://www.ncbi.nlm.nih.gov/entrez/query.fcgi?cmd=search&db=gene&term=CMTM3) |  |
| 0.0010347 | 0.036641 | 6441.971028 | 1617.02742 | 3.9838354 | 3440670 | [LOC402251](http://www.ncbi.nlm.nih.gov/entrez/query.fcgi?cmd=search&db=gene&term=LOC402251) |  |
| 0.0031562 | 0.060421 | 2062.195537 | 520.8630357 | 3.9591896 | 6840192 | [ECM2](http://www.ncbi.nlm.nih.gov/entrez/query.fcgi?cmd=search&db=gene&term=ECM2) |  |
| 0.0001732 | 0.019771 | 832.045222 | 211.6915956 | 3.9304594 | 4830632 | [SLC7A7](http://www.ncbi.nlm.nih.gov/entrez/query.fcgi?cmd=search&db=gene&term=SLC7A7) | immunology |
| 0.0025472 | 0.054641 | 1936.001999 | 494.1262716 | 3.9180309 | 2070170 | [UBE2L6](http://www.ncbi.nlm.nih.gov/entrez/query.fcgi?cmd=search&db=gene&term=UBE2L6) | Role of Parkin in the Ubiquitin-Proteasomal Pathway, Parkinson\'s disease |
| 0.0002428 | 0.021435 | 955.4351622 | 243.9121983 | 3.9171274 | 5390333 | [TMEM98](http://www.ncbi.nlm.nih.gov/entrez/query.fcgi?cmd=search&db=gene&term=TMEM98) |  |
| 0.0006785 | 0.030043 | 1582.135986 | 405.0905983 | 3.9056349 | 4880537 | [LIMA1](http://www.ncbi.nlm.nih.gov/entrez/query.fcgi?cmd=search&db=gene&term=LIMA1) |  |
| 0.0035453 | 0.063738 | 1290.339473 | 330.5061104 | 3.904132 | 2100767 | [COLEC12](http://www.ncbi.nlm.nih.gov/entrez/query.fcgi?cmd=search&db=gene&term=COLEC12) |  |
| 0.0039045 | 0.06667 | 554.9255346 | 142.1762055 | 3.903083 | 4480364 | [FOLR2](http://www.ncbi.nlm.nih.gov/entrez/query.fcgi?cmd=search&db=gene&term=FOLR2) |  |
| 0.00369 | 0.064645 | 676.0048906 | 173.3522013 | 3.8996037 | 3780095 | [AOX1](http://www.ncbi.nlm.nih.gov/entrez/query.fcgi?cmd=search&db=gene&term=AOX1) | Nicotinate and nicotinamide metabolism, Tryptophan metabolism, Tyrosine metabolism, Valine, leucine and isoleucine degradation, Vitamin B6 metabolism, misc |
| 0.0001605 | 0.019731 | 1113.065996 | 285.6830341 | 3.8961571 | 240086 | [PHGDH](http://www.ncbi.nlm.nih.gov/entrez/query.fcgi?cmd=search&db=gene&term=PHGDH) | Glycine, serine and threonine metabolism |
| 1.28E-05 | 0.012163 | 804.9172199 | 208.5112306 | 3.8603063 | 3990433 | [CENTA2](http://www.ncbi.nlm.nih.gov/entrez/query.fcgi?cmd=search&db=gene&term=CENTA2) |  |
| 6.05E-05 | 0.015962 | 3437.899916 | 897.5581877 | 3.8302808 | 6060484 | [MARCKS](http://www.ncbi.nlm.nih.gov/entrez/query.fcgi?cmd=search&db=gene&term=MARCKS) | Effects of calcineurin in Keratinocyte Differentiation |
| 0.0011096 | 0.037691 | 1572.587285 | 410.696373 | 3.8290752 | 3400019 | [RGS2](http://www.ncbi.nlm.nih.gov/entrez/query.fcgi?cmd=search&db=gene&term=RGS2) | immunology |
| 0.001635 | 0.044476 | 838.4802355 | 220.2844712 | 3.806352 | 1400121 | [SLCO2B1](http://www.ncbi.nlm.nih.gov/entrez/query.fcgi?cmd=search&db=gene&term=SLCO2B1) |  |
| 0.0033188 | 0.061791 | 3361.877989 | 884.2004779 | 3.8021671 | 4070736 | [TAGLN2](http://www.ncbi.nlm.nih.gov/entrez/query.fcgi?cmd=search&db=gene&term=TAGLN2) |  |
| 0.0042836 | 0.069391 | 653.641043 | 172.1908784 | 3.796026 | 4230626 | [PRKCD](http://www.ncbi.nlm.nih.gov/entrez/query.fcgi?cmd=search&db=gene&term=PRKCD) | HIV-I Nef: negative effector of Fas and TNF, Fc epsilon RI signaling pathway, GnRH signaling pathway, Tight junction, Type II diabetes mellitus |
| 0.0006061 | 0.028845 | 725.1052379 | 191.198848 | 3.7924143 | 1820722 | [APBB1IP](http://www.ncbi.nlm.nih.gov/entrez/query.fcgi?cmd=search&db=gene&term=APBB1IP) |  |
| 0.0016926 | 0.04531 | 7552.329149 | 1995.305914 | 3.7850482 | 1230767 | [IFITM2](http://www.ncbi.nlm.nih.gov/entrez/query.fcgi?cmd=search&db=gene&term=IFITM2) |  |
| 2.26E-05 | 0.013372 | 37648.35019 | 9954.406486 | 3.7820788 | 4920767 | [FTL](http://www.ncbi.nlm.nih.gov/entrez/query.fcgi?cmd=search&db=gene&term=FTL) | immunology |
| 0.0005181 | 0.02772 | 888.5848971 | 235.621144 | 3.7712443 | 3870594 | [IFI16](http://www.ncbi.nlm.nih.gov/entrez/query.fcgi?cmd=search&db=gene&term=IFI16) | cell_signaling |
| 7.46E-05 | 0.016325 | 556.3385106 | 147.8130463 | 3.7637984 | 5360376 | [LAPTM5](http://www.ncbi.nlm.nih.gov/entrez/query.fcgi?cmd=search&db=gene&term=LAPTM5) |  |
| 0.0002749 | 0.022619 | 635.712684 | 168.9953716 | 3.7617165 | 4290730 | [LGALS3BP](http://www.ncbi.nlm.nih.gov/entrez/query.fcgi?cmd=search&db=gene&term=LGALS3BP) | immunology |
| 0.0023149 | 0.052324 | 1225.894201 | 327.699717 | 3.7409071 | 7160253 | [LAMA4](http://www.ncbi.nlm.nih.gov/entrez/query.fcgi?cmd=search&db=gene&term=LAMA4) | Cell Communication, ECM-receptor interaction, Focal adhesion, angiogenesis |
| 0.0021158 | 0.050014 | 1945.826348 | 523.3842131 | 3.7177781 | 2850100 | [RNASET2](http://www.ncbi.nlm.nih.gov/entrez/query.fcgi?cmd=search&db=gene&term=RNASET2) |  |
| 8.29E-05 | 0.016957 | 5103.92156 | 1374.071218 | 3.714452 | 620300 | [LASP1](http://www.ncbi.nlm.nih.gov/entrez/query.fcgi?cmd=search&db=gene&term=LASP1) |  |
| 3.50E-05 | 0.014558 | 1187.909978 | 320.2265092 | 3.7095929 | 3460008 | [TMEM173](http://www.ncbi.nlm.nih.gov/entrez/query.fcgi?cmd=search&db=gene&term=TMEM173) |  |
| 0.0001241 | 0.018034 | 901.7362436 | 243.1121958 | 3.7091362 | 7650017 | [SERPINH1](http://www.ncbi.nlm.nih.gov/entrez/query.fcgi?cmd=search&db=gene&term=SERPINH1) |  |
| 0.0004861 | 0.027279 | 958.6483362 | 258.5090518 | 3.7083743 | 7320204 | [C1QTNF5](http://www.ncbi.nlm.nih.gov/entrez/query.fcgi?cmd=search&db=gene&term=C1QTNF5) |  |
| 0.0015087 | 0.042751 | 1581.047108 | 427.3469488 | 3.6996803 | 3390458 | [COL6A2](http://www.ncbi.nlm.nih.gov/entrez/query.fcgi?cmd=search&db=gene&term=COL6A2) | Cell Communication, ECM-receptor interaction, Focal adhesion |
| 0.0026307 | 0.055555 | 2712.570391 | 739.4868722 | 3.6681792 | 7550484 | [SRGN](http://www.ncbi.nlm.nih.gov/entrez/query.fcgi?cmd=search&db=gene&term=SRGN) |  |
| 0.0009531 | 0.034956 | 13951.08101 | 3807.749089 | 3.6638656 | 7650142 | [TMSB10](http://www.ncbi.nlm.nih.gov/entrez/query.fcgi?cmd=search&db=gene&term=TMSB10) |  |
| 0.0016079 | 0.044182 | 475.9846826 | 130.7293816 | 3.6409924 | 6590538 | [TF](http://www.ncbi.nlm.nih.gov/entrez/query.fcgi?cmd=search&db=gene&term=TF) | The role of FYVE-finger proteins in vesicle transport, immunology |
| 3.30E-06 | 0.012163 | 3268.908342 | 901.1134054 | 3.6276326 | 6580474 | [TUBB](http://www.ncbi.nlm.nih.gov/entrez/query.fcgi?cmd=search&db=gene&term=TUBB) | Gap junction, immunology |
| 0.0035866 | 0.063988 | 849.1530581 | 234.112105 | 3.6271215 | 670255 | [GADD45A](http://www.ncbi.nlm.nih.gov/entrez/query.fcgi?cmd=search&db=gene&term=GADD45A) | ATM Signaling Pathway, Cell Cycle: G2/M Checkpoint, Hypoxia and p53 in the Cardiovascular system, p53 Signaling Pathway, Cell cycle, MAPK signaling pathway |
| 0.0004909 | 0.027279 | 537.4399957 | 148.25745 | 3.6250455 | 1990278 | [FCGR2A](http://www.ncbi.nlm.nih.gov/entrez/query.fcgi?cmd=search&db=gene&term=FCGR2A) | immunology |
| 0.0003547 | 0.024601 | 3825.006037 | 1055.653397 | 3.6233541 | 5490070 | [TGFBR2](http://www.ncbi.nlm.nih.gov/entrez/query.fcgi?cmd=search&db=gene&term=TGFBR2) | ALK in cardiac myocytes, NFkB activation by Nontypeable Hemophilus influenzae, TGF beta signaling pathway, Adherens junction, Colorectal cancer, Cytokine-cytokine receptor interaction, MAPK signaling pathway, TGF-beta signaling pathway, angiogenesis, tsonc |
| 0.0013513 | 0.040636 | 44183.27269 | 12196.97023 | 3.6224793 | 3850121 | [EEF1A1](http://www.ncbi.nlm.nih.gov/entrez/query.fcgi?cmd=search&db=gene&term=EEF1A1) | West Nile Virus, gene_regulation |
| 0.0006285 | 0.029291 | 745.5802723 | 206.9958054 | 3.6019101 | 4560193 | [CD44](http://www.ncbi.nlm.nih.gov/entrez/query.fcgi?cmd=search&db=gene&term=CD44) | Adhesion Molecules on Lymphocyte, Monocyte and its Surface Molecules, Neutrophil and Its Surface Molecules, ECM-receptor interaction, Hematopoietic cell lineage, cell_signaling, immunology, metastasis |
| 0.0001753 | 0.019771 | 728.5980484 | 202.7305368 | 3.5939235 | 2940746 | [NNMT](http://www.ncbi.nlm.nih.gov/entrez/query.fcgi?cmd=search&db=gene&term=NNMT) | Nicotinate and nicotinamide metabolism, immunology |
| 0.0006828 | 0.030043 | 703.8995017 | 195.9504983 | 3.5922312 | 7560037 | [KIAA1949](http://www.ncbi.nlm.nih.gov/entrez/query.fcgi?cmd=search&db=gene&term=KIAA1949) |  |
| 0.0034204 | 0.062837 | 572.6130616 | 159.9947833 | 3.5789483 | 5700725 | [EPSTI1](http://www.ncbi.nlm.nih.gov/entrez/query.fcgi?cmd=search&db=gene&term=EPSTI1) |  |
| 6.62E-05 | 0.016179 | 5257.076291 | 1469.056319 | 3.5785397 | 6480630 | [ATP9A](http://www.ncbi.nlm.nih.gov/entrez/query.fcgi?cmd=search&db=gene&term=ATP9A) |  |
| 0.001213 | 0.038949 | 1765.905781 | 493.8667461 | 3.5756726 | 5820601 | [CCND1](http://www.ncbi.nlm.nih.gov/entrez/query.fcgi?cmd=search&db=gene&term=CCND1) | BTG family proteins and cell cycle regulation, CARM1 and Regulation of the Estrogen Receptor, Cell Cycle: G1/S Check Point , Cyclins and Cell Cycle Regulation, Inactivation of Gsk3 by AKT causes accumulation of b-catenin in Alveolar Macrophages, Influence of Ras and Rho proteins on G1 to S Transition, p53 Signaling Pathway, WNT Signaling Pathway, Cell cycle, Colorectal cancer, Focal adhesion, Jak-STAT signaling pathway, Wnt signaling pathway, cell_cycle, tsonc |
| 0.0005101 | 0.02772 | 933.5647226 | 261.3170347 | 3.5725368 | 4760338 | [CDC25B](http://www.ncbi.nlm.nih.gov/entrez/query.fcgi?cmd=search&db=gene&term=CDC25B) | Cell cycle, MAPK signaling pathway |
| 0.0011758 | 0.038633 | 1125.855169 | 315.7320426 | 3.5658565 | 2030132 | [LOC653506](http://www.ncbi.nlm.nih.gov/entrez/query.fcgi?cmd=search&db=gene&term=LOC653506) |  |
| 5.80E-06 | 0.012163 | 1815.551802 | 511.0661363 | 3.5524792 | 2640377 | [PGCP](http://www.ncbi.nlm.nih.gov/entrez/query.fcgi?cmd=search&db=gene&term=PGCP) |  |
| 0.0032959 | 0.061599 | 1509.096619 | 425.25086 | 3.5487209 | 5290348 | [SGCE](http://www.ncbi.nlm.nih.gov/entrez/query.fcgi?cmd=search&db=gene&term=SGCE) |  |
| 0.002026 | 0.04919 | 3979.494573 | 1121.856461 | 3.5472404 | 4640743 | [CNN3](http://www.ncbi.nlm.nih.gov/entrez/query.fcgi?cmd=search&db=gene&term=CNN3) |  |
| 1.21E-05 | 0.012163 | 916.058463 | 258.6891835 | 3.5411549 | 1570154 | [GPNMB](http://www.ncbi.nlm.nih.gov/entrez/query.fcgi?cmd=search&db=gene&term=GPNMB) |  |
| 6.20E-06 | 0.012163 | 21289.25225 | 6042.78228 | 3.5230878 | 610440 | [CD81](http://www.ncbi.nlm.nih.gov/entrez/query.fcgi?cmd=search&db=gene&term=CD81) | B cell receptor signaling pathway, immunology |
| 0.0015973 | 0.044064 | 423.6640308 | 120.4194382 | 3.5182362 | 5310402 | [FPR3](http://www.ncbi.nlm.nih.gov/entrez/query.fcgi?cmd=search&db=gene&term=FPR3) | Neuroactive ligand-receptor interaction |
| 0.0014492 | 0.042008 | 525.8497196 | 149.6235299 | 3.5144855 | 6280167 | [LOC401115](http://www.ncbi.nlm.nih.gov/entrez/query.fcgi?cmd=search&db=gene&term=LOC401115) |  |
| 5.55E-05 | 0.0155 | 851.7559569 | 242.388651 | 3.5140092 | 6400647 | [EHD4](http://www.ncbi.nlm.nih.gov/entrez/query.fcgi?cmd=search&db=gene&term=EHD4) |  |
| 0.0040023 | 0.067546 | 3987.211653 | 1136.014654 | 3.5098241 | 7380719 | [IGFBP6](http://www.ncbi.nlm.nih.gov/entrez/query.fcgi?cmd=search&db=gene&term=IGFBP6) | Ghrelin: Regulation of Food Intake and Energy Homeostasis |
| 5.09E-05 | 0.015414 | 1464.346766 | 417.5426591 | 3.5070591 | 7100010 | [NBL1](http://www.ncbi.nlm.nih.gov/entrez/query.fcgi?cmd=search&db=gene&term=NBL1) | tsonc |
| 0.0003384 | 0.024302 | 18966.72635 | 5418.735996 | 3.5002123 | 4610390 | [ACTB](http://www.ncbi.nlm.nih.gov/entrez/query.fcgi?cmd=search&db=gene&term=ACTB) | Chromatin Remodeling by hSWI/SNF ATP-dependent Complexes, Adherens junction, Cell Communication, Focal adhesion, Leukocyte transendothelial migration, Regulation of actin cytoskeleton, Tight junction |
| 0.001947 | 0.048417 | 2333.046341 | 667.4171084 | 3.4956346 | 3800050 | [ADCY3](http://www.ncbi.nlm.nih.gov/entrez/query.fcgi?cmd=search&db=gene&term=ADCY3) | Calcium signaling pathway, Gap junction, GnRH signaling pathway, Olfactory transduction, Purine metabolism |
| 0.0038623 | 0.066183 | 398.2597445 | 114.1882036 | 3.4877486 | 3400296 | [CRABP2](http://www.ncbi.nlm.nih.gov/entrez/query.fcgi?cmd=search&db=gene&term=CRABP2) |  |
| 0.0028575 | 0.058121 | 628.7552673 | 180.3702677 | 3.4859141 | 2490152 | [ENPP2](http://www.ncbi.nlm.nih.gov/entrez/query.fcgi?cmd=search&db=gene&term=ENPP2) | Nicotinate and nicotinamide metabolism, Pantothenate and CoA biosynthesis, Purine metabolism, Riboflavin metabolism, Starch and sucrose metabolism |
| 3.95E-05 | 0.014558 | 3651.875517 | 1050.328159 | 3.47689 | 730332 | [MSN](http://www.ncbi.nlm.nih.gov/entrez/query.fcgi?cmd=search&db=gene&term=MSN) | Leukocyte transendothelial migration, Regulation of actin cytoskeleton |
| 0.0001181 | 0.018034 | 19686.43847 | 5673.654146 | 3.4697988 | 5340017 | [H19](http://www.ncbi.nlm.nih.gov/entrez/query.fcgi?cmd=search&db=gene&term=H19) |  |
| 0.0004836 | 0.027274 | 512.2980926 | 147.7747001 | 3.466751 | 6510524 | [PSCD4](http://www.ncbi.nlm.nih.gov/entrez/query.fcgi?cmd=search&db=gene&term=PSCD4) |  |
| 4.24E-05 | 0.014661 | 645.9573939 | 186.780803 | 3.4583714 | 1780273 | [ALOX5](http://www.ncbi.nlm.nih.gov/entrez/query.fcgi?cmd=search&db=gene&term=ALOX5) | Eicosanoid Metabolism, Arachidonic acid metabolism, Linoleic acid metabolism, cell_signaling, immunology, signal_transduction |
| 0.0001405 | 0.018893 | 1621.533817 | 470.2746862 | 3.4480568 | 4560328 | [FSCN1](http://www.ncbi.nlm.nih.gov/entrez/query.fcgi?cmd=search&db=gene&term=FSCN1) |  |
| 0.0001606 | 0.019731 | 793.5269392 | 230.2608061 | 3.4462093 | 4150689 | [METRNL](http://www.ncbi.nlm.nih.gov/entrez/query.fcgi?cmd=search&db=gene&term=METRNL) |  |
| 0.000288 | 0.022818 | 1350.465408 | 392.6866405 | 3.4390409 | 160731 | [SHISA5](http://www.ncbi.nlm.nih.gov/entrez/query.fcgi?cmd=search&db=gene&term=SHISA5) |  |
| 0.0005197 | 0.02772 | 4679.265686 | 1363.039564 | 3.432964 | 5960068 | [PDGFRB](http://www.ncbi.nlm.nih.gov/entrez/query.fcgi?cmd=search&db=gene&term=PDGFRB) | Calcium signaling pathway, Colorectal cancer, Cytokine-cytokine receptor interaction, Focal adhesion, Gap junction, MAPK signaling pathway, Regulation of actin cytoskeleton, cell_cycle, cell_signaling, immunology, misc, signal_transduction |
| 0.0046302 | 0.072259 | 450.1793184 | 131.557491 | 3.4219208 | 7400368 | [RUNX1](http://www.ncbi.nlm.nih.gov/entrez/query.fcgi?cmd=search&db=gene&term=RUNX1) |  |
| 0.0017336 | 0.045557 | 635.5088004 | 186.5629765 | 3.4064036 | 4920202 | [MAP1A](http://www.ncbi.nlm.nih.gov/entrez/query.fcgi?cmd=search&db=gene&term=MAP1A) |  |
| 4.00E-07 | 0.002324 | 4486.804435 | 1319.213673 | 3.4011203 | 1660300 | [PRCP](http://www.ncbi.nlm.nih.gov/entrez/query.fcgi?cmd=search&db=gene&term=PRCP) |  |
| 0.0024372 | 0.053385 | 1796.903748 | 528.8972879 | 3.3974531 | 60136 | [CPVL](http://www.ncbi.nlm.nih.gov/entrez/query.fcgi?cmd=search&db=gene&term=CPVL) |  |
| 0.000161 | 0.019731 | 439.2741736 | 129.4522402 | 3.39333 | 3940390 | [TBXAS1](http://www.ncbi.nlm.nih.gov/entrez/query.fcgi?cmd=search&db=gene&term=TBXAS1) | Aspirin Blocks Signaling Pathway Involved in Platelet Activation, Eicosanoid Metabolism, Arachidonic acid metabolism, immunology |
| 0.0001447 | 0.019068 | 5429.331998 | 1609.477188 | 3.3733513 | 1070754 | [CAP1](http://www.ncbi.nlm.nih.gov/entrez/query.fcgi?cmd=search&db=gene&term=CAP1) | How Progesterone Initiates the Oocyte Maturation |
| 0.0020751 | 0.049403 | 2428.040648 | 721.2651752 | 3.3663633 | 3800452 | [EMP3](http://www.ncbi.nlm.nih.gov/entrez/query.fcgi?cmd=search&db=gene&term=EMP3) |  |
| 3.23E-05 | 0.014558 | 4652.373941 | 1385.838766 | 3.3570817 | 6620136 | [MYH9](http://www.ncbi.nlm.nih.gov/entrez/query.fcgi?cmd=search&db=gene&term=MYH9) | Regulation of actin cytoskeleton, Tight junction |
| 0.0044482 | 0.070963 | 870.3546265 | 259.7675041 | 3.3505139 | 4540682 | [PALM](http://www.ncbi.nlm.nih.gov/entrez/query.fcgi?cmd=search&db=gene&term=PALM) |  |
| 0.0027534 | 0.057123 | 1591.306325 | 475.4518966 | 3.3469344 | 6420008 | [PROS1](http://www.ncbi.nlm.nih.gov/entrez/query.fcgi?cmd=search&db=gene&term=PROS1) | Acute Myocardial Infarction, Extrinsic Prothrombin Activation Pathway, Intrinsic Prothrombin Activation Pathway, Complement and coagulation cascades, immunology |
| 0.0002827 | 0.022708 | 7981.366106 | 2387.958662 | 3.3423385 | 6650242 | [IFITM3](http://www.ncbi.nlm.nih.gov/entrez/query.fcgi?cmd=search&db=gene&term=IFITM3) |  |
| 1.82E-05 | 0.012893 | 6791.312873 | 2046.272599 | 3.3188701 | 6380441 | [ENO1](http://www.ncbi.nlm.nih.gov/entrez/query.fcgi?cmd=search&db=gene&term=ENO1) | Glycolysis Pathway, Glycolysis / Gluconeogenesis, Phenylalanine, tyrosine and tryptophan biosynthesis |
| 0.0034446 | 0.063016 | 440.0001693 | 132.6845662 | 3.3161368 | 990458 | [SLIT3](http://www.ncbi.nlm.nih.gov/entrez/query.fcgi?cmd=search&db=gene&term=SLIT3) | Axon guidance |
| 0.0006951 | 0.030043 | 480.1347234 | 144.8417318 | 3.3148922 | 1980059 | [RASSF2](http://www.ncbi.nlm.nih.gov/entrez/query.fcgi?cmd=search&db=gene&term=RASSF2) |  |
| 0.0008562 | 0.033634 | 497.3489512 | 150.4017661 | 3.3068026 | 5810678 | [RENBP](http://www.ncbi.nlm.nih.gov/entrez/query.fcgi?cmd=search&db=gene&term=RENBP) | Aminosugars metabolism, gene_regulation, immunology, transcription |
| 0.0002383 | 0.021374 | 1492.951919 | 452.8097017 | 3.2970847 | 7000382 | [PQLC3](http://www.ncbi.nlm.nih.gov/entrez/query.fcgi?cmd=search&db=gene&term=PQLC3) |  |
| 0.000366 | 0.024601 | 7166.011395 | 2175.015359 | 3.2946946 | 1570193 | [ARHGDIB](http://www.ncbi.nlm.nih.gov/entrez/query.fcgi?cmd=search&db=gene&term=ARHGDIB) | Caspase Cascade in Apoptosis, D4-GDI Signaling Pathway, FAS signaling pathway ( CD95 ), HIV-I Nef: negative effector of Fas and TNF, TNFR1 Signaling Pathway, cell_signaling, misc, signal_transduction |
| 0.0035908 | 0.064024 | 1638.621577 | 498.1796959 | 3.2892179 | 3370202 | [ANXA2P1](http://www.ncbi.nlm.nih.gov/entrez/query.fcgi?cmd=search&db=gene&term=ANXA2P1) |  |
| 0.0017915 | 0.046294 | 580.2512382 | 176.6578815 | 3.2846043 | 6660398 | [FCN1](http://www.ncbi.nlm.nih.gov/entrez/query.fcgi?cmd=search&db=gene&term=FCN1) | immunology |
| 9.82E-05 | 0.017498 | 2111.720297 | 643.9915939 | 3.2791116 | 5890242 | [SH3BGRL](http://www.ncbi.nlm.nih.gov/entrez/query.fcgi?cmd=search&db=gene&term=SH3BGRL) |  |
| 0.0005341 | 0.027911 | 1213.495795 | 370.3801934 | 3.2763518 | 3460161 | [RAB3IL1](http://www.ncbi.nlm.nih.gov/entrez/query.fcgi?cmd=search&db=gene&term=RAB3IL1) |  |
| 0.0010093 | 0.036217 | 1081.277964 | 331.1914165 | 3.2648128 | 4640209 | [OLFML2A](http://www.ncbi.nlm.nih.gov/entrez/query.fcgi?cmd=search&db=gene&term=OLFML2A) |  |
| 4.00E-07 | 0.002324 | 865.3246685 | 266.0518177 | 3.2524667 | 5390095 | [RASSF2](http://www.ncbi.nlm.nih.gov/entrez/query.fcgi?cmd=search&db=gene&term=RASSF2) |  |
| 0.0002399 | 0.021374 | 606.1971604 | 186.5955063 | 3.2487233 | 7150253 | [PPM1M](http://www.ncbi.nlm.nih.gov/entrez/query.fcgi?cmd=search&db=gene&term=PPM1M) |  |
| 0.0005408 | 0.028008 | 536.6689083 | 165.7572339 | 3.2376802 | 6370364 | [MGC87042](http://www.ncbi.nlm.nih.gov/entrez/query.fcgi?cmd=search&db=gene&term=MGC87042) |  |
| 0.0037774 | 0.065289 | 6633.162075 | 2050.479637 | 3.2349319 | 1580576 | [ITM2A](http://www.ncbi.nlm.nih.gov/entrez/query.fcgi?cmd=search&db=gene&term=ITM2A) |  |
| 0.0044564 | 0.070963 | 4390.543923 | 1361.990055 | 3.2236241 | 3990170 | [IFI27](http://www.ncbi.nlm.nih.gov/entrez/query.fcgi?cmd=search&db=gene&term=IFI27) |  |
| 0.0003636 | 0.024601 | 1248.392611 | 387.5153698 | 3.2215306 | 1940021 | [GRN](http://www.ncbi.nlm.nih.gov/entrez/query.fcgi?cmd=search&db=gene&term=GRN) | Proepithelin Conversion to Epithelin and Wound Repair Control |
| 0.0043585 | 0.070235 | 382.657135 | 119.2489696 | 3.2088926 | 3420612 | [GZMA](http://www.ncbi.nlm.nih.gov/entrez/query.fcgi?cmd=search&db=gene&term=GZMA) | Granzyme A mediated Apoptosis Pathway, Neuroactive ligand-receptor interaction, immunology, misc |
| 0.0011518 | 0.038243 | 425.0782957 | 132.8554462 | 3.1995549 | 6860193 | [RTN1](http://www.ncbi.nlm.nih.gov/entrez/query.fcgi?cmd=search&db=gene&term=RTN1) | angiogenesis, metastasis |
| 0.0024551 | 0.053737 | 627.6300212 | 196.5091322 | 3.1938975 | 160092 | [HSPA6](http://www.ncbi.nlm.nih.gov/entrez/query.fcgi?cmd=search&db=gene&term=HSPA6) | immunology |
| 0.0018714 | 0.04743 | 19146.59343 | 6020.52507 | 3.1802199 | 5570132 | [ACTB](http://www.ncbi.nlm.nih.gov/entrez/query.fcgi?cmd=search&db=gene&term=ACTB) | Chromatin Remodeling by hSWI/SNF ATP-dependent Complexes, Adherens junction, Cell Communication, Focal adhesion, Leukocyte transendothelial migration, Regulation of actin cytoskeleton, Tight junction |
| 0.0023335 | 0.052364 | 1318.467411 | 414.8763858 | 3.1779765 | 360500 | [SRGN](http://www.ncbi.nlm.nih.gov/entrez/query.fcgi?cmd=search&db=gene&term=SRGN) |  |
| 0.0018875 | 0.047672 | 711.7641649 | 224.4772826 | 3.1707626 | 4590390 | [FAM38A](http://www.ncbi.nlm.nih.gov/entrez/query.fcgi?cmd=search&db=gene&term=FAM38A) |  |
| 0.0016712 | 0.045114 | 7098.259523 | 2239.237826 | 3.1699445 | 4180343 | [YWHAZ](http://www.ncbi.nlm.nih.gov/entrez/query.fcgi?cmd=search&db=gene&term=YWHAZ) | Cell cycle |
| 0.0036425 | 0.064628 | 27069.72131 | 8546.762001 | 3.1672488 | 3400438 | [HLA-A](http://www.ncbi.nlm.nih.gov/entrez/query.fcgi?cmd=search&db=gene&term=HLA-A) | immunology |
| 0.0027107 | 0.056601 | 2008.125103 | 634.0464558 | 3.1671577 | 2000022 | [IRF9](http://www.ncbi.nlm.nih.gov/entrez/query.fcgi?cmd=search&db=gene&term=IRF9) | Bone Remodelling, IFN alpha signaling pathway, Jak-STAT signaling pathway |
| 0.0013055 | 0.039956 | 436.7763707 | 137.9641184 | 3.1658693 | 1710070 | [ITGAM](http://www.ncbi.nlm.nih.gov/entrez/query.fcgi?cmd=search&db=gene&term=ITGAM) | Adhesion and Diapedesis of Granulocytes, Monocyte and its Surface Molecules, Neutrophil and Its Surface Molecules, Cell adhesion molecules (CAMs), Hematopoietic cell lineage, Leukocyte transendothelial migration, Regulation of actin cytoskeleton, cell_signaling, immunology, metastasis |
| 0.0004496 | 0.026228 | 549.9763259 | 173.9694022 | 3.1613394 | 3180070 | [OLFML3](http://www.ncbi.nlm.nih.gov/entrez/query.fcgi?cmd=search&db=gene&term=OLFML3) |  |
| 0.0002034 | 0.020451 | 723.3857978 | 229.0780746 | 3.1578133 | 5900575 | [CD276](http://www.ncbi.nlm.nih.gov/entrez/query.fcgi?cmd=search&db=gene&term=CD276) | Cell adhesion molecules (CAMs) |
| 6.52E-05 | 0.016179 | 443.8423709 | 140.6153907 | 3.1564281 | 4810020 | [PLEK](http://www.ncbi.nlm.nih.gov/entrez/query.fcgi?cmd=search&db=gene&term=PLEK) | immunology |
| 0.0015791 | 0.043775 | 35714.10985 | 11318.76549 | 3.1553008 | 7650296 | [DCN](http://www.ncbi.nlm.nih.gov/entrez/query.fcgi?cmd=search&db=gene&term=DCN) | TGF-beta signaling pathway |
| 0.0016739 | 0.045143 | 414.8037163 | 131.7359867 | 3.1487502 | 3870246 | [NTRK2](http://www.ncbi.nlm.nih.gov/entrez/query.fcgi?cmd=search&db=gene&term=NTRK2) | MAPK signaling pathway, signal_transduction |
| 0.003284 | 0.061459 | 526.6029993 | 167.3436219 | 3.1468364 | 10747 | [DOCK2](http://www.ncbi.nlm.nih.gov/entrez/query.fcgi?cmd=search&db=gene&term=DOCK2) |  |
| 0.0006595 | 0.029691 | 9854.905748 | 3138.97361 | 3.1395313 | 1510458 | [MYH9](http://www.ncbi.nlm.nih.gov/entrez/query.fcgi?cmd=search&db=gene&term=MYH9) | Regulation of actin cytoskeleton, Tight junction |
| 0.0004693 | 0.026828 | 4453.576802 | 1418.642101 | 3.1393237 | 5910431 | [COL4A1](http://www.ncbi.nlm.nih.gov/entrez/query.fcgi?cmd=search&db=gene&term=COL4A1) | Cell Communication, ECM-receptor interaction, Focal adhesion, immunology |
| 0.000472 | 0.026828 | 725.9031471 | 231.260904 | 3.1388926 | 7320561 | [OAS2](http://www.ncbi.nlm.nih.gov/entrez/query.fcgi?cmd=search&db=gene&term=OAS2) | immunology, misc |
| 0.0001405 | 0.018893 | 470.0108326 | 149.7678919 | 3.1382617 | 5340162 | [KIAA1598](http://www.ncbi.nlm.nih.gov/entrez/query.fcgi?cmd=search&db=gene&term=KIAA1598) |  |
| 0.00149 | 0.042622 | 2868.304389 | 917.0734696 | 3.1276713 | 4200176 | [FCGRT](http://www.ncbi.nlm.nih.gov/entrez/query.fcgi?cmd=search&db=gene&term=FCGRT) |  |
| 6.74E-05 | 0.016179 | 4346.648973 | 1391.699444 | 3.123267 | 3800139 | [HEXB](http://www.ncbi.nlm.nih.gov/entrez/query.fcgi?cmd=search&db=gene&term=HEXB) | Aminosugars metabolism, Glycan structures - degradation, Glycosaminoglycan degradation, Glycosphingolipid biosynthesis - ganglioseries, Glycosphingolipid biosynthesis - globoseries, N-Glycan degradation, immunology |
| 0.0010393 | 0.036723 | 698.0305728 | 224.4806769 | 3.1095352 | 6330730 | [SNAI2](http://www.ncbi.nlm.nih.gov/entrez/query.fcgi?cmd=search&db=gene&term=SNAI2) | Adherens junction |
| 0.0012678 | 0.039594 | 1012.565122 | 326.2637203 | 3.1035174 | 3400397 | [EML3](http://www.ncbi.nlm.nih.gov/entrez/query.fcgi?cmd=search&db=gene&term=EML3) |  |
| 0.0001021 | 0.017719 | 455.3185244 | 147.4202016 | 3.0885762 | 6020746 | [CMTM3](http://www.ncbi.nlm.nih.gov/entrez/query.fcgi?cmd=search&db=gene&term=CMTM3) |  |
| 0.0006917 | 0.030043 | 893.0856574 | 289.5553123 | 3.0843353 | 270681 | [IL33](http://www.ncbi.nlm.nih.gov/entrez/query.fcgi?cmd=search&db=gene&term=IL33) |  |
| 7.48E-05 | 0.016325 | 866.4627177 | 281.1145226 | 3.082241 | 6380669 | [SPOCK1](http://www.ncbi.nlm.nih.gov/entrez/query.fcgi?cmd=search&db=gene&term=SPOCK1) |  |
| 0.0040938 | 0.068023 | 1201.772394 | 390.057368 | 3.0810145 | 1090307 | [RNASE1](http://www.ncbi.nlm.nih.gov/entrez/query.fcgi?cmd=search&db=gene&term=RNASE1) |  |
| 4.04E-05 | 0.014558 | 10040.44416 | 3260.186848 | 3.0797143 | 6330474 | [CD151](http://www.ncbi.nlm.nih.gov/entrez/query.fcgi?cmd=search&db=gene&term=CD151) | immunology |
| 6.96E-05 | 0.016325 | 817.0811049 | 265.7347592 | 3.0747995 | 3360646 | [MVP](http://www.ncbi.nlm.nih.gov/entrez/query.fcgi?cmd=search&db=gene&term=MVP) |  |
| 7.90E-06 | 0.012163 | 1481.972563 | 483.2332476 | 3.0667852 | 3060292 | [LAMP2](http://www.ncbi.nlm.nih.gov/entrez/query.fcgi?cmd=search&db=gene&term=LAMP2) | immunology |
| 0.0003676 | 0.024601 | 1556.284583 | 508.3926982 | 3.0611859 | 7570324 | [ID3](http://www.ncbi.nlm.nih.gov/entrez/query.fcgi?cmd=search&db=gene&term=ID3) | TGF-beta signaling pathway, gene_regulation, transcription |
| 0.0015208 | 0.042885 | 1503.869955 | 491.5978257 | 3.0591469 | 6980253 | [PTPLB](http://www.ncbi.nlm.nih.gov/entrez/query.fcgi?cmd=search&db=gene&term=PTPLB) |  |
| 0.0003102 | 0.02341 | 633.063002 | 207.4971476 | 3.050948 | 5960747 | [TRIM22](http://www.ncbi.nlm.nih.gov/entrez/query.fcgi?cmd=search&db=gene&term=TRIM22) |  |
| 8.77E-05 | 0.017191 | 462.8627617 | 151.7697955 | 3.0497686 | 6510707 | [FER1L3](http://www.ncbi.nlm.nih.gov/entrez/query.fcgi?cmd=search&db=gene&term=FER1L3) |  |
| 0.000313 | 0.023475 | 668.0733659 | 220.4384594 | 3.030657 | 650689 | [CLIP3](http://www.ncbi.nlm.nih.gov/entrez/query.fcgi?cmd=search&db=gene&term=CLIP3) |  |
| 0.0006201 | 0.029281 | 870.4436644 | 289.0158333 | 3.0117508 | 4120019 | [PLSCR4](http://www.ncbi.nlm.nih.gov/entrez/query.fcgi?cmd=search&db=gene&term=PLSCR4) |  |
| 0.0046839 | 0.072596 | 583.6522857 | 193.9466964 | 3.0093438 | 7610730 | [DOCK11](http://www.ncbi.nlm.nih.gov/entrez/query.fcgi?cmd=search&db=gene&term=DOCK11) |  |
| 0.0017164 | 0.045525 | 1842.893343 | 613.1483055 | 3.0056241 | 1400689 | [JAM3](http://www.ncbi.nlm.nih.gov/entrez/query.fcgi?cmd=search&db=gene&term=JAM3) | Cell adhesion molecules (CAMs), Epithelial cell signaling in Helicobacter pylori infection, Leukocyte transendothelial migration, Tight junction |
| 1.78E-05 | 0.012893 | 1996.458697 | 664.262269 | 3.0055278 | 5960021 |  |  |
| 0.0006515 | 0.029521 | 566.2590019 | 188.4581612 | 3.0046934 | 450491 | [CASP1](http://www.ncbi.nlm.nih.gov/entrez/query.fcgi?cmd=search&db=gene&term=CASP1) | Caspase Cascade in Apoptosis, D4-GDI Signaling Pathway, IL 18 Signaling Pathway, Dentatorubropallidoluysian atrophy (DRPLA), Huntington\'s disease, MAPK signaling pathway, Neurodegenerative Disorders, apoptosis, immunology |
| 7.21E-05 | 0.016325 | 1732.22566 | 576.96392 | 3.0023119 | 6520725 | [TNFRSF14](http://www.ncbi.nlm.nih.gov/entrez/query.fcgi?cmd=search&db=gene&term=TNFRSF14) | Cytokine-cytokine receptor interaction, immunology |
| 0.0002558 | 0.022164 | 1590.328611 | 530.6852908 | 2.9967452 | 6560301 | [SH2B3](http://www.ncbi.nlm.nih.gov/entrez/query.fcgi?cmd=search&db=gene&term=SH2B3) |  |
| 0.0023817 | 0.05282 | 509.7422799 | 170.3603775 | 2.9921411 | 2640768 | [CTSC](http://www.ncbi.nlm.nih.gov/entrez/query.fcgi?cmd=search&db=gene&term=CTSC) | immunology |
| 5.82E-05 | 0.01576 | 348.6529109 | 116.5480183 | 2.9914958 | 7560487 | [CD163](http://www.ncbi.nlm.nih.gov/entrez/query.fcgi?cmd=search&db=gene&term=CD163) |  |
| 0.0049188 | 0.074056 | 10114.58919 | 3384.725211 | 2.9883044 | 1070215 | [CAV1](http://www.ncbi.nlm.nih.gov/entrez/query.fcgi?cmd=search&db=gene&term=CAV1) | Actions of Nitric Oxide in the Heart, Integrin Signaling Pathway, Focal adhesion |
| 0.0004658 | 0.026685 | 1202.348284 | 403.8005526 | 2.9775796 | 2900594 | [PGD](http://www.ncbi.nlm.nih.gov/entrez/query.fcgi?cmd=search&db=gene&term=PGD) | Pentose phosphate pathway, immunology |
| 1.81E-05 | 0.012893 | 1659.899137 | 558.5770531 | 2.9716565 | 6200128 | [ROD1](http://www.ncbi.nlm.nih.gov/entrez/query.fcgi?cmd=search&db=gene&term=ROD1) |  |
| 0.0009554 | 0.034956 | 480.2768543 | 162.0494092 | 2.963768 | 110180 | [ARHGAP9](http://www.ncbi.nlm.nih.gov/entrez/query.fcgi?cmd=search&db=gene&term=ARHGAP9) |  |
| 0.0002838 | 0.022708 | 839.0097268 | 284.0554836 | 2.9536826 | 2850039 | [TBC1D2B](http://www.ncbi.nlm.nih.gov/entrez/query.fcgi?cmd=search&db=gene&term=TBC1D2B) |  |
| 0.0006951 | 0.030043 | 310.0225064 | 105.1176647 | 2.9492903 | 4610341 | [TMEM45A](http://www.ncbi.nlm.nih.gov/entrez/query.fcgi?cmd=search&db=gene&term=TMEM45A) |  |
| 0.0018332 | 0.046912 | 379.8207741 | 129.047623 | 2.9432605 | 1340593 | [GPC3](http://www.ncbi.nlm.nih.gov/entrez/query.fcgi?cmd=search&db=gene&term=GPC3) |  |
| 0.0009044 | 0.034003 | 2298.374296 | 781.5147848 | 2.9409223 | 4210095 | [ATP1A1](http://www.ncbi.nlm.nih.gov/entrez/query.fcgi?cmd=search&db=gene&term=ATP1A1) | immunology |
| 0.0041841 | 0.068783 | 1864.087978 | 633.9293203 | 2.9405297 | 4880626 | [GIMAP4](http://www.ncbi.nlm.nih.gov/entrez/query.fcgi?cmd=search&db=gene&term=GIMAP4) |  |
| 7.17E-05 | 0.016325 | 307.2706126 | 104.6380883 | 2.9365083 | 6420630 | [SCARA3](http://www.ncbi.nlm.nih.gov/entrez/query.fcgi?cmd=search&db=gene&term=SCARA3) |  |
| 5.14E-05 | 0.015414 | 924.9167412 | 315.1236906 | 2.9350911 | 630091 | [NCOA7](http://www.ncbi.nlm.nih.gov/entrez/query.fcgi?cmd=search&db=gene&term=NCOA7) |  |
| 0.0023291 | 0.052324 | 945.9188903 | 322.797779 | 2.9303761 | 7380546 | [FXYD5](http://www.ncbi.nlm.nih.gov/entrez/query.fcgi?cmd=search&db=gene&term=FXYD5) |  |
| 0.0001229 | 0.018034 | 2307.169272 | 787.9645232 | 2.9280116 | 5090215 | [IFI6](http://www.ncbi.nlm.nih.gov/entrez/query.fcgi?cmd=search&db=gene&term=IFI6) |  |
| 2.88E-05 | 0.014422 | 1642.563703 | 561.5172677 | 2.9252238 | 510288 | [MFSD1](http://www.ncbi.nlm.nih.gov/entrez/query.fcgi?cmd=search&db=gene&term=MFSD1) |  |
| 0.0023639 | 0.052605 | 501.4357726 | 171.5121893 | 2.923616 | 6520598 | [SLC2A10](http://www.ncbi.nlm.nih.gov/entrez/query.fcgi?cmd=search&db=gene&term=SLC2A10) |  |
| 0.0001521 | 0.019292 | 610.6256701 | 209.2130861 | 2.9186782 | 6290725 | [FYN](http://www.ncbi.nlm.nih.gov/entrez/query.fcgi?cmd=search&db=gene&term=FYN) | Bioactive Peptide Induced Signaling Pathway, Eph Kinases and ephrins support platelet aggregation, Erk and PI-3 Kinase Are Necessary for Collagen Binding in Corneal Epithelia, IL-7 Signal Transduction, Integrin Signaling Pathway, Lck and Fyn tyrosine kinases in initiation of TCR Activation, Reelin Signaling Pathway, T Cell Receptor Signaling Pathway, TSP-1 Induced Apoptosis in Microvascular Endothelial Cell , Adherens junction, Axon guidance, Fc epsilon RI signaling pathway, Focal adhesion, NA, Natural killer cell mediated cytotoxicity, T cell receptor signaling pathway, signal_transduction, tsonc |
| 0.0001227 | 0.018034 | 794.9825887 | 272.3826968 | 2.9186237 | 2190128 | [RAB32](http://www.ncbi.nlm.nih.gov/entrez/query.fcgi?cmd=search&db=gene&term=RAB32) |  |
| 0.0048497 | 0.073632 | 893.7669001 | 306.2835541 | 2.9181028 | 4560270 | [PTPLB](http://www.ncbi.nlm.nih.gov/entrez/query.fcgi?cmd=search&db=gene&term=PTPLB) |  |
| 4.04E-05 | 0.014558 | 653.495988 | 224.0760302 | 2.9164029 | 1470520 | [CMTM6](http://www.ncbi.nlm.nih.gov/entrez/query.fcgi?cmd=search&db=gene&term=CMTM6) |  |
| 0.000453 | 0.026262 | 1373.743811 | 471.2028449 | 2.915398 | 10685 | [ANTXR2](http://www.ncbi.nlm.nih.gov/entrez/query.fcgi?cmd=search&db=gene&term=ANTXR2) |  |
| 0.0001471 | 0.019074 | 566.2333617 | 194.396836 | 2.9127705 | 5900725 | [PHLDA1](http://www.ncbi.nlm.nih.gov/entrez/query.fcgi?cmd=search&db=gene&term=PHLDA1) |  |
| 0.0001761 | 0.019771 | 6364.146132 | 2197.226086 | 2.8964457 | 3290685 | [DPYSL2](http://www.ncbi.nlm.nih.gov/entrez/query.fcgi?cmd=search&db=gene&term=DPYSL2) | Axon guidance |
| 0.001599 | 0.044064 | 1025.520433 | 354.4327191 | 2.8934136 | 3370164 | [ATP1A1](http://www.ncbi.nlm.nih.gov/entrez/query.fcgi?cmd=search&db=gene&term=ATP1A1) | immunology |
| 0.0014371 | 0.041807 | 726.4254102 | 251.7577162 | 2.8854147 | 1400703 | [CPVL](http://www.ncbi.nlm.nih.gov/entrez/query.fcgi?cmd=search&db=gene&term=CPVL) |  |
| 0.0001385 | 0.018893 | 862.9035918 | 299.1275303 | 2.8847348 | 6580487 | [HSD17B11](http://www.ncbi.nlm.nih.gov/entrez/query.fcgi?cmd=search&db=gene&term=HSD17B11) |  |
| 0.0006068 | 0.028845 | 1118.373448 | 387.894098 | 2.8831927 | 4250398 | [STK4](http://www.ncbi.nlm.nih.gov/entrez/query.fcgi?cmd=search&db=gene&term=STK4) | MAPK signaling pathway, cell_cycle, cell_signaling, signal_transduction |
| 0.0008482 | 0.033518 | 862.4376919 | 299.3260224 | 2.8812653 | 6380463 | [OSTF1](http://www.ncbi.nlm.nih.gov/entrez/query.fcgi?cmd=search&db=gene&term=OSTF1) |  |
| 0.0002542 | 0.022106 | 973.8348959 | 339.1775011 | 2.871166 | 7150678 | [RAB8A](http://www.ncbi.nlm.nih.gov/entrez/query.fcgi?cmd=search&db=gene&term=RAB8A) | Rab GTPases Mark Targets In The Endocytotic Machinery |
| 0.0015138 | 0.042844 | 1258.693351 | 438.5444114 | 2.8701616 | 4290605 | [SLC44A1](http://www.ncbi.nlm.nih.gov/entrez/query.fcgi?cmd=search&db=gene&term=SLC44A1) |  |
| 0.0039252 | 0.066827 | 606.1858402 | 211.2085989 | 2.8700813 | 1260086 | [ID2](http://www.ncbi.nlm.nih.gov/entrez/query.fcgi?cmd=search&db=gene&term=ID2) | TGF-beta signaling pathway, gene_regulation, transcription |
| 0.0001872 | 0.020011 | 636.0328744 | 221.6702904 | 2.8692743 | 4290575 | [C7orf23](http://www.ncbi.nlm.nih.gov/entrez/query.fcgi?cmd=search&db=gene&term=C7orf23) |  |
| 0.0001031 | 0.017719 | 3499.506152 | 1219.712603 | 2.8691235 | 2690047 | [ARL6IP1](http://www.ncbi.nlm.nih.gov/entrez/query.fcgi?cmd=search&db=gene&term=ARL6IP1) |  |
| 0.0037984 | 0.065513 | 274.8749618 | 95.8543986 | 2.8676301 | 730577 | [CPXM1](http://www.ncbi.nlm.nih.gov/entrez/query.fcgi?cmd=search&db=gene&term=CPXM1) |  |
| 0.0018236 | 0.046787 | 14491.92444 | 5063.015291 | 2.862311 | 4730025 | [LOC341457](http://www.ncbi.nlm.nih.gov/entrez/query.fcgi?cmd=search&db=gene&term=LOC341457) |  |
| 0.0041203 | 0.06821 | 1495.329762 | 523.1828937 | 2.8581396 | 5890131 | [PDXK](http://www.ncbi.nlm.nih.gov/entrez/query.fcgi?cmd=search&db=gene&term=PDXK) | Vitamin B6 metabolism |
| 0.0001607 | 0.019731 | 937.5421822 | 328.5737298 | 2.8533693 | 1430647 | [TAX1BP3](http://www.ncbi.nlm.nih.gov/entrez/query.fcgi?cmd=search&db=gene&term=TAX1BP3) | Chaperones modulate interferon Signaling Pathway |
| 0.0024749 | 0.05386 | 427.6360358 | 150.1436174 | 2.8481799 | 5570445 | [PRR5](http://www.ncbi.nlm.nih.gov/entrez/query.fcgi?cmd=search&db=gene&term=PRR5) |  |
| 0.000283 | 0.022708 | 551.0644289 | 193.8422207 | 2.8428504 | 540619 | [TSPAN4](http://www.ncbi.nlm.nih.gov/entrez/query.fcgi?cmd=search&db=gene&term=TSPAN4) |  |
| 0.0001819 | 0.019937 | 452.2515078 | 159.8804025 | 2.8286863 | 5700670 | [WASPIP](http://www.ncbi.nlm.nih.gov/entrez/query.fcgi?cmd=search&db=gene&term=WASPIP) |  |
| 0.0046806 | 0.072588 | 1448.576672 | 513.1138668 | 2.8231096 | 4610615 | [CXCL12](http://www.ncbi.nlm.nih.gov/entrez/query.fcgi?cmd=search&db=gene&term=CXCL12) | CXCR4 Signaling Pathway, Pertussis toxin-insensitive CCR5 Signaling in Macrophage, Axon guidance, Cytokine-cytokine receptor interaction, Leukocyte transendothelial migration |
| 0.0049455 | 0.074081 | 290.776711 | 103.1864215 | 2.8179746 | 2810010 | [CCL3L3](http://www.ncbi.nlm.nih.gov/entrez/query.fcgi?cmd=search&db=gene&term=CCL3L3) |  |
| 0.0023236 | 0.052324 | 527.9681145 | 187.3822451 | 2.8175995 | 3890484 | [ANG](http://www.ncbi.nlm.nih.gov/entrez/query.fcgi?cmd=search&db=gene&term=ANG) | angiogenesis |
| 0.0014124 | 0.041638 | 402.4661921 | 142.94496 | 2.8155326 | 610164 | [FYN](http://www.ncbi.nlm.nih.gov/entrez/query.fcgi?cmd=search&db=gene&term=FYN) | Bioactive Peptide Induced Signaling Pathway, Eph Kinases and ephrins support platelet aggregation, Erk and PI-3 Kinase Are Necessary for Collagen Binding in Corneal Epithelia, IL-7 Signal Transduction, Integrin Signaling Pathway, Lck and Fyn tyrosine kinases in initiation of TCR Activation, Reelin Signaling Pathway, T Cell Receptor Signaling Pathway, TSP-1 Induced Apoptosis in Microvascular Endothelial Cell , Adherens junction, Axon guidance, Fc epsilon RI signaling pathway, Focal adhesion, NA, Natural killer cell mediated cytotoxicity, T cell receptor signaling pathway, signal_transduction, tsonc |
| 0.0002056 | 0.020451 | 1361.125792 | 483.8976493 | 2.8128382 | 7380279 | [GLT25D1](http://www.ncbi.nlm.nih.gov/entrez/query.fcgi?cmd=search&db=gene&term=GLT25D1) |  |
| 0.0010076 | 0.036217 | 3851.087217 | 1369.1563 | 2.8127448 | 3610228 | [PEA15](http://www.ncbi.nlm.nih.gov/entrez/query.fcgi?cmd=search&db=gene&term=PEA15) | Perou's- Intrinsic- Breast-Cancer-Genes |
| 0.0001648 | 0.019753 | 1014.604652 | 361.9483994 | 2.8031749 | 5550136 | [SMAP2](http://www.ncbi.nlm.nih.gov/entrez/query.fcgi?cmd=search&db=gene&term=SMAP2) |  |
| 0.0042765 | 0.069314 | 577.4072704 | 205.9961052 | 2.8030009 | 7320551 | [LYN](http://www.ncbi.nlm.nih.gov/entrez/query.fcgi?cmd=search&db=gene&term=LYN) | BCR Signaling Pathway, Eph Kinases and ephrins support platelet aggregation, Fc Epsilon Receptor I Signaling in Mast Cells, Phosphoinositides and their downstream targets., B cell receptor signaling pathway, Epithelial cell signaling in Helicobacter pylori infection, Fc epsilon RI signaling pathway, Long-term depression, NA, immunology, signal_transduction, tsonc |
| 8.70E-06 | 0.012163 | 1138.623997 | 406.3809374 | 2.8018637 | 6110630 | [HIST1H2BK](http://www.ncbi.nlm.nih.gov/entrez/query.fcgi?cmd=search&db=gene&term=HIST1H2BK) |  |
| 8.79E-05 | 0.017191 | 526.0730169 | 187.7933129 | 2.8013405 | 6560767 | [C17orf62](http://www.ncbi.nlm.nih.gov/entrez/query.fcgi?cmd=search&db=gene&term=C17orf62) |  |
| 0.0044112 | 0.070669 | 1497.990116 | 535.3294208 | 2.7982585 | 5090079 | [C1QTNF1](http://www.ncbi.nlm.nih.gov/entrez/query.fcgi?cmd=search&db=gene&term=C1QTNF1) |  |
| 4.83E-05 | 0.015375 | 490.770676 | 175.4181355 | 2.7977191 | 4260253 | [OBFC2A](http://www.ncbi.nlm.nih.gov/entrez/query.fcgi?cmd=search&db=gene&term=OBFC2A) |  |
| 7.47E-05 | 0.016325 | 1158.567651 | 415.5892208 | 2.7877712 | 1340360 | [RCC2](http://www.ncbi.nlm.nih.gov/entrez/query.fcgi?cmd=search&db=gene&term=RCC2) |  |
| 0.0020414 | 0.049303 | 822.6274383 | 295.330912 | 2.7854431 | 6100050 | [NECAP2](http://www.ncbi.nlm.nih.gov/entrez/query.fcgi?cmd=search&db=gene&term=NECAP2) |  |
| 0.000319 | 0.023516 | 358.5353231 | 129.0121623 | 2.7790816 | 4490520 | [EBI2](http://www.ncbi.nlm.nih.gov/entrez/query.fcgi?cmd=search&db=gene&term=EBI2) |  |
| 0.0027567 | 0.057151 | 647.9051028 | 233.7034788 | 2.7723383 | 4880204 | [SPRYD5](http://www.ncbi.nlm.nih.gov/entrez/query.fcgi?cmd=search&db=gene&term=SPRYD5) |  |
| 1.89E-05 | 0.01307 | 12207.30487 | 4404.730795 | 2.7714077 | 1450670 | [EIF4A1](http://www.ncbi.nlm.nih.gov/entrez/query.fcgi?cmd=search&db=gene&term=EIF4A1) | Internal Ribosome entry pathway, mTOR Signaling Pathway, Regulation of eIF4e and p70 S6 Kinase |
| 0.000282 | 0.022708 | 370.8206682 | 133.9235416 | 2.7688983 | 1780446 | [PCK2](http://www.ncbi.nlm.nih.gov/entrez/query.fcgi?cmd=search&db=gene&term=PCK2) | Adipocytokine signaling pathway, Citrate cycle (TCA cycle), Insulin signaling pathway, PPAR signaling pathway, Pyruvate metabolism |
| 0.000245 | 0.021499 | 15769.56206 | 5697.070723 | 2.7680123 | 6770309 | [MYL6](http://www.ncbi.nlm.nih.gov/entrez/query.fcgi?cmd=search&db=gene&term=MYL6) |  |
| 0.0049701 | 0.074258 | 491.8312866 | 177.752088 | 2.7669508 | 4640630 | [CD302](http://www.ncbi.nlm.nih.gov/entrez/query.fcgi?cmd=search&db=gene&term=CD302) |  |
| 7.19E-05 | 0.016325 | 13146.69577 | 4755.750128 | 2.764379 | 6510176 | [TUBA1B](http://www.ncbi.nlm.nih.gov/entrez/query.fcgi?cmd=search&db=gene&term=TUBA1B) | Gap junction |
| 2.00E-07 | 0.002324 | 384.770437 | 139.351567 | 2.761149 | 4860093 | [LOC653888](http://www.ncbi.nlm.nih.gov/entrez/query.fcgi?cmd=search&db=gene&term=LOC653888) |  |
| 0.0029222 | 0.058389 | 557.792877 | 202.2903661 | 2.7573873 | 5420689 | [ECM2](http://www.ncbi.nlm.nih.gov/entrez/query.fcgi?cmd=search&db=gene&term=ECM2) |  |
| 0.0003028 | 0.023329 | 4946.103133 | 1794.454947 | 2.7563262 | 2970730 | [MYADM](http://www.ncbi.nlm.nih.gov/entrez/query.fcgi?cmd=search&db=gene&term=MYADM) |  |
| 0.0005447 | 0.028008 | 637.410257 | 231.3050883 | 2.7557122 | 5290132 | [COL18A1](http://www.ncbi.nlm.nih.gov/entrez/query.fcgi?cmd=search&db=gene&term=COL18A1) |  |
| 0.0011108 | 0.037691 | 521.2546629 | 189.214382 | 2.7548364 | 1470332 | [FXYD5](http://www.ncbi.nlm.nih.gov/entrez/query.fcgi?cmd=search&db=gene&term=FXYD5) |  |
| 0.0003636 | 0.024601 | 1108.058372 | 402.4105926 | 2.7535517 | 2140671 | [DBNL](http://www.ncbi.nlm.nih.gov/entrez/query.fcgi?cmd=search&db=gene&term=DBNL) |  |
| 0.0012763 | 0.039647 | 736.9602917 | 268.1456342 | 2.7483583 | 3290458 | [FEZ1](http://www.ncbi.nlm.nih.gov/entrez/query.fcgi?cmd=search&db=gene&term=FEZ1) |  |
| 0.0009234 | 0.034341 | 4272.632478 | 1555.482356 | 2.7468216 | 5420136 | [LOC650152](http://www.ncbi.nlm.nih.gov/entrez/query.fcgi?cmd=search&db=gene&term=LOC650152) |  |
| 0.0005694 | 0.028613 | 1060.62013 | 386.7407431 | 2.7424577 | 6900703 | [ARHGEF10](http://www.ncbi.nlm.nih.gov/entrez/query.fcgi?cmd=search&db=gene&term=ARHGEF10) |  |
| 0.0048691 | 0.073632 | 1315.588108 | 481.313673 | 2.7333279 | 3940133 | [FAM46A](http://www.ncbi.nlm.nih.gov/entrez/query.fcgi?cmd=search&db=gene&term=FAM46A) |  |
| 3.71E-05 | 0.014558 | 354.3514874 | 129.7580418 | 2.7308634 | 4780612 | [UNC93B1](http://www.ncbi.nlm.nih.gov/entrez/query.fcgi?cmd=search&db=gene&term=UNC93B1) |  |
| 0.001284 | 0.039772 | 498.2878876 | 183.3681209 | 2.7174183 | 4280482 | [DRAM](http://www.ncbi.nlm.nih.gov/entrez/query.fcgi?cmd=search&db=gene&term=DRAM) |  |
| 5.86E-05 | 0.01576 | 314.3829849 | 115.7873859 | 2.7151747 | 110653 | [NPL](http://www.ncbi.nlm.nih.gov/entrez/query.fcgi?cmd=search&db=gene&term=NPL) |  |
| 0.0003168 | 0.023516 | 2366.862631 | 872.7092053 | 2.7120862 | 240333 | [ETS1](http://www.ncbi.nlm.nih.gov/entrez/query.fcgi?cmd=search&db=gene&term=ETS1) | Keratinocyte Differentiation, METS affect on Macrophage Differentiation, Dorso-ventral axis formation, tsonc |
| 0.0005229 | 0.02772 | 558.20526 | 206.3941596 | 2.7045594 | 1850021 | [SLC25A43](http://www.ncbi.nlm.nih.gov/entrez/query.fcgi?cmd=search&db=gene&term=SLC25A43) |  |
| 5.92E-05 | 0.015775 | 1768.336371 | 654.6649031 | 2.7011321 | 4670398 | [TEAD2](http://www.ncbi.nlm.nih.gov/entrez/query.fcgi?cmd=search&db=gene&term=TEAD2) |  |
| 0.0019782 | 0.048816 | 344.2585637 | 127.4608444 | 2.7008966 | 2760427 | [PYGL](http://www.ncbi.nlm.nih.gov/entrez/query.fcgi?cmd=search&db=gene&term=PYGL) | Insulin signaling pathway, Starch and sucrose metabolism |
| 0.0010338 | 0.036641 | 341.0006888 | 126.282169 | 2.7003075 | 6480692 | [NCF4](http://www.ncbi.nlm.nih.gov/entrez/query.fcgi?cmd=search&db=gene&term=NCF4) | Leukocyte transendothelial migration, immunology |
| 0.0012323 | 0.039289 | 1624.900641 | 602.0679583 | 2.6988658 | 2970521 | [PGRMC1](http://www.ncbi.nlm.nih.gov/entrez/query.fcgi?cmd=search&db=gene&term=PGRMC1) |  |
| 0.0008474 | 0.033518 | 737.3325743 | 273.6694508 | 2.6942451 | 2490240 | [PTPRE](http://www.ncbi.nlm.nih.gov/entrez/query.fcgi?cmd=search&db=gene&term=PTPRE) |  |
| 0.0001898 | 0.020078 | 968.1600238 | 359.6952016 | 2.6916123 | 20142 | [ARPC5](http://www.ncbi.nlm.nih.gov/entrez/query.fcgi?cmd=search&db=gene&term=ARPC5) | Regulation of actin cytoskeleton |
| 0.0034604 | 0.063212 | 420.1041726 | 156.1850412 | 2.6897849 | 6250484 | [CFH](http://www.ncbi.nlm.nih.gov/entrez/query.fcgi?cmd=search&db=gene&term=CFH) | Complement and coagulation cascades |
| 0.0025972 | 0.055224 | 275.141694 | 102.3530304 | 2.6881636 | 1510181 | [PTGES](http://www.ncbi.nlm.nih.gov/entrez/query.fcgi?cmd=search&db=gene&term=PTGES) | Eicosanoid Metabolism, Arachidonic acid metabolism |
| 0.0003765 | 0.024741 | 318.6901445 | 118.6575956 | 2.6857964 | 510128 | [SLC9A9](http://www.ncbi.nlm.nih.gov/entrez/query.fcgi?cmd=search&db=gene&term=SLC9A9) |  |
| 0.004712 | 0.072636 | 554.9314956 | 206.6345259 | 2.6855701 | 2340072 | [PARP12](http://www.ncbi.nlm.nih.gov/entrez/query.fcgi?cmd=search&db=gene&term=PARP12) |  |
| 0.0033003 | 0.061599 | 270.0086356 | 100.8846955 | 2.6764083 | 6840408 | [LY86](http://www.ncbi.nlm.nih.gov/entrez/query.fcgi?cmd=search&db=gene&term=LY86) |  |
| 0.0021648 | 0.050503 | 3222.874126 | 1205.130304 | 2.6742951 | 620255 | [FZD4](http://www.ncbi.nlm.nih.gov/entrez/query.fcgi?cmd=search&db=gene&term=FZD4) | Colorectal cancer, Wnt signaling pathway, development |
| 0.0001427 | 0.019068 | 435.7590071 | 163.0092696 | 2.6732161 | 6060315 | [FERMT3](http://www.ncbi.nlm.nih.gov/entrez/query.fcgi?cmd=search&db=gene&term=FERMT3) |  |
| 0.0037276 | 0.065065 | 255.6323049 | 95.6844271 | 2.6716187 | 3390438 | [PLA2G7](http://www.ncbi.nlm.nih.gov/entrez/query.fcgi?cmd=search&db=gene&term=PLA2G7) | Glycerophospholipid metabolism |
| 1.00E-07 | 0.002324 | 289.0882981 | 108.5314749 | 2.6636356 | 540243 | [NPL](http://www.ncbi.nlm.nih.gov/entrez/query.fcgi?cmd=search&db=gene&term=NPL) |  |
| 0.0006852 | 0.030043 | 611.4421859 | 230.0248291 | 2.6581573 | 7000431 | [MPZL1](http://www.ncbi.nlm.nih.gov/entrez/query.fcgi?cmd=search&db=gene&term=MPZL1) | Cell adhesion molecules (CAMs) |
| 0.0006298 | 0.029291 | 1408.274391 | 531.4683328 | 2.6497804 | 3870253 | [CXCL12](http://www.ncbi.nlm.nih.gov/entrez/query.fcgi?cmd=search&db=gene&term=CXCL12) | CXCR4 Signaling Pathway, Pertussis toxin-insensitive CCR5 Signaling in Macrophage, Axon guidance, Cytokine-cytokine receptor interaction, Leukocyte transendothelial migration |
| 0.0034965 | 0.063552 | 628.2074975 | 237.575529 | 2.6442433 | 6370187 | [RAI14](http://www.ncbi.nlm.nih.gov/entrez/query.fcgi?cmd=search&db=gene&term=RAI14) |  |
| 0.0008472 | 0.033518 | 389.708599 | 147.7121586 | 2.6382974 | 5490470 | [MX2](http://www.ncbi.nlm.nih.gov/entrez/query.fcgi?cmd=search&db=gene&term=MX2) | cell_signaling |
| 0.0002156 | 0.020598 | 32774.00602 | 12438.87909 | 2.6348038 | 3310424 | [ACTG1](http://www.ncbi.nlm.nih.gov/entrez/query.fcgi?cmd=search&db=gene&term=ACTG1) | HIV-I Nef: negative effector of Fas and TNF, Adherens junction, Cell Communication, Focal adhesion, Leukocyte transendothelial migration, Regulation of actin cytoskeleton, Tight junction, cell_signaling, metastasis |
| 0.0006755 | 0.030043 | 426.5995642 | 161.9165805 | 2.6346873 | 610332 | [CHN1](http://www.ncbi.nlm.nih.gov/entrez/query.fcgi?cmd=search&db=gene&term=CHN1) | Rac 1 cell motility signaling pathway, signal_transduction |
| 0.0003616 | 0.024601 | 1263.066031 | 479.5789046 | 2.6336981 | 110451 | [SSR1](http://www.ncbi.nlm.nih.gov/entrez/query.fcgi?cmd=search&db=gene&term=SSR1) |  |
| 0.0035682 | 0.063895 | 445.4597225 | 169.1461105 | 2.6335795 | 360132 | [LHFPL2](http://www.ncbi.nlm.nih.gov/entrez/query.fcgi?cmd=search&db=gene&term=LHFPL2) |  |
| 0.0005211 | 0.02772 | 671.6123623 | 255.0241228 | 2.6335248 | 5360670 | [PLAU](http://www.ncbi.nlm.nih.gov/entrez/query.fcgi?cmd=search&db=gene&term=PLAU) | Fibrinolysis Pathway, Platelet Amyloid Precursor Protein Pathway, Complement and coagulation cascades, angiogenesis |
| 0.0028408 | 0.057943 | 3459.168016 | 1318.678998 | 2.623207 | 3180672 | [LITAF](http://www.ncbi.nlm.nih.gov/entrez/query.fcgi?cmd=search&db=gene&term=LITAF) |  |
| 0.0002817 | 0.022708 | 834.0924074 | 318.2476587 | 2.6208909 | 7550722 | [ZMYM6](http://www.ncbi.nlm.nih.gov/entrez/query.fcgi?cmd=search&db=gene&term=ZMYM6) |  |
| 0.0033961 | 0.062509 | 773.866207 | 295.3258118 | 2.6203812 | 3130612 | [TRIP6](http://www.ncbi.nlm.nih.gov/entrez/query.fcgi?cmd=search&db=gene&term=TRIP6) |  |
| 0.0002142 | 0.020598 | 454.400402 | 173.5623043 | 2.6180823 | 1710736 | [DOCK10](http://www.ncbi.nlm.nih.gov/entrez/query.fcgi?cmd=search&db=gene&term=DOCK10) |  |
| 0.0049369 | 0.074081 | 440.3107315 | 168.9034017 | 2.606879 | 1580465 | [AMICA1](http://www.ncbi.nlm.nih.gov/entrez/query.fcgi?cmd=search&db=gene&term=AMICA1) |  |
| 0.00101 | 0.036217 | 448.2774267 | 172.3299426 | 2.6012742 | 110682 | [S100A11](http://www.ncbi.nlm.nih.gov/entrez/query.fcgi?cmd=search&db=gene&term=S100A11) |  |
| 0.0013201 | 0.040294 | 5089.01564 | 1956.479289 | 2.6011089 | 110167 | [TRAM1](http://www.ncbi.nlm.nih.gov/entrez/query.fcgi?cmd=search&db=gene&term=TRAM1) |  |
| 0.0007144 | 0.03047 | 349.2328867 | 134.3538818 | 2.5993509 | 830735 | [CENTA1](http://www.ncbi.nlm.nih.gov/entrez/query.fcgi?cmd=search&db=gene&term=CENTA1) |  |
| 0.0014659 | 0.042352 | 606.450267 | 233.3831389 | 2.5985179 | 4220259 | [CTSZ](http://www.ncbi.nlm.nih.gov/entrez/query.fcgi?cmd=search&db=gene&term=CTSZ) |  |
| 0.0002009 | 0.020451 | 441.8781159 | 170.1799522 | 2.5965345 | 7050382 | [CASP1](http://www.ncbi.nlm.nih.gov/entrez/query.fcgi?cmd=search&db=gene&term=CASP1) | Caspase Cascade in Apoptosis, D4-GDI Signaling Pathway, IL 18 Signaling Pathway, Dentatorubropallidoluysian atrophy (DRPLA), Huntington\'s disease, MAPK signaling pathway, Neurodegenerative Disorders, apoptosis, immunology |
| 0.0005826 | 0.028704 | 509.9550292 | 196.8676574 | 2.5903444 | 10673 | [PLXNB2](http://www.ncbi.nlm.nih.gov/entrez/query.fcgi?cmd=search&db=gene&term=PLXNB2) | Axon guidance |
| 2.70E-05 | 0.014159 | 4435.667159 | 1712.809085 | 2.5897032 | 6060332 | [PTBP1](http://www.ncbi.nlm.nih.gov/entrez/query.fcgi?cmd=search&db=gene&term=PTBP1) | Internal Ribosome entry pathway |
| 0.002978 | 0.058801 | 489.314099 | 189.0015102 | 2.5889428 | 5720482 | [HERC5](http://www.ncbi.nlm.nih.gov/entrez/query.fcgi?cmd=search&db=gene&term=HERC5) |  |
| 0.0002102 | 0.020598 | 519.8694464 | 201.2800585 | 2.5828165 | 2600747 | [IFIT2](http://www.ncbi.nlm.nih.gov/entrez/query.fcgi?cmd=search&db=gene&term=IFIT2) |  |
| 0.0008576 | 0.033634 | 2047.256897 | 792.8658878 | 2.5820973 | 1170047 | [CRIP1](http://www.ncbi.nlm.nih.gov/entrez/query.fcgi?cmd=search&db=gene&term=CRIP1) |  |
| 0.0003843 | 0.025083 | 386.9043936 | 150.7860714 | 2.565916 | 6580689 | [ARSD](http://www.ncbi.nlm.nih.gov/entrez/query.fcgi?cmd=search&db=gene&term=ARSD) | Androgen and estrogen metabolism, Sphingolipid metabolism |
| 0.0045136 | 0.071365 | 3833.962863 | 1494.725684 | 2.5649943 | 4040706 | [CD34](http://www.ncbi.nlm.nih.gov/entrez/query.fcgi?cmd=search&db=gene&term=CD34) | Adhesion and Diapedesis of Lymphocytes, IL 17 Signaling Pathway, Cell adhesion molecules (CAMs), Hematopoietic cell lineage, angiogenesis, metastasis |
| 0.0045716 | 0.071969 | 312.3883314 | 122.1010245 | 2.5584415 | 6200019 | [KLRB1](http://www.ncbi.nlm.nih.gov/entrez/query.fcgi?cmd=search&db=gene&term=KLRB1) |  |
| 0.0007212 | 0.030535 | 2004.007615 | 783.9268057 | 2.5563708 | 2030577 | [TIMP3](http://www.ncbi.nlm.nih.gov/entrez/query.fcgi?cmd=search&db=gene&term=TIMP3) | Inhibition of Matrix Metalloproteinases, p53 Signaling Pathway, immunology |
| 0.0040394 | 0.067778 | 321.5010859 | 125.8650771 | 2.5543311 | 1260482 | [GZMK](http://www.ncbi.nlm.nih.gov/entrez/query.fcgi?cmd=search&db=gene&term=GZMK) | misc |
| 0.0004466 | 0.026228 | 2317.140281 | 909.7238753 | 2.5470809 | 5560561 | [TNS3](http://www.ncbi.nlm.nih.gov/entrez/query.fcgi?cmd=search&db=gene&term=TNS3) |  |
| 0.0006201 | 0.029281 | 1414.881461 | 556.3584619 | 2.5431112 | 3310288 | [LOC647000](http://www.ncbi.nlm.nih.gov/entrez/query.fcgi?cmd=search&db=gene&term=LOC647000) |  |
| 0.0030908 | 0.059888 | 292.3934808 | 115.048134 | 2.5414883 | 840168 | [CYBB](http://www.ncbi.nlm.nih.gov/entrez/query.fcgi?cmd=search&db=gene&term=CYBB) | Leukocyte transendothelial migration, immunology |
| 0.0021534 | 0.050377 | 1332.075461 | 524.5064638 | 2.5396741 | 6900630 | [ATP2B4](http://www.ncbi.nlm.nih.gov/entrez/query.fcgi?cmd=search&db=gene&term=ATP2B4) | Calcium signaling pathway |
| 0.0005433 | 0.028008 | 387.8817904 | 152.9782856 | 2.535535 | 2640471 | [RNF24](http://www.ncbi.nlm.nih.gov/entrez/query.fcgi?cmd=search&db=gene&term=RNF24) |  |
| 0.0029202 | 0.058389 | 421.4180631 | 166.7468964 | 2.5272918 | 3990553 |  |  |
| 1.10E-05 | 0.012163 | 478.2856279 | 189.7973269 | 2.5199808 | 870056 | [FAM119B](http://www.ncbi.nlm.nih.gov/entrez/query.fcgi?cmd=search&db=gene&term=FAM119B) |  |
| 9.90E-06 | 0.012163 | 765.196341 | 304.1251524 | 2.5160574 | 1340538 | [SGSH](http://www.ncbi.nlm.nih.gov/entrez/query.fcgi?cmd=search&db=gene&term=SGSH) | Glycan structures - degradation, Glycosaminoglycan degradation, immunology, metabolism |
| 0.0011847 | 0.038641 | 487.8198418 | 193.9157051 | 2.5156283 | 1440064 |  |  |
| 0.0012349 | 0.039329 | 1798.617994 | 716.76841 | 2.5093433 | 7560097 | [ATP6AP2](http://www.ncbi.nlm.nih.gov/entrez/query.fcgi?cmd=search&db=gene&term=ATP6AP2) |  |
| 0.0002745 | 0.022619 | 643.6925012 | 257.2964711 | 2.5017541 | 3170332 |  |  |
| 0.0040128 | 0.067591 | 308.448685 | 123.2997927 | 2.5016156 | 2850010 | [AKNA](http://www.ncbi.nlm.nih.gov/entrez/query.fcgi?cmd=search&db=gene&term=AKNA) |  |
| 0.0001388 | 0.018893 | 7401.952424 | 2961.643869 | 2.4992716 | 6940066 | [PFN1](http://www.ncbi.nlm.nih.gov/entrez/query.fcgi?cmd=search&db=gene&term=PFN1) | Erk and PI-3 Kinase Are Necessary for Collagen Binding in Corneal Epithelia, Rho cell motility signaling pathway, Regulation of actin cytoskeleton, immunology |
| 0.0003634 | 0.024601 | 413.9749518 | 165.939113 | 2.4947401 | 2680446 | [ARF3](http://www.ncbi.nlm.nih.gov/entrez/query.fcgi?cmd=search&db=gene&term=ARF3) |  |
| 0.0002043 | 0.020451 | 555.9820813 | 223.3629946 | 2.4891414 | 6960215 | [PITPNM1](http://www.ncbi.nlm.nih.gov/entrez/query.fcgi?cmd=search&db=gene&term=PITPNM1) |  |
| 0.0038033 | 0.065559 | 837.5314533 | 337.4926511 | 2.4816287 | 110433 | [ASS1](http://www.ncbi.nlm.nih.gov/entrez/query.fcgi?cmd=search&db=gene&term=ASS1) | Alanine and aspartate metabolism, Arginine and proline metabolism, Urea cycle and metabolism of amino groups |
| 0.0001633 | 0.019753 | 1352.301838 | 547.5601939 | 2.4696862 | 430747 | [PECAM1](http://www.ncbi.nlm.nih.gov/entrez/query.fcgi?cmd=search&db=gene&term=PECAM1) | Adhesion and Diapedesis of Granulocytes, Adhesion and Diapedesis of Lymphocytes, Adhesion Molecules on Lymphocyte, Cell to Cell Adhesion Signaling, Monocyte and its Surface Molecules, Neutrophil and Its Surface Molecules, Cell adhesion molecules (CAMs), Leukocyte transendothelial migration, cell_signaling, immunology, metastasis |
| 0.0002016 | 0.020451 | 6786.795889 | 2755.088429 | 2.4633677 | 6110392 | [GNS](http://www.ncbi.nlm.nih.gov/entrez/query.fcgi?cmd=search&db=gene&term=GNS) | Glycan structures - degradation, Glycosaminoglycan degradation |
| 0.0001518 | 0.019292 | 540.5616142 | 219.5038264 | 2.4626524 | 130390 | [OLFML1](http://www.ncbi.nlm.nih.gov/entrez/query.fcgi?cmd=search&db=gene&term=OLFML1) |  |
| 0.0006429 | 0.029369 | 512.3413567 | 208.1782324 | 2.4610707 | 2600746 | [VAT1](http://www.ncbi.nlm.nih.gov/entrez/query.fcgi?cmd=search&db=gene&term=VAT1) |  |
| 3.82E-05 | 0.014558 | 1820.459822 | 739.9103184 | 2.460379 | 6100521 | [PHF11](http://www.ncbi.nlm.nih.gov/entrez/query.fcgi?cmd=search&db=gene&term=PHF11) |  |
| 0.0003413 | 0.024356 | 396.5746764 | 161.1910035 | 2.460278 | 6860593 |  |  |
| 0.004638 | 0.072259 | 279.7387386 | 113.7989256 | 2.4581844 | 6200685 | [PARVG](http://www.ncbi.nlm.nih.gov/entrez/query.fcgi?cmd=search&db=gene&term=PARVG) | Focal adhesion |
| 0.0003346 | 0.024236 | 335.559929 | 136.8957268 | 2.4512082 | 1050377 | [PLVAP](http://www.ncbi.nlm.nih.gov/entrez/query.fcgi?cmd=search&db=gene&term=PLVAP) |  |
| 0.0043811 | 0.070373 | 613.6871025 | 250.5017799 | 2.4498313 | 5390730 | [PIK3IP1](http://www.ncbi.nlm.nih.gov/entrez/query.fcgi?cmd=search&db=gene&term=PIK3IP1) |  |
| 5.80E-06 | 0.012163 | 2808.831798 | 1148.350883 | 2.44597 | 4120333 | [ERP29](http://www.ncbi.nlm.nih.gov/entrez/query.fcgi?cmd=search&db=gene&term=ERP29) |  |
| 0.0004505 | 0.026228 | 1165.895267 | 478.2978004 | 2.4375928 | 4390195 | [HMGN1](http://www.ncbi.nlm.nih.gov/entrez/query.fcgi?cmd=search&db=gene&term=HMGN1) | p38 MAPK Signaling Pathway |
| 0.0020121 | 0.049021 | 1504.076247 | 618.7061774 | 2.4310025 | 7550307 | [PRKCDBP](http://www.ncbi.nlm.nih.gov/entrez/query.fcgi?cmd=search&db=gene&term=PRKCDBP) |  |
| 0.0035405 | 0.063738 | 336.6016444 | 138.6774134 | 2.4272276 | 6040037 | [CDC14B](http://www.ncbi.nlm.nih.gov/entrez/query.fcgi?cmd=search&db=gene&term=CDC14B) | Cell cycle |
| 0.0004167 | 0.025615 | 766.2246104 | 315.7928615 | 2.4263519 | 2350091 | [LPXN](http://www.ncbi.nlm.nih.gov/entrez/query.fcgi?cmd=search&db=gene&term=LPXN) |  |
| 0.0004133 | 0.025615 | 320.8771107 | 132.520213 | 2.4213447 | 6650079 | [C3orf14](http://www.ncbi.nlm.nih.gov/entrez/query.fcgi?cmd=search&db=gene&term=C3orf14) |  |
| 0.0023088 | 0.052308 | 813.5789285 | 336.0817161 | 2.4207771 | 6110386 | [PRCP](http://www.ncbi.nlm.nih.gov/entrez/query.fcgi?cmd=search&db=gene&term=PRCP) |  |
| 4.81E-05 | 0.015375 | 350.3379776 | 144.7294058 | 2.4206413 | 1400176 | [LOC642412](http://www.ncbi.nlm.nih.gov/entrez/query.fcgi?cmd=search&db=gene&term=LOC642412) |  |
| 0.0006809 | 0.030043 | 473.2574101 | 195.5148704 | 2.4205699 | 4780133 | [GBA](http://www.ncbi.nlm.nih.gov/entrez/query.fcgi?cmd=search&db=gene&term=GBA) | Cyanoamino acid metabolism, Glycan structures - degradation, Sphingolipid metabolism, Starch and sucrose metabolism, Stilbene, coumarine and lignin biosynthesis, immunology |
| 0.0005933 | 0.028704 | 728.1980441 | 300.8954611 | 2.4201031 | 3800168 | [SLC2A3](http://www.ncbi.nlm.nih.gov/entrez/query.fcgi?cmd=search&db=gene&term=SLC2A3) | Vitamin C in the Brain, cell_signaling, misc, pharmacology |
| 0.0004172 | 0.025615 | 1169.42095 | 484.1378544 | 2.415471 | 5050053 | [TXNDC5](http://www.ncbi.nlm.nih.gov/entrez/query.fcgi?cmd=search&db=gene&term=TXNDC5) |  |
| 0.0013543 | 0.040636 | 1053.592639 | 436.8637188 | 2.4117192 | 1940487 | [STAT5A](http://www.ncbi.nlm.nih.gov/entrez/query.fcgi?cmd=search&db=gene&term=STAT5A) | Bioactive Peptide Induced Signaling Pathway, EGF Signaling Pathway, EPO Signaling Pathway, Growth Hormone Signaling Pathway, IL 2 signaling pathway, IL 3 signaling pathway, IL-10 Anti-inflammatory Signaling Pathway, IL-2 Receptor Beta Chain in T cell Activation, IL22 Soluble Receptor Signaling Pathway , IL-7 Signal Transduction, Inhibition of Cellular Proliferation by Gleevec, Mechanism of Gene Regulation by Peroxisome Proliferators via PPARa(alpha), PDGF Signaling Pathway, TPO Signaling Pathway, Jak-STAT signaling pathway, gene_regulation, immunology, signal_transduction, transcription |
| 0.0007348 | 0.030931 | 774.796643 | 321.8150839 | 2.4075834 | 2570300 | [IFI44](http://www.ncbi.nlm.nih.gov/entrez/query.fcgi?cmd=search&db=gene&term=IFI44) |  |
| 5.35E-05 | 0.015414 | 302.3207584 | 125.8203331 | 2.4027973 | 1240392 | [GPR137B](http://www.ncbi.nlm.nih.gov/entrez/query.fcgi?cmd=search&db=gene&term=GPR137B) |  |
| 0.0003544 | 0.024601 | 692.7960654 | 288.367898 | 2.4024729 | 6620750 | [ZC3HAV1](http://www.ncbi.nlm.nih.gov/entrez/query.fcgi?cmd=search&db=gene&term=ZC3HAV1) |  |
| 0.0001794 | 0.019879 | 387.0961957 | 161.2371477 | 2.4007879 | 5220333 | [C14orf139](http://www.ncbi.nlm.nih.gov/entrez/query.fcgi?cmd=search&db=gene&term=C14orf139) |  |
| 0.0017792 | 0.046294 | 386.6319155 | 161.315639 | 2.3967417 | 4210280 | [PGCP](http://www.ncbi.nlm.nih.gov/entrez/query.fcgi?cmd=search&db=gene&term=PGCP) |  |
| 0.0006946 | 0.030043 | 547.0257534 | 228.2815077 | 2.3962771 | 2360196 | [DOCK1](http://www.ncbi.nlm.nih.gov/entrez/query.fcgi?cmd=search&db=gene&term=DOCK1) | Signaling of Hepatocyte Growth Factor Receptor, Focal adhesion, Regulation of actin cytoskeleton, immunology |
| 0.003771 | 0.065289 | 387.6513516 | 162.099978 | 2.3914337 | 240220 | [M160](http://www.ncbi.nlm.nih.gov/entrez/query.fcgi?cmd=search&db=gene&term=M160) |  |
| 0.0028016 | 0.057448 | 257.8873983 | 107.8568083 | 2.3910164 | 3800373 | [CD37](http://www.ncbi.nlm.nih.gov/entrez/query.fcgi?cmd=search&db=gene&term=CD37) | Hematopoietic cell lineage, angiogenesis, metastasis |
| 0.0044545 | 0.070963 | 795.3342344 | 332.8175276 | 2.3897006 | 4260441 | [CLEC3B](http://www.ncbi.nlm.nih.gov/entrez/query.fcgi?cmd=search&db=gene&term=CLEC3B) |  |
| 0.0006296 | 0.029291 | 15767.85257 | 6608.302225 | 2.3860671 | 1110338 | [MYL6](http://www.ncbi.nlm.nih.gov/entrez/query.fcgi?cmd=search&db=gene&term=MYL6) |  |
| 0.0001247 | 0.018034 | 3182.227674 | 1336.616699 | 2.380808 | 4260754 | [PTBP1](http://www.ncbi.nlm.nih.gov/entrez/query.fcgi?cmd=search&db=gene&term=PTBP1) | Internal Ribosome entry pathway |
| 0.0034862 | 0.06355 | 1066.600383 | 449.537402 | 2.3726622 | 1940504 | [FKBP1A](http://www.ncbi.nlm.nih.gov/entrez/query.fcgi?cmd=search&db=gene&term=FKBP1A) | mTOR Signaling Pathway, NFAT and Hypertrophy of the heart (Transcription in the broken heart) |
| 0.0021588 | 0.050404 | 570.4556078 | 240.506391 | 2.3718938 | 6770242 | [MARCKSL1](http://www.ncbi.nlm.nih.gov/entrez/query.fcgi?cmd=search&db=gene&term=MARCKSL1) |  |
| 0.0001691 | 0.019771 | 478.9235193 | 202.3294165 | 2.3670484 | 1740497 | [RAB8B](http://www.ncbi.nlm.nih.gov/entrez/query.fcgi?cmd=search&db=gene&term=RAB8B) |  |
| 0.0002742 | 0.022619 | 272.9468973 | 115.3183655 | 2.3668988 | 270615 | [ABCC3](http://www.ncbi.nlm.nih.gov/entrez/query.fcgi?cmd=search&db=gene&term=ABCC3) | Multi-Drug Resistance Factors, Nuclear Receptors in Lipid Metabolism and Toxicity |
| 0.0010431 | 0.036768 | 374.5934076 | 158.4113562 | 2.3646878 | 3170491 | [PDIA5](http://www.ncbi.nlm.nih.gov/entrez/query.fcgi?cmd=search&db=gene&term=PDIA5) |  |
| 0.0020585 | 0.049303 | 877.265531 | 371.1624087 | 2.3635624 | 3180053 | [FAM129B](http://www.ncbi.nlm.nih.gov/entrez/query.fcgi?cmd=search&db=gene&term=FAM129B) |  |
| 0.0011494 | 0.038241 | 3251.392979 | 1377.385323 | 2.3605544 | 1770703 | [VKORC1](http://www.ncbi.nlm.nih.gov/entrez/query.fcgi?cmd=search&db=gene&term=VKORC1) | Biosynthesis of steroids |
| 0.0030718 | 0.059639 | 2226.544064 | 945.7808683 | 2.354186 | 6250019 | [SHC1](http://www.ncbi.nlm.nih.gov/entrez/query.fcgi?cmd=search&db=gene&term=SHC1) | Angiotensin II mediated activation of JNK Pathway via Pyk2 dependent signaling, BCR Signaling Pathway, Bioactive Peptide Induced Signaling Pathway, Calcium Signaling by HBx of Hepatitis B virus, EGF Signaling Pathway, EPO Signaling Pathway, Erk and PI-3 Kinase Are Necessary for Collagen Binding in Corneal Epithelia, Erk1/Erk2 Mapk Signaling pathway, Fc Epsilon Receptor I Signaling in Mast Cells, Growth Hormone Signaling Pathway, IGF-1 Signaling Pathway, IL 2 signaling pathway, IL 3 signaling pathway, IL 4 signaling pathway, IL 6 signaling pathway, IL-2 Receptor Beta Chain in T cell Activation, Insulin Signaling Pathway, Integrin Signaling Pathway, Links between Pyk2 and Map Kinases, MAPKinase Signaling Pathway, Multiple antiapoptotic pathways from IGF-1R signaling lead to BAD phosphorylation, Nerve growth factor pathway (NGF), p38 MAPK Signaling Pathway , PDGF Signaling Pathway, PTEN dependent cell cycle arrest and apoptosis, Role of ERBB2 in Signal Transduction and Oncology, Role of E ... |
| 0.0021888 | 0.050818 | 414.9188965 | 176.6612972 | 2.3486689 | 5960343 | [PRIC285](http://www.ncbi.nlm.nih.gov/entrez/query.fcgi?cmd=search&db=gene&term=PRIC285) |  |
| 0.0002038 | 0.020451 | 284.4028142 | 121.3263249 | 2.3441146 | 7000368 | [UBE2L6](http://www.ncbi.nlm.nih.gov/entrez/query.fcgi?cmd=search&db=gene&term=UBE2L6) | Role of Parkin in the Ubiquitin-Proteasomal Pathway, Parkinson\'s disease |
| 0.0008239 | 0.033236 | 2974.946747 | 1269.202199 | 2.3439502 | 130519 | [STAT2](http://www.ncbi.nlm.nih.gov/entrez/query.fcgi?cmd=search&db=gene&term=STAT2) | IFN alpha signaling pathway, Jak-STAT signaling pathway, immunology, signal_transduction |
| 0.0004912 | 0.027279 | 4747.39752 | 2027.602965 | 2.3413842 | 6270100 | [LAMP1](http://www.ncbi.nlm.nih.gov/entrez/query.fcgi?cmd=search&db=gene&term=LAMP1) |  |
| 5.26E-05 | 0.015414 | 41309.78274 | 17650.40916 | 2.3404433 | 7050669 | [TMSB4X](http://www.ncbi.nlm.nih.gov/entrez/query.fcgi?cmd=search&db=gene&term=TMSB4X) | Regulation of actin cytoskeleton |
| 0.0007187 | 0.030519 | 1783.553207 | 762.4753112 | 2.3391619 | 1090167 | [GNAI2](http://www.ncbi.nlm.nih.gov/entrez/query.fcgi?cmd=search&db=gene&term=GNAI2) | Axon guidance, Gap junction, Leukocyte transendothelial migration, Long-term depression, Tight junction |
| 0.0035454 | 0.063738 | 253.0279359 | 108.3565893 | 2.3351412 | 650112 | [LST1](http://www.ncbi.nlm.nih.gov/entrez/query.fcgi?cmd=search&db=gene&term=LST1) |  |
| 0.0041118 | 0.068166 | 239.4526975 | 102.5729639 | 2.3344621 | 4010719 | [CSF2RA](http://www.ncbi.nlm.nih.gov/entrez/query.fcgi?cmd=search&db=gene&term=CSF2RA) | Cytokine-cytokine receptor interaction, Hematopoietic cell lineage, Jak-STAT signaling pathway, immunology |
| 0.000173 | 0.019771 | 6063.274784 | 2601.834622 | 2.3303844 | 6860753 | [TSPO](http://www.ncbi.nlm.nih.gov/entrez/query.fcgi?cmd=search&db=gene&term=TSPO) | Neuroactive ligand-receptor interaction |
| 0.0027725 | 0.057195 | 690.0595417 | 296.2504818 | 2.3293111 | 2760112 | [P2RY5](http://www.ncbi.nlm.nih.gov/entrez/query.fcgi?cmd=search&db=gene&term=P2RY5) | Neuroactive ligand-receptor interaction |
| 0.0029438 | 0.058427 | 1056.497929 | 453.9790958 | 2.3271951 | 7100639 | [ERRFI1](http://www.ncbi.nlm.nih.gov/entrez/query.fcgi?cmd=search&db=gene&term=ERRFI1) |  |
| 0.0009556 | 0.034956 | 323.0345055 | 138.8377696 | 2.3267048 | 7550048 | [CERCAM](http://www.ncbi.nlm.nih.gov/entrez/query.fcgi?cmd=search&db=gene&term=CERCAM) |  |
| 0.0005281 | 0.027838 | 1240.818173 | 533.3332421 | 2.3265345 | 4290279 | [ACLY](http://www.ncbi.nlm.nih.gov/entrez/query.fcgi?cmd=search&db=gene&term=ACLY) | Shuttle for transfer of acetyl groups from mitochondria to the cytosol, Citrate cycle (TCA cycle), Reductive carboxylate cycle (CO2 fixation) |
| 0.0016924 | 0.04531 | 306.7679539 | 132.0356834 | 2.3233716 | 4560576 | [MYD88](http://www.ncbi.nlm.nih.gov/entrez/query.fcgi?cmd=search&db=gene&term=MYD88) | Inactivation of Gsk3 by AKT causes accumulation of b-catenin in Alveolar Macrophages, NFkB activation by Nontypeable Hemophilus influenzae, NF-kB Signaling Pathway, Signal transduction through IL1R, Toll-Like Receptor Pathway, Apoptosis, Toll-like receptor signaling pathway, apoptosis, immunology, misc |
| 0.0035158 | 0.063698 | 5272.759398 | 2274.458419 | 2.3182483 | 1430187 | [CSTB](http://www.ncbi.nlm.nih.gov/entrez/query.fcgi?cmd=search&db=gene&term=CSTB) | immunology |
| 0.0017813 | 0.046294 | 1843.684747 | 796.4893457 | 2.3147639 | 6110253 | [SUMF2](http://www.ncbi.nlm.nih.gov/entrez/query.fcgi?cmd=search&db=gene&term=SUMF2) |  |
| 0.000859 | 0.033634 | 2173.134857 | 939.1085122 | 2.3140402 | 4670750 | [TNFRSF1A](http://www.ncbi.nlm.nih.gov/entrez/query.fcgi?cmd=search&db=gene&term=TNFRSF1A) | Acetylation and Deacetylation of RelA in The Nucleus, Ceramide Signaling Pathway, Chaperones modulate interferon Signaling Pathway, HIV-I Nef: negative effector of Fas and TNF, Keratinocyte Differentiation, NF-kB Signaling Pathway, Regulation of transcriptional activity by PML, SODD/TNFR1 Signaling Pathway, TNF/Stress Related Signaling, TNFR1 Signaling Pathway, Adipocytokine signaling pathway, Apoptosis, Cytokine-cytokine receptor interaction, MAPK signaling pathway, angiogenesis, immunology |
| 0.0001629 | 0.019753 | 1728.273404 | 746.9459932 | 2.3137863 | 4120411 | [ARHGEF2](http://www.ncbi.nlm.nih.gov/entrez/query.fcgi?cmd=search&db=gene&term=ARHGEF2) |  |
| 0.0019575 | 0.048578 | 1646.685658 | 712.0675859 | 2.3125412 | 1410309 | [NBPF11](http://www.ncbi.nlm.nih.gov/entrez/query.fcgi?cmd=search&db=gene&term=NBPF11) |  |
| 0.00114 | 0.038062 | 6905.016633 | 2986.093328 | 2.3123914 | 4590110 | [Sep-09](http://www.ncbi.nlm.nih.gov/entrez/query.fcgi?cmd=search&db=gene&term=Sep-09) |  |
| 0.0016063 | 0.044181 | 806.2237368 | 348.7452273 | 2.3117843 | 1990468 | [ADD3](http://www.ncbi.nlm.nih.gov/entrez/query.fcgi?cmd=search&db=gene&term=ADD3) | cell_signaling, metastasis |
| 0.0013618 | 0.040777 | 461.789673 | 199.8028904 | 2.3112262 | 3370730 | [CDC42EP5](http://www.ncbi.nlm.nih.gov/entrez/query.fcgi?cmd=search&db=gene&term=CDC42EP5) |  |
| 6.27E-05 | 0.016116 | 304.989837 | 132.1627812 | 2.3076833 | 3440386 | [ECM1](http://www.ncbi.nlm.nih.gov/entrez/query.fcgi?cmd=search&db=gene&term=ECM1) |  |
| 0.0012061 | 0.038833 | 659.1912184 | 285.9128825 | 2.3055667 | 2970437 | [PLEKHA2](http://www.ncbi.nlm.nih.gov/entrez/query.fcgi?cmd=search&db=gene&term=PLEKHA2) |  |
| 0.0014746 | 0.042448 | 716.1075128 | 310.6951698 | 2.3048556 | 840278 | [JAM2](http://www.ncbi.nlm.nih.gov/entrez/query.fcgi?cmd=search&db=gene&term=JAM2) | Cell adhesion molecules (CAMs), Epithelial cell signaling in Helicobacter pylori infection, Leukocyte transendothelial migration, Tight junction |
| 0.0019055 | 0.047943 | 267.1700784 | 115.9353264 | 2.3044751 | 7210551 | [BHLHB5](http://www.ncbi.nlm.nih.gov/entrez/query.fcgi?cmd=search&db=gene&term=BHLHB5) |  |
| 0.0023521 | 0.052551 | 816.2307228 | 354.4511702 | 2.3028016 | 4290097 | [CD99](http://www.ncbi.nlm.nih.gov/entrez/query.fcgi?cmd=search&db=gene&term=CD99) | Cell adhesion molecules (CAMs), Leukocyte transendothelial migration |
| 0.0040688 | 0.06788 | 2145.430085 | 931.7338267 | 2.3026212 | 2510392 | [TMEM43](http://www.ncbi.nlm.nih.gov/entrez/query.fcgi?cmd=search&db=gene&term=TMEM43) |  |
| 7.31E-05 | 0.016325 | 584.4543681 | 254.1119055 | 2.2999881 | 1340491 | [SP110](http://www.ncbi.nlm.nih.gov/entrez/query.fcgi?cmd=search&db=gene&term=SP110) |  |
| 0.0008424 | 0.033471 | 1089.463899 | 474.2602646 | 2.2971857 | 4200343 | [CYFIP1](http://www.ncbi.nlm.nih.gov/entrez/query.fcgi?cmd=search&db=gene&term=CYFIP1) | Regulation of actin cytoskeleton |
| 9.32E-05 | 0.017191 | 449.9545118 | 196.0875894 | 2.2946608 | 1660296 | [ID2](http://www.ncbi.nlm.nih.gov/entrez/query.fcgi?cmd=search&db=gene&term=ID2) | TGF-beta signaling pathway, gene_regulation, transcription |
| 0.003035 | 0.059237 | 2795.880808 | 1218.731358 | 2.2940911 | 5050427 | [FKBP1A](http://www.ncbi.nlm.nih.gov/entrez/query.fcgi?cmd=search&db=gene&term=FKBP1A) | mTOR Signaling Pathway, NFAT and Hypertrophy of the heart (Transcription in the broken heart) |
| 0.0040166 | 0.067591 | 274.7626926 | 119.8074885 | 2.2933683 | 6270022 | [HCK](http://www.ncbi.nlm.nih.gov/entrez/query.fcgi?cmd=search&db=gene&term=HCK) | HIV-1 defeats host-mediated resistance by CEM15, Roles of ?-arrestin-dependent Recruitment of Src Kinases in GPCR Signaling, NA, signal_transduction, tsonc |
| 0.0009524 | 0.034956 | 624.5770607 | 273.063975 | 2.2872921 | 6760008 | [DBN1](http://www.ncbi.nlm.nih.gov/entrez/query.fcgi?cmd=search&db=gene&term=DBN1) |  |
| 0.000621 | 0.029281 | 466.9892968 | 204.2085262 | 2.2868257 | 7040286 | [ACSL5](http://www.ncbi.nlm.nih.gov/entrez/query.fcgi?cmd=search&db=gene&term=ACSL5) | Adipocytokine signaling pathway, Fatty acid metabolism, PPAR signaling pathway |
| 0.0014669 | 0.042352 | 392.5326747 | 171.6561094 | 2.2867387 | 5810176 | [RASSF5](http://www.ncbi.nlm.nih.gov/entrez/query.fcgi?cmd=search&db=gene&term=RASSF5) | Leukocyte transendothelial migration |
| 0.0001339 | 0.018672 | 3632.931287 | 1595.3856 | 2.2771494 | 2710114 | [GNB1](http://www.ncbi.nlm.nih.gov/entrez/query.fcgi?cmd=search&db=gene&term=GNB1) | ?-arrestins in GPCR Desensitization, Activation of cAMP-dependent protein kinase, PKA, Activation of Csk by cAMP-dependent Protein Kinase Inhibits Signaling through the T Cell Receptor, Aspirin Blocks Signaling Pathway Involved in Platelet Activation, Attenuation of GPCR Signaling, Bioactive Peptide Induced Signaling Pathway, CCR3 signaling in Eosinophils, ChREBP regulation by carbohydrates and cAMP, Corticosteroids and cardioprotection, CXCR4 Signaling Pathway, Erk1/Erk2 Mapk Signaling pathway, fMLP induced chemokine gene expression in HMC-1 cells, G-Protein Signaling Through Tubby Proteins, How Progesterone Initiates the Oocyte Maturation, Ion Channels and Their Functional Role in Vascular Endothelium, Phospholipids as signalling intermediaries, PKC-catalyzed phosphorylation of inhibitory phosphoprotein of myosin phosphatase, Role of ?-arrestins in the activation and targeting of MAP kinases, Roles of ?-arrestin-dependent Recruitment of Src Kinases in GPCR Signaling, Signaling Pathwa ... |
| 0.0025628 | 0.054816 | 799.2403549 | 351.2306622 | 2.2755427 | 3450577 | [APLP2](http://www.ncbi.nlm.nih.gov/entrez/query.fcgi?cmd=search&db=gene&term=APLP2) |  |
| 0.0015149 | 0.042844 | 582.6145937 | 256.0723541 | 2.2751952 | 3170239 | [CSK](http://www.ncbi.nlm.nih.gov/entrez/query.fcgi?cmd=search&db=gene&term=CSK) | Activation of Csk by cAMP-dependent Protein Kinase Inhibits Signaling through the T Cell Receptor, Activation of Src by Protein-tyrosine phosphatase alpha, Cell to Cell Adhesion Signaling, Integrin Signaling Pathway, Epithelial cell signaling in Helicobacter pylori infection, NA, Regulation of actin cytoskeleton, signal_transduction |
| 0.0019947 | 0.049006 | 336.3885233 | 147.9426052 | 2.2737772 | 5810189 | [ATP2A2](http://www.ncbi.nlm.nih.gov/entrez/query.fcgi?cmd=search&db=gene&term=ATP2A2) | Calcium signaling pathway |
| 0.0010301 | 0.036605 | 313.8361705 | 138.268318 | 2.269762 | 770754 | [TTYH3](http://www.ncbi.nlm.nih.gov/entrez/query.fcgi?cmd=search&db=gene&term=TTYH3) |  |
| 0.0011661 | 0.038487 | 413.2614026 | 182.458167 | 2.2649652 | 70672 | [TCIRG1](http://www.ncbi.nlm.nih.gov/entrez/query.fcgi?cmd=search&db=gene&term=TCIRG1) | ATP synthesis, Epithelial cell signaling in Helicobacter pylori infection, Oxidative phosphorylation |
| 0.0022563 | 0.051765 | 50145.76009 | 22215.31411 | 2.2572609 | 3450719 | [EEF1A1](http://www.ncbi.nlm.nih.gov/entrez/query.fcgi?cmd=search&db=gene&term=EEF1A1) | West Nile Virus, gene_regulation |
| 0.0003316 | 0.024078 | 234.0542138 | 103.8564659 | 2.2536316 | 6020327 | [DENND2D](http://www.ncbi.nlm.nih.gov/entrez/query.fcgi?cmd=search&db=gene&term=DENND2D) |  |
| 2.41E-05 | 0.013372 | 5597.418983 | 2485.049192 | 2.2524379 | 3170162 | [PRNP](http://www.ncbi.nlm.nih.gov/entrez/query.fcgi?cmd=search&db=gene&term=PRNP) | Prion Pathway, Neurodegenerative Disorders, Prion disease, immunology, misc |
| 0.0016403 | 0.044558 | 269.2795264 | 119.7910265 | 2.2479107 | 3290639 | [RAB7B](http://www.ncbi.nlm.nih.gov/entrez/query.fcgi?cmd=search&db=gene&term=RAB7B) |  |
| 0.0035837 | 0.063979 | 363.1568268 | 161.7267755 | 2.245496 | 3850021 | [CASP4](http://www.ncbi.nlm.nih.gov/entrez/query.fcgi?cmd=search&db=gene&term=CASP4) | Caspase Cascade in Apoptosis, MAPK signaling pathway, apoptosis, immunology |
| 0.0007627 | 0.031602 | 836.3498531 | 373.2891784 | 2.2404878 | 2450148 | [PARP4](http://www.ncbi.nlm.nih.gov/entrez/query.fcgi?cmd=search&db=gene&term=PARP4) |  |
| 0.0001348 | 0.018672 | 253.5646168 | 113.6927289 | 2.2302624 | 5570372 | [MXRA8](http://www.ncbi.nlm.nih.gov/entrez/query.fcgi?cmd=search&db=gene&term=MXRA8) |  |
| 0.0016354 | 0.044476 | 304.6247394 | 136.5948178 | 2.2301339 | 110685 | [SYK](http://www.ncbi.nlm.nih.gov/entrez/query.fcgi?cmd=search&db=gene&term=SYK) | Aspirin Blocks Signaling Pathway Involved in Platelet Activation, BCR Signaling Pathway, Fc Epsilon Receptor I Signaling in Mast Cells, IL 2 signaling pathway, IL-2 Receptor Beta Chain in T cell Activation, Ras-Independent pathway in NK cell-mediated cytotoxicity, B cell receptor signaling pathway, Fc epsilon RI signaling pathway, NA, Natural killer cell mediated cytotoxicity, cell_cycle, cell_signaling, signal_transduction |
| 0.0013376 | 0.040469 | 368.6766141 | 165.5296184 | 2.2272547 | 4760747 | [TPST1](http://www.ncbi.nlm.nih.gov/entrez/query.fcgi?cmd=search&db=gene&term=TPST1) |  |
| 0.0010763 | 0.037171 | 3603.806188 | 1620.723401 | 2.2235788 | 2480326 | [HSP90B1](http://www.ncbi.nlm.nih.gov/entrez/query.fcgi?cmd=search&db=gene&term=HSP90B1) |  |
| 0.0045457 | 0.071755 | 312.8434792 | 140.823478 | 2.2215293 | 1400474 | [MYO1D](http://www.ncbi.nlm.nih.gov/entrez/query.fcgi?cmd=search&db=gene&term=MYO1D) |  |
| 0.0010156 | 0.036328 | 2790.278589 | 1259.131081 | 2.216035 | 1260341 | [IL13RA1](http://www.ncbi.nlm.nih.gov/entrez/query.fcgi?cmd=search&db=gene&term=IL13RA1) | Cytokine-cytokine receptor interaction, Jak-STAT signaling pathway, immunology |
| 0.0012691 | 0.039594 | 732.0591693 | 330.3482441 | 2.2160226 | 2810674 | [TRIM4](http://www.ncbi.nlm.nih.gov/entrez/query.fcgi?cmd=search&db=gene&term=TRIM4) |  |
| 0.0027533 | 0.057123 | 4778.61974 | 2156.846791 | 2.2155583 | 3850131 | [YWHAQ](http://www.ncbi.nlm.nih.gov/entrez/query.fcgi?cmd=search&db=gene&term=YWHAQ) | Cell Cycle: G2/M Checkpoint, Cell cycle |
| 0.0005239 | 0.02772 | 1239.797206 | 560.2395326 | 2.212977 | 670113 | [TPP1](http://www.ncbi.nlm.nih.gov/entrez/query.fcgi?cmd=search&db=gene&term=TPP1) |  |
| 0.0046224 | 0.072259 | 620.8465238 | 280.5603064 | 2.2128808 | 3870338 | [IFI44L](http://www.ncbi.nlm.nih.gov/entrez/query.fcgi?cmd=search&db=gene&term=IFI44L) |  |
| 0.0017267 | 0.045557 | 278.6621913 | 126.1393266 | 2.2091619 | 5220678 | [LRRC33](http://www.ncbi.nlm.nih.gov/entrez/query.fcgi?cmd=search&db=gene&term=LRRC33) |  |
| 0.0038946 | 0.0666 | 1919.115942 | 871.1236154 | 2.2030351 | 3450059 | [SH3KBP1](http://www.ncbi.nlm.nih.gov/entrez/query.fcgi?cmd=search&db=gene&term=SH3KBP1) | CBL mediated ligand-induced downregulation of EGF receptors |
| 0.0005306 | 0.027911 | 321.1177884 | 145.8178784 | 2.2021839 | 5720136 | [RAB34](http://www.ncbi.nlm.nih.gov/entrez/query.fcgi?cmd=search&db=gene&term=RAB34) |  |
| 0.0037561 | 0.065289 | 578.50882 | 262.7689613 | 2.2015873 | 6770673 | [SOCS2](http://www.ncbi.nlm.nih.gov/entrez/query.fcgi?cmd=search&db=gene&term=SOCS2) | Insulin signaling pathway, Jak-STAT signaling pathway, Type II diabetes mellitus |
| 0.0042565 | 0.069183 | 624.7806379 | 283.8614416 | 2.2010057 | 1850379 | [LETMD1](http://www.ncbi.nlm.nih.gov/entrez/query.fcgi?cmd=search&db=gene&term=LETMD1) |  |
| 0.002297 | 0.052286 | 476.972664 | 216.924422 | 2.1987965 | 3370092 | [STK39](http://www.ncbi.nlm.nih.gov/entrez/query.fcgi?cmd=search&db=gene&term=STK39) |  |
| 0.0009907 | 0.035745 | 220.1246609 | 100.1931414 | 2.1970033 | 2600424 | [SAA1](http://www.ncbi.nlm.nih.gov/entrez/query.fcgi?cmd=search&db=gene&term=SAA1) | immunology |
| 0.0004712 | 0.026828 | 336.4219076 | 153.1503694 | 2.1966771 | 7100121 | [HNMT](http://www.ncbi.nlm.nih.gov/entrez/query.fcgi?cmd=search&db=gene&term=HNMT) | Histidine metabolism |
| 0.0001116 | 0.018033 | 721.5864791 | 328.9867764 | 2.1933601 | 3870484 | [LUZP1](http://www.ncbi.nlm.nih.gov/entrez/query.fcgi?cmd=search&db=gene&term=LUZP1) |  |
| 0.0001716 | 0.019771 | 1572.705797 | 720.0314834 | 2.1842181 | 1500504 | [AKR1A1](http://www.ncbi.nlm.nih.gov/entrez/query.fcgi?cmd=search&db=gene&term=AKR1A1) | Caprolactam degradation, Glycerolipid metabolism, Glycolysis / Gluconeogenesis |
| 0.0032594 | 0.061248 | 592.4881856 | 271.3058517 | 2.1838386 | 6840246 | [CTSA](http://www.ncbi.nlm.nih.gov/entrez/query.fcgi?cmd=search&db=gene&term=CTSA) |  |
| 0.0002442 | 0.021493 | 562.8351213 | 258.1298823 | 2.1804338 | 7200044 | [SH3BGRL2](http://www.ncbi.nlm.nih.gov/entrez/query.fcgi?cmd=search&db=gene&term=SH3BGRL2) |  |
| 0.0008332 | 0.033334 | 343.8153486 | 157.7353314 | 2.1796978 | 4150520 | [RIPK2](http://www.ncbi.nlm.nih.gov/entrez/query.fcgi?cmd=search&db=gene&term=RIPK2) | FAS signaling pathway ( CD95 ) |
| 0.0016216 | 0.044324 | 719.9514064 | 330.318001 | 2.1795706 | 4640079 | [PGLS](http://www.ncbi.nlm.nih.gov/entrez/query.fcgi?cmd=search&db=gene&term=PGLS) | Pentose phosphate pathway |
| 0.0008601 | 0.033634 | 1814.929033 | 833.2080725 | 2.1782423 | 4250327 | [RHOC](http://www.ncbi.nlm.nih.gov/entrez/query.fcgi?cmd=search&db=gene&term=RHOC) |  |
| 0.0036362 | 0.064556 | 280.6270338 | 128.9530009 | 2.1761962 | 1500301 | [TRO](http://www.ncbi.nlm.nih.gov/entrez/query.fcgi?cmd=search&db=gene&term=TRO) |  |
| 0.000266 | 0.022554 | 16867.28292 | 7767.703251 | 2.1714633 | 1450193 | [LGALS1](http://www.ncbi.nlm.nih.gov/entrez/query.fcgi?cmd=search&db=gene&term=LGALS1) | immunology, misc |
| 0.0003928 | 0.025241 | 1216.186227 | 561.2208632 | 2.1670367 | 4200259 | [ACLY](http://www.ncbi.nlm.nih.gov/entrez/query.fcgi?cmd=search&db=gene&term=ACLY) | Shuttle for transfer of acetyl groups from mitochondria to the cytosol, Citrate cycle (TCA cycle), Reductive carboxylate cycle (CO2 fixation) |
| 0.000884 | 0.03377 | 777.7004352 | 358.9680589 | 2.1664892 | 1940129 | [SIRPA](http://www.ncbi.nlm.nih.gov/entrez/query.fcgi?cmd=search&db=gene&term=SIRPA) |  |
| 0.0028689 | 0.05823 | 373.6741339 | 173.4510222 | 2.1543496 | 1500358 | [ANKRD29](http://www.ncbi.nlm.nih.gov/entrez/query.fcgi?cmd=search&db=gene&term=ANKRD29) |  |
| 0.0006659 | 0.029847 | 956.6846075 | 444.2438449 | 2.1535124 | 160630 | [PLOD3](http://www.ncbi.nlm.nih.gov/entrez/query.fcgi?cmd=search&db=gene&term=PLOD3) | Lysine degradation |
| 8.72E-05 | 0.017191 | 3196.539314 | 1484.5645 | 2.1531832 | 4880168 | [PTTG1IP](http://www.ncbi.nlm.nih.gov/entrez/query.fcgi?cmd=search&db=gene&term=PTTG1IP) |  |
| 0.0039695 | 0.067305 | 238.2039543 | 110.7383846 | 2.1510514 | 1690563 | [SAA1](http://www.ncbi.nlm.nih.gov/entrez/query.fcgi?cmd=search&db=gene&term=SAA1) | immunology |
| 0.0036853 | 0.064645 | 13850.15378 | 6445.321114 | 2.1488695 | 4590441 | [CYB5R3](http://www.ncbi.nlm.nih.gov/entrez/query.fcgi?cmd=search&db=gene&term=CYB5R3) | Aminosugars metabolism |
| 0.0012493 | 0.039505 | 1396.994687 | 650.603789 | 2.147228 | 3370048 | [SURF4](http://www.ncbi.nlm.nih.gov/entrez/query.fcgi?cmd=search&db=gene&term=SURF4) |  |
| 0.0013248 | 0.040307 | 1293.1118 | 602.9120872 | 2.1447767 | 6380370 | [CCND3](http://www.ncbi.nlm.nih.gov/entrez/query.fcgi?cmd=search&db=gene&term=CCND3) | Cyclins and Cell Cycle Regulation, Cell cycle, Focal adhesion, Jak-STAT signaling pathway, Wnt signaling pathway, cell_cycle |
| 0.0013775 | 0.041029 | 528.9777067 | 246.8091235 | 2.1432664 | 2480424 | [PLCG2](http://www.ncbi.nlm.nih.gov/entrez/query.fcgi?cmd=search&db=gene&term=PLCG2) | B cell receptor signaling pathway, Calcium signaling pathway, Epithelial cell signaling in Helicobacter pylori infection, Fc epsilon RI signaling pathway, Inositol phosphate metabolism, Leukocyte transendothelial migration, Natural killer cell mediated cytotoxicity, Phosphatidylinositol signaling system, VEGF signaling pathway, cell_signaling, signal_transduction |
| 0.0040502 | 0.06788 | 4123.255014 | 1927.189795 | 2.1395168 | 7150017 | [C6orf48](http://www.ncbi.nlm.nih.gov/entrez/query.fcgi?cmd=search&db=gene&term=C6orf48) |  |
| 0.0036517 | 0.064645 | 307.4977815 | 143.7571365 | 2.1390088 | 1940048 | [CLEC11A](http://www.ncbi.nlm.nih.gov/entrez/query.fcgi?cmd=search&db=gene&term=CLEC11A) |  |
| 0.0049363 | 0.074081 | 7146.396334 | 3344.42376 | 2.1368095 | 4230520 | [DNCL1](http://www.ncbi.nlm.nih.gov/entrez/query.fcgi?cmd=search&db=gene&term=DNCL1) |  |
| 0.0004382 | 0.026228 | 612.7141659 | 287.1342194 | 2.1338946 | 2810400 | [KLHL3](http://www.ncbi.nlm.nih.gov/entrez/query.fcgi?cmd=search&db=gene&term=KLHL3) |  |
| 0.0033375 | 0.061973 | 457.088949 | 214.2218051 | 2.1337181 | 6660131 | [TSPAN13](http://www.ncbi.nlm.nih.gov/entrez/query.fcgi?cmd=search&db=gene&term=TSPAN13) |  |
| 0.0033724 | 0.062332 | 1476.787612 | 692.8781602 | 2.1313814 | 6100482 | [ATP2B4](http://www.ncbi.nlm.nih.gov/entrez/query.fcgi?cmd=search&db=gene&term=ATP2B4) | Calcium signaling pathway |
| 0.0008792 | 0.033734 | 498.7356427 | 234.0995507 | 2.1304425 | 3850615 | [PRMT2](http://www.ncbi.nlm.nih.gov/entrez/query.fcgi?cmd=search&db=gene&term=PRMT2) | Aminophosphonate metabolism, Androgen and estrogen metabolism, Histidine metabolism, Nitrobenzene degradation, Selenoamino acid metabolism, Tryptophan metabolism, Tyrosine metabolism |
| 0.0015027 | 0.04266 | 501.3148016 | 235.3826882 | 2.1297862 | 6220379 | [PRELID1](http://www.ncbi.nlm.nih.gov/entrez/query.fcgi?cmd=search&db=gene&term=PRELID1) |  |
| 0.0001054 | 0.017903 | 1797.217656 | 844.3074376 | 2.1286294 | 6250053 | [PTTG1IP](http://www.ncbi.nlm.nih.gov/entrez/query.fcgi?cmd=search&db=gene&term=PTTG1IP) |  |
| 0.0012624 | 0.039566 | 250.8899148 | 117.8662868 | 2.1285978 | 4590224 | [EVI2A](http://www.ncbi.nlm.nih.gov/entrez/query.fcgi?cmd=search&db=gene&term=EVI2A) |  |
| 0.0031146 | 0.060071 | 4358.577991 | 2048.650606 | 2.127536 | 6550673 | [SLC25A5](http://www.ncbi.nlm.nih.gov/entrez/query.fcgi?cmd=search&db=gene&term=SLC25A5) | Calcium signaling pathway |
| 0.0002393 | 0.021374 | 374.7497503 | 176.1674564 | 2.127236 | 5560543 | [CBR3](http://www.ncbi.nlm.nih.gov/entrez/query.fcgi?cmd=search&db=gene&term=CBR3) | Arachidonic acid metabolism |
| 0.0002418 | 0.021412 | 517.0769765 | 243.1370262 | 2.1266896 | 4920053 | [BTG3](http://www.ncbi.nlm.nih.gov/entrez/query.fcgi?cmd=search&db=gene&term=BTG3) |  |
| 0.0006029 | 0.028801 | 254.2796441 | 119.6492581 | 2.1252087 | 5670100 | [TCN2](http://www.ncbi.nlm.nih.gov/entrez/query.fcgi?cmd=search&db=gene&term=TCN2) | immunology |
| 0.0017167 | 0.045525 | 637.9114409 | 300.2575852 | 2.1245473 | 4640403 | [AXL](http://www.ncbi.nlm.nih.gov/entrez/query.fcgi?cmd=search&db=gene&term=AXL) | immunology, tsonc |
| 0.0004401 | 0.026228 | 2084.869803 | 984.4316164 | 2.1178412 | 7200041 | [PPT1](http://www.ncbi.nlm.nih.gov/entrez/query.fcgi?cmd=search&db=gene&term=PPT1) | Fatty acid elongation in mitochondria |
| 0.0024806 | 0.05386 | 547.4584028 | 258.8656119 | 2.1148363 | 4230619 | [GCA](http://www.ncbi.nlm.nih.gov/entrez/query.fcgi?cmd=search&db=gene&term=GCA) |  |
| 4.30E-06 | 0.012163 | 263.3976156 | 124.731662 | 2.1117142 | 1240243 | [ATL3](http://www.ncbi.nlm.nih.gov/entrez/query.fcgi?cmd=search&db=gene&term=ATL3) |  |
| 0.0001731 | 0.019771 | 265.8392099 | 125.9814152 | 2.1101462 | 150400 | [LBA1](http://www.ncbi.nlm.nih.gov/entrez/query.fcgi?cmd=search&db=gene&term=LBA1) |  |
| 0.0023425 | 0.052418 | 215.5032496 | 102.3689822 | 2.1051616 | 3460685 | [KYNU](http://www.ncbi.nlm.nih.gov/entrez/query.fcgi?cmd=search&db=gene&term=KYNU) | Tryptophan metabolism |
| 0.0026281 | 0.055555 | 371.6888163 | 176.6633555 | 2.1039384 | 5310170 | [CORO1B](http://www.ncbi.nlm.nih.gov/entrez/query.fcgi?cmd=search&db=gene&term=CORO1B) |  |
| 0.0003661 | 0.024601 | 481.2305593 | 228.815477 | 2.1031382 | 3840593 | [SP110](http://www.ncbi.nlm.nih.gov/entrez/query.fcgi?cmd=search&db=gene&term=SP110) |  |
| 0.0043599 | 0.070235 | 424.895058 | 202.2120038 | 2.1012356 | 1570129 | [TRAFD1](http://www.ncbi.nlm.nih.gov/entrez/query.fcgi?cmd=search&db=gene&term=TRAFD1) |  |
| 0.0007162 | 0.030502 | 1248.153936 | 594.5818526 | 2.099213 | 650168 | [C17orf58](http://www.ncbi.nlm.nih.gov/entrez/query.fcgi?cmd=search&db=gene&term=C17orf58) |  |
| 0.0046982 | 0.072636 | 1385.490467 | 662.095222 | 2.0925849 | 5340246 | [CRIP2](http://www.ncbi.nlm.nih.gov/entrez/query.fcgi?cmd=search&db=gene&term=CRIP2) | misc |
| 0.0041404 | 0.068397 | 840.0808191 | 401.5627216 | 2.0920289 | 3940592 | [PGAM1](http://www.ncbi.nlm.nih.gov/entrez/query.fcgi?cmd=search&db=gene&term=PGAM1) | misc |
| 0.0037322 | 0.065067 | 363.5604117 | 173.8375757 | 2.0913799 | 110450 | [SLC12A9](http://www.ncbi.nlm.nih.gov/entrez/query.fcgi?cmd=search&db=gene&term=SLC12A9) |  |
| 0.0033455 | 0.06205 | 1359.002456 | 650.6944332 | 2.0885417 | 4560110 | [ARMET](http://www.ncbi.nlm.nih.gov/entrez/query.fcgi?cmd=search&db=gene&term=ARMET) |  |
| 0.0002163 | 0.020598 | 279.8845148 | 134.2280597 | 2.0851416 | 160291 | [MVP](http://www.ncbi.nlm.nih.gov/entrez/query.fcgi?cmd=search&db=gene&term=MVP) |  |
| 0.0003928 | 0.025241 | 278.9075003 | 133.9977489 | 2.0814342 | 3520202 | [DOCK8](http://www.ncbi.nlm.nih.gov/entrez/query.fcgi?cmd=search&db=gene&term=DOCK8) |  |
| 0.0005249 | 0.02772 | 2365.005027 | 1140.162933 | 2.0742694 | 2850402 | [PFN1](http://www.ncbi.nlm.nih.gov/entrez/query.fcgi?cmd=search&db=gene&term=PFN1) | Erk and PI-3 Kinase Are Necessary for Collagen Binding in Corneal Epithelia, Rho cell motility signaling pathway, Regulation of actin cytoskeleton, immunology |
| 0.0002997 | 0.023298 | 255.3698834 | 123.1383033 | 2.073846 | 4610246 | [OMD](http://www.ncbi.nlm.nih.gov/entrez/query.fcgi?cmd=search&db=gene&term=OMD) |  |
| 0.0041548 | 0.068477 | 429.9267943 | 207.4546313 | 2.0723895 | 5900152 | [NOX4](http://www.ncbi.nlm.nih.gov/entrez/query.fcgi?cmd=search&db=gene&term=NOX4) |  |
| 0.0041842 | 0.068783 | 459.0596695 | 221.74989 | 2.0701686 | 5050368 | [IL10RB](http://www.ncbi.nlm.nih.gov/entrez/query.fcgi?cmd=search&db=gene&term=IL10RB) | Cytokine-cytokine receptor interaction, Jak-STAT signaling pathway, immunology |
| 0.0019901 | 0.048985 | 935.0332347 | 452.8049874 | 2.06498 | 7380706 | [NINJ1](http://www.ncbi.nlm.nih.gov/entrez/query.fcgi?cmd=search&db=gene&term=NINJ1) |  |
| 0.0021025 | 0.049924 | 1133.838056 | 549.4275417 | 2.0636717 | 620731 | [SPTLC1](http://www.ncbi.nlm.nih.gov/entrez/query.fcgi?cmd=search&db=gene&term=SPTLC1) | Sphingolipid metabolism |
| 0.0001164 | 0.018034 | 397.1029333 | 192.4711435 | 2.0631817 | 2750154 | [ELMO1](http://www.ncbi.nlm.nih.gov/entrez/query.fcgi?cmd=search&db=gene&term=ELMO1) |  |
| 0.0001573 | 0.019693 | 426.472966 | 206.7646546 | 2.0626009 | 5720598 | [MYO1B](http://www.ncbi.nlm.nih.gov/entrez/query.fcgi?cmd=search&db=gene&term=MYO1B) |  |
| 0.0007261 | 0.030654 | 263.0339655 | 127.6066173 | 2.0612878 | 3370037 |  |  |
| 0.0031168 | 0.060071 | 1117.49035 | 542.74986 | 2.0589418 | 1260162 | [DNMT1](http://www.ncbi.nlm.nih.gov/entrez/query.fcgi?cmd=search&db=gene&term=DNMT1) | Methionine metabolism, DNA_adducts, DNA_damage |
| 0.0037719 | 0.065289 | 382.5058058 | 186.0157245 | 2.056309 | 5860519 | [CSPG4](http://www.ncbi.nlm.nih.gov/entrez/query.fcgi?cmd=search&db=gene&term=CSPG4) |  |
| 0.0001154 | 0.018034 | 361.5742091 | 176.0730818 | 2.0535462 | 5550619 | [ADPGK](http://www.ncbi.nlm.nih.gov/entrez/query.fcgi?cmd=search&db=gene&term=ADPGK) |  |
| 0.000597 | 0.028704 | 3587.731004 | 1749.461335 | 2.0507632 | 2570291 | [IFNGR2](http://www.ncbi.nlm.nih.gov/entrez/query.fcgi?cmd=search&db=gene&term=IFNGR2) | Chaperones modulate interferon Signaling Pathway, IFN gamma signaling pathway , Selective expression of chemokine receptors during T-cell polarization, Th1/Th2 Differentiation, Cytokine-cytokine receptor interaction, Jak-STAT signaling pathway, Natural killer cell mediated cytotoxicity, immunology |
| 0.0042736 | 0.069314 | 211.3329816 | 103.1251189 | 2.0492872 | 4260215 | [PLTP](http://www.ncbi.nlm.nih.gov/entrez/query.fcgi?cmd=search&db=gene&term=PLTP) | PPAR signaling pathway |
| 0.0026164 | 0.055429 | 836.8990212 | 408.3942735 | 2.0492428 | 1410161 | [KLHL5](http://www.ncbi.nlm.nih.gov/entrez/query.fcgi?cmd=search&db=gene&term=KLHL5) |  |
| 0.0022767 | 0.051946 | 256.6976498 | 125.4069323 | 2.0469175 | 840025 | [FYN](http://www.ncbi.nlm.nih.gov/entrez/query.fcgi?cmd=search&db=gene&term=FYN) | Bioactive Peptide Induced Signaling Pathway, Eph Kinases and ephrins support platelet aggregation, Erk and PI-3 Kinase Are Necessary for Collagen Binding in Corneal Epithelia, IL-7 Signal Transduction, Integrin Signaling Pathway, Lck and Fyn tyrosine kinases in initiation of TCR Activation, Reelin Signaling Pathway, T Cell Receptor Signaling Pathway, TSP-1 Induced Apoptosis in Microvascular Endothelial Cell , Adherens junction, Axon guidance, Fc epsilon RI signaling pathway, Focal adhesion, NA, Natural killer cell mediated cytotoxicity, T cell receptor signaling pathway, signal_transduction, tsonc |
| 0.0006303 | 0.029291 | 667.4541692 | 326.3633761 | 2.0451258 | 4150066 | [CKB](http://www.ncbi.nlm.nih.gov/entrez/query.fcgi?cmd=search&db=gene&term=CKB) | Arginine and proline metabolism, Urea cycle and metabolism of amino groups, immunology |
| 0.002991 | 0.058919 | 1143.319979 | 559.1519784 | 2.0447392 | 6650037 | [SPTAN1](http://www.ncbi.nlm.nih.gov/entrez/query.fcgi?cmd=search&db=gene&term=SPTAN1) | FAS signaling pathway ( CD95 ), HIV-I Nef: negative effector of Fas and TNF, Induction of apoptosis through DR3 and DR4/5 Death Receptors , Synaptic Proteins at the Synaptic Junction, TNFR1 Signaling Pathway, uCalpain and friends in Cell spread, Tight junction |
| 0.0033267 | 0.061899 | 228.3600848 | 111.7388376 | 2.0436948 | 290292 | [STEAP2](http://www.ncbi.nlm.nih.gov/entrez/query.fcgi?cmd=search&db=gene&term=STEAP2) |  |
| 0.0005122 | 0.02772 | 430.8637468 | 210.9606981 | 2.0423887 | 2510220 | [IFI35](http://www.ncbi.nlm.nih.gov/entrez/query.fcgi?cmd=search&db=gene&term=IFI35) |  |
| 0.0023032 | 0.052304 | 262.4916438 | 128.6176775 | 2.0408675 | 7510377 | [MSC](http://www.ncbi.nlm.nih.gov/entrez/query.fcgi?cmd=search&db=gene&term=MSC) |  |
| 0.0033942 | 0.062509 | 1765.648404 | 865.6944098 | 2.0395747 | 4830148 | [LDB2](http://www.ncbi.nlm.nih.gov/entrez/query.fcgi?cmd=search&db=gene&term=LDB2) |  |
| 0.0003857 | 0.025118 | 542.7452857 | 266.5777925 | 2.0359734 | 1470392 | [PRMT2](http://www.ncbi.nlm.nih.gov/entrez/query.fcgi?cmd=search&db=gene&term=PRMT2) | Aminophosphonate metabolism, Androgen and estrogen metabolism, Histidine metabolism, Nitrobenzene degradation, Selenoamino acid metabolism, Tryptophan metabolism, Tyrosine metabolism |
| 0.0048757 | 0.073681 | 270.1403008 | 132.7130419 | 2.0355219 | 1230286 | [LTC4S](http://www.ncbi.nlm.nih.gov/entrez/query.fcgi?cmd=search&db=gene&term=LTC4S) | Arachidonic acid metabolism, immunology |
| 0.0003626 | 0.024601 | 355.5637537 | 174.7424061 | 2.034788 | 6330725 | [BCL3](http://www.ncbi.nlm.nih.gov/entrez/query.fcgi?cmd=search&db=gene&term=BCL3) | tsonc |
| 0.0012855 | 0.039772 | 727.5517367 | 357.8522744 | 2.0331064 | 4480707 | [LOC653505](http://www.ncbi.nlm.nih.gov/entrez/query.fcgi?cmd=search&db=gene&term=LOC653505) |  |
| 0.0015471 | 0.043319 | 312.5593861 | 154.0558127 | 2.0288711 | 1240142 | [SAMD9](http://www.ncbi.nlm.nih.gov/entrez/query.fcgi?cmd=search&db=gene&term=SAMD9) |  |
| 0.0020644 | 0.049303 | 276.7649571 | 136.4860792 | 2.0277889 | 1230017 | [PHCA](http://www.ncbi.nlm.nih.gov/entrez/query.fcgi?cmd=search&db=gene&term=PHCA) |  |
| 0.0044756 | 0.071133 | 301.3361795 | 148.6560142 | 2.0270702 | 4610338 | [CAMK1](http://www.ncbi.nlm.nih.gov/entrez/query.fcgi?cmd=search&db=gene&term=CAMK1) | Actions of Nitric Oxide in the Heart, Angiotensin II mediated activation of JNK Pathway via Pyk2 dependent signaling, BCR Signaling Pathway, Bioactive Peptide Induced Signaling Pathway, Ca++/ Calmodulin-dependent Protein Kinase Activation, Control of skeletal myogenesis by HDAC & calcium/calmodulin-dependent kinase (CaMK), Corticosteroids and cardioprotection, Effects of calcineurin in Keratinocyte Differentiation, Endocytotic role of NDK, Phosphins and Dynamin, Fc Epsilon Receptor I Signaling in Mast Cells, fMLP induced chemokine gene expression in HMC-1 cells, Links between Pyk2 and Map Kinases, Neuropeptides VIP and PACAP inhibit the apoptosis of activated T cells, NFAT and Hypertrophy of the heart (Transcription in the broken heart), Nitric Oxide Signaling Pathway, Pertussis toxin-insensitive CCR5 Signaling in Macrophage, Regulation of PGC-1a, Role of MEF2D in T-cell Apoptosis, Signal Dependent Regulation of Myogenesis by Corepressor MITR, Signaling Pathway from G-Protein Families, ... |
| 6.21E-05 | 0.016104 | 520.5542816 | 257.2868829 | 2.0232445 | 6370678 | [ROD1](http://www.ncbi.nlm.nih.gov/entrez/query.fcgi?cmd=search&db=gene&term=ROD1) |  |
| 0.0002341 | 0.021257 | 1173.739125 | 580.4429577 | 2.0221438 | 990735 | [RNF149](http://www.ncbi.nlm.nih.gov/entrez/query.fcgi?cmd=search&db=gene&term=RNF149) |  |
| 0.0001736 | 0.019771 | 188.4627395 | 93.244213 | 2.0211736 | 7050082 | [ACP5](http://www.ncbi.nlm.nih.gov/entrez/query.fcgi?cmd=search&db=gene&term=ACP5) | gamma-Hexachlorocyclohexane degradation, Riboflavin metabolism, cell_cycle, cell_signaling, immunology |
| 0.0002962 | 0.023189 | 244.6394016 | 121.2124927 | 2.0182689 | 6130577 | [ELN](http://www.ncbi.nlm.nih.gov/entrez/query.fcgi?cmd=search&db=gene&term=ELN) | immunology |
| 0.0013253 | 0.040307 | 212.9049455 | 105.5286805 | 2.0175079 | 1410181 | [SAMSN1](http://www.ncbi.nlm.nih.gov/entrez/query.fcgi?cmd=search&db=gene&term=SAMSN1) |  |
| 0.0005562 | 0.028193 | 464.2616976 | 230.2899944 | 2.0159873 | 6840184 | [GRN](http://www.ncbi.nlm.nih.gov/entrez/query.fcgi?cmd=search&db=gene&term=GRN) | Proepithelin Conversion to Epithelin and Wound Repair Control |
| 0.0007073 | 0.030345 | 309.7080121 | 153.9517328 | 2.0117215 | 4920474 | [INF2](http://www.ncbi.nlm.nih.gov/entrez/query.fcgi?cmd=search&db=gene&term=INF2) |  |
| 0.0015752 | 0.043775 | 415.5559811 | 207.1232521 | 2.0063222 | 3780619 | [TMEM14A](http://www.ncbi.nlm.nih.gov/entrez/query.fcgi?cmd=search&db=gene&term=TMEM14A) |  |
| 0.0006811 | 0.030043 | 1109.395381 | 553.7972256 | 2.003252 | 2140288 | [FLJ22662](http://www.ncbi.nlm.nih.gov/entrez/query.fcgi?cmd=search&db=gene&term=FLJ22662) |  |
| 0.0002888 | 0.022818 | 1349.665556 | 673.9531064 | 2.0026105 | 1510196 | [PHF11](http://www.ncbi.nlm.nih.gov/entrez/query.fcgi?cmd=search&db=gene&term=PHF11) |  |
| 3.63E-05 | 0.014558 | 226.3200458 | 113.0915522 | 2.0012109 | 6100609 | [UAP1L1](http://www.ncbi.nlm.nih.gov/entrez/query.fcgi?cmd=search&db=gene&term=UAP1L1) |  |
| 0.0037684 | 0.065289 | 817.9287234 | 408.7792204 | 2.0009058 | 870202 | [TNFSF10](http://www.ncbi.nlm.nih.gov/entrez/query.fcgi?cmd=search&db=gene&term=TNFSF10) | Induction of apoptosis through DR3 and DR4/5 Death Receptors , Apoptosis, Cytokine-cytokine receptor interaction, Natural killer cell mediated cytotoxicity |
| 0.0009193 | 0.034232 | 3162.682679 | 1582.697184 | 1.9982867 | 4280632 | [GAS6](http://www.ncbi.nlm.nih.gov/entrez/query.fcgi?cmd=search&db=gene&term=GAS6) | immunology |
| 0.0031869 | 0.060634 | 591.219502 | 296.7576072 | 1.992264 | 1820300 | [CTSO](http://www.ncbi.nlm.nih.gov/entrez/query.fcgi?cmd=search&db=gene&term=CTSO) |  |
| 0.0013407 | 0.040521 | 495.3445702 | 248.7525492 | 1.9913145 | 4230195 | [NAV2](http://www.ncbi.nlm.nih.gov/entrez/query.fcgi?cmd=search&db=gene&term=NAV2) |  |
| 0.0010308 | 0.036605 | 7023.346492 | 3527.943405 | 1.9907764 | 3850053 | [ANP32B](http://www.ncbi.nlm.nih.gov/entrez/query.fcgi?cmd=search&db=gene&term=ANP32B) |  |
| 0.0019132 | 0.048028 | 409.9679458 | 205.9839183 | 1.990291 | 2340180 | [TAGLN2](http://www.ncbi.nlm.nih.gov/entrez/query.fcgi?cmd=search&db=gene&term=TAGLN2) |  |
| 0.0009827 | 0.035634 | 924.419345 | 465.2904703 | 1.9867575 | 1510088 | [ATP1B3](http://www.ncbi.nlm.nih.gov/entrez/query.fcgi?cmd=search&db=gene&term=ATP1B3) |  |
| 0.002256 | 0.051765 | 392.3138007 | 197.5243629 | 1.986154 | 3120379 | [DPY19L1](http://www.ncbi.nlm.nih.gov/entrez/query.fcgi?cmd=search&db=gene&term=DPY19L1) |  |
| 0.0049453 | 0.074081 | 219.4975327 | 110.5587735 | 1.985347 | 2470017 | [LILRB3](http://www.ncbi.nlm.nih.gov/entrez/query.fcgi?cmd=search&db=gene&term=LILRB3) |  |
| 0.0027798 | 0.057262 | 251.1675932 | 126.8165799 | 1.980558 | 2140707 | [SLPI](http://www.ncbi.nlm.nih.gov/entrez/query.fcgi?cmd=search&db=gene&term=SLPI) | Proepithelin Conversion to Epithelin and Wound Repair Control, immunology |
| 0.0015165 | 0.042847 | 1150.762802 | 581.1306567 | 1.9802136 | 7150152 | [PTPLAD1](http://www.ncbi.nlm.nih.gov/entrez/query.fcgi?cmd=search&db=gene&term=PTPLAD1) |  |
| 0.0046598 | 0.072454 | 667.7393632 | 337.7403495 | 1.977079 | 1980424 | [LOC647349](http://www.ncbi.nlm.nih.gov/entrez/query.fcgi?cmd=search&db=gene&term=LOC647349) |  |
| 0.0036582 | 0.064645 | 1317.078013 | 666.7856273 | 1.9752646 | 2320289 | [GLUD1](http://www.ncbi.nlm.nih.gov/entrez/query.fcgi?cmd=search&db=gene&term=GLUD1) | Catabolic Pathways for Arginine , Histidine, Glutamate, Glutamine, and Proline, Arginine and proline metabolism, D-Glutamine and D-glutamate metabolism, Glutamate metabolism, Nitrogen metabolism, Urea cycle and metabolism of amino groups, immunology |
| 0.0035755 | 0.063947 | 205.4268814 | 104.116307 | 1.9730519 | 5670424 | [GNA15](http://www.ncbi.nlm.nih.gov/entrez/query.fcgi?cmd=search&db=gene&term=GNA15) | fMLP induced chemokine gene expression in HMC-1 cells, Calcium signaling pathway |
| 0.0005213 | 0.02772 | 195.7681881 | 99.2979006 | 1.9715239 | 2640025 | [HP](http://www.ncbi.nlm.nih.gov/entrez/query.fcgi?cmd=search&db=gene&term=HP) | immunology, misc |
| 0.0007582 | 0.031505 | 430.6431416 | 218.5904588 | 1.9700912 | 3800273 | [LOC730994](http://www.ncbi.nlm.nih.gov/entrez/query.fcgi?cmd=search&db=gene&term=LOC730994) |  |
| 8.21E-05 | 0.016957 | 273.5843706 | 139.067298 | 1.9672804 | 1410168 | [ELF4](http://www.ncbi.nlm.nih.gov/entrez/query.fcgi?cmd=search&db=gene&term=ELF4) |  |
| 0.0035409 | 0.063738 | 1924.37262 | 978.458399 | 1.9667393 | 6060731 | [MAPRE1](http://www.ncbi.nlm.nih.gov/entrez/query.fcgi?cmd=search&db=gene&term=MAPRE1) |  |
| 0.0045655 | 0.071951 | 349.3125699 | 177.8205893 | 1.9644101 | 3520328 | [ANXA2](http://www.ncbi.nlm.nih.gov/entrez/query.fcgi?cmd=search&db=gene&term=ANXA2) |  |
| 0.004524 | 0.071452 | 807.9322961 | 411.6158357 | 1.9628309 | 1740242 | [FVT1](http://www.ncbi.nlm.nih.gov/entrez/query.fcgi?cmd=search&db=gene&term=FVT1) | misc |
| 0.0008005 | 0.032564 | 416.1582799 | 212.2347353 | 1.9608396 | 6370133 | [SEC23B](http://www.ncbi.nlm.nih.gov/entrez/query.fcgi?cmd=search&db=gene&term=SEC23B) |  |
| 0.000793 | 0.032304 | 256.1204745 | 130.6277085 | 1.9606903 | 4390301 | [TRPV2](http://www.ncbi.nlm.nih.gov/entrez/query.fcgi?cmd=search&db=gene&term=TRPV2) |  |
| 0.0030946 | 0.059922 | 346.1206179 | 176.9703179 | 1.9558117 | 3140369 | [TGIF1](http://www.ncbi.nlm.nih.gov/entrez/query.fcgi?cmd=search&db=gene&term=TGIF1) |  |
| 0.0001447 | 0.019068 | 981.552869 | 503.3543209 | 1.9500237 | 4150193 | [CD47](http://www.ncbi.nlm.nih.gov/entrez/query.fcgi?cmd=search&db=gene&term=CD47) | ECM-receptor interaction, angiogenesis, cell_signaling, immunology, metastasis |
| 0.0028792 | 0.058288 | 1033.944276 | 530.6656462 | 1.9483912 | 5550768 |  |  |
| 0.0018382 | 0.04697 | 252.7942552 | 129.7857071 | 1.9477819 | 1940390 | [PARVA](http://www.ncbi.nlm.nih.gov/entrez/query.fcgi?cmd=search&db=gene&term=PARVA) | Focal adhesion |
| 0.0029297 | 0.058391 | 2132.816893 | 1095.36003 | 1.9471378 | 2470358 | [IFNGR1](http://www.ncbi.nlm.nih.gov/entrez/query.fcgi?cmd=search&db=gene&term=IFNGR1) | Chaperones modulate interferon Signaling Pathway, IFN gamma signaling pathway , Selective expression of chemokine receptors during T-cell polarization, Th1/Th2 Differentiation, Cytokine-cytokine receptor interaction, Jak-STAT signaling pathway, Natural killer cell mediated cytotoxicity, immunology |
| 0.0006436 | 0.029369 | 820.4692651 | 421.8676407 | 1.94485 | 5670672 | [GUSBL1](http://www.ncbi.nlm.nih.gov/entrez/query.fcgi?cmd=search&db=gene&term=GUSBL1) |  |
| 0.0017369 | 0.045557 | 962.8233561 | 495.289645 | 1.9439602 | 4830433 | [LARP6](http://www.ncbi.nlm.nih.gov/entrez/query.fcgi?cmd=search&db=gene&term=LARP6) |  |
| 0.000464 | 0.026685 | 6810.728769 | 3504.851644 | 1.9432288 | 630356 | [DAZAP2](http://www.ncbi.nlm.nih.gov/entrez/query.fcgi?cmd=search&db=gene&term=DAZAP2) |  |
| 0.0012985 | 0.039913 | 451.9406269 | 232.8503621 | 1.9409058 | 2600646 | [WDR45L](http://www.ncbi.nlm.nih.gov/entrez/query.fcgi?cmd=search&db=gene&term=WDR45L) |  |
| 0.0006718 | 0.029973 | 1109.250187 | 573.0285414 | 1.9357678 | 5860477 | [CANX](http://www.ncbi.nlm.nih.gov/entrez/query.fcgi?cmd=search&db=gene&term=CANX) | ER?associated degradation (ERAD) Pathway, Antigen processing and presentation |
| 0.0010552 | 0.036985 | 11423.17637 | 5902.774742 | 1.9352215 | 6520026 | [NUCB1](http://www.ncbi.nlm.nih.gov/entrez/query.fcgi?cmd=search&db=gene&term=NUCB1) | immunology |
| 0.0014193 | 0.041638 | 710.0992435 | 367.2544903 | 1.9335345 | 3060128 | [CHSY1](http://www.ncbi.nlm.nih.gov/entrez/query.fcgi?cmd=search&db=gene&term=CHSY1) | Chondroitin sulfate biosynthesis, Glycan structures - biosynthesis 1 |
| 0.0042297 | 0.069095 | 342.5869702 | 177.2618915 | 1.93266 | 3170593 | [PTPRA](http://www.ncbi.nlm.nih.gov/entrez/query.fcgi?cmd=search&db=gene&term=PTPRA) | Activation of Src by Protein-tyrosine phosphatase alpha, cell_cycle, cell_signaling |
| 0.0043649 | 0.070276 | 350.0972685 | 181.1690631 | 1.9324341 | 840528 | [TRIM47](http://www.ncbi.nlm.nih.gov/entrez/query.fcgi?cmd=search&db=gene&term=TRIM47) |  |
| 0.0046136 | 0.072259 | 518.9299765 | 268.6201418 | 1.9318357 | 2510224 | [ANKRD57](http://www.ncbi.nlm.nih.gov/entrez/query.fcgi?cmd=search&db=gene&term=ANKRD57) |  |
| 0.0016094 | 0.044182 | 241.4250787 | 125.3552453 | 1.9259272 | 6220288 | [PRDM1](http://www.ncbi.nlm.nih.gov/entrez/query.fcgi?cmd=search&db=gene&term=PRDM1) |  |
| 0.0011563 | 0.038295 | 429.0922073 | 223.0666526 | 1.9236054 | 4260189 | [TGIF1](http://www.ncbi.nlm.nih.gov/entrez/query.fcgi?cmd=search&db=gene&term=TGIF1) |  |
| 0.0008757 | 0.033692 | 202.9226339 | 105.5137184 | 1.9231872 | 2710020 |  |  |
| 0.0011674 | 0.038487 | 284.6117596 | 148.0243481 | 1.9227361 | 7210717 | [FGD2](http://www.ncbi.nlm.nih.gov/entrez/query.fcgi?cmd=search&db=gene&term=FGD2) |  |
| 0.0019899 | 0.048985 | 348.5125527 | 181.3459742 | 1.9218103 | 60670 | [LXN](http://www.ncbi.nlm.nih.gov/entrez/query.fcgi?cmd=search&db=gene&term=LXN) |  |
| 0.0012705 | 0.039594 | 497.7680576 | 259.0545113 | 1.92148 | 6280184 | [MAN1C1](http://www.ncbi.nlm.nih.gov/entrez/query.fcgi?cmd=search&db=gene&term=MAN1C1) | Glycan structures - biosynthesis 1, N-Glycan biosynthesis |
| 0.0002954 | 0.023189 | 243.689856 | 127.0105965 | 1.9186577 | 6840075 | [NP](http://www.ncbi.nlm.nih.gov/entrez/query.fcgi?cmd=search&db=gene&term=NP) | Nicotinate and nicotinamide metabolism, Purine metabolism, Pyrimidine metabolism, gene_regulation, immunology, transcription |
| 0.0013217 | 0.040294 | 190.0343332 | 99.0509686 | 1.918551 | 4830575 | [CCDC80](http://www.ncbi.nlm.nih.gov/entrez/query.fcgi?cmd=search&db=gene&term=CCDC80) |  |
| 0.0031407 | 0.060341 | 666.5540431 | 347.5647663 | 1.9177837 | 3140543 | [ZMAT3](http://www.ncbi.nlm.nih.gov/entrez/query.fcgi?cmd=search&db=gene&term=ZMAT3) |  |
| 0.0003092 | 0.02341 | 850.2688553 | 444.2566928 | 1.9139134 | 1710630 | [ST6GAL1](http://www.ncbi.nlm.nih.gov/entrez/query.fcgi?cmd=search&db=gene&term=ST6GAL1) | Glycan structures - biosynthesis 1, N-Glycan biosynthesis |
| 0.0017073 | 0.045474 | 257.2539753 | 134.4257728 | 1.9137251 | 5860204 | [FKBP11](http://www.ncbi.nlm.nih.gov/entrez/query.fcgi?cmd=search&db=gene&term=FKBP11) |  |
| 0.0005674 | 0.028562 | 211.3541994 | 110.4998931 | 1.9127095 | 2760433 | [DCLK1](http://www.ncbi.nlm.nih.gov/entrez/query.fcgi?cmd=search&db=gene&term=DCLK1) |  |
| 0.0041869 | 0.068783 | 237.844387 | 124.4552591 | 1.9110835 | 830440 | [TLR5](http://www.ncbi.nlm.nih.gov/entrez/query.fcgi?cmd=search&db=gene&term=TLR5) | Toll-like receptor signaling pathway, immunology |
| 0.0003562 | 0.024601 | 535.3810886 | 280.4486581 | 1.9090164 | 3190112 | [SERPINB1](http://www.ncbi.nlm.nih.gov/entrez/query.fcgi?cmd=search&db=gene&term=SERPINB1) |  |
| 0.004251 | 0.069183 | 1356.138169 | 711.1587686 | 1.9069415 | 5720364 | [TMEM183B](http://www.ncbi.nlm.nih.gov/entrez/query.fcgi?cmd=search&db=gene&term=TMEM183B) |  |
| 0.0035746 | 0.063947 | 1541.558029 | 809.9704814 | 1.9032274 | 2470619 | [AKT1](http://www.ncbi.nlm.nih.gov/entrez/query.fcgi?cmd=search&db=gene&term=AKT1) | Actions of Nitric Oxide in the Heart, AKT Signaling Pathway, Apoptotic Signaling in Response to DNA Damage, B Cell Survival Pathway, Control of skeletal myogenesis by HDAC & calcium/calmodulin-dependent kinase (CaMK), Corticosteroids and cardioprotection, Human Cytomegalovirus and Map Kinase Pathways, Hypoxia and p53 in the Cardiovascular system, IL 4 signaling pathway, IL-2 Receptor Beta Chain in T cell Activation, Inactivation of Gsk3 by AKT causes accumulation of b-catenin in Alveolar Macrophages, Influence of Ras and Rho proteins on G1 to S Transition, Inhibition of Cellular Proliferation by Gleevec, mTOR Signaling Pathway, Multiple antiapoptotic pathways from IGF-1R signaling lead to BAD phosphorylation, NFAT and Hypertrophy of the heart (Transcription in the broken heart), Phosphoinositides and their downstream targets., Phospholipase C Signaling Pathway, Phospholipids as signalling intermediaries, PTEN dependent cell cycle arrest and apoptosis, Ras Signaling Pathway, Regulation ... |
| 0.0033566 | 0.062181 | 230.6509101 | 121.4891823 | 1.8985304 | 1740673 | [LOC399942](http://www.ncbi.nlm.nih.gov/entrez/query.fcgi?cmd=search&db=gene&term=LOC399942) |  |
| 0.0048573 | 0.073632 | 205.267232 | 108.2281743 | 1.8966155 | 3140110 | [C18orf10](http://www.ncbi.nlm.nih.gov/entrez/query.fcgi?cmd=search&db=gene&term=C18orf10) |  |
| 0.0035492 | 0.063738 | 6982.330177 | 3684.063917 | 1.8952793 | 450553 | [AHNAK](http://www.ncbi.nlm.nih.gov/entrez/query.fcgi?cmd=search&db=gene&term=AHNAK) |  |
| 0.0049514 | 0.074092 | 198.9600241 | 105.3216058 | 1.8890713 | 4780196 | [CD300A](http://www.ncbi.nlm.nih.gov/entrez/query.fcgi?cmd=search&db=gene&term=CD300A) |  |
| 0.0017884 | 0.046294 | 226.6604492 | 120.2507114 | 1.884899 | 5700220 | [DAPK1](http://www.ncbi.nlm.nih.gov/entrez/query.fcgi?cmd=search&db=gene&term=DAPK1) | cell_signaling, immunology |
| 0.0002207 | 0.020839 | 262.6076653 | 139.8330955 | 1.878008 | 7400259 | [IDUA](http://www.ncbi.nlm.nih.gov/entrez/query.fcgi?cmd=search&db=gene&term=IDUA) | Glycan structures - degradation, Glycosaminoglycan degradation, immunology, misc |
| 0.0001123 | 0.018033 | 376.0682183 | 200.3821599 | 1.876755 | 7550181 | [IFNAR1](http://www.ncbi.nlm.nih.gov/entrez/query.fcgi?cmd=search&db=gene&term=IFNAR1) | Bone Remodelling, IFN alpha signaling pathway, Cytokine-cytokine receptor interaction, Jak-STAT signaling pathway, Natural killer cell mediated cytotoxicity, Toll-like receptor signaling pathway, immunology |
| 0.003731 | 0.065067 | 699.5439507 | 373.156323 | 1.8746673 | 830424 | [STK38](http://www.ncbi.nlm.nih.gov/entrez/query.fcgi?cmd=search&db=gene&term=STK38) |  |
| 0.0024017 | 0.053047 | 227.5403676 | 121.4203972 | 1.873988 | 2710491 | [DAP](http://www.ncbi.nlm.nih.gov/entrez/query.fcgi?cmd=search&db=gene&term=DAP) | apoptosis, immunology |
| 0.0001694 | 0.019771 | 292.1975818 | 156.2788666 | 1.8697191 | 580048 | [GBA](http://www.ncbi.nlm.nih.gov/entrez/query.fcgi?cmd=search&db=gene&term=GBA) | Cyanoamino acid metabolism, Glycan structures - degradation, Sphingolipid metabolism, Starch and sucrose metabolism, Stilbene, coumarine and lignin biosynthesis, immunology |
| 0.0029378 | 0.058404 | 732.9434582 | 392.0359582 | 1.8695822 | 7160435 | [EBF3](http://www.ncbi.nlm.nih.gov/entrez/query.fcgi?cmd=search&db=gene&term=EBF3) |  |
| 0.0040117 | 0.067591 | 302.4152999 | 161.7627539 | 1.869499 | 7200240 | [ARHGAP4](http://www.ncbi.nlm.nih.gov/entrez/query.fcgi?cmd=search&db=gene&term=ARHGAP4) | Rho cell motility signaling pathway |
| 0.0014139 | 0.041638 | 869.4689157 | 465.6083798 | 1.8673824 | 2900450 | [ADAR](http://www.ncbi.nlm.nih.gov/entrez/query.fcgi?cmd=search&db=gene&term=ADAR) | RNA polymerase III transcription, Atrazine degradation, DNA_replication |
| 0.002715 | 0.05665 | 3397.840625 | 1821.754228 | 1.8651477 | 3890017 | [CTNNA1](http://www.ncbi.nlm.nih.gov/entrez/query.fcgi?cmd=search&db=gene&term=CTNNA1) | Cell to Cell Adhesion Signaling, Adherens junction, Leukocyte transendothelial migration, Tight junction, cell_signaling, signal_transduction |
| 0.0017458 | 0.045682 | 864.850601 | 463.8197506 | 1.8646265 | 4150544 | [ALDH7A1](http://www.ncbi.nlm.nih.gov/entrez/query.fcgi?cmd=search&db=gene&term=ALDH7A1) | Arginine and proline metabolism, Ascorbate and aldarate metabolism, beta-Alanine metabolism, Bile acid biosynthesis, Butanoate metabolism, Fatty acid metabolism, Glycerolipid metabolism, Glycolysis / Gluconeogenesis, Histidine metabolism, Limonene and pinene degradation, Lysine degradation, Propanoate metabolism, Pyruvate metabolism, Tryptophan metabolism, Valine, leucine and isoleucine degradation |
| 0.004185 | 0.068783 | 237.5606886 | 127.6819665 | 1.8605657 | 5960735 | [LOC199800](http://www.ncbi.nlm.nih.gov/entrez/query.fcgi?cmd=search&db=gene&term=LOC199800) |  |
| 0.0005859 | 0.028704 | 190.0497694 | 102.3704625 | 1.8564903 | 4390441 | [ARHGAP30](http://www.ncbi.nlm.nih.gov/entrez/query.fcgi?cmd=search&db=gene&term=ARHGAP30) |  |
| 0.0022659 | 0.051865 | 465.5972228 | 250.9234804 | 1.8555347 | 6590132 | [IGFBP3](http://www.ncbi.nlm.nih.gov/entrez/query.fcgi?cmd=search&db=gene&term=IGFBP3) | Ghrelin: Regulation of Food Intake and Energy Homeostasis, Hypoxia and p53 in the Cardiovascular system, immunology |
| 0.0038292 | 0.065927 | 300.9490454 | 162.4135859 | 1.8529795 | 1030239 | [SCRN1](http://www.ncbi.nlm.nih.gov/entrez/query.fcgi?cmd=search&db=gene&term=SCRN1) |  |
| 0.0024757 | 0.05386 | 688.1034771 | 371.5055079 | 1.8522026 | 7200167 | [EPB41L2](http://www.ncbi.nlm.nih.gov/entrez/query.fcgi?cmd=search&db=gene&term=EPB41L2) | Tight junction |
| 0.0010704 | 0.037056 | 204.319011 | 110.4717928 | 1.8495129 | 5720682 | [TMEM176A](http://www.ncbi.nlm.nih.gov/entrez/query.fcgi?cmd=search&db=gene&term=TMEM176A) |  |
| 0.0013995 | 0.041478 | 789.2234372 | 427.2203405 | 1.8473452 | 7330379 | [PTMS](http://www.ncbi.nlm.nih.gov/entrez/query.fcgi?cmd=search&db=gene&term=PTMS) |  |
| 0.0004078 | 0.025582 | 341.6273794 | 185.5734146 | 1.8409285 | 6330338 | [ITGA5](http://www.ncbi.nlm.nih.gov/entrez/query.fcgi?cmd=search&db=gene&term=ITGA5) | ECM-receptor interaction, Focal adhesion, Hematopoietic cell lineage, Regulation of actin cytoskeleton, cell_signaling, immunology, metastasis |
| 0.0039199 | 0.066776 | 437.8209263 | 238.1768331 | 1.8382179 | 7150433 | [TCTEX1D2](http://www.ncbi.nlm.nih.gov/entrez/query.fcgi?cmd=search&db=gene&term=TCTEX1D2) |  |
| 0.0011723 | 0.038561 | 1324.032455 | 720.7928382 | 1.8369112 | 940754 | [TMEM183A](http://www.ncbi.nlm.nih.gov/entrez/query.fcgi?cmd=search&db=gene&term=TMEM183A) |  |
| 0.002152 | 0.050377 | 239.3491607 | 130.452202 | 1.8347652 | 5720537 | [ACPL2](http://www.ncbi.nlm.nih.gov/entrez/query.fcgi?cmd=search&db=gene&term=ACPL2) |  |
| 0.0008911 | 0.033921 | 655.7445487 | 357.928571 | 1.8320542 | 3710554 | [FGD5](http://www.ncbi.nlm.nih.gov/entrez/query.fcgi?cmd=search&db=gene&term=FGD5) |  |
| 0.0010657 | 0.037046 | 222.3912998 | 121.3898408 | 1.8320421 | 1500608 | [HSD3B7](http://www.ncbi.nlm.nih.gov/entrez/query.fcgi?cmd=search&db=gene&term=HSD3B7) | Ascorbate and aldarate metabolism, Bile acid biosynthesis, Bisphenol A degradation, Butanoate metabolism, Fructose and mannose metabolism, Galactose metabolism, Glycerophospholipid metabolism, Glycine, serine and threonine metabolism, Linoleic acid metabolism, Lysine degradation, Nucleotide sugars metabolism, Tetrachloroethene degradation |
| 0.0025184 | 0.054402 | 233.2535262 | 127.3986363 | 1.830895 | 4050202 | [CLEC4A](http://www.ncbi.nlm.nih.gov/entrez/query.fcgi?cmd=search&db=gene&term=CLEC4A) |  |
| 0.0031966 | 0.060644 | 251.2115123 | 137.2078761 | 1.8308826 | 990768 | [OAS3](http://www.ncbi.nlm.nih.gov/entrez/query.fcgi?cmd=search&db=gene&term=OAS3) |  |
| 0.0003491 | 0.024601 | 280.0908119 | 153.124733 | 1.8291677 | 6660601 | [HMOX1](http://www.ncbi.nlm.nih.gov/entrez/query.fcgi?cmd=search&db=gene&term=HMOX1) | IL-10 Anti-inflammatory Signaling Pathway, Oxidative Stress Induced Gene Expression Via Nrf2, Porphyrin and chlorophyll metabolism |
| 0.0009542 | 0.034956 | 290.6880658 | 158.9344089 | 1.8289813 | 2470458 | [MICAL1](http://www.ncbi.nlm.nih.gov/entrez/query.fcgi?cmd=search&db=gene&term=MICAL1) |  |
| 0.000558 | 0.028235 | 411.0057113 | 224.927701 | 1.8272792 | 5560086 | [RIPK1](http://www.ncbi.nlm.nih.gov/entrez/query.fcgi?cmd=search&db=gene&term=RIPK1) | Acetylation and Deacetylation of RelA in The Nucleus, Ceramide Signaling Pathway, HIV-I Nef: negative effector of Fas and TNF, Induction of apoptosis through DR3 and DR4/5 Death Receptors , Keratinocyte Differentiation, MAPKinase Signaling Pathway, NF-kB Signaling Pathway, p38 MAPK Signaling Pathway , SODD/TNFR1 Signaling Pathway, TNF/Stress Related Signaling, TNFR1 Signaling Pathway, TNFR2 Signaling Pathway, Apoptosis |
| 0.001937 | 0.048328 | 1693.02949 | 927.124553 | 1.8261079 | 4250458 | [CFL1](http://www.ncbi.nlm.nih.gov/entrez/query.fcgi?cmd=search&db=gene&term=CFL1) |  |
| 0.0001763 | 0.019771 | 1075.665064 | 589.4401536 | 1.8248928 | 5390202 | [TMED9](http://www.ncbi.nlm.nih.gov/entrez/query.fcgi?cmd=search&db=gene&term=TMED9) |  |
| 0.0032967 | 0.061599 | 232.2452909 | 127.3429717 | 1.8237778 | 3990546 | [PLEKHO2](http://www.ncbi.nlm.nih.gov/entrez/query.fcgi?cmd=search&db=gene&term=PLEKHO2) | Cytokine-cytokine receptor interaction |
| 0.0045183 | 0.0714 | 899.8429952 | 493.7417262 | 1.8224974 | 5550736 | [SNX2](http://www.ncbi.nlm.nih.gov/entrez/query.fcgi?cmd=search&db=gene&term=SNX2) |  |
| 0.0030566 | 0.059464 | 804.1254757 | 441.4824279 | 1.8214213 | 4760255 | [ARHGDIA](http://www.ncbi.nlm.nih.gov/entrez/query.fcgi?cmd=search&db=gene&term=ARHGDIA) |  |
| 0.0001478 | 0.019079 | 241.5777158 | 132.6650851 | 1.8209593 | 6040204 | [ATP11C](http://www.ncbi.nlm.nih.gov/entrez/query.fcgi?cmd=search&db=gene&term=ATP11C) |  |
| 7.56E-05 | 0.016325 | 189.1951523 | 104.0468603 | 1.8183648 | 50095 | [METRNL](http://www.ncbi.nlm.nih.gov/entrez/query.fcgi?cmd=search&db=gene&term=METRNL) |  |
| 6.71E-05 | 0.016179 | 7071.754742 | 3892.822728 | 1.8166136 | 3460181 | [MTCH1](http://www.ncbi.nlm.nih.gov/entrez/query.fcgi?cmd=search&db=gene&term=MTCH1) |  |
| 0.0005494 | 0.028094 | 191.0503349 | 105.256182 | 1.8150985 | 3310520 | [MOXD1](http://www.ncbi.nlm.nih.gov/entrez/query.fcgi?cmd=search&db=gene&term=MOXD1) |  |
| 0.0023288 | 0.052324 | 190.4175607 | 105.0631406 | 1.8124107 | 6580639 | [ACOT7](http://www.ncbi.nlm.nih.gov/entrez/query.fcgi?cmd=search&db=gene&term=ACOT7) |  |
| 3.00E-04 | 0.023298 | 541.1503695 | 298.8793038 | 1.8105983 | 5290209 | [KIF13B](http://www.ncbi.nlm.nih.gov/entrez/query.fcgi?cmd=search&db=gene&term=KIF13B) |  |
| 0.0029507 | 0.058427 | 199.2128671 | 110.1045366 | 1.8093066 | 990719 |  |  |
| 0.0039556 | 0.067148 | 842.2837582 | 467.3389499 | 1.8022974 | 1340634 | [GLB1](http://www.ncbi.nlm.nih.gov/entrez/query.fcgi?cmd=search&db=gene&term=GLB1) |  |
| 0.0018104 | 0.046575 | 1057.433654 | 586.9726674 | 1.8015041 | 2060121 | [FUCA1](http://www.ncbi.nlm.nih.gov/entrez/query.fcgi?cmd=search&db=gene&term=FUCA1) | Glycan structures - degradation, N-Glycan degradation, immunology |
| 0.0009119 | 0.03403 | 3037.107293 | 1687.567612 | 1.7996952 | 6100615 | [CD93](http://www.ncbi.nlm.nih.gov/entrez/query.fcgi?cmd=search&db=gene&term=CD93) |  |
| 3.20E-05 | 0.014558 | 2234.727339 | 1242.378814 | 1.7987488 | 6420767 | [SFRS9](http://www.ncbi.nlm.nih.gov/entrez/query.fcgi?cmd=search&db=gene&term=SFRS9) |  |
| 0.0034345 | 0.062934 | 8540.106395 | 4769.98811 | 1.7903832 | 4210703 | [RPL15](http://www.ncbi.nlm.nih.gov/entrez/query.fcgi?cmd=search&db=gene&term=RPL15) | Ribosome |
| 0.0027411 | 0.056949 | 253.2737404 | 141.4816412 | 1.7901527 | 1340241 | [C5](http://www.ncbi.nlm.nih.gov/entrez/query.fcgi?cmd=search&db=gene&term=C5) | Adhesion and Diapedesis of Granulocytes, Alternative Complement Pathway, Cells and Molecules involved in local acute inflammatory response, Classical Complement Pathway, Complement Pathway, Lectin Induced Complement Pathway, Complement and coagulation cascades, immunology, misc |
| 0.0046368 | 0.072259 | 628.0564619 | 351.2208528 | 1.7882095 | 830168 | [CHFR](http://www.ncbi.nlm.nih.gov/entrez/query.fcgi?cmd=search&db=gene&term=CHFR) | Tryptophan metabolism |
| 0.0011854 | 0.038641 | 730.3096013 | 410.2549716 | 1.7801359 | 4880324 | [TXNDC1](http://www.ncbi.nlm.nih.gov/entrez/query.fcgi?cmd=search&db=gene&term=TXNDC1) |  |
| 0.0036561 | 0.064645 | 373.4816992 | 209.9335921 | 1.7790469 | 1450603 | [C14orf106](http://www.ncbi.nlm.nih.gov/entrez/query.fcgi?cmd=search&db=gene&term=C14orf106) |  |
| 0.0033894 | 0.062509 | 419.8094582 | 236.0739472 | 1.7782964 | 5260612 | [TRIM25](http://www.ncbi.nlm.nih.gov/entrez/query.fcgi?cmd=search&db=gene&term=TRIM25) | Estrogen-responsive protein Efp controls cell cycle and breast tumors growth |
| 0.0007775 | 0.03195 | 347.3513339 | 195.3488481 | 1.7781079 | 4880392 | [KIAA1539](http://www.ncbi.nlm.nih.gov/entrez/query.fcgi?cmd=search&db=gene&term=KIAA1539) |  |
| 0.0008547 | 0.033634 | 1027.877338 | 578.1827115 | 1.7777725 | 6280672 | [TMEM49](http://www.ncbi.nlm.nih.gov/entrez/query.fcgi?cmd=search&db=gene&term=TMEM49) |  |
| 0.0006561 | 0.029637 | 16453.53768 | 9271.418212 | 1.7746517 | 2470541 | [LOC642250](http://www.ncbi.nlm.nih.gov/entrez/query.fcgi?cmd=search&db=gene&term=LOC642250) |  |
| 0.00037 | 0.024648 | 254.1937026 | 143.2855713 | 1.7740356 | 540390 | [ALPK1](http://www.ncbi.nlm.nih.gov/entrez/query.fcgi?cmd=search&db=gene&term=ALPK1) |  |
| 0.0042099 | 0.068888 | 184.8538156 | 104.2298522 | 1.7735208 | 520678 | [CD53](http://www.ncbi.nlm.nih.gov/entrez/query.fcgi?cmd=search&db=gene&term=CD53) | angiogenesis, immunology, metastasis |
| 0.004378 | 0.07037 | 217.7348231 | 122.9942098 | 1.7702851 | 6220386 | [SLC38A10](http://www.ncbi.nlm.nih.gov/entrez/query.fcgi?cmd=search&db=gene&term=SLC38A10) |  |
| 0.0028245 | 0.057692 | 340.3031553 | 192.4524575 | 1.7682453 | 580465 | [VASH1](http://www.ncbi.nlm.nih.gov/entrez/query.fcgi?cmd=search&db=gene&term=VASH1) |  |
| 0.0029273 | 0.058391 | 429.8796202 | 243.3356598 | 1.7666117 | 5900189 | [PDE1A](http://www.ncbi.nlm.nih.gov/entrez/query.fcgi?cmd=search&db=gene&term=PDE1A) | Calcium signaling pathway, Purine metabolism, Taste transduction |
| 0.0013721 | 0.040959 | 1440.617337 | 815.9570777 | 1.7655553 | 130433 | [CTTN](http://www.ncbi.nlm.nih.gov/entrez/query.fcgi?cmd=search&db=gene&term=CTTN) | Agrin in Postsynaptic Differentiation, Tight junction |
| 0.002408 | 0.053106 | 247.0846334 | 140.0950813 | 1.7636924 | 5570139 | [QPCT](http://www.ncbi.nlm.nih.gov/entrez/query.fcgi?cmd=search&db=gene&term=QPCT) |  |
| 0.0003466 | 0.024601 | 1966.626266 | 1115.964495 | 1.762266 | 2640408 | [RAB6IP1](http://www.ncbi.nlm.nih.gov/entrez/query.fcgi?cmd=search&db=gene&term=RAB6IP1) |  |
| 0.0029357 | 0.058402 | 758.1517484 | 430.314401 | 1.7618554 | 2510209 | [KDELR1](http://www.ncbi.nlm.nih.gov/entrez/query.fcgi?cmd=search&db=gene&term=KDELR1) | ADP-Ribosylation Factor |
| 0.0001874 | 0.020011 | 242.4209991 | 137.6106046 | 1.7616447 | 2370348 | [SYDE1](http://www.ncbi.nlm.nih.gov/entrez/query.fcgi?cmd=search&db=gene&term=SYDE1) |  |
| 0.00479 | 0.073 | 537.5575133 | 305.2746677 | 1.7608979 | 1400035 | [RCBTB2](http://www.ncbi.nlm.nih.gov/entrez/query.fcgi?cmd=search&db=gene&term=RCBTB2) |  |
| 0.0009012 | 0.034003 | 3215.067552 | 1826.123648 | 1.7605969 | 70730 | [GAS6](http://www.ncbi.nlm.nih.gov/entrez/query.fcgi?cmd=search&db=gene&term=GAS6) | immunology |
| 0.0018633 | 0.047349 | 390.6963726 | 222.4109815 | 1.7566416 | 6330368 | [ODF2](http://www.ncbi.nlm.nih.gov/entrez/query.fcgi?cmd=search&db=gene&term=ODF2) |  |
| 0.0008137 | 0.032916 | 184.3480404 | 104.9448894 | 1.7566176 | 870242 | [STEAP2](http://www.ncbi.nlm.nih.gov/entrez/query.fcgi?cmd=search&db=gene&term=STEAP2) |  |
| 0.0006747 | 0.030043 | 714.7831752 | 407.2861617 | 1.7549901 | 1740576 | [LMF2](http://www.ncbi.nlm.nih.gov/entrez/query.fcgi?cmd=search&db=gene&term=LMF2) |  |
| 0.0018322 | 0.046912 | 176.025979 | 100.6141204 | 1.7495157 | 6940088 | [DGKA](http://www.ncbi.nlm.nih.gov/entrez/query.fcgi?cmd=search&db=gene&term=DGKA) | Glycerolipid metabolism, Glycerophospholipid metabolism, Phosphatidylinositol signaling system, cell_cycle, cell_signaling, signal_transduction |
| 0.0004133 | 0.025615 | 169.1571663 | 96.8972799 | 1.745737 | 7160468 | [DHRS9](http://www.ncbi.nlm.nih.gov/entrez/query.fcgi?cmd=search&db=gene&term=DHRS9) |  |
| 0.0042414 | 0.069183 | 1037.513377 | 594.6150654 | 1.7448488 | 4390327 | [CLTA](http://www.ncbi.nlm.nih.gov/entrez/query.fcgi?cmd=search&db=gene&term=CLTA) | Huntington\'s disease, immunology |
| 2.73E-05 | 0.014159 | 434.5378657 | 249.1296777 | 1.7442236 | 1690465 | [LOC650369](http://www.ncbi.nlm.nih.gov/entrez/query.fcgi?cmd=search&db=gene&term=LOC650369) |  |
| 0.001736 | 0.045557 | 285.8337501 | 164.2080676 | 1.7406803 | 580592 | [CPNE8](http://www.ncbi.nlm.nih.gov/entrez/query.fcgi?cmd=search&db=gene&term=CPNE8) |  |
| 0.0014219 | 0.041638 | 197.9207652 | 113.8749473 | 1.7380536 | 3800470 | [MBOAT7](http://www.ncbi.nlm.nih.gov/entrez/query.fcgi?cmd=search&db=gene&term=MBOAT7) |  |
| 0.0016312 | 0.044476 | 2228.950698 | 1283.466551 | 1.7366644 | 7210719 | [NCSTN](http://www.ncbi.nlm.nih.gov/entrez/query.fcgi?cmd=search&db=gene&term=NCSTN) | Alzheimer\'s disease, Notch signaling pathway |
| 0.003194 | 0.060634 | 834.0061231 | 480.7951828 | 1.7346391 | 5270021 | [UBE2E2](http://www.ncbi.nlm.nih.gov/entrez/query.fcgi?cmd=search&db=gene&term=UBE2E2) | Ubiquitin mediated proteolysis |
| 0.000357 | 0.024601 | 1931.382756 | 1113.696367 | 1.7342094 | 6200431 | [AKT1](http://www.ncbi.nlm.nih.gov/entrez/query.fcgi?cmd=search&db=gene&term=AKT1) | Actions of Nitric Oxide in the Heart, AKT Signaling Pathway, Apoptotic Signaling in Response to DNA Damage, B Cell Survival Pathway, Control of skeletal myogenesis by HDAC & calcium/calmodulin-dependent kinase (CaMK), Corticosteroids and cardioprotection, Human Cytomegalovirus and Map Kinase Pathways, Hypoxia and p53 in the Cardiovascular system, IL 4 signaling pathway, IL-2 Receptor Beta Chain in T cell Activation, Inactivation of Gsk3 by AKT causes accumulation of b-catenin in Alveolar Macrophages, Influence of Ras and Rho proteins on G1 to S Transition, Inhibition of Cellular Proliferation by Gleevec, mTOR Signaling Pathway, Multiple antiapoptotic pathways from IGF-1R signaling lead to BAD phosphorylation, NFAT and Hypertrophy of the heart (Transcription in the broken heart), Phosphoinositides and their downstream targets., Phospholipase C Signaling Pathway, Phospholipids as signalling intermediaries, PTEN dependent cell cycle arrest and apoptosis, Ras Signaling Pathway, Regulation ... |
| 0.0001846 | 0.019997 | 192.2021914 | 110.9201573 | 1.7327977 | 2490328 | [LOC731486](http://www.ncbi.nlm.nih.gov/entrez/query.fcgi?cmd=search&db=gene&term=LOC731486) |  |
| 0.0002043 | 0.020451 | 221.4029755 | 127.8841331 | 1.7312779 | 7150397 | [PCDH19](http://www.ncbi.nlm.nih.gov/entrez/query.fcgi?cmd=search&db=gene&term=PCDH19) |  |
| 0.0041501 | 0.068459 | 626.5555563 | 361.9938619 | 1.7308458 | 4560064 | [GLB1](http://www.ncbi.nlm.nih.gov/entrez/query.fcgi?cmd=search&db=gene&term=GLB1) |  |
| 0.0011188 | 0.037826 | 1100.656537 | 636.2595325 | 1.7298861 | 6560484 | [VPS41](http://www.ncbi.nlm.nih.gov/entrez/query.fcgi?cmd=search&db=gene&term=VPS41) |  |
| 0.0013439 | 0.040575 | 368.9625649 | 213.8885766 | 1.7250223 | 4050554 | [NMI](http://www.ncbi.nlm.nih.gov/entrez/query.fcgi?cmd=search&db=gene&term=NMI) | IL-2 Receptor Beta Chain in T cell Activation, IL-7 Signal Transduction, signal_transduction |
| 0.0005812 | 0.028704 | 380.2661919 | 220.5403033 | 1.7242481 | 780626 | [NBPF3](http://www.ncbi.nlm.nih.gov/entrez/query.fcgi?cmd=search&db=gene&term=NBPF3) |  |
| 0.0001127 | 0.018033 | 846.1176783 | 492.6574556 | 1.7174564 | 3610553 | [GUSB](http://www.ncbi.nlm.nih.gov/entrez/query.fcgi?cmd=search&db=gene&term=GUSB) | Glycan structures - degradation, Glycosaminoglycan degradation, Pentose and glucuronate interconversions, Porphyrin and chlorophyll metabolism, Starch and sucrose metabolism, immunology |
| 2.96E-05 | 0.014558 | 500.3212109 | 291.4229068 | 1.7168218 | 5870482 | [JARID1B](http://www.ncbi.nlm.nih.gov/entrez/query.fcgi?cmd=search&db=gene&term=JARID1B) |  |
| 0.0045749 | 0.071982 | 870.9130256 | 508.3110407 | 1.7133467 | 4280041 | [SSR2](http://www.ncbi.nlm.nih.gov/entrez/query.fcgi?cmd=search&db=gene&term=SSR2) |  |
| 3.31E-05 | 0.014558 | 572.6576338 | 334.2947997 | 1.7130318 | 6220477 | [USP3](http://www.ncbi.nlm.nih.gov/entrez/query.fcgi?cmd=search&db=gene&term=USP3) |  |
| 0.004383 | 0.070373 | 185.952102 | 108.6519 | 1.7114482 | 4290403 | [CMTM7](http://www.ncbi.nlm.nih.gov/entrez/query.fcgi?cmd=search&db=gene&term=CMTM7) |  |
| 0.0023394 | 0.052389 | 2108.910078 | 1233.500859 | 1.7096949 | 5270435 | [FNBP1](http://www.ncbi.nlm.nih.gov/entrez/query.fcgi?cmd=search&db=gene&term=FNBP1) |  |
| 0.0047736 | 0.072968 | 985.8892253 | 577.0733905 | 1.7084295 | 20427 | [RNH1](http://www.ncbi.nlm.nih.gov/entrez/query.fcgi?cmd=search&db=gene&term=RNH1) |  |
| 0.0011908 | 0.038645 | 2089.473505 | 1223.151163 | 1.7082709 | 670673 | [BCL2L1](http://www.ncbi.nlm.nih.gov/entrez/query.fcgi?cmd=search&db=gene&term=BCL2L1) | Apoptotic Signaling in Response to DNA Damage, IL-2 Receptor Beta Chain in T cell Activation, Opposing roles of AIF in Apoptosis and Cell Survival, Ras Signaling Pathway, Regulation of BAD phosphorylation, Role of Mitochondria in Apoptotic Signaling, Amyotrophic lateral sclerosis (ALS), Apoptosis, Jak-STAT signaling pathway, Neurodegenerative Disorders, apoptosis, immunology |
| 0.0028838 | 0.058288 | 182.568379 | 107.0232651 | 1.7058756 | 7320301 | [CCR1](http://www.ncbi.nlm.nih.gov/entrez/query.fcgi?cmd=search&db=gene&term=CCR1) | Selective expression of chemokine receptors during T-cell polarization, Cytokine-cytokine receptor interaction, immunology |
| 0.0027907 | 0.057371 | 209.0089722 | 122.6692422 | 1.7038417 | 3120367 | [SQLE](http://www.ncbi.nlm.nih.gov/entrez/query.fcgi?cmd=search&db=gene&term=SQLE) | Biosynthesis of steroids, Terpenoid biosynthesis |
| 0.0027374 | 0.056949 | 1480.398141 | 868.8604676 | 1.7038388 | 3130615 | [PDCD6](http://www.ncbi.nlm.nih.gov/entrez/query.fcgi?cmd=search&db=gene&term=PDCD6) |  |
| 0.0024298 | 0.053327 | 357.9524097 | 210.2632997 | 1.7024008 | 770152 | [ZSWIM6](http://www.ncbi.nlm.nih.gov/entrez/query.fcgi?cmd=search&db=gene&term=ZSWIM6) |  |
| 0.004204 | 0.068875 | 329.0014562 | 193.7556837 | 1.6980222 | 2140228 | [CDR2](http://www.ncbi.nlm.nih.gov/entrez/query.fcgi?cmd=search&db=gene&term=CDR2) | immunology |
| 0.0001292 | 0.01832 | 3289.778441 | 1937.529045 | 1.6979247 | 940435 | [TRIM8](http://www.ncbi.nlm.nih.gov/entrez/query.fcgi?cmd=search&db=gene&term=TRIM8) |  |
| 0.0034825 | 0.063536 | 277.8103003 | 163.7616902 | 1.6964303 | 6580672 | [RBBP8](http://www.ncbi.nlm.nih.gov/entrez/query.fcgi?cmd=search&db=gene&term=RBBP8) | ATM Signaling Pathway |
| 0.0044494 | 0.070963 | 293.3642086 | 173.1169029 | 1.6946018 | 670286 | [CDK6](http://www.ncbi.nlm.nih.gov/entrez/query.fcgi?cmd=search&db=gene&term=CDK6) | Cell Cycle: G1/S Check Point , Cyclins and Cell Cycle Regulation, Estrogen-responsive protein Efp controls cell cycle and breast tumors growth, Influence of Ras and Rho proteins on G1 to S Transition, Cell cycle, cell_cycle, cell_signaling, signal_transduction |
| 0.0042499 | 0.069183 | 260.8191112 | 153.9174409 | 1.694539 | 3360112 | [TMEM2](http://www.ncbi.nlm.nih.gov/entrez/query.fcgi?cmd=search&db=gene&term=TMEM2) |  |
| 0.0049433 | 0.074081 | 1057.706125 | 624.6420908 | 1.6932995 | 3140056 | [HDAC1](http://www.ncbi.nlm.nih.gov/entrez/query.fcgi?cmd=search&db=gene&term=HDAC1) | Cell Cycle: G1/S Check Point , Control of Gene Expression by Vitamin D Receptor, Downregulated of MTA-3 in ER-negative Breast Tumors, Inhibition of Huntington\'s disease neurodegeneration by histone deacetylase inhibitors, Multi-step Regulation of Transcription by Pitx2, Overview of telomerase protein component gene hTert Transcriptional Regulation , Role of MEF2D in T-cell Apoptosis, Sumoylation by RanBP2 Regulates Transcriptional Repression, The PRC2 Complex Sets Long-term Gene Silencing Through Modification of Histone Tails, WNT Signaling Pathway, Cell cycle, Notch signaling pathway, immunology |
| 0.0031327 | 0.060298 | 1177.22919 | 695.3242757 | 1.693065 | 1570538 | [GTF2E2](http://www.ncbi.nlm.nih.gov/entrez/query.fcgi?cmd=search&db=gene&term=GTF2E2) | Basal transcription factors |
| 0.000788 | 0.032145 | 904.8447855 | 534.5392766 | 1.6927564 | 1740471 | [TINF2](http://www.ncbi.nlm.nih.gov/entrez/query.fcgi?cmd=search&db=gene&term=TINF2) |  |
| 0.0003396 | 0.024302 | 420.7973035 | 248.8458833 | 1.6909956 | 6840630 | [SSBP4](http://www.ncbi.nlm.nih.gov/entrez/query.fcgi?cmd=search&db=gene&term=SSBP4) |  |
| 0.0009127 | 0.03403 | 259.7954094 | 153.7713534 | 1.6894916 | 1070373 | [SNX30](http://www.ncbi.nlm.nih.gov/entrez/query.fcgi?cmd=search&db=gene&term=SNX30) |  |
| 0.003359 | 0.062181 | 324.7298097 | 192.436524 | 1.6874645 | 4220431 | [EXT1](http://www.ncbi.nlm.nih.gov/entrez/query.fcgi?cmd=search&db=gene&term=EXT1) | Glycan structures - biosynthesis 1, Heparan sulfate biosynthesis |
| 0.0010646 | 0.037046 | 269.2712271 | 159.7819855 | 1.6852415 | 4220239 | [TOP1MT](http://www.ncbi.nlm.nih.gov/entrez/query.fcgi?cmd=search&db=gene&term=TOP1MT) |  |
| 0.0015689 | 0.043774 | 813.4673415 | 482.9319547 | 1.6844347 | 6330615 | [GIMAP6](http://www.ncbi.nlm.nih.gov/entrez/query.fcgi?cmd=search&db=gene&term=GIMAP6) |  |
| 0.0005953 | 0.028704 | 698.8221324 | 415.0867191 | 1.683557 | 2120224 | [ANKRD13A](http://www.ncbi.nlm.nih.gov/entrez/query.fcgi?cmd=search&db=gene&term=ANKRD13A) |  |
| 0.0002116 | 0.020598 | 298.4794425 | 177.3076842 | 1.6833982 | 6040736 | [GPR124](http://www.ncbi.nlm.nih.gov/entrez/query.fcgi?cmd=search&db=gene&term=GPR124) |  |
| 2.83E-05 | 0.014421 | 238.0661169 | 141.6705114 | 1.6804211 | 1660681 | [CCDC102A](http://www.ncbi.nlm.nih.gov/entrez/query.fcgi?cmd=search&db=gene&term=CCDC102A) |  |
| 0.0032214 | 0.060915 | 225.5314293 | 134.5581844 | 1.6760885 | 110706 | [EFS](http://www.ncbi.nlm.nih.gov/entrez/query.fcgi?cmd=search&db=gene&term=EFS) |  |
| 0.0013375 | 0.040469 | 172.4296768 | 103.0613149 | 1.6730786 | 360114 | [CTXN1](http://www.ncbi.nlm.nih.gov/entrez/query.fcgi?cmd=search&db=gene&term=CTXN1) |  |
| 0.0030309 | 0.059219 | 217.2225118 | 130.3617742 | 1.6663053 | 2070451 | [PAQR8](http://www.ncbi.nlm.nih.gov/entrez/query.fcgi?cmd=search&db=gene&term=PAQR8) |  |
| 0.001938 | 0.048328 | 199.3691751 | 119.7340062 | 1.6651007 | 1440408 | [GEM](http://www.ncbi.nlm.nih.gov/entrez/query.fcgi?cmd=search&db=gene&term=GEM) | immunology |
| 0.0043083 | 0.06965 | 705.1169728 | 423.7243174 | 1.6640937 | 5310088 | [GPR137](http://www.ncbi.nlm.nih.gov/entrez/query.fcgi?cmd=search&db=gene&term=GPR137) |  |
| 0.0027222 | 0.05676 | 1284.500553 | 772.2873739 | 1.6632417 | 670376 | [RUSC1](http://www.ncbi.nlm.nih.gov/entrez/query.fcgi?cmd=search&db=gene&term=RUSC1) |  |
| 0.0049431 | 0.074081 | 1071.017374 | 644.8597593 | 1.6608532 | 6860373 | [RIC8A](http://www.ncbi.nlm.nih.gov/entrez/query.fcgi?cmd=search&db=gene&term=RIC8A) |  |
| 0.001436 | 0.041807 | 492.652245 | 296.8140108 | 1.6598012 | 5900286 | [ZFP90](http://www.ncbi.nlm.nih.gov/entrez/query.fcgi?cmd=search&db=gene&term=ZFP90) |  |
| 0.0004491 | 0.026228 | 165.5644864 | 99.7943551 | 1.6590566 | 2970356 | [ALDH3B1](http://www.ncbi.nlm.nih.gov/entrez/query.fcgi?cmd=search&db=gene&term=ALDH3B1) | Glycolysis / Gluconeogenesis, Histidine metabolism, Metabolism of xenobiotics by cytochrome P450, Phenylalanine metabolism, Tyrosine metabolism |
| 0.0008406 | 0.033471 | 501.7104686 | 302.9936602 | 1.6558448 | 4900193 | [ZCCHC7](http://www.ncbi.nlm.nih.gov/entrez/query.fcgi?cmd=search&db=gene&term=ZCCHC7) |  |
| 0.0002971 | 0.023197 | 182.3345972 | 110.1794468 | 1.6548876 | 2900451 | [MR1](http://www.ncbi.nlm.nih.gov/entrez/query.fcgi?cmd=search&db=gene&term=MR1) |  |
| 0.0018511 | 0.047185 | 1456.730435 | 885.3174106 | 1.6454329 | 6650053 | [MCM3](http://www.ncbi.nlm.nih.gov/entrez/query.fcgi?cmd=search&db=gene&term=MCM3) | Cell cycle |
| 0.0006048 | 0.028845 | 280.733093 | 170.6627056 | 1.6449586 | 4290050 | [ACOT7](http://www.ncbi.nlm.nih.gov/entrez/query.fcgi?cmd=search&db=gene&term=ACOT7) |  |
| 0.0014968 | 0.042622 | 600.8136115 | 365.4911385 | 1.6438527 | 60273 | [KCNJ8](http://www.ncbi.nlm.nih.gov/entrez/query.fcgi?cmd=search&db=gene&term=KCNJ8) |  |
| 0.0030171 | 0.059206 | 859.9013352 | 523.4396588 | 1.6427898 | 6420731 | [SLC20A1](http://www.ncbi.nlm.nih.gov/entrez/query.fcgi?cmd=search&db=gene&term=SLC20A1) | immunology |
| 0.0031827 | 0.060634 | 808.2507998 | 492.0868961 | 1.6424961 | 6560390 | [CRELD2](http://www.ncbi.nlm.nih.gov/entrez/query.fcgi?cmd=search&db=gene&term=CRELD2) |  |
| 0.0045611 | 0.07192 | 514.4459695 | 313.2363633 | 1.6423571 | 5860358 | [CANT1](http://www.ncbi.nlm.nih.gov/entrez/query.fcgi?cmd=search&db=gene&term=CANT1) | Purine metabolism, Pyrimidine metabolism |
| 0.0038492 | 0.065998 | 888.9710412 | 541.6625093 | 1.6411899 | 1500102 | [KDELR2](http://www.ncbi.nlm.nih.gov/entrez/query.fcgi?cmd=search&db=gene&term=KDELR2) |  |
| 0.0009425 | 0.034802 | 481.6883001 | 293.5439104 | 1.6409412 | 5900561 | [SFRS3](http://www.ncbi.nlm.nih.gov/entrez/query.fcgi?cmd=search&db=gene&term=SFRS3) |  |
| 0.0029061 | 0.058373 | 312.577207 | 191.597057 | 1.6314301 | 1050079 | [TPK1](http://www.ncbi.nlm.nih.gov/entrez/query.fcgi?cmd=search&db=gene&term=TPK1) | Thiamine metabolism |
| 0.0027619 | 0.057195 | 411.5715549 | 252.4882392 | 1.6300623 | 2940438 | [PTGER4](http://www.ncbi.nlm.nih.gov/entrez/query.fcgi?cmd=search&db=gene&term=PTGER4) | Eicosanoid Metabolism, Neuroactive ligand-receptor interaction |
| 0.0023366 | 0.052366 | 371.5702235 | 228.0633316 | 1.6292414 | 2970296 | [ALG8](http://www.ncbi.nlm.nih.gov/entrez/query.fcgi?cmd=search&db=gene&term=ALG8) | Glycan structures - biosynthesis 1, N-Glycan biosynthesis |
| 0.0024235 | 0.053326 | 186.0150175 | 114.3009926 | 1.6274138 | 4120037 | [CBL](http://www.ncbi.nlm.nih.gov/entrez/query.fcgi?cmd=search&db=gene&term=CBL) | CBL mediated ligand-induced downregulation of EGF receptors, IL-2 Receptor Beta Chain in T cell Activation, Sprouty regulation of tyrosine kinase signals, Insulin signaling pathway, Jak-STAT signaling pathway, T cell receptor signaling pathway, tsonc |
| 0.0012036 | 0.038833 | 283.5712128 | 174.2718997 | 1.6271769 | 2450037 | [KIF3B](http://www.ncbi.nlm.nih.gov/entrez/query.fcgi?cmd=search&db=gene&term=KIF3B) |  |
| 0.0001455 | 0.019068 | 394.9054177 | 243.2557487 | 1.6234166 | 1450136 | [LOC648470](http://www.ncbi.nlm.nih.gov/entrez/query.fcgi?cmd=search&db=gene&term=LOC648470) |  |
| 0.0046238 | 0.072259 | 644.7184643 | 397.6738862 | 1.621224 | 1570373 | [GATAD2A](http://www.ncbi.nlm.nih.gov/entrez/query.fcgi?cmd=search&db=gene&term=GATAD2A) |  |
| 0.0010229 | 0.03649 | 457.9059433 | 282.5113823 | 1.6208407 | 3610274 | [PJA1](http://www.ncbi.nlm.nih.gov/entrez/query.fcgi?cmd=search&db=gene&term=PJA1) | Tryptophan metabolism |
| 0.0036937 | 0.064668 | 268.5201676 | 165.968801 | 1.6178954 | 730348 | [PDE1A](http://www.ncbi.nlm.nih.gov/entrez/query.fcgi?cmd=search&db=gene&term=PDE1A) | Calcium signaling pathway, Purine metabolism, Taste transduction |
| 0.0045118 | 0.071365 | 212.1036 | 131.1914032 | 1.6167492 | 2190220 | [TTF2](http://www.ncbi.nlm.nih.gov/entrez/query.fcgi?cmd=search&db=gene&term=TTF2) |  |
| 0.0039151 | 0.066776 | 923.5274532 | 571.8823256 | 1.6148907 | 4260010 | [CYB5R3](http://www.ncbi.nlm.nih.gov/entrez/query.fcgi?cmd=search&db=gene&term=CYB5R3) | Aminosugars metabolism |
| 0.0004402 | 0.026228 | 162.7037338 | 100.8098572 | 1.6139665 | 1090390 | [OAS1](http://www.ncbi.nlm.nih.gov/entrez/query.fcgi?cmd=search&db=gene&term=OAS1) | cell_cycle, cell_signaling, immunology |
| 0.0042542 | 0.069183 | 201.8861464 | 125.0915171 | 1.6139076 | 3370112 | [RPN2](http://www.ncbi.nlm.nih.gov/entrez/query.fcgi?cmd=search&db=gene&term=RPN2) | Glycan structures - biosynthesis 1, N-Glycan biosynthesis |
| 0.0008725 | 0.033692 | 1858.476239 | 1151.845401 | 1.6134772 | 5890156 | [HNRPUL1](http://www.ncbi.nlm.nih.gov/entrez/query.fcgi?cmd=search&db=gene&term=HNRPUL1) |  |
| 0.0044698 | 0.071098 | 227.8894794 | 141.5714533 | 1.6097135 | 2570022 | [CLEC4A](http://www.ncbi.nlm.nih.gov/entrez/query.fcgi?cmd=search&db=gene&term=CLEC4A) |  |
| 0.0005233 | 0.02772 | 1769.498674 | 1100.774774 | 1.6075029 | 1470184 | [CLSTN1](http://www.ncbi.nlm.nih.gov/entrez/query.fcgi?cmd=search&db=gene&term=CLSTN1) |  |
| 0.0001977 | 0.020451 | 271.2205202 | 169.1972582 | 1.6029841 | 5290246 | [XPR1](http://www.ncbi.nlm.nih.gov/entrez/query.fcgi?cmd=search&db=gene&term=XPR1) |  |
| 0.0032404 | 0.061081 | 237.0456974 | 147.9405026 | 1.6023043 | 4570441 | [IFIH1](http://www.ncbi.nlm.nih.gov/entrez/query.fcgi?cmd=search&db=gene&term=IFIH1) | Folate biosynthesis, Starch and sucrose metabolism |
| 0.0002414 | 0.021412 | 186.121057 | 116.2784699 | 1.6006493 | 2810471 | [PTGDR](http://www.ncbi.nlm.nih.gov/entrez/query.fcgi?cmd=search&db=gene&term=PTGDR) | Neuroactive ligand-receptor interaction |
| 0.0003554 | 0.024601 | 186.6748349 | 116.9096959 | 1.5967438 | 1500180 | [TLR4](http://www.ncbi.nlm.nih.gov/entrez/query.fcgi?cmd=search&db=gene&term=TLR4) | Dendritic cells in regulating TH1 and TH2 Development, Inactivation of Gsk3 by AKT causes accumulation of b-catenin in Alveolar Macrophages, NF-kB Signaling Pathway, Toll-Like Receptor Pathway, Toll-like receptor signaling pathway, immunology |
| 0.0036725 | 0.064645 | 156.1621615 | 98.1055057 | 1.5917778 | 130156 | [PDE1B](http://www.ncbi.nlm.nih.gov/entrez/query.fcgi?cmd=search&db=gene&term=PDE1B) | Calcium signaling pathway, Purine metabolism |
| 0.0033021 | 0.061599 | 617.8806761 | 388.2312983 | 1.5915272 | 3140364 | [PTPN12](http://www.ncbi.nlm.nih.gov/entrez/query.fcgi?cmd=search&db=gene&term=PTPN12) | cell_cycle, cell_signaling |
| 0.0032537 | 0.061248 | 210.0920621 | 132.0281448 | 1.5912672 | 730632 | [LSP1](http://www.ncbi.nlm.nih.gov/entrez/query.fcgi?cmd=search&db=gene&term=LSP1) | immunology |
| 0.0048958 | 0.07384 | 162.232823 | 102.0384517 | 1.5899185 | 2030403 | [OLIG1](http://www.ncbi.nlm.nih.gov/entrez/query.fcgi?cmd=search&db=gene&term=OLIG1) |  |
| 0.0043697 | 0.070315 | 273.4753854 | 172.1840206 | 1.5882739 | 3840491 | [PELI2](http://www.ncbi.nlm.nih.gov/entrez/query.fcgi?cmd=search&db=gene&term=PELI2) |  |
| 0.0021473 | 0.050338 | 461.1323062 | 291.313796 | 1.5829402 | 6510397 | [WDR19](http://www.ncbi.nlm.nih.gov/entrez/query.fcgi?cmd=search&db=gene&term=WDR19) |  |
| 0.0033573 | 0.062181 | 7623.578453 | 4818.280007 | 1.5822199 | 4640376 | [MRLC2](http://www.ncbi.nlm.nih.gov/entrez/query.fcgi?cmd=search&db=gene&term=MRLC2) |  |
| 0.0049953 | 0.074481 | 178.2464237 | 112.8240498 | 1.579862 | 830377 | [S100A13](http://www.ncbi.nlm.nih.gov/entrez/query.fcgi?cmd=search&db=gene&term=S100A13) |  |
| 0.0009073 | 0.034003 | 279.3314309 | 176.8106631 | 1.5798336 | 6200682 | [CAPRIN2](http://www.ncbi.nlm.nih.gov/entrez/query.fcgi?cmd=search&db=gene&term=CAPRIN2) |  |
| 0.0039401 | 0.067018 | 153.9256824 | 97.7305887 | 1.5750001 | 4050768 | [CHST13](http://www.ncbi.nlm.nih.gov/entrez/query.fcgi?cmd=search&db=gene&term=CHST13) | Chondroitin sulfate biosynthesis, Glycan structures - biosynthesis 1, Sulfur metabolism |
| 0.0030318 | 0.059219 | 2592.64808 | 1648.629812 | 1.5726078 | 1300750 | [GOLGA7](http://www.ncbi.nlm.nih.gov/entrez/query.fcgi?cmd=search&db=gene&term=GOLGA7) |  |
| 0.0015886 | 0.043944 | 440.1583144 | 280.2531663 | 1.5705739 | 4810592 | [SYT15](http://www.ncbi.nlm.nih.gov/entrez/query.fcgi?cmd=search&db=gene&term=SYT15) |  |
| 0.0035128 | 0.063698 | 290.1341536 | 184.8412452 | 1.5696397 | 2940403 | [TUBB2A](http://www.ncbi.nlm.nih.gov/entrez/query.fcgi?cmd=search&db=gene&term=TUBB2A) | Gap junction |
| 0.0043859 | 0.07038 | 156.9639011 | 100.0203687 | 1.5693194 | 60349 | [MAPK13](http://www.ncbi.nlm.nih.gov/entrez/query.fcgi?cmd=search&db=gene&term=MAPK13) | Keratinocyte Differentiation, MAPKinase Signaling Pathway, Stathmin and breast cancer resistance to antimicrotubule agents, Epithelial cell signaling in Helicobacter pylori infection, Fc epsilon RI signaling pathway, GnRH signaling pathway, Leukocyte transendothelial migration, MAPK signaling pathway, Toll-like receptor signaling pathway, VEGF signaling pathway |
| 0.0017908 | 0.046294 | 197.7733388 | 126.1194935 | 1.5681425 | 5080348 | [WDFY2](http://www.ncbi.nlm.nih.gov/entrez/query.fcgi?cmd=search&db=gene&term=WDFY2) |  |
| 0.0003668 | 0.024601 | 172.32157 | 109.9324118 | 1.5675229 | 3520735 | [LZTS1](http://www.ncbi.nlm.nih.gov/entrez/query.fcgi?cmd=search&db=gene&term=LZTS1) |  |
| 0.0012666 | 0.039594 | 194.8632517 | 124.4123938 | 1.5662688 | 5810709 | [LOC644330](http://www.ncbi.nlm.nih.gov/entrez/query.fcgi?cmd=search&db=gene&term=LOC644330) |  |
| 0.0036587 | 0.064645 | 2482.092844 | 1585.751692 | 1.5652469 | 7400626 | [NFKB1](http://www.ncbi.nlm.nih.gov/entrez/query.fcgi?cmd=search&db=gene&term=NFKB1) | Acetylation and Deacetylation of RelA in The Nucleus, Activation of PKC through G protein coupled receptor, AKT Signaling Pathway, ATM Signaling Pathway, Bone Remodelling, Cadmium induces DNA synthesis and proliferation in macrophages, CD40L Signaling Pathway, Ceramide Signaling Pathway, Chaperones modulate interferon Signaling Pathway, Corticosteroids and cardioprotection, CXCR4 Signaling Pathway, Double Stranded RNA Induced Gene Expression, Erythropoietin mediated neuroprotection through NF-kB, fMLP induced chemokine gene expression in HMC-1 cells, Free Radical Induced Apoptosis, HIV-I Nef: negative effector of Fas and TNF, Human Cytomegalovirus and Map Kinase Pathways, Inactivation of Gsk3 by AKT causes accumulation of b-catenin in Alveolar Macrophages, Induction of apoptosis through DR3 and DR4/5 Death Receptors , Influence of Ras and Rho proteins on G1 to S Transition, Keratinocyte Differentiation, MAPKinase Signaling Pathway, Neuropeptides VIP and PACAP inhibit the apoptosis of a ... |
| 0.0016631 | 0.044977 | 252.9265544 | 161.7746638 | 1.5634497 | 3440025 | [AGPAT4](http://www.ncbi.nlm.nih.gov/entrez/query.fcgi?cmd=search&db=gene&term=AGPAT4) | Glycerolipid metabolism, Glycerophospholipid metabolism |
| 0.002708 | 0.056586 | 311.7122341 | 199.5085096 | 1.5624007 | 1430047 | [APEX2](http://www.ncbi.nlm.nih.gov/entrez/query.fcgi?cmd=search&db=gene&term=APEX2) |  |
| 0.0006237 | 0.029291 | 169.4557349 | 108.5846677 | 1.5605862 | 1940368 | [NRSN2](http://www.ncbi.nlm.nih.gov/entrez/query.fcgi?cmd=search&db=gene&term=NRSN2) |  |
| 0.0034671 | 0.063295 | 177.5469475 | 113.778438 | 1.5604622 | 3940541 | [PTPRO](http://www.ncbi.nlm.nih.gov/entrez/query.fcgi?cmd=search&db=gene&term=PTPRO) |  |
| 0.0038467 | 0.065994 | 287.0771883 | 184.0646883 | 1.5596538 | 1230630 | [PITPNC1](http://www.ncbi.nlm.nih.gov/entrez/query.fcgi?cmd=search&db=gene&term=PITPNC1) |  |
| 0.0031776 | 0.060634 | 201.204199 | 129.010414 | 1.5595966 | 1050746 | [HM13](http://www.ncbi.nlm.nih.gov/entrez/query.fcgi?cmd=search&db=gene&term=HM13) |  |
| 0.0001039 | 0.017752 | 161.3883275 | 103.497877 | 1.5593395 | 3190685 | [SLC35F2](http://www.ncbi.nlm.nih.gov/entrez/query.fcgi?cmd=search&db=gene&term=SLC35F2) |  |
| 0.0015281 | 0.043049 | 178.2672067 | 114.4133824 | 1.5580975 | 6420520 | [CD40](http://www.ncbi.nlm.nih.gov/entrez/query.fcgi?cmd=search&db=gene&term=CD40) | Antigen Dependent B Cell Activation, B Lymphocyte Cell Surface Molecules, Bystander B Cell Activation, CD40L Signaling Pathway, Dendritic cells in regulating TH1 and TH2 Development, Th1/Th2 Differentiation, Cell adhesion molecules (CAMs), Cytokine-cytokine receptor interaction, Toll-like receptor signaling pathway |
| 0.0014256 | 0.041657 | 558.0908835 | 358.4337808 | 1.5570265 | 4850632 | [ALG8](http://www.ncbi.nlm.nih.gov/entrez/query.fcgi?cmd=search&db=gene&term=ALG8) | Glycan structures - biosynthesis 1, N-Glycan biosynthesis |
| 0.0048864 | 0.073787 | 214.0341146 | 137.476223 | 1.556881 | 3780441 | [PHLDB2](http://www.ncbi.nlm.nih.gov/entrez/query.fcgi?cmd=search&db=gene&term=PHLDB2) |  |
| 0.0020414 | 0.049303 | 218.2977068 | 140.62873 | 1.5522981 | 3360408 | [MYO9B](http://www.ncbi.nlm.nih.gov/entrez/query.fcgi?cmd=search&db=gene&term=MYO9B) | immunology |
| 0.0009531 | 0.034956 | 175.6441523 | 113.1822416 | 1.5518702 | 2060451 | [PLCB2](http://www.ncbi.nlm.nih.gov/entrez/query.fcgi?cmd=search&db=gene&term=PLCB2) | Calcium signaling pathway, Gap junction, GnRH signaling pathway, Inositol phosphate metabolism, Long-term depression, Long-term potentiation, Phosphatidylinositol signaling system, Taste transduction, Wnt signaling pathway |
| 0.0003658 | 0.024601 | 211.9577109 | 136.5857887 | 1.5518284 | 110397 | [LOC652755](http://www.ncbi.nlm.nih.gov/entrez/query.fcgi?cmd=search&db=gene&term=LOC652755) |  |
| 0.0020043 | 0.049013 | 979.3429699 | 631.3794067 | 1.5511164 | 4230403 | [NUMB](http://www.ncbi.nlm.nih.gov/entrez/query.fcgi?cmd=search&db=gene&term=NUMB) | Notch signaling pathway |
| 0.0019891 | 0.048985 | 214.3289764 | 138.2529636 | 1.5502668 | 6020246 | [UBXD5](http://www.ncbi.nlm.nih.gov/entrez/query.fcgi?cmd=search&db=gene&term=UBXD5) |  |
| 0.0011 | 0.037499 | 169.7663888 | 109.74659 | 1.5468944 | 1820450 | [OSCAR](http://www.ncbi.nlm.nih.gov/entrez/query.fcgi?cmd=search&db=gene&term=OSCAR) |  |
| 0.0004607 | 0.026602 | 218.5558366 | 141.5778288 | 1.5437151 | 2970040 | [B3GALNT1](http://www.ncbi.nlm.nih.gov/entrez/query.fcgi?cmd=search&db=gene&term=B3GALNT1) | Glycan structures - biosynthesis 2, Glycosphingolipid biosynthesis - globoseries |
| 0.0020086 | 0.049021 | 158.6642106 | 102.9069945 | 1.5418214 | 3310142 | [C18orf17](http://www.ncbi.nlm.nih.gov/entrez/query.fcgi?cmd=search&db=gene&term=C18orf17) |  |
| 0.0046113 | 0.072259 | 6187.471494 | 4016.588055 | 1.5404795 | 4810653 | [YWHAH](http://www.ncbi.nlm.nih.gov/entrez/query.fcgi?cmd=search&db=gene&term=YWHAH) | AKT Signaling Pathway, cdc25 and chk1 Regulatory Pathway in response to DNA damage, Cell Cycle: G2/M Checkpoint, Control of skeletal myogenesis by HDAC & calcium/calmodulin-dependent kinase (CaMK), Multiple antiapoptotic pathways from IGF-1R signaling lead to BAD phosphorylation, RB Tumor Suppressor/Checkpoint Signaling in response to DNA damage, Regulation of BAD phosphorylation, Regulation of cell cycle progression by Plk3, Regulation of PGC-1a, Role of nicotinic acetylcholine receptors in the regulation of apoptosis, Signal Dependent Regulation of Myogenesis by Corepressor MITR, Cell cycle |
| 0.0042536 | 0.069183 | 346.7335389 | 225.3332154 | 1.5387591 | 2640128 | [DOCK7](http://www.ncbi.nlm.nih.gov/entrez/query.fcgi?cmd=search&db=gene&term=DOCK7) |  |
| 0.0010357 | 0.036641 | 246.4829172 | 160.2800538 | 1.5378265 | 50402 | [ENO2](http://www.ncbi.nlm.nih.gov/entrez/query.fcgi?cmd=search&db=gene&term=ENO2) | Glycolysis / Gluconeogenesis, Phenylalanine, tyrosine and tryptophan biosynthesis |
| 0.0022678 | 0.051865 | 573.4152299 | 373.1599614 | 1.5366473 | 1780661 | [MAN1B1](http://www.ncbi.nlm.nih.gov/entrez/query.fcgi?cmd=search&db=gene&term=MAN1B1) | Glycan structures - biosynthesis 1, N-Glycan biosynthesis |
| 0.0032153 | 0.060896 | 337.9827838 | 220.4147269 | 1.5333947 | 6660259 | [RBBP9](http://www.ncbi.nlm.nih.gov/entrez/query.fcgi?cmd=search&db=gene&term=RBBP9) |  |
| 0.0016891 | 0.04531 | 180.1006638 | 117.5996751 | 1.5314725 | 1440241 | [FAM26B](http://www.ncbi.nlm.nih.gov/entrez/query.fcgi?cmd=search&db=gene&term=FAM26B) |  |
| 0.0019994 | 0.049006 | 179.824583 | 117.5071453 | 1.5303289 | 3390021 | [SDSL](http://www.ncbi.nlm.nih.gov/entrez/query.fcgi?cmd=search&db=gene&term=SDSL) |  |
| 0.0034947 | 0.063552 | 4976.993503 | 3253.684929 | 1.5296483 | 4730343 | [ATP6V0E1](http://www.ncbi.nlm.nih.gov/entrez/query.fcgi?cmd=search&db=gene&term=ATP6V0E1) | ATP synthesis, Epithelial cell signaling in Helicobacter pylori infection, Oxidative phosphorylation |
| 0.0020635 | 0.049303 | 298.3954021 | 195.1551644 | 1.5290162 | 4230168 | [MANBA](http://www.ncbi.nlm.nih.gov/entrez/query.fcgi?cmd=search&db=gene&term=MANBA) | Glycan structures - degradation, N-Glycan degradation |
| 0.0027261 | 0.0568 | 200.5493112 | 131.1641752 | 1.5289946 | 6840301 | [NT5DC2](http://www.ncbi.nlm.nih.gov/entrez/query.fcgi?cmd=search&db=gene&term=NT5DC2) |  |
| 0.0001864 | 0.020011 | 181.9679529 | 119.1130197 | 1.5276915 | 3170451 | [NUDT18](http://www.ncbi.nlm.nih.gov/entrez/query.fcgi?cmd=search&db=gene&term=NUDT18) |  |
| 0.0009868 | 0.035738 | 174.666373 | 114.4086366 | 1.5266887 | 3420309 | [EVC](http://www.ncbi.nlm.nih.gov/entrez/query.fcgi?cmd=search&db=gene&term=EVC) |  |
| 0.0006802 | 0.030043 | 190.7024984 | 125.1497548 | 1.5237944 | 6840286 | [HNMT](http://www.ncbi.nlm.nih.gov/entrez/query.fcgi?cmd=search&db=gene&term=HNMT) | Histidine metabolism |
| 0.0004877 | 0.027279 | 210.4087701 | 138.3226548 | 1.5211447 | 450398 | [KIF3B](http://www.ncbi.nlm.nih.gov/entrez/query.fcgi?cmd=search&db=gene&term=KIF3B) |  |
| 0.0014032 | 0.041545 | 197.2277493 | 129.8391136 | 1.5190164 | 10187 | [CNPY3](http://www.ncbi.nlm.nih.gov/entrez/query.fcgi?cmd=search&db=gene&term=CNPY3) |  |
| 0.0018568 | 0.047225 | 235.1670912 | 155.2827751 | 1.5144442 | 840446 | [CYB561](http://www.ncbi.nlm.nih.gov/entrez/query.fcgi?cmd=search&db=gene&term=CYB561) |  |
| 0.0001176 | 0.018034 | 309.6135188 | 204.6924617 | 1.512579 | 5360736 | [HABP4](http://www.ncbi.nlm.nih.gov/entrez/query.fcgi?cmd=search&db=gene&term=HABP4) |  |
| 0.0034325 | 0.062934 | 361.3886028 | 239.0310957 | 1.5118895 | 2470070 | [TBL1X](http://www.ncbi.nlm.nih.gov/entrez/query.fcgi?cmd=search&db=gene&term=TBL1X) | Wnt signaling pathway |
| 0.0015203 | 0.042885 | 168.385354 | 111.3838424 | 1.5117575 | 6840026 | [ODF2L](http://www.ncbi.nlm.nih.gov/entrez/query.fcgi?cmd=search&db=gene&term=ODF2L) |  |
| 0.0047121 | 0.072636 | 283.2027129 | 187.4716206 | 1.5106431 | 2680278 | [FAM111A](http://www.ncbi.nlm.nih.gov/entrez/query.fcgi?cmd=search&db=gene&term=FAM111A) |  |
| 0.0006255 | 0.029291 | 170.4137511 | 112.9074713 | 1.5093222 | 1580324 | [PLCD1](http://www.ncbi.nlm.nih.gov/entrez/query.fcgi?cmd=search&db=gene&term=PLCD1) | Phospholipase C d1 in phospholipid associated cell signaling, Calcium signaling pathway, Inositol phosphate metabolism, Phosphatidylinositol signaling system, immunology |
| 0.0010809 | 0.037286 | 1326.106632 | 878.821053 | 1.5089609 | 1780402 | [SEPN1](http://www.ncbi.nlm.nih.gov/entrez/query.fcgi?cmd=search&db=gene&term=SEPN1) |  |
| 0.0048643 | 0.073632 | 243.2090204 | 161.3377941 | 1.5074522 | 1660047 | [SNX29](http://www.ncbi.nlm.nih.gov/entrez/query.fcgi?cmd=search&db=gene&term=SNX29) |  |
| 0.0002274 | 0.020993 | 166.285648 | 110.3322554 | 1.5071354 | 2600735 | [TLR6](http://www.ncbi.nlm.nih.gov/entrez/query.fcgi?cmd=search&db=gene&term=TLR6) | Toll-Like Receptor Pathway, Toll-like receptor signaling pathway |
| 0.0029479 | 0.058427 | 268.3784034 | 178.1386 | 1.5065707 | 2690639 | [AQR](http://www.ncbi.nlm.nih.gov/entrez/query.fcgi?cmd=search&db=gene&term=AQR) |  |
| 0.0006396 | 0.029369 | 159.2097901 | 105.7465598 | 1.5055789 | 4120626 | [NAALADL1](http://www.ncbi.nlm.nih.gov/entrez/query.fcgi?cmd=search&db=gene&term=NAALADL1) |  |
| 0.0014941 | 0.042622 | 152.6948949 | 101.566201 | 1.5034026 | 2510546 | [C10orf125](http://www.ncbi.nlm.nih.gov/entrez/query.fcgi?cmd=search&db=gene&term=C10orf125) |  |
| 0.0006999 | 0.030116 | 378.6820996 | 252.2432179 | 1.5012578 | 4050161 | [STX6](http://www.ncbi.nlm.nih.gov/entrez/query.fcgi?cmd=search&db=gene&term=STX6) | SNARE interactions in vesicular transport |
| 0.0046155 | 0.072259 | 427.5456574 | 285.9334841 | 1.4952626 | 2480487 | [STX6](http://www.ncbi.nlm.nih.gov/entrez/query.fcgi?cmd=search&db=gene&term=STX6) | SNARE interactions in vesicular transport |
| 5.19E-05 | 0.015414 | 161.4115328 | 108.239908 | 1.4912386 | 7000521 | [PRPS2](http://www.ncbi.nlm.nih.gov/entrez/query.fcgi?cmd=search&db=gene&term=PRPS2) | Histidine metabolism, Pentose phosphate pathway, Purine metabolism |
| 0.0046363 | 0.072259 | 194.9549873 | 131.0884609 | 1.4872017 | 20358 | [MEIS2](http://www.ncbi.nlm.nih.gov/entrez/query.fcgi?cmd=search&db=gene&term=MEIS2) | development |
| 0.0017449 | 0.045682 | 170.9581397 | 115.0818581 | 1.4855351 | 5310364 | [PAPLN](http://www.ncbi.nlm.nih.gov/entrez/query.fcgi?cmd=search&db=gene&term=PAPLN) |  |
| 0.002067 | 0.049303 | 1160.062992 | 781.7222312 | 1.4839836 | 6130424 | [AKR1A1](http://www.ncbi.nlm.nih.gov/entrez/query.fcgi?cmd=search&db=gene&term=AKR1A1) | Caprolactam degradation, Glycerolipid metabolism, Glycolysis / Gluconeogenesis |
| 0.0036813 | 0.064645 | 421.4287187 | 284.1020547 | 1.4833709 | 6220630 | [SLC35B3](http://www.ncbi.nlm.nih.gov/entrez/query.fcgi?cmd=search&db=gene&term=SLC35B3) |  |
| 0.0025274 | 0.054402 | 145.6254765 | 98.3643521 | 1.48047 | 3390612 | [TLR8](http://www.ncbi.nlm.nih.gov/entrez/query.fcgi?cmd=search&db=gene&term=TLR8) |  |
| 0.0012597 | 0.039555 | 137.2240896 | 92.744863 | 1.479587 | 1070044 | [GMIP](http://www.ncbi.nlm.nih.gov/entrez/query.fcgi?cmd=search&db=gene&term=GMIP) |  |
| 0.0041369 | 0.068387 | 180.359567 | 121.9177716 | 1.4793542 | 70541 | [REC8](http://www.ncbi.nlm.nih.gov/entrez/query.fcgi?cmd=search&db=gene&term=REC8) |  |
| 0.0012526 | 0.039505 | 371.7672028 | 251.9172397 | 1.4757513 | 5260537 | [YEATS2](http://www.ncbi.nlm.nih.gov/entrez/query.fcgi?cmd=search&db=gene&term=YEATS2) |  |
| 0.0016967 | 0.045378 | 164.4011243 | 111.4609677 | 1.4749659 | 4280273 | [GM2A](http://www.ncbi.nlm.nih.gov/entrez/query.fcgi?cmd=search&db=gene&term=GM2A) |  |
| 0.0040854 | 0.068002 | 140.763041 | 95.5756499 | 1.4727919 | 5270674 | [GPR68](http://www.ncbi.nlm.nih.gov/entrez/query.fcgi?cmd=search&db=gene&term=GPR68) |  |
| 0.0021468 | 0.050338 | 168.483042 | 114.5708608 | 1.4705575 | 1660647 | [GALIG](http://www.ncbi.nlm.nih.gov/entrez/query.fcgi?cmd=search&db=gene&term=GALIG) |  |
| 0.0017894 | 0.046294 | 169.6228197 | 115.5824029 | 1.4675488 | 6020470 | [ST8SIA4](http://www.ncbi.nlm.nih.gov/entrez/query.fcgi?cmd=search&db=gene&term=ST8SIA4) | Steps in the Glycosylation of Mammalian N-linked Oligosaccarides |
| 0.0030311 | 0.059219 | 1895.196988 | 1292.521913 | 1.4662784 | 3290338 | [POLR2A](http://www.ncbi.nlm.nih.gov/entrez/query.fcgi?cmd=search&db=gene&term=POLR2A) | CARM1 and Regulation of the Estrogen Receptor, Chromatin Remodeling by hSWI/SNF ATP-dependent Complexes, Nuclear receptors coordinate the activities of chromatin remodeling complexes and coactivators to facilitate initiation of transcription in carcinoma cells, Repression of Pain Sensation by the Transcriptional Regulator DREAM, Telomeres, Telomerase, Cellular Aging, and Immortality, The information-processing pathway at the IFN-beta enhancer, Purine metabolism, Pyrimidine metabolism, RNA polymerase |
| 0.0037775 | 0.065289 | 176.8619947 | 120.6605738 | 1.4657812 | 430372 | [SPG3A](http://www.ncbi.nlm.nih.gov/entrez/query.fcgi?cmd=search&db=gene&term=SPG3A) | immunology |
| 0.0011238 | 0.037826 | 8633.91246 | 5891.931438 | 1.465379 | 7650333 | [PSAP](http://www.ncbi.nlm.nih.gov/entrez/query.fcgi?cmd=search&db=gene&term=PSAP) | immunology |
| 0.0001025 | 0.017719 | 610.6735041 | 416.7719895 | 1.465246 | 6290379 | [FAM156A](http://www.ncbi.nlm.nih.gov/entrez/query.fcgi?cmd=search&db=gene&term=FAM156A) |  |
| 0.0017521 | 0.045805 | 151.3032485 | 103.4113757 | 1.46312 | 1820528 | [PAPSS1](http://www.ncbi.nlm.nih.gov/entrez/query.fcgi?cmd=search&db=gene&term=PAPSS1) | Purine metabolism, Selenoamino acid metabolism, Sulfur metabolism |
| 0.0006929 | 0.030043 | 168.8858175 | 115.6673096 | 1.4600998 | 1850041 | [DPM2](http://www.ncbi.nlm.nih.gov/entrez/query.fcgi?cmd=search&db=gene&term=DPM2) |  |
| 0.0030767 | 0.059695 | 143.8200118 | 98.5103951 | 1.4599476 | 5900364 |  |  |
| 0.003511 | 0.063698 | 151.9455293 | 104.2744657 | 1.4571691 | 4290064 | [VCAM1](http://www.ncbi.nlm.nih.gov/entrez/query.fcgi?cmd=search&db=gene&term=VCAM1) | Adhesion and Diapedesis of Lymphocytes, Cells and Molecules involved in local acute inflammatory response, Cell adhesion molecules (CAMs), Leukocyte transendothelial migration, angiogenesis, cell_signaling, immunology, metastasis |
| 0.0012206 | 0.039002 | 356.6660368 | 245.2816275 | 1.4541082 | 6220164 | [CENTG3](http://www.ncbi.nlm.nih.gov/entrez/query.fcgi?cmd=search&db=gene&term=CENTG3) |  |
| 0.0047704 | 0.072968 | 2056.726539 | 1416.645813 | 1.4518283 | 4490017 | [GLTP](http://www.ncbi.nlm.nih.gov/entrez/query.fcgi?cmd=search&db=gene&term=GLTP) |  |
| 0.0034295 | 0.062934 | 593.7717591 | 409.0575046 | 1.4515606 | 5910095 | [CCDC115](http://www.ncbi.nlm.nih.gov/entrez/query.fcgi?cmd=search&db=gene&term=CCDC115) |  |
| 0.001852 | 0.047185 | 817.1719853 | 563.7597847 | 1.4495039 | 7380110 | [CDK4](http://www.ncbi.nlm.nih.gov/entrez/query.fcgi?cmd=search&db=gene&term=CDK4) | Cell Cycle: G1/S Check Point , Cyclins and Cell Cycle Regulation, Influence of Ras and Rho proteins on G1 to S Transition, p53 Signaling Pathway, RB Tumor Suppressor/Checkpoint Signaling in response to DNA damage, Cell cycle, T cell receptor signaling pathway, Tight junction, immunology, tsonc |
| 0.0003945 | 0.025294 | 149.2253556 | 103.1009301 | 1.4473716 | 4280048 | [WFDC3](http://www.ncbi.nlm.nih.gov/entrez/query.fcgi?cmd=search&db=gene&term=WFDC3) |  |
| 0.000659 | 0.029691 | 159.4043331 | 110.3500436 | 1.4445335 | 7380376 | [CHEK2](http://www.ncbi.nlm.nih.gov/entrez/query.fcgi?cmd=search&db=gene&term=CHEK2) | ATM Signaling Pathway, Cell Cycle: G2/M Checkpoint, Regulation of cell cycle progression by Plk3, Role of BRCA1, BRCA2 and ATR in Cancer Susceptibility, Cell cycle |
| 0.0005794 | 0.028704 | 139.116431 | 96.3452035 | 1.4439373 | 2370768 | [DMRT3](http://www.ncbi.nlm.nih.gov/entrez/query.fcgi?cmd=search&db=gene&term=DMRT3) |  |
| 0.0011401 | 0.038062 | 136.8393951 | 94.7919623 | 1.4435759 | 5290176 | [SIGLEC1](http://www.ncbi.nlm.nih.gov/entrez/query.fcgi?cmd=search&db=gene&term=SIGLEC1) | Cell adhesion molecules (CAMs) |
| 0.0029037 | 0.058373 | 229.3780814 | 158.9704359 | 1.4428977 | 7200242 | [SULF2](http://www.ncbi.nlm.nih.gov/entrez/query.fcgi?cmd=search&db=gene&term=SULF2) |  |
| 0.0013979 | 0.041473 | 200.8990383 | 139.4014686 | 1.4411544 | 7160373 | [FUT4](http://www.ncbi.nlm.nih.gov/entrez/query.fcgi?cmd=search&db=gene&term=FUT4) | Glycan structures - biosynthesis 2, Glycosphingolipid biosynthesis - neo-lactoseries |
| 0.0014868 | 0.042622 | 185.5495873 | 128.9790452 | 1.4386026 | 1500669 | [MYO1B](http://www.ncbi.nlm.nih.gov/entrez/query.fcgi?cmd=search&db=gene&term=MYO1B) |  |
| 3.16E-05 | 0.014558 | 232.4841798 | 162.0772629 | 1.4344034 | 2480241 | [ATP6V1C1](http://www.ncbi.nlm.nih.gov/entrez/query.fcgi?cmd=search&db=gene&term=ATP6V1C1) | ATP synthesis, Epithelial cell signaling in Helicobacter pylori infection, Oxidative phosphorylation |
| 0.0030243 | 0.059206 | 8494.083158 | 5924.030753 | 1.4338351 | 6370661 | [AP2S1](http://www.ncbi.nlm.nih.gov/entrez/query.fcgi?cmd=search&db=gene&term=AP2S1) |  |
| 0.004321 | 0.069735 | 200.3578384 | 139.823032 | 1.4329387 | 5260288 | [DUSP6](http://www.ncbi.nlm.nih.gov/entrez/query.fcgi?cmd=search&db=gene&term=DUSP6) | Regulation of MAP Kinase Pathways Through Dual Specificity Phosphatases, MAPK signaling pathway |
| 0.0047198 | 0.072636 | 165.266137 | 115.3451919 | 1.4327961 | 620577 | [ACSL5](http://www.ncbi.nlm.nih.gov/entrez/query.fcgi?cmd=search&db=gene&term=ACSL5) | Adipocytokine signaling pathway, Fatty acid metabolism, PPAR signaling pathway |
| 0.0042694 | 0.069314 | 137.5084426 | 96.151096 | 1.4301287 | 4180747 |  |  |
| 0.0020836 | 0.049564 | 16097.09859 | 11275.69266 | 1.4275929 | 3610064 | [NACA](http://www.ncbi.nlm.nih.gov/entrez/query.fcgi?cmd=search&db=gene&term=NACA) |  |
| 0.0032585 | 0.061248 | 3872.197248 | 2712.750156 | 1.4274065 | 4230132 | [ILK](http://www.ncbi.nlm.nih.gov/entrez/query.fcgi?cmd=search&db=gene&term=ILK) | PTEN dependent cell cycle arrest and apoptosis, Focal adhesion, PPAR signaling pathway, cell_cycle, cell_signaling, signal_transduction |
| 0.0002004 | 0.020451 | 7979.987877 | 5603.390426 | 1.4241356 | 6200086 | [PSAP](http://www.ncbi.nlm.nih.gov/entrez/query.fcgi?cmd=search&db=gene&term=PSAP) | immunology |
| 0.0037385 | 0.065138 | 183.1255069 | 128.7348898 | 1.422501 | 4060576 | [SLC35D2](http://www.ncbi.nlm.nih.gov/entrez/query.fcgi?cmd=search&db=gene&term=SLC35D2) |  |
| 0.004775 | 0.072968 | 154.7648086 | 108.9502932 | 1.4205084 | 580411 | [LAX1](http://www.ncbi.nlm.nih.gov/entrez/query.fcgi?cmd=search&db=gene&term=LAX1) |  |
| 0.0037654 | 0.065289 | 185.6898657 | 130.8030944 | 1.4196137 | 6480131 | [ADAM15](http://www.ncbi.nlm.nih.gov/entrez/query.fcgi?cmd=search&db=gene&term=ADAM15) |  |
| 0.0026967 | 0.056512 | 149.129123 | 105.3930355 | 1.4149808 | 1010035 | [NLRP3](http://www.ncbi.nlm.nih.gov/entrez/query.fcgi?cmd=search&db=gene&term=NLRP3) |  |
| 0.0047831 | 0.072968 | 143.3043147 | 101.4197674 | 1.4129821 | 4290390 | [VCAM1](http://www.ncbi.nlm.nih.gov/entrez/query.fcgi?cmd=search&db=gene&term=VCAM1) | Adhesion and Diapedesis of Lymphocytes, Cells and Molecules involved in local acute inflammatory response, Cell adhesion molecules (CAMs), Leukocyte transendothelial migration, angiogenesis, cell_signaling, immunology, metastasis |
| 0.0035235 | 0.063701 | 736.6710985 | 521.8072988 | 1.4117685 | 1050082 | [KIAA1147](http://www.ncbi.nlm.nih.gov/entrez/query.fcgi?cmd=search&db=gene&term=KIAA1147) |  |
| 0.0002284 | 0.020993 | 248.8556093 | 176.5724649 | 1.4093682 | 670026 | [CRLF3](http://www.ncbi.nlm.nih.gov/entrez/query.fcgi?cmd=search&db=gene&term=CRLF3) |  |
| 7.44E-05 | 0.016325 | 143.3543405 | 101.9143412 | 1.406616 | 3800463 | [SLC29A3](http://www.ncbi.nlm.nih.gov/entrez/query.fcgi?cmd=search&db=gene&term=SLC29A3) |  |
| 0.0003977 | 0.025361 | 202.3722535 | 143.8764708 | 1.4065695 | 4570026 | [CASP2](http://www.ncbi.nlm.nih.gov/entrez/query.fcgi?cmd=search&db=gene&term=CASP2) | Caspase Cascade in Apoptosis, HIV-I Nef: negative effector of Fas and TNF, TNF/Stress Related Signaling, TNFR1 Signaling Pathway, MAPK signaling pathway, apoptosis, immunology |
| 0.0008627 | 0.033634 | 197.6440347 | 140.6382704 | 1.4053361 | 670273 | [DPYD](http://www.ncbi.nlm.nih.gov/entrez/query.fcgi?cmd=search&db=gene&term=DPYD) | beta-Alanine metabolism, Pantothenate and CoA biosynthesis, Pyrimidine metabolism, immunology, pharmacology |
| 0.0022322 | 0.051415 | 140.2073904 | 100.0224845 | 1.4017587 | 4900086 | [HAPLN3](http://www.ncbi.nlm.nih.gov/entrez/query.fcgi?cmd=search&db=gene&term=HAPLN3) |  |
| 0.0015406 | 0.043264 | 139.3376697 | 99.5674289 | 1.3994302 | 7650209 | [BMF](http://www.ncbi.nlm.nih.gov/entrez/query.fcgi?cmd=search&db=gene&term=BMF) |  |
| 0.0001073 | 0.017911 | 155.7870916 | 111.3608358 | 1.3989397 | 2490719 | [STK17B](http://www.ncbi.nlm.nih.gov/entrez/query.fcgi?cmd=search&db=gene&term=STK17B) | apoptosis, signal_transduction |
| 0.0009379 | 0.034702 | 136.4566022 | 97.6687908 | 1.3971362 | 6060324 | [PRND](http://www.ncbi.nlm.nih.gov/entrez/query.fcgi?cmd=search&db=gene&term=PRND) |  |
| 0.0029622 | 0.058608 | 173.574538 | 124.3116994 | 1.3962848 | 1440630 | [CEP110](http://www.ncbi.nlm.nih.gov/entrez/query.fcgi?cmd=search&db=gene&term=CEP110) |  |
| 0.0026075 | 0.055362 | 181.5462632 | 130.0255056 | 1.3962358 | 4480519 | [EDEM2](http://www.ncbi.nlm.nih.gov/entrez/query.fcgi?cmd=search&db=gene&term=EDEM2) | Aminosugars metabolism, Nucleotide sugars metabolism |
| 0.0026869 | 0.05636 | 143.8184431 | 103.4703006 | 1.389949 | 3130609 | [INPP5D](http://www.ncbi.nlm.nih.gov/entrez/query.fcgi?cmd=search&db=gene&term=INPP5D) | B cell receptor signaling pathway, Fc epsilon RI signaling pathway, Insulin signaling pathway, Phosphatidylinositol signaling system, cell_cycle, cell_signaling, immunology |
| 0.00035 | 0.024601 | 145.973657 | 105.1114372 | 1.3887514 | 4070048 | [NPHP4](http://www.ncbi.nlm.nih.gov/entrez/query.fcgi?cmd=search&db=gene&term=NPHP4) |  |
| 0.0007187 | 0.030519 | 142.4631802 | 102.6074737 | 1.3884289 | 2970020 | [NHS](http://www.ncbi.nlm.nih.gov/entrez/query.fcgi?cmd=search&db=gene&term=NHS) |  |
| 0.0030189 | 0.059206 | 2048.817183 | 1478.93195 | 1.3853357 | 2060088 | [CLCN7](http://www.ncbi.nlm.nih.gov/entrez/query.fcgi?cmd=search&db=gene&term=CLCN7) |  |
| 0.0012529 | 0.039505 | 468.3959828 | 338.349166 | 1.3843568 | 1190040 | [GIMAP8](http://www.ncbi.nlm.nih.gov/entrez/query.fcgi?cmd=search&db=gene&term=GIMAP8) |  |
| 0.0027654 | 0.057195 | 148.0541096 | 107.097194 | 1.3824275 | 830687 | [CHN2](http://www.ncbi.nlm.nih.gov/entrez/query.fcgi?cmd=search&db=gene&term=CHN2) | signal_transduction |
| 0.0010239 | 0.03649 | 2765.72888 | 2006.5139 | 1.3783751 | 4560056 | [NONO](http://www.ncbi.nlm.nih.gov/entrez/query.fcgi?cmd=search&db=gene&term=NONO) | RNA polymerase III transcription |
| 0.0041036 | 0.068069 | 139.290933 | 101.0700345 | 1.3781625 | 6110747 | [GIMAP2](http://www.ncbi.nlm.nih.gov/entrez/query.fcgi?cmd=search&db=gene&term=GIMAP2) |  |
| 0.0003992 | 0.025361 | 134.2359457 | 97.4306839 | 1.3777584 | 2350168 | [SCN9A](http://www.ncbi.nlm.nih.gov/entrez/query.fcgi?cmd=search&db=gene&term=SCN9A) |  |
| 0.0005528 | 0.028179 | 137.0792339 | 99.6799585 | 1.3751935 | 1690170 | [PLEKHG2](http://www.ncbi.nlm.nih.gov/entrez/query.fcgi?cmd=search&db=gene&term=PLEKHG2) |  |
| 0.0003786 | 0.024823 | 151.8482953 | 110.9425687 | 1.3687108 | 2140746 | [PVRL2](http://www.ncbi.nlm.nih.gov/entrez/query.fcgi?cmd=search&db=gene&term=PVRL2) | Adherens junction, Cell adhesion molecules (CAMs), immunology |
| 0.0001202 | 0.018034 | 148.33694 | 108.6635629 | 1.3651029 | 5670040 | [ELOVL6](http://www.ncbi.nlm.nih.gov/entrez/query.fcgi?cmd=search&db=gene&term=ELOVL6) |  |
| 0.0049209 | 0.074056 | 259.9431643 | 190.6256638 | 1.3636315 | 7040431 | [PRDM8](http://www.ncbi.nlm.nih.gov/entrez/query.fcgi?cmd=search&db=gene&term=PRDM8) |  |
| 0.0030698 | 0.059639 | 136.9091091 | 100.6778984 | 1.3598725 | 1770593 | [CH25H](http://www.ncbi.nlm.nih.gov/entrez/query.fcgi?cmd=search&db=gene&term=CH25H) |  |
| 0.0005473 | 0.028036 | 196.895834 | 144.9156304 | 1.3586929 | 5050500 | [ZBTB9](http://www.ncbi.nlm.nih.gov/entrez/query.fcgi?cmd=search&db=gene&term=ZBTB9) |  |
| 0.001121 | 0.037826 | 138.5479086 | 102.3462985 | 1.3537168 | 4070538 | [TDP1](http://www.ncbi.nlm.nih.gov/entrez/query.fcgi?cmd=search&db=gene&term=TDP1) |  |
| 0.0038818 | 0.066478 | 141.0061216 | 104.358007 | 1.3511768 | 2490537 | [TNFRSF1B](http://www.ncbi.nlm.nih.gov/entrez/query.fcgi?cmd=search&db=gene&term=TNFRSF1B) | TNFR2 Signaling Pathway, Adipocytokine signaling pathway, Cytokine-cytokine receptor interaction, immunology |
| 0.0011851 | 0.038641 | 139.8371743 | 103.5735641 | 1.3501242 | 2070392 | [SLFN12](http://www.ncbi.nlm.nih.gov/entrez/query.fcgi?cmd=search&db=gene&term=SLFN12) |  |
| 0.0031433 | 0.060342 | 139.4750002 | 103.4205019 | 1.3486204 | 3940280 | [MLKL](http://www.ncbi.nlm.nih.gov/entrez/query.fcgi?cmd=search&db=gene&term=MLKL) |  |
| 0.0019981 | 0.049006 | 512.1207566 | 380.5209777 | 1.3458411 | 60309 | [ASB3](http://www.ncbi.nlm.nih.gov/entrez/query.fcgi?cmd=search&db=gene&term=ASB3) |  |
| 0.0023272 | 0.052324 | 134.4833667 | 99.9278889 | 1.3458041 | 2810373 | [FAM20A](http://www.ncbi.nlm.nih.gov/entrez/query.fcgi?cmd=search&db=gene&term=FAM20A) |  |
| 0.0003265 | 0.023767 | 1193.890345 | 888.5217552 | 1.3436816 | 5270110 | [EIF4A3](http://www.ncbi.nlm.nih.gov/entrez/query.fcgi?cmd=search&db=gene&term=EIF4A3) |  |
| 0.0010663 | 0.037046 | 253.4054948 | 188.6019008 | 1.3435999 | 240424 | [CABIN1](http://www.ncbi.nlm.nih.gov/entrez/query.fcgi?cmd=search&db=gene&term=CABIN1) | Control of skeletal myogenesis by HDAC & calcium/calmodulin-dependent kinase (CaMK), Role of MEF2D in T-cell Apoptosis |
| 0.0048564 | 0.073632 | 129.5040541 | 96.5107266 | 1.3418618 | 7200768 | [ITGA4](http://www.ncbi.nlm.nih.gov/entrez/query.fcgi?cmd=search&db=gene&term=ITGA4) | Adhesion and Diapedesis of Lymphocytes, Adhesion Molecules on Lymphocyte, Cells and Molecules involved in local acute inflammatory response, Monocyte and its Surface Molecules, Cell adhesion molecules (CAMs), ECM-receptor interaction, Focal adhesion, Hematopoietic cell lineage, Leukocyte transendothelial migration, Regulation of actin cytoskeleton, cell_signaling, immunology, metastasis |
| 0.0025029 | 0.054211 | 152.0681551 | 113.4779041 | 1.3400684 | 6660446 | [WDR51A](http://www.ncbi.nlm.nih.gov/entrez/query.fcgi?cmd=search&db=gene&term=WDR51A) |  |
| 0.0010447 | 0.036768 | 132.4778538 | 98.9186312 | 1.3392609 | 4570224 | [C17orf53](http://www.ncbi.nlm.nih.gov/entrez/query.fcgi?cmd=search&db=gene&term=C17orf53) |  |
| 0.0030401 | 0.059262 | 148.1333804 | 110.746309 | 1.337592 | 4200070 | [MGC39900](http://www.ncbi.nlm.nih.gov/entrez/query.fcgi?cmd=search&db=gene&term=MGC39900) |  |
| 0.0032718 | 0.061309 | 610.2242753 | 459.184786 | 1.3289296 | 6420017 | [SIN3A](http://www.ncbi.nlm.nih.gov/entrez/query.fcgi?cmd=search&db=gene&term=SIN3A) | NA |
| 0.0036832 | 0.064645 | 653.8839175 | 492.60234 | 1.3274073 | 4640435 | [CHD4](http://www.ncbi.nlm.nih.gov/entrez/query.fcgi?cmd=search&db=gene&term=CHD4) | NA, DNA_replication, transcription |
| 0.0042504 | 0.069183 | 153.5550833 | 116.5288941 | 1.3177426 | 5900050 | [LOC653800](http://www.ncbi.nlm.nih.gov/entrez/query.fcgi?cmd=search&db=gene&term=LOC653800) |  |
| 0.0001596 | 0.019731 | 162.6238415 | 123.4290673 | 1.317549 | 3420075 | [SPIN4](http://www.ncbi.nlm.nih.gov/entrez/query.fcgi?cmd=search&db=gene&term=SPIN4) |  |
| 0.000435 | 0.026228 | 336.0189055 | 255.187909 | 1.3167509 | 3440243 | [RHOJ](http://www.ncbi.nlm.nih.gov/entrez/query.fcgi?cmd=search&db=gene&term=RHOJ) |  |
| 0.0046107 | 0.072259 | 132.4801745 | 100.8166879 | 1.3140699 | 7040035 | [OAS1](http://www.ncbi.nlm.nih.gov/entrez/query.fcgi?cmd=search&db=gene&term=OAS1) | cell_cycle, cell_signaling, immunology |
| 0.0002214 | 0.020839 | 140.0142579 | 106.8697232 | 1.3101396 | 2320598 | [NDP](http://www.ncbi.nlm.nih.gov/entrez/query.fcgi?cmd=search&db=gene&term=NDP) | immunology |
| 0.0010569 | 0.036985 | 122.8312329 | 93.8745236 | 1.3084619 | 7210400 | [STEAP1](http://www.ncbi.nlm.nih.gov/entrez/query.fcgi?cmd=search&db=gene&term=STEAP1) |  |
| 0.000122 | 0.018034 | 142.2348751 | 109.0793847 | 1.3039574 | 2710400 | [DSC2](http://www.ncbi.nlm.nih.gov/entrez/query.fcgi?cmd=search&db=gene&term=DSC2) | Cell Communication |
| 0.0013856 | 0.041192 | 120.9235431 | 92.8299128 | 1.3026355 | 5810022 | [MLKL](http://www.ncbi.nlm.nih.gov/entrez/query.fcgi?cmd=search&db=gene&term=MLKL) |  |
| 0.0035655 | 0.063887 | 133.8266712 | 102.9461335 | 1.2999679 | 6450594 | [CD79B](http://www.ncbi.nlm.nih.gov/entrez/query.fcgi?cmd=search&db=gene&term=CD79B) | B Cell Receptor Complex, BCR Signaling Pathway, CTCF: First Multivalent Nuclear Factor, B cell receptor signaling pathway, immunology |
| 0.0018831 | 0.047602 | 192.0744348 | 148.1140421 | 1.296801 | 6840753 | [SPTLC2](http://www.ncbi.nlm.nih.gov/entrez/query.fcgi?cmd=search&db=gene&term=SPTLC2) | Sphingolipid metabolism |
| 0.0041863 | 0.068783 | 137.8651798 | 106.7491653 | 1.2914872 | 3310491 | [IRAK4](http://www.ncbi.nlm.nih.gov/entrez/query.fcgi?cmd=search&db=gene&term=IRAK4) | Apoptosis, Toll-like receptor signaling pathway |
| 7.70E-06 | 0.012163 | 112.2611057 | 87.0039298 | 1.2902993 | 2970661 | [KLF4](http://www.ncbi.nlm.nih.gov/entrez/query.fcgi?cmd=search&db=gene&term=KLF4) |  |
| 0.00406 | 0.06788 | 157.150973 | 121.8142044 | 1.2900874 | 4180369 | [ATP2B4](http://www.ncbi.nlm.nih.gov/entrez/query.fcgi?cmd=search&db=gene&term=ATP2B4) | Calcium signaling pathway |
| 0.0022673 | 0.051865 | 407.4051119 | 315.9824118 | 1.2893284 | 20603 | [ZNF700](http://www.ncbi.nlm.nih.gov/entrez/query.fcgi?cmd=search&db=gene&term=ZNF700) |  |
| 0.000713 | 0.030455 | 121.023563 | 94.1358336 | 1.2856269 | 6940181 | [FAM24B](http://www.ncbi.nlm.nih.gov/entrez/query.fcgi?cmd=search&db=gene&term=FAM24B) |  |
| 0.0033736 | 0.062332 | 130.5085355 | 101.6513941 | 1.2838834 | 3940050 | [FLJ46906](http://www.ncbi.nlm.nih.gov/entrez/query.fcgi?cmd=search&db=gene&term=FLJ46906) |  |
| 0.0025025 | 0.054211 | 127.9714253 | 99.7150605 | 1.2833711 | 4010022 | [NEK11](http://www.ncbi.nlm.nih.gov/entrez/query.fcgi?cmd=search&db=gene&term=NEK11) |  |
| 0.0030995 | 0.059971 | 192.94429 | 150.3838217 | 1.2830123 | 2630369 | [CHST14](http://www.ncbi.nlm.nih.gov/entrez/query.fcgi?cmd=search&db=gene&term=CHST14) | Chondroitin sulfate biosynthesis, Glycan structures - biosynthesis 1 |
| 0.0011924 | 0.038653 | 122.5179224 | 95.5955613 | 1.2816277 | 2650612 | [LAMA3](http://www.ncbi.nlm.nih.gov/entrez/query.fcgi?cmd=search&db=gene&term=LAMA3) | Agrin in Postsynaptic Differentiation, Cell Communication, ECM-receptor interaction, Focal adhesion, immunology |
| 0.0027006 | 0.056544 | 158.1482672 | 123.4097929 | 1.2814888 | 1440037 | [BID](http://www.ncbi.nlm.nih.gov/entrez/query.fcgi?cmd=search&db=gene&term=BID) | Apoptotic Signaling in Response to DNA Damage, HIV-I Nef: negative effector of Fas and TNF, Induction of apoptosis through DR3 and DR4/5 Death Receptors , Role of Mitochondria in Apoptotic Signaling, Apoptosis, Natural killer cell mediated cytotoxicity, immunology |
| 0.0044499 | 0.070963 | 134.6608027 | 105.2420325 | 1.2795344 | 5870390 | [TOP1MT](http://www.ncbi.nlm.nih.gov/entrez/query.fcgi?cmd=search&db=gene&term=TOP1MT) |  |
| 0.0017033 | 0.045429 | 122.5517197 | 95.9093741 | 1.2777867 | 1710017 | [LOC644336](http://www.ncbi.nlm.nih.gov/entrez/query.fcgi?cmd=search&db=gene&term=LOC644336) |  |
| 0.0044913 | 0.071298 | 129.087904 | 101.1664135 | 1.2759957 | 6180168 | [NCF4](http://www.ncbi.nlm.nih.gov/entrez/query.fcgi?cmd=search&db=gene&term=NCF4) | Leukocyte transendothelial migration, immunology |
| 0.0017008 | 0.045404 | 134.3642067 | 105.3431994 | 1.2754901 | 2940050 | [CYB5R2](http://www.ncbi.nlm.nih.gov/entrez/query.fcgi?cmd=search&db=gene&term=CYB5R2) |  |
| 0.0018049 | 0.046475 | 166.697192 | 130.9262037 | 1.2732149 | 6520113 | [C19orf25](http://www.ncbi.nlm.nih.gov/entrez/query.fcgi?cmd=search&db=gene&term=C19orf25) |  |
| 0.0027996 | 0.057448 | 128.7841606 | 101.2362715 | 1.2721148 | 4180500 | [TP53](http://www.ncbi.nlm.nih.gov/entrez/query.fcgi?cmd=search&db=gene&term=TP53) | Apoptotic Signaling in Response to DNA Damage, ATM Signaling Pathway, BTG family proteins and cell cycle regulation, Cell Cycle: G1/S Check Point , Cell Cycle: G2/M Checkpoint, Chaperones modulate interferon Signaling Pathway, CTCF: First Multivalent Nuclear Factor, Double Stranded RNA Induced Gene Expression, Estrogen-responsive protein Efp controls cell cycle and breast tumors growth, Hypoxia and p53 in the Cardiovascular system, Overview of telomerase protein component gene hTert Transcriptional Regulation , p53 Signaling Pathway, RB Tumor Suppressor/Checkpoint Signaling in response to DNA damage, Regulation of cell cycle progression by Plk3, Regulation of transcriptional activity by PML, Role of BRCA1, BRCA2 and ATR in Cancer Susceptibility, Telomeres, Telomerase, Cellular Aging, and Immortality, Tumor Suppressor Arf Inhibits Ribosomal Biogenesis, Amyotrophic lateral sclerosis (ALS), Apoptosis, Cell cycle, Colorectal cancer, Huntington\'s disease, MAPK signaling pathway, Wnt sign ... |
| 0.000216 | 0.020598 | 122.4753095 | 96.4415952 | 1.2699428 | 2940632 | [KCTD14](http://www.ncbi.nlm.nih.gov/entrez/query.fcgi?cmd=search&db=gene&term=KCTD14) |  |
| 0.0029655 | 0.058634 | 140.8386408 | 111.2299542 | 1.2661935 | 3130114 | [ANKRD29](http://www.ncbi.nlm.nih.gov/entrez/query.fcgi?cmd=search&db=gene&term=ANKRD29) |  |
| 0.0018681 | 0.047429 | 155.7133232 | 122.9938338 | 1.2660254 | 5560139 | [RAP1GDS1](http://www.ncbi.nlm.nih.gov/entrez/query.fcgi?cmd=search&db=gene&term=RAP1GDS1) |  |
| 0.0003645 | 0.024601 | 181.8736719 | 144.6240501 | 1.2575617 | 1110494 | [TMEM194](http://www.ncbi.nlm.nih.gov/entrez/query.fcgi?cmd=search&db=gene&term=TMEM194) |  |
| 0.0044952 | 0.071307 | 162.1702059 | 128.9899056 | 1.2572318 | 4880554 | [SALL2](http://www.ncbi.nlm.nih.gov/entrez/query.fcgi?cmd=search&db=gene&term=SALL2) |  |
| 0.00274 | 0.056949 | 169.0614483 | 134.5728505 | 1.256282 | 1410279 | [APOBEC3F](http://www.ncbi.nlm.nih.gov/entrez/query.fcgi?cmd=search&db=gene&term=APOBEC3F) | Atrazine degradation |
| 0.0024751 | 0.05386 | 123.5184011 | 98.7241029 | 1.2511474 | 4070180 | [PRRT3](http://www.ncbi.nlm.nih.gov/entrez/query.fcgi?cmd=search&db=gene&term=PRRT3) |  |
| 0.00051 | 0.02772 | 124.3372583 | 99.9097664 | 1.2444955 | 7320152 |  |  |
| 0.0002135 | 0.020598 | 115.5651082 | 93.1139708 | 1.2411146 | 7000619 | [VAV1](http://www.ncbi.nlm.nih.gov/entrez/query.fcgi?cmd=search&db=gene&term=VAV1) | BCR Signaling Pathway, Fc Epsilon Receptor I Signaling in Mast Cells, Phospholipase C Signaling Pathway, Rac 1 cell motility signaling pathway, Ras-Independent pathway in NK cell-mediated cytotoxicity, T Cell Receptor Signaling Pathway, B cell receptor signaling pathway, Fc epsilon RI signaling pathway, Focal adhesion, Leukocyte transendothelial migration, Natural killer cell mediated cytotoxicity, Regulation of actin cytoskeleton, T cell receptor signaling pathway, tsonc |
| 0.0031412 | 0.060341 | 131.3931442 | 105.8873057 | 1.2408772 | 1300022 | [PABPC5](http://www.ncbi.nlm.nih.gov/entrez/query.fcgi?cmd=search&db=gene&term=PABPC5) |  |
| 0.000135 | 0.018672 | 115.6232482 | 93.2983313 | 1.2392853 | 5340521 |  |  |
| 0.0007838 | 0.032019 | 126.321305 | 102.0119131 | 1.2382995 | 610379 | [APOBEC3F](http://www.ncbi.nlm.nih.gov/entrez/query.fcgi?cmd=search&db=gene&term=APOBEC3F) | Atrazine degradation |
| 0.0032684 | 0.061309 | 138.1491033 | 111.6362053 | 1.2374937 | 450414 | [PPM1H](http://www.ncbi.nlm.nih.gov/entrez/query.fcgi?cmd=search&db=gene&term=PPM1H) |  |
| 0.0005191 | 0.02772 | 131.1660936 | 106.0891633 | 1.236376 | 6660048 | [LOC646064](http://www.ncbi.nlm.nih.gov/entrez/query.fcgi?cmd=search&db=gene&term=LOC646064) |  |
| 0.0008848 | 0.03377 | 122.9640499 | 99.8168362 | 1.2318969 | 3170020 | [DGKA](http://www.ncbi.nlm.nih.gov/entrez/query.fcgi?cmd=search&db=gene&term=DGKA) | Glycerolipid metabolism, Glycerophospholipid metabolism, Phosphatidylinositol signaling system, cell_cycle, cell_signaling, signal_transduction |
| 0.0047813 | 0.072968 | 215.0394512 | 175.5492893 | 1.224952 | 3370121 | [LASS2](http://www.ncbi.nlm.nih.gov/entrez/query.fcgi?cmd=search&db=gene&term=LASS2) |  |
| 0.0035401 | 0.063738 | 122.9929903 | 100.7585832 | 1.2206701 | 6590669 | [RAB24](http://www.ncbi.nlm.nih.gov/entrez/query.fcgi?cmd=search&db=gene&term=RAB24) |  |
| 0.0040938 | 0.068023 | 120.8118856 | 99.5076844 | 1.214096 | 3930408 | [ZNF761](http://www.ncbi.nlm.nih.gov/entrez/query.fcgi?cmd=search&db=gene&term=ZNF761) |  |
| 0.0002529 | 0.022059 | 120.1360519 | 99.1610135 | 1.2115251 | 1010477 | [ADD3](http://www.ncbi.nlm.nih.gov/entrez/query.fcgi?cmd=search&db=gene&term=ADD3) | cell_signaling, metastasis |
| 0.0003737 | 0.024685 | 126.9583325 | 104.8616149 | 1.2107227 | 5860630 | [CCRL1](http://www.ncbi.nlm.nih.gov/entrez/query.fcgi?cmd=search&db=gene&term=CCRL1) |  |
| 0.0003604 | 0.024601 | 111.666401 | 92.2848659 | 1.2100186 | 1190278 |  |  |
| 0.0010823 | 0.03729 | 116.8649777 | 96.8625615 | 1.2065031 | 60102 | [MAPKAP1](http://www.ncbi.nlm.nih.gov/entrez/query.fcgi?cmd=search&db=gene&term=MAPKAP1) |  |
| 0.0009571 | 0.034967 | 134.4408133 | 111.6446275 | 1.2041852 | 4920189 | [C18orf54](http://www.ncbi.nlm.nih.gov/entrez/query.fcgi?cmd=search&db=gene&term=C18orf54) |  |
| 0.0006531 | 0.029547 | 119.4135674 | 99.1716412 | 1.20411 | 7200603 | [PIP5K2A](http://www.ncbi.nlm.nih.gov/entrez/query.fcgi?cmd=search&db=gene&term=PIP5K2A) |  |
| 0.0005902 | 0.028704 | 128.2432915 | 106.6805363 | 1.2021245 | 650612 | [SLC16A5](http://www.ncbi.nlm.nih.gov/entrez/query.fcgi?cmd=search&db=gene&term=SLC16A5) |  |
| 9.04E-05 | 0.017191 | 111.5697537 | 93.0718021 | 1.1987493 | 1450112 | [REPS2](http://www.ncbi.nlm.nih.gov/entrez/query.fcgi?cmd=search&db=gene&term=REPS2) |  |
| 0.0039474 | 0.067074 | 115.7107376 | 96.8946858 | 1.1941908 | 2970397 | [ZNF342](http://www.ncbi.nlm.nih.gov/entrez/query.fcgi?cmd=search&db=gene&term=ZNF342) |  |
| 0.0031578 | 0.060421 | 585.4094216 | 492.6436151 | 1.1883021 | 50114 | [DNAJC9](http://www.ncbi.nlm.nih.gov/entrez/query.fcgi?cmd=search&db=gene&term=DNAJC9) |  |
| 0.004913 | 0.074052 | 119.011363 | 100.1888364 | 1.1878705 | 7380626 | [FLJ20273](http://www.ncbi.nlm.nih.gov/entrez/query.fcgi?cmd=search&db=gene&term=FLJ20273) |  |
| 0.0048699 | 0.073632 | 115.8510366 | 97.707964 | 1.1856867 | 5560546 | [LOC148137](http://www.ncbi.nlm.nih.gov/entrez/query.fcgi?cmd=search&db=gene&term=LOC148137) |  |
| 0.0017369 | 0.045557 | 111.6810359 | 94.3108001 | 1.1841808 | 730482 | [IKZF1](http://www.ncbi.nlm.nih.gov/entrez/query.fcgi?cmd=search&db=gene&term=IKZF1) |  |
| 0.0049301 | 0.074081 | 16874.88328 | 14294.69186 | 1.1805 | 7330630 | [BTF3](http://www.ncbi.nlm.nih.gov/entrez/query.fcgi?cmd=search&db=gene&term=BTF3) |  |
| 0.0028611 | 0.058153 | 105.6725242 | 89.8754863 | 1.1757658 | 2000451 | [PYHIN1](http://www.ncbi.nlm.nih.gov/entrez/query.fcgi?cmd=search&db=gene&term=PYHIN1) |  |
| 0.004653 | 0.072387 | 123.0307308 | 105.2629922 | 1.1687938 | 5360386 | [KIF15](http://www.ncbi.nlm.nih.gov/entrez/query.fcgi?cmd=search&db=gene&term=KIF15) | Role of Ran in mitotic spindle regulation |
| 0.0006255 | 0.029291 | 110.8571638 | 94.9679365 | 1.1673115 | 5560646 | [RBM43](http://www.ncbi.nlm.nih.gov/entrez/query.fcgi?cmd=search&db=gene&term=RBM43) |  |
| 0.0014962 | 0.042622 | 116.0041673 | 99.4172886 | 1.166841 | 7150768 | [NR2F1](http://www.ncbi.nlm.nih.gov/entrez/query.fcgi?cmd=search&db=gene&term=NR2F1) | Mechanism of Gene Regulation by Peroxisome Proliferators via PPARa(alpha) |
| 0.00265 | 0.05581 | 113.0297725 | 96.8790666 | 1.16671 | 6280367 |  |  |
| 0.0025237 | 0.054402 | 131.4570747 | 112.7181574 | 1.1662458 | 2650181 |  |  |
| 0.0015762 | 0.043775 | 138.5164183 | 118.898907 | 1.1649932 | 10209 | [HNRNPA1](http://www.ncbi.nlm.nih.gov/entrez/query.fcgi?cmd=search&db=gene&term=HNRNPA1) |  |
| 0.0039991 | 0.067531 | 110.3552046 | 94.9155084 | 1.1626678 | 1580706 | [FAM70B](http://www.ncbi.nlm.nih.gov/entrez/query.fcgi?cmd=search&db=gene&term=FAM70B) |  |
| 0.0002642 | 0.022504 | 111.1128138 | 95.5987525 | 1.1622831 | 5270148 | [PLEK2](http://www.ncbi.nlm.nih.gov/entrez/query.fcgi?cmd=search&db=gene&term=PLEK2) |  |
| 0.0014158 | 0.041638 | 130.1999324 | 112.1066631 | 1.1613933 | 1170326 | [POLE2](http://www.ncbi.nlm.nih.gov/entrez/query.fcgi?cmd=search&db=gene&term=POLE2) | DNA polymerase, Purine metabolism, Pyrimidine metabolism |
| 0.0017235 | 0.04555 | 105.8777255 | 91.3980507 | 1.1584243 | 7570474 | [FLJ12334](http://www.ncbi.nlm.nih.gov/entrez/query.fcgi?cmd=search&db=gene&term=FLJ12334) |  |
| 0.0047478 | 0.072809 | 108.4156535 | 93.6786516 | 1.1573144 | 1010463 | [BHMT](http://www.ncbi.nlm.nih.gov/entrez/query.fcgi?cmd=search&db=gene&term=BHMT) | Glycine, serine and threonine metabolism, Methionine metabolism, misc |
| 0.0037645 | 0.065289 | 106.5584308 | 92.1749875 | 1.156045 | 6620301 |  |  |
| 0.0008905 | 0.033921 | 26974.56273 | 23341.1678 | 1.1556647 | 6550139 | [LOC441034](http://www.ncbi.nlm.nih.gov/entrez/query.fcgi?cmd=search&db=gene&term=LOC441034) |  |
| 0.0023012 | 0.052299 | 112.0769781 | 97.2533848 | 1.1524224 | 3930064 | [FAM150A](http://www.ncbi.nlm.nih.gov/entrez/query.fcgi?cmd=search&db=gene&term=FAM150A) |  |
| 0.0042291 | 0.069095 | 116.9615827 | 101.5811153 | 1.1514107 | 4860050 | [FYN](http://www.ncbi.nlm.nih.gov/entrez/query.fcgi?cmd=search&db=gene&term=FYN) | Bioactive Peptide Induced Signaling Pathway, Eph Kinases and ephrins support platelet aggregation, Erk and PI-3 Kinase Are Necessary for Collagen Binding in Corneal Epithelia, IL-7 Signal Transduction, Integrin Signaling Pathway, Lck and Fyn tyrosine kinases in initiation of TCR Activation, Reelin Signaling Pathway, T Cell Receptor Signaling Pathway, TSP-1 Induced Apoptosis in Microvascular Endothelial Cell , Adherens junction, Axon guidance, Fc epsilon RI signaling pathway, Focal adhesion, NA, Natural killer cell mediated cytotoxicity, T cell receptor signaling pathway, signal_transduction, tsonc |
| 0.0013049 | 0.039956 | 124.3173901 | 108.1009924 | 1.1500116 | 1580333 | [KIAA1467](http://www.ncbi.nlm.nih.gov/entrez/query.fcgi?cmd=search&db=gene&term=KIAA1467) |  |
| 0.001918 | 0.048107 | 111.4598441 | 97.0433017 | 1.1485578 | 1710376 | [C6orf208](http://www.ncbi.nlm.nih.gov/entrez/query.fcgi?cmd=search&db=gene&term=C6orf208) |  |
| 0.0020642 | 0.049303 | 110.8136895 | 96.5007747 | 1.1483192 | 580209 | [SH3BP2](http://www.ncbi.nlm.nih.gov/entrez/query.fcgi?cmd=search&db=gene&term=SH3BP2) | Natural killer cell mediated cytotoxicity |
| 0.0040347 | 0.067739 | 106.393344 | 92.861091 | 1.1457258 | 2230204 | [OAS2](http://www.ncbi.nlm.nih.gov/entrez/query.fcgi?cmd=search&db=gene&term=OAS2) | immunology, misc |
| 0.0047272 | 0.072685 | 114.0797207 | 99.6871575 | 1.1443773 | 3460537 |  |  |
| 0.0049821 | 0.074372 | 420.6834809 | 367.6482293 | 1.1442554 | 7050626 | [PCNX](http://www.ncbi.nlm.nih.gov/entrez/query.fcgi?cmd=search&db=gene&term=PCNX) |  |
| 0.0006893 | 0.030043 | 111.2727736 | 97.4203152 | 1.1421927 | 1990608 |  |  |
| 0.003419 | 0.062837 | 120.4994545 | 105.6842428 | 1.1401837 | 7050347 | [CTNS](http://www.ncbi.nlm.nih.gov/entrez/query.fcgi?cmd=search&db=gene&term=CTNS) | immunology |
| 0.0020573 | 0.049303 | 107.9396609 | 95.1571864 | 1.1343301 | 5090070 | [MMP14](http://www.ncbi.nlm.nih.gov/entrez/query.fcgi?cmd=search&db=gene&term=MMP14) | Inhibition of Matrix Metalloproteinases, GnRH signaling pathway, immunology |
| 0.0018492 | 0.047185 | 105.4469467 | 93.0259627 | 1.1335217 | 5690189 |  |  |
| 0.0028018 | 0.057448 | 108.5515201 | 96.1540992 | 1.1289328 | 4640561 |  |  |
| 0.0048582 | 0.073632 | 103.2160885 | 92.1418926 | 1.1201863 | 7160634 | [C12orf50](http://www.ncbi.nlm.nih.gov/entrez/query.fcgi?cmd=search&db=gene&term=C12orf50) |  |
| 0.0031831 | 0.060634 | 120.9993351 | 109.0707324 | 1.1093658 | 4060068 | [PDIA4](http://www.ncbi.nlm.nih.gov/entrez/query.fcgi?cmd=search&db=gene&term=PDIA4) |  |
| 0.0023549 | 0.052574 | 106.814758 | 96.2947601 | 1.1092479 | 7610711 | [OR5P2](http://www.ncbi.nlm.nih.gov/entrez/query.fcgi?cmd=search&db=gene&term=OR5P2) |  |
| 0.0030452 | 0.059321 | 107.915823 | 97.406044 | 1.1078966 | 1850463 | [LOC732371](http://www.ncbi.nlm.nih.gov/entrez/query.fcgi?cmd=search&db=gene&term=LOC732371) |  |
| 0.0003899 | 0.025241 | 106.4624832 | 97.0993442 | 1.0964284 | 5270348 |  |  |
| 0.0027065 | 0.056586 | 100.2683504 | 92.0780211 | 1.0889499 | 4280039 | [CBY1](http://www.ncbi.nlm.nih.gov/entrez/query.fcgi?cmd=search&db=gene&term=CBY1) |  |
| 0.0025286 | 0.054402 | 106.9319176 | 98.3269388 | 1.0875139 | 6250021 |  |  |
| 0.0034902 | 0.06355 | 100.7691656 | 107.130225 | 0.9406231 | 3940619 | [MGC40170](http://www.ncbi.nlm.nih.gov/entrez/query.fcgi?cmd=search&db=gene&term=MGC40170) |  |
| 0.0003144 | 0.023475 | 95.2252633 | 104.5521199 | 0.9107923 | 4060349 | [PBEF1](http://www.ncbi.nlm.nih.gov/entrez/query.fcgi?cmd=search&db=gene&term=PBEF1) |  |
| 0.0025616 | 0.054816 | 104.9107464 | 116.00688 | 0.9043493 | 6420338 |  |  |
| 0.0019669 | 0.048685 | 97.5846858 | 108.1959491 | 0.9019255 | 2760154 |  |  |
| 0.0015807 | 0.043775 | 110.7685572 | 123.7509042 | 0.8950929 | 6860471 | [ZNF516](http://www.ncbi.nlm.nih.gov/entrez/query.fcgi?cmd=search&db=gene&term=ZNF516) |  |
| 0.0043414 | 0.070014 | 99.2268056 | 111.5312804 | 0.8896769 | 5310400 | [CRLF2](http://www.ncbi.nlm.nih.gov/entrez/query.fcgi?cmd=search&db=gene&term=CRLF2) | Cytokine-cytokine receptor interaction, Jak-STAT signaling pathway |
| 0.0032669 | 0.061309 | 93.8045135 | 105.6270856 | 0.8880725 | 3360397 |  |  |
| 0.0008381 | 0.033438 | 93.5901019 | 106.3882628 | 0.8797033 | 4250731 | [MYCL1](http://www.ncbi.nlm.nih.gov/entrez/query.fcgi?cmd=search&db=gene&term=MYCL1) | immunology, tsonc |
| 0.0020415 | 0.049303 | 88.131953 | 100.2037721 | 0.8795273 | 5360746 |  |  |
| 0.0048 | 0.073108 | 96.0384395 | 109.9067897 | 0.8738172 | 610520 |  |  |
| 0.0036027 | 0.064157 | 101.9866876 | 117.1619927 | 0.8704759 | 3450437 |  |  |
| 0.004465 | 0.071061 | 100.4365304 | 116.8277868 | 0.8596973 | 3890133 | [LOC643719](http://www.ncbi.nlm.nih.gov/entrez/query.fcgi?cmd=search&db=gene&term=LOC643719) |  |
| 0.002483 | 0.05386 | 173.4776248 | 202.3048921 | 0.8575058 | 4760300 | [ZMYND8](http://www.ncbi.nlm.nih.gov/entrez/query.fcgi?cmd=search&db=gene&term=ZMYND8) |  |
| 0.0036623 | 0.064645 | 105.8692484 | 123.7068709 | 0.8558073 | 4860661 | [HERC2P2](http://www.ncbi.nlm.nih.gov/entrez/query.fcgi?cmd=search&db=gene&term=HERC2P2) |  |
| 0.0023651 | 0.052605 | 104.5709289 | 122.1964346 | 0.8557609 | 1570113 | [GPS1](http://www.ncbi.nlm.nih.gov/entrez/query.fcgi?cmd=search&db=gene&term=GPS1) |  |
| 0.0026536 | 0.05581 | 96.6446738 | 112.9842885 | 0.8553815 | 7550176 | [PQLC2](http://www.ncbi.nlm.nih.gov/entrez/query.fcgi?cmd=search&db=gene&term=PQLC2) |  |
| 0.0023506 | 0.052551 | 95.7991618 | 112.0106806 | 0.8552681 | 3800239 |  |  |
| 0.0049897 | 0.074436 | 93.3402711 | 109.4840865 | 0.8525465 | 1260739 |  |  |
| 0.00204 | 0.049303 | 97.7928056 | 114.9622189 | 0.8506517 | 2850736 |  |  |
| 0.0023823 | 0.05282 | 98.0319999 | 115.6112395 | 0.8479452 | 4860601 |  |  |
| 0.0047211 | 0.072636 | 130.4594458 | 154.063858 | 0.8467881 | 6660440 |  |  |
| 0.0047204 | 0.072636 | 97.4575044 | 115.2866011 | 0.8453498 | 60072 | [NRN1L](http://www.ncbi.nlm.nih.gov/entrez/query.fcgi?cmd=search&db=gene&term=NRN1L) |  |
| 0.0031537 | 0.060421 | 103.5745398 | 122.6094234 | 0.8447519 | 6350725 | [LOC641959](http://www.ncbi.nlm.nih.gov/entrez/query.fcgi?cmd=search&db=gene&term=LOC641959) |  |
| 0.0012554 | 0.039505 | 102.4262129 | 121.3273274 | 0.8442139 | 3940072 | [LOC200493](http://www.ncbi.nlm.nih.gov/entrez/query.fcgi?cmd=search&db=gene&term=LOC200493) |  |
| 0.0033354 | 0.061973 | 91.4805427 | 108.4326577 | 0.8436623 | 2760324 |  |  |
| 0.0030799 | 0.059717 | 93.3297131 | 110.7110104 | 0.843003 | 3180575 |  |  |
| 4.16E-05 | 0.014558 | 165.5701913 | 197.1176949 | 0.839956 | 5870148 | [CHD1L](http://www.ncbi.nlm.nih.gov/entrez/query.fcgi?cmd=search&db=gene&term=CHD1L) |  |
| 0.0047077 | 0.072636 | 93.577281 | 111.917647 | 0.8361262 | 7560386 |  |  |
| 0.0036902 | 0.064645 | 450.2729558 | 539.1254466 | 0.8351914 | 6650201 | [CCDC97](http://www.ncbi.nlm.nih.gov/entrez/query.fcgi?cmd=search&db=gene&term=CCDC97) |  |
| 0.0003182 | 0.023516 | 94.1237778 | 112.9011927 | 0.8336828 | 6290019 |  |  |
| 0.0049443 | 0.074081 | 92.3582062 | 111.0392416 | 0.8317619 | 1410634 |  |  |
| 0.0024279 | 0.053327 | 106.5969767 | 128.8166422 | 0.8275094 | 6760487 | [CXXC6](http://www.ncbi.nlm.nih.gov/entrez/query.fcgi?cmd=search&db=gene&term=CXXC6) |  |
| 0.0046153 | 0.072259 | 96.3256135 | 116.786159 | 0.8248033 | 6580278 | [FLJ45994](http://www.ncbi.nlm.nih.gov/entrez/query.fcgi?cmd=search&db=gene&term=FLJ45994) |  |
| 0.0001248 | 0.018034 | 116.0411152 | 141.0804646 | 0.8225172 | 2760692 | [EXOC7](http://www.ncbi.nlm.nih.gov/entrez/query.fcgi?cmd=search&db=gene&term=EXOC7) | Insulin signaling pathway |
| 0.004103 | 0.068069 | 97.3654552 | 118.4954301 | 0.8216811 | 3140328 | [TUBB4](http://www.ncbi.nlm.nih.gov/entrez/query.fcgi?cmd=search&db=gene&term=TUBB4) | Gap junction |
| 0.0020036 | 0.049013 | 109.9424315 | 133.9870979 | 0.8205449 | 6550576 |  |  |
| 0.0036222 | 0.064347 | 91.3582745 | 111.6925717 | 0.8179441 | 2690575 | [PAX6](http://www.ncbi.nlm.nih.gov/entrez/query.fcgi?cmd=search&db=gene&term=PAX6) | Maturity onset diabetes of the young, development |
| 0.0038449 | 0.065994 | 104.9756606 | 128.5560414 | 0.8165751 | 3130440 | [GPR113](http://www.ncbi.nlm.nih.gov/entrez/query.fcgi?cmd=search&db=gene&term=GPR113) |  |
| 0.0003397 | 0.024302 | 146.9778096 | 181.5522938 | 0.8095618 | 5560465 | [NOL5A](http://www.ncbi.nlm.nih.gov/entrez/query.fcgi?cmd=search&db=gene&term=NOL5A) |  |
| 0.0037154 | 0.06498 | 116.3007242 | 143.9355805 | 0.8080054 | 5340288 | [UBOX5](http://www.ncbi.nlm.nih.gov/entrez/query.fcgi?cmd=search&db=gene&term=UBOX5) |  |
| 0.0025848 | 0.055088 | 187.7519483 | 232.6161712 | 0.807132 | 6450088 |  |  |
| 0.0024697 | 0.053853 | 109.6331949 | 135.8905504 | 0.8067757 | 2760711 | [PZP](http://www.ncbi.nlm.nih.gov/entrez/query.fcgi?cmd=search&db=gene&term=PZP) |  |
| 0.0023879 | 0.052863 | 111.6466386 | 138.5183755 | 0.806006 | 150725 | [MYOCD](http://www.ncbi.nlm.nih.gov/entrez/query.fcgi?cmd=search&db=gene&term=MYOCD) |  |
| 0.0001649 | 0.019753 | 89.2710457 | 110.8153834 | 0.8055835 | 5910048 | [EPS8L3](http://www.ncbi.nlm.nih.gov/entrez/query.fcgi?cmd=search&db=gene&term=EPS8L3) |  |
| 0.0048208 | 0.073309 | 126.7903018 | 157.4942117 | 0.8050474 | 4640762 | [LOC642502](http://www.ncbi.nlm.nih.gov/entrez/query.fcgi?cmd=search&db=gene&term=LOC642502) |  |
| 0.0049571 | 0.074101 | 124.4384875 | 155.4878078 | 0.8003103 | 830086 | [HYPK](http://www.ncbi.nlm.nih.gov/entrez/query.fcgi?cmd=search&db=gene&term=HYPK) |  |
| 0.0007551 | 0.031421 | 132.2123808 | 165.2034821 | 0.8003002 | 60408 | [NFRKB](http://www.ncbi.nlm.nih.gov/entrez/query.fcgi?cmd=search&db=gene&term=NFRKB) | immunology |
| 0.0039489 | 0.067074 | 206.6964459 | 258.2830498 | 0.800271 | 4010528 | [BAP1](http://www.ncbi.nlm.nih.gov/entrez/query.fcgi?cmd=search&db=gene&term=BAP1) |  |
| 0.0042756 | 0.069314 | 117.5755206 | 147.1280679 | 0.7991373 | 7320687 | [POU2F1](http://www.ncbi.nlm.nih.gov/entrez/query.fcgi?cmd=search&db=gene&term=POU2F1) | gene_regulation, misc, transcription |
| 0.0011253 | 0.037826 | 120.2860997 | 150.9676142 | 0.7967676 | 6060736 | [FMO6P](http://www.ncbi.nlm.nih.gov/entrez/query.fcgi?cmd=search&db=gene&term=FMO6P) |  |
| 0.0007524 | 0.031354 | 116.6760852 | 146.4595147 | 0.7966439 | 5290537 | [NUPL1](http://www.ncbi.nlm.nih.gov/entrez/query.fcgi?cmd=search&db=gene&term=NUPL1) |  |
| 0.0012509 | 0.039505 | 129.690501 | 164.0007913 | 0.7907919 | 2970315 | [RNPC3](http://www.ncbi.nlm.nih.gov/entrez/query.fcgi?cmd=search&db=gene&term=RNPC3) |  |
| 0.0045842 | 0.072089 | 212.0023412 | 268.620164 | 0.7892272 | 1170156 | [EARS2](http://www.ncbi.nlm.nih.gov/entrez/query.fcgi?cmd=search&db=gene&term=EARS2) |  |
| 0.0005198 | 0.02772 | 117.4381597 | 149.1473121 | 0.7873971 | 60689 | [AAAS](http://www.ncbi.nlm.nih.gov/entrez/query.fcgi?cmd=search&db=gene&term=AAAS) |  |
| 0.00212 | 0.050021 | 185.0018226 | 235.315169 | 0.7861874 | 4590646 | [C8orf41](http://www.ncbi.nlm.nih.gov/entrez/query.fcgi?cmd=search&db=gene&term=C8orf41) |  |
| 0.0014055 | 0.041571 | 136.6776427 | 174.015394 | 0.7854342 | 2850553 | [FLJ35801](http://www.ncbi.nlm.nih.gov/entrez/query.fcgi?cmd=search&db=gene&term=FLJ35801) |  |
| 0.0006394 | 0.029369 | 15189.02941 | 19341.94756 | 0.7852896 | 3140019 | [LOC645317](http://www.ncbi.nlm.nih.gov/entrez/query.fcgi?cmd=search&db=gene&term=LOC645317) |  |
| 0.0012073 | 0.038833 | 1719.43437 | 2197.760651 | 0.7823574 | 5550296 | [OSBP](http://www.ncbi.nlm.nih.gov/entrez/query.fcgi?cmd=search&db=gene&term=OSBP) |  |
| 0.0011269 | 0.037826 | 94.6084496 | 121.1723462 | 0.7807759 | 4760390 | [CHST9](http://www.ncbi.nlm.nih.gov/entrez/query.fcgi?cmd=search&db=gene&term=CHST9) |  |
| 0.0046271 | 0.072259 | 119.9819381 | 153.9122774 | 0.7795475 | 3460193 | [H2AFV](http://www.ncbi.nlm.nih.gov/entrez/query.fcgi?cmd=search&db=gene&term=H2AFV) |  |
| 0.0022169 | 0.051103 | 118.0912342 | 151.7705103 | 0.7780908 | 4830717 | [SART3](http://www.ncbi.nlm.nih.gov/entrez/query.fcgi?cmd=search&db=gene&term=SART3) |  |
| 0.0028836 | 0.058288 | 107.9530491 | 138.7617304 | 0.7779742 | 6250184 | [FAM78A](http://www.ncbi.nlm.nih.gov/entrez/query.fcgi?cmd=search&db=gene&term=FAM78A) |  |
| 0.004747 | 0.072809 | 116.926931 | 150.3669231 | 0.7776107 | 3360743 | [NUDT6](http://www.ncbi.nlm.nih.gov/entrez/query.fcgi?cmd=search&db=gene&term=NUDT6) |  |
| 0.0004642 | 0.026685 | 100.0600882 | 129.2220193 | 0.7743269 | 3890102 | [NR4A1](http://www.ncbi.nlm.nih.gov/entrez/query.fcgi?cmd=search&db=gene&term=NR4A1) | MAPK signaling pathway |
| 0.0004181 | 0.025615 | 162.9352586 | 210.6529066 | 0.7734774 | 5720070 | [THAP6](http://www.ncbi.nlm.nih.gov/entrez/query.fcgi?cmd=search&db=gene&term=THAP6) |  |
| 0.0013219 | 0.040294 | 124.5841289 | 161.2237986 | 0.7727403 | 6110753 | [NBN](http://www.ncbi.nlm.nih.gov/entrez/query.fcgi?cmd=search&db=gene&term=NBN) | ATM Signaling Pathway, Role of BRCA1, BRCA2 and ATR in Cancer Susceptibility |
| 0.0012986 | 0.039913 | 96.3562061 | 124.8438164 | 0.771814 | 4830008 | [FAM155B](http://www.ncbi.nlm.nih.gov/entrez/query.fcgi?cmd=search&db=gene&term=FAM155B) |  |
| 0.0040688 | 0.06788 | 254.7340558 | 330.2553398 | 0.7713246 | 5310259 | [COG1](http://www.ncbi.nlm.nih.gov/entrez/query.fcgi?cmd=search&db=gene&term=COG1) |  |
| 0.0006477 | 0.029394 | 105.8012638 | 137.2826782 | 0.7706818 | 5490255 | [MYH14](http://www.ncbi.nlm.nih.gov/entrez/query.fcgi?cmd=search&db=gene&term=MYH14) | Regulation of actin cytoskeleton, Tight junction |
| 0.0009072 | 0.034003 | 413.0141112 | 536.2388481 | 0.7702055 | 130544 | [KIAA1160](http://www.ncbi.nlm.nih.gov/entrez/query.fcgi?cmd=search&db=gene&term=KIAA1160) |  |
| 0.0005452 | 0.028008 | 127.043838 | 165.4955509 | 0.7676571 | 2710711 | [TOM1](http://www.ncbi.nlm.nih.gov/entrez/query.fcgi?cmd=search&db=gene&term=TOM1) |  |
| 0.0043217 | 0.069735 | 434.342798 | 566.6220194 | 0.7665477 | 1050132 | [ZNF518B](http://www.ncbi.nlm.nih.gov/entrez/query.fcgi?cmd=search&db=gene&term=ZNF518B) |  |
| 0.0032288 | 0.061015 | 252.3181796 | 329.162541 | 0.7665458 | 6280044 | [CDC2L5](http://www.ncbi.nlm.nih.gov/entrez/query.fcgi?cmd=search&db=gene&term=CDC2L5) |  |
| 0.0023967 | 0.052977 | 109.7381023 | 143.8932345 | 0.7626356 | 7160332 |  |  |
| 0.0028551 | 0.058112 | 465.9690116 | 612.4050062 | 0.7608837 | 5820333 | [RPUSD2](http://www.ncbi.nlm.nih.gov/entrez/query.fcgi?cmd=search&db=gene&term=RPUSD2) |  |
| 0.0013956 | 0.041447 | 102.7984579 | 136.0881822 | 0.7553812 | 6130474 | [COL4A3](http://www.ncbi.nlm.nih.gov/entrez/query.fcgi?cmd=search&db=gene&term=COL4A3) | immunology |
| 0.0042051 | 0.068875 | 126.8840394 | 168.2746659 | 0.7540294 | 5820458 | [CCDC140](http://www.ncbi.nlm.nih.gov/entrez/query.fcgi?cmd=search&db=gene&term=CCDC140) |  |
| 0.0038467 | 0.065994 | 140.7446998 | 186.9530907 | 0.7528343 | 1300075 | [PCTP](http://www.ncbi.nlm.nih.gov/entrez/query.fcgi?cmd=search&db=gene&term=PCTP) |  |
| 0.0008728 | 0.033692 | 318.681955 | 423.797219 | 0.751968 | 770537 | [ZNF407](http://www.ncbi.nlm.nih.gov/entrez/query.fcgi?cmd=search&db=gene&term=ZNF407) |  |
| 0.0012423 | 0.039435 | 752.5080533 | 1000.963117 | 0.751784 | 1570433 | [GAPVD1](http://www.ncbi.nlm.nih.gov/entrez/query.fcgi?cmd=search&db=gene&term=GAPVD1) |  |
| 0.0026338 | 0.055555 | 244.9147358 | 325.8020772 | 0.7517286 | 2490598 | [ANXA6](http://www.ncbi.nlm.nih.gov/entrez/query.fcgi?cmd=search&db=gene&term=ANXA6) |  |
| 0.0017379 | 0.045557 | 2127.023864 | 2844.323414 | 0.7478136 | 6370091 | [ATP6V1G1](http://www.ncbi.nlm.nih.gov/entrez/query.fcgi?cmd=search&db=gene&term=ATP6V1G1) | ATP synthesis, Epithelial cell signaling in Helicobacter pylori infection, Oxidative phosphorylation |
| 0.0047062 | 0.072636 | 163.7378358 | 219.8020462 | 0.7449332 | 4860142 | [UTP18](http://www.ncbi.nlm.nih.gov/entrez/query.fcgi?cmd=search&db=gene&term=UTP18) |  |
| 0.0039758 | 0.067327 | 108.2409634 | 145.3401778 | 0.7447422 | 6380414 | [C10orf59](http://www.ncbi.nlm.nih.gov/entrez/query.fcgi?cmd=search&db=gene&term=C10orf59) |  |
| 0.0024278 | 0.053327 | 100.3195407 | 134.72354 | 0.7446326 | 6130451 | [ACCN3](http://www.ncbi.nlm.nih.gov/entrez/query.fcgi?cmd=search&db=gene&term=ACCN3) |  |
| 0.0030155 | 0.059206 | 129.3978702 | 173.8181278 | 0.744444 | 5820187 | [SCCPDH](http://www.ncbi.nlm.nih.gov/entrez/query.fcgi?cmd=search&db=gene&term=SCCPDH) |  |
| 0.0009653 | 0.03509 | 145.0984857 | 194.9859997 | 0.7441482 | 3990202 | [HMG1L1](http://www.ncbi.nlm.nih.gov/entrez/query.fcgi?cmd=search&db=gene&term=HMG1L1) |  |
| 0.0040674 | 0.06788 | 114.8348946 | 154.4190202 | 0.7436577 | 130440 | [LOC643446](http://www.ncbi.nlm.nih.gov/entrez/query.fcgi?cmd=search&db=gene&term=LOC643446) |  |
| 0.00018 | 0.019879 | 120.172171 | 161.610609 | 0.7435909 | 1940471 | [LOC389257](http://www.ncbi.nlm.nih.gov/entrez/query.fcgi?cmd=search&db=gene&term=LOC389257) |  |
| 0.0006257 | 0.029291 | 250.2653657 | 336.716032 | 0.7432535 | 5900181 | [SH2B1](http://www.ncbi.nlm.nih.gov/entrez/query.fcgi?cmd=search&db=gene&term=SH2B1) |  |
| 0.0004806 | 0.02718 | 109.0185447 | 146.8630989 | 0.7423141 | 7380300 | [LOC642468](http://www.ncbi.nlm.nih.gov/entrez/query.fcgi?cmd=search&db=gene&term=LOC642468) |  |
| 0.0046011 | 0.072259 | 721.7491528 | 973.7003634 | 0.7412436 | 70019 | [SFRS14](http://www.ncbi.nlm.nih.gov/entrez/query.fcgi?cmd=search&db=gene&term=SFRS14) |  |
| 0.0049553 | 0.074101 | 931.8744188 | 1258.043901 | 0.7407328 | 4590537 | [ERAL1](http://www.ncbi.nlm.nih.gov/entrez/query.fcgi?cmd=search&db=gene&term=ERAL1) |  |
| 0.0032367 | 0.061081 | 181.2915735 | 244.78329 | 0.7406207 | 1990433 | [RMI1](http://www.ncbi.nlm.nih.gov/entrez/query.fcgi?cmd=search&db=gene&term=RMI1) |  |
| 0.0002675 | 0.022554 | 150.9556393 | 203.8634984 | 0.7404741 | 5670470 | [KIAA0564](http://www.ncbi.nlm.nih.gov/entrez/query.fcgi?cmd=search&db=gene&term=KIAA0564) |  |
| 0.0029151 | 0.058389 | 190.4508256 | 257.3005744 | 0.7401881 | 4760392 | [CACNA1B](http://www.ncbi.nlm.nih.gov/entrez/query.fcgi?cmd=search&db=gene&term=CACNA1B) | Calcium signaling pathway, MAPK signaling pathway, Taste transduction, Type II diabetes mellitus |
| 0.0002848 | 0.022725 | 123.1795693 | 166.8943197 | 0.7380693 | 4560279 | [LOC649754](http://www.ncbi.nlm.nih.gov/entrez/query.fcgi?cmd=search&db=gene&term=LOC649754) |  |
| 0.0033635 | 0.062225 | 121.1976102 | 164.233049 | 0.7379612 | 3060538 | [PRKCABP](http://www.ncbi.nlm.nih.gov/entrez/query.fcgi?cmd=search&db=gene&term=PRKCABP) |  |
| 0.0036852 | 0.064645 | 124.9840042 | 170.1586076 | 0.7345147 | 50360 | [C11orf31](http://www.ncbi.nlm.nih.gov/entrez/query.fcgi?cmd=search&db=gene&term=C11orf31) |  |
| 0.0046361 | 0.072259 | 355.2079628 | 484.6737258 | 0.7328806 | 6020661 | [C12orf26](http://www.ncbi.nlm.nih.gov/entrez/query.fcgi?cmd=search&db=gene&term=C12orf26) |  |
| 0.0031054 | 0.059971 | 220.4477674 | 300.9542924 | 0.7324958 | 6450538 | [TCERG1](http://www.ncbi.nlm.nih.gov/entrez/query.fcgi?cmd=search&db=gene&term=TCERG1) |  |
| 0.0016025 | 0.044118 | 116.7056383 | 159.5006986 | 0.7316936 | 4050440 |  |  |
| 0.0032986 | 0.061599 | 1044.930487 | 1429.466893 | 0.7309931 | 4210754 | [SERBP1](http://www.ncbi.nlm.nih.gov/entrez/query.fcgi?cmd=search&db=gene&term=SERBP1) |  |
| 0.0037809 | 0.065289 | 186.6724595 | 255.5191329 | 0.7305616 | 620292 | [ZNF594](http://www.ncbi.nlm.nih.gov/entrez/query.fcgi?cmd=search&db=gene&term=ZNF594) |  |
| 0.0037602 | 0.065289 | 108.8423345 | 149.0487405 | 0.7302466 | 270035 | [TMEM120B](http://www.ncbi.nlm.nih.gov/entrez/query.fcgi?cmd=search&db=gene&term=TMEM120B) |  |
| 5.55E-05 | 0.0155 | 107.8702933 | 148.0958322 | 0.7283817 | 4070279 | [SMPD4](http://www.ncbi.nlm.nih.gov/entrez/query.fcgi?cmd=search&db=gene&term=SMPD4) |  |
| 0.0032745 | 0.06132 | 1079.449893 | 1485.924295 | 0.7264501 | 7320328 | [NSFL1C](http://www.ncbi.nlm.nih.gov/entrez/query.fcgi?cmd=search&db=gene&term=NSFL1C) |  |
| 0.0008606 | 0.033634 | 470.7075369 | 649.2365205 | 0.725017 | 2760424 | [LOC644935](http://www.ncbi.nlm.nih.gov/entrez/query.fcgi?cmd=search&db=gene&term=LOC644935) |  |
| 3.98E-05 | 0.014558 | 303.6776199 | 419.1871887 | 0.7244439 | 2030446 | [SNAPC3](http://www.ncbi.nlm.nih.gov/entrez/query.fcgi?cmd=search&db=gene&term=SNAPC3) |  |
| 0.0019979 | 0.049006 | 251.8731348 | 348.0112333 | 0.72375 | 150048 | [SEC24B](http://www.ncbi.nlm.nih.gov/entrez/query.fcgi?cmd=search&db=gene&term=SEC24B) |  |
| 0.0028372 | 0.05791 | 109.0051684 | 150.9423806 | 0.7221641 | 160491 | [ZNF268](http://www.ncbi.nlm.nih.gov/entrez/query.fcgi?cmd=search&db=gene&term=ZNF268) |  |
| 0.0010612 | 0.037012 | 101.6892657 | 140.847567 | 0.721981 | 1110246 | [ZNF768](http://www.ncbi.nlm.nih.gov/entrez/query.fcgi?cmd=search&db=gene&term=ZNF768) |  |
| 0.0021427 | 0.050338 | 150.9549782 | 209.2458998 | 0.7214238 | 6590324 | [PTDSS2](http://www.ncbi.nlm.nih.gov/entrez/query.fcgi?cmd=search&db=gene&term=PTDSS2) |  |
| 0.0021949 | 0.050838 | 226.3576936 | 314.7175431 | 0.7192408 | 7150189 | [PHF17](http://www.ncbi.nlm.nih.gov/entrez/query.fcgi?cmd=search&db=gene&term=PHF17) |  |
| 0.0017081 | 0.045474 | 152.5805762 | 212.3920817 | 0.7183911 | 1010367 | [CSTF3](http://www.ncbi.nlm.nih.gov/entrez/query.fcgi?cmd=search&db=gene&term=CSTF3) | Polyadenylation of mRNA, gene_regulation, transcription |
| 0.0035275 | 0.063701 | 116.0355061 | 161.6260468 | 0.7179258 | 5550445 | [FLJ22795](http://www.ncbi.nlm.nih.gov/entrez/query.fcgi?cmd=search&db=gene&term=FLJ22795) |  |
| 0.0004471 | 0.026228 | 146.1275011 | 203.6185655 | 0.7176531 | 2140273 | [PRIM1](http://www.ncbi.nlm.nih.gov/entrez/query.fcgi?cmd=search&db=gene&term=PRIM1) | DNA polymerase, Purine metabolism, Pyrimidine metabolism, DNA_adducts, DNA_damage |
| 0.0011239 | 0.037826 | 232.9785765 | 324.6451917 | 0.7176406 | 6510661 | [SS18L1](http://www.ncbi.nlm.nih.gov/entrez/query.fcgi?cmd=search&db=gene&term=SS18L1) |  |
| 0.004707 | 0.072636 | 203.5494268 | 283.6928008 | 0.7174994 | 5570246 | [DISP1](http://www.ncbi.nlm.nih.gov/entrez/query.fcgi?cmd=search&db=gene&term=DISP1) |  |
| 0.0032407 | 0.061081 | 436.2049246 | 609.5504977 | 0.7156174 | 3290382 | [TSC1](http://www.ncbi.nlm.nih.gov/entrez/query.fcgi?cmd=search&db=gene&term=TSC1) | mTOR Signaling Pathway, Insulin signaling pathway, mTOR signaling pathway, misc |
| 0.0011242 | 0.037826 | 110.275965 | 154.1305662 | 0.7154711 | 7160671 | [PRPF6](http://www.ncbi.nlm.nih.gov/entrez/query.fcgi?cmd=search&db=gene&term=PRPF6) |  |
| 0.000585 | 0.028704 | 112.4819544 | 157.3987203 | 0.7146307 | 630327 | [RET](http://www.ncbi.nlm.nih.gov/entrez/query.fcgi?cmd=search&db=gene&term=RET) | cell_cycle, cell_signaling, development, signal_transduction, tsonc |
| 0.0006017 | 0.028791 | 133.6557126 | 187.0853711 | 0.7144103 | 4060736 |  |  |
| 0.0014522 | 0.042053 | 110.523699 | 154.9686579 | 0.7132003 | 1170382 | [ASB4](http://www.ncbi.nlm.nih.gov/entrez/query.fcgi?cmd=search&db=gene&term=ASB4) |  |
| 0.0035168 | 0.063698 | 801.2446956 | 1124.051667 | 0.7128184 | 7570465 | [KIAA1191](http://www.ncbi.nlm.nih.gov/entrez/query.fcgi?cmd=search&db=gene&term=KIAA1191) |  |
| 0.0047833 | 0.072968 | 590.7807765 | 830.1587587 | 0.7116479 | 6590441 | [TRIP12](http://www.ncbi.nlm.nih.gov/entrez/query.fcgi?cmd=search&db=gene&term=TRIP12) |  |
| 0.0017807 | 0.046294 | 154.7098037 | 217.4339821 | 0.7115254 | 6960730 | [NUBP2](http://www.ncbi.nlm.nih.gov/entrez/query.fcgi?cmd=search&db=gene&term=NUBP2) |  |
| 0.0021317 | 0.050143 | 116.9090487 | 164.7342655 | 0.7096826 | 990594 | [TRAPPC6B](http://www.ncbi.nlm.nih.gov/entrez/query.fcgi?cmd=search&db=gene&term=TRAPPC6B) |  |
| 0.0039875 | 0.067453 | 158.5618387 | 223.7161228 | 0.7087636 | 6370477 | [HDHD2](http://www.ncbi.nlm.nih.gov/entrez/query.fcgi?cmd=search&db=gene&term=HDHD2) |  |
| 0.0040335 | 0.067739 | 136.0608261 | 192.0537877 | 0.7084517 | 5910187 | [SDHALP1](http://www.ncbi.nlm.nih.gov/entrez/query.fcgi?cmd=search&db=gene&term=SDHALP1) |  |
| 0.0046965 | 0.072636 | 94.3941972 | 133.7147775 | 0.7059369 | 2650519 | [LRRC2](http://www.ncbi.nlm.nih.gov/entrez/query.fcgi?cmd=search&db=gene&term=LRRC2) |  |
| 0.0002259 | 0.020993 | 204.1304835 | 289.1884986 | 0.7058735 | 4150242 | [VPS13D](http://www.ncbi.nlm.nih.gov/entrez/query.fcgi?cmd=search&db=gene&term=VPS13D) |  |
| 0.0040729 | 0.067909 | 394.5509446 | 559.0898857 | 0.7057022 | 5820240 | [FNTB](http://www.ncbi.nlm.nih.gov/entrez/query.fcgi?cmd=search&db=gene&term=FNTB) | N-Glycan biosynthesis |
| 0.0005827 | 0.028704 | 503.9859164 | 714.7304937 | 0.7051412 | 5960736 | [C20orf72](http://www.ncbi.nlm.nih.gov/entrez/query.fcgi?cmd=search&db=gene&term=C20orf72) |  |
| 0.003234 | 0.061074 | 846.8746298 | 1202.271986 | 0.7043952 | 2350209 | [YY1AP1](http://www.ncbi.nlm.nih.gov/entrez/query.fcgi?cmd=search&db=gene&term=YY1AP1) |  |
| 0.0022735 | 0.051913 | 109.0516276 | 154.8480051 | 0.7042495 | 7510181 | [LOC648500](http://www.ncbi.nlm.nih.gov/entrez/query.fcgi?cmd=search&db=gene&term=LOC648500) |  |
| 0.0022152 | 0.051103 | 158.5588253 | 225.2895763 | 0.7038001 | 4760072 | [RORA](http://www.ncbi.nlm.nih.gov/entrez/query.fcgi?cmd=search&db=gene&term=RORA) |  |
| 0.0033769 | 0.062354 | 145.4109544 | 206.6474855 | 0.7036667 | 7150333 | [CTDSP1](http://www.ncbi.nlm.nih.gov/entrez/query.fcgi?cmd=search&db=gene&term=CTDSP1) |  |
| 0.0018387 | 0.04697 | 117.3222461 | 166.7943829 | 0.7033945 | 1340703 | [LUC7L2](http://www.ncbi.nlm.nih.gov/entrez/query.fcgi?cmd=search&db=gene&term=LUC7L2) |  |
| 0.0036898 | 0.064645 | 1440.63186 | 2050.171974 | 0.7026883 | 1010682 | [ARIH2](http://www.ncbi.nlm.nih.gov/entrez/query.fcgi?cmd=search&db=gene&term=ARIH2) |  |
| 0.0017909 | 0.046294 | 539.1665211 | 767.4118849 | 0.7025778 | 5890368 | [LOC653377](http://www.ncbi.nlm.nih.gov/entrez/query.fcgi?cmd=search&db=gene&term=LOC653377) |  |
| 0.0040656 | 0.06788 | 256.9274233 | 365.8386684 | 0.7022971 | 2940367 | [TBC1D7](http://www.ncbi.nlm.nih.gov/entrez/query.fcgi?cmd=search&db=gene&term=TBC1D7) |  |
| 0.0014711 | 0.042401 | 265.2354969 | 378.2537895 | 0.7012104 | 7100131 | [BCS1L](http://www.ncbi.nlm.nih.gov/entrez/query.fcgi?cmd=search&db=gene&term=BCS1L) |  |
| 0.0019255 | 0.048229 | 194.5305416 | 277.8704958 | 0.7000763 | 540382 | [RSAD1](http://www.ncbi.nlm.nih.gov/entrez/query.fcgi?cmd=search&db=gene&term=RSAD1) |  |
| 0.0040922 | 0.068023 | 129.8027274 | 185.6740933 | 0.6990891 | 3120544 | [METRN](http://www.ncbi.nlm.nih.gov/entrez/query.fcgi?cmd=search&db=gene&term=METRN) |  |
| 0.0008663 | 0.033692 | 110.3682632 | 158.0273325 | 0.6984125 | 2320204 | [OPLAH](http://www.ncbi.nlm.nih.gov/entrez/query.fcgi?cmd=search&db=gene&term=OPLAH) | Glutathione metabolism |
| 0.0006454 | 0.029382 | 216.1548808 | 310.4943722 | 0.6961636 | 2480315 | [RPAP2](http://www.ncbi.nlm.nih.gov/entrez/query.fcgi?cmd=search&db=gene&term=RPAP2) |  |
| 0.000402 | 0.025383 | 183.1970782 | 263.2202082 | 0.6959841 | 3360735 | [ZNF473](http://www.ncbi.nlm.nih.gov/entrez/query.fcgi?cmd=search&db=gene&term=ZNF473) |  |
| 0.0003905 | 0.025241 | 139.0340698 | 200.2391045 | 0.6943402 | 3400273 |  |  |
| 0.0048649 | 0.073632 | 248.2328835 | 357.817621 | 0.6937414 | 6250564 | [BCAP29](http://www.ncbi.nlm.nih.gov/entrez/query.fcgi?cmd=search&db=gene&term=BCAP29) |  |
| 0.00468 | 0.072588 | 144.1303942 | 207.76793 | 0.6937086 | 7210280 | [KBTBD7](http://www.ncbi.nlm.nih.gov/entrez/query.fcgi?cmd=search&db=gene&term=KBTBD7) |  |
| 0.0005396 | 0.028008 | 413.643486 | 596.6771002 | 0.6932451 | 1940561 | [CGRRF1](http://www.ncbi.nlm.nih.gov/entrez/query.fcgi?cmd=search&db=gene&term=CGRRF1) |  |
| 0.0034917 | 0.06355 | 2396.383889 | 3457.91288 | 0.6930145 | 510341 | [VEZF1](http://www.ncbi.nlm.nih.gov/entrez/query.fcgi?cmd=search&db=gene&term=VEZF1) |  |
| 0.0048058 | 0.073158 | 113.6815971 | 164.2349082 | 0.692189 | 6350053 | [NUSAP1](http://www.ncbi.nlm.nih.gov/entrez/query.fcgi?cmd=search&db=gene&term=NUSAP1) |  |
| 0.0007261 | 0.030654 | 131.1307441 | 189.8695446 | 0.690636 | 2320762 | [ATP5G2](http://www.ncbi.nlm.nih.gov/entrez/query.fcgi?cmd=search&db=gene&term=ATP5G2) | ATP synthesis, Oxidative phosphorylation |
| 0.0036823 | 0.064645 | 104.7079902 | 151.6370353 | 0.6905173 | 270379 | [SLC2A11](http://www.ncbi.nlm.nih.gov/entrez/query.fcgi?cmd=search&db=gene&term=SLC2A11) |  |
| 0.0007824 | 0.032019 | 946.8583814 | 1371.87985 | 0.6901905 | 3930376 | [LCMT1](http://www.ncbi.nlm.nih.gov/entrez/query.fcgi?cmd=search&db=gene&term=LCMT1) | Aminophosphonate metabolism, Androgen and estrogen metabolism, Histidine metabolism, Nitrobenzene degradation, Selenoamino acid metabolism, Tryptophan metabolism, Tyrosine metabolism |
| 0.0035836 | 0.063979 | 617.3222823 | 897.6905075 | 0.6876783 | 7210075 | [TTC1](http://www.ncbi.nlm.nih.gov/entrez/query.fcgi?cmd=search&db=gene&term=TTC1) |  |
| 0.0008381 | 0.033438 | 519.5239325 | 755.9606901 | 0.6872367 | 5420326 | [RTCD1](http://www.ncbi.nlm.nih.gov/entrez/query.fcgi?cmd=search&db=gene&term=RTCD1) |  |
| 0.0010469 | 0.036768 | 199.1339529 | 290.1954368 | 0.6862064 | 2230201 | [TARBP1](http://www.ncbi.nlm.nih.gov/entrez/query.fcgi?cmd=search&db=gene&term=TARBP1) |  |
| 0.0012385 | 0.0394 | 155.9752613 | 227.4354603 | 0.6858001 | 4280767 | [LARS2](http://www.ncbi.nlm.nih.gov/entrez/query.fcgi?cmd=search&db=gene&term=LARS2) | Aminoacyl-tRNA biosynthesis, Valine, leucine and isoleucine biosynthesis |
| 0.0018549 | 0.047218 | 146.0547465 | 213.2508963 | 0.6848963 | 4730440 |  |  |
| 0.0017992 | 0.04641 | 274.951719 | 402.306065 | 0.6834392 | 6860743 | [C14orf138](http://www.ncbi.nlm.nih.gov/entrez/query.fcgi?cmd=search&db=gene&term=C14orf138) |  |
| 0.0021056 | 0.049924 | 543.4459758 | 795.1855938 | 0.6834203 | 1090367 | [BIVM](http://www.ncbi.nlm.nih.gov/entrez/query.fcgi?cmd=search&db=gene&term=BIVM) |  |
| 0.0042982 | 0.069549 | 110.8476469 | 162.239204 | 0.6832359 | 6580458 | [CNOT4](http://www.ncbi.nlm.nih.gov/entrez/query.fcgi?cmd=search&db=gene&term=CNOT4) | Tryptophan metabolism |
| 0.0042607 | 0.069213 | 782.8181149 | 1147.647031 | 0.682107 | 3450093 | [NSUN2](http://www.ncbi.nlm.nih.gov/entrez/query.fcgi?cmd=search&db=gene&term=NSUN2) |  |
| 0.0028069 | 0.057453 | 113.6271657 | 166.7157054 | 0.6815625 | 7000121 | [INADL](http://www.ncbi.nlm.nih.gov/entrez/query.fcgi?cmd=search&db=gene&term=INADL) | Tight junction |
| 0.0044922 | 0.071298 | 134.5930009 | 197.6674224 | 0.6809063 | 5720689 |  |  |
| 6.88E-05 | 0.016325 | 846.8400619 | 1245.364589 | 0.6799937 | 5310427 | [PATL1](http://www.ncbi.nlm.nih.gov/entrez/query.fcgi?cmd=search&db=gene&term=PATL1) |  |
| 0.0013691 | 0.040911 | 141.2169691 | 207.7328592 | 0.6798008 | 7210327 | [AADACL1](http://www.ncbi.nlm.nih.gov/entrez/query.fcgi?cmd=search&db=gene&term=AADACL1) |  |
| 0.0012072 | 0.038833 | 167.6295704 | 246.8211154 | 0.6791541 | 60360 | [PSMD3](http://www.ncbi.nlm.nih.gov/entrez/query.fcgi?cmd=search&db=gene&term=PSMD3) | Proteasome |
| 0.0004189 | 0.025615 | 146.1403489 | 215.5997314 | 0.6778318 | 730475 | [LOC728758](http://www.ncbi.nlm.nih.gov/entrez/query.fcgi?cmd=search&db=gene&term=LOC728758) |  |
| 0.0023061 | 0.052308 | 97.4614745 | 143.8027694 | 0.6777441 | 460370 | [CLCN1](http://www.ncbi.nlm.nih.gov/entrez/query.fcgi?cmd=search&db=gene&term=CLCN1) | immunology |
| 0.0026008 | 0.05526 | 9000.937066 | 13317.87266 | 0.675854 | 3450743 | [ATP5H](http://www.ncbi.nlm.nih.gov/entrez/query.fcgi?cmd=search&db=gene&term=ATP5H) | ATP synthesis, Oxidative phosphorylation |
| 0.0007739 | 0.031929 | 636.9850445 | 942.6530392 | 0.6757365 | 990167 | [C1orf52](http://www.ncbi.nlm.nih.gov/entrez/query.fcgi?cmd=search&db=gene&term=C1orf52) |  |
| 0.0032124 | 0.060896 | 297.91253 | 441.1796789 | 0.6752635 | 2450367 | [LRIG1](http://www.ncbi.nlm.nih.gov/entrez/query.fcgi?cmd=search&db=gene&term=LRIG1) |  |
| 0.0025629 | 0.054816 | 107.2024296 | 158.9419079 | 0.6744755 | 3130669 | [SATB1](http://www.ncbi.nlm.nih.gov/entrez/query.fcgi?cmd=search&db=gene&term=SATB1) | gene_regulation, transcription |
| 0.0002056 | 0.020451 | 171.7752953 | 254.7082937 | 0.6744001 | 6660674 | [ERGIC2](http://www.ncbi.nlm.nih.gov/entrez/query.fcgi?cmd=search&db=gene&term=ERGIC2) |  |
| 4.85E-05 | 0.015375 | 131.3940019 | 194.8475051 | 0.6743427 | 2350215 | [EXOC4](http://www.ncbi.nlm.nih.gov/entrez/query.fcgi?cmd=search&db=gene&term=EXOC4) | Tight junction |
| 0.000669 | 0.029894 | 5191.843715 | 7717.814585 | 0.672709 | 4060326 | [ATP5F1](http://www.ncbi.nlm.nih.gov/entrez/query.fcgi?cmd=search&db=gene&term=ATP5F1) | ATP synthesis, Oxidative phosphorylation |
| 0.0025166 | 0.054402 | 740.5174279 | 1100.897754 | 0.6726487 | 4830056 | [ARPC5L](http://www.ncbi.nlm.nih.gov/entrez/query.fcgi?cmd=search&db=gene&term=ARPC5L) | Regulation of actin cytoskeleton |
| 6.99E-05 | 0.016325 | 126.9908572 | 188.9296851 | 0.6721594 | 3840398 | [RSRC2](http://www.ncbi.nlm.nih.gov/entrez/query.fcgi?cmd=search&db=gene&term=RSRC2) |  |
| 0.0042532 | 0.069183 | 136.0045121 | 202.6869037 | 0.6710079 | 4010156 | [FLJ46309](http://www.ncbi.nlm.nih.gov/entrez/query.fcgi?cmd=search&db=gene&term=FLJ46309) |  |
| 0.0010852 | 0.037337 | 117.4162407 | 175.0793155 | 0.670646 | 4570546 | [LEAP2](http://www.ncbi.nlm.nih.gov/entrez/query.fcgi?cmd=search&db=gene&term=LEAP2) |  |
| 0.0001815 | 0.019937 | 139.886926 | 208.9075509 | 0.6696116 | 630243 | [ATAD4](http://www.ncbi.nlm.nih.gov/entrez/query.fcgi?cmd=search&db=gene&term=ATAD4) |  |
| 0.0020675 | 0.049303 | 1753.710109 | 2619.495834 | 0.6694838 | 1300491 | [POLE3](http://www.ncbi.nlm.nih.gov/entrez/query.fcgi?cmd=search&db=gene&term=POLE3) | DNA polymerase, Purine metabolism, Pyrimidine metabolism |
| 0.0005184 | 0.02772 | 339.3023523 | 506.9587972 | 0.6692898 | 2970368 | [ZW10](http://www.ncbi.nlm.nih.gov/entrez/query.fcgi?cmd=search&db=gene&term=ZW10) |  |
| 0.0017863 | 0.046294 | 13739.84864 | 20550.06281 | 0.6686037 | 730228 | [VDAC3](http://www.ncbi.nlm.nih.gov/entrez/query.fcgi?cmd=search&db=gene&term=VDAC3) | Calcium signaling pathway |
| 0.0013306 | 0.040384 | 447.8415923 | 670.5752954 | 0.6678468 | 1990291 | [CRADD](http://www.ncbi.nlm.nih.gov/entrez/query.fcgi?cmd=search&db=gene&term=CRADD) | HIV-I Nef: negative effector of Fas and TNF, TNF/Stress Related Signaling, TNFR1 Signaling Pathway |
| 0.0017116 | 0.045484 | 467.3511527 | 700.2373524 | 0.6674182 | 5270138 | [PPP2R3C](http://www.ncbi.nlm.nih.gov/entrez/query.fcgi?cmd=search&db=gene&term=PPP2R3C) |  |
| 0.0016222 | 0.044324 | 109.8815732 | 164.7167887 | 0.6670939 | 6580670 | [ITGB1BP2](http://www.ncbi.nlm.nih.gov/entrez/query.fcgi?cmd=search&db=gene&term=ITGB1BP2) |  |
| 0.0031013 | 0.059971 | 120.7762629 | 181.2750962 | 0.6662595 | 940386 | [TXNL1](http://www.ncbi.nlm.nih.gov/entrez/query.fcgi?cmd=search&db=gene&term=TXNL1) |  |
| 0.0019432 | 0.048364 | 161.5734096 | 243.0517377 | 0.6647696 | 6200634 | [FIGNL1](http://www.ncbi.nlm.nih.gov/entrez/query.fcgi?cmd=search&db=gene&term=FIGNL1) |  |
| 0.0026429 | 0.055706 | 122.5084066 | 184.9926967 | 0.6622337 | 4850414 | [KIAA0564](http://www.ncbi.nlm.nih.gov/entrez/query.fcgi?cmd=search&db=gene&term=KIAA0564) |  |
| 0.0005723 | 0.028659 | 96.5903702 | 145.8588262 | 0.6622182 | 2100356 | [PLCL1](http://www.ncbi.nlm.nih.gov/entrez/query.fcgi?cmd=search&db=gene&term=PLCL1) |  |
| 0.0015987 | 0.044064 | 249.6103177 | 377.4531984 | 0.6613014 | 450468 | [MRPL44](http://www.ncbi.nlm.nih.gov/entrez/query.fcgi?cmd=search&db=gene&term=MRPL44) |  |
| 0.0048147 | 0.073255 | 245.3691582 | 371.4409877 | 0.6605872 | 4260682 | [NASP](http://www.ncbi.nlm.nih.gov/entrez/query.fcgi?cmd=search&db=gene&term=NASP) |  |
| 0.0013335 | 0.04043 | 172.1384844 | 261.4814703 | 0.65832 | 1190327 | [ACOX1](http://www.ncbi.nlm.nih.gov/entrez/query.fcgi?cmd=search&db=gene&term=ACOX1) | Mechanism of Gene Regulation by Peroxisome Proliferators via PPARa(alpha), Fatty acid metabolism, PPAR signaling pathway |
| 0.0037967 | 0.065513 | 287.6848289 | 437.6544715 | 0.6573332 | 1090221 | [VPS37A](http://www.ncbi.nlm.nih.gov/entrez/query.fcgi?cmd=search&db=gene&term=VPS37A) |  |
| 0.0019401 | 0.048328 | 158.9489475 | 242.0174874 | 0.6567664 | 3400039 |  |  |
| 0.0004879 | 0.027279 | 113.7600878 | 173.2786774 | 0.6565152 | 830221 | [TNNI3K](http://www.ncbi.nlm.nih.gov/entrez/query.fcgi?cmd=search&db=gene&term=TNNI3K) |  |
| 0.0005442 | 0.028008 | 183.8762821 | 280.850981 | 0.6547112 | 2030228 | [ATG5](http://www.ncbi.nlm.nih.gov/entrez/query.fcgi?cmd=search&db=gene&term=ATG5) | Regulation of autophagy |
| 0.0005181 | 0.02772 | 114.1097947 | 174.573019 | 0.6536508 | 990474 | [TAF9](http://www.ncbi.nlm.nih.gov/entrez/query.fcgi?cmd=search&db=gene&term=TAF9) | Basal transcription factors |
| 0.0011838 | 0.038641 | 816.3816783 | 1249.879948 | 0.6531681 | 2810364 | [KLHDC2](http://www.ncbi.nlm.nih.gov/entrez/query.fcgi?cmd=search&db=gene&term=KLHDC2) |  |
| 0.0008684 | 0.033692 | 157.6205992 | 241.4738985 | 0.6527438 | 2600128 | [DPF3](http://www.ncbi.nlm.nih.gov/entrez/query.fcgi?cmd=search&db=gene&term=DPF3) |  |
| 2.44E-05 | 0.013372 | 108.5055787 | 166.2679242 | 0.6525948 | 3840719 | [U1SNRNPBP](http://www.ncbi.nlm.nih.gov/entrez/query.fcgi?cmd=search&db=gene&term=U1SNRNPBP) |  |
| 0.0001663 | 0.019753 | 166.6092261 | 255.6560057 | 0.651693 | 5550537 | [OTUD6B](http://www.ncbi.nlm.nih.gov/entrez/query.fcgi?cmd=search&db=gene&term=OTUD6B) |  |
| 0.0031036 | 0.059971 | 140.6133574 | 215.9414961 | 0.6511641 | 160068 | [CCDC69](http://www.ncbi.nlm.nih.gov/entrez/query.fcgi?cmd=search&db=gene&term=CCDC69) |  |
| 0.0004441 | 0.026228 | 1066.101315 | 1637.790711 | 0.6509387 | 5090408 | [LRIG1](http://www.ncbi.nlm.nih.gov/entrez/query.fcgi?cmd=search&db=gene&term=LRIG1) |  |
| 0.0046937 | 0.072636 | 302.228469 | 464.3794064 | 0.6508223 | 1410427 | [ZRANB1](http://www.ncbi.nlm.nih.gov/entrez/query.fcgi?cmd=search&db=gene&term=ZRANB1) |  |
| 0.0011663 | 0.038487 | 198.9512617 | 305.8810407 | 0.6504204 | 4570603 | [C21orf51](http://www.ncbi.nlm.nih.gov/entrez/query.fcgi?cmd=search&db=gene&term=C21orf51) |  |
| 0.0002934 | 0.023094 | 492.5219978 | 758.5576762 | 0.6492875 | 1260025 | [CDK5RAP1](http://www.ncbi.nlm.nih.gov/entrez/query.fcgi?cmd=search&db=gene&term=CDK5RAP1) |  |
| 0.0047668 | 0.072968 | 117.5000964 | 180.9796879 | 0.6492447 | 1010521 | [SP4](http://www.ncbi.nlm.nih.gov/entrez/query.fcgi?cmd=search&db=gene&term=SP4) |  |
| 0.0018251 | 0.046787 | 147.4558727 | 227.4401586 | 0.6483282 | 6280386 | [LOC389203](http://www.ncbi.nlm.nih.gov/entrez/query.fcgi?cmd=search&db=gene&term=LOC389203) |  |
| 0.0021274 | 0.050133 | 275.6052838 | 425.6338994 | 0.6475172 | 7550753 | [BCL2L12](http://www.ncbi.nlm.nih.gov/entrez/query.fcgi?cmd=search&db=gene&term=BCL2L12) |  |
| 0.0044071 | 0.070643 | 522.1070461 | 807.9045248 | 0.6462484 | 7210040 | [WDR37](http://www.ncbi.nlm.nih.gov/entrez/query.fcgi?cmd=search&db=gene&term=WDR37) |  |
| 0.0013037 | 0.039956 | 379.7175334 | 588.8624037 | 0.6448324 | 3170669 | [REV1](http://www.ncbi.nlm.nih.gov/entrez/query.fcgi?cmd=search&db=gene&term=REV1) | DNA polymerase |
| 0.0030172 | 0.059206 | 1944.741367 | 3018.085004 | 0.6443627 | 3850239 | [TRIM54](http://www.ncbi.nlm.nih.gov/entrez/query.fcgi?cmd=search&db=gene&term=TRIM54) |  |
| 0.0040686 | 0.06788 | 103.54299 | 160.6955485 | 0.6443426 | 1940435 | [C6orf66](http://www.ncbi.nlm.nih.gov/entrez/query.fcgi?cmd=search&db=gene&term=C6orf66) |  |
| 0.0001464 | 0.019068 | 171.3436397 | 265.9444267 | 0.6442836 | 3130102 | [TSFM](http://www.ncbi.nlm.nih.gov/entrez/query.fcgi?cmd=search&db=gene&term=TSFM) |  |
| 0.0003359 | 0.024267 | 688.4674627 | 1070.098808 | 0.6433681 | 6860092 | [PRKAG1](http://www.ncbi.nlm.nih.gov/entrez/query.fcgi?cmd=search&db=gene&term=PRKAG1) | Adipocytokine signaling pathway, Insulin signaling pathway, cell_cycle, cell_signaling, signal_transduction |
| 0.0047904 | 0.073 | 105.5589164 | 164.304907 | 0.6424575 | 2900681 | [LOC200810](http://www.ncbi.nlm.nih.gov/entrez/query.fcgi?cmd=search&db=gene&term=LOC200810) |  |
| 0.0030368 | 0.059237 | 150.4952013 | 234.4653713 | 0.6418654 | 5420300 | [TTC32](http://www.ncbi.nlm.nih.gov/entrez/query.fcgi?cmd=search&db=gene&term=TTC32) |  |
| 0.003617 | 0.064294 | 532.0910608 | 829.6100375 | 0.6413749 | 7510538 | [CTBP1](http://www.ncbi.nlm.nih.gov/entrez/query.fcgi?cmd=search&db=gene&term=CTBP1) | SUMOylation as a mechanism to modulate CtBP-dependent gene responses, WNT Signaling Pathway, Notch signaling pathway, Wnt signaling pathway |
| 0.0038981 | 0.0666 | 206.2606509 | 321.855931 | 0.6408478 | 1660674 | [NUDT22](http://www.ncbi.nlm.nih.gov/entrez/query.fcgi?cmd=search&db=gene&term=NUDT22) |  |
| 0.0013494 | 0.040636 | 522.37252 | 815.2708736 | 0.6407349 | 2750592 | [TATDN1](http://www.ncbi.nlm.nih.gov/entrez/query.fcgi?cmd=search&db=gene&term=TATDN1) |  |
| 3.76E-05 | 0.014558 | 1080.793819 | 1687.183351 | 0.6405906 | 2120465 | [ZNF313](http://www.ncbi.nlm.nih.gov/entrez/query.fcgi?cmd=search&db=gene&term=ZNF313) |  |
| 0.0014254 | 0.041657 | 467.2411843 | 729.55652 | 0.6404455 | 5340148 | [TMEM188](http://www.ncbi.nlm.nih.gov/entrez/query.fcgi?cmd=search&db=gene&term=TMEM188) |  |
| 0.0032183 | 0.060896 | 1157.154744 | 1810.655818 | 0.6390805 | 5490524 | [ZNF302](http://www.ncbi.nlm.nih.gov/entrez/query.fcgi?cmd=search&db=gene&term=ZNF302) |  |
| 0.0047063 | 0.072636 | 1469.762965 | 2301.551848 | 0.6385965 | 6040114 | [PPP2R4](http://www.ncbi.nlm.nih.gov/entrez/query.fcgi?cmd=search&db=gene&term=PPP2R4) | Tight junction, cell_cycle, cell_signaling |
| 0.004297 | 0.069549 | 300.9649857 | 471.6628951 | 0.6380934 | 70484 | [AKR7A3](http://www.ncbi.nlm.nih.gov/entrez/query.fcgi?cmd=search&db=gene&term=AKR7A3) |  |
| 0.002284 | 0.05203 | 316.0007934 | 495.6286247 | 0.6375758 | 1980382 | [MED24](http://www.ncbi.nlm.nih.gov/entrez/query.fcgi?cmd=search&db=gene&term=MED24) |  |
| 0.0006435 | 0.029369 | 167.786841 | 263.1904996 | 0.637511 | 7550280 | [C3orf17](http://www.ncbi.nlm.nih.gov/entrez/query.fcgi?cmd=search&db=gene&term=C3orf17) |  |
| 0.0004988 | 0.027596 | 210.5405624 | 331.0138028 | 0.6360477 | 990072 | [SRGAP2](http://www.ncbi.nlm.nih.gov/entrez/query.fcgi?cmd=search&db=gene&term=SRGAP2) | Axon guidance |
| 0.0028027 | 0.057448 | 529.0171339 | 833.9818659 | 0.6343269 | 3440500 | [ELP3](http://www.ncbi.nlm.nih.gov/entrez/query.fcgi?cmd=search&db=gene&term=ELP3) |  |
| 0.002118 | 0.050014 | 890.0456067 | 1403.647419 | 0.6340949 | 1940576 | [RPS6KB1](http://www.ncbi.nlm.nih.gov/entrez/query.fcgi?cmd=search&db=gene&term=RPS6KB1) | CTCF: First Multivalent Nuclear Factor, IL 4 signaling pathway, IL-2 Receptor Beta Chain in T cell Activation, MAPKinase Signaling Pathway, mTOR Signaling Pathway, NFAT and Hypertrophy of the heart (Transcription in the broken heart), Phosphoinositides and their downstream targets., Rac 1 cell motility signaling pathway, Regulation of eIF4e and p70 S6 Kinase, Skeletal muscle hypertrophy is regulated via AKT/mTOR pathway, Insulin signaling pathway, mTOR signaling pathway, TGF-beta signaling pathway, cell_cycle, cell_signaling, signal_transduction |
| 0.0029834 | 0.058853 | 141.2144253 | 222.7124149 | 0.6340662 | 3060358 |  |  |
| 0.0008929 | 0.033935 | 140.9264028 | 222.3584458 | 0.6337803 | 6250075 | [DHTKD1](http://www.ncbi.nlm.nih.gov/entrez/query.fcgi?cmd=search&db=gene&term=DHTKD1) |  |
| 0.0005923 | 0.028704 | 529.8320367 | 836.1319173 | 0.6336704 | 3140167 | [ZNF146](http://www.ncbi.nlm.nih.gov/entrez/query.fcgi?cmd=search&db=gene&term=ZNF146) |  |
| 0.0010999 | 0.037499 | 122.8024827 | 193.8232734 | 0.6335797 | 5900360 | [C17orf75](http://www.ncbi.nlm.nih.gov/entrez/query.fcgi?cmd=search&db=gene&term=C17orf75) |  |
| 0.0021941 | 0.050838 | 161.0770586 | 254.4211928 | 0.6331118 | 5130020 | [TRAF3IP1](http://www.ncbi.nlm.nih.gov/entrez/query.fcgi?cmd=search&db=gene&term=TRAF3IP1) |  |
| 0.0039189 | 0.066776 | 140.9002512 | 222.5719183 | 0.6330549 | 2060300 | [RSBN1L](http://www.ncbi.nlm.nih.gov/entrez/query.fcgi?cmd=search&db=gene&term=RSBN1L) |  |
| 0.00259 | 0.05512 | 384.2688733 | 607.6034076 | 0.6324337 | 1710091 | [IRS1](http://www.ncbi.nlm.nih.gov/entrez/query.fcgi?cmd=search&db=gene&term=IRS1) | immunology |
| 0.0027726 | 0.057195 | 774.944674 | 1229.963279 | 0.6300551 | 630403 | [MRPS31](http://www.ncbi.nlm.nih.gov/entrez/query.fcgi?cmd=search&db=gene&term=MRPS31) |  |
| 0.0014783 | 0.042512 | 178.7050521 | 283.7643061 | 0.6297658 | 1820209 | [LOC647009](http://www.ncbi.nlm.nih.gov/entrez/query.fcgi?cmd=search&db=gene&term=LOC647009) |  |
| 0.0010615 | 0.037012 | 11113.87428 | 17663.88159 | 0.6291864 | 7510014 | [UQCRFS1](http://www.ncbi.nlm.nih.gov/entrez/query.fcgi?cmd=search&db=gene&term=UQCRFS1) | Oxidative phosphorylation |
| 0.0017099 | 0.04548 | 326.2597843 | 518.6551951 | 0.6290495 | 7330180 | [AUH](http://www.ncbi.nlm.nih.gov/entrez/query.fcgi?cmd=search&db=gene&term=AUH) | Valine, leucine and isoleucine degradation, immunology |
| 0.0023618 | 0.052605 | 110.1231615 | 175.4241219 | 0.6277538 | 4070358 | [KLHL23](http://www.ncbi.nlm.nih.gov/entrez/query.fcgi?cmd=search&db=gene&term=KLHL23) |  |
| 0.0006316 | 0.029305 | 216.3767898 | 345.0203628 | 0.6271421 | 4390360 | [PARP6](http://www.ncbi.nlm.nih.gov/entrez/query.fcgi?cmd=search&db=gene&term=PARP6) |  |
| 0.0017887 | 0.046294 | 1403.291629 | 2238.796765 | 0.6268062 | 6280092 | [SLC25A23](http://www.ncbi.nlm.nih.gov/entrez/query.fcgi?cmd=search&db=gene&term=SLC25A23) |  |
| 0.0036614 | 0.064645 | 113.6304237 | 181.3001573 | 0.626753 | 4610598 | [TMEM164](http://www.ncbi.nlm.nih.gov/entrez/query.fcgi?cmd=search&db=gene&term=TMEM164) |  |
| 0.0014919 | 0.042622 | 387.5602648 | 619.1458928 | 0.6259595 | 7330519 | [DDX19A](http://www.ncbi.nlm.nih.gov/entrez/query.fcgi?cmd=search&db=gene&term=DDX19A) |  |
| 0.0031168 | 0.060071 | 179.2500796 | 286.7803541 | 0.6250431 | 4780093 | [OSGEPL1](http://www.ncbi.nlm.nih.gov/entrez/query.fcgi?cmd=search&db=gene&term=OSGEPL1) |  |
| 0.0023655 | 0.052605 | 1247.455292 | 1996.304954 | 0.6248821 | 4780519 | [ZC3HC1](http://www.ncbi.nlm.nih.gov/entrez/query.fcgi?cmd=search&db=gene&term=ZC3HC1) |  |
| 0.0016853 | 0.045282 | 184.6776484 | 296.1273532 | 0.6236427 | 1110167 | [TATDN1](http://www.ncbi.nlm.nih.gov/entrez/query.fcgi?cmd=search&db=gene&term=TATDN1) |  |
| 0.0003064 | 0.02341 | 202.3812 | 324.5262165 | 0.6236205 | 4590102 | [WRB](http://www.ncbi.nlm.nih.gov/entrez/query.fcgi?cmd=search&db=gene&term=WRB) |  |
| 0.0036821 | 0.064645 | 154.7768171 | 248.2773356 | 0.6234029 | 4920162 | [FLYWCH2](http://www.ncbi.nlm.nih.gov/entrez/query.fcgi?cmd=search&db=gene&term=FLYWCH2) |  |
| 0.0019271 | 0.048229 | 231.6652403 | 371.6800326 | 0.6232921 | 4390687 | [POMT1](http://www.ncbi.nlm.nih.gov/entrez/query.fcgi?cmd=search&db=gene&term=POMT1) |  |
| 0.0011472 | 0.038212 | 203.0306951 | 325.855331 | 0.62307 | 2630626 | [BXDC5](http://www.ncbi.nlm.nih.gov/entrez/query.fcgi?cmd=search&db=gene&term=BXDC5) |  |
| 0.0005739 | 0.02869 | 192.0126112 | 308.2407049 | 0.6229307 | 3780164 | [FXC1](http://www.ncbi.nlm.nih.gov/entrez/query.fcgi?cmd=search&db=gene&term=FXC1) |  |
| 0.0042025 | 0.068875 | 255.9203072 | 411.1572063 | 0.6224391 | 3930278 | [TMEM177](http://www.ncbi.nlm.nih.gov/entrez/query.fcgi?cmd=search&db=gene&term=TMEM177) |  |
| 0.0025568 | 0.054806 | 134.4417575 | 216.1487546 | 0.6219872 | 1660181 | [LOC440145](http://www.ncbi.nlm.nih.gov/entrez/query.fcgi?cmd=search&db=gene&term=LOC440145) |  |
| 8.66E-05 | 0.017191 | 2519.806612 | 4052.885161 | 0.6217316 | 1400025 | [PTOV1](http://www.ncbi.nlm.nih.gov/entrez/query.fcgi?cmd=search&db=gene&term=PTOV1) |  |
| 0.0008822 | 0.03376 | 346.3262543 | 558.1563704 | 0.6204825 | 4050358 | [SUMO1P3](http://www.ncbi.nlm.nih.gov/entrez/query.fcgi?cmd=search&db=gene&term=SUMO1P3) |  |
| 0.0044498 | 0.070963 | 162.8146051 | 262.4983677 | 0.62025 | 4570692 | [CPSF3L](http://www.ncbi.nlm.nih.gov/entrez/query.fcgi?cmd=search&db=gene&term=CPSF3L) |  |
| 0.0030689 | 0.059639 | 291.5585096 | 470.1290667 | 0.620167 | 6020497 | [DCTN4](http://www.ncbi.nlm.nih.gov/entrez/query.fcgi?cmd=search&db=gene&term=DCTN4) |  |
| 0.001087 | 0.037337 | 111.1785523 | 179.333398 | 0.6199545 | 540465 | [ENPP5](http://www.ncbi.nlm.nih.gov/entrez/query.fcgi?cmd=search&db=gene&term=ENPP5) |  |
| 0.003597 | 0.064095 | 100.2225358 | 161.6895093 | 0.6198456 | 2680142 |  |  |
| 0.0029007 | 0.058373 | 121.1509416 | 195.4582865 | 0.6198302 | 3710189 | [LIAS](http://www.ncbi.nlm.nih.gov/entrez/query.fcgi?cmd=search&db=gene&term=LIAS) |  |
| 0.0008305 | 0.033292 | 537.6205908 | 868.8775546 | 0.618753 | 4250288 | [UFSP2](http://www.ncbi.nlm.nih.gov/entrez/query.fcgi?cmd=search&db=gene&term=UFSP2) |  |
| 0.0018047 | 0.046475 | 120.3448845 | 194.5112698 | 0.6187039 | 3520070 | [EXOSC9](http://www.ncbi.nlm.nih.gov/entrez/query.fcgi?cmd=search&db=gene&term=EXOSC9) |  |
| 0.0015472 | 0.043319 | 937.7561752 | 1519.191457 | 0.6172732 | 5960722 | [TUSC4](http://www.ncbi.nlm.nih.gov/entrez/query.fcgi?cmd=search&db=gene&term=TUSC4) |  |
| 0.0001951 | 0.020451 | 120.255789 | 195.3703176 | 0.6155274 | 4900017 | [MAP2K6](http://www.ncbi.nlm.nih.gov/entrez/query.fcgi?cmd=search&db=gene&term=MAP2K6) | Control of skeletal myogenesis by HDAC & calcium/calmodulin-dependent kinase (CaMK), fMLP induced chemokine gene expression in HMC-1 cells, Human Cytomegalovirus and Map Kinase Pathways, IL12 and Stat4 Dependent Signaling Pathway in Th1 Development, Keratinocyte Differentiation, MAPKinase Signaling Pathway, NFkB activation by Nontypeable Hemophilus influenzae, p38 MAPK Signaling Pathway , Signal transduction through IL1R, TNF/Stress Related Signaling, Toll-Like Receptor Pathway, Fc epsilon RI signaling pathway, GnRH signaling pathway, MAPK signaling pathway, Toll-like receptor signaling pathway |
| 0.0042537 | 0.069183 | 5005.837728 | 8135.571079 | 0.6153026 | 2360682 | [PSMB6](http://www.ncbi.nlm.nih.gov/entrez/query.fcgi?cmd=search&db=gene&term=PSMB6) | Proteasome |
| 0.0047407 | 0.072809 | 388.6073224 | 631.6884367 | 0.6151883 | 840292 | [RBM15](http://www.ncbi.nlm.nih.gov/entrez/query.fcgi?cmd=search&db=gene&term=RBM15) |  |
| 0.0024581 | 0.053762 | 528.2285374 | 859.8300006 | 0.6143407 | 2360494 |  |  |
| 0.0045036 | 0.071346 | 2763.185586 | 4501.169194 | 0.6138817 | 4480338 | [AKTIP](http://www.ncbi.nlm.nih.gov/entrez/query.fcgi?cmd=search&db=gene&term=AKTIP) |  |
| 0.0035637 | 0.063887 | 503.1325183 | 819.6534188 | 0.6138357 | 4210102 | [TMEM103](http://www.ncbi.nlm.nih.gov/entrez/query.fcgi?cmd=search&db=gene&term=TMEM103) |  |
| 0.0023862 | 0.052863 | 202.9613703 | 330.7587113 | 0.6136237 | 160026 | [MTMR6](http://www.ncbi.nlm.nih.gov/entrez/query.fcgi?cmd=search&db=gene&term=MTMR6) | Aminosugars metabolism, Fructose and mannose metabolism, Nicotinate and nicotinamide metabolism, Riboflavin metabolism, Thiamine metabolism, Vitamin B6 metabolism |
| 0.0041565 | 0.068477 | 162.4090097 | 264.7914722 | 0.6133468 | 6770201 | [GTF2IRD2P](http://www.ncbi.nlm.nih.gov/entrez/query.fcgi?cmd=search&db=gene&term=GTF2IRD2P) |  |
| 0.0007735 | 0.031929 | 148.6484601 | 242.7364143 | 0.6123863 | 2640195 | [LRRC15](http://www.ncbi.nlm.nih.gov/entrez/query.fcgi?cmd=search&db=gene&term=LRRC15) |  |
| 0.0025825 | 0.055088 | 1715.629241 | 2801.779944 | 0.6123355 | 730605 | [MRPS22](http://www.ncbi.nlm.nih.gov/entrez/query.fcgi?cmd=search&db=gene&term=MRPS22) |  |
| 0.0047567 | 0.072868 | 183.8535539 | 300.4629346 | 0.6119009 | 7050180 | [SLC22A5](http://www.ncbi.nlm.nih.gov/entrez/query.fcgi?cmd=search&db=gene&term=SLC22A5) |  |
| 0.0028542 | 0.058112 | 356.876523 | 583.4183354 | 0.6116992 | 1500753 | [VARS2](http://www.ncbi.nlm.nih.gov/entrez/query.fcgi?cmd=search&db=gene&term=VARS2) |  |
| 0.0003234 | 0.023725 | 1048.377413 | 1714.498355 | 0.6114776 | 2260632 | [RY1](http://www.ncbi.nlm.nih.gov/entrez/query.fcgi?cmd=search&db=gene&term=RY1) |  |
| 0.0006923 | 0.030043 | 361.9966138 | 592.2915088 | 0.6111798 | 7150554 | [LCOR](http://www.ncbi.nlm.nih.gov/entrez/query.fcgi?cmd=search&db=gene&term=LCOR) |  |
| 0.0030504 | 0.059383 | 131.8638856 | 215.8331618 | 0.6109529 | 7150259 | [C7orf49](http://www.ncbi.nlm.nih.gov/entrez/query.fcgi?cmd=search&db=gene&term=C7orf49) |  |
| 0.0040848 | 0.068002 | 1981.780092 | 3243.902211 | 0.6109247 | 10435 | [LYRM5](http://www.ncbi.nlm.nih.gov/entrez/query.fcgi?cmd=search&db=gene&term=LYRM5) |  |
| 1.81E-05 | 0.012893 | 114.637499 | 187.6598341 | 0.6108793 | 3830021 | [C11orf73](http://www.ncbi.nlm.nih.gov/entrez/query.fcgi?cmd=search&db=gene&term=C11orf73) |  |
| 0.0044226 | 0.070813 | 1680.641507 | 2751.59437 | 0.6107882 | 6180022 | [PCM1](http://www.ncbi.nlm.nih.gov/entrez/query.fcgi?cmd=search&db=gene&term=PCM1) | immunology |
| 9.03E-05 | 0.017191 | 1129.774773 | 1849.79955 | 0.6107552 | 770021 | [PRKRA](http://www.ncbi.nlm.nih.gov/entrez/query.fcgi?cmd=search&db=gene&term=PRKRA) |  |
| 0.0037238 | 0.065038 | 1411.533821 | 2319.174525 | 0.6086363 | 6580370 | [THAP11](http://www.ncbi.nlm.nih.gov/entrez/query.fcgi?cmd=search&db=gene&term=THAP11) |  |
| 0.0048519 | 0.073632 | 106.7168778 | 175.3765261 | 0.6085015 | 2970681 | [LOC652164](http://www.ncbi.nlm.nih.gov/entrez/query.fcgi?cmd=search&db=gene&term=LOC652164) |  |
| 0.0019014 | 0.047898 | 120.0821715 | 197.6921034 | 0.6074202 | 5560400 | [SLC14A1](http://www.ncbi.nlm.nih.gov/entrez/query.fcgi?cmd=search&db=gene&term=SLC14A1) | immunology |
| 0.0006357 | 0.029354 | 135.5093705 | 223.1525618 | 0.6072499 | 5910554 | [ADAL](http://www.ncbi.nlm.nih.gov/entrez/query.fcgi?cmd=search&db=gene&term=ADAL) |  |
| 0.0009306 | 0.034476 | 105.6865919 | 174.2955749 | 0.6063642 | 5560021 | [THAP7](http://www.ncbi.nlm.nih.gov/entrez/query.fcgi?cmd=search&db=gene&term=THAP7) |  |
| 0.0001067 | 0.017911 | 162.3002251 | 267.795476 | 0.6060604 | 430192 | [PDSS1](http://www.ncbi.nlm.nih.gov/entrez/query.fcgi?cmd=search&db=gene&term=PDSS1) |  |
| 0.0003745 | 0.024685 | 187.092977 | 308.8972505 | 0.6056803 | 7210072 | [MRPL30](http://www.ncbi.nlm.nih.gov/entrez/query.fcgi?cmd=search&db=gene&term=MRPL30) |  |
| 0.0009605 | 0.035003 | 120.8912836 | 199.9199945 | 0.6046983 | 4040747 | [UQCR](http://www.ncbi.nlm.nih.gov/entrez/query.fcgi?cmd=search&db=gene&term=UQCR) | Oxidative phosphorylation |
| 0.00457 | 0.071969 | 405.5385736 | 671.0766049 | 0.6043104 | 7330047 | [EIF5B](http://www.ncbi.nlm.nih.gov/entrez/query.fcgi?cmd=search&db=gene&term=EIF5B) |  |
| 0.0001222 | 0.018034 | 191.1672532 | 316.4822814 | 0.6040378 | 4210241 | [PHF20](http://www.ncbi.nlm.nih.gov/entrez/query.fcgi?cmd=search&db=gene&term=PHF20) |  |
| 0.0010885 | 0.037337 | 8530.390679 | 14123.86374 | 0.60397 | 6110754 | [ATP5O](http://www.ncbi.nlm.nih.gov/entrez/query.fcgi?cmd=search&db=gene&term=ATP5O) | ATP synthesis, Oxidative phosphorylation |
| 0.0006003 | 0.028772 | 380.9925267 | 631.1369642 | 0.6036606 | 6520605 | [SMNDC1](http://www.ncbi.nlm.nih.gov/entrez/query.fcgi?cmd=search&db=gene&term=SMNDC1) |  |
| 0.0047541 | 0.072867 | 144.6590804 | 239.9063222 | 0.6029815 | 7160349 | [ZNF398](http://www.ncbi.nlm.nih.gov/entrez/query.fcgi?cmd=search&db=gene&term=ZNF398) |  |
| 0.0023252 | 0.052324 | 150.1062543 | 249.2284252 | 0.6022838 | 770382 | [ST3GAL3](http://www.ncbi.nlm.nih.gov/entrez/query.fcgi?cmd=search&db=gene&term=ST3GAL3) | Glycan structures - biosynthesis 1, Glycan structures - biosynthesis 2, Glycosphingolipid biosynthesis - lactoseries, Keratan sulfate biosynthesis |
| 0.0029032 | 0.058373 | 240.5064904 | 399.6131772 | 0.6018482 | 1770717 | [HIRIP3](http://www.ncbi.nlm.nih.gov/entrez/query.fcgi?cmd=search&db=gene&term=HIRIP3) |  |
| 0.0023196 | 0.052324 | 254.4483766 | 423.0125584 | 0.6015149 | 4180725 | [NR2C1](http://www.ncbi.nlm.nih.gov/entrez/query.fcgi?cmd=search&db=gene&term=NR2C1) |  |
| 0.002369 | 0.052605 | 180.8613635 | 300.7056184 | 0.6014565 | 460427 | [TESK2](http://www.ncbi.nlm.nih.gov/entrez/query.fcgi?cmd=search&db=gene&term=TESK2) |  |
| 0.000882 | 0.03376 | 229.3837486 | 382.062437 | 0.6003829 | 5310110 | [HAX1](http://www.ncbi.nlm.nih.gov/entrez/query.fcgi?cmd=search&db=gene&term=HAX1) |  |
| 0.0038458 | 0.065994 | 1759.80974 | 2933.772602 | 0.5998453 | 110669 | [USO1](http://www.ncbi.nlm.nih.gov/entrez/query.fcgi?cmd=search&db=gene&term=USO1) |  |
| 7.77E-05 | 0.016354 | 185.3040718 | 309.3210871 | 0.5990671 | 7550113 | [PARP2](http://www.ncbi.nlm.nih.gov/entrez/query.fcgi?cmd=search&db=gene&term=PARP2) |  |
| 0.0017379 | 0.045557 | 111.318792 | 186.0337088 | 0.5983797 | 2100519 | [IPP](http://www.ncbi.nlm.nih.gov/entrez/query.fcgi?cmd=search&db=gene&term=IPP) |  |
| 0.0037711 | 0.065289 | 282.2787871 | 471.7641586 | 0.5983473 | 1450397 | [HSD17B8](http://www.ncbi.nlm.nih.gov/entrez/query.fcgi?cmd=search&db=gene&term=HSD17B8) | Androgen and estrogen metabolism |
| 0.0047215 | 0.072636 | 1676.752357 | 2803.794804 | 0.5980296 | 5860242 | [LOC642755](http://www.ncbi.nlm.nih.gov/entrez/query.fcgi?cmd=search&db=gene&term=LOC642755) |  |
| 0.0002695 | 0.022558 | 458.7403939 | 767.9940911 | 0.5973228 | 6130368 | [LOC729446](http://www.ncbi.nlm.nih.gov/entrez/query.fcgi?cmd=search&db=gene&term=LOC729446) |  |
| 0.0043771 | 0.07037 | 299.4358827 | 501.3736263 | 0.597231 | 2140563 | [UBE4B](http://www.ncbi.nlm.nih.gov/entrez/query.fcgi?cmd=search&db=gene&term=UBE4B) |  |
| 0.00122 | 0.039002 | 285.2610607 | 478.4936303 | 0.5961648 | 4890056 | [LOC283951](http://www.ncbi.nlm.nih.gov/entrez/query.fcgi?cmd=search&db=gene&term=LOC283951) |  |
| 0.0036635 | 0.064645 | 128.5813574 | 215.8747017 | 0.5956296 | 2350192 | [ARL6IP4](http://www.ncbi.nlm.nih.gov/entrez/query.fcgi?cmd=search&db=gene&term=ARL6IP4) |  |
| 0.004627 | 0.072259 | 208.9924106 | 350.9898038 | 0.5954373 | 6580674 |  |  |
| 0.0009039 | 0.034003 | 246.1998797 | 414.2344558 | 0.5943491 | 5820646 | [CALCOCO2](http://www.ncbi.nlm.nih.gov/entrez/query.fcgi?cmd=search&db=gene&term=CALCOCO2) |  |
| 0.0010591 | 0.037012 | 778.7776819 | 1311.194804 | 0.5939451 | 7200670 | [MRPS30](http://www.ncbi.nlm.nih.gov/entrez/query.fcgi?cmd=search&db=gene&term=MRPS30) |  |
| 0.0014715 | 0.042401 | 225.1680493 | 379.7165921 | 0.5929898 | 1110544 | [GPHN](http://www.ncbi.nlm.nih.gov/entrez/query.fcgi?cmd=search&db=gene&term=GPHN) | Gamma-aminobutyric Acid Receptor Life Cycle |
| 0.0005246 | 0.02772 | 187.2934229 | 316.1227554 | 0.5924706 | 670128 | [PRDM10](http://www.ncbi.nlm.nih.gov/entrez/query.fcgi?cmd=search&db=gene&term=PRDM10) |  |
| 0.0038387 | 0.065994 | 3271.588917 | 5525.866149 | 0.59205 | 6900278 | [FYCO1](http://www.ncbi.nlm.nih.gov/entrez/query.fcgi?cmd=search&db=gene&term=FYCO1) |  |
| 0.0023347 | 0.052364 | 324.3087623 | 548.7682905 | 0.5909758 | 5550356 | [EXOSC8](http://www.ncbi.nlm.nih.gov/entrez/query.fcgi?cmd=search&db=gene&term=EXOSC8) |  |
| 0.0016804 | 0.045192 | 938.3190503 | 1591.099638 | 0.5897299 | 7040037 | [CUEDC2](http://www.ncbi.nlm.nih.gov/entrez/query.fcgi?cmd=search&db=gene&term=CUEDC2) |  |
| 0.0049829 | 0.074372 | 462.7556083 | 785.4305205 | 0.5891745 | 6550180 | [ZMAT5](http://www.ncbi.nlm.nih.gov/entrez/query.fcgi?cmd=search&db=gene&term=ZMAT5) |  |
| 0.0028817 | 0.058288 | 325.3357645 | 552.5765055 | 0.5887615 | 6250288 | [MPP5](http://www.ncbi.nlm.nih.gov/entrez/query.fcgi?cmd=search&db=gene&term=MPP5) | Tight junction |
| 0.0004656 | 0.026685 | 1146.580126 | 1947.534289 | 0.5887342 | 4210762 | [MRPL46](http://www.ncbi.nlm.nih.gov/entrez/query.fcgi?cmd=search&db=gene&term=MRPL46) |  |
| 0.0033934 | 0.062509 | 371.9846338 | 632.2221067 | 0.5883765 | 5870487 | [IMMP2L](http://www.ncbi.nlm.nih.gov/entrez/query.fcgi?cmd=search&db=gene&term=IMMP2L) |  |
| 7.66E-05 | 0.016325 | 283.5670655 | 482.5323572 | 0.5876644 | 7160025 | [ING2](http://www.ncbi.nlm.nih.gov/entrez/query.fcgi?cmd=search&db=gene&term=ING2) |  |
| 0.0047475 | 0.072809 | 295.8135594 | 503.6959067 | 0.587286 | 160358 | [C16orf13](http://www.ncbi.nlm.nih.gov/entrez/query.fcgi?cmd=search&db=gene&term=C16orf13) |  |
| 0.002951 | 0.058427 | 446.5090317 | 760.4894007 | 0.5871338 | 3800445 | [MRPL24](http://www.ncbi.nlm.nih.gov/entrez/query.fcgi?cmd=search&db=gene&term=MRPL24) |  |
| 0.002922 | 0.058389 | 150.5270516 | 257.1046047 | 0.5854701 | 7570619 | [CHCHD1](http://www.ncbi.nlm.nih.gov/entrez/query.fcgi?cmd=search&db=gene&term=CHCHD1) |  |
| 0.0007727 | 0.031929 | 986.0922535 | 1685.282017 | 0.58512 | 6330242 | [OGFOD1](http://www.ncbi.nlm.nih.gov/entrez/query.fcgi?cmd=search&db=gene&term=OGFOD1) |  |
| 0.0014995 | 0.042657 | 109.1875605 | 186.8453729 | 0.5843739 | 520681 | [UQCRB](http://www.ncbi.nlm.nih.gov/entrez/query.fcgi?cmd=search&db=gene&term=UQCRB) | Oxidative phosphorylation |
| 0.0035468 | 0.063738 | 462.7827576 | 793.3347147 | 0.5833386 | 5890168 | [TMLHE](http://www.ncbi.nlm.nih.gov/entrez/query.fcgi?cmd=search&db=gene&term=TMLHE) | Lysine degradation |
| 0.0012439 | 0.039442 | 399.4399925 | 684.7853276 | 0.5833069 | 3990382 | [C18orf37](http://www.ncbi.nlm.nih.gov/entrez/query.fcgi?cmd=search&db=gene&term=C18orf37) |  |
| 0.0004392 | 0.026228 | 185.0762529 | 317.5966414 | 0.58274 | 4210491 | [TBCE](http://www.ncbi.nlm.nih.gov/entrez/query.fcgi?cmd=search&db=gene&term=TBCE) |  |
| 0.0018907 | 0.047687 | 131.1703747 | 225.4015768 | 0.5819408 | 2850382 | [TMTC4](http://www.ncbi.nlm.nih.gov/entrez/query.fcgi?cmd=search&db=gene&term=TMTC4) |  |
| 0.0001124 | 0.018033 | 116.4912422 | 200.2814328 | 0.5816378 | 610554 | [USP25](http://www.ncbi.nlm.nih.gov/entrez/query.fcgi?cmd=search&db=gene&term=USP25) |  |
| 0.0027021 | 0.056544 | 146.8371197 | 252.9260528 | 0.5805536 | 4850184 | [CASZ1](http://www.ncbi.nlm.nih.gov/entrez/query.fcgi?cmd=search&db=gene&term=CASZ1) |  |
| 0.0001201 | 0.018034 | 107.4223089 | 185.1241101 | 0.5802718 | 5890408 | [LOC644715](http://www.ncbi.nlm.nih.gov/entrez/query.fcgi?cmd=search&db=gene&term=LOC644715) |  |
| 4.00E-07 | 0.002324 | 124.2622299 | 214.2557408 | 0.5799715 | 3130239 | [ART5](http://www.ncbi.nlm.nih.gov/entrez/query.fcgi?cmd=search&db=gene&term=ART5) |  |
| 2.06E-05 | 0.013372 | 442.0539207 | 762.7414824 | 0.5795593 | 1470475 | [SECISBP2](http://www.ncbi.nlm.nih.gov/entrez/query.fcgi?cmd=search&db=gene&term=SECISBP2) |  |
| 0.0035528 | 0.063738 | 171.6714854 | 296.5281798 | 0.5789382 | 1450451 | [DOPEY2](http://www.ncbi.nlm.nih.gov/entrez/query.fcgi?cmd=search&db=gene&term=DOPEY2) |  |
| 0.0028059 | 0.057453 | 348.3378248 | 602.5412536 | 0.5781145 | 6560520 | [TLE2](http://www.ncbi.nlm.nih.gov/entrez/query.fcgi?cmd=search&db=gene&term=TLE2) |  |
| 0.0024821 | 0.05386 | 145.5182902 | 251.9299377 | 0.5776141 | 2630400 | [CSTF2T](http://www.ncbi.nlm.nih.gov/entrez/query.fcgi?cmd=search&db=gene&term=CSTF2T) |  |
| 0.0033115 | 0.061735 | 191.7749283 | 332.0674709 | 0.577518 | 5820427 | [PDIK1L](http://www.ncbi.nlm.nih.gov/entrez/query.fcgi?cmd=search&db=gene&term=PDIK1L) |  |
| 0.0004771 | 0.027065 | 129.826594 | 224.8502492 | 0.5773914 | 6380092 | [CAV3](http://www.ncbi.nlm.nih.gov/entrez/query.fcgi?cmd=search&db=gene&term=CAV3) | Focal adhesion, immunology |
| 0.0001068 | 0.017911 | 119.3937658 | 206.8407475 | 0.5772256 | 6840750 | [GKAP1](http://www.ncbi.nlm.nih.gov/entrez/query.fcgi?cmd=search&db=gene&term=GKAP1) |  |
| 0.0002772 | 0.022708 | 393.7597162 | 682.1593547 | 0.5772254 | 4610164 | [MLH1](http://www.ncbi.nlm.nih.gov/entrez/query.fcgi?cmd=search&db=gene&term=MLH1) | Colorectal cancer, DNA_damage, DNA_replication, tsonc |
| 0.001068 | 0.037055 | 241.9800144 | 419.5462537 | 0.576766 | 7320148 | [ZNF419](http://www.ncbi.nlm.nih.gov/entrez/query.fcgi?cmd=search&db=gene&term=ZNF419) |  |
| 0.0015496 | 0.043319 | 113.5113555 | 196.8158861 | 0.5767388 | 2350128 | [SLIC1](http://www.ncbi.nlm.nih.gov/entrez/query.fcgi?cmd=search&db=gene&term=SLIC1) |  |
| 0.0011534 | 0.038243 | 441.0615092 | 764.7806676 | 0.5767163 | 3130577 | [C6orf153](http://www.ncbi.nlm.nih.gov/entrez/query.fcgi?cmd=search&db=gene&term=C6orf153) |  |
| 0.0012806 | 0.039738 | 114.6070018 | 198.7340534 | 0.5766853 | 6200066 | [CUL3](http://www.ncbi.nlm.nih.gov/entrez/query.fcgi?cmd=search&db=gene&term=CUL3) | Ubiquitin mediated proteolysis |
| 0.0020272 | 0.04919 | 208.0563082 | 361.4672662 | 0.5755882 | 630274 | [PCBP4](http://www.ncbi.nlm.nih.gov/entrez/query.fcgi?cmd=search&db=gene&term=PCBP4) |  |
| 0.0004466 | 0.026228 | 3313.399227 | 5762.320792 | 0.5750112 | 1980201 | [WWP1](http://www.ncbi.nlm.nih.gov/entrez/query.fcgi?cmd=search&db=gene&term=WWP1) | Dentatorubropallidoluysian atrophy (DRPLA), Ubiquitin mediated proteolysis |
| 0.0037202 | 0.065014 | 157.8554818 | 274.9827324 | 0.574056 | 7000411 | [GTPBP8](http://www.ncbi.nlm.nih.gov/entrez/query.fcgi?cmd=search&db=gene&term=GTPBP8) |  |
| 0.0008419 | 0.033471 | 228.8099374 | 398.7049756 | 0.5738828 | 2450047 | [CASZ1](http://www.ncbi.nlm.nih.gov/entrez/query.fcgi?cmd=search&db=gene&term=CASZ1) |  |
| 0.0044409 | 0.070963 | 646.934578 | 1127.407403 | 0.573825 | 4120377 | [C2orf47](http://www.ncbi.nlm.nih.gov/entrez/query.fcgi?cmd=search&db=gene&term=C2orf47) |  |
| 0.0005927 | 0.028704 | 299.4990811 | 522.0491534 | 0.573699 | 1850612 | [PARP2](http://www.ncbi.nlm.nih.gov/entrez/query.fcgi?cmd=search&db=gene&term=PARP2) |  |
| 0.0013522 | 0.040636 | 989.0737193 | 1724.844884 | 0.5734276 | 830634 | [USP24](http://www.ncbi.nlm.nih.gov/entrez/query.fcgi?cmd=search&db=gene&term=USP24) |  |
| 0.0004172 | 0.025615 | 183.1350703 | 319.8784824 | 0.5725145 | 1110315 | [FAM36A](http://www.ncbi.nlm.nih.gov/entrez/query.fcgi?cmd=search&db=gene&term=FAM36A) |  |
| 0.0035177 | 0.063698 | 440.1821011 | 772.6500489 | 0.5697044 | 1050386 | [SMUG1](http://www.ncbi.nlm.nih.gov/entrez/query.fcgi?cmd=search&db=gene&term=SMUG1) |  |
| 0.004455 | 0.070963 | 133.5109501 | 234.6151978 | 0.5690635 | 7100039 | [ABCF2](http://www.ncbi.nlm.nih.gov/entrez/query.fcgi?cmd=search&db=gene&term=ABCF2) |  |
| 0.0024309 | 0.053327 | 2701.262031 | 4750.000589 | 0.5686867 | 1030551 | [COPS3](http://www.ncbi.nlm.nih.gov/entrez/query.fcgi?cmd=search&db=gene&term=COPS3) |  |
| 0.0038879 | 0.066543 | 2131.884116 | 3749.718817 | 0.5685451 | 6040475 | [C18orf55](http://www.ncbi.nlm.nih.gov/entrez/query.fcgi?cmd=search&db=gene&term=C18orf55) |  |
| 0.0044769 | 0.071133 | 1804.361164 | 3180.65617 | 0.5672921 | 4900333 | [HAGH](http://www.ncbi.nlm.nih.gov/entrez/query.fcgi?cmd=search&db=gene&term=HAGH) | immunology |
| 0.0041201 | 0.06821 | 552.7792694 | 974.6131672 | 0.5671781 | 1500446 | [DPP8](http://www.ncbi.nlm.nih.gov/entrez/query.fcgi?cmd=search&db=gene&term=DPP8) |  |
| 0.0025278 | 0.054402 | 9116.984342 | 16106.99755 | 0.5660263 | 2750431 | [RYR1](http://www.ncbi.nlm.nih.gov/entrez/query.fcgi?cmd=search&db=gene&term=RYR1) | Calcium signaling pathway, Long-term depression, immunology |
| 0.0012511 | 0.039505 | 260.5617855 | 460.3844908 | 0.5659656 | 4010735 | [MRPL9](http://www.ncbi.nlm.nih.gov/entrez/query.fcgi?cmd=search&db=gene&term=MRPL9) |  |
| 0.0034318 | 0.062934 | 768.2974852 | 1359.116551 | 0.5652918 | 1510468 | [GRPEL1](http://www.ncbi.nlm.nih.gov/entrez/query.fcgi?cmd=search&db=gene&term=GRPEL1) |  |
| 0.0039906 | 0.067466 | 201.4267369 | 356.3307537 | 0.5652802 | 5720327 | [PSMA3](http://www.ncbi.nlm.nih.gov/entrez/query.fcgi?cmd=search&db=gene&term=PSMA3) | Proteasome |
| 0.0020158 | 0.049036 | 163.512723 | 289.4239175 | 0.5649593 | 3290324 | [TTC33](http://www.ncbi.nlm.nih.gov/entrez/query.fcgi?cmd=search&db=gene&term=TTC33) |  |
| 0.0016166 | 0.044302 | 11284.20421 | 20002.27349 | 0.5641461 | 160370 | [TPM2](http://www.ncbi.nlm.nih.gov/entrez/query.fcgi?cmd=search&db=gene&term=TPM2) |  |
| 0.0006441 | 0.029369 | 201.5421115 | 357.5986607 | 0.5635986 | 130064 | [AGA](http://www.ncbi.nlm.nih.gov/entrez/query.fcgi?cmd=search&db=gene&term=AGA) | Glycan structures - degradation, N-Glycan degradation, immunology |
| 0.0008304 | 0.033292 | 675.3513329 | 1198.646072 | 0.5634285 | 1010598 | [ATP5F1](http://www.ncbi.nlm.nih.gov/entrez/query.fcgi?cmd=search&db=gene&term=ATP5F1) | ATP synthesis, Oxidative phosphorylation |
| 0.002019 | 0.049073 | 779.3578208 | 1384.070696 | 0.563091 | 4230431 | [CEBPG](http://www.ncbi.nlm.nih.gov/entrez/query.fcgi?cmd=search&db=gene&term=CEBPG) | gene_regulation, transcription |
| 0.0004521 | 0.026262 | 442.3995255 | 785.8830023 | 0.5629331 | 1070551 | [CSRP2BP](http://www.ncbi.nlm.nih.gov/entrez/query.fcgi?cmd=search&db=gene&term=CSRP2BP) |  |
| 0.001784 | 0.046294 | 1564.354977 | 2781.289749 | 0.5624567 | 6520661 | [C9orf46](http://www.ncbi.nlm.nih.gov/entrez/query.fcgi?cmd=search&db=gene&term=C9orf46) |  |
| 0.0006335 | 0.029309 | 5288.042711 | 9405.137326 | 0.5622505 | 3370195 | [SDHB](http://www.ncbi.nlm.nih.gov/entrez/query.fcgi?cmd=search&db=gene&term=SDHB) | Citrate cycle (TCA cycle), Oxidative phosphorylation |
| 0.0035172 | 0.063698 | 1071.102158 | 1905.062653 | 0.5622399 | 2360630 | [FDFT1](http://www.ncbi.nlm.nih.gov/entrez/query.fcgi?cmd=search&db=gene&term=FDFT1) | Biosynthesis of steroids, Terpenoid biosynthesis |
| 0.0021111 | 0.050014 | 305.869642 | 545.0849497 | 0.5611412 | 5890072 | [NIBP](http://www.ncbi.nlm.nih.gov/entrez/query.fcgi?cmd=search&db=gene&term=NIBP) |  |
| 0.0037809 | 0.065289 | 445.2516579 | 793.6217573 | 0.5610376 | 1230196 | [AHCTF1](http://www.ncbi.nlm.nih.gov/entrez/query.fcgi?cmd=search&db=gene&term=AHCTF1) |  |
| 0.0043116 | 0.06965 | 162.5492802 | 289.8336317 | 0.5608365 | 2120082 | [LOC651302](http://www.ncbi.nlm.nih.gov/entrez/query.fcgi?cmd=search&db=gene&term=LOC651302) |  |
| 0.0038089 | 0.065617 | 3536.825291 | 6316.027055 | 0.5599763 | 4780450 | [ATP5C1](http://www.ncbi.nlm.nih.gov/entrez/query.fcgi?cmd=search&db=gene&term=ATP5C1) | ATP synthesis, Oxidative phosphorylation |
| 0.0020585 | 0.049303 | 122.0950418 | 218.2486494 | 0.5594309 | 3370372 | [C3orf18](http://www.ncbi.nlm.nih.gov/entrez/query.fcgi?cmd=search&db=gene&term=C3orf18) |  |
| 0.0001833 | 0.01994 | 120.7112128 | 216.2654852 | 0.5581622 | 4250500 | [HAT1](http://www.ncbi.nlm.nih.gov/entrez/query.fcgi?cmd=search&db=gene&term=HAT1) |  |
| 3.66E-05 | 0.014558 | 149.4346633 | 267.8013178 | 0.5580057 | 1170121 | [MRPL4](http://www.ncbi.nlm.nih.gov/entrez/query.fcgi?cmd=search&db=gene&term=MRPL4) |  |
| 0.0023688 | 0.052605 | 188.3741211 | 337.6339834 | 0.5579241 | 1090433 | [RNF41](http://www.ncbi.nlm.nih.gov/entrez/query.fcgi?cmd=search&db=gene&term=RNF41) |  |
| 0.0028722 | 0.058256 | 4850.645716 | 8704.380117 | 0.5572649 | 2140408 | [CS](http://www.ncbi.nlm.nih.gov/entrez/query.fcgi?cmd=search&db=gene&term=CS) | Shuttle for transfer of acetyl groups from mitochondria to the cytosol, Citrate cycle (TCA cycle), Glyoxylate and dicarboxylate metabolism |
| 2.22E-05 | 0.013372 | 99.4575165 | 178.5300535 | 0.5570912 | 4200059 |  |  |
| 0.0027723 | 0.057195 | 145.2962901 | 261.2182461 | 0.5562257 | 6770768 | [SYNGR1](http://www.ncbi.nlm.nih.gov/entrez/query.fcgi?cmd=search&db=gene&term=SYNGR1) |  |
| 0.0008541 | 0.033634 | 115.1268642 | 207.2728356 | 0.5554363 | 1110632 | [NKX3-1](http://www.ncbi.nlm.nih.gov/entrez/query.fcgi?cmd=search&db=gene&term=NKX3-1) |  |
| 0.0006098 | 0.028941 | 115.2262311 | 207.7676381 | 0.5545918 | 5360168 | [LOC652324](http://www.ncbi.nlm.nih.gov/entrez/query.fcgi?cmd=search&db=gene&term=LOC652324) |  |
| 0.0003099 | 0.02341 | 135.3640879 | 244.1245565 | 0.5544878 | 1230066 | [LOC650826](http://www.ncbi.nlm.nih.gov/entrez/query.fcgi?cmd=search&db=gene&term=LOC650826) |  |
| 0.0006882 | 0.030043 | 144.583437 | 260.7570722 | 0.5544756 | 2480523 | [DUSP13](http://www.ncbi.nlm.nih.gov/entrez/query.fcgi?cmd=search&db=gene&term=DUSP13) |  |
| 0.0005898 | 0.028704 | 927.684726 | 1673.7217 | 0.5542646 | 5670164 | [RWDD4A](http://www.ncbi.nlm.nih.gov/entrez/query.fcgi?cmd=search&db=gene&term=RWDD4A) |  |
| 0.000488 | 0.027279 | 197.6825965 | 357.3439276 | 0.5531998 | 4220372 | [RNF141](http://www.ncbi.nlm.nih.gov/entrez/query.fcgi?cmd=search&db=gene&term=RNF141) |  |
| 0.0007113 | 0.030455 | 281.7271175 | 509.4550196 | 0.552997 | 5080162 | [KTN1](http://www.ncbi.nlm.nih.gov/entrez/query.fcgi?cmd=search&db=gene&term=KTN1) |  |
| 0.0024649 | 0.053789 | 964.8939202 | 1746.163915 | 0.5525792 | 2490259 | [PINK1](http://www.ncbi.nlm.nih.gov/entrez/query.fcgi?cmd=search&db=gene&term=PINK1) | Neurodegenerative Disorders, Parkinson\'s disease |
| 3.25E-05 | 0.014558 | 181.8448652 | 329.2239067 | 0.552344 | 4200608 | [SPATA2](http://www.ncbi.nlm.nih.gov/entrez/query.fcgi?cmd=search&db=gene&term=SPATA2) |  |
| 0.0019991 | 0.049006 | 242.6811429 | 440.7223393 | 0.5506441 | 2490593 | [COL7A1](http://www.ncbi.nlm.nih.gov/entrez/query.fcgi?cmd=search&db=gene&term=COL7A1) | immunology |
| 0.0028965 | 0.058373 | 290.2684852 | 527.5636839 | 0.5502056 | 1660451 | [PAPD1](http://www.ncbi.nlm.nih.gov/entrez/query.fcgi?cmd=search&db=gene&term=PAPD1) |  |
| 0.0039579 | 0.067148 | 102.7957058 | 186.8954945 | 0.550017 | 70100 | [UGT3A1](http://www.ncbi.nlm.nih.gov/entrez/query.fcgi?cmd=search&db=gene&term=UGT3A1) |  |
| 0.0011341 | 0.037949 | 958.6614856 | 1745.203531 | 0.5493121 | 1850482 | [TNK2](http://www.ncbi.nlm.nih.gov/entrez/query.fcgi?cmd=search&db=gene&term=TNK2) |  |
| 0.0013048 | 0.039956 | 260.7566413 | 474.7563296 | 0.5492431 | 620168 | [ATG4A](http://www.ncbi.nlm.nih.gov/entrez/query.fcgi?cmd=search&db=gene&term=ATG4A) |  |
| 0.0025736 | 0.055004 | 434.8100442 | 792.7452198 | 0.5484865 | 2750544 | [FLJ20323](http://www.ncbi.nlm.nih.gov/entrez/query.fcgi?cmd=search&db=gene&term=FLJ20323) |  |
| 1.73E-05 | 0.012893 | 102.0377435 | 186.0691444 | 0.5483862 | 6660491 | [AES](http://www.ncbi.nlm.nih.gov/entrez/query.fcgi?cmd=search&db=gene&term=AES) | development |
| 0.0013297 | 0.040384 | 109.9192322 | 200.5384407 | 0.5481205 | 20382 | [RNF157](http://www.ncbi.nlm.nih.gov/entrez/query.fcgi?cmd=search&db=gene&term=RNF157) |  |
| 0.0010036 | 0.036166 | 772.4681156 | 1409.550331 | 0.5480245 | 4280204 | [PHB](http://www.ncbi.nlm.nih.gov/entrez/query.fcgi?cmd=search&db=gene&term=PHB) | immunology |
| 0.0010236 | 0.03649 | 247.5735609 | 451.8970116 | 0.5478539 | 4200577 | [GTF2IRD1](http://www.ncbi.nlm.nih.gov/entrez/query.fcgi?cmd=search&db=gene&term=GTF2IRD1) | Basal transcription factors |
| 0.0009056 | 0.034003 | 293.6784618 | 536.3163062 | 0.5475844 | 1500470 | [MED25](http://www.ncbi.nlm.nih.gov/entrez/query.fcgi?cmd=search&db=gene&term=MED25) |  |
| 6.70E-06 | 0.012163 | 299.7702382 | 547.6503287 | 0.5473753 | 4200152 | [IFRD2](http://www.ncbi.nlm.nih.gov/entrez/query.fcgi?cmd=search&db=gene&term=IFRD2) |  |
| 0.0005614 | 0.028358 | 1352.422747 | 2473.68635 | 0.5467236 | 160639 | [EBAG9](http://www.ncbi.nlm.nih.gov/entrez/query.fcgi?cmd=search&db=gene&term=EBAG9) |  |
| 0.0004132 | 0.025615 | 488.9427524 | 894.6964361 | 0.5464901 | 6760291 | [RNF10](http://www.ncbi.nlm.nih.gov/entrez/query.fcgi?cmd=search&db=gene&term=RNF10) |  |
| 0.0004174 | 0.025615 | 324.7654986 | 594.7408587 | 0.5460622 | 670561 | [DHRS7B](http://www.ncbi.nlm.nih.gov/entrez/query.fcgi?cmd=search&db=gene&term=DHRS7B) |  |
| 1.81E-05 | 0.012893 | 139.1543127 | 254.8393625 | 0.5460472 | 4200209 | [RBKS](http://www.ncbi.nlm.nih.gov/entrez/query.fcgi?cmd=search&db=gene&term=RBKS) |  |
| 0.0005755 | 0.028704 | 805.4028891 | 1475.184325 | 0.5459676 | 6840112 | [ZNF25](http://www.ncbi.nlm.nih.gov/entrez/query.fcgi?cmd=search&db=gene&term=ZNF25) |  |
| 0.0011048 | 0.037575 | 182.5047743 | 334.8003997 | 0.5451152 | 50056 | [MRPS18B](http://www.ncbi.nlm.nih.gov/entrez/query.fcgi?cmd=search&db=gene&term=MRPS18B) |  |
| 0.0040855 | 0.068002 | 135.8623696 | 249.2817273 | 0.5450154 | 2640224 | [VEGFA](http://www.ncbi.nlm.nih.gov/entrez/query.fcgi?cmd=search&db=gene&term=VEGFA) | Actions of Nitric Oxide in the Heart, Hypoxia-Inducible Factor in the Cardiovascular System, VEGF, Hypoxia, and Angiogenesis, Cytokine-cytokine receptor interaction, Focal adhesion, mTOR signaling pathway, VEGF signaling pathway |
| 0.0016168 | 0.044302 | 130.8867226 | 240.2892778 | 0.5447048 | 4780041 | [ZC3H6](http://www.ncbi.nlm.nih.gov/entrez/query.fcgi?cmd=search&db=gene&term=ZC3H6) |  |
| 0.0003595 | 0.024601 | 10054.54296 | 18507.99356 | 0.5432541 | 4900670 | [COX5A](http://www.ncbi.nlm.nih.gov/entrez/query.fcgi?cmd=search&db=gene&term=COX5A) | Oxidative phosphorylation, misc |
| 0.0019288 | 0.048229 | 143.4276817 | 264.0606971 | 0.5431618 | 5310537 | [MRPL16](http://www.ncbi.nlm.nih.gov/entrez/query.fcgi?cmd=search&db=gene&term=MRPL16) |  |
| 0.0028914 | 0.058373 | 170.9287597 | 314.7335105 | 0.5430904 | 5720192 | [FANCE](http://www.ncbi.nlm.nih.gov/entrez/query.fcgi?cmd=search&db=gene&term=FANCE) | BRCA1-dependent Ub-ligase activity, Role of BRCA1, BRCA2 and ATR in Cancer Susceptibility, immunology |
| 0.0029224 | 0.058389 | 419.1704246 | 772.9934769 | 0.542269 | 4850133 | [SNRPN](http://www.ncbi.nlm.nih.gov/entrez/query.fcgi?cmd=search&db=gene&term=SNRPN) | immunology |
| 0.0006794 | 0.030043 | 108.5176114 | 200.3918628 | 0.541527 | 2680541 | [MRPL19](http://www.ncbi.nlm.nih.gov/entrez/query.fcgi?cmd=search&db=gene&term=MRPL19) |  |
| 0.0014147 | 0.041638 | 398.6008177 | 736.2857653 | 0.541367 | 4250725 |  |  |
| 0.0008045 | 0.03259 | 331.8528058 | 613.3762354 | 0.5410265 | 7650615 | [GRHPR](http://www.ncbi.nlm.nih.gov/entrez/query.fcgi?cmd=search&db=gene&term=GRHPR) |  |
| 0.001072 | 0.037067 | 2312.070499 | 4273.50459 | 0.5410245 | 3290246 | [IDH3B](http://www.ncbi.nlm.nih.gov/entrez/query.fcgi?cmd=search&db=gene&term=IDH3B) | Citrate cycle (TCA cycle) |
| 0.0020674 | 0.049303 | 149.7871119 | 276.9576267 | 0.5408304 | 360706 | [GRTP1](http://www.ncbi.nlm.nih.gov/entrez/query.fcgi?cmd=search&db=gene&term=GRTP1) |  |
| 0.0035839 | 0.063979 | 518.3253068 | 958.5060807 | 0.5407637 | 6450681 | [DLST](http://www.ncbi.nlm.nih.gov/entrez/query.fcgi?cmd=search&db=gene&term=DLST) | Citrate cycle (TCA cycle), Lysine degradation |
| 0.0001963 | 0.020451 | 290.792497 | 538.0940857 | 0.540412 | 2480044 | [MTX2](http://www.ncbi.nlm.nih.gov/entrez/query.fcgi?cmd=search&db=gene&term=MTX2) |  |
| 0.003194 | 0.060634 | 524.0305494 | 972.1815659 | 0.5390254 | 6560750 | [UBE3C](http://www.ncbi.nlm.nih.gov/entrez/query.fcgi?cmd=search&db=gene&term=UBE3C) | Tryptophan metabolism |
| 0.0011903 | 0.038645 | 1931.045414 | 3584.671139 | 0.5386953 | 2260471 | [ABLIM2](http://www.ncbi.nlm.nih.gov/entrez/query.fcgi?cmd=search&db=gene&term=ABLIM2) | Axon guidance |
| 0.0001103 | 0.018033 | 4696.482915 | 8720.821613 | 0.5385367 | 4860563 | [NDUFAB1](http://www.ncbi.nlm.nih.gov/entrez/query.fcgi?cmd=search&db=gene&term=NDUFAB1) | Oxidative phosphorylation |
| 0.0006474 | 0.029394 | 175.2007624 | 325.3630781 | 0.5384777 | 2060564 | [MNAT1](http://www.ncbi.nlm.nih.gov/entrez/query.fcgi?cmd=search&db=gene&term=MNAT1) |  |
| 0.0021542 | 0.050377 | 240.0310634 | 446.0491899 | 0.5381269 | 1580356 | [TMED1](http://www.ncbi.nlm.nih.gov/entrez/query.fcgi?cmd=search&db=gene&term=TMED1) |  |
| 0.0002802 | 0.022708 | 164.8893102 | 307.153741 | 0.5368299 | 380279 | [SPTB](http://www.ncbi.nlm.nih.gov/entrez/query.fcgi?cmd=search&db=gene&term=SPTB) | cell_signaling, metastasis |
| 0.0005336 | 0.027911 | 148.5147365 | 276.744856 | 0.5366486 | 2690521 |  |  |
| 0.003492 | 0.06355 | 1855.133372 | 3457.428894 | 0.5365644 | 6900450 | [QDPR](http://www.ncbi.nlm.nih.gov/entrez/query.fcgi?cmd=search&db=gene&term=QDPR) | Folate biosynthesis, immunology, metabolism |
| 0.0046634 | 0.072471 | 185.5854981 | 346.0347147 | 0.5363205 | 4070379 | [PCYT2](http://www.ncbi.nlm.nih.gov/entrez/query.fcgi?cmd=search&db=gene&term=PCYT2) | Aminophosphonate metabolism, Glycerophospholipid metabolism, immunology |
| 0.002535 | 0.0545 | 194.8129733 | 363.6374446 | 0.5357341 | 5340332 | [LOC729101](http://www.ncbi.nlm.nih.gov/entrez/query.fcgi?cmd=search&db=gene&term=LOC729101) |  |
| 0.0020638 | 0.049303 | 130.6742593 | 244.2256387 | 0.5350555 | 1090129 | [NMNAT1](http://www.ncbi.nlm.nih.gov/entrez/query.fcgi?cmd=search&db=gene&term=NMNAT1) | Nicotinate and nicotinamide metabolism |
| 0.0006859 | 0.030043 | 2031.066626 | 3796.81748 | 0.5349392 | 6550064 | [PRDX2](http://www.ncbi.nlm.nih.gov/entrez/query.fcgi?cmd=search&db=gene&term=PRDX2) |  |
| 0.0033186 | 0.061791 | 304.0344727 | 568.4641806 | 0.5348349 | 450161 | [FAHD1](http://www.ncbi.nlm.nih.gov/entrez/query.fcgi?cmd=search&db=gene&term=FAHD1) |  |
| 0.0008732 | 0.033692 | 271.2268626 | 507.8656837 | 0.5340524 | 6660497 | [HSPA14](http://www.ncbi.nlm.nih.gov/entrez/query.fcgi?cmd=search&db=gene&term=HSPA14) |  |
| 0.003174 | 0.060611 | 2022.838708 | 3788.097046 | 0.5339986 | 2230156 | [C15orf52](http://www.ncbi.nlm.nih.gov/entrez/query.fcgi?cmd=search&db=gene&term=C15orf52) |  |
| 0.0003248 | 0.023725 | 123.9073814 | 232.287763 | 0.5334219 | 5900343 | [CNKSR1](http://www.ncbi.nlm.nih.gov/entrez/query.fcgi?cmd=search&db=gene&term=CNKSR1) |  |
| 0.0005328 | 0.027911 | 1056.021739 | 1979.734555 | 0.5334158 | 5090687 | [C7orf44](http://www.ncbi.nlm.nih.gov/entrez/query.fcgi?cmd=search&db=gene&term=C7orf44) |  |
| 2.37E-05 | 0.013372 | 147.7773376 | 277.8371824 | 0.5318847 | 5550608 | [IKZF4](http://www.ncbi.nlm.nih.gov/entrez/query.fcgi?cmd=search&db=gene&term=IKZF4) |  |
| 0.0005979 | 0.028704 | 214.6738531 | 403.9284309 | 0.5314651 | 3290341 | [AIFM1](http://www.ncbi.nlm.nih.gov/entrez/query.fcgi?cmd=search&db=gene&term=AIFM1) | Ceramide Signaling Pathway, Opposing roles of AIF in Apoptosis and Cell Survival, Role of Mitochondria in Apoptotic Signaling, Apoptosis |
| 0.0003669 | 0.024601 | 178.8490078 | 336.6121024 | 0.5313208 | 1660646 | [RIF1](http://www.ncbi.nlm.nih.gov/entrez/query.fcgi?cmd=search&db=gene&term=RIF1) |  |
| 0.0008298 | 0.033292 | 125.7833998 | 236.8003799 | 0.531179 | 3710240 | [CELSR2](http://www.ncbi.nlm.nih.gov/entrez/query.fcgi?cmd=search&db=gene&term=CELSR2) |  |
| 0.0008782 | 0.033734 | 200.5280182 | 378.1990973 | 0.5302181 | 730139 | [MRPL48](http://www.ncbi.nlm.nih.gov/entrez/query.fcgi?cmd=search&db=gene&term=MRPL48) |  |
| 0.0019638 | 0.048668 | 1201.154894 | 2265.73946 | 0.5301381 | 7330678 | [STK25](http://www.ncbi.nlm.nih.gov/entrez/query.fcgi?cmd=search&db=gene&term=STK25) |  |
| 0.0013787 | 0.041029 | 2084.503585 | 3933.049735 | 0.5299968 | 3930286 | [SUCLA2](http://www.ncbi.nlm.nih.gov/entrez/query.fcgi?cmd=search&db=gene&term=SUCLA2) | C5-Branched dibasic acid metabolism, Citrate cycle (TCA cycle), Propanoate metabolism, Reductive carboxylate cycle (CO2 fixation) |
| 0.0005043 | 0.02772 | 6197.098999 | 11699.77607 | 0.5296767 | 4050681 | [NDUFV2](http://www.ncbi.nlm.nih.gov/entrez/query.fcgi?cmd=search&db=gene&term=NDUFV2) | Oxidative phosphorylation |
| 0.0016573 | 0.044903 | 310.6244498 | 586.5317622 | 0.5295953 | 7650379 | [C9orf32](http://www.ncbi.nlm.nih.gov/entrez/query.fcgi?cmd=search&db=gene&term=C9orf32) |  |
| 0.0041936 | 0.068854 | 825.754022 | 1561.885181 | 0.5286906 | 1340128 | [HSDL2](http://www.ncbi.nlm.nih.gov/entrez/query.fcgi?cmd=search&db=gene&term=HSDL2) |  |
| 0.00015 | 0.019278 | 222.5887099 | 421.3898586 | 0.5282251 | 3460068 | [TRIM54](http://www.ncbi.nlm.nih.gov/entrez/query.fcgi?cmd=search&db=gene&term=TRIM54) |  |
| 0.0011278 | 0.037826 | 141.0793922 | 267.0960452 | 0.5281972 | 5130487 | [SRRD](http://www.ncbi.nlm.nih.gov/entrez/query.fcgi?cmd=search&db=gene&term=SRRD) |  |
| 0.0021037 | 0.049924 | 639.066526 | 1211.393138 | 0.5275468 | 4200672 | [STRAP](http://www.ncbi.nlm.nih.gov/entrez/query.fcgi?cmd=search&db=gene&term=STRAP) |  |
| 0.0011962 | 0.038713 | 665.6770223 | 1262.699966 | 0.5271854 | 4150630 | [NKIRAS1](http://www.ncbi.nlm.nih.gov/entrez/query.fcgi?cmd=search&db=gene&term=NKIRAS1) |  |
| 0.0001571 | 0.019693 | 423.2443626 | 803.3250846 | 0.5268656 | 1030348 | [CDKN1B](http://www.ncbi.nlm.nih.gov/entrez/query.fcgi?cmd=search&db=gene&term=CDKN1B) | CDK Regulation of DNA Replication, Cell Cycle: G1/S Check Point , CTCF: First Multivalent Nuclear Factor, Cyclins and Cell Cycle Regulation, Influence of Ras and Rho proteins on G1 to S Transition, PTEN dependent cell cycle arrest and apoptosis, Regulation of p27 Phosphorylation during Cell Cycle Progression, Cell cycle, cell_cycle, immunology |
| 0.000319 | 0.023516 | 154.6738348 | 293.6445391 | 0.5267383 | 4230377 | [PDK2](http://www.ncbi.nlm.nih.gov/entrez/query.fcgi?cmd=search&db=gene&term=PDK2) | mTOR Signaling Pathway, PTEN dependent cell cycle arrest and apoptosis, Regulation of eIF4e and p70 S6 Kinase, Skeletal muscle hypertrophy is regulated via AKT/mTOR pathway, cell_cycle, cell_signaling, signal_transduction |
| 0.0048244 | 0.073325 | 3760.607421 | 7142.887242 | 0.5264828 | 5550377 | [NDUFB11](http://www.ncbi.nlm.nih.gov/entrez/query.fcgi?cmd=search&db=gene&term=NDUFB11) | Oxidative phosphorylation, Ubiquinone biosynthesis |
| 0.0004145 | 0.025615 | 2053.662989 | 3906.636198 | 0.5256858 | 4860358 | [VBP1](http://www.ncbi.nlm.nih.gov/entrez/query.fcgi?cmd=search&db=gene&term=VBP1) |  |
| 0.003775 | 0.065289 | 1159.417105 | 2206.833178 | 0.525376 | 2760121 | [ADHFE1](http://www.ncbi.nlm.nih.gov/entrez/query.fcgi?cmd=search&db=gene&term=ADHFE1) | 1- and 2-Methylnaphthalene degradation, Bile acid biosynthesis, Fatty acid metabolism, Glycerolipid metabolism, Glycolysis / Gluconeogenesis, Metabolism of xenobiotics by cytochrome P450, Tyrosine metabolism |
| 0.0031877 | 0.060634 | 838.0424795 | 1598.186723 | 0.5243708 | 6020692 |  |  |
| 0.0043116 | 0.06965 | 454.6038768 | 868.3207476 | 0.5235437 | 5570376 | [FAM50B](http://www.ncbi.nlm.nih.gov/entrez/query.fcgi?cmd=search&db=gene&term=FAM50B) |  |
| 0.0004486 | 0.026228 | 370.1784432 | 707.4223755 | 0.5232778 | 6330279 | [ATP6V1H](http://www.ncbi.nlm.nih.gov/entrez/query.fcgi?cmd=search&db=gene&term=ATP6V1H) | ATP synthesis, Epithelial cell signaling in Helicobacter pylori infection, Oxidative phosphorylation |
| 0.0007385 | 0.03099 | 256.2321343 | 489.7184224 | 0.5232234 | 1430152 | [SCML1](http://www.ncbi.nlm.nih.gov/entrez/query.fcgi?cmd=search&db=gene&term=SCML1) |  |
| 0.004646 | 0.072317 | 1141.023406 | 2182.86026 | 0.5227194 | 6620201 | [KLHL24](http://www.ncbi.nlm.nih.gov/entrez/query.fcgi?cmd=search&db=gene&term=KLHL24) |  |
| 0.0033357 | 0.061973 | 213.4728228 | 408.9155006 | 0.5220463 | 60196 | [GCSH](http://www.ncbi.nlm.nih.gov/entrez/query.fcgi?cmd=search&db=gene&term=GCSH) |  |
| 1.62E-05 | 0.012893 | 196.9570165 | 377.3880818 | 0.5218952 | 6860681 | [IER3IP1](http://www.ncbi.nlm.nih.gov/entrez/query.fcgi?cmd=search&db=gene&term=IER3IP1) |  |
| 0.0014958 | 0.042622 | 138.3733198 | 265.2763739 | 0.5216195 | 2000433 | [KCNJ11](http://www.ncbi.nlm.nih.gov/entrez/query.fcgi?cmd=search&db=gene&term=KCNJ11) | Type II diabetes mellitus |
| 0.0025439 | 0.05461 | 205.0371474 | 393.2018556 | 0.5214552 | 6550187 | [COL20A1](http://www.ncbi.nlm.nih.gov/entrez/query.fcgi?cmd=search&db=gene&term=COL20A1) |  |
| 0.003025 | 0.059206 | 234.5798207 | 450.224814 | 0.5210282 | 60707 | [DPP8](http://www.ncbi.nlm.nih.gov/entrez/query.fcgi?cmd=search&db=gene&term=DPP8) |  |
| 0.000406 | 0.025524 | 708.7512297 | 1360.681386 | 0.5208796 | 6940092 | [COPS8](http://www.ncbi.nlm.nih.gov/entrez/query.fcgi?cmd=search&db=gene&term=COPS8) |  |
| 0.003552 | 0.063738 | 237.8266606 | 457.1644178 | 0.5202213 | 6770494 | [HAX1](http://www.ncbi.nlm.nih.gov/entrez/query.fcgi?cmd=search&db=gene&term=HAX1) |  |
| 0.0024065 | 0.053106 | 132.6620048 | 255.3772689 | 0.5194746 | 2900646 | [LDB3](http://www.ncbi.nlm.nih.gov/entrez/query.fcgi?cmd=search&db=gene&term=LDB3) |  |
| 0.0042172 | 0.068969 | 220.1499812 | 424.5002664 | 0.5186098 | 2850301 | [AGT](http://www.ncbi.nlm.nih.gov/entrez/query.fcgi?cmd=search&db=gene&term=AGT) | Angiotensin II mediated activation of JNK Pathway via Pyk2 dependent signaling, Angiotensin-converting enzyme 2 regulates heart function, Bioactive Peptide Induced Signaling Pathway, NFAT and Hypertrophy of the heart (Transcription in the broken heart), Role of EGF Receptor Transactivation by GPCRs in Cardiac Hypertrophy, immunology |
| 0.0049276 | 0.074081 | 1160.852918 | 2240.274219 | 0.5181745 | 4670424 | [MRPL11](http://www.ncbi.nlm.nih.gov/entrez/query.fcgi?cmd=search&db=gene&term=MRPL11) | Mechanism of Gene Regulation by Peroxisome Proliferators via PPARa(alpha) |
| 0.0009007 | 0.034003 | 421.5578602 | 814.0442633 | 0.5178562 | 7200356 | [POLDIP2](http://www.ncbi.nlm.nih.gov/entrez/query.fcgi?cmd=search&db=gene&term=POLDIP2) |  |
| 0.0013603 | 0.040774 | 238.3952484 | 460.6801672 | 0.5174854 | 6960382 | [ING2](http://www.ncbi.nlm.nih.gov/entrez/query.fcgi?cmd=search&db=gene&term=ING2) |  |
| 0.003941 | 0.067018 | 630.8190301 | 1220.545971 | 0.5168335 | 6450647 | [MRPS28](http://www.ncbi.nlm.nih.gov/entrez/query.fcgi?cmd=search&db=gene&term=MRPS28) |  |
| 0.0009029 | 0.034003 | 2431.166838 | 4707.48277 | 0.5164473 | 510228 | [IDH3B](http://www.ncbi.nlm.nih.gov/entrez/query.fcgi?cmd=search&db=gene&term=IDH3B) | Citrate cycle (TCA cycle) |
| 0.0029311 | 0.058391 | 327.6154891 | 634.693605 | 0.516179 | 5820563 | [CDH13](http://www.ncbi.nlm.nih.gov/entrez/query.fcgi?cmd=search&db=gene&term=CDH13) | cell_signaling, metastasis |
| 0.0019585 | 0.048578 | 621.4332658 | 1204.489781 | 0.5159307 | 5690553 | [HADHA](http://www.ncbi.nlm.nih.gov/entrez/query.fcgi?cmd=search&db=gene&term=HADHA) | Benzoate degradation via CoA ligation, beta-Alanine metabolism, Butanoate metabolism, Caprolactam degradation, Fatty acid elongation in mitochondria, Fatty acid metabolism, Limonene and pinene degradation, Lysine degradation, Propanoate metabolism, Tryptophan metabolism, Valine, leucine and isoleucine degradation, immunology |
| 0.0020696 | 0.049312 | 188.9481541 | 366.3290709 | 0.515788 | 6020400 | [CNNM4](http://www.ncbi.nlm.nih.gov/entrez/query.fcgi?cmd=search&db=gene&term=CNNM4) |  |
| 7.59E-05 | 0.016325 | 833.5908155 | 1616.717999 | 0.5156068 | 5290215 | [CLASP1](http://www.ncbi.nlm.nih.gov/entrez/query.fcgi?cmd=search&db=gene&term=CLASP1) |  |
| 0.0009256 | 0.034346 | 1002.887787 | 1949.252105 | 0.5144988 | 6450458 | [DHPS](http://www.ncbi.nlm.nih.gov/entrez/query.fcgi?cmd=search&db=gene&term=DHPS) |  |
| 0.0011707 | 0.038552 | 181.8673001 | 354.0750172 | 0.5136406 | 7040025 | [DUPD1](http://www.ncbi.nlm.nih.gov/entrez/query.fcgi?cmd=search&db=gene&term=DUPD1) |  |
| 0.0011609 | 0.038404 | 407.4796502 | 794.8794635 | 0.5126307 | 3890598 | [ADHFE1](http://www.ncbi.nlm.nih.gov/entrez/query.fcgi?cmd=search&db=gene&term=ADHFE1) | 1- and 2-Methylnaphthalene degradation, Bile acid biosynthesis, Fatty acid metabolism, Glycerolipid metabolism, Glycolysis / Gluconeogenesis, Metabolism of xenobiotics by cytochrome P450, Tyrosine metabolism |
| 0.0033922 | 0.062509 | 615.9315074 | 1201.680444 | 0.5125585 | 3180600 | [PNPO](http://www.ncbi.nlm.nih.gov/entrez/query.fcgi?cmd=search&db=gene&term=PNPO) | Vitamin B6 metabolism |
| 0.0046099 | 0.072259 | 396.7182953 | 774.2246335 | 0.5124072 | 2350754 | [FBXO34](http://www.ncbi.nlm.nih.gov/entrez/query.fcgi?cmd=search&db=gene&term=FBXO34) |  |
| 0.0022708 | 0.051893 | 142.8321458 | 278.8289936 | 0.5122572 | 6200243 | [LOC283871](http://www.ncbi.nlm.nih.gov/entrez/query.fcgi?cmd=search&db=gene&term=LOC283871) |  |
| 0.0009105 | 0.03403 | 207.4197505 | 406.0398499 | 0.5108359 | 4570131 | [SIRT4](http://www.ncbi.nlm.nih.gov/entrez/query.fcgi?cmd=search&db=gene&term=SIRT4) |  |
| 0.0002033 | 0.020451 | 1704.23614 | 3337.39257 | 0.510649 | 1510014 | [PCNT](http://www.ncbi.nlm.nih.gov/entrez/query.fcgi?cmd=search&db=gene&term=PCNT) | Protein Kinase A at the Centrosome, immunology |
| 0.0004497 | 0.026228 | 335.6694692 | 658.021616 | 0.5101192 | 3420068 | [HAT1](http://www.ncbi.nlm.nih.gov/entrez/query.fcgi?cmd=search&db=gene&term=HAT1) |  |
| 0.0003999 | 0.025361 | 410.6627102 | 805.5527739 | 0.50979 | 7380270 | [MRPS35](http://www.ncbi.nlm.nih.gov/entrez/query.fcgi?cmd=search&db=gene&term=MRPS35) |  |
| 0.0026331 | 0.055555 | 6452.479688 | 12658.025 | 0.5097541 | 3830315 | [CKMT2](http://www.ncbi.nlm.nih.gov/entrez/query.fcgi?cmd=search&db=gene&term=CKMT2) | Arginine and proline metabolism, Urea cycle and metabolism of amino groups |
| 0.0002129 | 0.020598 | 151.1884475 | 296.8819055 | 0.5092545 | 1770392 | [MGC26718](http://www.ncbi.nlm.nih.gov/entrez/query.fcgi?cmd=search&db=gene&term=MGC26718) |  |
| 0.0046809 | 0.072588 | 540.5453558 | 1061.70723 | 0.5091284 | 2100576 | [ARAF](http://www.ncbi.nlm.nih.gov/entrez/query.fcgi?cmd=search&db=gene&term=ARAF) | MAPKinase Signaling Pathway |
| 0.0021282 | 0.050133 | 781.2811829 | 1535.441009 | 0.5088318 | 6450050 | [NDUFB6](http://www.ncbi.nlm.nih.gov/entrez/query.fcgi?cmd=search&db=gene&term=NDUFB6) | Oxidative phosphorylation |
| 0.001911 | 0.048015 | 2872.596947 | 5652.619213 | 0.5081887 | 4560367 | [NDUFA8](http://www.ncbi.nlm.nih.gov/entrez/query.fcgi?cmd=search&db=gene&term=NDUFA8) | Oxidative phosphorylation |
| 1.34E-05 | 0.012163 | 109.4646651 | 215.4576331 | 0.5080566 | 2480725 | [PCTK1](http://www.ncbi.nlm.nih.gov/entrez/query.fcgi?cmd=search&db=gene&term=PCTK1) | immunology |
| 0.0016283 | 0.044449 | 1091.438968 | 2149.018312 | 0.5078779 | 2750598 | [ANKRD46](http://www.ncbi.nlm.nih.gov/entrez/query.fcgi?cmd=search&db=gene&term=ANKRD46) |  |
| 0.0007394 | 0.03099 | 180.2165993 | 354.9020843 | 0.5077925 | 6220685 | [C3orf1](http://www.ncbi.nlm.nih.gov/entrez/query.fcgi?cmd=search&db=gene&term=C3orf1) |  |
| 3.31E-05 | 0.014558 | 248.5261232 | 489.838376 | 0.5073635 | 450070 | [C18orf21](http://www.ncbi.nlm.nih.gov/entrez/query.fcgi?cmd=search&db=gene&term=C18orf21) |  |
| 0.002422 | 0.053326 | 489.8291478 | 966.1418441 | 0.5069951 | 6040088 | [ACN9](http://www.ncbi.nlm.nih.gov/entrez/query.fcgi?cmd=search&db=gene&term=ACN9) |  |
| 0.003531 | 0.063701 | 1137.646507 | 2245.217737 | 0.5066976 | 5550253 | [MFN2](http://www.ncbi.nlm.nih.gov/entrez/query.fcgi?cmd=search&db=gene&term=MFN2) |  |
| 0.0022067 | 0.050949 | 131.9449648 | 260.5432057 | 0.5064226 | 4290170 | [PRKAR2A](http://www.ncbi.nlm.nih.gov/entrez/query.fcgi?cmd=search&db=gene&term=PRKAR2A) | AKAP95 role in mitosis and chromosome dynamics, Protein Kinase A at the Centrosome, Rho-Selective Guanine Exchange Factor AKAP13 Mediates Stress Fiber Formation, Apoptosis, Insulin signaling pathway |
| 0.0027904 | 0.057371 | 683.939135 | 1350.821645 | 0.5063134 | 670711 | [PLCL2](http://www.ncbi.nlm.nih.gov/entrez/query.fcgi?cmd=search&db=gene&term=PLCL2) |  |
| 0.0048878 | 0.073787 | 1260.413942 | 2497.821106 | 0.5046054 | 2940075 | [ALS2CR2](http://www.ncbi.nlm.nih.gov/entrez/query.fcgi?cmd=search&db=gene&term=ALS2CR2) |  |
| 0.0029847 | 0.058853 | 2572.364477 | 5114.309326 | 0.502974 | 4890093 | [BRP44L](http://www.ncbi.nlm.nih.gov/entrez/query.fcgi?cmd=search&db=gene&term=BRP44L) |  |
| 0.0010062 | 0.036215 | 168.0384698 | 334.276735 | 0.5026927 | 3310440 | [COQ7](http://www.ncbi.nlm.nih.gov/entrez/query.fcgi?cmd=search&db=gene&term=COQ7) | Ubiquinone biosynthesis |
| 0.0005094 | 0.02772 | 263.9415473 | 525.2170232 | 0.5025381 | 7210093 | [PGM2L1](http://www.ncbi.nlm.nih.gov/entrez/query.fcgi?cmd=search&db=gene&term=PGM2L1) |  |
| 0.0019712 | 0.048685 | 157.0196425 | 312.5682965 | 0.5023531 | 2470102 | [C7orf10](http://www.ncbi.nlm.nih.gov/entrez/query.fcgi?cmd=search&db=gene&term=C7orf10) |  |
| 0.0012187 | 0.039002 | 471.1590058 | 940.0483853 | 0.5012072 | 6200647 | [IBTK](http://www.ncbi.nlm.nih.gov/entrez/query.fcgi?cmd=search&db=gene&term=IBTK) |  |
| 0.0015973 | 0.044064 | 637.226153 | 1273.029134 | 0.500559 | 5340504 | [AKAP7](http://www.ncbi.nlm.nih.gov/entrez/query.fcgi?cmd=search&db=gene&term=AKAP7) |  |
| 0.0045014 | 0.071346 | 158.9580758 | 319.3606874 | 0.4977384 | 5690041 | [OSBPL11](http://www.ncbi.nlm.nih.gov/entrez/query.fcgi?cmd=search&db=gene&term=OSBPL11) |  |
| 0.0011859 | 0.038641 | 344.4369945 | 692.0807523 | 0.4976832 | 5860156 | [C22orf39](http://www.ncbi.nlm.nih.gov/entrez/query.fcgi?cmd=search&db=gene&term=C22orf39) |  |
| 0.0017324 | 0.045557 | 183.1950346 | 368.3551502 | 0.4973326 | 6620176 | [GTPBP8](http://www.ncbi.nlm.nih.gov/entrez/query.fcgi?cmd=search&db=gene&term=GTPBP8) |  |
| 0.0026137 | 0.055412 | 166.8533258 | 335.6624797 | 0.4970866 | 5290050 | [HOMER1](http://www.ncbi.nlm.nih.gov/entrez/query.fcgi?cmd=search&db=gene&term=HOMER1) |  |
| 0.0010152 | 0.036328 | 240.4739804 | 484.3404776 | 0.4964978 | 3140524 | [NARG1](http://www.ncbi.nlm.nih.gov/entrez/query.fcgi?cmd=search&db=gene&term=NARG1) |  |
| 2.40E-05 | 0.013372 | 230.697475 | 464.7297165 | 0.4964121 | 5260639 | [ZNF330](http://www.ncbi.nlm.nih.gov/entrez/query.fcgi?cmd=search&db=gene&term=ZNF330) |  |
| 0.0012628 | 0.039566 | 2155.450004 | 4347.83365 | 0.4957526 | 3390292 | [KLF9](http://www.ncbi.nlm.nih.gov/entrez/query.fcgi?cmd=search&db=gene&term=KLF9) |  |
| 0.002066 | 0.049303 | 211.8834122 | 427.671514 | 0.4954349 | 2710291 |  |  |
| 0.0020433 | 0.049303 | 1681.864292 | 3397.056267 | 0.4950946 | 7570500 | [COQ5](http://www.ncbi.nlm.nih.gov/entrez/query.fcgi?cmd=search&db=gene&term=COQ5) | Ubiquinone biosynthesis |
| 0.0005009 | 0.027607 | 261.0243826 | 527.3360066 | 0.4949868 | 6330653 | [FLJ45244](http://www.ncbi.nlm.nih.gov/entrez/query.fcgi?cmd=search&db=gene&term=FLJ45244) |  |
| 0.0003128 | 0.023475 | 127.1695246 | 256.9521573 | 0.4949152 | 4890255 | [AGMAT](http://www.ncbi.nlm.nih.gov/entrez/query.fcgi?cmd=search&db=gene&term=AGMAT) | Arginine and proline metabolism |
| 0.004732 | 0.07272 | 237.2693883 | 480.217013 | 0.4940878 | 6590053 | [ARD1A](http://www.ncbi.nlm.nih.gov/entrez/query.fcgi?cmd=search&db=gene&term=ARD1A) | 1- and 2-Methylnaphthalene degradation, Alkaloid biosynthesis II, Benzoate degradation via CoA ligation, Ethylbenzene degradation, Glycerophospholipid metabolism, Limonene and pinene degradation, Phenylalanine metabolism, Tyrosine metabolism, Valine, leucine and isoleucine degradation |
| 0.0004385 | 0.026228 | 431.3438591 | 873.1432808 | 0.4940127 | 3890192 | [DDX46](http://www.ncbi.nlm.nih.gov/entrez/query.fcgi?cmd=search&db=gene&term=DDX46) |  |
| 0.000318 | 0.023516 | 1121.579512 | 2276.442024 | 0.4926897 | 5260632 | [UBE2D4](http://www.ncbi.nlm.nih.gov/entrez/query.fcgi?cmd=search&db=gene&term=UBE2D4) | Ubiquitin mediated proteolysis |
| 0.0014859 | 0.042622 | 184.8020732 | 375.1006907 | 0.4926732 | 5690131 | [CCDC101](http://www.ncbi.nlm.nih.gov/entrez/query.fcgi?cmd=search&db=gene&term=CCDC101) |  |
| 0.0026231 | 0.05549 | 553.1397699 | 1124.480297 | 0.491907 | 4230528 | [BTBD1](http://www.ncbi.nlm.nih.gov/entrez/query.fcgi?cmd=search&db=gene&term=BTBD1) |  |
| 0.0003633 | 0.024601 | 652.4434416 | 1328.056538 | 0.4912769 | 730092 | [SFXN4](http://www.ncbi.nlm.nih.gov/entrez/query.fcgi?cmd=search&db=gene&term=SFXN4) |  |
| 0.0041507 | 0.068459 | 519.5808583 | 1059.87774 | 0.4902272 | 50170 | [SBDS](http://www.ncbi.nlm.nih.gov/entrez/query.fcgi?cmd=search&db=gene&term=SBDS) |  |
| 0.0034453 | 0.063016 | 124.3707045 | 253.9610227 | 0.4897236 | 7380717 | [ZBTB16](http://www.ncbi.nlm.nih.gov/entrez/query.fcgi?cmd=search&db=gene&term=ZBTB16) | Map Kinase Inactivation of SMRT Corepressor |
| 0.0012547 | 0.039505 | 787.8959011 | 1609.228781 | 0.4896109 | 6100703 | [RAF1](http://www.ncbi.nlm.nih.gov/entrez/query.fcgi?cmd=search&db=gene&term=RAF1) | Angiotensin II mediated activation of JNK Pathway via Pyk2 dependent signaling, Aspirin Blocks Signaling Pathway Involved in Platelet Activation, BCR Signaling Pathway, Bioactive Peptide Induced Signaling Pathway, Cadmium induces DNA synthesis and proliferation in macrophages, CCR3 signaling in Eosinophils, Ceramide Signaling Pathway, CXCR4 Signaling Pathway, EGF Signaling Pathway, EPO Signaling Pathway, Erk and PI-3 Kinase Are Necessary for Collagen Binding in Corneal Epithelia, Erk1/Erk2 Mapk Signaling pathway, Fc Epsilon Receptor I Signaling in Mast Cells, fMLP induced chemokine gene expression in HMC-1 cells, Growth Hormone Signaling Pathway, IGF-1 Signaling Pathway, IL 2 signaling pathway, IL 3 signaling pathway, IL 6 signaling pathway, IL-2 Receptor Beta Chain in T cell Activation, Influence of Ras and Rho proteins on G1 to S Transition, Inhibition of Cellular Proliferation by Gleevec, Insulin Signaling Pathway, Integrin Signaling Pathway, Keratinocyte Differentiation, Links betw ... |
| 0.0016206 | 0.044324 | 1238.966255 | 2531.635665 | 0.4893936 | 3420300 | [EEF2K](http://www.ncbi.nlm.nih.gov/entrez/query.fcgi?cmd=search&db=gene&term=EEF2K) | Eukaryotic protein translation |
| 0.0027689 | 0.057195 | 3620.378765 | 7409.234284 | 0.4886306 | 20392 | [CISD1](http://www.ncbi.nlm.nih.gov/entrez/query.fcgi?cmd=search&db=gene&term=CISD1) |  |
| 0.0045581 | 0.071912 | 1557.510577 | 3193.111706 | 0.487772 | 1110575 | [NDUFC1](http://www.ncbi.nlm.nih.gov/entrez/query.fcgi?cmd=search&db=gene&term=NDUFC1) | Oxidative phosphorylation |
| 1.39E-05 | 0.012234 | 162.9579324 | 334.4067454 | 0.4873046 | 4590669 | [PRPH2](http://www.ncbi.nlm.nih.gov/entrez/query.fcgi?cmd=search&db=gene&term=PRPH2) |  |
| 0.0001129 | 0.018033 | 887.1258517 | 1820.917769 | 0.4871861 | 4210347 | [DUSP28](http://www.ncbi.nlm.nih.gov/entrez/query.fcgi?cmd=search&db=gene&term=DUSP28) |  |
| 0.0014602 | 0.042243 | 493.004655 | 1012.216287 | 0.4870547 | 6590520 | [C14orf131](http://www.ncbi.nlm.nih.gov/entrez/query.fcgi?cmd=search&db=gene&term=C14orf131) |  |
| 0.0039777 | 0.067327 | 564.3186877 | 1158.727152 | 0.487016 | 6620626 | [CHCHD3](http://www.ncbi.nlm.nih.gov/entrez/query.fcgi?cmd=search&db=gene&term=CHCHD3) |  |
| 0.0001301 | 0.018344 | 173.0634978 | 355.4118911 | 0.4869378 | 3400577 | [EIF2S2](http://www.ncbi.nlm.nih.gov/entrez/query.fcgi?cmd=search&db=gene&term=EIF2S2) | Double Stranded RNA Induced Gene Expression, Regulation of eIF2, Skeletal muscle hypertrophy is regulated via AKT/mTOR pathway |
| 0.0018712 | 0.04743 | 566.7591242 | 1164.537123 | 0.4866819 | 1660039 | [DHPS](http://www.ncbi.nlm.nih.gov/entrez/query.fcgi?cmd=search&db=gene&term=DHPS) |  |
| 0.0031933 | 0.060634 | 3719.667886 | 7643.725091 | 0.4866303 | 6560156 | [DUSP3](http://www.ncbi.nlm.nih.gov/entrez/query.fcgi?cmd=search&db=gene&term=DUSP3) | MAPK signaling pathway, cell_cycle, cell_signaling |
| 0.000744 | 0.031048 | 290.5872737 | 597.5104122 | 0.4863301 | 5720703 | [AKTIP](http://www.ncbi.nlm.nih.gov/entrez/query.fcgi?cmd=search&db=gene&term=AKTIP) |  |
| 0.0002709 | 0.02261 | 168.0827547 | 345.719081 | 0.486183 | 610768 | [TAPT1](http://www.ncbi.nlm.nih.gov/entrez/query.fcgi?cmd=search&db=gene&term=TAPT1) |  |
| 0.0005973 | 0.028704 | 834.2503886 | 1716.065072 | 0.4861415 | 3440524 | [PTP4A3](http://www.ncbi.nlm.nih.gov/entrez/query.fcgi?cmd=search&db=gene&term=PTP4A3) |  |
| 0.0002564 | 0.022164 | 765.0017504 | 1579.402146 | 0.4843616 | 6580646 | [PSMC5](http://www.ncbi.nlm.nih.gov/entrez/query.fcgi?cmd=search&db=gene&term=PSMC5) | Proteasome |
| 0.0014078 | 0.041597 | 859.1449505 | 1774.160224 | 0.4842544 | 460072 | [MTX2](http://www.ncbi.nlm.nih.gov/entrez/query.fcgi?cmd=search&db=gene&term=MTX2) |  |
| 0.0011013 | 0.0375 | 596.7516512 | 1232.516669 | 0.4841733 | 4640392 | [MLLT6](http://www.ncbi.nlm.nih.gov/entrez/query.fcgi?cmd=search&db=gene&term=MLLT6) | misc |
| 0.0021054 | 0.049924 | 216.8668744 | 448.1617997 | 0.4839031 | 4590593 | [YAF2](http://www.ncbi.nlm.nih.gov/entrez/query.fcgi?cmd=search&db=gene&term=YAF2) |  |
| 0.00384 | 0.065994 | 833.0299138 | 1722.383555 | 0.4836495 | 4900431 | [STUB1](http://www.ncbi.nlm.nih.gov/entrez/query.fcgi?cmd=search&db=gene&term=STUB1) |  |
| 0.0003235 | 0.023725 | 444.0547672 | 919.2918796 | 0.48304 | 2140470 | [PSMA2](http://www.ncbi.nlm.nih.gov/entrez/query.fcgi?cmd=search&db=gene&term=PSMA2) | Proteasome |
| 0.0049495 | 0.074092 | 498.65669 | 1033.215265 | 0.4826261 | 6380064 | [LRPPRC](http://www.ncbi.nlm.nih.gov/entrez/query.fcgi?cmd=search&db=gene&term=LRPPRC) |  |
| 0.0042002 | 0.068875 | 154.7120153 | 320.9828922 | 0.4819946 | 4890041 | [DUSP8](http://www.ncbi.nlm.nih.gov/entrez/query.fcgi?cmd=search&db=gene&term=DUSP8) | Regulation of MAP Kinase Pathways Through Dual Specificity Phosphatases, MAPK signaling pathway |
| 0.0021907 | 0.050822 | 157.2799052 | 326.7849308 | 0.4812949 | 4120176 | [C9orf23](http://www.ncbi.nlm.nih.gov/entrez/query.fcgi?cmd=search&db=gene&term=C9orf23) |  |
| 0.0001235 | 0.018034 | 967.7103758 | 2012.973463 | 0.4807368 | 3610386 | [CPEB3](http://www.ncbi.nlm.nih.gov/entrez/query.fcgi?cmd=search&db=gene&term=CPEB3) |  |
| 0.0042067 | 0.068875 | 400.3162596 | 832.7612335 | 0.4807095 | 7210300 | [C10orf104](http://www.ncbi.nlm.nih.gov/entrez/query.fcgi?cmd=search&db=gene&term=C10orf104) |  |
| 0.0024643 | 0.053789 | 2031.708495 | 4227.895242 | 0.4805484 | 1440452 | [LOC88523](http://www.ncbi.nlm.nih.gov/entrez/query.fcgi?cmd=search&db=gene&term=LOC88523) |  |
| 0.0002872 | 0.022818 | 3417.846883 | 7113.40569 | 0.4804797 | 1710168 | [NDUFS3](http://www.ncbi.nlm.nih.gov/entrez/query.fcgi?cmd=search&db=gene&term=NDUFS3) | Oxidative phosphorylation |
| 0.0003666 | 0.024601 | 131.380605 | 273.5959678 | 0.4801993 | 3420168 |  |  |
| 0.0002719 | 0.022619 | 761.4017403 | 1587.708915 | 0.47956 | 4900609 | [C18orf55](http://www.ncbi.nlm.nih.gov/entrez/query.fcgi?cmd=search&db=gene&term=C18orf55) |  |
| 0.0019562 | 0.048578 | 663.7786648 | 1385.269304 | 0.4791694 | 6060113 | [TBX15](http://www.ncbi.nlm.nih.gov/entrez/query.fcgi?cmd=search&db=gene&term=TBX15) | development |
| 0.0005624 | 0.028359 | 163.0356313 | 340.7592151 | 0.4784482 | 270546 | [PIP4K2B](http://www.ncbi.nlm.nih.gov/entrez/query.fcgi?cmd=search&db=gene&term=PIP4K2B) | Inositol phosphate metabolism, Phosphatidylinositol signaling system, Regulation of actin cytoskeleton |
| 0.0029731 | 0.058744 | 1794.591958 | 3753.918229 | 0.4780584 | 50129 | [FHOD1](http://www.ncbi.nlm.nih.gov/entrez/query.fcgi?cmd=search&db=gene&term=FHOD1) |  |
| 0.0008229 | 0.033236 | 259.6299103 | 543.9727836 | 0.4772847 | 1240477 | [RTN4IP1](http://www.ncbi.nlm.nih.gov/entrez/query.fcgi?cmd=search&db=gene&term=RTN4IP1) |  |
| 0.0020135 | 0.049021 | 105.6790383 | 221.4403542 | 0.4772348 | 1780594 | [PTPN20A](http://www.ncbi.nlm.nih.gov/entrez/query.fcgi?cmd=search&db=gene&term=PTPN20A) |  |
| 0.0046398 | 0.072259 | 198.2392886 | 415.6219138 | 0.4769703 | 1450408 | [SIX1](http://www.ncbi.nlm.nih.gov/entrez/query.fcgi?cmd=search&db=gene&term=SIX1) |  |
| 0.0009636 | 0.035072 | 129.7309934 | 272.2592296 | 0.4764981 | 5810017 | [ALPK3](http://www.ncbi.nlm.nih.gov/entrez/query.fcgi?cmd=search&db=gene&term=ALPK3) |  |
| 3.35E-05 | 0.014558 | 176.0931895 | 370.2493522 | 0.475607 | 2640670 | [IDI1](http://www.ncbi.nlm.nih.gov/entrez/query.fcgi?cmd=search&db=gene&term=IDI1) | Biosynthesis of steroids, Terpenoid biosynthesis |
| 0.000359 | 0.024601 | 320.9670321 | 675.6596646 | 0.4750425 | 5690280 | [FXR2](http://www.ncbi.nlm.nih.gov/entrez/query.fcgi?cmd=search&db=gene&term=FXR2) |  |
| 0.0005343 | 0.027911 | 369.4606352 | 777.9999494 | 0.4748852 | 1090301 | [SDHC](http://www.ncbi.nlm.nih.gov/entrez/query.fcgi?cmd=search&db=gene&term=SDHC) | immunology |
| 0.0044534 | 0.070963 | 624.0393248 | 1316.00104 | 0.4741936 | 7200021 | [CUL4A](http://www.ncbi.nlm.nih.gov/entrez/query.fcgi?cmd=search&db=gene&term=CUL4A) |  |
| 0.000615 | 0.02914 | 274.7061893 | 579.4047478 | 0.4741179 | 6620133 | [PANK1](http://www.ncbi.nlm.nih.gov/entrez/query.fcgi?cmd=search&db=gene&term=PANK1) | Pantothenate and CoA biosynthesis |
| 0.000831 | 0.033292 | 154.1161118 | 325.1619359 | 0.4739673 | 4290544 | [SNRPD3](http://www.ncbi.nlm.nih.gov/entrez/query.fcgi?cmd=search&db=gene&term=SNRPD3) | Spliceosomal Assembly |
| 0.0001671 | 0.019753 | 1584.545103 | 3345.647655 | 0.4736139 | 1780348 | [FH](http://www.ncbi.nlm.nih.gov/entrez/query.fcgi?cmd=search&db=gene&term=FH) | Citrate cycle (TCA cycle), Reductive carboxylate cycle (CO2 fixation) |
| 0.0003367 | 0.024267 | 152.6128426 | 322.2710777 | 0.4735543 | 5860369 | [KIAA0564](http://www.ncbi.nlm.nih.gov/entrez/query.fcgi?cmd=search&db=gene&term=KIAA0564) |  |
| 0.0001322 | 0.01855 | 147.3630095 | 311.415144 | 0.4732044 | 5550164 |  |  |
| 0.0019065 | 0.047943 | 317.1789197 | 670.800406 | 0.4728365 | 5560576 | [NEDD4](http://www.ncbi.nlm.nih.gov/entrez/query.fcgi?cmd=search&db=gene&term=NEDD4) | Ubiquitin mediated proteolysis |
| 0.0030221 | 0.059206 | 2176.557549 | 4609.188791 | 0.4722214 | 3400674 | [MRPS15](http://www.ncbi.nlm.nih.gov/entrez/query.fcgi?cmd=search&db=gene&term=MRPS15) |  |
| 0.0022019 | 0.050881 | 357.9215321 | 758.9036195 | 0.4716298 | 4150070 | [DNAJC19](http://www.ncbi.nlm.nih.gov/entrez/query.fcgi?cmd=search&db=gene&term=DNAJC19) |  |
| 0.00228 | 0.05198 | 180.6950927 | 383.1588429 | 0.4715932 | 1500553 | [NUSAP1](http://www.ncbi.nlm.nih.gov/entrez/query.fcgi?cmd=search&db=gene&term=NUSAP1) |  |
| 0.002202 | 0.050881 | 1218.061664 | 2587.849075 | 0.470685 | 5340138 | [MRPS18B](http://www.ncbi.nlm.nih.gov/entrez/query.fcgi?cmd=search&db=gene&term=MRPS18B) |  |
| 0.0041341 | 0.06838 | 481.5332576 | 1023.503444 | 0.4704755 | 130121 | [HSF2](http://www.ncbi.nlm.nih.gov/entrez/query.fcgi?cmd=search&db=gene&term=HSF2) |  |
| 0.0024609 | 0.053782 | 752.2245686 | 1599.129363 | 0.4703963 | 5220195 | [UQCRC2](http://www.ncbi.nlm.nih.gov/entrez/query.fcgi?cmd=search&db=gene&term=UQCRC2) | Oxidative phosphorylation |
| 0.0015012 | 0.04266 | 236.861064 | 503.7635857 | 0.470183 | 7380010 | [APIP](http://www.ncbi.nlm.nih.gov/entrez/query.fcgi?cmd=search&db=gene&term=APIP) |  |
| 0.0005452 | 0.028008 | 296.6132385 | 630.9842858 | 0.4700802 | 4540020 | [SCN3A](http://www.ncbi.nlm.nih.gov/entrez/query.fcgi?cmd=search&db=gene&term=SCN3A) |  |
| 0.0044068 | 0.070643 | 524.2534207 | 1115.348138 | 0.4700357 | 270114 | [FAM134B](http://www.ncbi.nlm.nih.gov/entrez/query.fcgi?cmd=search&db=gene&term=FAM134B) |  |
| 0.0032601 | 0.061248 | 403.0185475 | 857.5702579 | 0.469954 | 1260435 | [ZNF511](http://www.ncbi.nlm.nih.gov/entrez/query.fcgi?cmd=search&db=gene&term=ZNF511) |  |
| 0.0001441 | 0.019068 | 568.0462838 | 1214.440512 | 0.4677432 | 1260195 | [DLD](http://www.ncbi.nlm.nih.gov/entrez/query.fcgi?cmd=search&db=gene&term=DLD) | Alanine and aspartate metabolism, Citrate cycle (TCA cycle), Glycine, serine and threonine metabolism, Glycolysis / Gluconeogenesis, Pyruvate metabolism |
| 0.0027311 | 0.056864 | 3137.127943 | 6707.698885 | 0.4676906 | 1990079 | [FBXO32](http://www.ncbi.nlm.nih.gov/entrez/query.fcgi?cmd=search&db=gene&term=FBXO32) |  |
| 0.0012272 | 0.039169 | 808.1092271 | 1731.062092 | 0.4668286 | 3170110 | [CCNG1](http://www.ncbi.nlm.nih.gov/entrez/query.fcgi?cmd=search&db=gene&term=CCNG1) |  |
| 0.0003599 | 0.024601 | 219.8127076 | 470.9029828 | 0.4667898 | 1090347 | [UBFD1](http://www.ncbi.nlm.nih.gov/entrez/query.fcgi?cmd=search&db=gene&term=UBFD1) |  |
| 0.001573 | 0.043775 | 1252.464129 | 2687.937573 | 0.4659573 | 5960674 | [PRDX2](http://www.ncbi.nlm.nih.gov/entrez/query.fcgi?cmd=search&db=gene&term=PRDX2) |  |
| 0.0008712 | 0.033692 | 142.4994298 | 305.9863538 | 0.4657052 | 5900133 | [LOC90624](http://www.ncbi.nlm.nih.gov/entrez/query.fcgi?cmd=search&db=gene&term=LOC90624) |  |
| 0.0008038 | 0.03259 | 123.7067278 | 266.5677263 | 0.4640724 | 4730050 | [CLTCL1](http://www.ncbi.nlm.nih.gov/entrez/query.fcgi?cmd=search&db=gene&term=CLTCL1) | Huntington\'s disease |
| 0.0039979 | 0.067531 | 756.7761692 | 1631.369745 | 0.46389 | 1070025 | [ATPIF1](http://www.ncbi.nlm.nih.gov/entrez/query.fcgi?cmd=search&db=gene&term=ATPIF1) |  |
| 0.0004046 | 0.025492 | 1046.420259 | 2256.985294 | 0.4636363 | 3780411 | [C1orf43](http://www.ncbi.nlm.nih.gov/entrez/query.fcgi?cmd=search&db=gene&term=C1orf43) |  |
| 0.0017682 | 0.046185 | 644.7025892 | 1392.800847 | 0.4628821 | 2070470 | [C1orf21](http://www.ncbi.nlm.nih.gov/entrez/query.fcgi?cmd=search&db=gene&term=C1orf21) |  |
| 0.0025432 | 0.05461 | 272.7307851 | 589.3497637 | 0.4627656 | 5570279 | [HIST1H1C](http://www.ncbi.nlm.nih.gov/entrez/query.fcgi?cmd=search&db=gene&term=HIST1H1C) |  |
| 0.0025172 | 0.054402 | 199.6621954 | 431.5686746 | 0.4626429 | 3840360 | [LARP4](http://www.ncbi.nlm.nih.gov/entrez/query.fcgi?cmd=search&db=gene&term=LARP4) |  |
| 0.0002241 | 0.020929 | 138.2826115 | 299.0051825 | 0.4624756 | 6560634 | [PNPLA4](http://www.ncbi.nlm.nih.gov/entrez/query.fcgi?cmd=search&db=gene&term=PNPLA4) |  |
| 0.0021773 | 0.050596 | 319.3955751 | 692.9237345 | 0.460939 | 1820189 | [ICT1](http://www.ncbi.nlm.nih.gov/entrez/query.fcgi?cmd=search&db=gene&term=ICT1) | immunology |
| 0.0005867 | 0.028704 | 172.3445673 | 374.2705265 | 0.4604813 | 4780307 | [PLCL1](http://www.ncbi.nlm.nih.gov/entrez/query.fcgi?cmd=search&db=gene&term=PLCL1) |  |
| 0.0031537 | 0.060421 | 176.2368179 | 382.8991269 | 0.4602696 | 2970600 | [MB](http://www.ncbi.nlm.nih.gov/entrez/query.fcgi?cmd=search&db=gene&term=MB) |  |
| 0.000901 | 0.034003 | 1699.936976 | 3693.684939 | 0.4602279 | 940669 | [TRPT1](http://www.ncbi.nlm.nih.gov/entrez/query.fcgi?cmd=search&db=gene&term=TRPT1) |  |
| 0.000162 | 0.019753 | 113.3597545 | 246.3748137 | 0.460111 | 5560639 | [MB](http://www.ncbi.nlm.nih.gov/entrez/query.fcgi?cmd=search&db=gene&term=MB) |  |
| 0.0005893 | 0.028704 | 1158.231872 | 2517.769054 | 0.4600231 | 4150142 | [SUCLG1](http://www.ncbi.nlm.nih.gov/entrez/query.fcgi?cmd=search&db=gene&term=SUCLG1) | Citrate cycle (TCA cycle), Propanoate metabolism |
| 0.0008301 | 0.033292 | 4009.744367 | 8717.082512 | 0.459987 | 4050491 | [TCEA3](http://www.ncbi.nlm.nih.gov/entrez/query.fcgi?cmd=search&db=gene&term=TCEA3) |  |
| 0.0031356 | 0.060314 | 238.9594695 | 520.3640261 | 0.459216 | 1570079 | [PLCB1](http://www.ncbi.nlm.nih.gov/entrez/query.fcgi?cmd=search&db=gene&term=PLCB1) | ?-arrestins in GPCR Desensitization, Activation of PKC through G protein coupled receptor, Aspirin Blocks Signaling Pathway Involved in Platelet Activation, Cadmium induces DNA synthesis and proliferation in macrophages, CCR3 signaling in Eosinophils, Eicosanoid Metabolism, fMLP induced chemokine gene expression in HMC-1 cells, G-Protein Signaling Through Tubby Proteins, Phospholipase C Signaling Pathway, Phospholipids as signalling intermediaries, PKC-catalyzed phosphorylation of inhibitory phosphoprotein of myosin phosphatase, Regulation of ck1/cdk5 by type 1 glutamate receptors, Role of ?-arrestins in the activation and targeting of MAP kinases, Roles of ?-arrestin-dependent Recruitment of Src Kinases in GPCR Signaling, Thrombin signaling and protease-activated receptors, Calcium signaling pathway, Gap junction, GnRH signaling pathway, Inositol phosphate metabolism, Long-term depression, Long-term potentiation, Phosphatidylinositol signaling system, Wnt signaling pathway |
| 0.0002043 | 0.020451 | 118.0349156 | 257.1134213 | 0.4590772 | 70435 | [ATP2B2](http://www.ncbi.nlm.nih.gov/entrez/query.fcgi?cmd=search&db=gene&term=ATP2B2) | Calcium signaling pathway, immunology |
| 0.0004506 | 0.026228 | 267.5855874 | 582.9948407 | 0.4589845 | 4760328 | [FASTK](http://www.ncbi.nlm.nih.gov/entrez/query.fcgi?cmd=search&db=gene&term=FASTK) |  |
| 0.0029032 | 0.058373 | 188.8638642 | 413.2554609 | 0.4570148 | 7160471 | [MSRB3](http://www.ncbi.nlm.nih.gov/entrez/query.fcgi?cmd=search&db=gene&term=MSRB3) |  |
| 0.0023132 | 0.052324 | 2275.841308 | 4986.967173 | 0.4563578 | 5130593 | [CAMK2B](http://www.ncbi.nlm.nih.gov/entrez/query.fcgi?cmd=search&db=gene&term=CAMK2B) | Calcium signaling pathway, GnRH signaling pathway, Long-term potentiation, Olfactory transduction, Wnt signaling pathway |
| 0.004098 | 0.068054 | 178.6020571 | 393.997823 | 0.4533072 | 2750392 | [EPM2A](http://www.ncbi.nlm.nih.gov/entrez/query.fcgi?cmd=search&db=gene&term=EPM2A) |  |
| 0.0021707 | 0.05056 | 652.5382243 | 1440.579008 | 0.4529694 | 7040307 | [UQCC](http://www.ncbi.nlm.nih.gov/entrez/query.fcgi?cmd=search&db=gene&term=UQCC) |  |
| 0.0004463 | 0.026228 | 177.7729749 | 392.5074412 | 0.4529162 | 1510079 | [ARMETL1](http://www.ncbi.nlm.nih.gov/entrez/query.fcgi?cmd=search&db=gene&term=ARMETL1) |  |
| 0.0014394 | 0.041807 | 2130.676023 | 4705.067063 | 0.4528471 | 6960056 | [JTV1](http://www.ncbi.nlm.nih.gov/entrez/query.fcgi?cmd=search&db=gene&term=JTV1) | Regulation of eIF2 |
| 0.0021775 | 0.050596 | 153.265229 | 338.5964579 | 0.4526486 | 5080259 | [KCNS3](http://www.ncbi.nlm.nih.gov/entrez/query.fcgi?cmd=search&db=gene&term=KCNS3) |  |
| 0.000261 | 0.022362 | 318.2682759 | 703.9404496 | 0.4521239 | 7650343 | [USP47](http://www.ncbi.nlm.nih.gov/entrez/query.fcgi?cmd=search&db=gene&term=USP47) |  |
| 0.0014391 | 0.041807 | 1888.593762 | 4216.107196 | 0.4479473 | 6200369 | [PPP1CB](http://www.ncbi.nlm.nih.gov/entrez/query.fcgi?cmd=search&db=gene&term=PPP1CB) | Focal adhesion, Insulin signaling pathway, Long-term potentiation, Regulation of actin cytoskeleton, cell_cycle, cell_signaling |
| 0.0002473 | 0.021635 | 2156.839306 | 4819.086691 | 0.4475618 | 3180470 | [SDHA](http://www.ncbi.nlm.nih.gov/entrez/query.fcgi?cmd=search&db=gene&term=SDHA) | Citrate cycle (TCA cycle), Oxidative phosphorylation |
| 8.50E-05 | 0.017191 | 350.903736 | 785.2694557 | 0.4468577 | 2450400 | [RASGRP3](http://www.ncbi.nlm.nih.gov/entrez/query.fcgi?cmd=search&db=gene&term=RASGRP3) | B cell receptor signaling pathway, MAPK signaling pathway |
| 0.0004114 | 0.025615 | 700.9743018 | 1570.948269 | 0.4462109 | 6380563 | [UCRC](http://www.ncbi.nlm.nih.gov/entrez/query.fcgi?cmd=search&db=gene&term=UCRC) | Oxidative phosphorylation |
| 0.0040238 | 0.067639 | 123.5250864 | 277.0238177 | 0.4459006 | 7000754 | [AMY2B](http://www.ncbi.nlm.nih.gov/entrez/query.fcgi?cmd=search&db=gene&term=AMY2B) | Starch and sucrose metabolism |
| 0.000943 | 0.034802 | 274.0880922 | 615.1836141 | 0.4455387 | 4220327 | [MRPL38](http://www.ncbi.nlm.nih.gov/entrez/query.fcgi?cmd=search&db=gene&term=MRPL38) |  |
| 0.0025254 | 0.054402 | 1427.04072 | 3209.330178 | 0.4446538 | 4810224 | [NDUFA9](http://www.ncbi.nlm.nih.gov/entrez/query.fcgi?cmd=search&db=gene&term=NDUFA9) | Oxidative phosphorylation |
| 0.0012858 | 0.039772 | 388.3329391 | 874.8719114 | 0.4438741 | 2640554 | [EEF1B2](http://www.ncbi.nlm.nih.gov/entrez/query.fcgi?cmd=search&db=gene&term=EEF1B2) |  |
| 0.0040152 | 0.067591 | 396.1667182 | 893.494577 | 0.4433902 | 3130020 | [CAV3](http://www.ncbi.nlm.nih.gov/entrez/query.fcgi?cmd=search&db=gene&term=CAV3) | Focal adhesion, immunology |
| 0.003449 | 0.063044 | 724.95271 | 1635.83582 | 0.4431696 | 1990563 | [BRP44L](http://www.ncbi.nlm.nih.gov/entrez/query.fcgi?cmd=search&db=gene&term=BRP44L) |  |
| 0.0037422 | 0.065163 | 1717.193869 | 3881.921444 | 0.4423567 | 4780040 | [MRPL41](http://www.ncbi.nlm.nih.gov/entrez/query.fcgi?cmd=search&db=gene&term=MRPL41) |  |
| 0.000262 | 0.022382 | 117.2150568 | 265.4285401 | 0.4416068 | 4610731 | [CHAF1B](http://www.ncbi.nlm.nih.gov/entrez/query.fcgi?cmd=search&db=gene&term=CHAF1B) |  |
| 0.003716 | 0.06498 | 330.2408224 | 753.5088121 | 0.4382707 | 6580121 | [MID1IP1](http://www.ncbi.nlm.nih.gov/entrez/query.fcgi?cmd=search&db=gene&term=MID1IP1) |  |
| 0.0001852 | 0.019997 | 3809.515846 | 8695.135612 | 0.4381203 | 1770520 | [CYC1](http://www.ncbi.nlm.nih.gov/entrez/query.fcgi?cmd=search&db=gene&term=CYC1) | Oxidative phosphorylation |
| 0.0017746 | 0.046294 | 232.745449 | 531.3712017 | 0.4380092 | 1470427 | [ALDH4A1](http://www.ncbi.nlm.nih.gov/entrez/query.fcgi?cmd=search&db=gene&term=ALDH4A1) | Catabolic Pathways for Arginine , Histidine, Glutamate, Glutamine, and Proline, Arginine and proline metabolism, Glutamate metabolism |
| 0.0004587 | 0.02654 | 412.724132 | 942.4366827 | 0.437933 | 7150673 | [PRRG1](http://www.ncbi.nlm.nih.gov/entrez/query.fcgi?cmd=search&db=gene&term=PRRG1) |  |
| 0.0016629 | 0.044977 | 1290.926017 | 2952.648458 | 0.4372095 | 3390435 | [SCN4A](http://www.ncbi.nlm.nih.gov/entrez/query.fcgi?cmd=search&db=gene&term=SCN4A) | immunology |
| 0.002117 | 0.050014 | 704.7528033 | 1612.719031 | 0.4369966 | 4290088 | [MSRB3](http://www.ncbi.nlm.nih.gov/entrez/query.fcgi?cmd=search&db=gene&term=MSRB3) |  |
| 0.0031682 | 0.06054 | 335.7876463 | 769.1407626 | 0.436575 | 110110 | [HSPE1](http://www.ncbi.nlm.nih.gov/entrez/query.fcgi?cmd=search&db=gene&term=HSPE1) |  |
| 0.0002233 | 0.020922 | 208.7477492 | 478.2789885 | 0.436456 | 6100594 | [PCBD2](http://www.ncbi.nlm.nih.gov/entrez/query.fcgi?cmd=search&db=gene&term=PCBD2) |  |
| 0.0003062 | 0.02341 | 129.1421225 | 295.9606203 | 0.436349 | 5050554 | [LOC649276](http://www.ncbi.nlm.nih.gov/entrez/query.fcgi?cmd=search&db=gene&term=LOC649276) |  |
| 0.0029954 | 0.058944 | 544.8906811 | 1250.563623 | 0.4357161 | 5910632 | [SMARCD3](http://www.ncbi.nlm.nih.gov/entrez/query.fcgi?cmd=search&db=gene&term=SMARCD3) | development |
| 0.0016757 | 0.045149 | 1713.912232 | 3933.577548 | 0.4357133 | 4250372 | [KLHDC3](http://www.ncbi.nlm.nih.gov/entrez/query.fcgi?cmd=search&db=gene&term=KLHDC3) |  |
| 0.0023943 | 0.052965 | 271.5497334 | 623.6965177 | 0.4353876 | 1990491 | [AK2](http://www.ncbi.nlm.nih.gov/entrez/query.fcgi?cmd=search&db=gene&term=AK2) | Purine metabolism, cell_cycle, cell_signaling, signal_transduction |
| 0.0025904 | 0.05512 | 165.070305 | 381.5983294 | 0.4325761 | 3290397 |  |  |
| 5.74E-05 | 0.01576 | 339.6516975 | 785.3192901 | 0.4325014 | 6400240 | [KIAA0368](http://www.ncbi.nlm.nih.gov/entrez/query.fcgi?cmd=search&db=gene&term=KIAA0368) |  |
| 0.003528 | 0.063701 | 201.9672194 | 467.0005644 | 0.4324775 | 4730767 | [ANKRD9](http://www.ncbi.nlm.nih.gov/entrez/query.fcgi?cmd=search&db=gene&term=ANKRD9) |  |
| 0.0006273 | 0.029291 | 224.4298777 | 519.1330052 | 0.4323167 | 7320162 | [DCUN1D2](http://www.ncbi.nlm.nih.gov/entrez/query.fcgi?cmd=search&db=gene&term=DCUN1D2) |  |
| 0.0005396 | 0.028008 | 413.2849651 | 956.0374034 | 0.4322895 | 430202 | [NOL3](http://www.ncbi.nlm.nih.gov/entrez/query.fcgi?cmd=search&db=gene&term=NOL3) |  |
| 0.0036632 | 0.064645 | 1622.434994 | 3755.18896 | 0.4320515 | 6020682 | [RGMA](http://www.ncbi.nlm.nih.gov/entrez/query.fcgi?cmd=search&db=gene&term=RGMA) |  |
| 0.0006974 | 0.030053 | 324.9219858 | 753.3871617 | 0.4312816 | 770291 | [HOXA10](http://www.ncbi.nlm.nih.gov/entrez/query.fcgi?cmd=search&db=gene&term=HOXA10) | development |
| 0.0046875 | 0.072613 | 197.6507959 | 458.3697518 | 0.4312038 | 4290368 | [PSTPIP2](http://www.ncbi.nlm.nih.gov/entrez/query.fcgi?cmd=search&db=gene&term=PSTPIP2) |  |
| 0.00197 | 0.048685 | 141.1174468 | 327.7475126 | 0.4305676 | 160343 | [ALDH4A1](http://www.ncbi.nlm.nih.gov/entrez/query.fcgi?cmd=search&db=gene&term=ALDH4A1) | Catabolic Pathways for Arginine , Histidine, Glutamate, Glutamine, and Proline, Arginine and proline metabolism, Glutamate metabolism |
| 0.0041215 | 0.06821 | 1495.807021 | 3475.844512 | 0.4303435 | 1740682 | [PHKB](http://www.ncbi.nlm.nih.gov/entrez/query.fcgi?cmd=search&db=gene&term=PHKB) | Calcium signaling pathway, Insulin signaling pathway |
| 0.0010309 | 0.036605 | 1266.912568 | 2947.61416 | 0.4298095 | 6060025 | [CUL5](http://www.ncbi.nlm.nih.gov/entrez/query.fcgi?cmd=search&db=gene&term=CUL5) |  |
| 0.0001016 | 0.017719 | 892.3058342 | 2076.660026 | 0.4296832 | 3440195 | [GFM1](http://www.ncbi.nlm.nih.gov/entrez/query.fcgi?cmd=search&db=gene&term=GFM1) |  |
| 0.0010558 | 0.036985 | 499.2229041 | 1163.446055 | 0.4290899 | 540131 | [PXMP2](http://www.ncbi.nlm.nih.gov/entrez/query.fcgi?cmd=search&db=gene&term=PXMP2) |  |
| 0.0003098 | 0.02341 | 3743.256221 | 8731.494428 | 0.4287074 | 5890091 | [DUSP26](http://www.ncbi.nlm.nih.gov/entrez/query.fcgi?cmd=search&db=gene&term=DUSP26) |  |
| 0.0002369 | 0.021369 | 2741.453157 | 6403.542453 | 0.4281151 | 6660600 | [COQ9](http://www.ncbi.nlm.nih.gov/entrez/query.fcgi?cmd=search&db=gene&term=COQ9) |  |
| 8.50E-06 | 0.012163 | 230.3769152 | 538.7083741 | 0.4276468 | 2750632 | [POLR2J3](http://www.ncbi.nlm.nih.gov/entrez/query.fcgi?cmd=search&db=gene&term=POLR2J3) |  |
| 0.004011 | 0.067591 | 139.2980001 | 326.2195644 | 0.4270069 | 4590347 | [ZNF784](http://www.ncbi.nlm.nih.gov/entrez/query.fcgi?cmd=search&db=gene&term=ZNF784) |  |
| 0.0024335 | 0.053344 | 391.2488681 | 916.3393291 | 0.4269694 | 6330689 | [DLAT](http://www.ncbi.nlm.nih.gov/entrez/query.fcgi?cmd=search&db=gene&term=DLAT) | Alanine and aspartate metabolism, Glycolysis / Gluconeogenesis, Pyruvate metabolism |
| 0.0037667 | 0.065289 | 725.1659944 | 1700.829163 | 0.4263603 | 7050706 | [PDHX](http://www.ncbi.nlm.nih.gov/entrez/query.fcgi?cmd=search&db=gene&term=PDHX) | Alanine and aspartate metabolism, Glycolysis / Gluconeogenesis, Pyruvate metabolism |
| 0.0029015 | 0.058373 | 496.0584961 | 1163.657187 | 0.4262926 | 4050711 | [LACTB2](http://www.ncbi.nlm.nih.gov/entrez/query.fcgi?cmd=search&db=gene&term=LACTB2) |  |
| 7.98E-05 | 0.016675 | 129.47907 | 303.9042418 | 0.4260522 | 460487 | [FAM55C](http://www.ncbi.nlm.nih.gov/entrez/query.fcgi?cmd=search&db=gene&term=FAM55C) |  |
| 0.000512 | 0.02772 | 3397.672734 | 7985.060144 | 0.4255037 | 6290296 | [NDUFB7](http://www.ncbi.nlm.nih.gov/entrez/query.fcgi?cmd=search&db=gene&term=NDUFB7) | Oxidative phosphorylation |
| 0.0018813 | 0.047598 | 133.256311 | 313.3180594 | 0.4253068 | 5810669 | [LBX1](http://www.ncbi.nlm.nih.gov/entrez/query.fcgi?cmd=search&db=gene&term=LBX1) |  |
| 0.0015493 | 0.043319 | 730.3860023 | 1719.133479 | 0.4248571 | 5570270 | [GYS1](http://www.ncbi.nlm.nih.gov/entrez/query.fcgi?cmd=search&db=gene&term=GYS1) | Insulin signaling pathway, Starch and sucrose metabolism, immunology |
| 0.0014166 | 0.041638 | 143.4123113 | 337.9063552 | 0.4244144 | 5080687 | [NDUFS1](http://www.ncbi.nlm.nih.gov/entrez/query.fcgi?cmd=search&db=gene&term=NDUFS1) | Oxidative phosphorylation |
| 0.0021971 | 0.050848 | 309.8767286 | 730.9558219 | 0.4239336 | 4070544 | [ATP5L](http://www.ncbi.nlm.nih.gov/entrez/query.fcgi?cmd=search&db=gene&term=ATP5L) | ATP synthesis, Oxidative phosphorylation |
| 0.0001237 | 0.018034 | 836.6846756 | 1978.227859 | 0.4229466 | 3390139 | [SDHD](http://www.ncbi.nlm.nih.gov/entrez/query.fcgi?cmd=search&db=gene&term=SDHD) | Electron Transport Reaction in Mitochondria, Citrate cycle (TCA cycle), Oxidative phosphorylation |
| 0.0026186 | 0.055435 | 878.7093757 | 2080.079953 | 0.4224402 | 4760520 | [MRPL15](http://www.ncbi.nlm.nih.gov/entrez/query.fcgi?cmd=search&db=gene&term=MRPL15) |  |
| 0.0009903 | 0.035745 | 139.0658042 | 329.2006756 | 0.4224347 | 7200170 | [PPM1J](http://www.ncbi.nlm.nih.gov/entrez/query.fcgi?cmd=search&db=gene&term=PPM1J) | Tight junction |
| 0.0029338 | 0.058402 | 648.9366006 | 1536.199896 | 0.4224298 | 3930639 | [ACO2](http://www.ncbi.nlm.nih.gov/entrez/query.fcgi?cmd=search&db=gene&term=ACO2) | immunology |
| 0.0025265 | 0.054402 | 279.4718913 | 661.9947845 | 0.4221663 | 6100333 | [LOC652634](http://www.ncbi.nlm.nih.gov/entrez/query.fcgi?cmd=search&db=gene&term=LOC652634) |  |
| 0.0023085 | 0.052308 | 158.8617886 | 376.9649108 | 0.4214233 | 6620630 | [XK](http://www.ncbi.nlm.nih.gov/entrez/query.fcgi?cmd=search&db=gene&term=XK) | immunology |
| 0.0003902 | 0.025241 | 259.1442376 | 616.3524441 | 0.4204481 | 3940066 | [KLHL30](http://www.ncbi.nlm.nih.gov/entrez/query.fcgi?cmd=search&db=gene&term=KLHL30) |  |
| 0.0007289 | 0.030727 | 237.7758079 | 566.1039619 | 0.4200215 | 160307 | [C9orf3](http://www.ncbi.nlm.nih.gov/entrez/query.fcgi?cmd=search&db=gene&term=C9orf3) |  |
| 0.0002592 | 0.022274 | 3517.18147 | 8376.586205 | 0.4198824 | 2470279 | [ACADM](http://www.ncbi.nlm.nih.gov/entrez/query.fcgi?cmd=search&db=gene&term=ACADM) | beta-Alanine metabolism, Fatty acid metabolism, PPAR signaling pathway, Propanoate metabolism, Valine, leucine and isoleucine degradation, immunology |
| 0.0027777 | 0.057259 | 237.3185639 | 566.0377296 | 0.4192628 | 520201 | [C6orf136](http://www.ncbi.nlm.nih.gov/entrez/query.fcgi?cmd=search&db=gene&term=C6orf136) |  |
| 0.0029185 | 0.058389 | 182.5229017 | 435.9363396 | 0.4186916 | 7380619 | [LOC728037](http://www.ncbi.nlm.nih.gov/entrez/query.fcgi?cmd=search&db=gene&term=LOC728037) |  |
| 3.50E-06 | 0.012163 | 520.351041 | 1247.093545 | 0.417251 | 4060692 | [SUCLG2](http://www.ncbi.nlm.nih.gov/entrez/query.fcgi?cmd=search&db=gene&term=SUCLG2) | Citrate cycle (TCA cycle), Propanoate metabolism |
| 0.0002217 | 0.020839 | 192.0334284 | 461.1111213 | 0.416458 | 1770338 | [C9orf23](http://www.ncbi.nlm.nih.gov/entrez/query.fcgi?cmd=search&db=gene&term=C9orf23) |  |
| 0.0039752 | 0.067327 | 344.4093887 | 827.7162373 | 0.416096 | 5080328 | [DOPEY2](http://www.ncbi.nlm.nih.gov/entrez/query.fcgi?cmd=search&db=gene&term=DOPEY2) |  |
| 0.0007423 | 0.031022 | 130.3385955 | 314.8307371 | 0.4139958 | 2140603 | [LOC645261](http://www.ncbi.nlm.nih.gov/entrez/query.fcgi?cmd=search&db=gene&term=LOC645261) |  |
| 0.0021726 | 0.050563 | 293.7713687 | 710.2386695 | 0.4136234 | 60719 | [KCNJ2](http://www.ncbi.nlm.nih.gov/entrez/query.fcgi?cmd=search&db=gene&term=KCNJ2) |  |
| 0.0002679 | 0.022554 | 248.9698827 | 602.2909729 | 0.4133714 | 5910397 | [LIAS](http://www.ncbi.nlm.nih.gov/entrez/query.fcgi?cmd=search&db=gene&term=LIAS) |  |
| 0.0015772 | 0.043775 | 418.0719003 | 1012.13215 | 0.4130606 | 840044 | [C10orf71](http://www.ncbi.nlm.nih.gov/entrez/query.fcgi?cmd=search&db=gene&term=C10orf71) |  |
| 0.0001887 | 0.020076 | 162.9066921 | 394.9026767 | 0.4125236 | 4040722 | [JPH2](http://www.ncbi.nlm.nih.gov/entrez/query.fcgi?cmd=search&db=gene&term=JPH2) |  |
| 0.0011191 | 0.037826 | 4636.346539 | 11263.89413 | 0.4116113 | 2940113 | [AGL](http://www.ncbi.nlm.nih.gov/entrez/query.fcgi?cmd=search&db=gene&term=AGL) | Starch and sucrose metabolism |
| 0.0014214 | 0.041638 | 189.6802277 | 462.3501648 | 0.4102523 | 4570014 | [C14orf122](http://www.ncbi.nlm.nih.gov/entrez/query.fcgi?cmd=search&db=gene&term=C14orf122) |  |
| 0.0010417 | 0.036763 | 3431.982109 | 8371.525322 | 0.409959 | 6620379 | [LRRC20](http://www.ncbi.nlm.nih.gov/entrez/query.fcgi?cmd=search&db=gene&term=LRRC20) |  |
| 0.0002195 | 0.020835 | 1980.010705 | 4831.484214 | 0.4098142 | 270376 | [GBAS](http://www.ncbi.nlm.nih.gov/entrez/query.fcgi?cmd=search&db=gene&term=GBAS) |  |
| 0.0033392 | 0.061973 | 251.5438727 | 614.2803767 | 0.4094936 | 520484 | [USP15](http://www.ncbi.nlm.nih.gov/entrez/query.fcgi?cmd=search&db=gene&term=USP15) |  |
| 0.0020124 | 0.049021 | 813.9548069 | 1988.060806 | 0.4094215 | 5220220 | [SUCLA2](http://www.ncbi.nlm.nih.gov/entrez/query.fcgi?cmd=search&db=gene&term=SUCLA2) | C5-Branched dibasic acid metabolism, Citrate cycle (TCA cycle), Propanoate metabolism, Reductive carboxylate cycle (CO2 fixation) |
| 0.0006829 | 0.030043 | 2727.643294 | 6681.595653 | 0.4082323 | 5860187 | [PHYH](http://www.ncbi.nlm.nih.gov/entrez/query.fcgi?cmd=search&db=gene&term=PHYH) |  |
| 4.35E-05 | 0.014864 | 544.3357269 | 1336.013369 | 0.4074328 | 1010475 | [CAMK2G](http://www.ncbi.nlm.nih.gov/entrez/query.fcgi?cmd=search&db=gene&term=CAMK2G) | Bioactive Peptide Induced Signaling Pathway, Ca++/ Calmodulin-dependent Protein Kinase Activation, Regulation of PGC-1a, Stathmin and breast cancer resistance to antimicrotubule agents, Transcription factor CREB and its extracellular signals, Calcium signaling pathway, GnRH signaling pathway, Long-term potentiation, Olfactory transduction, Wnt signaling pathway |
| 4.01E-05 | 0.014558 | 123.9810175 | 304.4914628 | 0.407174 | 6060156 |  |  |
| 0.0006434 | 0.029369 | 659.32784 | 1622.390486 | 0.4063928 | 3850767 | [SBK1](http://www.ncbi.nlm.nih.gov/entrez/query.fcgi?cmd=search&db=gene&term=SBK1) |  |
| 0.0020511 | 0.049303 | 337.1789859 | 833.7699208 | 0.4044029 | 3520689 | [DDX19A](http://www.ncbi.nlm.nih.gov/entrez/query.fcgi?cmd=search&db=gene&term=DDX19A) |  |
| 0.0003717 | 0.024685 | 222.0771069 | 549.1911547 | 0.4043712 | 3170544 | [CAMK2B](http://www.ncbi.nlm.nih.gov/entrez/query.fcgi?cmd=search&db=gene&term=CAMK2B) | Calcium signaling pathway, GnRH signaling pathway, Long-term potentiation, Olfactory transduction, Wnt signaling pathway |
| 0.0002891 | 0.022818 | 162.7537806 | 402.7592142 | 0.404097 | 4150195 | [FKBP3](http://www.ncbi.nlm.nih.gov/entrez/query.fcgi?cmd=search&db=gene&term=FKBP3) |  |
| 0.0035306 | 0.063701 | 5058.196464 | 12523.63272 | 0.4038921 | 4830372 | [ADSSL1](http://www.ncbi.nlm.nih.gov/entrez/query.fcgi?cmd=search&db=gene&term=ADSSL1) | Alanine and aspartate metabolism, Purine metabolism |
| 0.0001901 | 0.020078 | 671.9767306 | 1669.321291 | 0.4025449 | 1500689 | [AIFM1](http://www.ncbi.nlm.nih.gov/entrez/query.fcgi?cmd=search&db=gene&term=AIFM1) | Ceramide Signaling Pathway, Opposing roles of AIF in Apoptosis and Cell Survival, Role of Mitochondria in Apoptotic Signaling, Apoptosis |
| 0.0023274 | 0.052324 | 212.1206886 | 530.8725646 | 0.3995699 | 1570747 | [LINGO4](http://www.ncbi.nlm.nih.gov/entrez/query.fcgi?cmd=search&db=gene&term=LINGO4) |  |
| 0.0006614 | 0.029691 | 437.3018055 | 1094.863428 | 0.3994122 | 2850056 | [PHYH](http://www.ncbi.nlm.nih.gov/entrez/query.fcgi?cmd=search&db=gene&term=PHYH) |  |
| 0.0009602 | 0.035003 | 177.8304194 | 445.4243145 | 0.3992382 | 2680435 | [MRPL1](http://www.ncbi.nlm.nih.gov/entrez/query.fcgi?cmd=search&db=gene&term=MRPL1) |  |
| 0.0019712 | 0.048685 | 461.0490202 | 1156.727232 | 0.3985806 | 5860022 | [FXYD1](http://www.ncbi.nlm.nih.gov/entrez/query.fcgi?cmd=search&db=gene&term=FXYD1) |  |
| 0.00158 | 0.043775 | 106.7117723 | 268.5969036 | 0.3972934 | 520626 | [LOC151121](http://www.ncbi.nlm.nih.gov/entrez/query.fcgi?cmd=search&db=gene&term=LOC151121) |  |
| 0.0012956 | 0.039905 | 287.4992351 | 723.8394981 | 0.3971864 | 4050500 | [TAF6](http://www.ncbi.nlm.nih.gov/entrez/query.fcgi?cmd=search&db=gene&term=TAF6) | Basal transcription factors |
| 0.002791 | 0.057371 | 1238.371597 | 3125.250233 | 0.3962472 | 2060450 | [NNT](http://www.ncbi.nlm.nih.gov/entrez/query.fcgi?cmd=search&db=gene&term=NNT) | Nicotinate and nicotinamide metabolism |
| 0.0002835 | 0.022708 | 247.3620247 | 624.9194223 | 0.3958303 | 4890113 | [C1orf43](http://www.ncbi.nlm.nih.gov/entrez/query.fcgi?cmd=search&db=gene&term=C1orf43) |  |
| 0.0022527 | 0.051764 | 504.1025335 | 1274.461362 | 0.3955416 | 5360500 | [CPT1B](http://www.ncbi.nlm.nih.gov/entrez/query.fcgi?cmd=search&db=gene&term=CPT1B) | Adipocytokine signaling pathway, Fatty acid metabolism, PPAR signaling pathway |
| 0.0036766 | 0.064645 | 233.83595 | 592.9585557 | 0.3943546 | 3850452 | [LOC644310](http://www.ncbi.nlm.nih.gov/entrez/query.fcgi?cmd=search&db=gene&term=LOC644310) |  |
| 0.0002732 | 0.022619 | 222.0020125 | 563.2517906 | 0.3941435 | 5820324 | [PXMP2](http://www.ncbi.nlm.nih.gov/entrez/query.fcgi?cmd=search&db=gene&term=PXMP2) |  |
| 0.0017197 | 0.045525 | 194.9102254 | 494.6364946 | 0.3940474 | 940446 | [LOC646675](http://www.ncbi.nlm.nih.gov/entrez/query.fcgi?cmd=search&db=gene&term=LOC646675) |  |
| 0.0014943 | 0.042622 | 273.8298253 | 696.5615631 | 0.3931165 | 5690132 | [C16orf14](http://www.ncbi.nlm.nih.gov/entrez/query.fcgi?cmd=search&db=gene&term=C16orf14) |  |
| 0.0011458 | 0.038209 | 621.4304929 | 1586.017181 | 0.3918183 | 6450372 | [PDHA1](http://www.ncbi.nlm.nih.gov/entrez/query.fcgi?cmd=search&db=gene&term=PDHA1) | Shuttle for transfer of acetyl groups from mitochondria to the cytosol, Alanine and aspartate metabolism, Butanoate metabolism, Glycolysis / Gluconeogenesis, Pyruvate metabolism, Valine, leucine and isoleucine biosynthesis, immunology, metabolism, misc |
| 0.0023009 | 0.052299 | 361.6925403 | 926.7840167 | 0.3902663 | 1170524 | [C2orf7](http://www.ncbi.nlm.nih.gov/entrez/query.fcgi?cmd=search&db=gene&term=C2orf7) |  |
| 0.0029123 | 0.058389 | 427.2463934 | 1095.274955 | 0.3900814 | 2900543 | [ENDOG](http://www.ncbi.nlm.nih.gov/entrez/query.fcgi?cmd=search&db=gene&term=ENDOG) | Apoptotic DNA fragmentation and tissue homeostasis, Role of Mitochondria in Apoptotic Signaling, Apoptosis |
| 1.03E-05 | 0.012163 | 119.3301308 | 305.9414535 | 0.3900424 | 4180307 | [ARNT2](http://www.ncbi.nlm.nih.gov/entrez/query.fcgi?cmd=search&db=gene&term=ARNT2) |  |
| 0.0040666 | 0.06788 | 209.7665922 | 538.4293483 | 0.3895898 | 5390386 | [FREM2](http://www.ncbi.nlm.nih.gov/entrez/query.fcgi?cmd=search&db=gene&term=FREM2) |  |
| 0.0003103 | 0.02341 | 483.2946628 | 1241.874664 | 0.3891654 | 3850292 | [CHCHD4](http://www.ncbi.nlm.nih.gov/entrez/query.fcgi?cmd=search&db=gene&term=CHCHD4) |  |
| 0.0018128 | 0.046593 | 3278.649977 | 8501.496656 | 0.3856556 | 5870446 | [ATP5G1](http://www.ncbi.nlm.nih.gov/entrez/query.fcgi?cmd=search&db=gene&term=ATP5G1) | ATP synthesis, Oxidative phosphorylation |
| 0.000495 | 0.027438 | 149.8739607 | 388.6581887 | 0.3856189 | 1470544 | [PFKFB1](http://www.ncbi.nlm.nih.gov/entrez/query.fcgi?cmd=search&db=gene&term=PFKFB1) | Fructose and mannose metabolism |
| 0.0021688 | 0.050556 | 2648.886882 | 6877.464697 | 0.3851546 | 6020037 | [JSRP1](http://www.ncbi.nlm.nih.gov/entrez/query.fcgi?cmd=search&db=gene&term=JSRP1) |  |
| 0.0002092 | 0.020598 | 304.5936799 | 792.8247459 | 0.3841879 | 5050768 | [LONRF1](http://www.ncbi.nlm.nih.gov/entrez/query.fcgi?cmd=search&db=gene&term=LONRF1) |  |
| 0.001153 | 0.038243 | 199.8478347 | 520.2267963 | 0.3841552 | 4150408 | [P2RY2](http://www.ncbi.nlm.nih.gov/entrez/query.fcgi?cmd=search&db=gene&term=P2RY2) | Ion Channel and Phorbal Esters Signaling Pathway, Neuroactive ligand-receptor interaction |
| 0.0049188 | 0.074056 | 173.9865381 | 455.4147736 | 0.3820397 | 1010563 | [LOC652668](http://www.ncbi.nlm.nih.gov/entrez/query.fcgi?cmd=search&db=gene&term=LOC652668) |  |
| 0.0012193 | 0.039002 | 332.6173561 | 876.2045126 | 0.3796116 | 7040195 | [ST3GAL3](http://www.ncbi.nlm.nih.gov/entrez/query.fcgi?cmd=search&db=gene&term=ST3GAL3) | Glycan structures - biosynthesis 1, Glycan structures - biosynthesis 2, Glycosphingolipid biosynthesis - lactoseries, Keratan sulfate biosynthesis |
| 0.0020304 | 0.049226 | 285.1578619 | 751.2380544 | 0.3795839 | 5080594 | [IQWD1](http://www.ncbi.nlm.nih.gov/entrez/query.fcgi?cmd=search&db=gene&term=IQWD1) |  |
| 0.00037 | 0.024648 | 1379.143941 | 3646.650483 | 0.3781947 | 4760450 | [C16orf33](http://www.ncbi.nlm.nih.gov/entrez/query.fcgi?cmd=search&db=gene&term=C16orf33) |  |
| 0.000553 | 0.028179 | 604.920315 | 1601.301822 | 0.3777678 | 6180414 | [PTP4A1](http://www.ncbi.nlm.nih.gov/entrez/query.fcgi?cmd=search&db=gene&term=PTP4A1) |  |
| 0.0012136 | 0.038949 | 2923.41792 | 7749.781903 | 0.3772258 | 1070538 | [NDUFS8](http://www.ncbi.nlm.nih.gov/entrez/query.fcgi?cmd=search&db=gene&term=NDUFS8) | Oxidative phosphorylation |
| 0.002847 | 0.058029 | 145.0387547 | 384.5899563 | 0.3771257 | 3520193 | [ATP2B2](http://www.ncbi.nlm.nih.gov/entrez/query.fcgi?cmd=search&db=gene&term=ATP2B2) | Calcium signaling pathway, immunology |
| 0.0007405 | 0.030991 | 103.0205403 | 273.3663307 | 0.3768589 | 4200400 | [CA14](http://www.ncbi.nlm.nih.gov/entrez/query.fcgi?cmd=search&db=gene&term=CA14) | Nitrogen metabolism |
| 0.002612 | 0.055412 | 269.5246377 | 715.5155675 | 0.3766859 | 4890592 | [PDE7A](http://www.ncbi.nlm.nih.gov/entrez/query.fcgi?cmd=search&db=gene&term=PDE7A) | immunology |
| 0.0024828 | 0.05386 | 257.1642447 | 685.0222272 | 0.3754101 | 4250202 | [STAC3](http://www.ncbi.nlm.nih.gov/entrez/query.fcgi?cmd=search&db=gene&term=STAC3) |  |
| 0.000214 | 0.020598 | 251.0735128 | 669.7130158 | 0.3748972 | 4610273 | [CLYBL](http://www.ncbi.nlm.nih.gov/entrez/query.fcgi?cmd=search&db=gene&term=CLYBL) | Citrate cycle (TCA cycle) |
| 0.0004427 | 0.026228 | 311.4471563 | 833.0511147 | 0.3738632 | 6960689 | [LOC401093](http://www.ncbi.nlm.nih.gov/entrez/query.fcgi?cmd=search&db=gene&term=LOC401093) |  |
| 9.44E-05 | 0.017191 | 128.7800318 | 344.5847941 | 0.3737252 | 4260148 |  |  |
| 0.00165 | 0.044747 | 2273.30778 | 6087.828564 | 0.3734185 | 3990092 | [ATP1A2](http://www.ncbi.nlm.nih.gov/entrez/query.fcgi?cmd=search&db=gene&term=ATP1A2) |  |
| 0.0005542 | 0.028182 | 186.1644926 | 501.5217958 | 0.3711992 | 2340632 | [INADL](http://www.ncbi.nlm.nih.gov/entrez/query.fcgi?cmd=search&db=gene&term=INADL) | Tight junction |
| 0.0011969 | 0.038713 | 354.5923672 | 955.9313953 | 0.3709391 | 3310324 | [ALKBH7](http://www.ncbi.nlm.nih.gov/entrez/query.fcgi?cmd=search&db=gene&term=ALKBH7) |  |
| 0.0029506 | 0.058427 | 416.4286494 | 1125.942434 | 0.369849 | 730647 | [ANAPC1](http://www.ncbi.nlm.nih.gov/entrez/query.fcgi?cmd=search&db=gene&term=ANAPC1) | Cell cycle, Ubiquitin mediated proteolysis |
| 0.0028648 | 0.058188 | 1458.6041 | 3957.253485 | 0.36859 | 3710154 | [SLC7A2](http://www.ncbi.nlm.nih.gov/entrez/query.fcgi?cmd=search&db=gene&term=SLC7A2) | metabolism |
| 4.42E-05 | 0.014928 | 224.8595205 | 611.8546954 | 0.3675048 | 2690132 | [APOO](http://www.ncbi.nlm.nih.gov/entrez/query.fcgi?cmd=search&db=gene&term=APOO) |  |
| 0.0040561 | 0.06788 | 240.5824966 | 655.232819 | 0.367171 | 5570348 | [SH3RF2](http://www.ncbi.nlm.nih.gov/entrez/query.fcgi?cmd=search&db=gene&term=SH3RF2) |  |
| 0.0012759 | 0.039647 | 174.5235198 | 475.8665487 | 0.3667489 | 7320349 | [KCNA5](http://www.ncbi.nlm.nih.gov/entrez/query.fcgi?cmd=search&db=gene&term=KCNA5) | immunology |
| 0.0046731 | 0.072583 | 541.2714713 | 1481.561172 | 0.3653386 | 6940608 | [PLCD4](http://www.ncbi.nlm.nih.gov/entrez/query.fcgi?cmd=search&db=gene&term=PLCD4) | Calcium signaling pathway, Inositol phosphate metabolism, Phosphatidylinositol signaling system |
| 0.0029921 | 0.058919 | 290.7672446 | 796.6361235 | 0.3649938 | 6200554 | [CYCS](http://www.ncbi.nlm.nih.gov/entrez/query.fcgi?cmd=search&db=gene&term=CYCS) | Apoptotic Signaling in Response to DNA Damage, Caspase Cascade in Apoptosis, Ceramide Signaling Pathway, D4-GDI Signaling Pathway, Electron Transport Reaction in Mitochondria, HIV-I Nef: negative effector of Fas and TNF, Induction of apoptosis through DR3 and DR4/5 Death Receptors , Opposing roles of AIF in Apoptosis and Cell Survival, Role of Mitochondria in Apoptotic Signaling, Stress Induction of HSP Regulation, Trefoil Factors Initiate Mucosal Healing, Apoptosis, Circadian rhythm, Colorectal cancer |
| 5.84E-05 | 0.01576 | 239.8408942 | 657.1991551 | 0.364944 | 7050288 | [C20orf7](http://www.ncbi.nlm.nih.gov/entrez/query.fcgi?cmd=search&db=gene&term=C20orf7) |  |
| 1.95E-05 | 0.013172 | 166.937034 | 458.9532449 | 0.3637343 | 3130184 | [RBM9](http://www.ncbi.nlm.nih.gov/entrez/query.fcgi?cmd=search&db=gene&term=RBM9) |  |
| 0.001189 | 0.038645 | 217.314484 | 597.8602661 | 0.3634871 | 7160139 | [LOC389293](http://www.ncbi.nlm.nih.gov/entrez/query.fcgi?cmd=search&db=gene&term=LOC389293) |  |
| 0.0035231 | 0.063701 | 206.3470611 | 568.3168017 | 0.3630846 | 4070025 | [POPDC2](http://www.ncbi.nlm.nih.gov/entrez/query.fcgi?cmd=search&db=gene&term=POPDC2) |  |
| 0.0003077 | 0.02341 | 102.1022889 | 281.5891301 | 0.3625931 | 6580008 | [C15orf27](http://www.ncbi.nlm.nih.gov/entrez/query.fcgi?cmd=search&db=gene&term=C15orf27) |  |
| 0.0015402 | 0.043264 | 186.3371973 | 514.7339173 | 0.3620068 | 2480475 | [SLC25A11](http://www.ncbi.nlm.nih.gov/entrez/query.fcgi?cmd=search&db=gene&term=SLC25A11) | Malate-aspartate shuttle, Shuttle for transfer of acetyl groups from mitochondria to the cytosol |
| 0.0020064 | 0.049013 | 301.0186321 | 833.268936 | 0.3612503 | 5490050 | [LRRC39](http://www.ncbi.nlm.nih.gov/entrez/query.fcgi?cmd=search&db=gene&term=LRRC39) |  |
| 0.000113 | 0.018033 | 1678.029437 | 4647.994342 | 0.3610223 | 2630008 | [COQ10A](http://www.ncbi.nlm.nih.gov/entrez/query.fcgi?cmd=search&db=gene&term=COQ10A) |  |
| 0.0047811 | 0.072968 | 4196.734801 | 11628.73751 | 0.3608934 | 1400181 | [PGAM2](http://www.ncbi.nlm.nih.gov/entrez/query.fcgi?cmd=search&db=gene&term=PGAM2) | Glycolysis / Gluconeogenesis |
| 0.0014443 | 0.041908 | 203.3247118 | 567.270844 | 0.3584262 | 5820209 | [DNAJB5](http://www.ncbi.nlm.nih.gov/entrez/query.fcgi?cmd=search&db=gene&term=DNAJB5) |  |
| 2.13E-05 | 0.013372 | 527.8590613 | 1477.430343 | 0.3572819 | 5810673 | [ATPAF1](http://www.ncbi.nlm.nih.gov/entrez/query.fcgi?cmd=search&db=gene&term=ATPAF1) |  |
| 0.0013763 | 0.041029 | 224.0136053 | 627.0460292 | 0.3572523 | 1580181 | [A2BP1](http://www.ncbi.nlm.nih.gov/entrez/query.fcgi?cmd=search&db=gene&term=A2BP1) |  |
| 0.0048964 | 0.07384 | 292.5670036 | 820.3563263 | 0.3566341 | 3130296 | [AMY2A](http://www.ncbi.nlm.nih.gov/entrez/query.fcgi?cmd=search&db=gene&term=AMY2A) | Starch and sucrose metabolism, immunology |
| 0.0029054 | 0.058373 | 1062.926477 | 3008.200716 | 0.3533429 | 1710010 |  |  |
| 0.000109 | 0.018033 | 223.6060498 | 632.8534839 | 0.3533299 | 5220554 | [A2BP1](http://www.ncbi.nlm.nih.gov/entrez/query.fcgi?cmd=search&db=gene&term=A2BP1) |  |
| 0.0036144 | 0.064294 | 3554.031811 | 10061.20322 | 0.3532412 | 5690373 | [GOT1](http://www.ncbi.nlm.nih.gov/entrez/query.fcgi?cmd=search&db=gene&term=GOT1) | Malate-aspartate shuttle, Alanine and aspartate metabolism, Alkaloid biosynthesis I, Arginine and proline metabolism, Carbon fixation, Cysteine metabolism, Glutamate metabolism, Novobiocin biosynthesis, Phenylalanine metabolism, Phenylalanine, tyrosine and tryptophan biosynthesis, Tyrosine metabolism |
| 0.0014221 | 0.041638 | 362.5051093 | 1032.833201 | 0.3509813 | 1090546 | [PPTC7](http://www.ncbi.nlm.nih.gov/entrez/query.fcgi?cmd=search&db=gene&term=PPTC7) |  |
| 0.0001541 | 0.01946 | 460.7438176 | 1313.014719 | 0.3509053 | 2070044 | [CLYBL](http://www.ncbi.nlm.nih.gov/entrez/query.fcgi?cmd=search&db=gene&term=CLYBL) | Citrate cycle (TCA cycle) |
| 0.004626 | 0.072259 | 293.0283783 | 846.9213757 | 0.3459924 | 6200097 | [TMEM182](http://www.ncbi.nlm.nih.gov/entrez/query.fcgi?cmd=search&db=gene&term=TMEM182) |  |
| 0.0006605 | 0.029691 | 726.8828187 | 2105.042306 | 0.3453056 | 3890008 | [CYCSL1](http://www.ncbi.nlm.nih.gov/entrez/query.fcgi?cmd=search&db=gene&term=CYCSL1) |  |
| 0.0022524 | 0.051764 | 307.8486176 | 895.8134515 | 0.3436526 | 5050537 | [OPLAH](http://www.ncbi.nlm.nih.gov/entrez/query.fcgi?cmd=search&db=gene&term=OPLAH) | Glutathione metabolism |
| 0.0010461 | 0.036768 | 220.8099885 | 643.7854806 | 0.3429869 | 4070091 | [RORC](http://www.ncbi.nlm.nih.gov/entrez/query.fcgi?cmd=search&db=gene&term=RORC) |  |
| 0.0016415 | 0.044558 | 442.5366291 | 1297.471532 | 0.3410762 | 6180678 | [TMEM143](http://www.ncbi.nlm.nih.gov/entrez/query.fcgi?cmd=search&db=gene&term=TMEM143) |  |
| 0.000712 | 0.030455 | 266.7961936 | 783.7581483 | 0.3404063 | 3850349 | [VGLL2](http://www.ncbi.nlm.nih.gov/entrez/query.fcgi?cmd=search&db=gene&term=VGLL2) |  |
| 0.0025851 | 0.055088 | 165.0630352 | 485.7700469 | 0.3397967 | 6980543 | [LMO1](http://www.ncbi.nlm.nih.gov/entrez/query.fcgi?cmd=search&db=gene&term=LMO1) | misc |
| 0.0015417 | 0.043264 | 444.4332235 | 1310.161831 | 0.3392201 | 4560543 | [TSPAN8](http://www.ncbi.nlm.nih.gov/entrez/query.fcgi?cmd=search&db=gene&term=TSPAN8) |  |
| 0.0012033 | 0.038833 | 528.33042 | 1558.860415 | 0.3389209 | 2640341 | [FKBP5](http://www.ncbi.nlm.nih.gov/entrez/query.fcgi?cmd=search&db=gene&term=FKBP5) |  |
| 8.24E-05 | 0.016957 | 141.7352496 | 419.5866773 | 0.3377973 | 2760500 | [CD38](http://www.ncbi.nlm.nih.gov/entrez/query.fcgi?cmd=search&db=gene&term=CD38) | Calcium signaling pathway, Hematopoietic cell lineage, Nicotinate and nicotinamide metabolism, angiogenesis, metastasis |
| 0.0018751 | 0.047482 | 368.9259515 | 1093.035871 | 0.3375241 | 3890288 | [TRIM63](http://www.ncbi.nlm.nih.gov/entrez/query.fcgi?cmd=search&db=gene&term=TRIM63) |  |
| 0.0012762 | 0.039647 | 235.3129718 | 702.9985535 | 0.3347275 | 6560152 | [PPP2R3A](http://www.ncbi.nlm.nih.gov/entrez/query.fcgi?cmd=search&db=gene&term=PPP2R3A) | Tight junction |
| 9.47E-05 | 0.017191 | 119.8239329 | 359.8005499 | 0.3330288 | 6840392 | [RGS9BP](http://www.ncbi.nlm.nih.gov/entrez/query.fcgi?cmd=search&db=gene&term=RGS9BP) |  |
| 0.0010942 | 0.03739 | 123.6830052 | 371.8849365 | 0.3325841 | 4920079 | [OR7E37P](http://www.ncbi.nlm.nih.gov/entrez/query.fcgi?cmd=search&db=gene&term=OR7E37P) |  |
| 0.0002281 | 0.020993 | 323.9385788 | 975.3312928 | 0.3321318 | 2760181 | [COQ3](http://www.ncbi.nlm.nih.gov/entrez/query.fcgi?cmd=search&db=gene&term=COQ3) | Ubiquinone biosynthesis |
| 0.0029249 | 0.058389 | 183.4978647 | 554.0123395 | 0.3312162 | 1990730 | [CCDC21](http://www.ncbi.nlm.nih.gov/entrez/query.fcgi?cmd=search&db=gene&term=CCDC21) |  |
| 0.0020625 | 0.049303 | 386.3033182 | 1168.362957 | 0.3306364 | 2470196 | [DNAJB5](http://www.ncbi.nlm.nih.gov/entrez/query.fcgi?cmd=search&db=gene&term=DNAJB5) |  |
| 0.0002066 | 0.02048 | 1878.589843 | 5683.468289 | 0.3305358 | 7050370 | [ANKRD9](http://www.ncbi.nlm.nih.gov/entrez/query.fcgi?cmd=search&db=gene&term=ANKRD9) |  |
| 8.82E-05 | 0.017191 | 709.3282009 | 2156.897406 | 0.3288651 | 460180 | [SLC25A12](http://www.ncbi.nlm.nih.gov/entrez/query.fcgi?cmd=search&db=gene&term=SLC25A12) |  |
| 0.0003994 | 0.025361 | 917.6439477 | 2800.426864 | 0.32768 | 160189 | [RYR1](http://www.ncbi.nlm.nih.gov/entrez/query.fcgi?cmd=search&db=gene&term=RYR1) | Calcium signaling pathway, Long-term depression, immunology |
| 0.0002313 | 0.021126 | 231.7644958 | 708.6015563 | 0.3270731 | 4640181 | [DYRK1B](http://www.ncbi.nlm.nih.gov/entrez/query.fcgi?cmd=search&db=gene&term=DYRK1B) | signal_transduction |
| 9.63E-05 | 0.017266 | 444.4388789 | 1359.970773 | 0.3268003 | 2350037 | [HRASLS](http://www.ncbi.nlm.nih.gov/entrez/query.fcgi?cmd=search&db=gene&term=HRASLS) |  |
| 0.0004294 | 0.026147 | 528.7935482 | 1619.217236 | 0.3265736 | 6040279 | [C20orf7](http://www.ncbi.nlm.nih.gov/entrez/query.fcgi?cmd=search&db=gene&term=C20orf7) |  |
| 0.0016713 | 0.045114 | 218.3722158 | 673.8107818 | 0.3240854 | 7050064 | [RAMP1](http://www.ncbi.nlm.nih.gov/entrez/query.fcgi?cmd=search&db=gene&term=RAMP1) |  |
| 0.0005008 | 0.027607 | 547.3436052 | 1699.292644 | 0.3221009 | 3190402 | [KLHDC6](http://www.ncbi.nlm.nih.gov/entrez/query.fcgi?cmd=search&db=gene&term=KLHDC6) |  |
| 0.0017208 | 0.045525 | 202.6681804 | 633.6086183 | 0.3198634 | 5420152 | [AMHR2](http://www.ncbi.nlm.nih.gov/entrez/query.fcgi?cmd=search&db=gene&term=AMHR2) | Cytokine-cytokine receptor interaction, TGF-beta signaling pathway, development |
| 0.0003469 | 0.024601 | 1080.597365 | 3379.552879 | 0.3197457 | 4570017 | [ASB10](http://www.ncbi.nlm.nih.gov/entrez/query.fcgi?cmd=search&db=gene&term=ASB10) |  |
| 0.001581 | 0.043775 | 238.4552379 | 746.660285 | 0.3193624 | 6380066 | [COL4A3](http://www.ncbi.nlm.nih.gov/entrez/query.fcgi?cmd=search&db=gene&term=COL4A3) | immunology |
| 0.002576 | 0.055015 | 158.8670919 | 497.6527414 | 0.3192328 | 2030747 | [ASB15](http://www.ncbi.nlm.nih.gov/entrez/query.fcgi?cmd=search&db=gene&term=ASB15) |  |
| 0.0004706 | 0.026828 | 425.3179795 | 1336.304621 | 0.3182792 | 3990671 | [DENND2C](http://www.ncbi.nlm.nih.gov/entrez/query.fcgi?cmd=search&db=gene&term=DENND2C) |  |
| 0.0026588 | 0.055879 | 732.1782228 | 2300.684047 | 0.3182437 | 5130577 | [SLC37A4](http://www.ncbi.nlm.nih.gov/entrez/query.fcgi?cmd=search&db=gene&term=SLC37A4) |  |
| 0.0026526 | 0.05581 | 200.5034139 | 631.1162086 | 0.3176965 | 270286 | [GADL1](http://www.ncbi.nlm.nih.gov/entrez/query.fcgi?cmd=search&db=gene&term=GADL1) |  |
| 3.96E-05 | 0.014558 | 393.0475579 | 1237.984661 | 0.3174898 | 870451 | [ASB8](http://www.ncbi.nlm.nih.gov/entrez/query.fcgi?cmd=search&db=gene&term=ASB8) |  |
| 0.0001673 | 0.019753 | 115.4681301 | 367.2113104 | 0.314446 | 6110017 | [C20orf26](http://www.ncbi.nlm.nih.gov/entrez/query.fcgi?cmd=search&db=gene&term=C20orf26) |  |
| 0.0009801 | 0.035584 | 487.7794545 | 1562.009082 | 0.312277 | 5130414 | [C9orf23](http://www.ncbi.nlm.nih.gov/entrez/query.fcgi?cmd=search&db=gene&term=C9orf23) |  |
| 9.50E-06 | 0.012163 | 625.6373798 | 2011.010715 | 0.3111059 | 6450523 | [FKBP3](http://www.ncbi.nlm.nih.gov/entrez/query.fcgi?cmd=search&db=gene&term=FKBP3) |  |
| 0.0039154 | 0.066776 | 502.369141 | 1615.834684 | 0.3109038 | 3420519 | [PPP2R3A](http://www.ncbi.nlm.nih.gov/entrez/query.fcgi?cmd=search&db=gene&term=PPP2R3A) | Tight junction |
| 5.80E-06 | 0.012163 | 272.9048383 | 879.3815414 | 0.3103372 | 4890553 | [RORC](http://www.ncbi.nlm.nih.gov/entrez/query.fcgi?cmd=search&db=gene&term=RORC) |  |
| 5.36E-05 | 0.015414 | 146.5310664 | 472.4700206 | 0.3101383 | 2850255 | [ALDOA](http://www.ncbi.nlm.nih.gov/entrez/query.fcgi?cmd=search&db=gene&term=ALDOA) | Downregulated of MTA-3 in ER-negative Breast Tumors, Carbon fixation, Fructose and mannose metabolism, Glycolysis / Gluconeogenesis, Pentose phosphate pathway, immunology |
| 0.0018914 | 0.047687 | 188.6927468 | 611.3444818 | 0.3086521 | 7040433 | [L2HGDH](http://www.ncbi.nlm.nih.gov/entrez/query.fcgi?cmd=search&db=gene&term=L2HGDH) | Butanoate metabolism |
| 0.0008625 | 0.033634 | 174.8687921 | 569.3613006 | 0.3071315 | 3610041 | [RASA4](http://www.ncbi.nlm.nih.gov/entrez/query.fcgi?cmd=search&db=gene&term=RASA4) |  |
| 0.0019295 | 0.048229 | 4741.600877 | 15528.54031 | 0.3053475 | 6960615 | [RPL3L](http://www.ncbi.nlm.nih.gov/entrez/query.fcgi?cmd=search&db=gene&term=RPL3L) | Ribosome |
| 0.0022347 | 0.051432 | 246.4028887 | 813.8451858 | 0.3027638 | 5910239 | [UCP3](http://www.ncbi.nlm.nih.gov/entrez/query.fcgi?cmd=search&db=gene&term=UCP3) | immunology |
| 0.000555 | 0.028182 | 249.348309 | 824.4071359 | 0.3024577 | 1690543 | [LOC145814](http://www.ncbi.nlm.nih.gov/entrez/query.fcgi?cmd=search&db=gene&term=LOC145814) |  |
| 0.0041422 | 0.068397 | 201.8913678 | 669.7519749 | 0.301442 | 840630 | [USP6](http://www.ncbi.nlm.nih.gov/entrez/query.fcgi?cmd=search&db=gene&term=USP6) | tsonc |
| 0.0023119 | 0.052324 | 158.4542562 | 530.1911527 | 0.2988625 | 610035 | [KCNA7](http://www.ncbi.nlm.nih.gov/entrez/query.fcgi?cmd=search&db=gene&term=KCNA7) |  |
| 0.0031217 | 0.060126 | 191.9870212 | 654.6044281 | 0.2932871 | 2100079 | [DCI](http://www.ncbi.nlm.nih.gov/entrez/query.fcgi?cmd=search&db=gene&term=DCI) | Fatty acid metabolism |
| 0.000481 | 0.02718 | 469.7083137 | 1603.870259 | 0.2928593 | 6020575 | [NNT](http://www.ncbi.nlm.nih.gov/entrez/query.fcgi?cmd=search&db=gene&term=NNT) | Nicotinate and nicotinamide metabolism |
| 0.0038978 | 0.0666 | 529.1744347 | 1813.445568 | 0.2918061 | 4760474 | [TUBA4A](http://www.ncbi.nlm.nih.gov/entrez/query.fcgi?cmd=search&db=gene&term=TUBA4A) | Gap junction |
| 0.0004485 | 0.026228 | 840.6766155 | 2895.124023 | 0.2903767 | 3190278 | [LDB3](http://www.ncbi.nlm.nih.gov/entrez/query.fcgi?cmd=search&db=gene&term=LDB3) |  |
| 0.0040241 | 0.067639 | 919.5007953 | 3219.32145 | 0.2856194 | 5860601 | [PPP1R3A](http://www.ncbi.nlm.nih.gov/entrez/query.fcgi?cmd=search&db=gene&term=PPP1R3A) | Insulin signaling pathway |
| 0.0012903 | 0.039869 | 154.2714879 | 549.1168282 | 0.2809447 | 5890121 | [NRG4](http://www.ncbi.nlm.nih.gov/entrez/query.fcgi?cmd=search&db=gene&term=NRG4) |  |
| 0.000183 | 0.01994 | 702.6055206 | 2534.727258 | 0.2771918 | 630546 | [MN1](http://www.ncbi.nlm.nih.gov/entrez/query.fcgi?cmd=search&db=gene&term=MN1) |  |
| 0.0026756 | 0.056192 | 407.3038977 | 1484.278709 | 0.274412 | 770026 | [TMEM38A](http://www.ncbi.nlm.nih.gov/entrez/query.fcgi?cmd=search&db=gene&term=TMEM38A) |  |
| 0.0001756 | 0.019771 | 110.2466645 | 404.6406437 | 0.2724557 | 5390168 | [SH2D1B](http://www.ncbi.nlm.nih.gov/entrez/query.fcgi?cmd=search&db=gene&term=SH2D1B) | Natural killer cell mediated cytotoxicity |
| 0.0030135 | 0.059206 | 182.6680613 | 675.6103131 | 0.2703749 | 3830056 | [PHKG1](http://www.ncbi.nlm.nih.gov/entrez/query.fcgi?cmd=search&db=gene&term=PHKG1) | Calcium signaling pathway, Insulin signaling pathway |
| 0.0036165 | 0.064294 | 304.4397382 | 1144.117791 | 0.2660913 | 1740594 | [RBM24](http://www.ncbi.nlm.nih.gov/entrez/query.fcgi?cmd=search&db=gene&term=RBM24) |  |
| 0.0028093 | 0.057462 | 256.7515097 | 966.6489097 | 0.2656099 | 4900703 | [ADSSL1](http://www.ncbi.nlm.nih.gov/entrez/query.fcgi?cmd=search&db=gene&term=ADSSL1) | Alanine and aspartate metabolism, Purine metabolism |
| 0.0011122 | 0.037694 | 355.9143133 | 1351.72985 | 0.2633028 | 10487 | [SLC25A25](http://www.ncbi.nlm.nih.gov/entrez/query.fcgi?cmd=search&db=gene&term=SLC25A25) |  |
| 0.0029448 | 0.058427 | 850.6269622 | 3280.461263 | 0.259301 | 6770189 | [RTN4](http://www.ncbi.nlm.nih.gov/entrez/query.fcgi?cmd=search&db=gene&term=RTN4) |  |
| 0.0016783 | 0.045177 | 165.5739946 | 641.0092411 | 0.258302 | 3930672 | [C10orf92](http://www.ncbi.nlm.nih.gov/entrez/query.fcgi?cmd=search&db=gene&term=C10orf92) |  |
| 2.23E-05 | 0.013372 | 1042.007927 | 4043.836551 | 0.2576781 | 6180711 | [SLC36A2](http://www.ncbi.nlm.nih.gov/entrez/query.fcgi?cmd=search&db=gene&term=SLC36A2) |  |
| 0.0012954 | 0.039905 | 286.2639507 | 1127.406098 | 0.2539138 | 150349 | [PHKB](http://www.ncbi.nlm.nih.gov/entrez/query.fcgi?cmd=search&db=gene&term=PHKB) | Calcium signaling pathway, Insulin signaling pathway |
| 0.0001516 | 0.019292 | 316.3243177 | 1247.483536 | 0.2535699 | 6330397 | [TRIM7](http://www.ncbi.nlm.nih.gov/entrez/query.fcgi?cmd=search&db=gene&term=TRIM7) |  |
| 0.0018143 | 0.046593 | 746.8784747 | 3016.239635 | 0.2476191 | 6560154 | [SCN1B](http://www.ncbi.nlm.nih.gov/entrez/query.fcgi?cmd=search&db=gene&term=SCN1B) | immunology |
| 0.0016344 | 0.044476 | 287.9358918 | 1178.711994 | 0.2442801 | 990086 | [KCNT1](http://www.ncbi.nlm.nih.gov/entrez/query.fcgi?cmd=search&db=gene&term=KCNT1) |  |
| 0.0002312 | 0.021126 | 549.6876485 | 2300.082635 | 0.238986 | 2600750 | [PHKG1](http://www.ncbi.nlm.nih.gov/entrez/query.fcgi?cmd=search&db=gene&term=PHKG1) | Calcium signaling pathway, Insulin signaling pathway |
| 1.01E-05 | 0.012163 | 611.3704471 | 2564.627713 | 0.2383857 | 6480259 | [MN1](http://www.ncbi.nlm.nih.gov/entrez/query.fcgi?cmd=search&db=gene&term=MN1) |  |
| 0.000491 | 0.027279 | 1751.303964 | 7668.010629 | 0.2283909 | 4540403 | [C20orf166](http://www.ncbi.nlm.nih.gov/entrez/query.fcgi?cmd=search&db=gene&term=C20orf166) |  |
| 0.0001153 | 0.018034 | 243.8271617 | 1073.311742 | 0.2271727 | 580390 | [PPARGC1A](http://www.ncbi.nlm.nih.gov/entrez/query.fcgi?cmd=search&db=gene&term=PPARGC1A) | CARM1 and Regulation of the Estrogen Receptor, Mechanism of Gene Regulation by Peroxisome Proliferators via PPARa(alpha), Regulation of PGC-1a, Role of PPAR-gamma Coactivators in Obesity and Thermogenesis, Adipocytokine signaling pathway, Insulin signaling pathway |
| 0.0004906 | 0.027279 | 142.6508028 | 631.152401 | 0.2260164 | 2120520 | [UGP2](http://www.ncbi.nlm.nih.gov/entrez/query.fcgi?cmd=search&db=gene&term=UGP2) | Galactose metabolism, Nucleotide sugars metabolism, Pentose and glucuronate interconversions, Starch and sucrose metabolism |
| 3.54E-05 | 0.014558 | 198.7515428 | 902.2256095 | 0.2202903 | 2970209 | [SLC47A1](http://www.ncbi.nlm.nih.gov/entrez/query.fcgi?cmd=search&db=gene&term=SLC47A1) |  |
| 0.003147 | 0.060373 | 223.3693991 | 1046.017063 | 0.2135428 | 2060674 | [CA2](http://www.ncbi.nlm.nih.gov/entrez/query.fcgi?cmd=search&db=gene&term=CA2) | Nitrogen metabolism, immunology |
| 0.0011814 | 0.038641 | 475.3469349 | 2510.162063 | 0.189369 | 460358 | [CA2](http://www.ncbi.nlm.nih.gov/entrez/query.fcgi?cmd=search&db=gene&term=CA2) | Nitrogen metabolism, immunology |
| 0.0007815 | 0.032019 | 371.620825 | 2019.555946 | 0.1840112 | 110719 | [SLC16A3](http://www.ncbi.nlm.nih.gov/entrez/query.fcgi?cmd=search&db=gene&term=SLC16A3) |  |
| 0.0010888 | 0.037337 | 1447.661292 | 7992.037299 | 0.181138 | 7040497 | [ART3](http://www.ncbi.nlm.nih.gov/entrez/query.fcgi?cmd=search&db=gene&term=ART3) |  |
| 0.0011317 | 0.037913 | 1198.573179 | 6881.534801 | 0.1741724 | 4290168 | [MYLK2](http://www.ncbi.nlm.nih.gov/entrez/query.fcgi?cmd=search&db=gene&term=MYLK2) | Calcium signaling pathway, Focal adhesion, Regulation of actin cytoskeleton |
| 0.0002691 | 0.022558 | 181.2382823 | 1049.777546 | 0.1726445 | 6660025 | [ZMYND17](http://www.ncbi.nlm.nih.gov/entrez/query.fcgi?cmd=search&db=gene&term=ZMYND17) |  |
| 0.0014393 | 0.041807 | 569.655038 | 3483.052845 | 0.1635505 | 6660437 | [PEBP4](http://www.ncbi.nlm.nih.gov/entrez/query.fcgi?cmd=search&db=gene&term=PEBP4) |  |
| 0.0023293 | 0.052324 | 186.255365 | 1204.743401 | 0.1546017 | 2750176 | [AQP4](http://www.ncbi.nlm.nih.gov/entrez/query.fcgi?cmd=search&db=gene&term=AQP4) |  |
| 0.0001237 | 0.018034 | 481.5505263 | 3736.772033 | 0.1288681 | 2680523 | [SAMD4A](http://www.ncbi.nlm.nih.gov/entrez/query.fcgi?cmd=search&db=gene&term=SAMD4A) |  |
|  |  |  |  |  |  |  |  |
| **Genes which did not meet the filtering criteria but are cited in text, figure or tables** | | | | | |  |  |
| 0.010702 | 0.104766 | 338.9937558 | 117.3713094 | 2.8882165 | 670594 | [C2](http://www.ncbi.nlm.nih.gov/entrez/query.fcgi?cmd=search&db=gene&term=C2) | Classical Complement Pathway, Complement Pathway, Lectin Induced Complement Pathway, Complement and coagulation cascades, immunology |
| 0.00619 | 0.081763 | 159.178549 | 98.6990788 | 1.6127663 | 4920221 | [C7](http://www.ncbi.nlm.nih.gov/entrez/query.fcgi?cmd=search&db=gene&term=C7) | Alternative Complement Pathway, Cells and Molecules involved in local acute inflammatory response, Classical Complement Pathway, Complement Pathway, Lectin Induced Complement Pathway, Complement and coagulation cascades, immunology |
| 0.0078847 | 0.091495 | 11129.95493 | 3772.149727 | 2.9505602 | 1240152 | [CFD](http://www.ncbi.nlm.nih.gov/entrez/query.fcgi?cmd=search&db=gene&term=CFD) | Alternative Complement Pathway, Complement Pathway, Complement and coagulation cascades |
| 0.005662 | 0.078417 | 239.8144981 | 117.6883197 | 2.0377086 | 7200753 | [TLR7](http://www.ncbi.nlm.nih.gov/entrez/query.fcgi?cmd=search&db=gene&term=TLR7) | Dendritic cells in regulating TH1 and TH2 Development, Toll-Like Receptor Pathway, Toll-like receptor signaling pathway |
| 0.0100603 | 0.101424 | 154.8699117 | 105.5070577 | 1.4678631 | 4670193 | [PRF1](http://www.ncbi.nlm.nih.gov/entrez/query.fcgi?cmd=search&db=gene&term=PRF1) | Caspase Cascade in Apoptosis, CTL mediated immune response against target cells , D4-GDI Signaling Pathway, Granzyme A mediated Apoptosis Pathway, Natural killer cell mediated cytotoxicity, Type I diabetes mellitus, immunology |
| 0.0210205 | 0.149125 | 113.7746963 | 103.9500991 | 1.0945126 | 4060600 | [SELE](http://www.ncbi.nlm.nih.gov/entrez/query.fcgi?cmd=search&db=gene&term=SELE) | Adhesion and Diapedesis of Granulocytes, Adhesion and Diapedesis of Lymphocytes, Adhesion Molecules on Lymphocyte, Monocyte and its Surface Molecules, Neutrophil and Its Surface Molecules, Cell adhesion molecules (CAMs), cell_signaling, immunology, metastasis |
| 0.0363842 | 0.200947 | 184.5334992 | 112.0244627 | 1.6472607 | 4180494 | [ITGAL](http://www.ncbi.nlm.nih.gov/entrez/query.fcgi?cmd=search&db=gene&term=ITGAL) | Adhesion and Diapedesis of Granulocytes, Adhesion and Diapedesis of Lymphocytes, Adhesion Molecules on Lymphocyte, B Lymphocyte Cell Surface Molecules, Cells and Molecules involved in local acute inflammatory response, CTL mediated immune response against target cells , Monocyte and its Surface Molecules, Neutrophil and Its Surface Molecules, T Cytotoxic Cell Surface Molecules, T Helper Cell Surface Molecules, Cell adhesion molecules (CAMs), Leukocyte transendothelial migration, Natural killer cell mediated cytotoxicity, Regulation of actin cytoskeleton, cell_signaling, immunology, metastasis |
| 0.0237081 | 0.158154 | 167.0578988 | 109.4621868 | 1.5261699 | 1850523 | [GZMB](http://www.ncbi.nlm.nih.gov/entrez/query.fcgi?cmd=search&db=gene&term=GZMB) | Apoptotic DNA fragmentation and tissue homeostasis, Caspase Cascade in Apoptosis, CTL mediated immune response against target cells , D4-GDI Signaling Pathway, Granzyme A mediated Apoptosis Pathway, Natural killer cell mediated cytotoxicity, Type I diabetes mellitus, immunology, misc |
| 0.0174263 | 0.135696 | 13886.69327 | 5483.097504 | 2.5326366 | 4890487 | [B2M](http://www.ncbi.nlm.nih.gov/entrez/query.fcgi?cmd=search&db=gene&term=B2M) | Antigen Processing and Presentation, CTL mediated immune response against target cells , Ras-Independent pathway in NK cell-mediated cytotoxicity, Antigen processing and presentation, immunology |
| 0.0067066 | 0.084512 | 346.2747765 | 138.7799737 | 2.495135 | 3890689 | [CD247](http://www.ncbi.nlm.nih.gov/entrez/query.fcgi?cmd=search&db=gene&term=CD247) | Activation of Csk by cAMP-dependent Protein Kinase Inhibits Signaling through the T Cell Receptor, CTL mediated immune response against target cells , HIV Induced T Cell Apoptosis, IL 17 Signaling Pathway, IL12 and Stat4 Dependent Signaling Pathway in Th1 Development, Lck and Fyn tyrosine kinases in initiation of TCR Activation, NO2-dependent IL 12 Pathway in NK cells, Role of Tob in T-cell activation, Stathmin and breast cancer resistance to antimicrotubule agents, T Cell Receptor and CD3 Complex, T Cell Receptor Signaling Pathway, T Cytotoxic Cell Surface Molecules, T Helper Cell Surface Molecules, The Co-Stimulatory Signal During T-cell Activation, Natural killer cell mediated cytotoxicity, T cell receptor signaling pathway |
| 0.0238058 | 0.158514 | 218.8979463 | 113.8017184 | 1.923503 | 6770711 | [CD4](http://www.ncbi.nlm.nih.gov/entrez/query.fcgi?cmd=search&db=gene&term=CD4) | Activation of Csk by cAMP-dependent Protein Kinase Inhibits Signaling through the T Cell Receptor, Antigen Dependent B Cell Activation, Bystander B Cell Activation, Cytokines and Inflammatory Response, HIV Induced T Cell Apoptosis, HIV-1 defeats host-mediated resistance by CEM15, IL 17 Signaling Pathway, IL 5 Signaling Pathway, Lck and Fyn tyrosine kinases in initiation of TCR Activation, NO2-dependent IL 12 Pathway in NK cells, Regulation of hematopoiesis by cytokines, Selective expression of chemokine receptors during T-cell polarization, T Helper Cell Surface Molecules, Antigen processing and presentation, Cell adhesion molecules (CAMs), Hematopoietic cell lineage, T cell receptor signaling pathway, angiogenesis, immunology, metastasis |
| 0.0087341 | 0.095083 | 483.3657913 | 141.6860697 | 3.4115266 | 1170671 | [CD3D](http://www.ncbi.nlm.nih.gov/entrez/query.fcgi?cmd=search&db=gene&term=CD3D) | Activation of Csk by cAMP-dependent Protein Kinase Inhibits Signaling through the T Cell Receptor, CTL mediated immune response against target cells , HIV Induced T Cell Apoptosis, IL 17 Signaling Pathway, IL12 and Stat4 Dependent Signaling Pathway in Th1 Development, Lck and Fyn tyrosine kinases in initiation of TCR Activation, Role of Tob in T-cell activation, T Cell Receptor and CD3 Complex, T Cell Receptor Signaling Pathway, T Cytotoxic Cell Surface Molecules, T Helper Cell Surface Molecules, The Co-Stimulatory Signal During T-cell Activation, Hematopoietic cell lineage, T cell receptor signaling pathway |
| 0.0910687 | 0.326031 | 160.7012316 | 94.9882785 | 1.6918007 | 1780600 | [CD3E](http://www.ncbi.nlm.nih.gov/entrez/query.fcgi?cmd=search&db=gene&term=CD3E) | Activation of Csk by cAMP-dependent Protein Kinase Inhibits Signaling through the T Cell Receptor, CTL mediated immune response against target cells , HIV Induced T Cell Apoptosis, IL 17 Signaling Pathway, IL12 and Stat4 Dependent Signaling Pathway in Th1 Development, Lck and Fyn tyrosine kinases in initiation of TCR Activation, Role of Tob in T-cell activation, T Cell Receptor and CD3 Complex, T Cell Receptor Signaling Pathway, T Cytotoxic Cell Surface Molecules, T Helper Cell Surface Molecules, The Co-Stimulatory Signal During T-cell Activation, Hematopoietic cell lineage, T cell receptor signaling pathway, immunology |
| 0.0691623 | 0.281607 | 112.6003429 | 98.4882409 | 1.1432872 | 4890722 | [CD28](http://www.ncbi.nlm.nih.gov/entrez/query.fcgi?cmd=search&db=gene&term=CD28) | Antigen Dependent B Cell Activation, Bystander B Cell Activation, HIV Induced T Cell Apoptosis, Role of Tob in T-cell activation, Selective expression of chemokine receptors during T-cell polarization, T Cytotoxic Cell Surface Molecules, T Helper Cell Surface Molecules, Th1/Th2 Differentiation, The Co-Stimulatory Signal During T-cell Activation, Cell adhesion molecules (CAMs), T cell receptor signaling pathway, Type I diabetes mellitus |
| 0.0721854 | 0.288156 | 646.0497395 | 281.3329542 | 2.2963884 | 1090326 | [TIMP4](http://www.ncbi.nlm.nih.gov/entrez/query.fcgi?cmd=search&db=gene&term=TIMP4) | Inhibition of Matrix Metalloproteinases, misc |
| 0.0092873 | 0.098063 | 176.8126354 | 126.0157306 | 1.4030997 | 510736 | [MMP2](http://www.ncbi.nlm.nih.gov/entrez/query.fcgi?cmd=search&db=gene&term=MMP2) | Inhibition of Matrix Metalloproteinases, GnRH signaling pathway, Leukocyte transendothelial migration, angiogenesis, immunology |
| 0.0247974 | 0.161856 | 565.2124414 | 101.2463436 | 5.5825467 | 3180528 | [MMP9](http://www.ncbi.nlm.nih.gov/entrez/query.fcgi?cmd=search&db=gene&term=MMP9) | Inhibition of Matrix Metalloproteinases, Leukocyte transendothelial migration, angiogenesis, immunology |
| 0.2514595 | 0.539173 | 238.0144657 | 275.5030968 | 0.8639266 | 450376 | [TSC2](http://www.ncbi.nlm.nih.gov/entrez/query.fcgi?cmd=search&db=gene&term=TSC2) | Control of Gene Expression by Vitamin D Receptor, mTOR Signaling Pathway, Insulin signaling pathway, mTOR signaling pathway, misc |
| 0.0883172 | 0.320567 | 105.8494799 | 122.8240878 | 0.8617974 | 3890176 | [EIF4EBP1](http://www.ncbi.nlm.nih.gov/entrez/query.fcgi?cmd=search&db=gene&term=EIF4EBP1) | mTOR Signaling Pathway, Regulation of eIF4e and p70 S6 Kinase, Skeletal muscle hypertrophy is regulated via AKT/mTOR pathway, Insulin signaling pathway, mTOR signaling pathway |
| 0.0051869 | 0.075855 | 6913.125606 | 418.4086434 | 16.522425 | 1070541 | [MYH3](http://www.ncbi.nlm.nih.gov/entrez/query.fcgi?cmd=search&db=gene&term=MYH3) |  |
| 0.0083843 | 0.093885 | 19656.67319 | 1343.055142 | 14.63579 | 4260201 | [ACTC1](http://www.ncbi.nlm.nih.gov/entrez/query.fcgi?cmd=search&db=gene&term=ACTC1) | Adherens junction, Cell Communication, Focal adhesion, Leukocyte transendothelial migration, Regulation of actin cytoskeleton, Tight junction |
| 0.0223536 | 0.153236 | 716.7646196 | 108.7504678 | 6.5909107 | 7400279 | [CCNA1](http://www.ncbi.nlm.nih.gov/entrez/query.fcgi?cmd=search&db=gene&term=CCNA1) | Cell Cycle: G1/S Check Point , Cyclins and Cell Cycle Regulation, E2F1 Destruction Pathway, Cell cycle |
| 0,0195502 | 0,1437195 | 945,5797329 | 121,8466698 | 7,7604069 | 5570278 | [CXCL9](http://www.ncbi.nlm.nih.gov/entrez/query.fcgi?cmd=search&db=gene&term=CXCL9) | Cytokine-cytokine receptor interaction, Toll-like receptor signaling pathway |
| 0,0130429 | 0,1162415 | 814,4269516 | 124,7151211 | 6,5302984 | 6270553 | [CXCL10](http://www.ncbi.nlm.nih.gov/entrez/query.fcgi?cmd=search&db=gene&term=CXCL10) | Cytokine-cytokine receptor interaction, Toll-like receptor signaling pathway |
