# Supplementary material for: Different Molecular Signatures in Magnetic Resonance Imaging-Staged Facioscapulohumeral Muscular Dystrophy Muscles
Source: PLoS One. 2012 Jun 13;7(6):e38779. doi: 10.1371/journal.pone.0038779 (PMC3374833; doi:10.1371/journal.pone.0038779)
Supplement: Table S2 — BioCarta, Gene Ontology, and KEGG gene set expression comparison of T2-STIR + vs. T2-STIR – FSHD muscles. (DOC) [file pone.0038779.s006.doc]

**Table S2: BioCarta, Gene Ontology, KEGG Gene Set Expression Comparison FSHD T2-STIR + vs. FSHD T2-STIR –**

| BioCarta Gene Set Expression Comparison FSHD T2-STIR + vs. FSHD T2-STIR - | | | | | | | |
| --- | --- | --- | --- | --- | --- | --- | --- |
|  | **Biocarta Pathway** | **Pathway description** | **Number of genes** | **LS permutation p-value** | **KS permutation p-value** | **Efron-Tibshirani's GSA test p-value** | **Goeman's global test p-value** |
| 1 | h_classicPathway | [Classical Complement Pathway](http://cgap.nci.nih.gov/Pathways/BioCarta/h_classicPathway) | [9](../../../../C:%5CDocuments%20and%20Settings%5CAdministrator%5CDesktop%5CDystro2009_all%20-Project%5COutput%5CFshdinf%20vs%20fshdPathwayClassComparison_Bioc_filtered%5CGeneSetGenesTable1.html#h_classicPathway) | 0.00001 | 0.00018 | < 0.005 (-) | 0.0000055 |
| 2 | h_compPathway | [Complement Pathway](http://cgap.nci.nih.gov/Pathways/BioCarta/h_compPathway) | [13](../../../../C:%5CDocuments%20and%20Settings%5CAdministrator%5CDesktop%5CDystro2009_all%20-Project%5COutput%5CFshdinf%20vs%20fshdPathwayClassComparison_Bioc_filtered%5CGeneSetGenesTable1.html#h_compPathway) | 0.00001 | 0.00058 | < 0.005 (-) | 0.0000037 |
| 3 | h_reckPathway | [Inhibition of Matrix Metalloproteinases](http://cgap.nci.nih.gov/Pathways/BioCarta/h_reckPathway) | [8](../../../../C:%5CDocuments%20and%20Settings%5CAdministrator%5CDesktop%5CDystro2009_all%20-Project%5COutput%5CFshdinf%20vs%20fshdPathwayClassComparison_Bioc_filtered%5CGeneSetGenesTable1.html#h_reckPathway) | 0.00008 | 0.00006 | < 0.005 (-) | 0.0000597 |
| 4 | h_neutrophilPathway | [Neutrophil and Its Surface Molecules](http://cgap.nci.nih.gov/Pathways/BioCarta/h_neutrophilPathway) | [6](../../../../C:%5CDocuments%20and%20Settings%5CAdministrator%5CDesktop%5CDystro2009_all%20-Project%5COutput%5CFshdinf%20vs%20fshdPathwayClassComparison_Bioc_filtered%5CGeneSetGenesTable1.html#h_neutrophilPathway) | 0.00009 | 0.00008 | < 0.005 (-) | 0.000117 |
| 5 | h_alternativePathway | [Alternative Complement Pathway](http://cgap.nci.nih.gov/Pathways/BioCarta/h_alternativePathway) | [7](../../../../C:%5CDocuments%20and%20Settings%5CAdministrator%5CDesktop%5CDystro2009_all%20-Project%5COutput%5CFshdinf%20vs%20fshdPathwayClassComparison_Bioc_filtered%5CGeneSetGenesTable1.html#h_alternativePathway) | 0.00026 | 0.00235 | < 0.005 (-) | 0.0000245 |
| 6 | h_monocytePathway | [Monocyte and its Surface Molecules](http://cgap.nci.nih.gov/Pathways/BioCarta/h_monocytePathway) | [9](../../../../C:%5CDocuments%20and%20Settings%5CAdministrator%5CDesktop%5CDystro2009_all%20-Project%5COutput%5CFshdinf%20vs%20fshdPathwayClassComparison_Bioc_filtered%5CGeneSetGenesTable1.html#h_monocytePathway) | 0.00026 | 0.00185 | 0.025 (-) | 0.0000754 |
| 7 | h_lymphocytePathway | [Adhesion Molecules on Lymphocyte](http://cgap.nci.nih.gov/Pathways/BioCarta/h_lymphocytePathway) | [7](../../../../C:%5CDocuments%20and%20Settings%5CAdministrator%5CDesktop%5CDystro2009_all%20-Project%5COutput%5CFshdinf%20vs%20fshdPathwayClassComparison_Bioc_filtered%5CGeneSetGenesTable1.html#h_lymphocytePathway) | 0.00033 | 0.00043 | < 0.005 (-) | 0.0000661 |
| 8 | h_iresPathway | [Internal Ribosome entry pathway](http://cgap.nci.nih.gov/Pathways/BioCarta/h_iresPathway) | [5](../../../../C:%5CDocuments%20and%20Settings%5CAdministrator%5CDesktop%5CDystro2009_all%20-Project%5COutput%5CFshdinf%20vs%20fshdPathwayClassComparison_Bioc_filtered%5CGeneSetGenesTable1.html#h_iresPathway) | 0.00047 | 0.01741 | 0.01 (-) | 0.000067 |
| 9 | h_eicosanoidPathway | [Eicosanoid Metabolism](http://cgap.nci.nih.gov/Pathways/BioCarta/h_eicosanoidPathway) | [18](../../../../C:%5CDocuments%20and%20Settings%5CAdministrator%5CDesktop%5CDystro2009_all%20-Project%5COutput%5CFshdinf%20vs%20fshdPathwayClassComparison_Bioc_filtered%5CGeneSetGenesTable1.html#h_eicosanoidPathway) | 0.00054 | 0.00144 | < 0.005 (-) | 0.0000162 |
| 10 | h_LairPathway | [Cells and Molecules involved in local acute inflammatory response](http://cgap.nci.nih.gov/Pathways/BioCarta/h_LairPathway) | [13](../../../../C:%5CDocuments%20and%20Settings%5CAdministrator%5CDesktop%5CDystro2009_all%20-Project%5COutput%5CFshdinf%20vs%20fshdPathwayClassComparison_Bioc_filtered%5CGeneSetGenesTable1.html#h_LairPathway) | 0.00082 | 0.01914 | 0.005 (-) | 0.0000056 |
| 11 | h_stressPathway | [TNF/Stress Related Signaling](http://cgap.nci.nih.gov/Pathways/BioCarta/h_stressPathway) | [20](../../../../C:%5CDocuments%20and%20Settings%5CAdministrator%5CDesktop%5CDystro2009_all%20-Project%5COutput%5CFshdinf%20vs%20fshdPathwayClassComparison_Bioc_filtered%5CGeneSetGenesTable1.html#h_stressPathway) | 0.00094 | 0.01419 | 0.005 (+) | 0.000076 |
| 12 | h_pgc1aPathway | [Regulation of PGC-1a](http://cgap.nci.nih.gov/Pathways/BioCarta/h_pgc1aPathway) | [11](../../../../C:%5CDocuments%20and%20Settings%5CAdministrator%5CDesktop%5CDystro2009_all%20-Project%5COutput%5CFshdinf%20vs%20fshdPathwayClassComparison_Bioc_filtered%5CGeneSetGenesTable1.html#h_pgc1aPathway) | 0.00122 | 0.01099 | 0.005 (+) | 0.0000694 |
| 13 | h_lympathway | [Adhesion and Diapedesis of Lymphocytes](http://cgap.nci.nih.gov/Pathways/BioCarta/h_lympathway) | [12](../../../../C:%5CDocuments%20and%20Settings%5CAdministrator%5CDesktop%5CDystro2009_all%20-Project%5COutput%5CFshdinf%20vs%20fshdPathwayClassComparison_Bioc_filtered%5CGeneSetGenesTable1.html#h_lympathway) | 0.00136 | 0.00618 | 0.005 (-) | 0.000081 |
| 14 | h_eif4Pathway | [Regulation of eIF4e and p70 S6 Kinase](http://cgap.nci.nih.gov/Pathways/BioCarta/h_eif4Pathway) | [17](../../../../C:%5CDocuments%20and%20Settings%5CAdministrator%5CDesktop%5CDystro2009_all%20-Project%5COutput%5CFshdinf%20vs%20fshdPathwayClassComparison_Bioc_filtered%5CGeneSetGenesTable1.html#h_eif4Pathway) | 0.00188 | 0.02423 | < 0.005 (-) | 0.0000055 |
| 15 | h_thelperPathway | [T Helper Cell Surface Molecules](http://cgap.nci.nih.gov/Pathways/BioCarta/h_thelperPathway) | [9](../../../../C:%5CDocuments%20and%20Settings%5CAdministrator%5CDesktop%5CDystro2009_all%20-Project%5COutput%5CFshdinf%20vs%20fshdPathwayClassComparison_Bioc_filtered%5CGeneSetGenesTable1.html#h_thelperPathway) | 0.00319 | 0.00179 | 0.055 (-) | 0.0000478 |
| 16 | h_tcytotoxicPathway | [T Cytotoxic Cell Surface Molecules](http://cgap.nci.nih.gov/Pathways/BioCarta/h_tcytotoxicPathway) | [9](../../../../C:%5CDocuments%20and%20Settings%5CAdministrator%5CDesktop%5CDystro2009_all%20-Project%5COutput%5CFshdinf%20vs%20fshdPathwayClassComparison_Bioc_filtered%5CGeneSetGenesTable1.html#h_tcytotoxicPathway) | 0.00332 | 0.00179 | 0.05 (-) | 0.0000902 |
| 17 | h_mTORPathway | [mTOR Signaling Pathway](http://cgap.nci.nih.gov/Pathways/BioCarta/h_mTORPathway) | [20](../../../../C:%5CDocuments%20and%20Settings%5CAdministrator%5CDesktop%5CDystro2009_all%20-Project%5COutput%5CFshdinf%20vs%20fshdPathwayClassComparison_Bioc_filtered%5CGeneSetGenesTable1.html#h_mTORPathway) | 0.00336 | 0.04125 | 0.005 (+) | 0.000003 |
| 18 | h_cellcyclePathway | [Cyclins and Cell Cycle Regulation](http://cgap.nci.nih.gov/Pathways/BioCarta/h_cellcyclePathway) | [21](../../../../C:%5CDocuments%20and%20Settings%5CAdministrator%5CDesktop%5CDystro2009_all%20-Project%5COutput%5CFshdinf%20vs%20fshdPathwayClassComparison_Bioc_filtered%5CGeneSetGenesTable1.html#h_cellcyclePathway) | 0.00357 | 0.02179 | 0.045 (-) | 0.0000789 |
| 19 | h_RacCycDPathway | [Influence of Ras and Rho proteins on G1 to S Transition](http://cgap.nci.nih.gov/Pathways/BioCarta/h_RacCycDPathway) | [22](../../../../C:%5CDocuments%20and%20Settings%5CAdministrator%5CDesktop%5CDystro2009_all%20-Project%5COutput%5CFshdinf%20vs%20fshdPathwayClassComparison_Bioc_filtered%5CGeneSetGenesTable1.html#h_RacCycDPathway) | 0.00405 | 0.03374 | 0.215 (+) | 0.0007088 |
| 20 | h_tollPathway | [Toll-Like Receptor Pathway](http://cgap.nci.nih.gov/Pathways/BioCarta/h_tollPathway) | [30](../../../../C:%5CDocuments%20and%20Settings%5CAdministrator%5CDesktop%5CDystro2009_all%20-Project%5COutput%5CFshdinf%20vs%20fshdPathwayClassComparison_Bioc_filtered%5CGeneSetGenesTable1.html#h_tollPathway) | 0.00429 | 0.06809 | 0.085 (+) | 0.0001518 |
| 21 | h_ctlPathway | [CTL mediated immune response against target cells](http://cgap.nci.nih.gov/Pathways/BioCarta/h_ctlPathway) | [11](../../../../C:%5CDocuments%20and%20Settings%5CAdministrator%5CDesktop%5CDystro2009_all%20-Project%5COutput%5CFshdinf%20vs%20fshdPathwayClassComparison_Bioc_filtered%5CGeneSetGenesTable1.html#h_ctlPathway) | 0.00484 | 0.00263 | 0.145 (-) | 0.0027349 |
| 22 | h_ceramidePathway | [Ceramide Signaling Pathway](http://cgap.nci.nih.gov/Pathways/BioCarta/h_ceramidePathway) | [19](../../../../C:%5CDocuments%20and%20Settings%5CAdministrator%5CDesktop%5CDystro2009_all%20-Project%5COutput%5CFshdinf%20vs%20fshdPathwayClassComparison_Bioc_filtered%5CGeneSetGenesTable1.html#h_ceramidePathway) | 0.00494 | 0.04717 | 0.02 (+) | 0.0001027 |
| 23 | h_granulocytesPathway | [Adhesion and Diapedesis of Granulocytes](http://cgap.nci.nih.gov/Pathways/BioCarta/h_granulocytesPathway) | [11](../../../../C:%5CDocuments%20and%20Settings%5CAdministrator%5CDesktop%5CDystro2009_all%20-Project%5COutput%5CFshdinf%20vs%20fshdPathwayClassComparison_Bioc_filtered%5CGeneSetGenesTable1.html#h_granulocytesPathway) | 0.0058 | 0.06515 | 0.005 (-) | 0.0003688 |
| 24 | h_il2rbPathway | [IL-2 Receptor Beta Chain in T cell Activation](http://cgap.nci.nih.gov/Pathways/BioCarta/h_il2rbPathway) | [28](../../../../C:%5CDocuments%20and%20Settings%5CAdministrator%5CDesktop%5CDystro2009_all%20-Project%5COutput%5CFshdinf%20vs%20fshdPathwayClassComparison_Bioc_filtered%5CGeneSetGenesTable1.html#h_il2rbPathway) | 0.00618 | 0.03246 | 0.185 (-) | 0.0000133 |
| 25 | h_dcPathway | [Dendritic cells in regulating TH1 and TH2 Development](http://cgap.nci.nih.gov/Pathways/BioCarta/h_dcPathway) | [11](../../../../C:%5CDocuments%20and%20Settings%5CAdministrator%5CDesktop%5CDystro2009_all%20-Project%5COutput%5CFshdinf%20vs%20fshdPathwayClassComparison_Bioc_filtered%5CGeneSetGenesTable1.html#h_dcPathway) | 0.0062 | 0.006 | 0.055 (-) | 0.0012504 |
| 26 | h_hdacPathway | [Control of skeletal myogenesis by HDAC & calcium/calmodulin-dependent kinase (CaMK)](http://cgap.nci.nih.gov/Pathways/BioCarta/h_hdacPathway) | [15](../../../../C:%5CDocuments%20and%20Settings%5CAdministrator%5CDesktop%5CDystro2009_all%20-Project%5COutput%5CFshdinf%20vs%20fshdPathwayClassComparison_Bioc_filtered%5CGeneSetGenesTable1.html#h_hdacPathway) | 0.00672 | 0.03978 | 0.06 (+) | 0.0000137 |
| 27 | h_malatexPathway | [Shuttle for transfer of acetyl groups from mitochondria to the cytosol](http://cgap.nci.nih.gov/Pathways/BioCarta/h_malatexPathway) | [8](../../../../C:%5CDocuments%20and%20Settings%5CAdministrator%5CDesktop%5CDystro2009_all%20-Project%5COutput%5CFshdinf%20vs%20fshdPathwayClassComparison_Bioc_filtered%5CGeneSetGenesTable1.html#h_malatexPathway) | 0.00806 | 0.01718 | 0.025 (+) | 0.0001679 |
| 28 | h_fMLPpathway | [fMLP induced chemokine gene expression in HMC-1 cells](http://cgap.nci.nih.gov/Pathways/BioCarta/h_fMLPpathway) | [21](../../../../C:%5CDocuments%20and%20Settings%5CAdministrator%5CDesktop%5CDystro2009_all%20-Project%5COutput%5CFshdinf%20vs%20fshdPathwayClassComparison_Bioc_filtered%5CGeneSetGenesTable1.html#h_fMLPpathway) | 0.00829 | 0.06782 | 0.005 (+) | 0.0013735 |
| 29 | h_keratinocytePathway | [Keratinocyte Differentiation](http://cgap.nci.nih.gov/Pathways/BioCarta/h_keratinocytePathway) | [32](../../../../C:%5CDocuments%20and%20Settings%5CAdministrator%5CDesktop%5CDystro2009_all%20-Project%5COutput%5CFshdinf%20vs%20fshdPathwayClassComparison_Bioc_filtered%5CGeneSetGenesTable1.html#h_keratinocytePathway) | 0.00862 | 0.07257 | 0.025 (+) | 0.0001975 |
| 30 | h_tidPathway | [Chaperones modulate interferon Signaling Pathway](http://cgap.nci.nih.gov/Pathways/BioCarta/h_tidPathway) | [16](../../../../C:%5CDocuments%20and%20Settings%5CAdministrator%5CDesktop%5CDystro2009_all%20-Project%5COutput%5CFshdinf%20vs%20fshdPathwayClassComparison_Bioc_filtered%5CGeneSetGenesTable1.html#h_tidPathway) | 0.0111 | 0.09455 | 0.03 (-) | 0.0000036 |
| 31 | h_etcPathway | [Electron Transport Reaction in Mitochondria](http://cgap.nci.nih.gov/Pathways/BioCarta/h_etcPathway) | [7](../../../../C:%5CDocuments%20and%20Settings%5CAdministrator%5CDesktop%5CDystro2009_all%20-Project%5COutput%5CFshdinf%20vs%20fshdPathwayClassComparison_Bioc_filtered%5CGeneSetGenesTable1.html#h_etcPathway) | 0.01282 | 0.03944 | < 0.005 (+) | 0.0000504 |
| 32 | h_lectinPathway | [Lectin Induced Complement Pathway](http://cgap.nci.nih.gov/Pathways/BioCarta/h_lectinPathway) | [8](../../../../C:%5CDocuments%20and%20Settings%5CAdministrator%5CDesktop%5CDystro2009_all%20-Project%5COutput%5CFshdinf%20vs%20fshdPathwayClassComparison_Bioc_filtered%5CGeneSetGenesTable1.html#h_lectinPathway) | 0.01366 | 0.07904 | 0.025 (-) | 0.0001074 |
| 33 | h_tcrPathway | [T Cell Receptor Signaling Pathway](http://cgap.nci.nih.gov/Pathways/BioCarta/h_tcrPathway) | [29](../../../../C:%5CDocuments%20and%20Settings%5CAdministrator%5CDesktop%5CDystro2009_all%20-Project%5COutput%5CFshdinf%20vs%20fshdPathwayClassComparison_Bioc_filtered%5CGeneSetGenesTable1.html#h_tcrPathway) | 0.01378 | 0.05146 | 0.21 (-) | 0.0003615 |
| 34 | h_ghrelinPathway | [Ghrelin: Regulation of Food Intake and Energy Homeostasis](http://cgap.nci.nih.gov/Pathways/BioCarta/h_ghrelinPathway) | [8](../../../../C:%5CDocuments%20and%20Settings%5CAdministrator%5CDesktop%5CDystro2009_all%20-Project%5COutput%5CFshdinf%20vs%20fshdPathwayClassComparison_Bioc_filtered%5CGeneSetGenesTable1.html#h_ghrelinPathway) | 0.0148 | 0.1085 | 0.01 (-) | 0.0000059 |
| 35 | h_tcraPathway | [Lck and Fyn tyrosine kinases in initiation of TCR Activation](http://cgap.nci.nih.gov/Pathways/BioCarta/h_tcraPathway) | [10](../../../../C:%5CDocuments%20and%20Settings%5CAdministrator%5CDesktop%5CDystro2009_all%20-Project%5COutput%5CFshdinf%20vs%20fshdPathwayClassComparison_Bioc_filtered%5CGeneSetGenesTable1.html#h_tcraPathway) | 0.01644 | 0.05407 | 0.08 (-) | 0.001193 |
| 36 | h_gsk3Pathway | [Inactivation of Gsk3 by AKT causes accumulation of b-catenin in Alveolar Macrophages](http://cgap.nci.nih.gov/Pathways/BioCarta/h_gsk3Pathway) | [22](../../../../C:%5CDocuments%20and%20Settings%5CAdministrator%5CDesktop%5CDystro2009_all%20-Project%5COutput%5CFshdinf%20vs%20fshdPathwayClassComparison_Bioc_filtered%5CGeneSetGenesTable1.html#h_gsk3Pathway) | 0.01715 | 0.27574 | 0.03 (-) | 0.0000152 |
| 37 | h_rnaPathway | [Double Stranded RNA Induced Gene Expression](http://cgap.nci.nih.gov/Pathways/BioCarta/h_rnaPathway) | [8](../../../../C:%5CDocuments%20and%20Settings%5CAdministrator%5CDesktop%5CDystro2009_all%20-Project%5COutput%5CFshdinf%20vs%20fshdPathwayClassComparison_Bioc_filtered%5CGeneSetGenesTable1.html#h_rnaPathway) | 0.01725 | 0.09728 | < 0.005 (+) | 0.0233829 |
| 38 | h_HivnefPathway | [HIV-I Nef: negative effector of Fas and TNF](http://cgap.nci.nih.gov/Pathways/BioCarta/h_HivnefPathway) | [45](../../../../C:%5CDocuments%20and%20Settings%5CAdministrator%5CDesktop%5CDystro2009_all%20-Project%5COutput%5CFshdinf%20vs%20fshdPathwayClassComparison_Bioc_filtered%5CGeneSetGenesTable1.html#h_HivnefPathway) | 0.01765 | 0.22939 | 0.25 (-) | 0.0000208 |
| 39 | h_rasPathway | [Ras Signaling Pathway](http://cgap.nci.nih.gov/Pathways/BioCarta/h_rasPathway) | [20](../../../../C:%5CDocuments%20and%20Settings%5CAdministrator%5CDesktop%5CDystro2009_all%20-Project%5COutput%5CFshdinf%20vs%20fshdPathwayClassComparison_Bioc_filtered%5CGeneSetGenesTable1.html#h_rasPathway) | 0.01776 | 0.00798 | 0.09 (+) | 0.000752 |
| 40 | h_blymphocytePathway | [B Lymphocyte Cell Surface Molecules](http://cgap.nci.nih.gov/Pathways/BioCarta/h_blymphocytePathway) | [9](../../../../C:%5CDocuments%20and%20Settings%5CAdministrator%5CDesktop%5CDystro2009_all%20-Project%5COutput%5CFshdinf%20vs%20fshdPathwayClassComparison_Bioc_filtered%5CGeneSetGenesTable1.html#h_blymphocytePathway) | 0.01829 | 0.09989 | 0.03 (-) | 0.0006266 |
| 41 | h_igf1mtorpathway | [Skeletal muscle hypertrophy is regulated via AKT/mTOR pathway](http://cgap.nci.nih.gov/Pathways/BioCarta/h_igf1mtorpathway) | [19](../../../../C:%5CDocuments%20and%20Settings%5CAdministrator%5CDesktop%5CDystro2009_all%20-Project%5COutput%5CFshdinf%20vs%20fshdPathwayClassComparison_Bioc_filtered%5CGeneSetGenesTable1.html#h_igf1mtorpathway) | 0.01879 | 0.03064 | < 0.005 (+) | 0.0000062 |
| 42 | h_fcer1Pathway | [Fc Epsilon Receptor I Signaling in Mast Cells](http://cgap.nci.nih.gov/Pathways/BioCarta/h_fcer1Pathway) | [23](../../../../C:%5CDocuments%20and%20Settings%5CAdministrator%5CDesktop%5CDystro2009_all%20-Project%5COutput%5CFshdinf%20vs%20fshdPathwayClassComparison_Bioc_filtered%5CGeneSetGenesTable1.html#h_fcer1Pathway) | 0.0188 | 0.1336 | 0.24 (+) | 0.0002554 |
| 43 | h_p53Pathway | [p53 Signaling Pathway](http://cgap.nci.nih.gov/Pathways/BioCarta/h_p53Pathway) | [14](../../../../C:%5CDocuments%20and%20Settings%5CAdministrator%5CDesktop%5CDystro2009_all%20-Project%5COutput%5CFshdinf%20vs%20fshdPathwayClassComparison_Bioc_filtered%5CGeneSetGenesTable1.html#h_p53Pathway) | 0.01903 | 0.15535 | 0.01 (-) | 0.0000015 |
| 44 | h_gcrPathway | [Corticosteroids and cardioprotection](http://cgap.nci.nih.gov/Pathways/BioCarta/h_gcrPathway) | [13](../../../../C:%5CDocuments%20and%20Settings%5CAdministrator%5CDesktop%5CDystro2009_all%20-Project%5COutput%5CFshdinf%20vs%20fshdPathwayClassComparison_Bioc_filtered%5CGeneSetGenesTable1.html#h_gcrPathway) | 0.01906 | 0.23037 | 0.015 (-) | 0.0000073 |
| 45 | h_mhcPathway | [Antigen Processing and Presentation](http://cgap.nci.nih.gov/Pathways/BioCarta/h_mhcPathway) | [9](../../../../C:%5CDocuments%20and%20Settings%5CAdministrator%5CDesktop%5CDystro2009_all%20-Project%5COutput%5CFshdinf%20vs%20fshdPathwayClassComparison_Bioc_filtered%5CGeneSetGenesTable1.html#h_mhcPathway) | 0.0194 | 0.00591 | 0.165 (-) | 0.0034947 |
| 46 | h_ifnaPathway | [IFN alpha signaling pathway](http://cgap.nci.nih.gov/Pathways/BioCarta/h_ifnaPathway) | [8](../../../../C:%5CDocuments%20and%20Settings%5CAdministrator%5CDesktop%5CDystro2009_all%20-Project%5COutput%5CFshdinf%20vs%20fshdPathwayClassComparison_Bioc_filtered%5CGeneSetGenesTable1.html#h_ifnaPathway) | 0.01951 | 0.27828 | 0.045 (-) | 0.0021248 |
| 47 | h_stathminPathway | [Stathmin and breast cancer resistance to antimicrotubule agents](http://cgap.nci.nih.gov/Pathways/BioCarta/h_stathminPathway) | [12](../../../../C:%5CDocuments%20and%20Settings%5CAdministrator%5CDesktop%5CDystro2009_all%20-Project%5COutput%5CFshdinf%20vs%20fshdPathwayClassComparison_Bioc_filtered%5CGeneSetGenesTable1.html#h_stathminPathway) | 0.0229 | 0.08417 | 0.16 (+) | 0.0000526 |
| 48 | h_caspasePathway | [Caspase Cascade in Apoptosis](http://cgap.nci.nih.gov/Pathways/BioCarta/h_caspasePathway) | [21](../../../../C:%5CDocuments%20and%20Settings%5CAdministrator%5CDesktop%5CDystro2009_all%20-Project%5COutput%5CFshdinf%20vs%20fshdPathwayClassComparison_Bioc_filtered%5CGeneSetGenesTable1.html#h_caspasePathway) | 0.02575 | 0.27856 | 0.14 (-) | 0.0001211 |
| 49 | h_CaCaMPathway | [Ca++/ Calmodulin-dependent Protein Kinase Activation](http://cgap.nci.nih.gov/Pathways/BioCarta/h_CaCaMPathway) | [5](../../../../C:%5CDocuments%20and%20Settings%5CAdministrator%5CDesktop%5CDystro2009_all%20-Project%5COutput%5CFshdinf%20vs%20fshdPathwayClassComparison_Bioc_filtered%5CGeneSetGenesTable1.html#h_CaCaMPathway) | 0.02898 | 0.28541 | 0.02 (+) | 0.0002683 |
| 50 | h_mitochondriaPathway | [Role of Mitochondria in Apoptotic Signaling](http://cgap.nci.nih.gov/Pathways/BioCarta/h_mitochondriaPathway) | [17](../../../../C:%5CDocuments%20and%20Settings%5CAdministrator%5CDesktop%5CDystro2009_all%20-Project%5COutput%5CFshdinf%20vs%20fshdPathwayClassComparison_Bioc_filtered%5CGeneSetGenesTable1.html#h_mitochondriaPathway) | 0.02994 | 0.17525 | 0.005 (+) | 0.0000203 |
| 51 | h_biopeptidesPathway | [Bioactive Peptide Induced Signaling Pathway](http://cgap.nci.nih.gov/Pathways/BioCarta/h_biopeptidesPathway) | [26](../../../../C:%5CDocuments%20and%20Settings%5CAdministrator%5CDesktop%5CDystro2009_all%20-Project%5COutput%5CFshdinf%20vs%20fshdPathwayClassComparison_Bioc_filtered%5CGeneSetGenesTable1.html#h_biopeptidesPathway) | 0.03235 | 0.47242 | 0.09 (+) | 0.000217 |
| 52 | h_d4gdiPathway | [D4-GDI Signaling Pathway](http://cgap.nci.nih.gov/Pathways/BioCarta/h_d4gdiPathway) | [13](../../../../C:%5CDocuments%20and%20Settings%5CAdministrator%5CDesktop%5CDystro2009_all%20-Project%5COutput%5CFshdinf%20vs%20fshdPathwayClassComparison_Bioc_filtered%5CGeneSetGenesTable1.html#h_d4gdiPathway) | 0.0334 | 0.15347 | 0.165 (-) | 0.000088 |
| 53 | h_ephA4Pathway | [Eph Kinases and ephrins support platelet aggregation](http://cgap.nci.nih.gov/Pathways/BioCarta/h_ephA4Pathway) | [9](../../../../C:%5CDocuments%20and%20Settings%5CAdministrator%5CDesktop%5CDystro2009_all%20-Project%5COutput%5CFshdinf%20vs%20fshdPathwayClassComparison_Bioc_filtered%5CGeneSetGenesTable1.html#h_ephA4Pathway) | 0.03345 | 0.10895 | 0.04 (-) | 0.0021961 |
| 54 | h_g1Pathway | [Cell Cycle: G1/S Check Point](http://cgap.nci.nih.gov/Pathways/BioCarta/h_g1Pathway) | [24](../../../../C:%5CDocuments%20and%20Settings%5CAdministrator%5CDesktop%5CDystro2009_all%20-Project%5COutput%5CFshdinf%20vs%20fshdPathwayClassComparison_Bioc_filtered%5CGeneSetGenesTable1.html#h_g1Pathway) | 0.03408 | 0.09542 | 0.36 (-) | 0.0005369 |
| 55 | h_plcPathway | [Phospholipase C Signaling Pathway](http://cgap.nci.nih.gov/Pathways/BioCarta/h_plcPathway) | [6](../../../../C:%5CDocuments%20and%20Settings%5CAdministrator%5CDesktop%5CDystro2009_all%20-Project%5COutput%5CFshdinf%20vs%20fshdPathwayClassComparison_Bioc_filtered%5CGeneSetGenesTable1.html#h_plcPathway) | 0.0352 | 0.17689 | 0.13 (+) | 0.0011075 |
| 56 | h_fibrinolysisPathway | [Fibrinolysis Pathway](http://cgap.nci.nih.gov/Pathways/BioCarta/h_fibrinolysisPathway) | [7](../../../../C:%5CDocuments%20and%20Settings%5CAdministrator%5CDesktop%5CDystro2009_all%20-Project%5COutput%5CFshdinf%20vs%20fshdPathwayClassComparison_Bioc_filtered%5CGeneSetGenesTable1.html#h_fibrinolysisPathway) | 0.03586 | 0.0775 | 0.06 (-) | 0.0004246 |
| 57 | h_sppaPathway | [Aspirin Blocks Signaling Pathway Involved in Platelet Activation](http://cgap.nci.nih.gov/Pathways/BioCarta/h_sppaPathway) | [17](../../../../C:%5CDocuments%20and%20Settings%5CAdministrator%5CDesktop%5CDystro2009_all%20-Project%5COutput%5CFshdinf%20vs%20fshdPathwayClassComparison_Bioc_filtered%5CGeneSetGenesTable1.html#h_sppaPathway) | 0.03627 | 0.3496 | 0.06 (-) | 0.0000804 |
| 58 | h_ifngPathway | [IFN gamma signaling pathway](http://cgap.nci.nih.gov/Pathways/BioCarta/h_ifngPathway) | [5](../../../../C:%5CDocuments%20and%20Settings%5CAdministrator%5CDesktop%5CDystro2009_all%20-Project%5COutput%5CFshdinf%20vs%20fshdPathwayClassComparison_Bioc_filtered%5CGeneSetGenesTable1.html#h_ifngPathway) | 0.03698 | 0.19978 | 0.025 (-) | 0.0004126 |
| 59 | h_il4Pathway | [IL 4 signaling pathway](http://cgap.nci.nih.gov/Pathways/BioCarta/h_il4Pathway) | [7](../../../../C:%5CDocuments%20and%20Settings%5CAdministrator%5CDesktop%5CDystro2009_all%20-Project%5COutput%5CFshdinf%20vs%20fshdPathwayClassComparison_Bioc_filtered%5CGeneSetGenesTable1.html#h_il4Pathway) | 0.03841 | 0.051 | 0.1 (-) | 0.0002387 |
| 60 | h_CSKPathway | [Activation of Csk by cAMP-dependent Protein Kinase Inhibits Signaling through the T Cell Receptor](http://cgap.nci.nih.gov/Pathways/BioCarta/h_CSKPathway) | [16](../../../../C:%5CDocuments%20and%20Settings%5CAdministrator%5CDesktop%5CDystro2009_all%20-Project%5COutput%5CFshdinf%20vs%20fshdPathwayClassComparison_Bioc_filtered%5CGeneSetGenesTable1.html#h_CSKPathway) | 0.04232 | 0.11672 | 0.09 (-) | 0.0011341 |
| 61 | h_bcrPathway | [BCR Signaling Pathway](http://cgap.nci.nih.gov/Pathways/BioCarta/h_bcrPathway) | [21](../../../../C:%5CDocuments%20and%20Settings%5CAdministrator%5CDesktop%5CDystro2009_all%20-Project%5COutput%5CFshdinf%20vs%20fshdPathwayClassComparison_Bioc_filtered%5CGeneSetGenesTable1.html#h_bcrPathway) | 0.04242 | 0.31835 | 0.26 (-) | 0.0033226 |
| 62 | h_glycolysisPathway | [Glycolysis Pathway](http://cgap.nci.nih.gov/Pathways/BioCarta/h_glycolysisPathway) | [6](../../../../C:%5CDocuments%20and%20Settings%5CAdministrator%5CDesktop%5CDystro2009_all%20-Project%5COutput%5CFshdinf%20vs%20fshdPathwayClassComparison_Bioc_filtered%5CGeneSetGenesTable1.html#h_glycolysisPathway) | 0.0425 | 0.13115 | 0.15 (+) | 0.0006349 |
| 63 | h_deathPathway | [Induction of apoptosis through DR3 and DR4/5 Death Receptors](http://cgap.nci.nih.gov/Pathways/BioCarta/h_deathPathway) | [26](../../../../C:%5CDocuments%20and%20Settings%5CAdministrator%5CDesktop%5CDystro2009_all%20-Project%5COutput%5CFshdinf%20vs%20fshdPathwayClassComparison_Bioc_filtered%5CGeneSetGenesTable1.html#h_deathPathway) | 0.04327 | 0.14719 | 0.3 (-) | 0.0001599 |
| 64 | h_eifPathway | [Eukaryotic protein translation](http://cgap.nci.nih.gov/Pathways/BioCarta/h_eifPathway) | [8](../../../../C:%5CDocuments%20and%20Settings%5CAdministrator%5CDesktop%5CDystro2009_all%20-Project%5COutput%5CFshdinf%20vs%20fshdPathwayClassComparison_Bioc_filtered%5CGeneSetGenesTable1.html#h_eifPathway) | 0.04342 | 0.00644 | 0.02 (+) | 0.0060586 |
| 65 | h_tnfr1Pathway | [TNFR1 Signaling Pathway](http://cgap.nci.nih.gov/Pathways/BioCarta/h_tnfr1Pathway) | [26](../../../../C:%5CDocuments%20and%20Settings%5CAdministrator%5CDesktop%5CDystro2009_all%20-Project%5COutput%5CFshdinf%20vs%20fshdPathwayClassComparison_Bioc_filtered%5CGeneSetGenesTable1.html#h_tnfr1Pathway) | 0.04546 | 0.36353 | 0.41 (-) | 0.0000683 |
| 66 | h_chemicalPathway | [Apoptotic Signaling in Response to DNA Damage](http://cgap.nci.nih.gov/Pathways/BioCarta/h_chemicalPathway) | [21](../../../../C:%5CDocuments%20and%20Settings%5CAdministrator%5CDesktop%5CDystro2009_all%20-Project%5COutput%5CFshdinf%20vs%20fshdPathwayClassComparison_Bioc_filtered%5CGeneSetGenesTable1.html#h_chemicalPathway) | 0.04688 | 0.1442 | 0.35 (-) | 0.0012456 |
| 67 | h_rac1Pathway | [Rac 1 cell motility signaling pathway](http://cgap.nci.nih.gov/Pathways/BioCarta/h_rac1Pathway) | [18](../../../../C:%5CDocuments%20and%20Settings%5CAdministrator%5CDesktop%5CDystro2009_all%20-Project%5COutput%5CFshdinf%20vs%20fshdPathwayClassComparison_Bioc_filtered%5CGeneSetGenesTable1.html#h_rac1Pathway) | 0.04691 | 0.08174 | 0.39 (-) | 0.0007488 |
| 68 | h_il7Pathway | [IL-7 Signal Transduction](http://cgap.nci.nih.gov/Pathways/BioCarta/h_il7Pathway) | [12](../../../../C:%5CDocuments%20and%20Settings%5CAdministrator%5CDesktop%5CDystro2009_all%20-Project%5COutput%5CFshdinf%20vs%20fshdPathwayClassComparison_Bioc_filtered%5CGeneSetGenesTable1.html#h_il7Pathway) | 0.05083 | 0.18271 | 0.08 (-) | 0.0002423 |
| 69 | h_th1th2Pathway | [Th1/Th2 Differentiation](http://cgap.nci.nih.gov/Pathways/BioCarta/h_th1th2Pathway) | [15](../../../../C:%5CDocuments%20and%20Settings%5CAdministrator%5CDesktop%5CDystro2009_all%20-Project%5COutput%5CFshdinf%20vs%20fshdPathwayClassComparison_Bioc_filtered%5CGeneSetGenesTable1.html#h_th1th2Pathway) | 0.05091 | 0.21466 | 0.09 (-) | 0.0001548 |
| 70 | h_plateletAppPathway | [Platelet Amyloid Precursor Protein Pathway](http://cgap.nci.nih.gov/Pathways/BioCarta/h_plateletAppPathway) | [7](../../../../C:%5CDocuments%20and%20Settings%5CAdministrator%5CDesktop%5CDystro2009_all%20-Project%5COutput%5CFshdinf%20vs%20fshdPathwayClassComparison_Bioc_filtered%5CGeneSetGenesTable1.html#h_plateletAppPathway) | 0.05403 | 0.00758 | 0.105 (-) | 0.0033863 |
| 71 | h_mapkPathway | [MAPKinase Signaling Pathway](http://cgap.nci.nih.gov/Pathways/BioCarta/h_mapkPathway) | [69](../../../../C:%5CDocuments%20and%20Settings%5CAdministrator%5CDesktop%5CDystro2009_all%20-Project%5COutput%5CFshdinf%20vs%20fshdPathwayClassComparison_Bioc_filtered%5CGeneSetGenesTable1.html#h_mapkPathway) | 0.05469 | 0.03863 | 0.005 (+) | 0.0001537 |
| 72 | h_atmPathway | [ATM Signaling Pathway](http://cgap.nci.nih.gov/Pathways/BioCarta/h_atmPathway) | [15](../../../../C:%5CDocuments%20and%20Settings%5CAdministrator%5CDesktop%5CDystro2009_all%20-Project%5COutput%5CFshdinf%20vs%20fshdPathwayClassComparison_Bioc_filtered%5CGeneSetGenesTable1.html#h_atmPathway) | 0.05675 | 0.10867 | 0.115 (-) | 0.0055718 |
| 73 | h_g2Pathway | [Cell Cycle: G2/M Checkpoint](http://cgap.nci.nih.gov/Pathways/BioCarta/h_g2Pathway) | [18](../../../../C:%5CDocuments%20and%20Settings%5CAdministrator%5CDesktop%5CDystro2009_all%20-Project%5COutput%5CFshdinf%20vs%20fshdPathwayClassComparison_Bioc_filtered%5CGeneSetGenesTable1.html#h_g2Pathway) | 0.06363 | 0.29771 | 0.14 (-) | 0.0006538 |
| 74 | h_nktPathway | [Selective expression of chemokine receptors during T-cell polarization](http://cgap.nci.nih.gov/Pathways/BioCarta/h_nktPathway) | [16](../../../../C:%5CDocuments%20and%20Settings%5CAdministrator%5CDesktop%5CDystro2009_all%20-Project%5COutput%5CFshdinf%20vs%20fshdPathwayClassComparison_Bioc_filtered%5CGeneSetGenesTable1.html#h_nktPathway) | 0.06375 | 0.11417 | 0.1 (-) | 0.0001542 |
| 75 | h_longevityPathway | [The IGF-1 Receptor and Longevity](http://cgap.nci.nih.gov/Pathways/BioCarta/h_longevityPathway) | [10](../../../../C:%5CDocuments%20and%20Settings%5CAdministrator%5CDesktop%5CDystro2009_all%20-Project%5COutput%5CFshdinf%20vs%20fshdPathwayClassComparison_Bioc_filtered%5CGeneSetGenesTable1.html#h_longevityPathway) | 0.06556 | 0.05921 | 0.215 (-) | 0.0000252 |
| 76 | h_bbcellPathway | [Bystander B Cell Activation](http://cgap.nci.nih.gov/Pathways/BioCarta/h_bbcellPathway) | [7](../../../../C:%5CDocuments%20and%20Settings%5CAdministrator%5CDesktop%5CDystro2009_all%20-Project%5COutput%5CFshdinf%20vs%20fshdPathwayClassComparison_Bioc_filtered%5CGeneSetGenesTable1.html#h_bbcellPathway) | 0.06607 | 0.07225 | 0.125 (-) | 0.0007485 |
| 77 | h_bArrestin-srcPathway | [Roles of ¿-arrestin-dependent Recruitment of Src Kinases in GPCR Signaling](http://cgap.nci.nih.gov/Pathways/BioCarta/h_bArrestin-srcPathway) | [16](../../../../C:%5CDocuments%20and%20Settings%5CAdministrator%5CDesktop%5CDystro2009_all%20-Project%5COutput%5CFshdinf%20vs%20fshdPathwayClassComparison_Bioc_filtered%5CGeneSetGenesTable1.html#h_bArrestin-srcPathway) | 0.06798 | 0.35784 | 0.29 (+) | 0.0007222 |
| 78 | h_nfkbPathway | [NF-kB Signaling Pathway](http://cgap.nci.nih.gov/Pathways/BioCarta/h_nfkbPathway) | [19](../../../../C:%5CDocuments%20and%20Settings%5CAdministrator%5CDesktop%5CDystro2009_all%20-Project%5COutput%5CFshdinf%20vs%20fshdPathwayClassComparison_Bioc_filtered%5CGeneSetGenesTable1.html#h_nfkbPathway) | 0.06871 | 0.43143 | 0.08 (-) | 0.0007018 |
| 79 | h_prionPathway | [Prion Pathway](http://cgap.nci.nih.gov/Pathways/BioCarta/h_prionPathway) | [10](../../../../C:%5CDocuments%20and%20Settings%5CAdministrator%5CDesktop%5CDystro2009_all%20-Project%5COutput%5CFshdinf%20vs%20fshdPathwayClassComparison_Bioc_filtered%5CGeneSetGenesTable1.html#h_prionPathway) | 0.07205 | 0.24763 | 0.07 (-) | 0.000646 |
| 80 | h_tall1Pathway | [TACI and BCMA stimulation of B cell immune responses.](http://cgap.nci.nih.gov/Pathways/BioCarta/h_tall1Pathway) | [11](../../../../C:%5CDocuments%20and%20Settings%5CAdministrator%5CDesktop%5CDystro2009_all%20-Project%5COutput%5CFshdinf%20vs%20fshdPathwayClassComparison_Bioc_filtered%5CGeneSetGenesTable1.html#h_tall1Pathway) | 0.07363 | 0.05006 | 0.215 (-) | 0.0010671 |
| 81 | h_plk3Pathway | [Regulation of cell cycle progression by Plk3](http://cgap.nci.nih.gov/Pathways/BioCarta/h_plk3Pathway) | [8](../../../../C:%5CDocuments%20and%20Settings%5CAdministrator%5CDesktop%5CDystro2009_all%20-Project%5COutput%5CFshdinf%20vs%20fshdPathwayClassComparison_Bioc_filtered%5CGeneSetGenesTable1.html#h_plk3Pathway) | 0.08307 | 0.19075 | 0.135 (-) | 0.0009677 |
| 82 | h_il22bppathway | [IL22 Soluble Receptor Signaling Pathway](http://cgap.nci.nih.gov/Pathways/BioCarta/h_il22bppathway) | [6](../../../../C:%5CDocuments%20and%20Settings%5CAdministrator%5CDesktop%5CDystro2009_all%20-Project%5COutput%5CFshdinf%20vs%20fshdPathwayClassComparison_Bioc_filtered%5CGeneSetGenesTable1.html#h_il22bppathway) | 0.08326 | 0.12134 | 0.15 (-) | 0.0050573 |
| 83 | h_nthiPathway | [NFkB activation by Nontypeable Hemophilus influenzae](http://cgap.nci.nih.gov/Pathways/BioCarta/h_nthiPathway) | [21](../../../../C:%5CDocuments%20and%20Settings%5CAdministrator%5CDesktop%5CDystro2009_all%20-Project%5COutput%5CFshdinf%20vs%20fshdPathwayClassComparison_Bioc_filtered%5CGeneSetGenesTable1.html#h_nthiPathway) | 0.08436 | 0.11116 | 0.185 (+) | 0.0001177 |
| 84 | h_tsp1Pathway | [TSP-1 Induced Apoptosis in Microvascular Endothelial Cell](http://cgap.nci.nih.gov/Pathways/BioCarta/h_tsp1Pathway) | [5](../../../../C:%5CDocuments%20and%20Settings%5CAdministrator%5CDesktop%5CDystro2009_all%20-Project%5COutput%5CFshdinf%20vs%20fshdPathwayClassComparison_Bioc_filtered%5CGeneSetGenesTable1.html#h_tsp1Pathway) | 0.08622 | 0.05175 | 0.09 (-) | 0.0045168 |
| 85 | h_arapPathway | [ADP-Ribosylation Factor](http://cgap.nci.nih.gov/Pathways/BioCarta/h_arapPathway) | [9](../../../../C:%5CDocuments%20and%20Settings%5CAdministrator%5CDesktop%5CDystro2009_all%20-Project%5COutput%5CFshdinf%20vs%20fshdPathwayClassComparison_Bioc_filtered%5CGeneSetGenesTable1.html#h_arapPathway) | 0.08885 | 0.06627 | 0.28 (+) | 0.002368 |
| 86 | h_EfpPathway | [Estrogen-responsive protein Efp controls cell cycle and breast tumors growth](http://cgap.nci.nih.gov/Pathways/BioCarta/h_EfpPathway) | [7](../../../../C:%5CDocuments%20and%20Settings%5CAdministrator%5CDesktop%5CDystro2009_all%20-Project%5COutput%5CFshdinf%20vs%20fshdPathwayClassComparison_Bioc_filtered%5CGeneSetGenesTable1.html#h_EfpPathway) | 0.08959 | 0.13413 | 0.125 (-) | 0.0007114 |
| 87 | h_badPathway | [Regulation of BAD phosphorylation](http://cgap.nci.nih.gov/Pathways/BioCarta/h_badPathway) | [17](../../../../C:%5CDocuments%20and%20Settings%5CAdministrator%5CDesktop%5CDystro2009_all%20-Project%5COutput%5CFshdinf%20vs%20fshdPathwayClassComparison_Bioc_filtered%5CGeneSetGenesTable1.html#h_badPathway) | 0.09161 | 0.03207 | 0.195 (-) | 0.0003991 |
| 88 | h_RELAPathway | [Acetylation and Deacetylation of RelA in The Nucleus](http://cgap.nci.nih.gov/Pathways/BioCarta/h_RELAPathway) | [12](../../../../C:%5CDocuments%20and%20Settings%5CAdministrator%5CDesktop%5CDystro2009_all%20-Project%5COutput%5CFshdinf%20vs%20fshdPathwayClassComparison_Bioc_filtered%5CGeneSetGenesTable1.html#h_RELAPathway) | 0.0967 | 0.27198 | 0.225 (-) | 0.0003392 |
| 89 | h_vitCBPathway | [Vitamin C in the Brain](http://cgap.nci.nih.gov/Pathways/BioCarta/h_vitCBPathway) | [6](../../../../C:%5CDocuments%20and%20Settings%5CAdministrator%5CDesktop%5CDystro2009_all%20-Project%5COutput%5CFshdinf%20vs%20fshdPathwayClassComparison_Bioc_filtered%5CGeneSetGenesTable1.html#h_vitCBPathway) | 0.09887 | 0.02062 | 0.125 (-) | 0.0000031 |
| 90 | h_rbPathway | [RB Tumor Suppressor/Checkpoint Signaling in response to DNA damage](http://cgap.nci.nih.gov/Pathways/BioCarta/h_rbPathway) | [9](../../../../C:%5CDocuments%20and%20Settings%5CAdministrator%5CDesktop%5CDystro2009_all%20-Project%5COutput%5CFshdinf%20vs%20fshdPathwayClassComparison_Bioc_filtered%5CGeneSetGenesTable1.html#h_rbPathway) | 0.09927 | 0.33915 | 0.185 (-) | 0.0005677 |
| 91 | h_raccPathway | [Ion Channels and Their Functional Role in Vascular Endothelium](http://cgap.nci.nih.gov/Pathways/BioCarta/h_raccPathway) | [7](../../../../C:%5CDocuments%20and%20Settings%5CAdministrator%5CDesktop%5CDystro2009_all%20-Project%5COutput%5CFshdinf%20vs%20fshdPathwayClassComparison_Bioc_filtered%5CGeneSetGenesTable1.html#h_raccPathway) | 0.10184 | 0.40776 | 0.38 (-) | 0.0009771 |
| 92 | h_tubbyPathway | [G-Protein Signaling Through Tubby Proteins](http://cgap.nci.nih.gov/Pathways/BioCarta/h_tubbyPathway) | [5](../../../../C:%5CDocuments%20and%20Settings%5CAdministrator%5CDesktop%5CDystro2009_all%20-Project%5COutput%5CFshdinf%20vs%20fshdPathwayClassComparison_Bioc_filtered%5CGeneSetGenesTable1.html#h_tubbyPathway) | 0.10237 | 0.59387 | 0.22 (-) | 0.0013622 |
| 93 | h_asbcellPathway | [Antigen Dependent B Cell Activation](http://cgap.nci.nih.gov/Pathways/BioCarta/h_asbcellPathway) | [8](../../../../C:%5CDocuments%20and%20Settings%5CAdministrator%5CDesktop%5CDystro2009_all%20-Project%5COutput%5CFshdinf%20vs%20fshdPathwayClassComparison_Bioc_filtered%5CGeneSetGenesTable1.html#h_asbcellPathway) | 0.10269 | 0.13233 | 0.205 (-) | 0.0006037 |
| 94 | h_edg1Pathway | [Phospholipids as signalling intermediaries](http://cgap.nci.nih.gov/Pathways/BioCarta/h_edg1Pathway) | [21](../../../../C:%5CDocuments%20and%20Settings%5CAdministrator%5CDesktop%5CDystro2009_all%20-Project%5COutput%5CFshdinf%20vs%20fshdPathwayClassComparison_Bioc_filtered%5CGeneSetGenesTable1.html#h_edg1Pathway) | 0.10278 | 0.12728 | 0.2 (-) | 0.0000194 |
| 95 | h_gleevecpathway | [Inhibition of Cellular Proliferation by Gleevec](http://cgap.nci.nih.gov/Pathways/BioCarta/h_gleevecpathway) | [16](../../../../C:%5CDocuments%20and%20Settings%5CAdministrator%5CDesktop%5CDystro2009_all%20-Project%5COutput%5CFshdinf%20vs%20fshdPathwayClassComparison_Bioc_filtered%5CGeneSetGenesTable1.html#h_gleevecpathway) | 0.11011 | 0.10547 | 0.13 (+) | 0.0014129 |
| 96 | h_il17Pathway | [IL 17 Signaling Pathway](http://cgap.nci.nih.gov/Pathways/BioCarta/h_il17Pathway) | [11](../../../../C:%5CDocuments%20and%20Settings%5CAdministrator%5CDesktop%5CDystro2009_all%20-Project%5COutput%5CFshdinf%20vs%20fshdPathwayClassComparison_Bioc_filtered%5CGeneSetGenesTable1.html#h_il17Pathway) | 0.11327 | 0.02387 | 0.13 (-) | 0.0061514 |
| 97 | h_spryPathway | [Sprouty regulation of tyrosine kinase signals](http://cgap.nci.nih.gov/Pathways/BioCarta/h_spryPathway) | [15](../../../../C:%5CDocuments%20and%20Settings%5CAdministrator%5CDesktop%5CDystro2009_all%20-Project%5COutput%5CFshdinf%20vs%20fshdPathwayClassComparison_Bioc_filtered%5CGeneSetGenesTable1.html#h_spryPathway) | 0.12064 | 0.18442 | 0.185 (+) | 0.0000455 |
| 98 | h_nfatPathway | [NFAT and Hypertrophy of the heart (Transcription in the broken heart)](http://cgap.nci.nih.gov/Pathways/BioCarta/h_nfatPathway) | [28](../../../../C:%5CDocuments%20and%20Settings%5CAdministrator%5CDesktop%5CDystro2009_all%20-Project%5COutput%5CFshdinf%20vs%20fshdPathwayClassComparison_Bioc_filtered%5CGeneSetGenesTable1.html#h_nfatPathway) | 0.12532 | 0.0801 | 0.055 (+) | 0.0023028 |
| 99 | h_ctla4Pathway | [The Co-Stimulatory Signal During T-cell Activation](http://cgap.nci.nih.gov/Pathways/BioCarta/h_ctla4Pathway) | [15](../../../../C:%5CDocuments%20and%20Settings%5CAdministrator%5CDesktop%5CDystro2009_all%20-Project%5COutput%5CFshdinf%20vs%20fshdPathwayClassComparison_Bioc_filtered%5CGeneSetGenesTable1.html#h_ctla4Pathway) | 0.12641 | 0.03698 | 0.19 (-) | 0.0021905 |
| 100 | h_argininecPathway | [Catabolic Pathways for Arginine , Histidine, Glutamate, Glutamine, and Proline](http://cgap.nci.nih.gov/Pathways/BioCarta/h_argininecPathway) | [5](../../../../C:%5CDocuments%20and%20Settings%5CAdministrator%5CDesktop%5CDystro2009_all%20-Project%5COutput%5CFshdinf%20vs%20fshdPathwayClassComparison_Bioc_filtered%5CGeneSetGenesTable1.html#h_argininecPathway) | 0.12693 | 0.22501 | 0.05 (+) | 0.0022714 |
| 101 | h_eif2Pathway | [Regulation of eIF2](http://cgap.nci.nih.gov/Pathways/BioCarta/h_eif2Pathway) | [11](../../../../C:%5CDocuments%20and%20Settings%5CAdministrator%5CDesktop%5CDystro2009_all%20-Project%5COutput%5CFshdinf%20vs%20fshdPathwayClassComparison_Bioc_filtered%5CGeneSetGenesTable2.html#h_eif2Pathway) | 0.14073 | 0.47253 | 0.01 (+) | 0.0002542 |
| 102 | h_no1Pathway | [Actions of Nitric Oxide in the Heart](http://cgap.nci.nih.gov/Pathways/BioCarta/h_no1Pathway) | [13](../../../../C:%5CDocuments%20and%20Settings%5CAdministrator%5CDesktop%5CDystro2009_all%20-Project%5COutput%5CFshdinf%20vs%20fshdPathwayClassComparison_Bioc_filtered%5CGeneSetGenesTable2.html#h_no1Pathway) | 0.14632 | 0.06789 | 0.265 (-) | 0.0086385 |
| 103 | h_barr-mapkPathway | [Role of ¿-arrestins in the activation and targeting of MAP kinases](http://cgap.nci.nih.gov/Pathways/BioCarta/h_barr-mapkPathway) | [12](../../../../C:%5CDocuments%20and%20Settings%5CAdministrator%5CDesktop%5CDystro2009_all%20-Project%5COutput%5CFshdinf%20vs%20fshdPathwayClassComparison_Bioc_filtered%5CGeneSetGenesTable2.html#h_barr-mapkPathway) | 0.14773 | 0.62697 | 0.195 (+) | 0.0020233 |
| 104 | h_cblPathway | [CBL mediated ligand-induced downregulation of EGF receptors](http://cgap.nci.nih.gov/Pathways/BioCarta/h_cblPathway) | [10](../../../../C:%5CDocuments%20and%20Settings%5CAdministrator%5CDesktop%5CDystro2009_all%20-Project%5COutput%5CFshdinf%20vs%20fshdPathwayClassComparison_Bioc_filtered%5CGeneSetGenesTable2.html#h_cblPathway) | 0.14953 | 0.24295 | 0.24 (-) | 0.0004552 |
| 105 | h_tob1Pathway | [Role of Tob in T-cell activation](http://cgap.nci.nih.gov/Pathways/BioCarta/h_tob1Pathway) | [9](../../../../C:%5CDocuments%20and%20Settings%5CAdministrator%5CDesktop%5CDystro2009_all%20-Project%5COutput%5CFshdinf%20vs%20fshdPathwayClassComparison_Bioc_filtered%5CGeneSetGenesTable2.html#h_tob1Pathway) | 0.14984 | 0.02061 | 0.375 (-) | 0.0009477 |
| 106 | h_ptenPathway | [PTEN dependent cell cycle arrest and apoptosis](http://cgap.nci.nih.gov/Pathways/BioCarta/h_ptenPathway) | [18](../../../../C:%5CDocuments%20and%20Settings%5CAdministrator%5CDesktop%5CDystro2009_all%20-Project%5COutput%5CFshdinf%20vs%20fshdPathwayClassComparison_Bioc_filtered%5CGeneSetGenesTable2.html#h_ptenPathway) | 0.15286 | 0.47995 | 0.205 (+) | 0.0019556 |
| 107 | h_ppargPathway | [Role of PPAR-gamma Coactivators in Obesity and Thermogenesis](http://cgap.nci.nih.gov/Pathways/BioCarta/h_ppargPathway) | [7](../../../../C:%5CDocuments%20and%20Settings%5CAdministrator%5CDesktop%5CDystro2009_all%20-Project%5COutput%5CFshdinf%20vs%20fshdPathwayClassComparison_Bioc_filtered%5CGeneSetGenesTable2.html#h_ppargPathway) | 0.15376 | 0.21479 | 0.005 (+) | 0.0011308 |
| 108 | h_agpcrPathway | [Attenuation of GPCR Signaling](http://cgap.nci.nih.gov/Pathways/BioCarta/h_agpcrPathway) | [7](../../../../C:%5CDocuments%20and%20Settings%5CAdministrator%5CDesktop%5CDystro2009_all%20-Project%5COutput%5CFshdinf%20vs%20fshdPathwayClassComparison_Bioc_filtered%5CGeneSetGenesTable2.html#h_agpcrPathway) | 0.15391 | 0.50121 | 0.055 (-) | 0.0010258 |
| 109 | h_MITRPathway | [Signal Dependent Regulation of Myogenesis by Corepressor MITR](http://cgap.nci.nih.gov/Pathways/BioCarta/h_MITRPathway) | [5](../../../../C:%5CDocuments%20and%20Settings%5CAdministrator%5CDesktop%5CDystro2009_all%20-Project%5COutput%5CFshdinf%20vs%20fshdPathwayClassComparison_Bioc_filtered%5CGeneSetGenesTable2.html#h_MITRPathway) | 0.16174 | 0.23337 | 0.305 (-) | 0.0021276 |
| 110 | h_p38mapkPathway | [p38 MAPK Signaling Pathway](http://cgap.nci.nih.gov/Pathways/BioCarta/h_p38mapkPathway) | [28](../../../../C:%5CDocuments%20and%20Settings%5CAdministrator%5CDesktop%5CDystro2009_all%20-Project%5COutput%5CFshdinf%20vs%20fshdPathwayClassComparison_Bioc_filtered%5CGeneSetGenesTable2.html#h_p38mapkPathway) | 0.1623 | 0.20605 | 0.19 (+) | 0.0000331 |
| 111 | h_il1rPathway | [Signal transduction through IL1R](http://cgap.nci.nih.gov/Pathways/BioCarta/h_il1rPathway) | [22](../../../../C:%5CDocuments%20and%20Settings%5CAdministrator%5CDesktop%5CDystro2009_all%20-Project%5COutput%5CFshdinf%20vs%20fshdPathwayClassComparison_Bioc_filtered%5CGeneSetGenesTable2.html#h_il1rPathway) | 0.16464 | 0.33423 | 0.17 (+) | 0.0000505 |
| 112 | h_igf1rPathway | [Multiple antiapoptotic pathways from IGF-1R signaling lead to BAD phosphorylation](http://cgap.nci.nih.gov/Pathways/BioCarta/h_igf1rPathway) | [17](../../../../C:%5CDocuments%20and%20Settings%5CAdministrator%5CDesktop%5CDystro2009_all%20-Project%5COutput%5CFshdinf%20vs%20fshdPathwayClassComparison_Bioc_filtered%5CGeneSetGenesTable2.html#h_igf1rPathway) | 0.16647 | 0.2242 | 0.395 (-) | 0.0000348 |
| 113 | h_igf1Pathway | [IGF-1 Signaling Pathway](http://cgap.nci.nih.gov/Pathways/BioCarta/h_igf1Pathway) | [15](../../../../C:%5CDocuments%20and%20Settings%5CAdministrator%5CDesktop%5CDystro2009_all%20-Project%5COutput%5CFshdinf%20vs%20fshdPathwayClassComparison_Bioc_filtered%5CGeneSetGenesTable2.html#h_igf1Pathway) | 0.17319 | 0.03362 | 0.06 (+) | 0.0008122 |
| 114 | h_nkcellsPathway | [Ras-Independent pathway in NK cell-mediated cytotoxicity](http://cgap.nci.nih.gov/Pathways/BioCarta/h_nkcellsPathway) | [18](../../../../C:%5CDocuments%20and%20Settings%5CAdministrator%5CDesktop%5CDystro2009_all%20-Project%5COutput%5CFshdinf%20vs%20fshdPathwayClassComparison_Bioc_filtered%5CGeneSetGenesTable2.html#h_nkcellsPathway) | 0.17602 | 0.25091 | 0.175 (-) | 0.0005888 |
| 115 | h_ecmPathway | [Erk and PI-3 Kinase Are Necessary for Collagen Binding in Corneal Epithelia](http://cgap.nci.nih.gov/Pathways/BioCarta/h_ecmPathway) | [20](../../../../C:%5CDocuments%20and%20Settings%5CAdministrator%5CDesktop%5CDystro2009_all%20-Project%5COutput%5CFshdinf%20vs%20fshdPathwayClassComparison_Bioc_filtered%5CGeneSetGenesTable2.html#h_ecmPathway) | 0.17664 | 0.65525 | 0.255 (-) | 0.0003244 |
| 116 | h_bArrestinPathway | [¿-arrestins in GPCR Desensitization](http://cgap.nci.nih.gov/Pathways/BioCarta/h_bArrestinPathway) | [9](../../../../C:%5CDocuments%20and%20Settings%5CAdministrator%5CDesktop%5CDystro2009_all%20-Project%5COutput%5CFshdinf%20vs%20fshdPathwayClassComparison_Bioc_filtered%5CGeneSetGenesTable2.html#h_bArrestinPathway) | 0.18166 | 0.6533 | 0.32 (+) | 0.0011472 |
| 117 | h_cdMacPathway | [Cadmium induces DNA synthesis and proliferation in macrophages](http://cgap.nci.nih.gov/Pathways/BioCarta/h_cdMacPathway) | [12](../../../../C:%5CDocuments%20and%20Settings%5CAdministrator%5CDesktop%5CDystro2009_all%20-Project%5COutput%5CFshdinf%20vs%20fshdPathwayClassComparison_Bioc_filtered%5CGeneSetGenesTable2.html#h_cdMacPathway) | 0.18407 | 0.35347 | 0.05 (+) | 0.0014896 |
| 118 | h_akap13Pathway | [Rho-Selective Guanine Exchange Factor AKAP13 Mediates Stress Fiber Formation](http://cgap.nci.nih.gov/Pathways/BioCarta/h_akap13Pathway) | [7](../../../../C:%5CDocuments%20and%20Settings%5CAdministrator%5CDesktop%5CDystro2009_all%20-Project%5COutput%5CFshdinf%20vs%20fshdPathwayClassComparison_Bioc_filtered%5CGeneSetGenesTable2.html#h_akap13Pathway) | 0.18592 | 0.22639 | 0.315 (+) | 0.0009772 |
| 119 | h_il2Pathway | [IL 2 signaling pathway](http://cgap.nci.nih.gov/Pathways/BioCarta/h_il2Pathway) | [16](../../../../C:%5CDocuments%20and%20Settings%5CAdministrator%5CDesktop%5CDystro2009_all%20-Project%5COutput%5CFshdinf%20vs%20fshdPathwayClassComparison_Bioc_filtered%5CGeneSetGenesTable2.html#h_il2Pathway) | 0.18858 | 0.35784 | 0.35 (+) | 0.0000832 |
| 120 | h_IL12Pathway | [IL12 and Stat4 Dependent Signaling Pathway in Th1 Development](http://cgap.nci.nih.gov/Pathways/BioCarta/h_IL12Pathway) | [15](../../../../C:%5CDocuments%20and%20Settings%5CAdministrator%5CDesktop%5CDystro2009_all%20-Project%5COutput%5CFshdinf%20vs%20fshdPathwayClassComparison_Bioc_filtered%5CGeneSetGenesTable2.html#h_IL12Pathway) | 0.18929 | 0.36367 | 0.495 (+) | 0.0041591 |
| 121 | h_mta3Pathway | [Downregulated of MTA-3 in ER-negative Breast Tumors](http://cgap.nci.nih.gov/Pathways/BioCarta/h_mta3Pathway) | [13](../../../../C:%5CDocuments%20and%20Settings%5CAdministrator%5CDesktop%5CDystro2009_all%20-Project%5COutput%5CFshdinf%20vs%20fshdPathwayClassComparison_Bioc_filtered%5CGeneSetGenesTable2.html#h_mta3Pathway) | 0.19076 | 0.2183 | 0.14 (+) | 0.0007934 |
| 122 | h_pcafpathway | [The information-processing pathway at the IFN-beta enhancer](http://cgap.nci.nih.gov/Pathways/BioCarta/h_pcafpathway) | [6](../../../../C:%5CDocuments%20and%20Settings%5CAdministrator%5CDesktop%5CDystro2009_all%20-Project%5COutput%5CFshdinf%20vs%20fshdPathwayClassComparison_Bioc_filtered%5CGeneSetGenesTable2.html#h_pcafpathway) | 0.19145 | 0.19528 | 0.265 (-) | 0.0017272 |
| 123 | h_btg2Pathway | [BTG family proteins and cell cycle regulation](http://cgap.nci.nih.gov/Pathways/BioCarta/h_btg2Pathway) | [8](../../../../C:%5CDocuments%20and%20Settings%5CAdministrator%5CDesktop%5CDystro2009_all%20-Project%5COutput%5CFshdinf%20vs%20fshdPathwayClassComparison_Bioc_filtered%5CGeneSetGenesTable2.html#h_btg2Pathway) | 0.19212 | 0.50571 | 0.245 (+) | 0.0023117 |
| 124 | h_hcmvPathway | [Human Cytomegalovirus and Map Kinase Pathways](http://cgap.nci.nih.gov/Pathways/BioCarta/h_hcmvPathway) | [12](../../../../C:%5CDocuments%20and%20Settings%5CAdministrator%5CDesktop%5CDystro2009_all%20-Project%5COutput%5CFshdinf%20vs%20fshdPathwayClassComparison_Bioc_filtered%5CGeneSetGenesTable2.html#h_hcmvPathway) | 0.19614 | 0.6163 | 0.07 (+) | 0.0062192 |
| 125 | h_hesPathway | [Segmentation Clock](http://cgap.nci.nih.gov/Pathways/BioCarta/h_hesPathway) | [9](../../../../C:%5CDocuments%20and%20Settings%5CAdministrator%5CDesktop%5CDystro2009_all%20-Project%5COutput%5CFshdinf%20vs%20fshdPathwayClassComparison_Bioc_filtered%5CGeneSetGenesTable2.html#h_hesPathway) | 0.19807 | 0.20133 | 0.51 (+) | 0.0026145 |
| 126 | h_il10Pathway | [IL-10 Anti-inflammatory Signaling Pathway](http://cgap.nci.nih.gov/Pathways/BioCarta/h_il10Pathway) | [8](../../../../C:%5CDocuments%20and%20Settings%5CAdministrator%5CDesktop%5CDystro2009_all%20-Project%5COutput%5CFshdinf%20vs%20fshdPathwayClassComparison_Bioc_filtered%5CGeneSetGenesTable2.html#h_il10Pathway) | 0.19823 | 0.57792 | 0.17 (-) | 0.0008767 |
| 127 | h_insulinPathway | [Insulin Signaling Pathway](http://cgap.nci.nih.gov/Pathways/BioCarta/h_insulinPathway) | [14](../../../../C:%5CDocuments%20and%20Settings%5CAdministrator%5CDesktop%5CDystro2009_all%20-Project%5COutput%5CFshdinf%20vs%20fshdPathwayClassComparison_Bioc_filtered%5CGeneSetGenesTable2.html#h_insulinPathway) | 0.20037 | 0.05663 | 0.05 (+) | 0.0007504 |
| 128 | h_ranklPathway | [Bone Remodelling](http://cgap.nci.nih.gov/Pathways/BioCarta/h_ranklPathway) | [10](../../../../C:%5CDocuments%20and%20Settings%5CAdministrator%5CDesktop%5CDystro2009_all%20-Project%5COutput%5CFshdinf%20vs%20fshdPathwayClassComparison_Bioc_filtered%5CGeneSetGenesTable2.html#h_ranklPathway) | 0.20156 | 0.59844 | 0.135 (-) | 0.001411 |
| 129 | h_pdgfPathway | [PDGF Signaling Pathway](http://cgap.nci.nih.gov/Pathways/BioCarta/h_pdgfPathway) | [22](../../../../C:%5CDocuments%20and%20Settings%5CAdministrator%5CDesktop%5CDystro2009_all%20-Project%5COutput%5CFshdinf%20vs%20fshdPathwayClassComparison_Bioc_filtered%5CGeneSetGenesTable2.html#h_pdgfPathway) | 0.20259 | 0.19096 | 0.245 (+) | 0.0001427 |
| 130 | h_mPRPathway | [How Progesterone Initiates the Oocyte Maturation](http://cgap.nci.nih.gov/Pathways/BioCarta/h_mPRPathway) | [18](../../../../C:%5CDocuments%20and%20Settings%5CAdministrator%5CDesktop%5CDystro2009_all%20-Project%5COutput%5CFshdinf%20vs%20fshdPathwayClassComparison_Bioc_filtered%5CGeneSetGenesTable2.html#h_mPRPathway) | 0.20415 | 0.35214 | 0.26 (-) | 0.0003633 |
| 131 | h_eradPathway | [ER¿associated degradation (ERAD) Pathway](http://cgap.nci.nih.gov/Pathways/BioCarta/h_eradPathway) | [12](../../../../C:%5CDocuments%20and%20Settings%5CAdministrator%5CDesktop%5CDystro2009_all%20-Project%5COutput%5CFshdinf%20vs%20fshdPathwayClassComparison_Bioc_filtered%5CGeneSetGenesTable2.html#h_eradPathway) | 0.20581 | 0.14631 | 0.445 (-) | 0.0002386 |
| 132 | h_akapCentrosomePathway | [Protein Kinase A at the Centrosome](http://cgap.nci.nih.gov/Pathways/BioCarta/h_akapCentrosomePathway) | [12](../../../../C:%5CDocuments%20and%20Settings%5CAdministrator%5CDesktop%5CDystro2009_all%20-Project%5COutput%5CFshdinf%20vs%20fshdPathwayClassComparison_Bioc_filtered%5CGeneSetGenesTable2.html#h_akapCentrosomePathway) | 0.20611 | 0.57886 | 0.075 (+) | 0.0001648 |
| 133 | h_extrinsicPathway | [Extrinsic Prothrombin Activation Pathway](http://cgap.nci.nih.gov/Pathways/BioCarta/h_extrinsicPathway) | [8](../../../../C:%5CDocuments%20and%20Settings%5CAdministrator%5CDesktop%5CDystro2009_all%20-Project%5COutput%5CFshdinf%20vs%20fshdPathwayClassComparison_Bioc_filtered%5CGeneSetGenesTable2.html#h_extrinsicPathway) | 0.20893 | 0.07564 | 0.195 (-) | 0.0051623 |
| 134 | h_amiPathway | [Acute Myocardial Infarction](http://cgap.nci.nih.gov/Pathways/BioCarta/h_amiPathway) | [9](../../../../C:%5CDocuments%20and%20Settings%5CAdministrator%5CDesktop%5CDystro2009_all%20-Project%5COutput%5CFshdinf%20vs%20fshdPathwayClassComparison_Bioc_filtered%5CGeneSetGenesTable2.html#h_amiPathway) | 0.21663 | 0.13611 | 0.31 (-) | 0.0032616 |
| 135 | h_aktPathway | [AKT Signaling Pathway](http://cgap.nci.nih.gov/Pathways/BioCarta/h_aktPathway) | [17](../../../../C:%5CDocuments%20and%20Settings%5CAdministrator%5CDesktop%5CDystro2009_all%20-Project%5COutput%5CFshdinf%20vs%20fshdPathwayClassComparison_Bioc_filtered%5CGeneSetGenesTable2.html#h_aktPathway) | 0.21923 | 0.09898 | 0.275 (+) | 0.0001784 |
| 136 | h_egfPathway | [EGF Signaling Pathway](http://cgap.nci.nih.gov/Pathways/BioCarta/h_egfPathway) | [23](../../../../C:%5CDocuments%20and%20Settings%5CAdministrator%5CDesktop%5CDystro2009_all%20-Project%5COutput%5CFshdinf%20vs%20fshdPathwayClassComparison_Bioc_filtered%5CGeneSetGenesTable2.html#h_egfPathway) | 0.21939 | 0.25096 | 0.205 (+) | 0.0002785 |
| 137 | h_mcmPathway | [CDK Regulation of DNA Replication](http://cgap.nci.nih.gov/Pathways/BioCarta/h_mcmPathway) | [6](../../../../C:%5CDocuments%20and%20Settings%5CAdministrator%5CDesktop%5CDystro2009_all%20-Project%5COutput%5CFshdinf%20vs%20fshdPathwayClassComparison_Bioc_filtered%5CGeneSetGenesTable2.html#h_mcmPathway) | 0.21959 | 0.53318 | 0.085 (+) | 0.0001017 |
| 138 | h_soddPathway | [SODD/TNFR1 Signaling Pathway](http://cgap.nci.nih.gov/Pathways/BioCarta/h_soddPathway) | [9](../../../../C:%5CDocuments%20and%20Settings%5CAdministrator%5CDesktop%5CDystro2009_all%20-Project%5COutput%5CFshdinf%20vs%20fshdPathwayClassComparison_Bioc_filtered%5CGeneSetGenesTable2.html#h_soddPathway) | 0.22123 | 0.71612 | 0.205 (-) | 0.0050077 |
| 139 | h_cell2cellPathway | [Cell to Cell Adhesion Signaling](http://cgap.nci.nih.gov/Pathways/BioCarta/h_cell2cellPathway) | [10](../../../../C:%5CDocuments%20and%20Settings%5CAdministrator%5CDesktop%5CDystro2009_all%20-Project%5COutput%5CFshdinf%20vs%20fshdPathwayClassComparison_Bioc_filtered%5CGeneSetGenesTable2.html#h_cell2cellPathway) | 0.23005 | 0.75547 | 0.185 (-) | 0.0017705 |
| 140 | h_cdc25Pathway | [cdc25 and chk1 Regulatory Pathway in response to DNA damage](http://cgap.nci.nih.gov/Pathways/BioCarta/h_cdc25Pathway) | [5](../../../../C:%5CDocuments%20and%20Settings%5CAdministrator%5CDesktop%5CDystro2009_all%20-Project%5COutput%5CFshdinf%20vs%20fshdPathwayClassComparison_Bioc_filtered%5CGeneSetGenesTable2.html#h_cdc25Pathway) | 0.23837 | 0.39069 | 0.27 (-) | 0.0065489 |
| 141 | h_CCR3Pathway | [CCR3 signaling in Eosinophils](http://cgap.nci.nih.gov/Pathways/BioCarta/h_CCR3Pathway) | [19](../../../../C:%5CDocuments%20and%20Settings%5CAdministrator%5CDesktop%5CDystro2009_all%20-Project%5COutput%5CFshdinf%20vs%20fshdPathwayClassComparison_Bioc_filtered%5CGeneSetGenesTable2.html#h_CCR3Pathway) | 0.24013 | 0.38982 | 0.16 (+) | 0.0002642 |
| 142 | h_eea1Pathway | [The role of FYVE-finger proteins in vesicle transport](http://cgap.nci.nih.gov/Pathways/BioCarta/h_eea1Pathway) | [7](../../../../C:%5CDocuments%20and%20Settings%5CAdministrator%5CDesktop%5CDystro2009_all%20-Project%5COutput%5CFshdinf%20vs%20fshdPathwayClassComparison_Bioc_filtered%5CGeneSetGenesTable2.html#h_eea1Pathway) | 0.24304 | 0.27224 | 0.405 (+) | 0.0028923 |
| 143 | h_p27Pathway | [Regulation of p27 Phosphorylation during Cell Cycle Progression](http://cgap.nci.nih.gov/Pathways/BioCarta/h_p27Pathway) | [12](../../../../C:%5CDocuments%20and%20Settings%5CAdministrator%5CDesktop%5CDystro2009_all%20-Project%5COutput%5CFshdinf%20vs%20fshdPathwayClassComparison_Bioc_filtered%5CGeneSetGenesTable2.html#h_p27Pathway) | 0.24797 | 0.16051 | 0.03 (+) | 0.0011503 |
| 144 | h_TPOPathway | [TPO Signaling Pathway](http://cgap.nci.nih.gov/Pathways/BioCarta/h_TPOPathway) | [18](../../../../C:%5CDocuments%20and%20Settings%5CAdministrator%5CDesktop%5CDystro2009_all%20-Project%5COutput%5CFshdinf%20vs%20fshdPathwayClassComparison_Bioc_filtered%5CGeneSetGenesTable2.html#h_TPOPathway) | 0.24951 | 0.3542 | 0.325 (+) | 0.0003017 |
| 145 | h_pkcPathway | [Activation of PKC through G protein coupled receptor](http://cgap.nci.nih.gov/Pathways/BioCarta/h_pkcPathway) | [5](../../../../C:%5CDocuments%20and%20Settings%5CAdministrator%5CDesktop%5CDystro2009_all%20-Project%5COutput%5CFshdinf%20vs%20fshdPathwayClassComparison_Bioc_filtered%5CGeneSetGenesTable2.html#h_pkcPathway) | 0.25302 | 0.59489 | 0.37 (+) | 0.0028684 |
| 146 | h_bard1Pathway | [BRCA1-dependent Ub-ligase activity](http://cgap.nci.nih.gov/Pathways/BioCarta/h_bard1Pathway) | [6](../../../../C:%5CDocuments%20and%20Settings%5CAdministrator%5CDesktop%5CDystro2009_all%20-Project%5COutput%5CFshdinf%20vs%20fshdPathwayClassComparison_Bioc_filtered%5CGeneSetGenesTable2.html#h_bard1Pathway) | 0.26213 | 0.33024 | 0.14 (+) | 0.0088057 |
| 147 | h_il5Pathway | [IL 5 Signaling Pathway](http://cgap.nci.nih.gov/Pathways/BioCarta/h_il5Pathway) | [7](../../../../C:%5CDocuments%20and%20Settings%5CAdministrator%5CDesktop%5CDystro2009_all%20-Project%5COutput%5CFshdinf%20vs%20fshdPathwayClassComparison_Bioc_filtered%5CGeneSetGenesTable2.html#h_il5Pathway) | 0.26506 | 0.39799 | 0.29 (-) | 0.0009779 |
| 148 | h_crebPathway | [Transcription factor CREB and its extracellular signals](http://cgap.nci.nih.gov/Pathways/BioCarta/h_crebPathway) | [17](../../../../C:%5CDocuments%20and%20Settings%5CAdministrator%5CDesktop%5CDystro2009_all%20-Project%5COutput%5CFshdinf%20vs%20fshdPathwayClassComparison_Bioc_filtered%5CGeneSetGenesTable2.html#h_crebPathway) | 0.26677 | 0.41719 | 0.145 (+) | 0.0002398 |
| 149 | h_npp1Pathway | [Regulators of Bone Mineralization](http://cgap.nci.nih.gov/Pathways/BioCarta/h_npp1Pathway) | [5](../../../../C:%5CDocuments%20and%20Settings%5CAdministrator%5CDesktop%5CDystro2009_all%20-Project%5COutput%5CFshdinf%20vs%20fshdPathwayClassComparison_Bioc_filtered%5CGeneSetGenesTable2.html#h_npp1Pathway) | 0.26842 | 0.30082 | 0.41 (-) | 0.0031791 |
| 150 | h_p53hypoxiaPathway | [Hypoxia and p53 in the Cardiovascular system](http://cgap.nci.nih.gov/Pathways/BioCarta/h_p53hypoxiaPathway) | [19](../../../../C:%5CDocuments%20and%20Settings%5CAdministrator%5CDesktop%5CDystro2009_all%20-Project%5COutput%5CFshdinf%20vs%20fshdPathwayClassComparison_Bioc_filtered%5CGeneSetGenesTable2.html#h_p53hypoxiaPathway) | 0.27558 | 0.54278 | 0.23 (-) | 0.0000533 |
| 151 | h_ptdinsPathway | [Phosphoinositides and their downstream targets.](http://cgap.nci.nih.gov/Pathways/BioCarta/h_ptdinsPathway) | [17](../../../../C:%5CDocuments%20and%20Settings%5CAdministrator%5CDesktop%5CDystro2009_all%20-Project%5COutput%5CFshdinf%20vs%20fshdPathwayClassComparison_Bioc_filtered%5CGeneSetGenesTable2.html#h_ptdinsPathway) | 0.27828 | 0.53651 | 0.41 (-) | 0.0006144 |
| 152 | h_ghPathway | [Growth Hormone Signaling Pathway](http://cgap.nci.nih.gov/Pathways/BioCarta/h_ghPathway) | [18](../../../../C:%5CDocuments%20and%20Settings%5CAdministrator%5CDesktop%5CDystro2009_all%20-Project%5COutput%5CFshdinf%20vs%20fshdPathwayClassComparison_Bioc_filtered%5CGeneSetGenesTable2.html#h_ghPathway) | 0.28175 | 0.27524 | 0.17 (+) | 0.0002846 |
| 153 | h_mrpPathway | [Multi-Drug Resistance Factors](http://cgap.nci.nih.gov/Pathways/BioCarta/h_mrpPathway) | [6](../../../../C:%5CDocuments%20and%20Settings%5CAdministrator%5CDesktop%5CDystro2009_all%20-Project%5COutput%5CFshdinf%20vs%20fshdPathwayClassComparison_Bioc_filtered%5CGeneSetGenesTable2.html#h_mrpPathway) | 0.28416 | 0.58767 | 0.46 (-) | 0.0003219 |
| 154 | h_intrinsicPathway | [Intrinsic Prothrombin Activation Pathway](http://cgap.nci.nih.gov/Pathways/BioCarta/h_intrinsicPathway) | [12](../../../../C:%5CDocuments%20and%20Settings%5CAdministrator%5CDesktop%5CDystro2009_all%20-Project%5COutput%5CFshdinf%20vs%20fshdPathwayClassComparison_Bioc_filtered%5CGeneSetGenesTable2.html#h_intrinsicPathway) | 0.28881 | 0.27683 | 0.29 (-) | 0.0000663 |
| 155 | h_Par1Pathway | [Thrombin signaling and protease-activated receptors](http://cgap.nci.nih.gov/Pathways/BioCarta/h_Par1Pathway) | [16](../../../../C:%5CDocuments%20and%20Settings%5CAdministrator%5CDesktop%5CDystro2009_all%20-Project%5COutput%5CFshdinf%20vs%20fshdPathwayClassComparison_Bioc_filtered%5CGeneSetGenesTable2.html#h_Par1Pathway) | 0.30643 | 0.40249 | 0.155 (+) | 0.0006684 |
| 156 | h_reelinPathway | [Reelin Signaling Pathway](http://cgap.nci.nih.gov/Pathways/BioCarta/h_reelinPathway) | [5](../../../../C:%5CDocuments%20and%20Settings%5CAdministrator%5CDesktop%5CDystro2009_all%20-Project%5COutput%5CFshdinf%20vs%20fshdPathwayClassComparison_Bioc_filtered%5CGeneSetGenesTable2.html#h_reelinPathway) | 0.31368 | 0.69197 | 0.23 (-) | 0.0002044 |
| 157 | h_rhoPathway | [Rho cell motility signaling pathway](http://cgap.nci.nih.gov/Pathways/BioCarta/h_rhoPathway) | [20](../../../../C:%5CDocuments%20and%20Settings%5CAdministrator%5CDesktop%5CDystro2009_all%20-Project%5COutput%5CFshdinf%20vs%20fshdPathwayClassComparison_Bioc_filtered%5CGeneSetGenesTable2.html#h_rhoPathway) | 0.32506 | 0.45908 | 0.37 (-) | 0.0016845 |
| 158 | h_Ccr5Pathway | [Pertussis toxin-insensitive CCR5 Signaling in Macrophage](http://cgap.nci.nih.gov/Pathways/BioCarta/h_Ccr5Pathway) | [10](../../../../C:%5CDocuments%20and%20Settings%5CAdministrator%5CDesktop%5CDystro2009_all%20-Project%5COutput%5CFshdinf%20vs%20fshdPathwayClassComparison_Bioc_filtered%5CGeneSetGenesTable2.html#h_Ccr5Pathway) | 0.33297 | 0.52291 | 0.315 (-) | 0.0028468 |
| 159 | h_pmlPathway | [Regulation of transcriptional activity by PML](http://cgap.nci.nih.gov/Pathways/BioCarta/h_pmlPathway) | [13](../../../../C:%5CDocuments%20and%20Settings%5CAdministrator%5CDesktop%5CDystro2009_all%20-Project%5COutput%5CFshdinf%20vs%20fshdPathwayClassComparison_Bioc_filtered%5CGeneSetGenesTable2.html#h_pmlPathway) | 0.34539 | 0.53084 | 0.415 (-) | 0.0003109 |
| 160 | h_myosinPathway | [PKC-catalyzed phosphorylation of inhibitory phosphoprotein of myosin phosphatase](http://cgap.nci.nih.gov/Pathways/BioCarta/h_myosinPathway) | [13](../../../../C:%5CDocuments%20and%20Settings%5CAdministrator%5CDesktop%5CDystro2009_all%20-Project%5COutput%5CFshdinf%20vs%20fshdPathwayClassComparison_Bioc_filtered%5CGeneSetGenesTable2.html#h_myosinPathway) | 0.34665 | 0.69953 | 0.315 (+) | 0.0047739 |
| 161 | h_DNAfragmentPathway | [Apoptotic DNA fragmentation and tissue homeostasis](http://cgap.nci.nih.gov/Pathways/BioCarta/h_DNAfragmentPathway) | [7](../../../../C:%5CDocuments%20and%20Settings%5CAdministrator%5CDesktop%5CDystro2009_all%20-Project%5COutput%5CFshdinf%20vs%20fshdPathwayClassComparison_Bioc_filtered%5CGeneSetGenesTable2.html#h_DNAfragmentPathway) | 0.34932 | 0.35305 | 0.44 (+) | 0.0021068 |
| 162 | h_nuclearRsPathway | [Nuclear Receptors in Lipid Metabolism and Toxicity](http://cgap.nci.nih.gov/Pathways/BioCarta/h_nuclearRsPathway) | [21](../../../../C:%5CDocuments%20and%20Settings%5CAdministrator%5CDesktop%5CDystro2009_all%20-Project%5COutput%5CFshdinf%20vs%20fshdPathwayClassComparison_Bioc_filtered%5CGeneSetGenesTable2.html#h_nuclearRsPathway) | 0.36197 | 0.21545 | 0.48 (-) | 0.0013509 |
| 163 | h_carm-erPathway | [CARM1 and Regulation of the Estrogen Receptor](http://cgap.nci.nih.gov/Pathways/BioCarta/h_carm-erPathway) | [21](../../../../C:%5CDocuments%20and%20Settings%5CAdministrator%5CDesktop%5CDystro2009_all%20-Project%5COutput%5CFshdinf%20vs%20fshdPathwayClassComparison_Bioc_filtered%5CGeneSetGenesTable2.html#h_carm-erPathway) | 0.368 | 0.68339 | 0.08 (+) | 0.0000373 |
| 164 | h_hSWI-SNFpathway | [Chromatin Remodeling by hSWI/SNF ATP-dependent Complexes](http://cgap.nci.nih.gov/Pathways/BioCarta/h_hSWI-SNFpathway) | [14](../../../../C:%5CDocuments%20and%20Settings%5CAdministrator%5CDesktop%5CDystro2009_all%20-Project%5COutput%5CFshdinf%20vs%20fshdPathwayClassComparison_Bioc_filtered%5CGeneSetGenesTable2.html#h_hSWI-SNFpathway) | 0.37387 | 0.72383 | 0.425 (+) | 0.0004429 |
| 165 | h_cxcr4Pathway | [CXCR4 Signaling Pathway](http://cgap.nci.nih.gov/Pathways/BioCarta/h_cxcr4Pathway) | [21](../../../../C:%5CDocuments%20and%20Settings%5CAdministrator%5CDesktop%5CDystro2009_all%20-Project%5COutput%5CFshdinf%20vs%20fshdPathwayClassComparison_Bioc_filtered%5CGeneSetGenesTable2.html#h_cxcr4Pathway) | 0.37971 | 0.78425 | 0.42 (+) | 0.0001333 |
| 166 | h_malPathway | [Role of MAL in Rho-Mediated Activation of SRF](http://cgap.nci.nih.gov/Pathways/BioCarta/h_malPathway) | [15](../../../../C:%5CDocuments%20and%20Settings%5CAdministrator%5CDesktop%5CDystro2009_all%20-Project%5COutput%5CFshdinf%20vs%20fshdPathwayClassComparison_Bioc_filtered%5CGeneSetGenesTable2.html#h_malPathway) | 0.38318 | 0.26708 | 0.475 (+) | 0.0025161 |
| 167 | h_epoPathway | [EPO Signaling Pathway](http://cgap.nci.nih.gov/Pathways/BioCarta/h_epoPathway) | [15](../../../../C:%5CDocuments%20and%20Settings%5CAdministrator%5CDesktop%5CDystro2009_all%20-Project%5COutput%5CFshdinf%20vs%20fshdPathwayClassComparison_Bioc_filtered%5CGeneSetGenesTable2.html#h_epoPathway) | 0.38413 | 0.47306 | 0.355 (+) | 0.0008814 |
| 168 | h_integrinPathway | [Integrin Signaling Pathway](http://cgap.nci.nih.gov/Pathways/BioCarta/h_integrinPathway) | [32](../../../../C:%5CDocuments%20and%20Settings%5CAdministrator%5CDesktop%5CDystro2009_all%20-Project%5COutput%5CFshdinf%20vs%20fshdPathwayClassComparison_Bioc_filtered%5CGeneSetGenesTable2.html#h_integrinPathway) | 0.39481 | 0.38491 | 0.47 (+) | 0.001978 |
| 169 | h_il3Pathway | [IL 3 signaling pathway](http://cgap.nci.nih.gov/Pathways/BioCarta/h_il3Pathway) | [12](../../../../C:%5CDocuments%20and%20Settings%5CAdministrator%5CDesktop%5CDystro2009_all%20-Project%5COutput%5CFshdinf%20vs%20fshdPathwayClassComparison_Bioc_filtered%5CGeneSetGenesTable2.html#h_il3Pathway) | 0.407 | 0.75561 | 0.435 (+) | 0.0007595 |
| 170 | h_etsPathway | [METS affect on Macrophage Differentiation](http://cgap.nci.nih.gov/Pathways/BioCarta/h_etsPathway) | [12](../../../../C:%5CDocuments%20and%20Settings%5CAdministrator%5CDesktop%5CDystro2009_all%20-Project%5COutput%5CFshdinf%20vs%20fshdPathwayClassComparison_Bioc_filtered%5CGeneSetGenesTable2.html#h_etsPathway) | 0.40726 | 0.26524 | 0.325 (+) | 0.0026034 |
| 171 | h_prc2Pathway | [The PRC2 Complex Sets Long-term Gene Silencing Through Modification of Histone Tails](http://cgap.nci.nih.gov/Pathways/BioCarta/h_prc2Pathway) | [12](../../../../C:%5CDocuments%20and%20Settings%5CAdministrator%5CDesktop%5CDystro2009_all%20-Project%5COutput%5CFshdinf%20vs%20fshdPathwayClassComparison_Bioc_filtered%5CGeneSetGenesTable2.html#h_prc2Pathway) | 0.4213 | 0.17391 | 0.365 (+) | 0.0077198 |
| 172 | h_vegfPathway | [VEGF, Hypoxia, and Angiogenesis](http://cgap.nci.nih.gov/Pathways/BioCarta/h_vegfPathway) | [14](../../../../C:%5CDocuments%20and%20Settings%5CAdministrator%5CDesktop%5CDystro2009_all%20-Project%5COutput%5CFshdinf%20vs%20fshdPathwayClassComparison_Bioc_filtered%5CGeneSetGenesTable2.html#h_vegfPathway) | 0.42142 | 0.45791 | 0.13 (+) | 0.0005431 |
| 173 | h_erk5Pathway | [Role of Erk5 in Neuronal Survival](http://cgap.nci.nih.gov/Pathways/BioCarta/h_erk5Pathway) | [13](../../../../C:%5CDocuments%20and%20Settings%5CAdministrator%5CDesktop%5CDystro2009_all%20-Project%5COutput%5CFshdinf%20vs%20fshdPathwayClassComparison_Bioc_filtered%5CGeneSetGenesTable2.html#h_erk5Pathway) | 0.42254 | 0.4526 | 0.405 (+) | 0.0000731 |
| 174 | h_bcellsurvivalPathway | [B Cell Survival Pathway](http://cgap.nci.nih.gov/Pathways/BioCarta/h_bcellsurvivalPathway) | [10](../../../../C:%5CDocuments%20and%20Settings%5CAdministrator%5CDesktop%5CDystro2009_all%20-Project%5COutput%5CFshdinf%20vs%20fshdPathwayClassComparison_Bioc_filtered%5CGeneSetGenesTable2.html#h_bcellsurvivalPathway) | 0.4319 | 0.02856 | 0.295 (-) | 0.0036288 |
| 175 | h_chrebpPathway | [ChREBP regulation by carbohydrates and cAMP](http://cgap.nci.nih.gov/Pathways/BioCarta/h_chrebpPathway) | [8](../../../../C:%5CDocuments%20and%20Settings%5CAdministrator%5CDesktop%5CDystro2009_all%20-Project%5COutput%5CFshdinf%20vs%20fshdPathwayClassComparison_Bioc_filtered%5CGeneSetGenesTable2.html#h_chrebpPathway) | 0.43759 | 0.47511 | 0.435 (+) | 0.0004856 |
| 176 | h_calcineurinPathway | [Effects of calcineurin in Keratinocyte Differentiation](http://cgap.nci.nih.gov/Pathways/BioCarta/h_calcineurinPathway) | [9](../../../../C:%5CDocuments%20and%20Settings%5CAdministrator%5CDesktop%5CDystro2009_all%20-Project%5COutput%5CFshdinf%20vs%20fshdPathwayClassComparison_Bioc_filtered%5CGeneSetGenesTable2.html#h_calcineurinPathway) | 0.45439 | 0.9033 | 0.42 (-) | 0.0000755 |
| 177 | h_ck1Pathway | [Regulation of ck1/cdk5 by type 1 glutamate receptors](http://cgap.nci.nih.gov/Pathways/BioCarta/h_ck1Pathway) | [9](../../../../C:%5CDocuments%20and%20Settings%5CAdministrator%5CDesktop%5CDystro2009_all%20-Project%5COutput%5CFshdinf%20vs%20fshdPathwayClassComparison_Bioc_filtered%5CGeneSetGenesTable2.html#h_ck1Pathway) | 0.45557 | 0.37738 | 0.175 (+) | 0.0040249 |
| 178 | h_erkPathway | [Erk1/Erk2 Mapk Signaling pathway](http://cgap.nci.nih.gov/Pathways/BioCarta/h_erkPathway) | [27](../../../../C:%5CDocuments%20and%20Settings%5CAdministrator%5CDesktop%5CDystro2009_all%20-Project%5COutput%5CFshdinf%20vs%20fshdPathwayClassComparison_Bioc_filtered%5CGeneSetGenesTable2.html#h_erkPathway) | 0.45757 | 0.57853 | 0.26 (-) | 0.0011969 |
| 179 | h_her2Pathway | [Role of ERBB2 in Signal Transduction and Oncology](http://cgap.nci.nih.gov/Pathways/BioCarta/h_her2Pathway) | [19](../../../../C:%5CDocuments%20and%20Settings%5CAdministrator%5CDesktop%5CDystro2009_all%20-Project%5COutput%5CFshdinf%20vs%20fshdPathwayClassComparison_Bioc_filtered%5CGeneSetGenesTable2.html#h_her2Pathway) | 0.46553 | 0.34122 | 0.215 (+) | 0.0006998 |
| 180 | h_erythPathway | [Erythrocyte Differentiation Pathway](http://cgap.nci.nih.gov/Pathways/BioCarta/h_erythPathway) | [6](../../../../C:%5CDocuments%20and%20Settings%5CAdministrator%5CDesktop%5CDystro2009_all%20-Project%5COutput%5CFshdinf%20vs%20fshdPathwayClassComparison_Bioc_filtered%5CGeneSetGenesTable2.html#h_erythPathway) | 0.473 | 0.52491 | 0.33 (+) | 0.0084307 |
| 181 | h_tffPathway | [Trefoil Factors Initiate Mucosal Healing](http://cgap.nci.nih.gov/Pathways/BioCarta/h_tffPathway) | [20](../../../../C:%5CDocuments%20and%20Settings%5CAdministrator%5CDesktop%5CDystro2009_all%20-Project%5COutput%5CFshdinf%20vs%20fshdPathwayClassComparison_Bioc_filtered%5CGeneSetGenesTable2.html#h_tffPathway) | 0.47457 | 0.4142 | 0.425 (-) | 0.0001281 |
| 182 | h_fasPathway | [FAS signaling pathway ( CD95 )](http://cgap.nci.nih.gov/Pathways/BioCarta/h_fasPathway) | [26](../../../../C:%5CDocuments%20and%20Settings%5CAdministrator%5CDesktop%5CDystro2009_all%20-Project%5COutput%5CFshdinf%20vs%20fshdPathwayClassComparison_Bioc_filtered%5CGeneSetGenesTable2.html#h_fasPathway) | 0.50195 | 0.828 | 0.49 (-) | 0.000026 |
| 183 | h_il6Pathway | [IL 6 signaling pathway](http://cgap.nci.nih.gov/Pathways/BioCarta/h_il6Pathway) | [16](../../../../C:%5CDocuments%20and%20Settings%5CAdministrator%5CDesktop%5CDystro2009_all%20-Project%5COutput%5CFshdinf%20vs%20fshdPathwayClassComparison_Bioc_filtered%5CGeneSetGenesTable2.html#h_il6Pathway) | 0.50403 | 0.35784 | 0.115 (+) | 0.0007374 |
| 184 | h_gpcrPathway | [Signaling Pathway from G-Protein Families](http://cgap.nci.nih.gov/Pathways/BioCarta/h_gpcrPathway) | [20](../../../../C:%5CDocuments%20and%20Settings%5CAdministrator%5CDesktop%5CDystro2009_all%20-Project%5COutput%5CFshdinf%20vs%20fshdPathwayClassComparison_Bioc_filtered%5CGeneSetGenesTable2.html#h_gpcrPathway) | 0.50744 | 0.81368 | 0.275 (+) | 0.0018106 |
| 185 | h_wntPathway | [WNT Signaling Pathway](http://cgap.nci.nih.gov/Pathways/BioCarta/h_wntPathway) | [19](../../../../C:%5CDocuments%20and%20Settings%5CAdministrator%5CDesktop%5CDystro2009_all%20-Project%5COutput%5CFshdinf%20vs%20fshdPathwayClassComparison_Bioc_filtered%5CGeneSetGenesTable2.html#h_wntPathway) | 0.51717 | 0.37584 | 0.15 (+) | 0.0007173 |
| 186 | h_pparaPathway | [Mechanism of Gene Regulation by Peroxisome Proliferators via PPARa(alpha)](http://cgap.nci.nih.gov/Pathways/BioCarta/h_pparaPathway) | [35](../../../../C:%5CDocuments%20and%20Settings%5CAdministrator%5CDesktop%5CDystro2009_all%20-Project%5COutput%5CFshdinf%20vs%20fshdPathwayClassComparison_Bioc_filtered%5CGeneSetGenesTable2.html#h_pparaPathway) | 0.52501 | 0.66474 | 0.255 (+) | 0.000538 |
| 187 | h_SARSpathway | [SARS Coronavirus Protease](http://cgap.nci.nih.gov/Pathways/BioCarta/h_SARSpathway) | [7](../../../../C:%5CDocuments%20and%20Settings%5CAdministrator%5CDesktop%5CDystro2009_all%20-Project%5COutput%5CFshdinf%20vs%20fshdPathwayClassComparison_Bioc_filtered%5CGeneSetGenesTable2.html#h_SARSpathway) | 0.52926 | 0.71953 | 0.245 (+) | 0.0016019 |
| 188 | h_vipPathway | [Neuropeptides VIP and PACAP inhibit the apoptosis of activated T cells](http://cgap.nci.nih.gov/Pathways/BioCarta/h_vipPathway) | [13](../../../../C:%5CDocuments%20and%20Settings%5CAdministrator%5CDesktop%5CDystro2009_all%20-Project%5COutput%5CFshdinf%20vs%20fshdPathwayClassComparison_Bioc_filtered%5CGeneSetGenesTable2.html#h_vipPathway) | 0.54461 | 0.34941 | 0.27 (+) | 0.0041807 |
| 189 | h_trkaPathway | [Trka Receptor Signaling Pathway](http://cgap.nci.nih.gov/Pathways/BioCarta/h_trkaPathway) | [11](../../../../C:%5CDocuments%20and%20Settings%5CAdministrator%5CDesktop%5CDystro2009_all%20-Project%5COutput%5CFshdinf%20vs%20fshdPathwayClassComparison_Bioc_filtered%5CGeneSetGenesTable2.html#h_trkaPathway) | 0.54547 | 0.60826 | 0.39 (+) | 0.0001544 |
| 190 | h_pyk2Pathway | [Links between Pyk2 and Map Kinases](http://cgap.nci.nih.gov/Pathways/BioCarta/h_pyk2Pathway) | [22](../../../../C:%5CDocuments%20and%20Settings%5CAdministrator%5CDesktop%5CDystro2009_all%20-Project%5COutput%5CFshdinf%20vs%20fshdPathwayClassComparison_Bioc_filtered%5CGeneSetGenesTable2.html#h_pyk2Pathway) | 0.56085 | 0.76607 | 0.305 (+) | 0.0096224 |
| 191 | h_ace2Pathway | [Angiotensin-converting enzyme 2 regulates heart function](http://cgap.nci.nih.gov/Pathways/BioCarta/h_ace2Pathway) | [5](../../../../C:%5CDocuments%20and%20Settings%5CAdministrator%5CDesktop%5CDystro2009_all%20-Project%5COutput%5CFshdinf%20vs%20fshdPathwayClassComparison_Bioc_filtered%5CGeneSetGenesTable2.html#h_ace2Pathway) | 0.56597 | 0.72006 | 0.185 (+) | 0.0032433 |
| 192 | h_carm1Pathway | [Transcription Regulation by Methyltransferase of CARM1](http://cgap.nci.nih.gov/Pathways/BioCarta/h_carm1Pathway) | [8](../../../../C:%5CDocuments%20and%20Settings%5CAdministrator%5CDesktop%5CDystro2009_all%20-Project%5COutput%5CFshdinf%20vs%20fshdPathwayClassComparison_Bioc_filtered%5CGeneSetGenesTable2.html#h_carm1Pathway) | 0.56914 | 0.11537 | 0.255 (+) | 0.006641 |
| 193 | h_ngfPathway | [Nerve growth factor pathway (NGF)](http://cgap.nci.nih.gov/Pathways/BioCarta/h_ngfPathway) | [14](../../../../C:%5CDocuments%20and%20Settings%5CAdministrator%5CDesktop%5CDystro2009_all%20-Project%5COutput%5CFshdinf%20vs%20fshdPathwayClassComparison_Bioc_filtered%5CGeneSetGenesTable2.html#h_ngfPathway) | 0.57415 | 0.60101 | 0.24 (+) | 0.0011068 |
| 194 | h_hifPathway | [Hypoxia-Inducible Factor in the Cardiovascular System](http://cgap.nci.nih.gov/Pathways/BioCarta/h_hifPathway) | [13](../../../../C:%5CDocuments%20and%20Settings%5CAdministrator%5CDesktop%5CDystro2009_all%20-Project%5COutput%5CFshdinf%20vs%20fshdPathwayClassComparison_Bioc_filtered%5CGeneSetGenesTable2.html#h_hifPathway) | 0.58172 | 0.78161 | 0.145 (+) | 0.0014996 |
| 195 | h_metPathway | [Signaling of Hepatocyte Growth Factor Receptor](http://cgap.nci.nih.gov/Pathways/BioCarta/h_metPathway) | [31](../../../../C:%5CDocuments%20and%20Settings%5CAdministrator%5CDesktop%5CDystro2009_all%20-Project%5COutput%5CFshdinf%20vs%20fshdPathwayClassComparison_Bioc_filtered%5CGeneSetGenesTable2.html#h_metPathway) | 0.59418 | 0.50088 | 0.465 (-) | 0.0033987 |
| 196 | h_At1rPathway | [Angiotensin II mediated activation of JNK Pathway via Pyk2 dependent signaling](http://cgap.nci.nih.gov/Pathways/BioCarta/h_At1rPathway) | [24](../../../../C:%5CDocuments%20and%20Settings%5CAdministrator%5CDesktop%5CDystro2009_all%20-Project%5COutput%5CFshdinf%20vs%20fshdPathwayClassComparison_Bioc_filtered%5CGeneSetGenesTable2.html#h_At1rPathway) | 0.60108 | 0.84236 | 0.325 (+) | 0.0073552 |
| 197 | h_rabPathway | [Rab GTPases Mark Targets In The Endocytotic Machinery](http://cgap.nci.nih.gov/Pathways/BioCarta/h_rabPathway) | [12](../../../../C:%5CDocuments%20and%20Settings%5CAdministrator%5CDesktop%5CDystro2009_all%20-Project%5COutput%5CFshdinf%20vs%20fshdPathwayClassComparison_Bioc_filtered%5CGeneSetGenesTable2.html#h_rabPathway) | 0.61019 | 0.87385 | 0.315 (+) | 0.0002105 |
| 198 | h_PDZsPathway | [Synaptic Proteins at the Synaptic Junction](http://cgap.nci.nih.gov/Pathways/BioCarta/h_PDZsPathway) | [13](../../../../C:%5CDocuments%20and%20Settings%5CAdministrator%5CDesktop%5CDystro2009_all%20-Project%5COutput%5CFshdinf%20vs%20fshdPathwayClassComparison_Bioc_filtered%5CGeneSetGenesTable2.html#h_PDZsPathway) | 0.65052 | 0.84002 | 0.26 (+) | 0.0011712 |
| 199 | h_tgfbPathway | [TGF beta signaling pathway](http://cgap.nci.nih.gov/Pathways/BioCarta/h_tgfbPathway) | [15](../../../../C:%5CDocuments%20and%20Settings%5CAdministrator%5CDesktop%5CDystro2009_all%20-Project%5COutput%5CFshdinf%20vs%20fshdPathwayClassComparison_Bioc_filtered%5CGeneSetGenesTable2.html#h_tgfbPathway) | 0.65093 | 0.5732 | 0.46 (+) | 0.0003487 |
| 200 | h_hsp27Pathway | [Stress Induction of HSP Regulation](http://cgap.nci.nih.gov/Pathways/BioCarta/h_hsp27Pathway) | [12](../../../../C:%5CDocuments%20and%20Settings%5CAdministrator%5CDesktop%5CDystro2009_all%20-Project%5COutput%5CFshdinf%20vs%20fshdPathwayClassComparison_Bioc_filtered%5CGeneSetGenesTable2.html#h_hsp27Pathway) | 0.65129 | 0.35347 | 0.295 (+) | 0.0023846 |
| 201 | h_Lis1Pathway | [Lissencephaly gene (LIS1) in neuronal migration and development](http://cgap.nci.nih.gov/Pathways/BioCarta/h_Lis1Pathway) | [10](../../../../C:%5CDocuments%20and%20Settings%5CAdministrator%5CDesktop%5CDystro2009_all%20-Project%5COutput%5CFshdinf%20vs%20fshdPathwayClassComparison_Bioc_filtered%5CGeneSetGenesTable3.html#h_Lis1Pathway) | 0.67503 | 0.26643 | 0.29 (-) | 0.0066671 |
| 202 | h_inflamPathway | [Cytokines and Inflammatory Response](http://cgap.nci.nih.gov/Pathways/BioCarta/h_inflamPathway) | [15](../../../../C:%5CDocuments%20and%20Settings%5CAdministrator%5CDesktop%5CDystro2009_all%20-Project%5COutput%5CFshdinf%20vs%20fshdPathwayClassComparison_Bioc_filtered%5CGeneSetGenesTable3.html#h_inflamPathway) | 0.72275 | 0.55646 | 0.375 (+) | 0.001204 |
| 203 | h_pitx2Pathway | [Multi-step Regulation of Transcription by Pitx2](http://cgap.nci.nih.gov/Pathways/BioCarta/h_pitx2Pathway) | [10](../../../../C:%5CDocuments%20and%20Settings%5CAdministrator%5CDesktop%5CDystro2009_all%20-Project%5COutput%5CFshdinf%20vs%20fshdPathwayClassComparison_Bioc_filtered%5CGeneSetGenesTable3.html#h_pitx2Pathway) | 0.73634 | 0.69374 | 0.255 (-) | 0.0076559 |
| 204 | h_RNApol3Pathway | [RNA polymerase III transcription](http://cgap.nci.nih.gov/Pathways/BioCarta/h_RNApol3Pathway) | [7](../../../../C:%5CDocuments%20and%20Settings%5CAdministrator%5CDesktop%5CDystro2009_all%20-Project%5COutput%5CFshdinf%20vs%20fshdPathwayClassComparison_Bioc_filtered%5CGeneSetGenesTable3.html#h_RNApol3Pathway) | 0.76819 | 0.76163 | 0.315 (+) | 0.0073713 |
| 205 | h_ranbp2Pathway | [Sumoylation by RanBP2 Regulates Transcriptional Repression](http://cgap.nci.nih.gov/Pathways/BioCarta/h_ranbp2Pathway) | [10](../../../../C:%5CDocuments%20and%20Settings%5CAdministrator%5CDesktop%5CDystro2009_all%20-Project%5COutput%5CFshdinf%20vs%20fshdPathwayClassComparison_Bioc_filtered%5CGeneSetGenesTable3.html#h_ranbp2Pathway) | 0.79633 | 0.792 | 0.45 (+) | 0.0089581 |
| 206 | h_vdrPathway | [Control of Gene Expression by Vitamin D Receptor](http://cgap.nci.nih.gov/Pathways/BioCarta/h_vdrPathway) | [21](../../../../C:%5CDocuments%20and%20Settings%5CAdministrator%5CDesktop%5CDystro2009_all%20-Project%5COutput%5CFshdinf%20vs%20fshdPathwayClassComparison_Bioc_filtered%5CGeneSetGenesTable3.html#h_vdrPathway) | 0.82985 | 0.47438 | 0.455 (+) | 0.00283 |

| Gene Ontology Gene Set Expression Comparison FSHD T2-STIR + vs. FSHD T2-STIR - | | | | | | | | |
| --- | --- | --- | --- | --- | --- | --- | --- | --- |
|  | **GO category** | **GO ontology** | **GO term** | **Number of genes** | **LS permutation p-value** | **KS permutation p-value** | **Efron-Tibshirani's GSA test p-value** | **Goeman's global test p-value** |
| 1 | GO:0002376 | BP | immune system process | [62](../../../../C:%5CDocuments%20and%20Settings%5CAdministrator%5CDesktop%5CDystro2009_all%20-Project%5COutput%5CGOClassComparison%20FSHD%20inf%20vs%20fshd%20filtered%5CGeneSetGenesTable1.html#GO:0002376) | 0.00042 | 0.00043 | < 0.005 (-) | 0.0025932 |
| 2 | GO:0006955 | BP | immune response | [57](../../../../C:%5CDocuments%20and%20Settings%5CAdministrator%5CDesktop%5CDystro2009_all%20-Project%5COutput%5CGOClassComparison%20FSHD%20inf%20vs%20fshd%20filtered%5CGeneSetGenesTable1.html#GO:0006955) | 0.00047 | 0.00058 | 0.025 (-) | 0.0030565 |
| 3 | GO:0019725 | BP | cellular homeostasis | [5](../../../../C:%5CDocuments%20and%20Settings%5CAdministrator%5CDesktop%5CDystro2009_all%20-Project%5COutput%5CGOClassComparison%20FSHD%20inf%20vs%20fshd%20filtered%5CGeneSetGenesTable1.html#GO:0019725) | 0.00507 | 0.00053 | < 0.005 (-) | 0.0024958 |
| 4 | GO:0042981 | BP | regulation of apoptosis | [8](../../../../C:%5CDocuments%20and%20Settings%5CAdministrator%5CDesktop%5CDystro2009_all%20-Project%5COutput%5CGOClassComparison%20FSHD%20inf%20vs%20fshd%20filtered%5CGeneSetGenesTable1.html#GO:0042981) | 0.01486 | 0.12693 | < 0.005 (-) | 0.0000243 |
| 5 | GO:0043067 | BP | regulation of programmed cell death | [8](../../../../C:%5CDocuments%20and%20Settings%5CAdministrator%5CDesktop%5CDystro2009_all%20-Project%5COutput%5CGOClassComparison%20FSHD%20inf%20vs%20fshd%20filtered%5CGeneSetGenesTable1.html#GO:0043067) | 0.01486 | 0.12693 | < 0.005 (-) | 0.0000243 |
| 6 | GO:0042611 | CC | MHC protein complex | [23](../../../../C:%5CDocuments%20and%20Settings%5CAdministrator%5CDesktop%5CDystro2009_all%20-Project%5COutput%5CGOClassComparison%20FSHD%20inf%20vs%20fshd%20filtered%5CGeneSetGenesTable1.html#GO:0042611) | 0.01511 | 0.0048 | 0.12 (-) | 0.0076753 |
| 7 | GO:0042592 | BP | homeostatic process | [6](../../../../C:%5CDocuments%20and%20Settings%5CAdministrator%5CDesktop%5CDystro2009_all%20-Project%5COutput%5CGOClassComparison%20FSHD%20inf%20vs%20fshd%20filtered%5CGeneSetGenesTable1.html#GO:0042592) | 0.01704 | 0.01493 | < 0.005 (-) | 0.0025172 |
| 8 | GO:0016874 | MF | ligase activity | [5](../../../../C:%5CDocuments%20and%20Settings%5CAdministrator%5CDesktop%5CDystro2009_all%20-Project%5COutput%5CGOClassComparison%20FSHD%20inf%20vs%20fshd%20filtered%5CGeneSetGenesTable1.html#GO:0016874) | 0.02328 | 0.01312 | < 0.005 (+) | 0.0002538 |
| 9 | GO:0005654 | CC | nucleoplasm | [5](../../../../C:%5CDocuments%20and%20Settings%5CAdministrator%5CDesktop%5CDystro2009_all%20-Project%5COutput%5CGOClassComparison%20FSHD%20inf%20vs%20fshd%20filtered%5CGeneSetGenesTable1.html#GO:0005654) | 0.02368 | 0.05224 | 0.005 (+) | 0.000214 |
| 10 | GO:0008283 | BP | cell proliferation | [8](../../../../C:%5CDocuments%20and%20Settings%5CAdministrator%5CDesktop%5CDystro2009_all%20-Project%5COutput%5CGOClassComparison%20FSHD%20inf%20vs%20fshd%20filtered%5CGeneSetGenesTable1.html#GO:0008283) | 0.02523 | 0.01461 | 0.01 (-) | 0.0013755 |
| 11 | GO:0019882 | BP | antigen processing and presentation | [25](../../../../C:%5CDocuments%20and%20Settings%5CAdministrator%5CDesktop%5CDystro2009_all%20-Project%5COutput%5CGOClassComparison%20FSHD%20inf%20vs%20fshd%20filtered%5CGeneSetGenesTable1.html#GO:0019882) | 0.02541 | 0.00613 | 0.135 (-) | 0.0078882 |
| 12 | GO:0016772 | MF | transferase activity, transferring phosphorus-containing groups | [7](../../../../C:%5CDocuments%20and%20Settings%5CAdministrator%5CDesktop%5CDystro2009_all%20-Project%5COutput%5CGOClassComparison%20FSHD%20inf%20vs%20fshd%20filtered%5CGeneSetGenesTable1.html#GO:0016772) | 0.02918 | 0.50268 | < 0.005 (+) | 0.0000043 |
| 13 | GO:0002504 | BP | antigen processing and presentation of peptide or polysaccharide antigen via MHC class II | [11](../../../../C:%5CDocuments%20and%20Settings%5CAdministrator%5CDesktop%5CDystro2009_all%20-Project%5COutput%5CGOClassComparison%20FSHD%20inf%20vs%20fshd%20filtered%5CGeneSetGenesTable1.html#GO:0002504) | 0.03122 | 0.02848 | 0.1 (-) | 0.0078331 |
| 14 | GO:0016614 | MF | oxidoreductase activity, acting on CH-OH group of donors | [5](../../../../C:%5CDocuments%20and%20Settings%5CAdministrator%5CDesktop%5CDystro2009_all%20-Project%5COutput%5CGOClassComparison%20FSHD%20inf%20vs%20fshd%20filtered%5CGeneSetGenesTable1.html#GO:0016614) | 0.03603 | 0.02619 | 0.025 (-) | 0.0039721 |
| 15 | GO:0016616 | MF | oxidoreductase activity, acting on the CH-OH group of donors, NAD or NADP as acceptor | [5](../../../../C:%5CDocuments%20and%20Settings%5CAdministrator%5CDesktop%5CDystro2009_all%20-Project%5COutput%5CGOClassComparison%20FSHD%20inf%20vs%20fshd%20filtered%5CGeneSetGenesTable1.html#GO:0016616) | 0.03603 | 0.02619 | 0.025 (-) | 0.0039721 |
| 16 | GO:0050793 | BP | regulation of developmental process | [13](../../../../C:%5CDocuments%20and%20Settings%5CAdministrator%5CDesktop%5CDystro2009_all%20-Project%5COutput%5CGOClassComparison%20FSHD%20inf%20vs%20fshd%20filtered%5CGeneSetGenesTable1.html#GO:0050793) | 0.03745 | 0.11158 | < 0.005 (-) | 0.0002375 |
| 17 | GO:0043234 | CC | protein complex | [37](../../../../C:%5CDocuments%20and%20Settings%5CAdministrator%5CDesktop%5CDystro2009_all%20-Project%5COutput%5CGOClassComparison%20FSHD%20inf%20vs%20fshd%20filtered%5CGeneSetGenesTable1.html#GO:0043234) | 0.03784 | 0.04612 | 0.105 (-) | 0.0058991 |
| 18 | GO:0009615 | BP | response to virus | [8](../../../../C:%5CDocuments%20and%20Settings%5CAdministrator%5CDesktop%5CDystro2009_all%20-Project%5COutput%5CGOClassComparison%20FSHD%20inf%20vs%20fshd%20filtered%5CGeneSetGenesTable1.html#GO:0009615) | 0.04346 | 0.01149 | 0.035 (-) | 0.0011466 |
| 19 | GO:0044419 | BP | interspecies interaction between organisms | [8](../../../../C:%5CDocuments%20and%20Settings%5CAdministrator%5CDesktop%5CDystro2009_all%20-Project%5COutput%5CGOClassComparison%20FSHD%20inf%20vs%20fshd%20filtered%5CGeneSetGenesTable1.html#GO:0044419) | 0.04865 | 0.03138 | 0.07 (-) | 0.005004 |
| 20 | GO:0001871 | MF | pattern binding | [5](../../../../C:%5CDocuments%20and%20Settings%5CAdministrator%5CDesktop%5CDystro2009_all%20-Project%5COutput%5CGOClassComparison%20FSHD%20inf%20vs%20fshd%20filtered%5CGeneSetGenesTable1.html#GO:0001871) | 0.0567 | 0.08867 | < 0.005 (+) | 0.0072593 |
| 21 | GO:0005539 | MF | glycosaminoglycan binding | [5](../../../../C:%5CDocuments%20and%20Settings%5CAdministrator%5CDesktop%5CDystro2009_all%20-Project%5COutput%5CGOClassComparison%20FSHD%20inf%20vs%20fshd%20filtered%5CGeneSetGenesTable1.html#GO:0005539) | 0.0567 | 0.08867 | < 0.005 (+) | 0.0072593 |
| 22 | GO:0030247 | MF | polysaccharide binding | [5](../../../../C:%5CDocuments%20and%20Settings%5CAdministrator%5CDesktop%5CDystro2009_all%20-Project%5COutput%5CGOClassComparison%20FSHD%20inf%20vs%20fshd%20filtered%5CGeneSetGenesTable1.html#GO:0030247) | 0.0567 | 0.08867 | < 0.005 (+) | 0.0072593 |
| 23 | GO:0016564 | MF | transcription repressor activity | [9](../../../../C:%5CDocuments%20and%20Settings%5CAdministrator%5CDesktop%5CDystro2009_all%20-Project%5COutput%5CGOClassComparison%20FSHD%20inf%20vs%20fshd%20filtered%5CGeneSetGenesTable1.html#GO:0016564) | 0.06807 | 0.13344 | 0.095 (-) | 0.000141 |
| 24 | GO:0005739 | CC | mitochondrion | [16](../../../../C:%5CDocuments%20and%20Settings%5CAdministrator%5CDesktop%5CDystro2009_all%20-Project%5COutput%5CGOClassComparison%20FSHD%20inf%20vs%20fshd%20filtered%5CGeneSetGenesTable1.html#GO:0005739) | 0.06838 | 0.16797 | 0.07 (+) | 0.0022437 |
| 25 | GO:0008219 | BP | cell death | [15](../../../../C:%5CDocuments%20and%20Settings%5CAdministrator%5CDesktop%5CDystro2009_all%20-Project%5COutput%5CGOClassComparison%20FSHD%20inf%20vs%20fshd%20filtered%5CGeneSetGenesTable1.html#GO:0008219) | 0.06926 | 0.28263 | 0.01 (-) | 0.000017 |
| 26 | GO:0016265 | BP | death | [15](../../../../C:%5CDocuments%20and%20Settings%5CAdministrator%5CDesktop%5CDystro2009_all%20-Project%5COutput%5CGOClassComparison%20FSHD%20inf%20vs%20fshd%20filtered%5CGeneSetGenesTable1.html#GO:0016265) | 0.06926 | 0.28263 | 0.01 (-) | 0.000017 |
| 27 | GO:0006915 | BP | apoptosis | [13](../../../../C:%5CDocuments%20and%20Settings%5CAdministrator%5CDesktop%5CDystro2009_all%20-Project%5COutput%5CGOClassComparison%20FSHD%20inf%20vs%20fshd%20filtered%5CGeneSetGenesTable1.html#GO:0006915) | 0.06967 | 0.27707 | < 0.005 (-) | 0.0000142 |
| 28 | GO:0012501 | BP | programmed cell death | [13](../../../../C:%5CDocuments%20and%20Settings%5CAdministrator%5CDesktop%5CDystro2009_all%20-Project%5COutput%5CGOClassComparison%20FSHD%20inf%20vs%20fshd%20filtered%5CGeneSetGenesTable1.html#GO:0012501) | 0.06967 | 0.27707 | < 0.005 (-) | 0.0000142 |
| 29 | GO:0044459 | CC | plasma membrane part | [46](../../../../C:%5CDocuments%20and%20Settings%5CAdministrator%5CDesktop%5CDystro2009_all%20-Project%5COutput%5CGOClassComparison%20FSHD%20inf%20vs%20fshd%20filtered%5CGeneSetGenesTable1.html#GO:0044459) | 0.07404 | 0.0013 | 0.13 (-) | 0.0072706 |
| 30 | GO:0006954 | BP | inflammatory response | [12](../../../../C:%5CDocuments%20and%20Settings%5CAdministrator%5CDesktop%5CDystro2009_all%20-Project%5COutput%5CGOClassComparison%20FSHD%20inf%20vs%20fshd%20filtered%5CGeneSetGenesTable1.html#GO:0006954) | 0.08041 | 0.03157 | 0.025 (-) | 0.001462 |
| 31 | GO:0009611 | BP | response to wounding | [12](../../../../C:%5CDocuments%20and%20Settings%5CAdministrator%5CDesktop%5CDystro2009_all%20-Project%5COutput%5CGOClassComparison%20FSHD%20inf%20vs%20fshd%20filtered%5CGeneSetGenesTable1.html#GO:0009611) | 0.08041 | 0.03157 | 0.025 (-) | 0.001462 |
| 32 | GO:0019941 | BP | modification-dependent protein catabolic process | [5](../../../../C:%5CDocuments%20and%20Settings%5CAdministrator%5CDesktop%5CDystro2009_all%20-Project%5COutput%5CGOClassComparison%20FSHD%20inf%20vs%20fshd%20filtered%5CGeneSetGenesTable1.html#GO:0019941) | 0.08203 | 0.11469 | < 0.005 (+) | 0.0006399 |
| 33 | GO:0030163 | BP | protein catabolic process | [5](../../../../C:%5CDocuments%20and%20Settings%5CAdministrator%5CDesktop%5CDystro2009_all%20-Project%5COutput%5CGOClassComparison%20FSHD%20inf%20vs%20fshd%20filtered%5CGeneSetGenesTable1.html#GO:0030163) | 0.08203 | 0.11469 | < 0.005 (+) | 0.0006399 |
| 34 | GO:0034962 | BP | cellular biopolymer catabolic process | [5](../../../../C:%5CDocuments%20and%20Settings%5CAdministrator%5CDesktop%5CDystro2009_all%20-Project%5COutput%5CGOClassComparison%20FSHD%20inf%20vs%20fshd%20filtered%5CGeneSetGenesTable1.html#GO:0034962) | 0.08203 | 0.11469 | < 0.005 (+) | 0.0006399 |
| 35 | GO:0043285 | BP | biopolymer catabolic process | [5](../../../../C:%5CDocuments%20and%20Settings%5CAdministrator%5CDesktop%5CDystro2009_all%20-Project%5COutput%5CGOClassComparison%20FSHD%20inf%20vs%20fshd%20filtered%5CGeneSetGenesTable1.html#GO:0043285) | 0.08203 | 0.11469 | < 0.005 (+) | 0.0006399 |
| 36 | GO:0043632 | BP | modification-dependent macromolecule catabolic process | [5](../../../../C:%5CDocuments%20and%20Settings%5CAdministrator%5CDesktop%5CDystro2009_all%20-Project%5COutput%5CGOClassComparison%20FSHD%20inf%20vs%20fshd%20filtered%5CGeneSetGenesTable1.html#GO:0043632) | 0.08203 | 0.11469 | < 0.005 (+) | 0.0006399 |
| 37 | GO:0044257 | BP | cellular protein catabolic process | [5](../../../../C:%5CDocuments%20and%20Settings%5CAdministrator%5CDesktop%5CDystro2009_all%20-Project%5COutput%5CGOClassComparison%20FSHD%20inf%20vs%20fshd%20filtered%5CGeneSetGenesTable1.html#GO:0044257) | 0.08203 | 0.11469 | < 0.005 (+) | 0.0006399 |
| 38 | GO:0051603 | BP | proteolysis involved in cellular protein catabolic process | [5](../../../../C:%5CDocuments%20and%20Settings%5CAdministrator%5CDesktop%5CDystro2009_all%20-Project%5COutput%5CGOClassComparison%20FSHD%20inf%20vs%20fshd%20filtered%5CGeneSetGenesTable1.html#GO:0051603) | 0.08203 | 0.11469 | < 0.005 (+) | 0.0006399 |
| 39 | GO:0051093 | BP | negative regulation of developmental process | [7](../../../../C:%5CDocuments%20and%20Settings%5CAdministrator%5CDesktop%5CDystro2009_all%20-Project%5COutput%5CGOClassComparison%20FSHD%20inf%20vs%20fshd%20filtered%5CGeneSetGenesTable1.html#GO:0051093) | 0.08333 | 0.21819 | 0.02 (-) | 0.0000323 |
| 40 | GO:0009605 | BP | response to external stimulus | [16](../../../../C:%5CDocuments%20and%20Settings%5CAdministrator%5CDesktop%5CDystro2009_all%20-Project%5COutput%5CGOClassComparison%20FSHD%20inf%20vs%20fshd%20filtered%5CGeneSetGenesTable1.html#GO:0009605) | 0.08463 | 0.04963 | 0.025 (-) | 0.0009416 |
| 41 | GO:0005887 | CC | integral to plasma membrane | [34](../../../../C:%5CDocuments%20and%20Settings%5CAdministrator%5CDesktop%5CDystro2009_all%20-Project%5COutput%5CGOClassComparison%20FSHD%20inf%20vs%20fshd%20filtered%5CGeneSetGenesTable1.html#GO:0005887) | 0.0892 | 0.02953 | 0.095 (-) | 0.0043586 |
| 42 | GO:0031226 | CC | intrinsic to plasma membrane | [34](../../../../C:%5CDocuments%20and%20Settings%5CAdministrator%5CDesktop%5CDystro2009_all%20-Project%5COutput%5CGOClassComparison%20FSHD%20inf%20vs%20fshd%20filtered%5CGeneSetGenesTable1.html#GO:0031226) | 0.0892 | 0.02953 | 0.095 (-) | 0.0043586 |
| 43 | GO:0006464 | BP | protein modification process | [5](../../../../C:%5CDocuments%20and%20Settings%5CAdministrator%5CDesktop%5CDystro2009_all%20-Project%5COutput%5CGOClassComparison%20FSHD%20inf%20vs%20fshd%20filtered%5CGeneSetGenesTable1.html#GO:0006464) | 0.12346 | 0.33965 | < 0.005 (+) | 0.0002037 |
| 44 | GO:0043412 | BP | biopolymer modification | [5](../../../../C:%5CDocuments%20and%20Settings%5CAdministrator%5CDesktop%5CDystro2009_all%20-Project%5COutput%5CGOClassComparison%20FSHD%20inf%20vs%20fshd%20filtered%5CGeneSetGenesTable1.html#GO:0043412) | 0.12346 | 0.33965 | < 0.005 (+) | 0.0002037 |
| 45 | GO:0002520 | BP | immune system development | [5](../../../../C:%5CDocuments%20and%20Settings%5CAdministrator%5CDesktop%5CDystro2009_all%20-Project%5COutput%5CGOClassComparison%20FSHD%20inf%20vs%20fshd%20filtered%5CGeneSetGenesTable1.html#GO:0002520) | 0.12505 | 0.27887 | 0.065 (-) | 0.0029444 |
| 46 | GO:0002521 | BP | leukocyte differentiation | [5](../../../../C:%5CDocuments%20and%20Settings%5CAdministrator%5CDesktop%5CDystro2009_all%20-Project%5COutput%5CGOClassComparison%20FSHD%20inf%20vs%20fshd%20filtered%5CGeneSetGenesTable1.html#GO:0002521) | 0.12505 | 0.27887 | 0.065 (-) | 0.0029444 |
| 47 | GO:0030097 | BP | hemopoiesis | [5](../../../../C:%5CDocuments%20and%20Settings%5CAdministrator%5CDesktop%5CDystro2009_all%20-Project%5COutput%5CGOClassComparison%20FSHD%20inf%20vs%20fshd%20filtered%5CGeneSetGenesTable1.html#GO:0030097) | 0.12505 | 0.27887 | 0.065 (-) | 0.0029444 |
| 48 | GO:0048534 | BP | hemopoietic or lymphoid organ development | [5](../../../../C:%5CDocuments%20and%20Settings%5CAdministrator%5CDesktop%5CDystro2009_all%20-Project%5COutput%5CGOClassComparison%20FSHD%20inf%20vs%20fshd%20filtered%5CGeneSetGenesTable1.html#GO:0048534) | 0.12505 | 0.27887 | 0.065 (-) | 0.0029444 |
| 49 | GO:0043566 | MF | structure-specific DNA binding | [6](../../../../C:%5CDocuments%20and%20Settings%5CAdministrator%5CDesktop%5CDystro2009_all%20-Project%5COutput%5CGOClassComparison%20FSHD%20inf%20vs%20fshd%20filtered%5CGeneSetGenesTable1.html#GO:0043566) | 0.13815 | 0.37398 | 0.05 (+) | 0.0003399 |
| 50 | GO:0006974 | BP | response to DNA damage stimulus | [7](../../../../C:%5CDocuments%20and%20Settings%5CAdministrator%5CDesktop%5CDystro2009_all%20-Project%5COutput%5CGOClassComparison%20FSHD%20inf%20vs%20fshd%20filtered%5CGeneSetGenesTable1.html#GO:0006974) | 0.14555 | 0.29697 | 0.085 (+) | 0.0001323 |
| 51 | GO:0034984 | BP | cellular response to DNA damage stimulus | [7](../../../../C:%5CDocuments%20and%20Settings%5CAdministrator%5CDesktop%5CDystro2009_all%20-Project%5COutput%5CGOClassComparison%20FSHD%20inf%20vs%20fshd%20filtered%5CGeneSetGenesTable1.html#GO:0034984) | 0.14555 | 0.29697 | 0.085 (+) | 0.0001323 |
| 52 | GO:0002682 | BP | regulation of immune system process | [8](../../../../C:%5CDocuments%20and%20Settings%5CAdministrator%5CDesktop%5CDystro2009_all%20-Project%5COutput%5CGOClassComparison%20FSHD%20inf%20vs%20fshd%20filtered%5CGeneSetGenesTable1.html#GO:0002682) | 0.14901 | 0.02862 | 0.1 (-) | 0.0067013 |
| 53 | GO:0046983 | MF | protein dimerization activity | [5](../../../../C:%5CDocuments%20and%20Settings%5CAdministrator%5CDesktop%5CDystro2009_all%20-Project%5COutput%5CGOClassComparison%20FSHD%20inf%20vs%20fshd%20filtered%5CGeneSetGenesTable1.html#GO:0046983) | 0.14955 | 0.62166 | < 0.005 (+) | 0.0003086 |
| 54 | GO:0009057 | BP | macromolecule catabolic process | [6](../../../../C:%5CDocuments%20and%20Settings%5CAdministrator%5CDesktop%5CDystro2009_all%20-Project%5COutput%5CGOClassComparison%20FSHD%20inf%20vs%20fshd%20filtered%5CGeneSetGenesTable1.html#GO:0009057) | 0.16027 | 0.27561 | < 0.005 (+) | 0.002306 |
| 55 | GO:0044265 | BP | cellular macromolecule catabolic process | [6](../../../../C:%5CDocuments%20and%20Settings%5CAdministrator%5CDesktop%5CDystro2009_all%20-Project%5COutput%5CGOClassComparison%20FSHD%20inf%20vs%20fshd%20filtered%5CGeneSetGenesTable1.html#GO:0044265) | 0.16027 | 0.27561 | < 0.005 (+) | 0.002306 |
| 56 | GO:0003690 | MF | double-stranded DNA binding | [5](../../../../C:%5CDocuments%20and%20Settings%5CAdministrator%5CDesktop%5CDystro2009_all%20-Project%5COutput%5CGOClassComparison%20FSHD%20inf%20vs%20fshd%20filtered%5CGeneSetGenesTable1.html#GO:0003690) | 0.16777 | 0.41211 | 0.085 (+) | 0.0004245 |
| 57 | GO:0007267 | BP | cell-cell signaling | [12](../../../../C:%5CDocuments%20and%20Settings%5CAdministrator%5CDesktop%5CDystro2009_all%20-Project%5COutput%5CGOClassComparison%20FSHD%20inf%20vs%20fshd%20filtered%5CGeneSetGenesTable1.html#GO:0007267) | 0.1852 | 0.03157 | 0.125 (-) | 0.004244 |
| 58 | GO:0065008 | BP | regulation of biological quality | [14](../../../../C:%5CDocuments%20and%20Settings%5CAdministrator%5CDesktop%5CDystro2009_all%20-Project%5COutput%5CGOClassComparison%20FSHD%20inf%20vs%20fshd%20filtered%5CGeneSetGenesTable1.html#GO:0065008) | 0.18888 | 0.29879 | 0.04 (-) | 0.0006496 |
| 59 | GO:0033554 | BP | cellular response to stress | [8](../../../../C:%5CDocuments%20and%20Settings%5CAdministrator%5CDesktop%5CDystro2009_all%20-Project%5COutput%5CGOClassComparison%20FSHD%20inf%20vs%20fshd%20filtered%5CGeneSetGenesTable1.html#GO:0033554) | 0.21575 | 0.45957 | 0.075 (+) | 0.0001305 |
| 60 | GO:0051716 | BP | cellular response to stimulus | [8](../../../../C:%5CDocuments%20and%20Settings%5CAdministrator%5CDesktop%5CDystro2009_all%20-Project%5COutput%5CGOClassComparison%20FSHD%20inf%20vs%20fshd%20filtered%5CGeneSetGenesTable1.html#GO:0051716) | 0.21575 | 0.45957 | 0.075 (+) | 0.0001305 |
| 61 | GO:0030554 | MF | adenyl nucleotide binding | [22](../../../../C:%5CDocuments%20and%20Settings%5CAdministrator%5CDesktop%5CDystro2009_all%20-Project%5COutput%5CGOClassComparison%20FSHD%20inf%20vs%20fshd%20filtered%5CGeneSetGenesTable1.html#GO:0030554) | 0.2204 | 0.5996 | 0.045 (+) | 0.0001293 |
| 62 | GO:0048518 | BP | positive regulation of biological process | [14](../../../../C:%5CDocuments%20and%20Settings%5CAdministrator%5CDesktop%5CDystro2009_all%20-Project%5COutput%5CGOClassComparison%20FSHD%20inf%20vs%20fshd%20filtered%5CGeneSetGenesTable1.html#GO:0048518) | 0.22522 | 0.27298 | 0.11 (-) | 0.008006 |
| 63 | GO:0048523 | BP | negative regulation of cellular process | [24](../../../../C:%5CDocuments%20and%20Settings%5CAdministrator%5CDesktop%5CDystro2009_all%20-Project%5COutput%5CGOClassComparison%20FSHD%20inf%20vs%20fshd%20filtered%5CGeneSetGenesTable1.html#GO:0048523) | 0.22847 | 0.11557 | 0.08 (-) | 0.0008854 |
| 64 | GO:0048519 | BP | negative regulation of biological process | [27](../../../../C:%5CDocuments%20and%20Settings%5CAdministrator%5CDesktop%5CDystro2009_all%20-Project%5COutput%5CGOClassComparison%20FSHD%20inf%20vs%20fshd%20filtered%5CGeneSetGenesTable1.html#GO:0048519) | 0.23292 | 0.08231 | 0.055 (-) | 0.0010702 |
| 65 | GO:0017076 | MF | purine nucleotide binding | [27](../../../../C:%5CDocuments%20and%20Settings%5CAdministrator%5CDesktop%5CDystro2009_all%20-Project%5COutput%5CGOClassComparison%20FSHD%20inf%20vs%20fshd%20filtered%5CGeneSetGenesTable1.html#GO:0017076) | 0.24805 | 0.60729 | 0.04 (+) | 0.0000922 |
| 66 | GO:0048522 | BP | positive regulation of cellular process | [9](../../../../C:%5CDocuments%20and%20Settings%5CAdministrator%5CDesktop%5CDystro2009_all%20-Project%5COutput%5CGOClassComparison%20FSHD%20inf%20vs%20fshd%20filtered%5CGeneSetGenesTable1.html#GO:0048522) | 0.25186 | 0.25475 | 0.155 (-) | 0.0097811 |
| 67 | GO:0044248 | BP | cellular catabolic process | [8](../../../../C:%5CDocuments%20and%20Settings%5CAdministrator%5CDesktop%5CDystro2009_all%20-Project%5COutput%5CGOClassComparison%20FSHD%20inf%20vs%20fshd%20filtered%5CGeneSetGenesTable1.html#GO:0044248) | 0.26218 | 0.27652 | 0.045 (+) | 0.0024625 |
| 68 | GO:0048513 | BP | organ development | [15](../../../../C:%5CDocuments%20and%20Settings%5CAdministrator%5CDesktop%5CDystro2009_all%20-Project%5COutput%5CGOClassComparison%20FSHD%20inf%20vs%20fshd%20filtered%5CGeneSetGenesTable1.html#GO:0048513) | 0.26453 | 0.08472 | 0.11 (-) | 0.0018259 |
| 69 | GO:0001664 | MF | G-protein-coupled receptor binding | [9](../../../../C:%5CDocuments%20and%20Settings%5CAdministrator%5CDesktop%5CDystro2009_all%20-Project%5COutput%5CGOClassComparison%20FSHD%20inf%20vs%20fshd%20filtered%5CGeneSetGenesTable1.html#GO:0001664) | 0.2891 | 0.198 | 0.125 (-) | 0.0050663 |
| 70 | GO:0042379 | MF | chemokine receptor binding | [9](../../../../C:%5CDocuments%20and%20Settings%5CAdministrator%5CDesktop%5CDystro2009_all%20-Project%5COutput%5CGOClassComparison%20FSHD%20inf%20vs%20fshd%20filtered%5CGeneSetGenesTable1.html#GO:0042379) | 0.2891 | 0.198 | 0.125 (-) | 0.0050663 |
| 71 | GO:0006935 | BP | chemotaxis | [9](../../../../C:%5CDocuments%20and%20Settings%5CAdministrator%5CDesktop%5CDystro2009_all%20-Project%5COutput%5CGOClassComparison%20FSHD%20inf%20vs%20fshd%20filtered%5CGeneSetGenesTable1.html#GO:0006935) | 0.31758 | 0.198 | 0.135 (-) | 0.0051015 |
| 72 | GO:0007626 | BP | locomotory behavior | [9](../../../../C:%5CDocuments%20and%20Settings%5CAdministrator%5CDesktop%5CDystro2009_all%20-Project%5COutput%5CGOClassComparison%20FSHD%20inf%20vs%20fshd%20filtered%5CGeneSetGenesTable1.html#GO:0007626) | 0.31758 | 0.198 | 0.135 (-) | 0.0051015 |
| 73 | GO:0042330 | BP | taxis | [9](../../../../C:%5CDocuments%20and%20Settings%5CAdministrator%5CDesktop%5CDystro2009_all%20-Project%5COutput%5CGOClassComparison%20FSHD%20inf%20vs%20fshd%20filtered%5CGeneSetGenesTable1.html#GO:0042330) | 0.31758 | 0.198 | 0.135 (-) | 0.0051015 |
| 74 | GO:0000323 | CC | lytic vacuole | [6](../../../../C:%5CDocuments%20and%20Settings%5CAdministrator%5CDesktop%5CDystro2009_all%20-Project%5COutput%5CGOClassComparison%20FSHD%20inf%20vs%20fshd%20filtered%5CGeneSetGenesTable1.html#GO:0000323) | 0.32077 | 0.33027 | 0.13 (-) | 0.0015467 |
| 75 | GO:0005764 | CC | lysosome | [6](../../../../C:%5CDocuments%20and%20Settings%5CAdministrator%5CDesktop%5CDystro2009_all%20-Project%5COutput%5CGOClassComparison%20FSHD%20inf%20vs%20fshd%20filtered%5CGeneSetGenesTable1.html#GO:0005764) | 0.32077 | 0.33027 | 0.13 (-) | 0.0015467 |
| 76 | GO:0005773 | CC | vacuole | [6](../../../../C:%5CDocuments%20and%20Settings%5CAdministrator%5CDesktop%5CDystro2009_all%20-Project%5COutput%5CGOClassComparison%20FSHD%20inf%20vs%20fshd%20filtered%5CGeneSetGenesTable1.html#GO:0005773) | 0.32077 | 0.33027 | 0.13 (-) | 0.0015467 |
| 77 | GO:0051246 | BP | regulation of protein metabolic process | [5](../../../../C:%5CDocuments%20and%20Settings%5CAdministrator%5CDesktop%5CDystro2009_all%20-Project%5COutput%5CGOClassComparison%20FSHD%20inf%20vs%20fshd%20filtered%5CGeneSetGenesTable1.html#GO:0051246) | 0.34417 | 0.58893 | < 0.005 (+) | 0.0003129 |
| 78 | GO:0009056 | BP | catabolic process | [9](../../../../C:%5CDocuments%20and%20Settings%5CAdministrator%5CDesktop%5CDystro2009_all%20-Project%5COutput%5CGOClassComparison%20FSHD%20inf%20vs%20fshd%20filtered%5CGeneSetGenesTable1.html#GO:0009056) | 0.35392 | 0.42749 | 0.045 (+) | 0.0025263 |
| 79 | GO:0006950 | BP | response to stress | [49](../../../../C:%5CDocuments%20and%20Settings%5CAdministrator%5CDesktop%5CDystro2009_all%20-Project%5COutput%5CGOClassComparison%20FSHD%20inf%20vs%20fshd%20filtered%5CGeneSetGenesTable1.html#GO:0006950) | 0.35411 | 0.62745 | 0.075 (-) | 0.0001955 |
| 80 | GO:0006690 | BP | icosanoid metabolic process | [5](../../../../C:%5CDocuments%20and%20Settings%5CAdministrator%5CDesktop%5CDystro2009_all%20-Project%5COutput%5CGOClassComparison%20FSHD%20inf%20vs%20fshd%20filtered%5CGeneSetGenesTable1.html#GO:0006690) | 0.36261 | 0.59838 | 0.085 (-) | 0.0055936 |
| 81 | GO:0032559 | MF | adenyl ribonucleotide binding | [21](../../../../C:%5CDocuments%20and%20Settings%5CAdministrator%5CDesktop%5CDystro2009_all%20-Project%5COutput%5CGOClassComparison%20FSHD%20inf%20vs%20fshd%20filtered%5CGeneSetGenesTable1.html#GO:0032559) | 0.37219 | 0.6799 | 0.245 (+) | 0.0001381 |
| 82 | GO:0031981 | CC | nuclear lumen | [22](../../../../C:%5CDocuments%20and%20Settings%5CAdministrator%5CDesktop%5CDystro2009_all%20-Project%5COutput%5CGOClassComparison%20FSHD%20inf%20vs%20fshd%20filtered%5CGeneSetGenesTable1.html#GO:0031981) | 0.38482 | 0.54323 | < 0.005 (+) | 0.000096 |
| 83 | GO:0022857 | MF | transmembrane transporter activity | [6](../../../../C:%5CDocuments%20and%20Settings%5CAdministrator%5CDesktop%5CDystro2009_all%20-Project%5COutput%5CGOClassComparison%20FSHD%20inf%20vs%20fshd%20filtered%5CGeneSetGenesTable1.html#GO:0022857) | 0.38741 | 0.74944 | 0.13 (-) | 0.0042243 |
| 84 | GO:0022891 | MF | substrate-specific transmembrane transporter activity | [6](../../../../C:%5CDocuments%20and%20Settings%5CAdministrator%5CDesktop%5CDystro2009_all%20-Project%5COutput%5CGOClassComparison%20FSHD%20inf%20vs%20fshd%20filtered%5CGeneSetGenesTable1.html#GO:0022891) | 0.38741 | 0.74944 | 0.13 (-) | 0.0042243 |
| 85 | GO:0044428 | CC | nuclear part | [24](../../../../C:%5CDocuments%20and%20Settings%5CAdministrator%5CDesktop%5CDystro2009_all%20-Project%5COutput%5CGOClassComparison%20FSHD%20inf%20vs%20fshd%20filtered%5CGeneSetGenesTable1.html#GO:0044428) | 0.3882 | 0.56149 | 0.005 (+) | 0.0000675 |
| 86 | GO:0032553 | MF | ribonucleotide binding | [26](../../../../C:%5CDocuments%20and%20Settings%5CAdministrator%5CDesktop%5CDystro2009_all%20-Project%5COutput%5CGOClassComparison%20FSHD%20inf%20vs%20fshd%20filtered%5CGeneSetGenesTable1.html#GO:0032553) | 0.39178 | 0.66214 | 0.335 (+) | 0.0000977 |
| 87 | GO:0032555 | MF | purine ribonucleotide binding | [26](../../../../C:%5CDocuments%20and%20Settings%5CAdministrator%5CDesktop%5CDystro2009_all%20-Project%5COutput%5CGOClassComparison%20FSHD%20inf%20vs%20fshd%20filtered%5CGeneSetGenesTable1.html#GO:0032555) | 0.39178 | 0.66214 | 0.335 (+) | 0.0000977 |
| 88 | GO:0044431 | CC | Golgi apparatus part | [5](../../../../C:%5CDocuments%20and%20Settings%5CAdministrator%5CDesktop%5CDystro2009_all%20-Project%5COutput%5CGOClassComparison%20FSHD%20inf%20vs%20fshd%20filtered%5CGeneSetGenesTable1.html#GO:0044431) | 0.39817 | 0.42018 | 0.185 (-) | 0.0041772 |
| 89 | GO:0040011 | BP | locomotion | [10](../../../../C:%5CDocuments%20and%20Settings%5CAdministrator%5CDesktop%5CDystro2009_all%20-Project%5COutput%5CGOClassComparison%20FSHD%20inf%20vs%20fshd%20filtered%5CGeneSetGenesTable1.html#GO:0040011) | 0.40813 | 0.32308 | 0.185 (-) | 0.0052077 |
| 90 | GO:0006952 | BP | defense response | [37](../../../../C:%5CDocuments%20and%20Settings%5CAdministrator%5CDesktop%5CDystro2009_all%20-Project%5COutput%5CGOClassComparison%20FSHD%20inf%20vs%20fshd%20filtered%5CGeneSetGenesTable1.html#GO:0006952) | 0.41169 | 0.48758 | 0.075 (-) | 0.0005599 |
| 91 | GO:0007610 | BP | behavior | [11](../../../../C:%5CDocuments%20and%20Settings%5CAdministrator%5CDesktop%5CDystro2009_all%20-Project%5COutput%5CGOClassComparison%20FSHD%20inf%20vs%20fshd%20filtered%5CGeneSetGenesTable1.html#GO:0007610) | 0.41284 | 0.16735 | 0.16 (-) | 0.0049741 |
| 92 | GO:0048731 | BP | system development | [18](../../../../C:%5CDocuments%20and%20Settings%5CAdministrator%5CDesktop%5CDystro2009_all%20-Project%5COutput%5CGOClassComparison%20FSHD%20inf%20vs%20fshd%20filtered%5CGeneSetGenesTable1.html#GO:0048731) | 0.43802 | 0.19536 | 0.33 (-) | 0.0019091 |
| 93 | GO:0051704 | BP | multi-organism process | [31](../../../../C:%5CDocuments%20and%20Settings%5CAdministrator%5CDesktop%5CDystro2009_all%20-Project%5COutput%5CGOClassComparison%20FSHD%20inf%20vs%20fshd%20filtered%5CGeneSetGenesTable1.html#GO:0051704) | 0.43832 | 0.48361 | 0.1 (-) | 0.0013438 |
| 94 | GO:0009607 | BP | response to biotic stimulus | [22](../../../../C:%5CDocuments%20and%20Settings%5CAdministrator%5CDesktop%5CDystro2009_all%20-Project%5COutput%5CGOClassComparison%20FSHD%20inf%20vs%20fshd%20filtered%5CGeneSetGenesTable1.html#GO:0009607) | 0.43865 | 0.47232 | 0.14 (-) | 0.000353 |
| 95 | GO:0051336 | BP | regulation of hydrolase activity | [7](../../../../C:%5CDocuments%20and%20Settings%5CAdministrator%5CDesktop%5CDystro2009_all%20-Project%5COutput%5CGOClassComparison%20FSHD%20inf%20vs%20fshd%20filtered%5CGeneSetGenesTable1.html#GO:0051336) | 0.45698 | 0.89612 | 0.42 (-) | 0.0001675 |
| 96 | GO:0008202 | BP | steroid metabolic process | [8](../../../../C:%5CDocuments%20and%20Settings%5CAdministrator%5CDesktop%5CDystro2009_all%20-Project%5COutput%5CGOClassComparison%20FSHD%20inf%20vs%20fshd%20filtered%5CGeneSetGenesTable1.html#GO:0008202) | 0.46488 | 0.57527 | 0.19 (-) | 0.0068017 |
| 97 | GO:0000166 | MF | nucleotide binding | [36](../../../../C:%5CDocuments%20and%20Settings%5CAdministrator%5CDesktop%5CDystro2009_all%20-Project%5COutput%5CGOClassComparison%20FSHD%20inf%20vs%20fshd%20filtered%5CGeneSetGenesTable1.html#GO:0000166) | 0.47349 | 0.61391 | 0.065 (+) | 0.0000994 |
| 98 | GO:0048856 | BP | anatomical structure development | [20](../../../../C:%5CDocuments%20and%20Settings%5CAdministrator%5CDesktop%5CDystro2009_all%20-Project%5COutput%5CGOClassComparison%20FSHD%20inf%20vs%20fshd%20filtered%5CGeneSetGenesTable1.html#GO:0048856) | 0.49176 | 0.19966 | 0.295 (-) | 0.0019196 |
| 99 | GO:0006508 | BP | proteolysis | [14](../../../../C:%5CDocuments%20and%20Settings%5CAdministrator%5CDesktop%5CDystro2009_all%20-Project%5COutput%5CGOClassComparison%20FSHD%20inf%20vs%20fshd%20filtered%5CGeneSetGenesTable1.html#GO:0006508) | 0.49214 | 0.7276 | 0.115 (+) | 0.0000042 |
| 100 | GO:0006259 | BP | DNA metabolic process | [10](../../../../C:%5CDocuments%20and%20Settings%5CAdministrator%5CDesktop%5CDystro2009_all%20-Project%5COutput%5CGOClassComparison%20FSHD%20inf%20vs%20fshd%20filtered%5CGeneSetGenesTable1.html#GO:0006259) | 0.49852 | 0.30215 | 0.07 (+) | 0.0012629 |
| 101 | GO:0030246 | MF | carbohydrate binding | [23](../../../../C:%5CDocuments%20and%20Settings%5CAdministrator%5CDesktop%5CDystro2009_all%20-Project%5COutput%5CGOClassComparison%20FSHD%20inf%20vs%20fshd%20filtered%5CGeneSetGenesTable2.html#GO:0030246) | 0.50952 | 0.7132 | 0.175 (+) | 0.0030997 |
| 102 | GO:0006631 | BP | fatty acid metabolic process | [7](../../../../C:%5CDocuments%20and%20Settings%5CAdministrator%5CDesktop%5CDystro2009_all%20-Project%5COutput%5CGOClassComparison%20FSHD%20inf%20vs%20fshd%20filtered%5CGeneSetGenesTable2.html#GO:0006631) | 0.51042 | 0.81222 | 0.2 (-) | 0.0055623 |
| 103 | GO:0032787 | BP | monocarboxylic acid metabolic process | [7](../../../../C:%5CDocuments%20and%20Settings%5CAdministrator%5CDesktop%5CDystro2009_all%20-Project%5COutput%5CGOClassComparison%20FSHD%20inf%20vs%20fshd%20filtered%5CGeneSetGenesTable2.html#GO:0032787) | 0.51042 | 0.81222 | 0.2 (-) | 0.0055623 |
| 104 | GO:0051707 | BP | response to other organism | [20](../../../../C:%5CDocuments%20and%20Settings%5CAdministrator%5CDesktop%5CDystro2009_all%20-Project%5COutput%5CGOClassComparison%20FSHD%20inf%20vs%20fshd%20filtered%5CGeneSetGenesTable2.html#GO:0051707) | 0.52536 | 0.39876 | 0.18 (-) | 0.0008624 |
| 105 | GO:0015630 | CC | microtubule cytoskeleton | [6](../../../../C:%5CDocuments%20and%20Settings%5CAdministrator%5CDesktop%5CDystro2009_all%20-Project%5COutput%5CGOClassComparison%20FSHD%20inf%20vs%20fshd%20filtered%5CGeneSetGenesTable2.html#GO:0015630) | 0.55262 | 0.87301 | 0.44 (-) | 0.001807 |
| 106 | GO:0031974 | CC | membrane-enclosed lumen | [26](../../../../C:%5CDocuments%20and%20Settings%5CAdministrator%5CDesktop%5CDystro2009_all%20-Project%5COutput%5CGOClassComparison%20FSHD%20inf%20vs%20fshd%20filtered%5CGeneSetGenesTable2.html#GO:0031974) | 0.55351 | 0.54977 | < 0.005 (+) | 0.0001923 |
| 107 | GO:0043233 | CC | organelle lumen | [26](../../../../C:%5CDocuments%20and%20Settings%5CAdministrator%5CDesktop%5CDystro2009_all%20-Project%5COutput%5CGOClassComparison%20FSHD%20inf%20vs%20fshd%20filtered%5CGeneSetGenesTable2.html#GO:0043233) | 0.55351 | 0.54977 | < 0.005 (+) | 0.0001923 |
| 108 | GO:0070013 | CC | intracellular organelle lumen | [26](../../../../C:%5CDocuments%20and%20Settings%5CAdministrator%5CDesktop%5CDystro2009_all%20-Project%5COutput%5CGOClassComparison%20FSHD%20inf%20vs%20fshd%20filtered%5CGeneSetGenesTable2.html#GO:0070013) | 0.55351 | 0.54977 | < 0.005 (+) | 0.0001923 |
| 109 | GO:0005624 | CC | membrane fraction | [26](../../../../C:%5CDocuments%20and%20Settings%5CAdministrator%5CDesktop%5CDystro2009_all%20-Project%5COutput%5CGOClassComparison%20FSHD%20inf%20vs%20fshd%20filtered%5CGeneSetGenesTable2.html#GO:0005624) | 0.56138 | 0.74722 | 0.36 (-) | 0.0019351 |
| 110 | GO:0015075 | MF | ion transmembrane transporter activity | [5](../../../../C:%5CDocuments%20and%20Settings%5CAdministrator%5CDesktop%5CDystro2009_all%20-Project%5COutput%5CGOClassComparison%20FSHD%20inf%20vs%20fshd%20filtered%5CGeneSetGenesTable2.html#GO:0015075) | 0.5619 | 0.90605 | 0.325 (-) | 0.0011226 |
| 111 | GO:0005626 | CC | insoluble fraction | [27](../../../../C:%5CDocuments%20and%20Settings%5CAdministrator%5CDesktop%5CDystro2009_all%20-Project%5COutput%5CGOClassComparison%20FSHD%20inf%20vs%20fshd%20filtered%5CGeneSetGenesTable2.html#GO:0005626) | 0.58817 | 0.79878 | 0.34 (-) | 0.0019028 |
| 112 | GO:0005794 | CC | Golgi apparatus | [10](../../../../C:%5CDocuments%20and%20Settings%5CAdministrator%5CDesktop%5CDystro2009_all%20-Project%5COutput%5CGOClassComparison%20FSHD%20inf%20vs%20fshd%20filtered%5CGeneSetGenesTable2.html#GO:0005794) | 0.59785 | 0.51235 | 0.215 (-) | 0.0036795 |
| 113 | GO:0006082 | BP | organic acid metabolic process | [8](../../../../C:%5CDocuments%20and%20Settings%5CAdministrator%5CDesktop%5CDystro2009_all%20-Project%5COutput%5CGOClassComparison%20FSHD%20inf%20vs%20fshd%20filtered%5CGeneSetGenesTable2.html#GO:0006082) | 0.60169 | 0.81614 | 0.275 (-) | 0.0055453 |
| 114 | GO:0019752 | BP | carboxylic acid metabolic process | [8](../../../../C:%5CDocuments%20and%20Settings%5CAdministrator%5CDesktop%5CDystro2009_all%20-Project%5COutput%5CGOClassComparison%20FSHD%20inf%20vs%20fshd%20filtered%5CGeneSetGenesTable2.html#GO:0019752) | 0.60169 | 0.81614 | 0.275 (-) | 0.0055453 |
| 115 | GO:0050790 | BP | regulation of catalytic activity | [10](../../../../C:%5CDocuments%20and%20Settings%5CAdministrator%5CDesktop%5CDystro2009_all%20-Project%5COutput%5CGOClassComparison%20FSHD%20inf%20vs%20fshd%20filtered%5CGeneSetGenesTable2.html#GO:0050790) | 0.60445 | 0.87629 | 0.38 (-) | 0.0002102 |
| 116 | GO:0065009 | BP | regulation of molecular function | [10](../../../../C:%5CDocuments%20and%20Settings%5CAdministrator%5CDesktop%5CDystro2009_all%20-Project%5COutput%5CGOClassComparison%20FSHD%20inf%20vs%20fshd%20filtered%5CGeneSetGenesTable2.html#GO:0065009) | 0.60445 | 0.87629 | 0.38 (-) | 0.0002102 |
| 117 | GO:0016740 | MF | transferase activity | [24](../../../../C:%5CDocuments%20and%20Settings%5CAdministrator%5CDesktop%5CDystro2009_all%20-Project%5COutput%5CGOClassComparison%20FSHD%20inf%20vs%20fshd%20filtered%5CGeneSetGenesTable2.html#GO:0016740) | 0.61484 | 0.91048 | 0.08 (+) | 0.0000107 |
| 118 | GO:0000267 | CC | cell fraction | [30](../../../../C:%5CDocuments%20and%20Settings%5CAdministrator%5CDesktop%5CDystro2009_all%20-Project%5COutput%5CGOClassComparison%20FSHD%20inf%20vs%20fshd%20filtered%5CGeneSetGenesTable2.html#GO:0000267) | 0.61598 | 0.80636 | 0.435 (-) | 0.0016168 |
| 119 | GO:0016491 | MF | oxidoreductase activity | [23](../../../../C:%5CDocuments%20and%20Settings%5CAdministrator%5CDesktop%5CDystro2009_all%20-Project%5COutput%5CGOClassComparison%20FSHD%20inf%20vs%20fshd%20filtered%5CGeneSetGenesTable2.html#GO:0016491) | 0.63685 | 0.83348 | 0.34 (-) | 0.0042776 |
| 120 | GO:0007242 | BP | intracellular signaling cascade | [22](../../../../C:%5CDocuments%20and%20Settings%5CAdministrator%5CDesktop%5CDystro2009_all%20-Project%5COutput%5CGOClassComparison%20FSHD%20inf%20vs%20fshd%20filtered%5CGeneSetGenesTable2.html#GO:0007242) | 0.63841 | 0.81325 | 0.41 (-) | 0.000078 |
| 121 | GO:0044444 | CC | cytoplasmic part | [72](../../../../C:%5CDocuments%20and%20Settings%5CAdministrator%5CDesktop%5CDystro2009_all%20-Project%5COutput%5CGOClassComparison%20FSHD%20inf%20vs%20fshd%20filtered%5CGeneSetGenesTable2.html#GO:0044444) | 0.64197 | 0.52759 | 0.255 (-) | 0.0085563 |
| 122 | GO:0030154 | BP | cell differentiation | [16](../../../../C:%5CDocuments%20and%20Settings%5CAdministrator%5CDesktop%5CDystro2009_all%20-Project%5COutput%5CGOClassComparison%20FSHD%20inf%20vs%20fshd%20filtered%5CGeneSetGenesTable2.html#GO:0030154) | 0.64257 | 0.81765 | 0.485 (+) | 0.0031694 |
| 123 | GO:0006366 | BP | transcription from RNA polymerase II promoter | [12](../../../../C:%5CDocuments%20and%20Settings%5CAdministrator%5CDesktop%5CDystro2009_all%20-Project%5COutput%5CGOClassComparison%20FSHD%20inf%20vs%20fshd%20filtered%5CGeneSetGenesTable2.html#GO:0006366) | 0.65043 | 0.86381 | 0.145 (+) | 0.0066626 |
| 124 | GO:0044430 | CC | cytoskeletal part | [11](../../../../C:%5CDocuments%20and%20Settings%5CAdministrator%5CDesktop%5CDystro2009_all%20-Project%5COutput%5CGOClassComparison%20FSHD%20inf%20vs%20fshd%20filtered%5CGeneSetGenesTable2.html#GO:0044430) | 0.66615 | 0.81608 | 0.495 (-) | 0.0053271 |
| 125 | GO:0006996 | BP | organelle organization | [14](../../../../C:%5CDocuments%20and%20Settings%5CAdministrator%5CDesktop%5CDystro2009_all%20-Project%5COutput%5CGOClassComparison%20FSHD%20inf%20vs%20fshd%20filtered%5CGeneSetGenesTable2.html#GO:0006996) | 0.68913 | 0.901 | 0.145 (+) | 0.0005884 |
| 126 | GO:0004888 | MF | transmembrane receptor activity | [69](../../../../C:%5CDocuments%20and%20Settings%5CAdministrator%5CDesktop%5CDystro2009_all%20-Project%5COutput%5CGOClassComparison%20FSHD%20inf%20vs%20fshd%20filtered%5CGeneSetGenesTable2.html#GO:0004888) | 0.69586 | 0.44353 | 0.215 (-) | 0.0061954 |
| 127 | GO:0005125 | MF | cytokine activity | [15](../../../../C:%5CDocuments%20and%20Settings%5CAdministrator%5CDesktop%5CDystro2009_all%20-Project%5COutput%5CGOClassComparison%20FSHD%20inf%20vs%20fshd%20filtered%5CGeneSetGenesTable2.html#GO:0005125) | 0.71206 | 0.66734 | 0.41 (-) | 0.0055347 |
| 128 | GO:0048869 | BP | cellular developmental process | [17](../../../../C:%5CDocuments%20and%20Settings%5CAdministrator%5CDesktop%5CDystro2009_all%20-Project%5COutput%5CGOClassComparison%20FSHD%20inf%20vs%20fshd%20filtered%5CGeneSetGenesTable2.html#GO:0048869) | 0.72304 | 0.89358 | 0.41 (-) | 0.0031784 |
| 129 | GO:0044255 | BP | cellular lipid metabolic process | [15](../../../../C:%5CDocuments%20and%20Settings%5CAdministrator%5CDesktop%5CDystro2009_all%20-Project%5COutput%5CGOClassComparison%20FSHD%20inf%20vs%20fshd%20filtered%5CGeneSetGenesTable2.html#GO:0044255) | 0.73298 | 0.89922 | 0.325 (-) | 0.0065818 |
| 130 | GO:0030528 | MF | transcription regulator activity | [59](../../../../C:%5CDocuments%20and%20Settings%5CAdministrator%5CDesktop%5CDystro2009_all%20-Project%5COutput%5CGOClassComparison%20FSHD%20inf%20vs%20fshd%20filtered%5CGeneSetGenesTable2.html#GO:0030528) | 0.74474 | 0.35886 | 0.035 (+) | 0.0002427 |
| 131 | GO:0005856 | CC | cytoskeleton | [21](../../../../C:%5CDocuments%20and%20Settings%5CAdministrator%5CDesktop%5CDystro2009_all%20-Project%5COutput%5CGOClassComparison%20FSHD%20inf%20vs%20fshd%20filtered%5CGeneSetGenesTable2.html#GO:0005856) | 0.74777 | 0.85346 | 0.425 (+) | 0.001654 |
| 132 | GO:0005102 | MF | receptor binding | [20](../../../../C:%5CDocuments%20and%20Settings%5CAdministrator%5CDesktop%5CDystro2009_all%20-Project%5COutput%5CGOClassComparison%20FSHD%20inf%20vs%20fshd%20filtered%5CGeneSetGenesTable2.html#GO:0005102) | 0.75049 | 0.49955 | 0.44 (-) | 0.0061304 |
| 133 | GO:0004175 | MF | endopeptidase activity | [7](../../../../C:%5CDocuments%20and%20Settings%5CAdministrator%5CDesktop%5CDystro2009_all%20-Project%5COutput%5CGOClassComparison%20FSHD%20inf%20vs%20fshd%20filtered%5CGeneSetGenesTable2.html#GO:0004175) | 0.75999 | 0.8286 | 0.475 (-) | 0.0008654 |
| 134 | GO:0022892 | MF | substrate-specific transporter activity | [10](../../../../C:%5CDocuments%20and%20Settings%5CAdministrator%5CDesktop%5CDystro2009_all%20-Project%5COutput%5CGOClassComparison%20FSHD%20inf%20vs%20fshd%20filtered%5CGeneSetGenesTable2.html#GO:0022892) | 0.77646 | 0.91684 | 0.395 (-) | 0.009663 |
| 135 | GO:0005529 | MF | sugar binding | [16](../../../../C:%5CDocuments%20and%20Settings%5CAdministrator%5CDesktop%5CDystro2009_all%20-Project%5COutput%5CGOClassComparison%20FSHD%20inf%20vs%20fshd%20filtered%5CGeneSetGenesTable2.html#GO:0005529) | 0.77938 | 0.88537 | 0.33 (+) | 0.0015793 |
| 136 | GO:0042221 | BP | response to chemical stimulus | [23](../../../../C:%5CDocuments%20and%20Settings%5CAdministrator%5CDesktop%5CDystro2009_all%20-Project%5COutput%5CGOClassComparison%20FSHD%20inf%20vs%20fshd%20filtered%5CGeneSetGenesTable2.html#GO:0042221) | 0.80948 | 0.82898 | 0.275 (-) | 0.0029224 |
| 137 | GO:0044421 | CC | extracellular region part | [28](../../../../C:%5CDocuments%20and%20Settings%5CAdministrator%5CDesktop%5CDystro2009_all%20-Project%5COutput%5CGOClassComparison%20FSHD%20inf%20vs%20fshd%20filtered%5CGeneSetGenesTable2.html#GO:0044421) | 0.81145 | 0.86651 | 0.415 (-) | 0.0045292 |
| 138 | GO:0006810 | BP | transport | [27](../../../../C:%5CDocuments%20and%20Settings%5CAdministrator%5CDesktop%5CDystro2009_all%20-Project%5COutput%5CGOClassComparison%20FSHD%20inf%20vs%20fshd%20filtered%5CGeneSetGenesTable2.html#GO:0006810) | 0.84104 | 0.91974 | 0.325 (-) | 0.0030147 |
| 139 | GO:0051234 | BP | establishment of localization | [27](../../../../C:%5CDocuments%20and%20Settings%5CAdministrator%5CDesktop%5CDystro2009_all%20-Project%5COutput%5CGOClassComparison%20FSHD%20inf%20vs%20fshd%20filtered%5CGeneSetGenesTable2.html#GO:0051234) | 0.84104 | 0.91974 | 0.325 (-) | 0.0030147 |
| 140 | GO:0006629 | BP | lipid metabolic process | [24](../../../../C:%5CDocuments%20and%20Settings%5CAdministrator%5CDesktop%5CDystro2009_all%20-Project%5COutput%5CGOClassComparison%20FSHD%20inf%20vs%20fshd%20filtered%5CGeneSetGenesTable2.html#GO:0006629) | 0.84279 | 0.91496 | 0.185 (-) | 0.0009177 |
| 141 | GO:0004871 | MF | signal transducer activity | [94](../../../../C:%5CDocuments%20and%20Settings%5CAdministrator%5CDesktop%5CDystro2009_all%20-Project%5COutput%5CGOClassComparison%20FSHD%20inf%20vs%20fshd%20filtered%5CGeneSetGenesTable2.html#GO:0004871) | 0.84307 | 0.73487 | 0.2 (-) | 0.0048845 |
| 142 | GO:0060089 | MF | molecular transducer activity | [94](../../../../C:%5CDocuments%20and%20Settings%5CAdministrator%5CDesktop%5CDystro2009_all%20-Project%5COutput%5CGOClassComparison%20FSHD%20inf%20vs%20fshd%20filtered%5CGeneSetGenesTable2.html#GO:0060089) | 0.84307 | 0.73487 | 0.2 (-) | 0.0048845 |
| 143 | GO:0003824 | MF | catalytic activity | [84](../../../../C:%5CDocuments%20and%20Settings%5CAdministrator%5CDesktop%5CDystro2009_all%20-Project%5COutput%5CGOClassComparison%20FSHD%20inf%20vs%20fshd%20filtered%5CGeneSetGenesTable2.html#GO:0003824) | 0.84888 | 0.91772 | 0.015 (+) | 0.0002393 |
| 144 | GO:0004872 | MF | receptor activity | [90](../../../../C:%5CDocuments%20and%20Settings%5CAdministrator%5CDesktop%5CDystro2009_all%20-Project%5COutput%5CGOClassComparison%20FSHD%20inf%20vs%20fshd%20filtered%5CGeneSetGenesTable2.html#GO:0004872) | 0.86496 | 0.66581 | 0.42 (-) | 0.0058027 |
| 145 | GO:0016043 | BP | cellular component organization | [23](../../../../C:%5CDocuments%20and%20Settings%5CAdministrator%5CDesktop%5CDystro2009_all%20-Project%5COutput%5CGOClassComparison%20FSHD%20inf%20vs%20fshd%20filtered%5CGeneSetGenesTable2.html#GO:0016043) | 0.86926 | 0.92116 | 0.29 (+) | 0.0004958 |
| 146 | GO:0051179 | BP | localization | [31](../../../../C:%5CDocuments%20and%20Settings%5CAdministrator%5CDesktop%5CDystro2009_all%20-Project%5COutput%5CGOClassComparison%20FSHD%20inf%20vs%20fshd%20filtered%5CGeneSetGenesTable2.html#GO:0051179) | 0.87086 | 0.91402 | 0.31 (-) | 0.0051319 |
| 147 | GO:0030234 | MF | enzyme regulator activity | [12](../../../../C:%5CDocuments%20and%20Settings%5CAdministrator%5CDesktop%5CDystro2009_all%20-Project%5COutput%5CGOClassComparison%20FSHD%20inf%20vs%20fshd%20filtered%5CGeneSetGenesTable2.html#GO:0030234) | 0.8972 | 0.91666 | 0.29 (-) | 0.0045547 |
| 148 | GO:0012505 | CC | endomembrane system | [33](../../../../C:%5CDocuments%20and%20Settings%5CAdministrator%5CDesktop%5CDystro2009_all%20-Project%5COutput%5CGOClassComparison%20FSHD%20inf%20vs%20fshd%20filtered%5CGeneSetGenesTable2.html#GO:0012505) | 0.91472 | 0.91001 | 0.12 (+) | 0.0045437 |
| 149 | GO:0005576 | CC | extracellular region | [84](../../../../C:%5CDocuments%20and%20Settings%5CAdministrator%5CDesktop%5CDystro2009_all%20-Project%5COutput%5CGOClassComparison%20FSHD%20inf%20vs%20fshd%20filtered%5CGeneSetGenesTable2.html#GO:0005576) | 0.91494 | 0.90943 | 0.51 (-) | 0.00033 |
| 150 | GO:0016787 | MF | hydrolase activity | [29](../../../../C:%5CDocuments%20and%20Settings%5CAdministrator%5CDesktop%5CDystro2009_all%20-Project%5COutput%5CGOClassComparison%20FSHD%20inf%20vs%20fshd%20filtered%5CGeneSetGenesTable2.html#GO:0016787) | 0.91884 | 0.90804 | 0.385 (+) | 0.0012933 |
| 151 | GO:0008233 | MF | peptidase activity | [10](../../../../C:%5CDocuments%20and%20Settings%5CAdministrator%5CDesktop%5CDystro2009_all%20-Project%5COutput%5CGOClassComparison%20FSHD%20inf%20vs%20fshd%20filtered%5CGeneSetGenesTable2.html#GO:0008233) | 0.91943 | 0.9186 | 0.355 (+) | 0.0044943 |
| 152 | GO:0070011 | MF | peptidase activity, acting on L-amino acid peptides | [10](../../../../C:%5CDocuments%20and%20Settings%5CAdministrator%5CDesktop%5CDystro2009_all%20-Project%5COutput%5CGOClassComparison%20FSHD%20inf%20vs%20fshd%20filtered%5CGeneSetGenesTable2.html#GO:0070011) | 0.91943 | 0.9186 | 0.355 (+) | 0.0044943 |
| 153 | GO:0007165 | BP | signal transduction | [91](../../../../C:%5CDocuments%20and%20Settings%5CAdministrator%5CDesktop%5CDystro2009_all%20-Project%5COutput%5CGOClassComparison%20FSHD%20inf%20vs%20fshd%20filtered%5CGeneSetGenesTable2.html#GO:0007165) | 0.91953 | 0.90836 | 0.445 (-) | 0.0011656 |
| 154 | GO:0007154 | BP | cell communication | [94](../../../../C:%5CDocuments%20and%20Settings%5CAdministrator%5CDesktop%5CDystro2009_all%20-Project%5COutput%5CGOClassComparison%20FSHD%20inf%20vs%20fshd%20filtered%5CGeneSetGenesTable2.html#GO:0007154) | 0.92275 | 0.90921 | 0.48 (+) | 0.0010849 |
| 155 | GO:0031090 | CC | organelle membrane | [37](../../../../C:%5CDocuments%20and%20Settings%5CAdministrator%5CDesktop%5CDystro2009_all%20-Project%5COutput%5CGOClassComparison%20FSHD%20inf%20vs%20fshd%20filtered%5CGeneSetGenesTable2.html#GO:0031090) | 0.92935 | 0.92017 | 0.075 (+) | 0.0058045 |
| 156 | GO:0007166 | BP | cell surface receptor linked signal transduction | [58](../../../../C:%5CDocuments%20and%20Settings%5CAdministrator%5CDesktop%5CDystro2009_all%20-Project%5COutput%5CGOClassComparison%20FSHD%20inf%20vs%20fshd%20filtered%5CGeneSetGenesTable2.html#GO:0007166) | 0.94036 | 0.88348 | 0.44 (+) | 0.000632 |

| KEGG Gene Set Expression ComparisonFSHD T2-STIR + vs. FSHD T2-STIR - | | | | | | | |
| --- | --- | --- | --- | --- | --- | --- | --- |
|  | **Kegg Pathway** | **Pathway description** | **Number of genes** | **LS permutation p-value** | **KS permutation p-value** | **Efron-Tibshirani's GSA test p-value** | **Goeman's global test p-value** |
| 1 | hsa00010 | [Glycolysis / Gluconeogenesis](http://cgap.nci.nih.gov/Pathways/Kegg/hsa00010) | [50](../../../../C:%5CDocuments%20and%20Settings%5CAdministrator%5CDesktop%5CDystro2009_all%20-Project%5COutput%5CFshdnf%20vs%20fshd_PathwayClassComparison_filtered%20KEGG%5CGeneSetGenesTable1.html#hsa00010) | 0.00001 | 0.00004 | 0.01 (+) | 0.000278 |
| 2 | hsa00020 | [Citrate cycle (TCA cycle)](http://cgap.nci.nih.gov/Pathways/Kegg/hsa00020) | [24](../../../../C:%5CDocuments%20and%20Settings%5CAdministrator%5CDesktop%5CDystro2009_all%20-Project%5COutput%5CFshdnf%20vs%20fshd_PathwayClassComparison_filtered%20KEGG%5CGeneSetGenesTable1.html#hsa00020) | 0.00001 | 0.00005 | < 0.005 (+) | 0.0000236 |
| 3 | hsa00190 | [Oxidative phosphorylation](http://cgap.nci.nih.gov/Pathways/Kegg/hsa00190) | [99](../../../../C:%5CDocuments%20and%20Settings%5CAdministrator%5CDesktop%5CDystro2009_all%20-Project%5COutput%5CFshdnf%20vs%20fshd_PathwayClassComparison_filtered%20KEGG%5CGeneSetGenesTable1.html#hsa00190) | 0.00001 | 0.00001 | < 0.005 (+) | 0.000214 |
| 4 | hsa01032 | [Glycan structures - degradation](http://cgap.nci.nih.gov/Pathways/Kegg/hsa01032) | [22](../../../../C:%5CDocuments%20and%20Settings%5CAdministrator%5CDesktop%5CDystro2009_all%20-Project%5COutput%5CFshdnf%20vs%20fshd_PathwayClassComparison_filtered%20KEGG%5CGeneSetGenesTable1.html#hsa01032) | 0.00001 | 0.01637 | < 0.005 (-) | 0.0000135 |
| 5 | hsa04610 | [Complement and coagulation cascades](http://cgap.nci.nih.gov/Pathways/Kegg/hsa04610) | [53](../../../../C:%5CDocuments%20and%20Settings%5CAdministrator%5CDesktop%5CDystro2009_all%20-Project%5COutput%5CFshdnf%20vs%20fshd_PathwayClassComparison_filtered%20KEGG%5CGeneSetGenesTable1.html#hsa04610) | 0.00001 | 0.00021 | < 0.005 (-) | 0.0000132 |
| 6 | hsa04670 | [Leukocyte transendothelial migration](http://cgap.nci.nih.gov/Pathways/Kegg/hsa04670) | [87](../../../../C:%5CDocuments%20and%20Settings%5CAdministrator%5CDesktop%5CDystro2009_all%20-Project%5COutput%5CFshdnf%20vs%20fshd_PathwayClassComparison_filtered%20KEGG%5CGeneSetGenesTable1.html#hsa04670) | 0.00001 | 0.00002 | < 0.005 (-) | 0.000002 |
| 7 | hsa04510 | [Focal adhesion](http://cgap.nci.nih.gov/Pathways/Kegg/hsa04510) | [174](../../../../C:%5CDocuments%20and%20Settings%5CAdministrator%5CDesktop%5CDystro2009_all%20-Project%5COutput%5CFshdnf%20vs%20fshd_PathwayClassComparison_filtered%20KEGG%5CGeneSetGenesTable1.html#hsa04510) | 0.00011 | 0.00596 | 0.07 (-) | 0.0004106 |
| 8 | hsa00531 | [Glycosaminoglycan degradation](http://cgap.nci.nih.gov/Pathways/Kegg/hsa00531) | [13](../../../../C:%5CDocuments%20and%20Settings%5CAdministrator%5CDesktop%5CDystro2009_all%20-Project%5COutput%5CFshdnf%20vs%20fshd_PathwayClassComparison_filtered%20KEGG%5CGeneSetGenesTable1.html#hsa00531) | 0.00018 | 0.06034 | < 0.005 (-) | 0.0000346 |
| 9 | hsa00130 | [Ubiquinone biosynthesis](http://cgap.nci.nih.gov/Pathways/Kegg/hsa00130) | [8](../../../../C:%5CDocuments%20and%20Settings%5CAdministrator%5CDesktop%5CDystro2009_all%20-Project%5COutput%5CFshdnf%20vs%20fshd_PathwayClassComparison_filtered%20KEGG%5CGeneSetGenesTable1.html#hsa00130) | 0.00022 | 0.00001 | < 0.005 (+) | 0.0000263 |
| 10 | hsa04650 | [Natural killer cell mediated cytotoxicity](http://cgap.nci.nih.gov/Pathways/Kegg/hsa04650) | [94](../../../../C:%5CDocuments%20and%20Settings%5CAdministrator%5CDesktop%5CDystro2009_all%20-Project%5COutput%5CFshdnf%20vs%20fshd_PathwayClassComparison_filtered%20KEGG%5CGeneSetGenesTable1.html#hsa04650) | 0.00024 | 0.00256 | 0.05 (-) | 0.0002659 |
| 11 | hsa04514 | [Cell adhesion molecules (CAMs)](http://cgap.nci.nih.gov/Pathways/Kegg/hsa04514) | [102](../../../../C:%5CDocuments%20and%20Settings%5CAdministrator%5CDesktop%5CDystro2009_all%20-Project%5COutput%5CFshdnf%20vs%20fshd_PathwayClassComparison_filtered%20KEGG%5CGeneSetGenesTable1.html#hsa04514) | 0.00028 | 0.00055 | 0.1 (-) | 0.0010746 |
| 12 | hsa00252 | [Alanine and aspartate metabolism](http://cgap.nci.nih.gov/Pathways/Kegg/hsa00252) | [24](../../../../C:%5CDocuments%20and%20Settings%5CAdministrator%5CDesktop%5CDystro2009_all%20-Project%5COutput%5CFshdnf%20vs%20fshd_PathwayClassComparison_filtered%20KEGG%5CGeneSetGenesTable1.html#hsa00252) | 0.00039 | 0.00029 | 0.01 (+) | 0.000143 |
| 13 | hsa00760 | [Nicotinate and nicotinamide metabolism](http://cgap.nci.nih.gov/Pathways/Kegg/hsa00760) | [40](../../../../C:%5CDocuments%20and%20Settings%5CAdministrator%5CDesktop%5CDystro2009_all%20-Project%5COutput%5CFshdnf%20vs%20fshd_PathwayClassComparison_filtered%20KEGG%5CGeneSetGenesTable1.html#hsa00760) | 0.00042 | 0.00016 | < 0.005 (+) | 0.0000235 |
| 14 | hsa04512 | [ECM-receptor interaction](http://cgap.nci.nih.gov/Pathways/Kegg/hsa04512) | [71](../../../../C:%5CDocuments%20and%20Settings%5CAdministrator%5CDesktop%5CDystro2009_all%20-Project%5COutput%5CFshdnf%20vs%20fshd_PathwayClassComparison_filtered%20KEGG%5CGeneSetGenesTable1.html#hsa04512) | 0.0005 | 0.00686 | 0.07 (-) | 0.0013389 |
| 15 | hsa04620 | [Toll-like receptor signaling pathway](http://cgap.nci.nih.gov/Pathways/Kegg/hsa04620) | [71](../../../../C:%5CDocuments%20and%20Settings%5CAdministrator%5CDesktop%5CDystro2009_all%20-Project%5COutput%5CFshdnf%20vs%20fshd_PathwayClassComparison_filtered%20KEGG%5CGeneSetGenesTable1.html#hsa04620) | 0.00051 | 0.00106 | 0.1 (-) | 0.0003535 |
| 16 | hsa00193 | [ATP synthesis](http://cgap.nci.nih.gov/Pathways/Kegg/hsa00193) | [35](../../../../C:%5CDocuments%20and%20Settings%5CAdministrator%5CDesktop%5CDystro2009_all%20-Project%5COutput%5CFshdnf%20vs%20fshd_PathwayClassComparison_filtered%20KEGG%5CGeneSetGenesTable1.html#hsa00193) | 0.00055 | 0.0044 | 0.01 (+) | 0.0000734 |
| 17 | hsa00530 | [Aminosugars metabolism](http://cgap.nci.nih.gov/Pathways/Kegg/hsa00530) | [26](../../../../C:%5CDocuments%20and%20Settings%5CAdministrator%5CDesktop%5CDystro2009_all%20-Project%5COutput%5CFshdnf%20vs%20fshd_PathwayClassComparison_filtered%20KEGG%5CGeneSetGenesTable1.html#hsa00530) | 0.0006 | 0.00008 | 0.005 (-) | 0.0000133 |
| 18 | hsa04810 | [Regulation of actin cytoskeleton](http://cgap.nci.nih.gov/Pathways/Kegg/hsa04810) | [168](../../../../C:%5CDocuments%20and%20Settings%5CAdministrator%5CDesktop%5CDystro2009_all%20-Project%5COutput%5CFshdnf%20vs%20fshd_PathwayClassComparison_filtered%20KEGG%5CGeneSetGenesTable1.html#hsa04810) | 0.00094 | 0.09359 | 0.07 (-) | 0.0000022 |
| 19 | hsa04530 | [Tight junction](http://cgap.nci.nih.gov/Pathways/Kegg/hsa04530) | [94](../../../../C:%5CDocuments%20and%20Settings%5CAdministrator%5CDesktop%5CDystro2009_all%20-Project%5COutput%5CFshdnf%20vs%20fshd_PathwayClassComparison_filtered%20KEGG%5CGeneSetGenesTable1.html#hsa04530) | 0.00109 | 0.05666 | 0.035 (-) | 0.000037 |
| 20 | hsa00511 | [N-Glycan degradation](http://cgap.nci.nih.gov/Pathways/Kegg/hsa00511) | [9](../../../../C:%5CDocuments%20and%20Settings%5CAdministrator%5CDesktop%5CDystro2009_all%20-Project%5COutput%5CFshdnf%20vs%20fshd_PathwayClassComparison_filtered%20KEGG%5CGeneSetGenesTable1.html#hsa00511) | 0.0014 | 0.01491 | < 0.005 (-) | 0.0000335 |
| 21 | hsa04640 | [Hematopoietic cell lineage](http://cgap.nci.nih.gov/Pathways/Kegg/hsa04640) | [63](../../../../C:%5CDocuments%20and%20Settings%5CAdministrator%5CDesktop%5CDystro2009_all%20-Project%5COutput%5CFshdnf%20vs%20fshd_PathwayClassComparison_filtered%20KEGG%5CGeneSetGenesTable1.html#hsa04640) | 0.00194 | 0.00082 | 0.125 (-) | 0.0003818 |
| 22 | hsa00900 | [Terpenoid biosynthesis](http://cgap.nci.nih.gov/Pathways/Kegg/hsa00900) | [6](../../../../C:%5CDocuments%20and%20Settings%5CAdministrator%5CDesktop%5CDystro2009_all%20-Project%5COutput%5CFshdnf%20vs%20fshd_PathwayClassComparison_filtered%20KEGG%5CGeneSetGenesTable1.html#hsa00900) | 0.00218 | 0.01254 | < 0.005 (+) | 0.0018777 |
| 23 | hsa00330 | [Arginine and proline metabolism](http://cgap.nci.nih.gov/Pathways/Kegg/hsa00330) | [44](../../../../C:%5CDocuments%20and%20Settings%5CAdministrator%5CDesktop%5CDystro2009_all%20-Project%5COutput%5CFshdnf%20vs%20fshd_PathwayClassComparison_filtered%20KEGG%5CGeneSetGenesTable1.html#hsa00330) | 0.00248 | 0.03442 | 0.06 (+) | 0.0001255 |
| 24 | hsa01430 | [Cell Communication](http://cgap.nci.nih.gov/Pathways/Kegg/hsa01430) | [80](../../../../C:%5CDocuments%20and%20Settings%5CAdministrator%5CDesktop%5CDystro2009_all%20-Project%5COutput%5CFshdnf%20vs%20fshd_PathwayClassComparison_filtered%20KEGG%5CGeneSetGenesTable1.html#hsa01430) | 0.00266 | 0.06153 | 0.045 (-) | 0.0013601 |
| 25 | hsa00030 | [Pentose phosphate pathway](http://cgap.nci.nih.gov/Pathways/Kegg/hsa00030) | [23](../../../../C:%5CDocuments%20and%20Settings%5CAdministrator%5CDesktop%5CDystro2009_all%20-Project%5COutput%5CFshdnf%20vs%20fshd_PathwayClassComparison_filtered%20KEGG%5CGeneSetGenesTable1.html#hsa00030) | 0.00292 | 0.13477 | 0.215 (-) | 0.0004529 |
| 26 | hsa04612 | [Antigen processing and presentation](http://cgap.nci.nih.gov/Pathways/Kegg/hsa04612) | [61](../../../../C:%5CDocuments%20and%20Settings%5CAdministrator%5CDesktop%5CDystro2009_all%20-Project%5COutput%5CFshdnf%20vs%20fshd_PathwayClassComparison_filtered%20KEGG%5CGeneSetGenesTable1.html#hsa04612) | 0.00319 | 0.00179 | 0.215 (-) | 0.0052561 |
| 27 | hsa00720 | [Reductive carboxylate cycle (CO2 fixation)](http://cgap.nci.nih.gov/Pathways/Kegg/hsa00720) | [8](../../../../C:%5CDocuments%20and%20Settings%5CAdministrator%5CDesktop%5CDystro2009_all%20-Project%5COutput%5CFshdnf%20vs%20fshd_PathwayClassComparison_filtered%20KEGG%5CGeneSetGenesTable1.html#hsa00720) | 0.00349 | 0.00051 | < 0.005 (+) | 0.0002507 |
| 28 | hsa04664 | [Fc epsilon RI signaling pathway](http://cgap.nci.nih.gov/Pathways/Kegg/hsa04664) | [59](../../../../C:%5CDocuments%20and%20Settings%5CAdministrator%5CDesktop%5CDystro2009_all%20-Project%5COutput%5CFshdnf%20vs%20fshd_PathwayClassComparison_filtered%20KEGG%5CGeneSetGenesTable1.html#hsa04664) | 0.00368 | 0.0759 | 0.095 (-) | 0.0000249 |
| 29 | hsa04110 | [Cell cycle](http://cgap.nci.nih.gov/Pathways/Kegg/hsa04110) | [103](../../../../C:%5CDocuments%20and%20Settings%5CAdministrator%5CDesktop%5CDystro2009_all%20-Project%5COutput%5CFshdnf%20vs%20fshd_PathwayClassComparison_filtered%20KEGG%5CGeneSetGenesTable1.html#hsa04110) | 0.00753 | 0.01365 | 0.295 (-) | 0.0000028 |
| 30 | hsa04940 | [Type I diabetes mellitus](http://cgap.nci.nih.gov/Pathways/Kegg/hsa04940) | [34](../../../../C:%5CDocuments%20and%20Settings%5CAdministrator%5CDesktop%5CDystro2009_all%20-Project%5COutput%5CFshdnf%20vs%20fshd_PathwayClassComparison_filtered%20KEGG%5CGeneSetGenesTable1.html#hsa04940) | 0.00823 | 0.01196 | 0.185 (-) | 0.0055757 |
| 31 | hsa04910 | [Insulin signaling pathway](http://cgap.nci.nih.gov/Pathways/Kegg/hsa04910) | [116](../../../../C:%5CDocuments%20and%20Settings%5CAdministrator%5CDesktop%5CDystro2009_all%20-Project%5COutput%5CFshdnf%20vs%20fshd_PathwayClassComparison_filtered%20KEGG%5CGeneSetGenesTable1.html#hsa04910) | 0.00886 | 0.01561 | 0.115 (+) | 0.0002425 |
| 32 | hsa04662 | [B cell receptor signaling pathway](http://cgap.nci.nih.gov/Pathways/Kegg/hsa04662) | [56](../../../../C:%5CDocuments%20and%20Settings%5CAdministrator%5CDesktop%5CDystro2009_all%20-Project%5COutput%5CFshdnf%20vs%20fshd_PathwayClassComparison_filtered%20KEGG%5CGeneSetGenesTable1.html#hsa04662) | 0.0093 | 0.00508 | 0.1 (-) | 0.0000571 |
| 33 | hsa04020 | [Calcium signaling pathway](http://cgap.nci.nih.gov/Pathways/Kegg/hsa04020) | [133](../../../../C:%5CDocuments%20and%20Settings%5CAdministrator%5CDesktop%5CDystro2009_all%20-Project%5COutput%5CFshdnf%20vs%20fshd_PathwayClassComparison_filtered%20KEGG%5CGeneSetGenesTable1.html#hsa04020) | 0.00959 | 0.04167 | 0.01 (+) | 0.0000186 |
| 34 | hsa00051 | [Fructose and mannose metabolism](http://cgap.nci.nih.gov/Pathways/Kegg/hsa00051) | [41](../../../../C:%5CDocuments%20and%20Settings%5CAdministrator%5CDesktop%5CDystro2009_all%20-Project%5COutput%5CFshdnf%20vs%20fshd_PathwayClassComparison_filtered%20KEGG%5CGeneSetGenesTable1.html#hsa00051) | 0.01035 | 0.0077 | < 0.005 (+) | 0.0002736 |
| 35 | hsa00071 | [Fatty acid metabolism](http://cgap.nci.nih.gov/Pathways/Kegg/hsa00071) | [46](../../../../C:%5CDocuments%20and%20Settings%5CAdministrator%5CDesktop%5CDystro2009_all%20-Project%5COutput%5CFshdnf%20vs%20fshd_PathwayClassComparison_filtered%20KEGG%5CGeneSetGenesTable1.html#hsa00071) | 0.01279 | 0.01819 | 0.105 (+) | 0.0000265 |
| 36 | hsa04920 | [Adipocytokine signaling pathway](http://cgap.nci.nih.gov/Pathways/Kegg/hsa04920) | [59](../../../../C:%5CDocuments%20and%20Settings%5CAdministrator%5CDesktop%5CDystro2009_all%20-Project%5COutput%5CFshdnf%20vs%20fshd_PathwayClassComparison_filtered%20KEGG%5CGeneSetGenesTable1.html#hsa04920) | 0.01319 | 0.00001 | 0.415 (+) | 0.0003006 |
| 37 | hsa05120 | [Epithelial cell signaling in Helicobacter pylori infection](http://cgap.nci.nih.gov/Pathways/Kegg/hsa05120) | [59](../../../../C:%5CDocuments%20and%20Settings%5CAdministrator%5CDesktop%5CDystro2009_all%20-Project%5COutput%5CFshdnf%20vs%20fshd_PathwayClassComparison_filtered%20KEGG%5CGeneSetGenesTable1.html#hsa05120) | 0.01384 | 0.06194 | 0.185 (-) | 0.000022 |
| 38 | hsa00620 | [Pyruvate metabolism](http://cgap.nci.nih.gov/Pathways/Kegg/hsa00620) | [34](../../../../C:%5CDocuments%20and%20Settings%5CAdministrator%5CDesktop%5CDystro2009_all%20-Project%5COutput%5CFshdnf%20vs%20fshd_PathwayClassComparison_filtered%20KEGG%5CGeneSetGenesTable1.html#hsa00620) | 0.01403 | 0.0346 | 0.075 (+) | 0.000102 |
| 39 | hsa04540 | [Gap junction](http://cgap.nci.nih.gov/Pathways/Kegg/hsa04540) | [81](../../../../C:%5CDocuments%20and%20Settings%5CAdministrator%5CDesktop%5CDystro2009_all%20-Project%5COutput%5CFshdnf%20vs%20fshd_PathwayClassComparison_filtered%20KEGG%5CGeneSetGenesTable1.html#hsa04540) | 0.01493 | 0.20759 | 0.11 (-) | 0.0001018 |
| 40 | hsa00400 | [Phenylalanine, tyrosine and tryptophan biosynthesis](http://cgap.nci.nih.gov/Pathways/Kegg/hsa00400) | [11](../../../../C:%5CDocuments%20and%20Settings%5CAdministrator%5CDesktop%5CDystro2009_all%20-Project%5COutput%5CFshdnf%20vs%20fshd_PathwayClassComparison_filtered%20KEGG%5CGeneSetGenesTable1.html#hsa00400) | 0.01494 | 0.07123 | 0.1 (+) | 0.0001735 |
| 41 | hsa00052 | [Galactose metabolism](http://cgap.nci.nih.gov/Pathways/Kegg/hsa00052) | [21](../../../../C:%5CDocuments%20and%20Settings%5CAdministrator%5CDesktop%5CDystro2009_all%20-Project%5COutput%5CFshdnf%20vs%20fshd_PathwayClassComparison_filtered%20KEGG%5CGeneSetGenesTable1.html#hsa00052) | 0.01563 | 0.00092 | 0.065 (+) | 0.0000892 |
| 42 | hsa00640 | [Propanoate metabolism](http://cgap.nci.nih.gov/Pathways/Kegg/hsa00640) | [33](../../../../C:%5CDocuments%20and%20Settings%5CAdministrator%5CDesktop%5CDystro2009_all%20-Project%5COutput%5CFshdnf%20vs%20fshd_PathwayClassComparison_filtered%20KEGG%5CGeneSetGenesTable1.html#hsa00640) | 0.01647 | 0.10502 | 0.04 (+) | 0.0000749 |
| 43 | hsa00750 | [Vitamin B6 metabolism](http://cgap.nci.nih.gov/Pathways/Kegg/hsa00750) | [8](../../../../C:%5CDocuments%20and%20Settings%5CAdministrator%5CDesktop%5CDystro2009_all%20-Project%5COutput%5CFshdnf%20vs%20fshd_PathwayClassComparison_filtered%20KEGG%5CGeneSetGenesTable1.html#hsa00750) | 0.01832 | 0.02327 | 0.015 (+) | 0.0003438 |
| 44 | hsa04010 | [MAPK signaling pathway](http://cgap.nci.nih.gov/Pathways/Kegg/hsa04010) | [222](../../../../C:%5CDocuments%20and%20Settings%5CAdministrator%5CDesktop%5CDystro2009_all%20-Project%5COutput%5CFshdnf%20vs%20fshd_PathwayClassComparison_filtered%20KEGG%5CGeneSetGenesTable1.html#hsa04010) | 0.01887 | 0.0165 | 0.44 (+) | 0.0000002 |
| 45 | hsa00561 | [Glycerolipid metabolism](http://cgap.nci.nih.gov/Pathways/Kegg/hsa00561) | [45](../../../../C:%5CDocuments%20and%20Settings%5CAdministrator%5CDesktop%5CDystro2009_all%20-Project%5COutput%5CFshdnf%20vs%20fshd_PathwayClassComparison_filtered%20KEGG%5CGeneSetGenesTable1.html#hsa00561) | 0.02049 | 0.13728 | 0.075 (-) | 0.0005276 |
| 46 | hsa00120 | [Bile acid biosynthesis](http://cgap.nci.nih.gov/Pathways/Kegg/hsa00120) | [34](../../../../C:%5CDocuments%20and%20Settings%5CAdministrator%5CDesktop%5CDystro2009_all%20-Project%5COutput%5CFshdnf%20vs%20fshd_PathwayClassComparison_filtered%20KEGG%5CGeneSetGenesTable1.html#hsa00120) | 0.02096 | 0.04212 | 0.13 (-) | 0.0000977 |
| 47 | hsa00600 | [Sphingolipid metabolism](http://cgap.nci.nih.gov/Pathways/Kegg/hsa00600) | [26](../../../../C:%5CDocuments%20and%20Settings%5CAdministrator%5CDesktop%5CDystro2009_all%20-Project%5COutput%5CFshdnf%20vs%20fshd_PathwayClassComparison_filtered%20KEGG%5CGeneSetGenesTable1.html#hsa00600) | 0.0243 | 0.08342 | 0.05 (-) | 0.0003275 |
| 48 | hsa00520 | [Nucleotide sugars metabolism](http://cgap.nci.nih.gov/Pathways/Kegg/hsa00520) | [15](../../../../C:%5CDocuments%20and%20Settings%5CAdministrator%5CDesktop%5CDystro2009_all%20-Project%5COutput%5CFshdnf%20vs%20fshd_PathwayClassComparison_filtered%20KEGG%5CGeneSetGenesTable1.html#hsa00520) | 0.02437 | 0.05626 | 0.11 (-) | 0.0000587 |
| 49 | hsa04120 | [Ubiquitin mediated proteolysis](http://cgap.nci.nih.gov/Pathways/Kegg/hsa04120) | [40](../../../../C:%5CDocuments%20and%20Settings%5CAdministrator%5CDesktop%5CDystro2009_all%20-Project%5COutput%5CFshdnf%20vs%20fshd_PathwayClassComparison_filtered%20KEGG%5CGeneSetGenesTable1.html#hsa04120) | 0.02525 | 0.00957 | 0.03 (+) | 0.0000299 |
| 50 | hsa03320 | [PPAR signaling pathway](http://cgap.nci.nih.gov/Pathways/Kegg/hsa03320) | [56](../../../../C:%5CDocuments%20and%20Settings%5CAdministrator%5CDesktop%5CDystro2009_all%20-Project%5COutput%5CFshdnf%20vs%20fshd_PathwayClassComparison_filtered%20KEGG%5CGeneSetGenesTable1.html#hsa03320) | 0.02587 | 0.064 | 0.375 (+) | 0.0000702 |
| 51 | hsa01510 | [Neurodegenerative Disorders](http://cgap.nci.nih.gov/Pathways/Kegg/hsa01510) | [30](../../../../C:%5CDocuments%20and%20Settings%5CAdministrator%5CDesktop%5CDystro2009_all%20-Project%5COutput%5CFshdnf%20vs%20fshd_PathwayClassComparison_filtered%20KEGG%5CGeneSetGenesTable1.html#hsa01510) | 0.02649 | 0.02306 | 0.175 (-) | 0.0000363 |
| 52 | hsa04210 | [Apoptosis](http://cgap.nci.nih.gov/Pathways/Kegg/hsa04210) | [74](../../../../C:%5CDocuments%20and%20Settings%5CAdministrator%5CDesktop%5CDystro2009_all%20-Project%5COutput%5CFshdnf%20vs%20fshd_PathwayClassComparison_filtered%20KEGG%5CGeneSetGenesTable1.html#hsa04210) | 0.02823 | 0.09089 | 0.465 (-) | 0.0000098 |
| 53 | hsa03050 | [Proteasome](http://cgap.nci.nih.gov/Pathways/Kegg/hsa03050) | [30](../../../../C:%5CDocuments%20and%20Settings%5CAdministrator%5CDesktop%5CDystro2009_all%20-Project%5COutput%5CFshdnf%20vs%20fshd_PathwayClassComparison_filtered%20KEGG%5CGeneSetGenesTable1.html#hsa03050) | 0.02913 | 0.00933 | 0.045 (+) | 0.0012204 |
| 54 | hsa00603 | [Glycosphingolipid biosynthesis - globoseries](http://cgap.nci.nih.gov/Pathways/Kegg/hsa00603) | [10](../../../../C:%5CDocuments%20and%20Settings%5CAdministrator%5CDesktop%5CDystro2009_all%20-Project%5COutput%5CFshdnf%20vs%20fshd_PathwayClassComparison_filtered%20KEGG%5CGeneSetGenesTable1.html#hsa00603) | 0.02999 | 0.18945 | 0.055 (-) | 0.0002204 |
| 55 | hsa00770 | [Pantothenate and CoA biosynthesis](http://cgap.nci.nih.gov/Pathways/Kegg/hsa00770) | [20](../../../../C:%5CDocuments%20and%20Settings%5CAdministrator%5CDesktop%5CDystro2009_all%20-Project%5COutput%5CFshdnf%20vs%20fshd_PathwayClassComparison_filtered%20KEGG%5CGeneSetGenesTable1.html#hsa00770) | 0.03093 | 0.03719 | 0.145 (+) | 0.0031236 |
| 56 | hsa00100 | [Biosynthesis of steroids](http://cgap.nci.nih.gov/Pathways/Kegg/hsa00100) | [16](../../../../C:%5CDocuments%20and%20Settings%5CAdministrator%5CDesktop%5CDystro2009_all%20-Project%5COutput%5CFshdnf%20vs%20fshd_PathwayClassComparison_filtered%20KEGG%5CGeneSetGenesTable1.html#hsa00100) | 0.03314 | 0.1126 | 0.02 (+) | 0.0006394 |
| 57 | hsa00230 | [Purine metabolism](http://cgap.nci.nih.gov/Pathways/Kegg/hsa00230) | [124](../../../../C:%5CDocuments%20and%20Settings%5CAdministrator%5CDesktop%5CDystro2009_all%20-Project%5COutput%5CFshdnf%20vs%20fshd_PathwayClassComparison_filtered%20KEGG%5CGeneSetGenesTable1.html#hsa00230) | 0.03553 | 0.00531 | 0.17 (+) | 0.0015936 |
| 58 | hsa05130 | [NA](http://cgap.nci.nih.gov/Pathways/Kegg/hsa05130) | [25](../../../../C:%5CDocuments%20and%20Settings%5CAdministrator%5CDesktop%5CDystro2009_all%20-Project%5COutput%5CFshdnf%20vs%20fshd_PathwayClassComparison_filtered%20KEGG%5CGeneSetGenesTable1.html#hsa05130) | 0.03982 | 0.01956 | 0.075 (-) | 0.0001084 |
| 59 | hsa05131 | [NA](http://cgap.nci.nih.gov/Pathways/Kegg/hsa05131) | [25](../../../../C:%5CDocuments%20and%20Settings%5CAdministrator%5CDesktop%5CDystro2009_all%20-Project%5COutput%5CFshdnf%20vs%20fshd_PathwayClassComparison_filtered%20KEGG%5CGeneSetGenesTable1.html#hsa05131) | 0.03982 | 0.01956 | 0.075 (-) | 0.0001084 |
| 60 | hsa00251 | [Glutamate metabolism](http://cgap.nci.nih.gov/Pathways/Kegg/hsa00251) | [26](../../../../C:%5CDocuments%20and%20Settings%5CAdministrator%5CDesktop%5CDystro2009_all%20-Project%5COutput%5CFshdnf%20vs%20fshd_PathwayClassComparison_filtered%20KEGG%5CGeneSetGenesTable1.html#hsa00251) | 0.04411 | 0.00423 | 0.15 (+) | 0.0012586 |
| 61 | hsa04630 | [Jak-STAT signaling pathway](http://cgap.nci.nih.gov/Pathways/Kegg/hsa04630) | [111](../../../../C:%5CDocuments%20and%20Settings%5CAdministrator%5CDesktop%5CDystro2009_all%20-Project%5COutput%5CFshdnf%20vs%20fshd_PathwayClassComparison_filtered%20KEGG%5CGeneSetGenesTable1.html#hsa04630) | 0.04511 | 0.02235 | 0.105 (-) | 0.0000165 |
| 62 | hsa00350 | [Tyrosine metabolism](http://cgap.nci.nih.gov/Pathways/Kegg/hsa00350) | [44](../../../../C:%5CDocuments%20and%20Settings%5CAdministrator%5CDesktop%5CDystro2009_all%20-Project%5COutput%5CFshdnf%20vs%20fshd_PathwayClassComparison_filtered%20KEGG%5CGeneSetGenesTable1.html#hsa00350) | 0.04759 | 0.0802 | 0.1 (+) | 0.0002275 |
| 63 | hsa00062 | [Fatty acid elongation in mitochondria](http://cgap.nci.nih.gov/Pathways/Kegg/hsa00062) | [9](../../../../C:%5CDocuments%20and%20Settings%5CAdministrator%5CDesktop%5CDystro2009_all%20-Project%5COutput%5CFshdnf%20vs%20fshd_PathwayClassComparison_filtered%20KEGG%5CGeneSetGenesTable1.html#hsa00062) | 0.0523 | 0.18151 | 0.1 (+) | 0.0017464 |
| 64 | hsa00740 | [Riboflavin metabolism](http://cgap.nci.nih.gov/Pathways/Kegg/hsa00740) | [14](../../../../C:%5CDocuments%20and%20Settings%5CAdministrator%5CDesktop%5CDystro2009_all%20-Project%5COutput%5CFshdnf%20vs%20fshd_PathwayClassComparison_filtered%20KEGG%5CGeneSetGenesTable1.html#hsa00740) | 0.0533 | 0.05482 | 0.195 (+) | 0.0007075 |
| 65 | hsa00340 | [Histidine metabolism](http://cgap.nci.nih.gov/Pathways/Kegg/hsa00340) | [34](../../../../C:%5CDocuments%20and%20Settings%5CAdministrator%5CDesktop%5CDystro2009_all%20-Project%5COutput%5CFshdnf%20vs%20fshd_PathwayClassComparison_filtered%20KEGG%5CGeneSetGenesTable1.html#hsa00340) | 0.05357 | 0.1817 | 0.3 (+) | 0.0006292 |
| 66 | hsa00280 | [Valine, leucine and isoleucine degradation](http://cgap.nci.nih.gov/Pathways/Kegg/hsa00280) | [45](../../../../C:%5CDocuments%20and%20Settings%5CAdministrator%5CDesktop%5CDystro2009_all%20-Project%5COutput%5CFshdnf%20vs%20fshd_PathwayClassComparison_filtered%20KEGG%5CGeneSetGenesTable1.html#hsa00280) | 0.05366 | 0.0126 | 0.12 (+) | 0.0002874 |
| 67 | hsa00710 | [Carbon fixation](http://cgap.nci.nih.gov/Pathways/Kegg/hsa00710) | [19](../../../../C:%5CDocuments%20and%20Settings%5CAdministrator%5CDesktop%5CDystro2009_all%20-Project%5COutput%5CFshdnf%20vs%20fshd_PathwayClassComparison_filtered%20KEGG%5CGeneSetGenesTable1.html#hsa00710) | 0.05489 | 0.0739 | < 0.005 (+) | 0.0003583 |
| 68 | hsa05060 | [Prion disease](http://cgap.nci.nih.gov/Pathways/Kegg/hsa05060) | [9](../../../../C:%5CDocuments%20and%20Settings%5CAdministrator%5CDesktop%5CDystro2009_all%20-Project%5COutput%5CFshdnf%20vs%20fshd_PathwayClassComparison_filtered%20KEGG%5CGeneSetGenesTable1.html#hsa05060) | 0.05731 | 0.14723 | 0.03 (-) | 0.0013101 |
| 69 | hsa00625 | [Tetrachloroethene degradation](http://cgap.nci.nih.gov/Pathways/Kegg/hsa00625) | [6](../../../../C:%5CDocuments%20and%20Settings%5CAdministrator%5CDesktop%5CDystro2009_all%20-Project%5COutput%5CFshdnf%20vs%20fshd_PathwayClassComparison_filtered%20KEGG%5CGeneSetGenesTable1.html#hsa00625) | 0.05916 | 0.02242 | 0.205 (-) | 0.000734 |
| 70 | hsa00260 | [Glycine, serine and threonine metabolism](http://cgap.nci.nih.gov/Pathways/Kegg/hsa00260) | [33](../../../../C:%5CDocuments%20and%20Settings%5CAdministrator%5CDesktop%5CDystro2009_all%20-Project%5COutput%5CFshdnf%20vs%20fshd_PathwayClassComparison_filtered%20KEGG%5CGeneSetGenesTable1.html#hsa00260) | 0.0595 | 0.14457 | 0.19 (+) | 0.0001163 |
| 71 | hsa04060 | [Cytokine-cytokine receptor interaction](http://cgap.nci.nih.gov/Pathways/Kegg/hsa04060) | [179](../../../../C:%5CDocuments%20and%20Settings%5CAdministrator%5CDesktop%5CDystro2009_all%20-Project%5COutput%5CFshdnf%20vs%20fshd_PathwayClassComparison_filtered%20KEGG%5CGeneSetGenesTable1.html#hsa04060) | 0.06394 | 0.08909 | 0.19 (-) | 0.0002426 |
| 72 | hsa00650 | [Butanoate metabolism](http://cgap.nci.nih.gov/Pathways/Kegg/hsa00650) | [35](../../../../C:%5CDocuments%20and%20Settings%5CAdministrator%5CDesktop%5CDystro2009_all%20-Project%5COutput%5CFshdnf%20vs%20fshd_PathwayClassComparison_filtered%20KEGG%5CGeneSetGenesTable1.html#hsa00650) | 0.06502 | 0.03211 | 0.115 (+) | 0.0004104 |
| 73 | hsa00590 | [Arachidonic acid metabolism](http://cgap.nci.nih.gov/Pathways/Kegg/hsa00590) | [44](../../../../C:%5CDocuments%20and%20Settings%5CAdministrator%5CDesktop%5CDystro2009_all%20-Project%5COutput%5CFshdnf%20vs%20fshd_PathwayClassComparison_filtered%20KEGG%5CGeneSetGenesTable1.html#hsa00590) | 0.06565 | 0.44954 | 0.005 (-) | 0.0000055 |
| 74 | hsa00220 | [Urea cycle and metabolism of amino groups](http://cgap.nci.nih.gov/Pathways/Kegg/hsa00220) | [19](../../../../C:%5CDocuments%20and%20Settings%5CAdministrator%5CDesktop%5CDystro2009_all%20-Project%5COutput%5CFshdnf%20vs%20fshd_PathwayClassComparison_filtered%20KEGG%5CGeneSetGenesTable1.html#hsa00220) | 0.06948 | 0.10582 | 0.355 (+) | 0.0002156 |
| 75 | hsa00380 | [Tryptophan metabolism](http://cgap.nci.nih.gov/Pathways/Kegg/hsa00380) | [74](../../../../C:%5CDocuments%20and%20Settings%5CAdministrator%5CDesktop%5CDystro2009_all%20-Project%5COutput%5CFshdnf%20vs%20fshd_PathwayClassComparison_filtered%20KEGG%5CGeneSetGenesTable1.html#hsa00380) | 0.07145 | 0.01732 | 0.17 (+) | 0.0000226 |
| 76 | hsa00363 | [Bisphenol A degradation](http://cgap.nci.nih.gov/Pathways/Kegg/hsa00363) | [11](../../../../C:%5CDocuments%20and%20Settings%5CAdministrator%5CDesktop%5CDystro2009_all%20-Project%5COutput%5CFshdnf%20vs%20fshd_PathwayClassComparison_filtered%20KEGG%5CGeneSetGenesTable1.html#hsa00363) | 0.07213 | 0.00384 | 0.105 (-) | 0.001261 |
| 77 | hsa00624 | [1- and 2-Methylnaphthalene degradation](http://cgap.nci.nih.gov/Pathways/Kegg/hsa00624) | [20](../../../../C:%5CDocuments%20and%20Settings%5CAdministrator%5CDesktop%5CDystro2009_all%20-Project%5COutput%5CFshdnf%20vs%20fshd_PathwayClassComparison_filtered%20KEGG%5CGeneSetGenesTable1.html#hsa00624) | 0.07566 | 0.03987 | 0.095 (+) | 0.0003934 |
| 78 | hsa00290 | [Valine, leucine and isoleucine biosynthesis](http://cgap.nci.nih.gov/Pathways/Kegg/hsa00290) | [8](../../../../C:%5CDocuments%20and%20Settings%5CAdministrator%5CDesktop%5CDystro2009_all%20-Project%5COutput%5CFshdnf%20vs%20fshd_PathwayClassComparison_filtered%20KEGG%5CGeneSetGenesTable1.html#hsa00290) | 0.07809 | 0.06909 | 0.06 (+) | 0.0006932 |
| 79 | hsa00440 | [Aminophosphonate metabolism](http://cgap.nci.nih.gov/Pathways/Kegg/hsa00440) | [14](../../../../C:%5CDocuments%20and%20Settings%5CAdministrator%5CDesktop%5CDystro2009_all%20-Project%5COutput%5CFshdnf%20vs%20fshd_PathwayClassComparison_filtered%20KEGG%5CGeneSetGenesTable1.html#hsa00440) | 0.08638 | 0.04655 | 0.11 (+) | 0.0002251 |
| 80 | hsa00450 | [Selenoamino acid metabolism](http://cgap.nci.nih.gov/Pathways/Kegg/hsa00450) | [30](../../../../C:%5CDocuments%20and%20Settings%5CAdministrator%5CDesktop%5CDystro2009_all%20-Project%5COutput%5CFshdnf%20vs%20fshd_PathwayClassComparison_filtered%20KEGG%5CGeneSetGenesTable1.html#hsa00450) | 0.09046 | 0.01585 | 0.115 (+) | 0.0004056 |
| 81 | hsa05210 | [Colorectal cancer](http://cgap.nci.nih.gov/Pathways/Kegg/hsa05210) | [68](../../../../C:%5CDocuments%20and%20Settings%5CAdministrator%5CDesktop%5CDystro2009_all%20-Project%5COutput%5CFshdnf%20vs%20fshd_PathwayClassComparison_filtered%20KEGG%5CGeneSetGenesTable1.html#hsa05210) | 0.09924 | 0.0866 | 0.365 (+) | 0.0000033 |
| 82 | hsa00730 | [Thiamine metabolism](http://cgap.nci.nih.gov/Pathways/Kegg/hsa00730) | [6](../../../../C:%5CDocuments%20and%20Settings%5CAdministrator%5CDesktop%5CDystro2009_all%20-Project%5COutput%5CFshdnf%20vs%20fshd_PathwayClassComparison_filtered%20KEGG%5CGeneSetGenesTable1.html#hsa00730) | 0.09991 | 0.07214 | 0.045 (+) | 0.0008411 |
| 83 | hsa05020 | [Parkinson@](http://cgap.nci.nih.gov/Pathways/Kegg/hsa05020) | [9](../../../../C:%5CDocuments%20and%20Settings%5CAdministrator%5CDesktop%5CDystro2009_all%20-Project%5COutput%5CFshdnf%20vs%20fshd_PathwayClassComparison_filtered%20KEGG%5CGeneSetGenesTable1.html#hsa05020) | 0.10085 | 0.03223 | 0.095 (+) | 0.0000176 |
| 84 | hsa05050 | [Dentatorubropallidoluysian atrophy (DRPLA)](http://cgap.nci.nih.gov/Pathways/Kegg/hsa05050) | [12](../../../../C:%5CDocuments%20and%20Settings%5CAdministrator%5CDesktop%5CDystro2009_all%20-Project%5COutput%5CFshdnf%20vs%20fshd_PathwayClassComparison_filtered%20KEGG%5CGeneSetGenesTable1.html#hsa05050) | 0.10947 | 0.40629 | 0.18 (+) | 0.0000259 |
| 85 | hsa05010 | [Alzheimer@](http://cgap.nci.nih.gov/Pathways/Kegg/hsa05010) | [20](../../../../C:%5CDocuments%20and%20Settings%5CAdministrator%5CDesktop%5CDystro2009_all%20-Project%5COutput%5CFshdnf%20vs%20fshd_PathwayClassComparison_filtered%20KEGG%5CGeneSetGenesTable1.html#hsa05010) | 0.12915 | 0.06476 | 0.18 (-) | 0.0002749 |
| 86 | hsa00410 | [beta-Alanine metabolism](http://cgap.nci.nih.gov/Pathways/Kegg/hsa00410) | [21](../../../../C:%5CDocuments%20and%20Settings%5CAdministrator%5CDesktop%5CDystro2009_all%20-Project%5COutput%5CFshdnf%20vs%20fshd_PathwayClassComparison_filtered%20KEGG%5CGeneSetGenesTable1.html#hsa00410) | 0.13315 | 0.39926 | 0.355 (+) | 0.0002124 |
| 87 | hsa00240 | [Pyrimidine metabolism](http://cgap.nci.nih.gov/Pathways/Kegg/hsa00240) | [80](../../../../C:%5CDocuments%20and%20Settings%5CAdministrator%5CDesktop%5CDystro2009_all%20-Project%5COutput%5CFshdnf%20vs%20fshd_PathwayClassComparison_filtered%20KEGG%5CGeneSetGenesTable1.html#hsa00240) | 0.13499 | 0.12555 | 0.24 (+) | 0.0000107 |
| 88 | hsa00604 | [Glycosphingolipid biosynthesis - ganglioseries](http://cgap.nci.nih.gov/Pathways/Kegg/hsa00604) | [13](../../../../C:%5CDocuments%20and%20Settings%5CAdministrator%5CDesktop%5CDystro2009_all%20-Project%5COutput%5CFshdnf%20vs%20fshd_PathwayClassComparison_filtered%20KEGG%5CGeneSetGenesTable1.html#hsa00604) | 0.14528 | 0.41551 | 0.13 (-) | 0.0000359 |
| 89 | hsa00500 | [Starch and sucrose metabolism](http://cgap.nci.nih.gov/Pathways/Kegg/hsa00500) | [55](../../../../C:%5CDocuments%20and%20Settings%5CAdministrator%5CDesktop%5CDystro2009_all%20-Project%5COutput%5CFshdnf%20vs%20fshd_PathwayClassComparison_filtered%20KEGG%5CGeneSetGenesTable1.html#hsa00500) | 0.14932 | 0.33571 | 0.375 (+) | 0.0000467 |
| 90 | hsa00930 | [Caprolactam degradation](http://cgap.nci.nih.gov/Pathways/Kegg/hsa00930) | [16](../../../../C:%5CDocuments%20and%20Settings%5CAdministrator%5CDesktop%5CDystro2009_all%20-Project%5COutput%5CFshdnf%20vs%20fshd_PathwayClassComparison_filtered%20KEGG%5CGeneSetGenesTable1.html#hsa00930) | 0.15013 | 0.28706 | 0.205 (+) | 0.0035911 |
| 91 | hsa00053 | [Ascorbate and aldarate metabolism](http://cgap.nci.nih.gov/Pathways/Kegg/hsa00053) | [11](../../../../C:%5CDocuments%20and%20Settings%5CAdministrator%5CDesktop%5CDystro2009_all%20-Project%5COutput%5CFshdnf%20vs%20fshd_PathwayClassComparison_filtered%20KEGG%5CGeneSetGenesTable1.html#hsa00053) | 0.15519 | 0.23375 | 0.11 (-) | 0.0024579 |
| 92 | hsa00626 | [Nitrobenzene degradation](http://cgap.nci.nih.gov/Pathways/Kegg/hsa00626) | [11](../../../../C:%5CDocuments%20and%20Settings%5CAdministrator%5CDesktop%5CDystro2009_all%20-Project%5COutput%5CFshdnf%20vs%20fshd_PathwayClassComparison_filtered%20KEGG%5CGeneSetGenesTable1.html#hsa00626) | 0.16931 | 0.09197 | 0.11 (+) | 0.0006068 |
| 93 | hsa00632 | [Benzoate degradation via CoA ligation](http://cgap.nci.nih.gov/Pathways/Kegg/hsa00632) | [25](../../../../C:%5CDocuments%20and%20Settings%5CAdministrator%5CDesktop%5CDystro2009_all%20-Project%5COutput%5CFshdnf%20vs%20fshd_PathwayClassComparison_filtered%20KEGG%5CGeneSetGenesTable1.html#hsa00632) | 0.18379 | 0.0756 | 0.06 (+) | 0.008942 |
| 94 | hsa04912 | [GnRH signaling pathway](http://cgap.nci.nih.gov/Pathways/Kegg/hsa04912) | [80](../../../../C:%5CDocuments%20and%20Settings%5CAdministrator%5CDesktop%5CDystro2009_all%20-Project%5COutput%5CFshdnf%20vs%20fshd_PathwayClassComparison_filtered%20KEGG%5CGeneSetGenesTable1.html#hsa04912) | 0.18933 | 0.33308 | 0.285 (+) | 0.0001129 |
| 95 | hsa00040 | [Pentose and glucuronate interconversions](http://cgap.nci.nih.gov/Pathways/Kegg/hsa00040) | [14](../../../../C:%5CDocuments%20and%20Settings%5CAdministrator%5CDesktop%5CDystro2009_all%20-Project%5COutput%5CFshdnf%20vs%20fshd_PathwayClassComparison_filtered%20KEGG%5CGeneSetGenesTable1.html#hsa00040) | 0.19221 | 0.56321 | 0.41 (-) | 0.0001114 |
| 96 | hsa04720 | [Long-term potentiation](http://cgap.nci.nih.gov/Pathways/Kegg/hsa04720) | [56](../../../../C:%5CDocuments%20and%20Settings%5CAdministrator%5CDesktop%5CDystro2009_all%20-Project%5COutput%5CFshdnf%20vs%20fshd_PathwayClassComparison_filtered%20KEGG%5CGeneSetGenesTable1.html#hsa04720) | 0.19269 | 0.13584 | 0.15 (+) | 0.0012555 |
| 97 | hsa05030 | [Amyotrophic lateral sclerosis (ALS)](http://cgap.nci.nih.gov/Pathways/Kegg/hsa05030) | [15](../../../../C:%5CDocuments%20and%20Settings%5CAdministrator%5CDesktop%5CDystro2009_all%20-Project%5COutput%5CFshdnf%20vs%20fshd_PathwayClassComparison_filtered%20KEGG%5CGeneSetGenesTable1.html#hsa05030) | 0.19877 | 0.18964 | 0.495 (-) | 0.0011225 |
| 98 | hsa00521 | [Streptomycin biosynthesis](http://cgap.nci.nih.gov/Pathways/Kegg/hsa00521) | [9](../../../../C:%5CDocuments%20and%20Settings%5CAdministrator%5CDesktop%5CDystro2009_all%20-Project%5COutput%5CFshdnf%20vs%20fshd_PathwayClassComparison_filtered%20KEGG%5CGeneSetGenesTable1.html#hsa00521) | 0.20392 | 0.06993 | 0.04 (+) | 0.0094907 |
| 99 | hsa00310 | [Lysine degradation](http://cgap.nci.nih.gov/Pathways/Kegg/hsa00310) | [45](../../../../C:%5CDocuments%20and%20Settings%5CAdministrator%5CDesktop%5CDystro2009_all%20-Project%5COutput%5CFshdnf%20vs%20fshd_PathwayClassComparison_filtered%20KEGG%5CGeneSetGenesTable1.html#hsa00310) | 0.20679 | 0.19229 | 0.205 (+) | 0.0006212 |
| 100 | hsa04520 | [Adherens junction](http://cgap.nci.nih.gov/Pathways/Kegg/hsa04520) | [69](../../../../C:%5CDocuments%20and%20Settings%5CAdministrator%5CDesktop%5CDystro2009_all%20-Project%5COutput%5CFshdnf%20vs%20fshd_PathwayClassComparison_filtered%20KEGG%5CGeneSetGenesTable1.html#hsa04520) | 0.21176 | 0.14659 | 0.19 (-) | 0.0002212 |
| 101 | hsa00271 | [Methionine metabolism](http://cgap.nci.nih.gov/Pathways/Kegg/hsa00271) | [16](../../../../C:%5CDocuments%20and%20Settings%5CAdministrator%5CDesktop%5CDystro2009_all%20-Project%5COutput%5CFshdnf%20vs%20fshd_PathwayClassComparison_filtered%20KEGG%5CGeneSetGenesTable2.html#hsa00271) | 0.21697 | 0.04381 | 0.345 (+) | 0.0005048 |
| 102 | hsa00601 | [Glycosphingolipid biosynthesis - lactoseries](http://cgap.nci.nih.gov/Pathways/Kegg/hsa00601) | [5](../../../../C:%5CDocuments%20and%20Settings%5CAdministrator%5CDesktop%5CDystro2009_all%20-Project%5COutput%5CFshdnf%20vs%20fshd_PathwayClassComparison_filtered%20KEGG%5CGeneSetGenesTable2.html#hsa00601) | 0.22647 | 0.24097 | 0.04 (+) | 0.0014191 |
| 103 | hsa04330 | [Notch signaling pathway](http://cgap.nci.nih.gov/Pathways/Kegg/hsa04330) | [37](../../../../C:%5CDocuments%20and%20Settings%5CAdministrator%5CDesktop%5CDystro2009_all%20-Project%5COutput%5CFshdnf%20vs%20fshd_PathwayClassComparison_filtered%20KEGG%5CGeneSetGenesTable2.html#hsa04330) | 0.23407 | 0.05874 | 0.36 (-) | 0.00123 |
| 104 | hsa00510 | [N-Glycan biosynthesis](http://cgap.nci.nih.gov/Pathways/Kegg/hsa00510) | [35](../../../../C:%5CDocuments%20and%20Settings%5CAdministrator%5CDesktop%5CDystro2009_all%20-Project%5COutput%5CFshdnf%20vs%20fshd_PathwayClassComparison_filtered%20KEGG%5CGeneSetGenesTable2.html#hsa00510) | 0.23995 | 0.3498 | 0.245 (-) | 0.0000021 |
| 105 | hsa04310 | [Wnt signaling pathway](http://cgap.nci.nih.gov/Pathways/Kegg/hsa04310) | [123](../../../../C:%5CDocuments%20and%20Settings%5CAdministrator%5CDesktop%5CDystro2009_all%20-Project%5COutput%5CFshdnf%20vs%20fshd_PathwayClassComparison_filtered%20KEGG%5CGeneSetGenesTable2.html#hsa04310) | 0.24125 | 0.28266 | 0.475 (+) | 0.000022 |
| 106 | hsa00564 | [Glycerophospholipid metabolism](http://cgap.nci.nih.gov/Pathways/Kegg/hsa00564) | [60](../../../../C:%5CDocuments%20and%20Settings%5CAdministrator%5CDesktop%5CDystro2009_all%20-Project%5COutput%5CFshdnf%20vs%20fshd_PathwayClassComparison_filtered%20KEGG%5CGeneSetGenesTable2.html#hsa00564) | 0.24613 | 0.15678 | 0.345 (+) | 0.0000221 |
| 107 | hsa00460 | [Cyanoamino acid metabolism](http://cgap.nci.nih.gov/Pathways/Kegg/hsa00460) | [7](../../../../C:%5CDocuments%20and%20Settings%5CAdministrator%5CDesktop%5CDystro2009_all%20-Project%5COutput%5CFshdnf%20vs%20fshd_PathwayClassComparison_filtered%20KEGG%5CGeneSetGenesTable2.html#hsa00460) | 0.24801 | 0.47133 | 0.24 (-) | 0.0018375 |
| 108 | hsa00910 | [Nitrogen metabolism](http://cgap.nci.nih.gov/Pathways/Kegg/hsa00910) | [22](../../../../C:%5CDocuments%20and%20Settings%5CAdministrator%5CDesktop%5CDystro2009_all%20-Project%5COutput%5CFshdnf%20vs%20fshd_PathwayClassComparison_filtered%20KEGG%5CGeneSetGenesTable2.html#hsa00910) | 0.25664 | 0.31076 | 0.14 (+) | 0.0020805 |
| 109 | hsa00920 | [Sulfur metabolism](http://cgap.nci.nih.gov/Pathways/Kegg/hsa00920) | [11](../../../../C:%5CDocuments%20and%20Settings%5CAdministrator%5CDesktop%5CDystro2009_all%20-Project%5COutput%5CFshdnf%20vs%20fshd_PathwayClassComparison_filtered%20KEGG%5CGeneSetGenesTable2.html#hsa00920) | 0.26803 | 0.41353 | 0.415 (-) | 0.0040385 |
| 110 | hsa00970 | [Aminoacyl-tRNA biosynthesis](http://cgap.nci.nih.gov/Pathways/Kegg/hsa00970) | [27](../../../../C:%5CDocuments%20and%20Settings%5CAdministrator%5CDesktop%5CDystro2009_all%20-Project%5COutput%5CFshdnf%20vs%20fshd_PathwayClassComparison_filtered%20KEGG%5CGeneSetGenesTable2.html#hsa00970) | 0.27334 | 0.08527 | 0.18 (+) | 0.0044829 |
| 111 | hsa00563 | [Glycosylphosphatidylinositol(GPI)-anchor biosynthesis](http://cgap.nci.nih.gov/Pathways/Kegg/hsa00563) | [22](../../../../C:%5CDocuments%20and%20Settings%5CAdministrator%5CDesktop%5CDystro2009_all%20-Project%5COutput%5CFshdnf%20vs%20fshd_PathwayClassComparison_filtered%20KEGG%5CGeneSetGenesTable2.html#hsa00563) | 0.29198 | 0.08664 | 0.415 (+) | 0.0000531 |
| 112 | hsa00780 | [Biotin metabolism](http://cgap.nci.nih.gov/Pathways/Kegg/hsa00780) | [6](../../../../C:%5CDocuments%20and%20Settings%5CAdministrator%5CDesktop%5CDystro2009_all%20-Project%5COutput%5CFshdnf%20vs%20fshd_PathwayClassComparison_filtered%20KEGG%5CGeneSetGenesTable2.html#hsa00780) | 0.29885 | 0.04307 | 0.2 (+) | 0.0032372 |
| 113 | hsa00980 | [Metabolism of xenobiotics by cytochrome P450](http://cgap.nci.nih.gov/Pathways/Kegg/hsa00980) | [50](../../../../C:%5CDocuments%20and%20Settings%5CAdministrator%5CDesktop%5CDystro2009_all%20-Project%5COutput%5CFshdnf%20vs%20fshd_PathwayClassComparison_filtered%20KEGG%5CGeneSetGenesTable2.html#hsa00980) | 0.30681 | 0.42042 | 0.5 (+) | 0.0005789 |
| 114 | hsa04360 | [Axon guidance](http://cgap.nci.nih.gov/Pathways/Kegg/hsa04360) | [112](../../../../C:%5CDocuments%20and%20Settings%5CAdministrator%5CDesktop%5CDystro2009_all%20-Project%5COutput%5CFshdnf%20vs%20fshd_PathwayClassComparison_filtered%20KEGG%5CGeneSetGenesTable2.html#hsa04360) | 0.31558 | 0.40441 | 0.46 (+) | 0.00005 |
| 115 | hsa00361 | [gamma-Hexachlorocyclohexane degradation](http://cgap.nci.nih.gov/Pathways/Kegg/hsa00361) | [20](../../../../C:%5CDocuments%20and%20Settings%5CAdministrator%5CDesktop%5CDystro2009_all%20-Project%5COutput%5CFshdnf%20vs%20fshd_PathwayClassComparison_filtered%20KEGG%5CGeneSetGenesTable2.html#hsa00361) | 0.34666 | 0.06612 | 0.34 (-) | 0.0004371 |
| 116 | hsa00562 | [Inositol phosphate metabolism](http://cgap.nci.nih.gov/Pathways/Kegg/hsa00562) | [45](../../../../C:%5CDocuments%20and%20Settings%5CAdministrator%5CDesktop%5CDystro2009_all%20-Project%5COutput%5CFshdnf%20vs%20fshd_PathwayClassComparison_filtered%20KEGG%5CGeneSetGenesTable2.html#hsa00562) | 0.36043 | 0.57052 | 0.27 (+) | 0.0000087 |
| 117 | hsa03020 | [RNA polymerase](http://cgap.nci.nih.gov/Pathways/Kegg/hsa03020) | [23](../../../../C:%5CDocuments%20and%20Settings%5CAdministrator%5CDesktop%5CDystro2009_all%20-Project%5COutput%5CFshdnf%20vs%20fshd_PathwayClassComparison_filtered%20KEGG%5CGeneSetGenesTable2.html#hsa03020) | 0.37361 | 0.39321 | 0.155 (+) | 0.000433 |
| 118 | hsa00360 | [Phenylalanine metabolism](http://cgap.nci.nih.gov/Pathways/Kegg/hsa00360) | [21](../../../../C:%5CDocuments%20and%20Settings%5CAdministrator%5CDesktop%5CDystro2009_all%20-Project%5COutput%5CFshdnf%20vs%20fshd_PathwayClassComparison_filtered%20KEGG%5CGeneSetGenesTable2.html#hsa00360) | 0.37645 | 0.73002 | 0.155 (+) | 0.0010025 |
| 119 | hsa04150 | [mTOR signaling pathway](http://cgap.nci.nih.gov/Pathways/Kegg/hsa04150) | [40](../../../../C:%5CDocuments%20and%20Settings%5CAdministrator%5CDesktop%5CDystro2009_all%20-Project%5COutput%5CFshdnf%20vs%20fshd_PathwayClassComparison_filtered%20KEGG%5CGeneSetGenesTable2.html#hsa04150) | 0.37906 | 0.03638 | 0.19 (+) | 0.0006234 |
| 120 | hsa04070 | [Phosphatidylinositol signaling system](http://cgap.nci.nih.gov/Pathways/Kegg/hsa04070) | [64](../../../../C:%5CDocuments%20and%20Settings%5CAdministrator%5CDesktop%5CDystro2009_all%20-Project%5COutput%5CFshdnf%20vs%20fshd_PathwayClassComparison_filtered%20KEGG%5CGeneSetGenesTable2.html#hsa04070) | 0.38274 | 0.47573 | 0.45 (-) | 0.0000071 |
| 121 | hsa05040 | [Huntington@](http://cgap.nci.nih.gov/Pathways/Kegg/hsa05040) | [27](../../../../C:%5CDocuments%20and%20Settings%5CAdministrator%5CDesktop%5CDystro2009_all%20-Project%5COutput%5CFshdnf%20vs%20fshd_PathwayClassComparison_filtered%20KEGG%5CGeneSetGenesTable2.html#hsa05040) | 0.3891 | 0.63816 | 0.505 (+) | 0.0007429 |
| 122 | hsa00960 | [Alkaloid biosynthesis II](http://cgap.nci.nih.gov/Pathways/Kegg/hsa00960) | [14](../../../../C:%5CDocuments%20and%20Settings%5CAdministrator%5CDesktop%5CDystro2009_all%20-Project%5COutput%5CFshdnf%20vs%20fshd_PathwayClassComparison_filtered%20KEGG%5CGeneSetGenesTable2.html#hsa00960) | 0.38946 | 0.59838 | 0.36 (-) | 0.0001417 |
| 123 | hsa00480 | [Glutathione metabolism](http://cgap.nci.nih.gov/Pathways/Kegg/hsa00480) | [36](../../../../C:%5CDocuments%20and%20Settings%5CAdministrator%5CDesktop%5CDystro2009_all%20-Project%5COutput%5CFshdnf%20vs%20fshd_PathwayClassComparison_filtered%20KEGG%5CGeneSetGenesTable2.html#hsa00480) | 0.39424 | 0.20604 | 0.385 (+) | 0.0008594 |
| 124 | hsa04660 | [T cell receptor signaling pathway](http://cgap.nci.nih.gov/Pathways/Kegg/hsa04660) | [77](../../../../C:%5CDocuments%20and%20Settings%5CAdministrator%5CDesktop%5CDystro2009_all%20-Project%5COutput%5CFshdnf%20vs%20fshd_PathwayClassComparison_filtered%20KEGG%5CGeneSetGenesTable2.html#hsa04660) | 0.40208 | 0.19077 | 0.4 (+) | 0.0009543 |
| 125 | hsa00150 | [Androgen and estrogen metabolism](http://cgap.nci.nih.gov/Pathways/Kegg/hsa00150) | [32](../../../../C:%5CDocuments%20and%20Settings%5CAdministrator%5CDesktop%5CDystro2009_all%20-Project%5COutput%5CFshdnf%20vs%20fshd_PathwayClassComparison_filtered%20KEGG%5CGeneSetGenesTable2.html#hsa00150) | 0.40909 | 0.4718 | 0.505 (-) | 0.0005961 |
| 126 | hsa00591 | [Linoleic acid metabolism](http://cgap.nci.nih.gov/Pathways/Kegg/hsa00591) | [26](../../../../C:%5CDocuments%20and%20Settings%5CAdministrator%5CDesktop%5CDystro2009_all%20-Project%5COutput%5CFshdnf%20vs%20fshd_PathwayClassComparison_filtered%20KEGG%5CGeneSetGenesTable2.html#hsa00591) | 0.41366 | 0.61608 | 0.295 (-) | 0.0001387 |
| 127 | hsa00903 | [Limonene and pinene degradation](http://cgap.nci.nih.gov/Pathways/Kegg/hsa00903) | [23](../../../../C:%5CDocuments%20and%20Settings%5CAdministrator%5CDesktop%5CDystro2009_all%20-Project%5COutput%5CFshdnf%20vs%20fshd_PathwayClassComparison_filtered%20KEGG%5CGeneSetGenesTable2.html#hsa00903) | 0.41483 | 0.47866 | 0.335 (+) | 0.0002507 |
| 128 | hsa00642 | [Ethylbenzene degradation](http://cgap.nci.nih.gov/Pathways/Kegg/hsa00642) | [11](../../../../C:%5CDocuments%20and%20Settings%5CAdministrator%5CDesktop%5CDystro2009_all%20-Project%5COutput%5CFshdnf%20vs%20fshd_PathwayClassComparison_filtered%20KEGG%5CGeneSetGenesTable2.html#hsa00642) | 0.44737 | 0.28114 | 0.39 (+) | 0.0003206 |
| 129 | hsa00532 | [Chondroitin sulfate biosynthesis](http://cgap.nci.nih.gov/Pathways/Kegg/hsa00532) | [18](../../../../C:%5CDocuments%20and%20Settings%5CAdministrator%5CDesktop%5CDystro2009_all%20-Project%5COutput%5CFshdnf%20vs%20fshd_PathwayClassComparison_filtered%20KEGG%5CGeneSetGenesTable2.html#hsa00532) | 0.45655 | 0.59926 | 0.315 (-) | 0.0002073 |
| 130 | hsa04930 | [Type II diabetes mellitus](http://cgap.nci.nih.gov/Pathways/Kegg/hsa04930) | [33](../../../../C:%5CDocuments%20and%20Settings%5CAdministrator%5CDesktop%5CDystro2009_all%20-Project%5COutput%5CFshdnf%20vs%20fshd_PathwayClassComparison_filtered%20KEGG%5CGeneSetGenesTable2.html#hsa04930) | 0.45939 | 0.31195 | 0.25 (-) | 0.0038697 |
| 131 | hsa00860 | [Porphyrin and chlorophyll metabolism](http://cgap.nci.nih.gov/Pathways/Kegg/hsa00860) | [23](../../../../C:%5CDocuments%20and%20Settings%5CAdministrator%5CDesktop%5CDystro2009_all%20-Project%5COutput%5CFshdnf%20vs%20fshd_PathwayClassComparison_filtered%20KEGG%5CGeneSetGenesTable2.html#hsa00860) | 0.46468 | 0.74219 | 0.33 (-) | 0.0000745 |
| 132 | hsa01031 | [Glycan structures - biosynthesis 2](http://cgap.nci.nih.gov/Pathways/Kegg/hsa01031) | [54](../../../../C:%5CDocuments%20and%20Settings%5CAdministrator%5CDesktop%5CDystro2009_all%20-Project%5COutput%5CFshdnf%20vs%20fshd_PathwayClassComparison_filtered%20KEGG%5CGeneSetGenesTable2.html#hsa01031) | 0.47738 | 0.27882 | 0.425 (-) | 0.0004623 |
| 133 | hsa04370 | [VEGF signaling pathway](http://cgap.nci.nih.gov/Pathways/Kegg/hsa04370) | [59](../../../../C:%5CDocuments%20and%20Settings%5CAdministrator%5CDesktop%5CDystro2009_all%20-Project%5COutput%5CFshdnf%20vs%20fshd_PathwayClassComparison_filtered%20KEGG%5CGeneSetGenesTable2.html#hsa04370) | 0.4898 | 0.28338 | 0.5 (-) | 0.0000056 |
| 134 | hsa00272 | [Cysteine metabolism](http://cgap.nci.nih.gov/Pathways/Kegg/hsa00272) | [18](../../../../C:%5CDocuments%20and%20Settings%5CAdministrator%5CDesktop%5CDystro2009_all%20-Project%5COutput%5CFshdnf%20vs%20fshd_PathwayClassComparison_filtered%20KEGG%5CGeneSetGenesTable2.html#hsa00272) | 0.49917 | 0.43315 | 0.375 (+) | 0.0004141 |
| 135 | hsa00670 | [One carbon pool by folate](http://cgap.nci.nih.gov/Pathways/Kegg/hsa00670) | [15](../../../../C:%5CDocuments%20and%20Settings%5CAdministrator%5CDesktop%5CDystro2009_all%20-Project%5COutput%5CFshdnf%20vs%20fshd_PathwayClassComparison_filtered%20KEGG%5CGeneSetGenesTable2.html#hsa00670) | 0.50874 | 0.3756 | 0.225 (+) | 0.0025295 |
| 136 | hsa00534 | [Heparan sulfate biosynthesis](http://cgap.nci.nih.gov/Pathways/Kegg/hsa00534) | [17](../../../../C:%5CDocuments%20and%20Settings%5CAdministrator%5CDesktop%5CDystro2009_all%20-Project%5COutput%5CFshdnf%20vs%20fshd_PathwayClassComparison_filtered%20KEGG%5CGeneSetGenesTable2.html#hsa00534) | 0.51948 | 0.25494 | 0.245 (-) | 0.0003239 |
| 137 | hsa04742 | [Taste transduction](http://cgap.nci.nih.gov/Pathways/Kegg/hsa04742) | [31](../../../../C:%5CDocuments%20and%20Settings%5CAdministrator%5CDesktop%5CDystro2009_all%20-Project%5COutput%5CFshdnf%20vs%20fshd_PathwayClassComparison_filtered%20KEGG%5CGeneSetGenesTable2.html#hsa04742) | 0.52585 | 0.78032 | 0.455 (+) | 0.0005584 |
| 138 | hsa00430 | [Taurine and hypotaurine metabolism](http://cgap.nci.nih.gov/Pathways/Kegg/hsa00430) | [8](../../../../C:%5CDocuments%20and%20Settings%5CAdministrator%5CDesktop%5CDystro2009_all%20-Project%5COutput%5CFshdnf%20vs%20fshd_PathwayClassComparison_filtered%20KEGG%5CGeneSetGenesTable2.html#hsa00430) | 0.5273 | 0.74017 | 0.445 (+) | 0.0016007 |
| 139 | hsa03022 | [Basal transcription factors](http://cgap.nci.nih.gov/Pathways/Kegg/hsa03022) | [28](../../../../C:%5CDocuments%20and%20Settings%5CAdministrator%5CDesktop%5CDystro2009_all%20-Project%5COutput%5CFshdnf%20vs%20fshd_PathwayClassComparison_filtered%20KEGG%5CGeneSetGenesTable2.html#hsa03022) | 0.54907 | 0.86153 | 0.21 (+) | 0.0000668 |
| 140 | hsa00940 | [Stilbene, coumarine and lignin biosynthesis](http://cgap.nci.nih.gov/Pathways/Kegg/hsa00940) | [5](../../../../C:%5CDocuments%20and%20Settings%5CAdministrator%5CDesktop%5CDystro2009_all%20-Project%5COutput%5CFshdnf%20vs%20fshd_PathwayClassComparison_filtered%20KEGG%5CGeneSetGenesTable2.html#hsa00940) | 0.57207 | 0.90589 | 0.365 (-) | 0.0009381 |
| 141 | hsa04350 | [TGF-beta signaling pathway](http://cgap.nci.nih.gov/Pathways/Kegg/hsa04350) | [69](../../../../C:%5CDocuments%20and%20Settings%5CAdministrator%5CDesktop%5CDystro2009_all%20-Project%5COutput%5CFshdnf%20vs%20fshd_PathwayClassComparison_filtered%20KEGG%5CGeneSetGenesTable2.html#hsa04350) | 0.58624 | 0.30977 | 0.195 (+) | 0.0018599 |
| 142 | hsa04320 | [Dorso-ventral axis formation](http://cgap.nci.nih.gov/Pathways/Kegg/hsa04320) | [24](../../../../C:%5CDocuments%20and%20Settings%5CAdministrator%5CDesktop%5CDystro2009_all%20-Project%5COutput%5CFshdnf%20vs%20fshd_PathwayClassComparison_filtered%20KEGG%5CGeneSetGenesTable2.html#hsa04320) | 0.59696 | 0.56581 | 0.275 (+) | 0.0013437 |
| 143 | hsa01030 | [Glycan structures - biosynthesis 1](http://cgap.nci.nih.gov/Pathways/Kegg/hsa01030) | [92](../../../../C:%5CDocuments%20and%20Settings%5CAdministrator%5CDesktop%5CDystro2009_all%20-Project%5COutput%5CFshdnf%20vs%20fshd_PathwayClassComparison_filtered%20KEGG%5CGeneSetGenesTable2.html#hsa01030) | 0.63607 | 0.83179 | 0.395 (+) | 0.0000791 |
| 144 | hsa04740 | [Olfactory transduction](http://cgap.nci.nih.gov/Pathways/Kegg/hsa04740) | [23](../../../../C:%5CDocuments%20and%20Settings%5CAdministrator%5CDesktop%5CDystro2009_all%20-Project%5COutput%5CFshdnf%20vs%20fshd_PathwayClassComparison_filtered%20KEGG%5CGeneSetGenesTable2.html#hsa04740) | 0.66222 | 0.85299 | 0.42 (+) | 0.0021962 |
| 145 | hsa04730 | [Long-term depression](http://cgap.nci.nih.gov/Pathways/Kegg/hsa04730) | [60](../../../../C:%5CDocuments%20and%20Settings%5CAdministrator%5CDesktop%5CDystro2009_all%20-Project%5COutput%5CFshdnf%20vs%20fshd_PathwayClassComparison_filtered%20KEGG%5CGeneSetGenesTable2.html#hsa04730) | 0.67174 | 0.85594 | 0.265 (-) | 0.0000295 |
| 146 | hsa04130 | [SNARE interactions in vesicular transport](http://cgap.nci.nih.gov/Pathways/Kegg/hsa04130) | [31](../../../../C:%5CDocuments%20and%20Settings%5CAdministrator%5CDesktop%5CDystro2009_all%20-Project%5COutput%5CFshdnf%20vs%20fshd_PathwayClassComparison_filtered%20KEGG%5CGeneSetGenesTable2.html#hsa04130) | 0.75809 | 0.8204 | 0.36 (+) | 0.0005629 |
| 147 | hsa00533 | [Keratan sulfate biosynthesis](http://cgap.nci.nih.gov/Pathways/Kegg/hsa00533) | [13](../../../../C:%5CDocuments%20and%20Settings%5CAdministrator%5CDesktop%5CDystro2009_all%20-Project%5COutput%5CFshdnf%20vs%20fshd_PathwayClassComparison_filtered%20KEGG%5CGeneSetGenesTable2.html#hsa00533) | 0.8063 | 0.90362 | 0.495 (-) | 0.0042149 |
| 148 | hsa00790 | [Folate biosynthesis](http://cgap.nci.nih.gov/Pathways/Kegg/hsa00790) | [30](../../../../C:%5CDocuments%20and%20Settings%5CAdministrator%5CDesktop%5CDystro2009_all%20-Project%5COutput%5CFshdnf%20vs%20fshd_PathwayClassComparison_filtered%20KEGG%5CGeneSetGenesTable2.html#hsa00790) | 0.80945 | 0.80694 | 0.495 (-) | 0.0000372 |
| 149 | hsa00512 | [O-Glycan biosynthesis](http://cgap.nci.nih.gov/Pathways/Kegg/hsa00512) | [21](../../../../C:%5CDocuments%20and%20Settings%5CAdministrator%5CDesktop%5CDystro2009_all%20-Project%5COutput%5CFshdnf%20vs%20fshd_PathwayClassComparison_filtered%20KEGG%5CGeneSetGenesTable2.html#hsa00512) | 0.81803 | 0.82928 | 0.205 (-) | 0.000756 |
| 150 | hsa04080 | [Neuroactive ligand-receptor interaction](http://cgap.nci.nih.gov/Pathways/Kegg/hsa04080) | [148](../../../../C:%5CDocuments%20and%20Settings%5CAdministrator%5CDesktop%5CDystro2009_all%20-Project%5COutput%5CFshdnf%20vs%20fshd_PathwayClassComparison_filtered%20KEGG%5CGeneSetGenesTable2.html#hsa04080) | 0.9288 | 0.90495 | 0.15 (+) | 0.0025007 |
