# Supplementary material for: Different Molecular Signatures in Magnetic Resonance Imaging-Staged Facioscapulohumeral Muscular Dystrophy Muscles
Source: PLoS One. 2012 Jun 13;7(6):e38779. doi: 10.1371/journal.pone.0038779 (PMC3374833; doi:10.1371/journal.pone.0038779)
Supplement: Table S3 — Class comparison of T2-STIR + FSHD vs. Inflammatory myopathy muscles. (DOC) [file pone.0038779.s007.doc]

**Table S3: Class Comparison FSHD T2-STIR + vs. Inflammatory myopathies**

| **Parametric p-value** | **FDR** | **Geom mean of intensities in FSHD T2-STIR+** | **Geom mean of intensities in IM** | **Fold-change** | **Unique id** | **Gene symbol** | **DefinedGenelist** |
| --- | --- | --- | --- | --- | --- | --- | --- |
| 0.000928 | 0.427352 | 1687.55813 | 173.5021957 | 9.7264367 | 10048 | [CIDEA](http://www.ncbi.nlm.nih.gov/entrez/query.fcgi?cmd=search&db=gene&term=CIDEA) |  |
| 0.0038166 | 0.488743 | 3252.113608 | 379.8174265 | 8.5623075 | 780309 | [CIDEC](http://www.ncbi.nlm.nih.gov/entrez/query.fcgi?cmd=search&db=gene&term=CIDEC) |  |
| 0.0004851 | 0.407673 | 1969.39594 | 254.4234212 | 7.7406236 | 4810445 | [PCOLCE2](http://www.ncbi.nlm.nih.gov/entrez/query.fcgi?cmd=search&db=gene&term=PCOLCE2) |  |
| 0.0017207 | 0.484726 | 794.7563877 | 140.8270193 | 5.6434936 | 3800524 | [PLIN](http://www.ncbi.nlm.nih.gov/entrez/query.fcgi?cmd=search&db=gene&term=PLIN) | PPAR signaling pathway |
| 0.0026292 | 0.488743 | 684.8577018 | 123.1172058 | 5.5626482 | 1940747 | [PPP1R1B](http://www.ncbi.nlm.nih.gov/entrez/query.fcgi?cmd=search&db=gene&term=PPP1R1B) | FOSB gene expression and drug abuse, Regulation of ck1/cdk5 by type 1 glutamate receptors |
| 0.0009395 | 0.427352 | 1875.728705 | 365.5510811 | 5.1312356 | 2100228 | [DPT](http://www.ncbi.nlm.nih.gov/entrez/query.fcgi?cmd=search&db=gene&term=DPT) |  |
| 0.0032185 | 0.488743 | 959.6380551 | 195.9166991 | 4.8981943 | 1940037 | [MYOC](http://www.ncbi.nlm.nih.gov/entrez/query.fcgi?cmd=search&db=gene&term=MYOC) |  |
| 0.0026041 | 0.488743 | 2619.070765 | 545.2745116 | 4.8032151 | 6960379 | [SFRP1](http://www.ncbi.nlm.nih.gov/entrez/query.fcgi?cmd=search&db=gene&term=SFRP1) | Wnt signaling pathway, development |
| 0.0023299 | 0.488743 | 1935.557011 | 410.7035901 | 4.7127833 | 7040372 | [GPAM](http://www.ncbi.nlm.nih.gov/entrez/query.fcgi?cmd=search&db=gene&term=GPAM) | Glycerolipid metabolism, Glycerophospholipid metabolism |
| 0.0016365 | 0.484726 | 4455.191867 | 1078.014994 | 4.1327736 | 3370347 | [ADH1A](http://www.ncbi.nlm.nih.gov/entrez/query.fcgi?cmd=search&db=gene&term=ADH1A) | 1- and 2-Methylnaphthalene degradation, Bile acid biosynthesis, Fatty acid metabolism, Glycerolipid metabolism, Glycolysis / Gluconeogenesis, Metabolism of xenobiotics by cytochrome P450, Tyrosine metabolism |

| 0.0027915 | 0.488743 | 3987.211653 | 1058.497022 | 3.7668615 | 7380719 | [IGFBP6](http://www.ncbi.nlm.nih.gov/entrez/query.fcgi?cmd=search&db=gene&term=IGFBP6) | Ghrelin: Regulation of Food Intake and Energy Homeostasis |
| --- | --- | --- | --- | --- | --- | --- | --- |
| 0.0047801 | 0.488743 | 583.2539706 | 163.8420377 | 3.5598554 | 4070241 | [GYG2](http://www.ncbi.nlm.nih.gov/entrez/query.fcgi?cmd=search&db=gene&term=GYG2) |  |
| 0.0006608 | 0.427352 | 647.9051028 | 193.8635941 | 3.3420669 | 4880204 | [SPRYD5](http://www.ncbi.nlm.nih.gov/entrez/query.fcgi?cmd=search&db=gene&term=SPRYD5) |  |
| 0.0017562 | 0.484726 | 1030.477194 | 314.0264492 | 3.2814981 | 770564 | [C1orf115](http://www.ncbi.nlm.nih.gov/entrez/query.fcgi?cmd=search&db=gene&term=C1orf115) |  |
| 0.004475 | 0.488743 | 2062.195537 | 676.179786 | 3.049774 | 6840192 | [ECM2](http://www.ncbi.nlm.nih.gov/entrez/query.fcgi?cmd=search&db=gene&term=ECM2) |  |
| 0.0012814 | 0.468254 | 1225.894201 | 422.5251935 | 2.9013517 | 7160253 | [LAMA4](http://www.ncbi.nlm.nih.gov/entrez/query.fcgi?cmd=search&db=gene&term=LAMA4) | Cell Communication, ECM-receptor interaction, Focal adhesion, angiogenesis |
| 0.002679 | 0.488743 | 628.0063323 | 217.0594763 | 2.8932454 | 3370327 | [MOSC1](http://www.ncbi.nlm.nih.gov/entrez/query.fcgi?cmd=search&db=gene&term=MOSC1) |  |
| 0.0002144 | 0.366309 | 795.3342344 | 280.2965817 | 2.8374739 | 4260441 | [CLEC3B](http://www.ncbi.nlm.nih.gov/entrez/query.fcgi?cmd=search&db=gene&term=CLEC3B) |  |
| 0.0049002 | 0.488743 | 414.8037163 | 146.2188689 | 2.8368686 | 3870246 | [NTRK2](http://www.ncbi.nlm.nih.gov/entrez/query.fcgi?cmd=search&db=gene&term=NTRK2) | MAPK signaling pathway, signal_transduction |
| 0.004083 | 0.488743 | 646.0497395 | 232.5457819 | 2.7781615 | 1090326 | [TIMP4](http://www.ncbi.nlm.nih.gov/entrez/query.fcgi?cmd=search&db=gene&term=TIMP4) | Inhibition of Matrix Metalloproteinases, misc |
| 0.004704 | 0.488743 | 440.0001693 | 163.1982468 | 2.6961084 | 990458 | [SLIT3](http://www.ncbi.nlm.nih.gov/entrez/query.fcgi?cmd=search&db=gene&term=SLIT3) | Axon guidance |
| 0.003198 | 0.488743 | 2159.396834 | 824.3779355 | 2.6194258 | 2710730 | [PLS3](http://www.ncbi.nlm.nih.gov/entrez/query.fcgi?cmd=search&db=gene&term=PLS3) |  |
| 0.0013004 | 0.468254 | 1497.990116 | 572.1582318 | 2.6181396 | 5090079 | [C1QTNF1](http://www.ncbi.nlm.nih.gov/entrez/query.fcgi?cmd=search&db=gene&term=C1QTNF1) |  |
| 0.0019106 | 0.484726 | 244.2994692 | 93.3429834 | 2.6172237 | 4200471 | [ADIPOQ](http://www.ncbi.nlm.nih.gov/entrez/query.fcgi?cmd=search&db=gene&term=ADIPOQ) | Visceral Fat Deposits and the Metabolic Syndrome, Adipocytokine signaling pathway, PPAR signaling pathway, Type II diabetes mellitus |
| 0.0014445 | 0.47141 | 10114.58919 | 3870.671136 | 2.6131358 | 1070215 | [CAV1](http://www.ncbi.nlm.nih.gov/entrez/query.fcgi?cmd=search&db=gene&term=CAV1) | Actions of Nitric Oxide in the Heart, Integrin Signaling Pathway, Focal adhesion |
| 0.0013293 | 0.468254 | 393.6124849 | 155.2692861 | 2.5350312 | 3440037 | [PALM](http://www.ncbi.nlm.nih.gov/entrez/query.fcgi?cmd=search&db=gene&term=PALM) |  |
| 0.0014371 | 0.47141 | 3563.715472 | 1425.370753 | 2.5002025 | 7650672 | [ANGPTL2](http://www.ncbi.nlm.nih.gov/entrez/query.fcgi?cmd=search&db=gene&term=ANGPTL2) |  |
| 0.000196 | 0.355801 | 501.5611659 | 202.8821446 | 2.4721799 | 4290315 | [LTBP4](http://www.ncbi.nlm.nih.gov/entrez/query.fcgi?cmd=search&db=gene&term=LTBP4) |  |
| 0.0020147 | 0.48698 | 489.0733463 | 198.9262866 | 2.4585657 | 7320669 | [DKFZP586H2123](http://www.ncbi.nlm.nih.gov/entrez/query.fcgi?cmd=search&db=gene&term=DKFZP586H2123) |  |
| 0.0035195 | 0.488743 | 6516.381514 | 2673.368438 | 2.4375172 | 5860152 | [CD44](http://www.ncbi.nlm.nih.gov/entrez/query.fcgi?cmd=search&db=gene&term=CD44) | Adhesion Molecules on Lymphocyte, Monocyte and its Surface Molecules, Neutrophil and Its Surface Molecules, ECM-receptor interaction, Hematopoietic cell lineage, cell_signaling, immunology, metastasis |
| 0.0009636 | 0.427352 | 1503.869955 | 629.0231981 | 2.3908021 | 6980253 | [PTPLB](http://www.ncbi.nlm.nih.gov/entrez/query.fcgi?cmd=search&db=gene&term=PTPLB) |  |
| 0.0019202 | 0.484726 | 893.7669001 | 374.0167941 | 2.3896438 | 4560270 | [PTPLB](http://www.ncbi.nlm.nih.gov/entrez/query.fcgi?cmd=search&db=gene&term=PTPLB) |  |
| 0.0032104 | 0.488743 | 628.7552673 | 264.0372424 | 2.3813128 | 2490152 | [ENPP2](http://www.ncbi.nlm.nih.gov/entrez/query.fcgi?cmd=search&db=gene&term=ENPP2) | Nicotinate and nicotinamide metabolism, Pantothenate and CoA biosynthesis, Purine metabolism, Riboflavin metabolism, Starch and sucrose metabolism |
| 0.0041301 | 0.488743 | 527.9681145 | 222.5333066 | 2.3725353 | 3890484 | [ANG](http://www.ncbi.nlm.nih.gov/entrez/query.fcgi?cmd=search&db=gene&term=ANG) | angiogenesis |
| 0.00122 | 0.466249 | 528.3287611 | 222.6866159 | 2.3725214 | 1230594 | [MEOX1](http://www.ncbi.nlm.nih.gov/entrez/query.fcgi?cmd=search&db=gene&term=MEOX1) |  |
| 0.0028906 | 0.488743 | 333.5577299 | 140.7496348 | 2.3698657 | 3890095 | [SOD3](http://www.ncbi.nlm.nih.gov/entrez/query.fcgi?cmd=search&db=gene&term=SOD3) | The IGF-1 Receptor and Longevity, pharmacology |
| 0.0024598 | 0.488743 | 2004.007615 | 858.4218588 | 2.3345254 | 2030577 | [TIMP3](http://www.ncbi.nlm.nih.gov/entrez/query.fcgi?cmd=search&db=gene&term=TIMP3) | Inhibition of Matrix Metalloproteinases, p53 Signaling Pathway, immunology |
| 0.0003418 | 0.373332 | 1060.62013 | 460.1492016 | 2.3049483 | 6900703 | [ARHGEF10](http://www.ncbi.nlm.nih.gov/entrez/query.fcgi?cmd=search&db=gene&term=ARHGEF10) |  |
| 0.0046863 | 0.488743 | 3833.962863 | 1671.685796 | 2.293471 | 4040706 | [CD34](http://www.ncbi.nlm.nih.gov/entrez/query.fcgi?cmd=search&db=gene&term=CD34) | Adhesion and Diapedesis of Lymphocytes, IL 17 Signaling Pathway, Cell adhesion molecules (CAMs), Hematopoietic cell lineage, angiogenesis, metastasis |
| 0.0001541 | 0.355801 | 1492.951919 | 693.791706 | 2.1518734 | 7000382 | [PQLC3](http://www.ncbi.nlm.nih.gov/entrez/query.fcgi?cmd=search&db=gene&term=PQLC3) |  |
| 0.0046601 | 0.488743 | 1582.135986 | 744.7391546 | 2.1244163 | 4880537 | [LIMA1](http://www.ncbi.nlm.nih.gov/entrez/query.fcgi?cmd=search&db=gene&term=LIMA1) |  |
| 0.0019359 | 0.484726 | 2366.862631 | 1126.140122 | 2.1017479 | 240333 | [ETS1](http://www.ncbi.nlm.nih.gov/entrez/query.fcgi?cmd=search&db=gene&term=ETS1) | Keratinocyte Differentiation, METS affect on Macrophage Differentiation, Dorso-ventral axis formation, tsonc |
| 0.0022598 | 0.488743 | 414.1118008 | 198.0890223 | 2.0905338 | 3390543 | [C13orf33](http://www.ncbi.nlm.nih.gov/entrez/query.fcgi?cmd=search&db=gene&term=C13orf33) |  |
| 8.26E-05 | 0.355801 | 235.4481646 | 113.5840191 | 2.0728987 | 650753 | [AFF3](http://www.ncbi.nlm.nih.gov/entrez/query.fcgi?cmd=search&db=gene&term=AFF3) |  |
| 0.0004721 | 0.407673 | 307.2706126 | 148.5942866 | 2.0678494 | 6420630 | [SCARA3](http://www.ncbi.nlm.nih.gov/entrez/query.fcgi?cmd=search&db=gene&term=SCARA3) |  |
| 0.0008674 | 0.427352 | 655.7445487 | 318.6512352 | 2.0578754 | 3710554 | [FGD5](http://www.ncbi.nlm.nih.gov/entrez/query.fcgi?cmd=search&db=gene&term=FGD5) |  |
| 0.0045917 | 0.488743 | 205.5227069 | 100.8209893 | 2.0384913 | 5810053 | [GLYAT](http://www.ncbi.nlm.nih.gov/entrez/query.fcgi?cmd=search&db=gene&term=GLYAT) |  |
| 0.0018916 | 0.484726 | 1815.551802 | 902.1060881 | 2.0125702 | 2640377 | [PGCP](http://www.ncbi.nlm.nih.gov/entrez/query.fcgi?cmd=search&db=gene&term=PGCP) |  |
| 0.0045067 | 0.488743 | 275.141694 | 137.4805549 | 2.0013135 | 1510181 | [PTGES](http://www.ncbi.nlm.nih.gov/entrez/query.fcgi?cmd=search&db=gene&term=PTGES) | Eicosanoid Metabolism, Arachidonic acid metabolism |
| 0.0007753 | 0.427352 | 534.7335661 | 268.1735606 | 1.9939832 | 1820743 |  |  |
| 0.0014229 | 0.47141 | 7098.259523 | 3561.380711 | 1.9931201 | 4180343 | [YWHAZ](http://www.ncbi.nlm.nih.gov/entrez/query.fcgi?cmd=search&db=gene&term=YWHAZ) | Cell cycle |
| 0.0042872 | 0.488743 | 6364.146132 | 3197.730756 | 1.990207 | 3290685 | [DPYSL2](http://www.ncbi.nlm.nih.gov/entrez/query.fcgi?cmd=search&db=gene&term=DPYSL2) | Axon guidance |
| 0.000973 | 0.427352 | 285.5274194 | 144.2387314 | 1.9795475 | 1030646 | [FLJ43692](http://www.ncbi.nlm.nih.gov/entrez/query.fcgi?cmd=search&db=gene&term=FLJ43692) |  |
| 0.0046694 | 0.488743 | 1332.075461 | 674.8740055 | 1.9738136 | 6900630 | [ATP2B4](http://www.ncbi.nlm.nih.gov/entrez/query.fcgi?cmd=search&db=gene&term=ATP2B4) | Calcium signaling pathway |
| 0.0014679 | 0.473724 | 540.5616142 | 283.4529715 | 1.9070593 | 130390 | [OLFML1](http://www.ncbi.nlm.nih.gov/entrez/query.fcgi?cmd=search&db=gene&term=OLFML1) |  |
| 3.63E-05 | 0.355801 | 205.2045192 | 108.1448084 | 1.8974976 | 7000446 | [PRELP](http://www.ncbi.nlm.nih.gov/entrez/query.fcgi?cmd=search&db=gene&term=PRELP) |  |
| 0.0033395 | 0.488743 | 862.9035918 | 457.1298169 | 1.8876555 | 6580487 | [HSD17B11](http://www.ncbi.nlm.nih.gov/entrez/query.fcgi?cmd=search&db=gene&term=HSD17B11) |  |
| 0.0038868 | 0.488743 | 341.2460274 | 181.2673324 | 1.8825567 | 6130292 | [LOC728411](http://www.ncbi.nlm.nih.gov/entrez/query.fcgi?cmd=search&db=gene&term=LOC728411) |  |
| 0.0024466 | 0.488743 | 547.0257534 | 291.5937333 | 1.875986 | 2360196 | [DOCK1](http://www.ncbi.nlm.nih.gov/entrez/query.fcgi?cmd=search&db=gene&term=DOCK1) | Signaling of Hepatocyte Growth Factor Receptor, Focal adhesion, Regulation of actin cytoskeleton, immunology |
| 0.0036596 | 0.488743 | 4946.103133 | 2642.86122 | 1.8714956 | 2970730 | [MYADM](http://www.ncbi.nlm.nih.gov/entrez/query.fcgi?cmd=search&db=gene&term=MYADM) |  |
| 0.003028 | 0.488743 | 12496.69839 | 6766.439515 | 1.8468647 | 6380315 | [ITM2B](http://www.ncbi.nlm.nih.gov/entrez/query.fcgi?cmd=search&db=gene&term=ITM2B) |  |
| 0.0020228 | 0.48698 | 2111.310633 | 1153.276666 | 1.8307061 | 3180039 | [RGL1](http://www.ncbi.nlm.nih.gov/entrez/query.fcgi?cmd=search&db=gene&term=RGL1) |  |
| 0.002039 | 0.48698 | 813.4673415 | 451.8296303 | 1.8003851 | 6330615 | [GIMAP6](http://www.ncbi.nlm.nih.gov/entrez/query.fcgi?cmd=search&db=gene&term=GIMAP6) |  |
| 0.0029917 | 0.488743 | 252.7942552 | 141.9414794 | 1.7809752 | 1940390 | [PARVA](http://www.ncbi.nlm.nih.gov/entrez/query.fcgi?cmd=search&db=gene&term=PARVA) | Focal adhesion |
| 0.0049554 | 0.488743 | 518.9299765 | 294.2155986 | 1.7637745 | 2510224 | [ANKRD57](http://www.ncbi.nlm.nih.gov/entrez/query.fcgi?cmd=search&db=gene&term=ANKRD57) |  |
| 0.0032064 | 0.488743 | 732.9434582 | 417.9629823 | 1.7536085 | 7160435 | [EBF3](http://www.ncbi.nlm.nih.gov/entrez/query.fcgi?cmd=search&db=gene&term=EBF3) |  |
| 0.0030403 | 0.488743 | 5089.01564 | 2916.702043 | 1.7447842 | 110167 | [TRAM1](http://www.ncbi.nlm.nih.gov/entrez/query.fcgi?cmd=search&db=gene&term=TRAM1) |  |
| 0.0037627 | 0.488743 | 232.3344663 | 135.7927772 | 1.7109486 | 780500 | [CAB39L](http://www.ncbi.nlm.nih.gov/entrez/query.fcgi?cmd=search&db=gene&term=CAB39L) |  |
| 0.003509 | 0.488743 | 5103.92156 | 3003.768566 | 1.6991727 | 620300 | [LASP1](http://www.ncbi.nlm.nih.gov/entrez/query.fcgi?cmd=search&db=gene&term=LASP1) |  |
| 0.0006317 | 0.427352 | 1276.068958 | 751.5088307 | 1.6980093 | 2120451 | [CD46](http://www.ncbi.nlm.nih.gov/entrez/query.fcgi?cmd=search&db=gene&term=CD46) | Complement and coagulation cascades |
| 0.0041286 | 0.488743 | 375.8687738 | 221.7358732 | 1.6951194 | 60014 | [FAM84B](http://www.ncbi.nlm.nih.gov/entrez/query.fcgi?cmd=search&db=gene&term=FAM84B) |  |
| 0.00143 | 0.47141 | 207.7740418 | 122.9579029 | 1.6897982 | 460338 | [PLOD2](http://www.ncbi.nlm.nih.gov/entrez/query.fcgi?cmd=search&db=gene&term=PLOD2) | Lysine degradation |
| 0.0038747 | 0.488743 | 293.7337218 | 174.9168669 | 1.6792761 | 580121 | [SNX1](http://www.ncbi.nlm.nih.gov/entrez/query.fcgi?cmd=search&db=gene&term=SNX1) |  |
| 0.0017938 | 0.484726 | 224.2200701 | 133.9107092 | 1.6743998 | 1010746 | [C4orf31](http://www.ncbi.nlm.nih.gov/entrez/query.fcgi?cmd=search&db=gene&term=C4orf31) |  |
| 0.0006627 | 0.427352 | 1545.753966 | 928.2983158 | 1.6651479 | 7610561 | [OAT](http://www.ncbi.nlm.nih.gov/entrez/query.fcgi?cmd=search&db=gene&term=OAT) | Catabolic Pathways for Arginine , Histidine, Glutamate, Glutamine, and Proline, Arginine and proline metabolism, Urea cycle and metabolism of amino groups, immunology, metabolism |
| 0.0040449 | 0.488743 | 542.4578937 | 325.9742907 | 1.6641125 | 1260241 | [EIF4EBP2](http://www.ncbi.nlm.nih.gov/entrez/query.fcgi?cmd=search&db=gene&term=EIF4EBP2) |  |
| 0.0013381 | 0.468254 | 981.552869 | 592.0569004 | 1.6578691 | 4150193 | [CD47](http://www.ncbi.nlm.nih.gov/entrez/query.fcgi?cmd=search&db=gene&term=CD47) | ECM-receptor interaction, angiogenesis, cell_signaling, immunology, metastasis |
| 0.0044995 | 0.488743 | 1205.301593 | 727.9699527 | 1.6557024 | 5870746 | [PPP2R5A](http://www.ncbi.nlm.nih.gov/entrez/query.fcgi?cmd=search&db=gene&term=PPP2R5A) | cell_cycle, cell_signaling, signal_transduction |
| 0.0008052 | 0.427352 | 2954.427092 | 1794.848413 | 1.6460594 | 830278 | [C9orf19](http://www.ncbi.nlm.nih.gov/entrez/query.fcgi?cmd=search&db=gene&term=C9orf19) |  |
| 0.0027593 | 0.488743 | 386.6319155 | 238.1781189 | 1.623289 | 4210280 | [PGCP](http://www.ncbi.nlm.nih.gov/entrez/query.fcgi?cmd=search&db=gene&term=PGCP) |  |
| 0.0023035 | 0.488743 | 478.2856279 | 295.5135278 | 1.6184898 | 870056 | [FAM119B](http://www.ncbi.nlm.nih.gov/entrez/query.fcgi?cmd=search&db=gene&term=FAM119B) |  |
| 0.0041734 | 0.488743 | 636.0328744 | 393.3231707 | 1.6170745 | 4290575 | [C7orf23](http://www.ncbi.nlm.nih.gov/entrez/query.fcgi?cmd=search&db=gene&term=C7orf23) |  |
| 0.0046139 | 0.488743 | 238.0409587 | 148.12274 | 1.6070521 | 3450114 | [FAM69B](http://www.ncbi.nlm.nih.gov/entrez/query.fcgi?cmd=search&db=gene&term=FAM69B) |  |
| 0.0008251 | 0.427352 | 186.121057 | 116.3933491 | 1.5990695 | 2810471 | [PTGDR](http://www.ncbi.nlm.nih.gov/entrez/query.fcgi?cmd=search&db=gene&term=PTGDR) | Neuroactive ligand-receptor interaction |
| 0.003632 | 0.488743 | 469.1486707 | 293.7702036 | 1.596992 | 4230373 | [MAP3K1](http://www.ncbi.nlm.nih.gov/entrez/query.fcgi?cmd=search&db=gene&term=MAP3K1) |  |
| 0.0035779 | 0.488743 | 415.5559811 | 260.5681729 | 1.5948071 | 3780619 | [TMEM14A](http://www.ncbi.nlm.nih.gov/entrez/query.fcgi?cmd=search&db=gene&term=TMEM14A) |  |
| 0.0001746 | 0.355801 | 179.2754829 | 112.414182 | 1.5947764 | 3460040 |  |  |
| 0.001052 | 0.436505 | 376.0682183 | 238.2900882 | 1.578195 | 7550181 | [IFNAR1](http://www.ncbi.nlm.nih.gov/entrez/query.fcgi?cmd=search&db=gene&term=IFNAR1) | Bone Remodelling, IFN alpha signaling pathway, Cytokine-cytokine receptor interaction, Jak-STAT signaling pathway, Natural killer cell mediated cytotoxicity, Toll-like receptor signaling pathway, immunology |
| 0.0037578 | 0.488743 | 540.579481 | 343.0602827 | 1.5757565 | 2000064 | [CTPS2](http://www.ncbi.nlm.nih.gov/entrez/query.fcgi?cmd=search&db=gene&term=CTPS2) | Pyrimidine metabolism |
| 0.0027217 | 0.488743 | 262.4846599 | 166.8749315 | 1.5729424 | 7380338 | [UST](http://www.ncbi.nlm.nih.gov/entrez/query.fcgi?cmd=search&db=gene&term=UST) | Chondroitin sulfate biosynthesis, Glycan structures - biosynthesis 1 |
| 0.0049211 | 0.488743 | 807.2963322 | 514.1741289 | 1.5700835 | 3310674 | [PAN3](http://www.ncbi.nlm.nih.gov/entrez/query.fcgi?cmd=search&db=gene&term=PAN3) |  |
| 0.0006133 | 0.427352 | 295.1946035 | 188.8366446 | 1.5632273 | 5290538 | [PER3](http://www.ncbi.nlm.nih.gov/entrez/query.fcgi?cmd=search&db=gene&term=PER3) | Circadian rhythm |
| 0.0028278 | 0.488743 | 396.5746764 | 254.260924 | 1.5597154 | 6860593 |  |  |
| 0.0031933 | 0.488743 | 284.8711095 | 182.6694801 | 1.5594894 | 3420332 | [SLC37A3](http://www.ncbi.nlm.nih.gov/entrez/query.fcgi?cmd=search&db=gene&term=SLC37A3) |  |
| 0.0028897 | 0.488743 | 314.1635011 | 202.5783727 | 1.5508245 | 2350142 | [C2orf32](http://www.ncbi.nlm.nih.gov/entrez/query.fcgi?cmd=search&db=gene&term=C2orf32) |  |
| 0.0025351 | 0.488743 | 193.3944521 | 125.185871 | 1.5448585 | 4060332 | [IGF1](http://www.ncbi.nlm.nih.gov/entrez/query.fcgi?cmd=search&db=gene&term=IGF1) | Control of skeletal myogenesis by HDAC & calcium/calmodulin-dependent kinase (CaMK), Erythrocyte Differentiation Pathway, Ghrelin: Regulation of Food Intake and Energy Homeostasis, IGF-1 Signaling Pathway, NFAT and Hypertrophy of the heart (Transcription in the broken heart), Regulation of BAD phosphorylation, Skeletal muscle hypertrophy is regulated via AKT/mTOR pathway, The IGF-1 Receptor and Longevity, Focal adhesion, Long-term depression, mTOR signaling pathway, angiogenesis, immunology, misc |
| 0.0036851 | 0.488743 | 6810.728769 | 4415.012058 | 1.5426297 | 630356 | [DAZAP2](http://www.ncbi.nlm.nih.gov/entrez/query.fcgi?cmd=search&db=gene&term=DAZAP2) |  |
| 0.0044717 | 0.488743 | 374.5934076 | 243.021039 | 1.5414032 | 3170491 | [PDIA5](http://www.ncbi.nlm.nih.gov/entrez/query.fcgi?cmd=search&db=gene&term=PDIA5) |  |
| 3.99E-05 | 0.355801 | 481.6883001 | 312.6628304 | 1.5405998 | 5900561 | [SFRS3](http://www.ncbi.nlm.nih.gov/entrez/query.fcgi?cmd=search&db=gene&term=SFRS3) |  |
| 0.0041877 | 0.488743 | 397.1029333 | 258.9594096 | 1.5334563 | 2750154 | [ELMO1](http://www.ncbi.nlm.nih.gov/entrez/query.fcgi?cmd=search&db=gene&term=ELMO1) |  |
| 0.0043212 | 0.488743 | 736.6710985 | 482.2740859 | 1.5274947 | 1050082 | [KIAA1147](http://www.ncbi.nlm.nih.gov/entrez/query.fcgi?cmd=search&db=gene&term=KIAA1147) |  |
| 0.0035986 | 0.488743 | 501.7104686 | 334.1523525 | 1.5014423 | 4900193 | [ZCCHC7](http://www.ncbi.nlm.nih.gov/entrez/query.fcgi?cmd=search&db=gene&term=ZCCHC7) |  |
| 0.0005474 | 0.407673 | 173.6722371 | 115.962258 | 1.4976617 | 6560196 |  |  |
| 0.0001635 | 0.355801 | 2592.64808 | 1736.463854 | 1.4930619 | 1300750 | [GOLGA7](http://www.ncbi.nlm.nih.gov/entrez/query.fcgi?cmd=search&db=gene&term=GOLGA7) |  |
| 0.0045727 | 0.488743 | 202.1314171 | 135.5852295 | 1.4908071 | 4200142 | [REM1](http://www.ncbi.nlm.nih.gov/entrez/query.fcgi?cmd=search&db=gene&term=REM1) |  |
| 0.0002713 | 0.373332 | 450.8023342 | 302.5577926 | 1.489971 | 60132 | [DCBLD2](http://www.ncbi.nlm.nih.gov/entrez/query.fcgi?cmd=search&db=gene&term=DCBLD2) |  |
| 0.0017585 | 0.484726 | 2415.217185 | 1641.7 | 1.4711684 | 4640066 | [VAMP3](http://www.ncbi.nlm.nih.gov/entrez/query.fcgi?cmd=search&db=gene&term=VAMP3) | SNARE interactions in vesicular transport |
| 0.0034827 | 0.488743 | 157.3032477 | 107.1599664 | 1.4679292 | 1440324 | [PPARG](http://www.ncbi.nlm.nih.gov/entrez/query.fcgi?cmd=search&db=gene&term=PPARG) | Basic mechanism of action of PPARa, PPARb(d) and PPARg and effects on gene expression, Nuclear Receptors in Lipid Metabolism and Toxicity, Role of PPAR-gamma Coactivators in Obesity and Thermogenesis, Visceral Fat Deposits and the Metabolic Syndrome, PPAR signaling pathway |
| 0.0021979 | 0.488743 | 156.1621615 | 106.4658642 | 1.4667815 | 130156 | [PDE1B](http://www.ncbi.nlm.nih.gov/entrez/query.fcgi?cmd=search&db=gene&term=PDE1B) | Calcium signaling pathway, Purine metabolism |
| 0.0003583 | 0.373332 | 709.7096297 | 484.6459864 | 1.4643877 | 20487 | [RNF13](http://www.ncbi.nlm.nih.gov/entrez/query.fcgi?cmd=search&db=gene&term=RNF13) |  |
| 0.0003728 | 0.373379 | 414.9386449 | 283.566386 | 1.4632857 | 3450092 | [PELI1](http://www.ncbi.nlm.nih.gov/entrez/query.fcgi?cmd=search&db=gene&term=PELI1) |  |
| 0.0016103 | 0.484726 | 160.4297397 | 109.8251557 | 1.4607741 | 770193 | [ITIH5](http://www.ncbi.nlm.nih.gov/entrez/query.fcgi?cmd=search&db=gene&term=ITIH5) |  |
| 0.0010802 | 0.441893 | 336.6228944 | 232.6830058 | 1.4467017 | 1230333 | [SS18](http://www.ncbi.nlm.nih.gov/entrez/query.fcgi?cmd=search&db=gene&term=SS18) |  |
| 0.0025431 | 0.488743 | 143.6859776 | 99.5245826 | 1.4437235 | 6650128 | [PRELP](http://www.ncbi.nlm.nih.gov/entrez/query.fcgi?cmd=search&db=gene&term=PRELP) |  |
| 0.0039924 | 0.488743 | 211.9577109 | 149.0139031 | 1.4224022 | 110397 | [LOC652755](http://www.ncbi.nlm.nih.gov/entrez/query.fcgi?cmd=search&db=gene&term=LOC652755) |  |
| 0.0009858 | 0.427352 | 10497.72117 | 7407.177504 | 1.4172363 | 1300671 | [NCOA4](http://www.ncbi.nlm.nih.gov/entrez/query.fcgi?cmd=search&db=gene&term=NCOA4) |  |
| 0.0044009 | 0.488743 | 295.9990907 | 208.9852902 | 1.4163633 | 5570615 | [FLJ11171](http://www.ncbi.nlm.nih.gov/entrez/query.fcgi?cmd=search&db=gene&term=FLJ11171) |  |
| 0.0039077 | 0.488743 | 242.4209991 | 171.2613399 | 1.4155033 | 2370348 | [SYDE1](http://www.ncbi.nlm.nih.gov/entrez/query.fcgi?cmd=search&db=gene&term=SYDE1) |  |
| 0.0042706 | 0.488743 | 333.0415951 | 236.5130345 | 1.4081321 | 4830239 | [ITPKB](http://www.ncbi.nlm.nih.gov/entrez/query.fcgi?cmd=search&db=gene&term=ITPKB) | Calcium signaling pathway, Inositol phosphate metabolism, Phosphatidylinositol signaling system, cell_signaling, signal_transduction |
| 0.0047572 | 0.488743 | 2763.548183 | 1967.005364 | 1.404952 | 5820619 | [HNRPK](http://www.ncbi.nlm.nih.gov/entrez/query.fcgi?cmd=search&db=gene&term=HNRPK) | gene_regulation, transcription |
| 0.003921 | 0.488743 | 154.1662558 | 110.5736865 | 1.39424 | 510180 | [FOXD2](http://www.ncbi.nlm.nih.gov/entrez/query.fcgi?cmd=search&db=gene&term=FOXD2) |  |
| 0.0041827 | 0.488743 | 181.8006798 | 130.9058199 | 1.3887899 | 240228 | [MRGPRF](http://www.ncbi.nlm.nih.gov/entrez/query.fcgi?cmd=search&db=gene&term=MRGPRF) |  |
| 0.0005383 | 0.407673 | 610.2242753 | 440.6365984 | 1.3848697 | 6420017 | [SIN3A](http://www.ncbi.nlm.nih.gov/entrez/query.fcgi?cmd=search&db=gene&term=SIN3A) | NA |
| 0.0004492 | 0.407673 | 259.7954094 | 188.3106981 | 1.3796105 | 1070373 | [SNX30](http://www.ncbi.nlm.nih.gov/entrez/query.fcgi?cmd=search&db=gene&term=SNX30) |  |
| 0.0034936 | 0.488743 | 411.0057113 | 298.0119968 | 1.3791583 | 5560086 | [RIPK1](http://www.ncbi.nlm.nih.gov/entrez/query.fcgi?cmd=search&db=gene&term=RIPK1) | Acetylation and Deacetylation of RelA in The Nucleus, Ceramide Signaling Pathway, HIV-I Nef: negative effector of Fas and TNF, Induction of apoptosis through DR3 and DR4/5 Death Receptors , Keratinocyte Differentiation, MAPKinase Signaling Pathway, NF-kB Signaling Pathway, p38 MAPK Signaling Pathway , SODD/TNFR1 Signaling Pathway, TNF/Stress Related Signaling, TNFR1 Signaling Pathway, TNFR2 Signaling Pathway, Apoptosis |
| 0.0010056 | 0.429524 | 2115.834599 | 1553.010997 | 1.362408 | 650681 | [CTDSP2](http://www.ncbi.nlm.nih.gov/entrez/query.fcgi?cmd=search&db=gene&term=CTDSP2) |  |
| 0.0017808 | 0.484726 | 141.2696514 | 104.1578367 | 1.3563036 | 2490475 | [SH2D3C](http://www.ncbi.nlm.nih.gov/entrez/query.fcgi?cmd=search&db=gene&term=SH2D3C) |  |
| 0.0033029 | 0.488743 | 848.8800192 | 626.1563831 | 1.3556997 | 6580164 | [PHF21A](http://www.ncbi.nlm.nih.gov/entrez/query.fcgi?cmd=search&db=gene&term=PHF21A) |  |
| 0.0001511 | 0.355801 | 283.5712128 | 209.5422913 | 1.3532887 | 2450037 | [KIF3B](http://www.ncbi.nlm.nih.gov/entrez/query.fcgi?cmd=search&db=gene&term=KIF3B) |  |
| 0.0044152 | 0.488743 | 198.7031016 | 148.6724953 | 1.3365155 | 6350154 | [FAM122A](http://www.ncbi.nlm.nih.gov/entrez/query.fcgi?cmd=search&db=gene&term=FAM122A) |  |
| 0.0022311 | 0.488743 | 2512.582918 | 1923.446768 | 1.3062919 | 1110541 | [C20orf30](http://www.ncbi.nlm.nih.gov/entrez/query.fcgi?cmd=search&db=gene&term=C20orf30) |  |
| 0.0001467 | 0.355801 | 260.2387544 | 200.7418572 | 1.2963851 | 4760349 | [LRP10](http://www.ncbi.nlm.nih.gov/entrez/query.fcgi?cmd=search&db=gene&term=LRP10) |  |
| 0.0033429 | 0.488743 | 172.4005141 | 134.1857275 | 1.2847902 | 1660605 | [ZNF436](http://www.ncbi.nlm.nih.gov/entrez/query.fcgi?cmd=search&db=gene&term=ZNF436) |  |
| 0.0032568 | 0.488743 | 179.0034625 | 139.5789923 | 1.2824528 | 7160440 | [ARID4A](http://www.ncbi.nlm.nih.gov/entrez/query.fcgi?cmd=search&db=gene&term=ARID4A) |  |
| 0.0017595 | 0.484726 | 123.1743989 | 96.2451994 | 1.2797978 | 2510711 | [ITIH5](http://www.ncbi.nlm.nih.gov/entrez/query.fcgi?cmd=search&db=gene&term=ITIH5) |  |
| 0.0008741 | 0.427352 | 634.5350327 | 496.7056987 | 1.2774869 | 1090687 | [POLR1D](http://www.ncbi.nlm.nih.gov/entrez/query.fcgi?cmd=search&db=gene&term=POLR1D) | Purine metabolism, Pyrimidine metabolism, RNA polymerase |
| 0.0036169 | 0.488743 | 149.1719744 | 117.4496302 | 1.2700932 | 990044 | [KIAA1147](http://www.ncbi.nlm.nih.gov/entrez/query.fcgi?cmd=search&db=gene&term=KIAA1147) |  |
| 0.0020455 | 0.48698 | 131.245258 | 104.4392247 | 1.2566663 | 7330537 | [FUT10](http://www.ncbi.nlm.nih.gov/entrez/query.fcgi?cmd=search&db=gene&term=FUT10) |  |
| 0.0003351 | 0.373332 | 108.7076489 | 86.7380336 | 1.253287 | 3060528 | [DUOX1](http://www.ncbi.nlm.nih.gov/entrez/query.fcgi?cmd=search&db=gene&term=DUOX1) |  |
| 0.0012187 | 0.466249 | 120.6285341 | 96.652729 | 1.2480613 | 2120392 | [ANKRD42](http://www.ncbi.nlm.nih.gov/entrez/query.fcgi?cmd=search&db=gene&term=ANKRD42) |  |
| 0.0009365 | 0.427352 | 152.5406907 | 122.8731101 | 1.2414489 | 730035 | [RAP1GDS1](http://www.ncbi.nlm.nih.gov/entrez/query.fcgi?cmd=search&db=gene&term=RAP1GDS1) |  |
| 0.002813 | 0.488743 | 243.8206139 | 196.4750257 | 1.2409751 | 7100253 | [VAMP4](http://www.ncbi.nlm.nih.gov/entrez/query.fcgi?cmd=search&db=gene&term=VAMP4) | SNARE interactions in vesicular transport |
| 0.0016912 | 0.484726 | 567.6355422 | 458.0476002 | 1.2392501 | 2470762 | [KIAA0256](http://www.ncbi.nlm.nih.gov/entrez/query.fcgi?cmd=search&db=gene&term=KIAA0256) |  |
| 0.0035057 | 0.488743 | 186.2239562 | 150.7795926 | 1.235074 | 3120014 | [ABHD4](http://www.ncbi.nlm.nih.gov/entrez/query.fcgi?cmd=search&db=gene&term=ABHD4) |  |
| 0.0005264 | 0.407673 | 126.9583325 | 102.9251447 | 1.2335016 | 5860630 | [CCRL1](http://www.ncbi.nlm.nih.gov/entrez/query.fcgi?cmd=search&db=gene&term=CCRL1) |  |
| 0.0045962 | 0.488743 | 116.0177697 | 95.0259133 | 1.2209067 | 3460446 | [FAM5C](http://www.ncbi.nlm.nih.gov/entrez/query.fcgi?cmd=search&db=gene&term=FAM5C) |  |
| 0.0031939 | 0.488743 | 117.4347731 | 96.2066948 | 1.2206507 | 4250551 |  |  |
| 0.0034369 | 0.488743 | 181.8736719 | 149.5748181 | 1.2159378 | 1110494 | [TMEM194](http://www.ncbi.nlm.nih.gov/entrez/query.fcgi?cmd=search&db=gene&term=TMEM194) |  |
| 0.0031588 | 0.488743 | 115.8282175 | 95.2750257 | 1.2157249 | 6840441 | [IFNE1](http://www.ncbi.nlm.nih.gov/entrez/query.fcgi?cmd=search&db=gene&term=IFNE1) |  |
| 0.0035495 | 0.488743 | 253.5547277 | 209.4213563 | 1.2107396 | 1710102 | [EIF2C1](http://www.ncbi.nlm.nih.gov/entrez/query.fcgi?cmd=search&db=gene&term=EIF2C1) | Dicer Pathway |
| 0.004273 | 0.488743 | 110.7898211 | 93.9943678 | 1.1786857 | 6480546 | [WDR33](http://www.ncbi.nlm.nih.gov/entrez/query.fcgi?cmd=search&db=gene&term=WDR33) |  |
| 0.0049993 | 0.488743 | 115.1095045 | 97.9671565 | 1.1749806 | 3310397 | [ACCN5](http://www.ncbi.nlm.nih.gov/entrez/query.fcgi?cmd=search&db=gene&term=ACCN5) |  |
| 0.0007147 | 0.427352 | 114.7704176 | 98.1777821 | 1.169006 | 5720678 |  |  |
| 0.0028974 | 0.488743 | 116.3638206 | 99.6873195 | 1.1672881 | 6220768 | [PRDM6](http://www.ncbi.nlm.nih.gov/entrez/query.fcgi?cmd=search&db=gene&term=PRDM6) |  |
| 0.0045553 | 0.488743 | 122.5179224 | 105.5928137 | 1.1602866 | 2650612 | [LAMA3](http://www.ncbi.nlm.nih.gov/entrez/query.fcgi?cmd=search&db=gene&term=LAMA3) | Agrin in Postsynaptic Differentiation, Cell Communication, ECM-receptor interaction, Focal adhesion, immunology |
| 0.0004128 | 0.399659 | 111.4870718 | 96.3095317 | 1.1575913 | 6510092 |  |  |
| 0.0034757 | 0.488743 | 106.521175 | 92.0833141 | 1.1567913 | 1470066 |  |  |
| 0.0030608 | 0.488743 | 106.9932463 | 92.498496 | 1.1567026 | 6380253 |  |  |
| 0.0007055 | 0.427352 | 112.8339322 | 97.6206147 | 1.1558412 | 7160356 | [KIAA0565](http://www.ncbi.nlm.nih.gov/entrez/query.fcgi?cmd=search&db=gene&term=KIAA0565) |  |
| 0.0031445 | 0.488743 | 105.2539381 | 91.139975 | 1.1548603 | 4040689 |  |  |
| 0.0003599 | 0.373332 | 110.8571638 | 96.1788649 | 1.1526146 | 5560646 | [RBM43](http://www.ncbi.nlm.nih.gov/entrez/query.fcgi?cmd=search&db=gene&term=RBM43) |  |
| 0.0003354 | 0.373332 | 113.0297725 | 98.4086505 | 1.1485756 | 6280367 |  |  |
| 0.0001394 | 0.355801 | 106.5298716 | 93.1021844 | 1.1442253 | 4220100 | [LOC646299](http://www.ncbi.nlm.nih.gov/entrez/query.fcgi?cmd=search&db=gene&term=LOC646299) |  |
| 0.0047449 | 0.488743 | 111.9261552 | 98.1659279 | 1.1401731 | 730292 |  |  |
| 0.0045216 | 0.488743 | 150.9556393 | 132.6506051 | 1.1379944 | 5670470 | [KIAA0564](http://www.ncbi.nlm.nih.gov/entrez/query.fcgi?cmd=search&db=gene&term=KIAA0564) |  |
| 0.0038706 | 0.488743 | 107.7214997 | 95.0959104 | 1.1327669 | 6380010 |  |  |
| 0.0003263 | 0.373332 | 108.3835246 | 95.8799955 | 1.1304081 | 7330743 |  |  |
| 0.0016758 | 0.484726 | 126.6507147 | 112.0593746 | 1.1302108 | 2970669 | [AP4S1](http://www.ncbi.nlm.nih.gov/entrez/query.fcgi?cmd=search&db=gene&term=AP4S1) |  |
| 0.0022196 | 0.488743 | 108.8729351 | 96.6894201 | 1.1260067 | 5820475 |  |  |
| 0.004334 | 0.488743 | 109.2231585 | 97.5988542 | 1.1191029 | 2570424 |  |  |
| 0.0036482 | 0.488743 | 109.8450253 | 98.2925258 | 1.1175318 | 5310176 | [LOC643799](http://www.ncbi.nlm.nih.gov/entrez/query.fcgi?cmd=search&db=gene&term=LOC643799) |  |
| 0.0005028 | 0.407673 | 120.3671512 | 108.1370404 | 1.1130983 | 510196 |  |  |
| 0.0029695 | 0.488743 | 115.0376869 | 103.3491623 | 1.1130974 | 2260612 | [ZNF304](http://www.ncbi.nlm.nih.gov/entrez/query.fcgi?cmd=search&db=gene&term=ZNF304) |  |
| 0.0044352 | 0.488743 | 108.1536792 | 97.6285362 | 1.1078081 | 2350537 | [MMP7](http://www.ncbi.nlm.nih.gov/entrez/query.fcgi?cmd=search&db=gene&term=MMP7) | Wnt signaling pathway, immunology |
| 0.0031614 | 0.488743 | 104.8177461 | 96.0591854 | 1.0911788 | 610369 | [NUDT17](http://www.ncbi.nlm.nih.gov/entrez/query.fcgi?cmd=search&db=gene&term=NUDT17) |  |
| 0.0028142 | 0.488743 | 110.2296217 | 101.0906116 | 1.0904041 | 540010 | [ZNF655](http://www.ncbi.nlm.nih.gov/entrez/query.fcgi?cmd=search&db=gene&term=ZNF655) |  |
| 0.0015021 | 0.474223 | 102.23516 | 108.5996674 | 0.9413948 | 770240 |  |  |
| 0.0046977 | 0.488743 | 89.8632187 | 96.2557134 | 0.9335884 | 6450274 | [POLR3G](http://www.ncbi.nlm.nih.gov/entrez/query.fcgi?cmd=search&db=gene&term=POLR3G) | Purine metabolism, Pyrimidine metabolism, RNA polymerase |
| 0.0026899 | 0.488743 | 92.2370209 | 101.810009 | 0.905972 | 2690537 | [UBE2U](http://www.ncbi.nlm.nih.gov/entrez/query.fcgi?cmd=search&db=gene&term=UBE2U) |  |
| 0.0013971 | 0.47141 | 95.9952636 | 106.4003509 | 0.9022081 | 3310563 |  |  |
| 0.000859 | 0.427352 | 96.1238408 | 107.2579547 | 0.8961931 | 4390719 |  |  |
| 0.0019968 | 0.48698 | 89.7938481 | 100.5695134 | 0.8928536 | 6280541 | [LAMC3](http://www.ncbi.nlm.nih.gov/entrez/query.fcgi?cmd=search&db=gene&term=LAMC3) | Cell Communication, ECM-receptor interaction, Focal adhesion |
| 0.0024124 | 0.488743 | 116.6760852 | 130.7563105 | 0.892317 | 5290537 | [NUPL1](http://www.ncbi.nlm.nih.gov/entrez/query.fcgi?cmd=search&db=gene&term=NUPL1) |  |
| 0.0017575 | 0.484726 | 97.5535258 | 109.6468536 | 0.8897066 | 2000612 |  |  |
| 0.001883 | 0.484726 | 100.6952533 | 114.179231 | 0.8819052 | 160176 | [ZNF233](http://www.ncbi.nlm.nih.gov/entrez/query.fcgi?cmd=search&db=gene&term=ZNF233) |  |
| 0.0037042 | 0.488743 | 624.9879998 | 710.1877704 | 0.8800321 | 110270 | [MCM3AP](http://www.ncbi.nlm.nih.gov/entrez/query.fcgi?cmd=search&db=gene&term=MCM3AP) |  |
| 0.0016297 | 0.484726 | 92.5970606 | 106.0213091 | 0.8733816 | 840632 |  |  |
| 0.0044302 | 0.488743 | 15189.02941 | 17555.5616 | 0.8651976 | 3140019 | [LOC645317](http://www.ncbi.nlm.nih.gov/entrez/query.fcgi?cmd=search&db=gene&term=LOC645317) |  |
| 0.0047774 | 0.488743 | 96.3078976 | 111.3644215 | 0.8647995 | 2360754 | [LSM14B](http://www.ncbi.nlm.nih.gov/entrez/query.fcgi?cmd=search&db=gene&term=LSM14B) |  |
| 0.0012741 | 0.468254 | 94.1237778 | 109.2377892 | 0.8616412 | 6290019 |  |  |
| 0.0001924 | 0.355801 | 93.8990306 | 109.2176203 | 0.8597425 | 5310576 |  |  |
| 0.0047198 | 0.488743 | 86.9216464 | 101.5012284 | 0.8563605 | 2230273 | [AFF3](http://www.ncbi.nlm.nih.gov/entrez/query.fcgi?cmd=search&db=gene&term=AFF3) |  |
| 0.0031951 | 0.488743 | 93.904749 | 109.6632052 | 0.8563013 | 4610379 | [SDHALP1](http://www.ncbi.nlm.nih.gov/entrez/query.fcgi?cmd=search&db=gene&term=SDHALP1) |  |
| 0.0043708 | 0.488743 | 115.5854209 | 135.0247671 | 0.8560313 | 6180068 |  |  |
| 0.0035903 | 0.488743 | 450.2729558 | 526.6311674 | 0.8550063 | 6650201 | [CCDC97](http://www.ncbi.nlm.nih.gov/entrez/query.fcgi?cmd=search&db=gene&term=CCDC97) |  |
| 0.000925 | 0.427352 | 90.9792553 | 106.7310492 | 0.852416 | 2600189 | [MPG](http://www.ncbi.nlm.nih.gov/entrez/query.fcgi?cmd=search&db=gene&term=MPG) | DNA_damage |
| 0.0025129 | 0.488743 | 111.4323191 | 131.0980582 | 0.8499921 | 2970598 | [CELSR1](http://www.ncbi.nlm.nih.gov/entrez/query.fcgi?cmd=search&db=gene&term=CELSR1) |  |
| 0.0009368 | 0.427352 | 96.3924952 | 113.5354569 | 0.8490079 | 5270487 | [LOC643012](http://www.ncbi.nlm.nih.gov/entrez/query.fcgi?cmd=search&db=gene&term=LOC643012) |  |
| 0.0039275 | 0.488743 | 189.6233582 | 224.885788 | 0.8431985 | 4780653 | [MUSK](http://www.ncbi.nlm.nih.gov/entrez/query.fcgi?cmd=search&db=gene&term=MUSK) | Agrin in Postsynaptic Differentiation, Role of nicotinic acetylcholine receptors in the regulation of apoptosis |
| 0.0017879 | 0.484726 | 95.4141588 | 113.7474299 | 0.8388247 | 2120131 | [LOC728656](http://www.ncbi.nlm.nih.gov/entrez/query.fcgi?cmd=search&db=gene&term=LOC728656) |  |
| 0.0033558 | 0.488743 | 94.7337916 | 113.5178212 | 0.8345279 | 110735 | [SOX6](http://www.ncbi.nlm.nih.gov/entrez/query.fcgi?cmd=search&db=gene&term=SOX6) |  |
| 0.0008969 | 0.427352 | 117.4381597 | 140.9021412 | 0.8334732 | 60689 | [AAAS](http://www.ncbi.nlm.nih.gov/entrez/query.fcgi?cmd=search&db=gene&term=AAAS) |  |
| 0.0024901 | 0.488743 | 95.2855702 | 114.9397838 | 0.8290043 | 5420100 |  |  |
| 0.0028549 | 0.488743 | 125.6421805 | 152.4819236 | 0.8239808 | 620440 | [C21orf70](http://www.ncbi.nlm.nih.gov/entrez/query.fcgi?cmd=search&db=gene&term=C21orf70) |  |
| 0.0027258 | 0.488743 | 162.9352586 | 197.9436405 | 0.8231396 | 5720070 | [THAP6](http://www.ncbi.nlm.nih.gov/entrez/query.fcgi?cmd=search&db=gene&term=THAP6) |  |
| 0.0010324 | 0.434581 | 106.6658163 | 129.6487785 | 0.822729 | 1410044 | [R3HDM1](http://www.ncbi.nlm.nih.gov/entrez/query.fcgi?cmd=search&db=gene&term=R3HDM1) |  |
| 0.002487 | 0.488743 | 109.7607465 | 134.476555 | 0.8162073 | 6280300 | [PMS2L1](http://www.ncbi.nlm.nih.gov/entrez/query.fcgi?cmd=search&db=gene&term=PMS2L1) | DNA_damage |
| 0.0022111 | 0.488743 | 97.2363098 | 120.1378487 | 0.8093728 | 2260086 | [LOC650251](http://www.ncbi.nlm.nih.gov/entrez/query.fcgi?cmd=search&db=gene&term=LOC650251) |  |
| 0.0018576 | 0.484726 | 170.6505785 | 212.2002138 | 0.8041961 | 6290053 | [AUP1](http://www.ncbi.nlm.nih.gov/entrez/query.fcgi?cmd=search&db=gene&term=AUP1) |  |
| 0.0041742 | 0.488743 | 176.6303825 | 219.9720127 | 0.8029675 | 2030386 | [COPG2](http://www.ncbi.nlm.nih.gov/entrez/query.fcgi?cmd=search&db=gene&term=COPG2) |  |
| 0.0019199 | 0.484726 | 127.1774794 | 158.8709076 | 0.8005083 | 1710113 | [PPP1R3E](http://www.ncbi.nlm.nih.gov/entrez/query.fcgi?cmd=search&db=gene&term=PPP1R3E) |  |
| 0.0043928 | 0.488743 | 155.2979805 | 194.8179474 | 0.7971441 | 2100709 | [TMEM41A](http://www.ncbi.nlm.nih.gov/entrez/query.fcgi?cmd=search&db=gene&term=TMEM41A) |  |
| 0.0015744 | 0.484726 | 109.8815732 | 139.0188065 | 0.790408 | 6580670 | [ITGB1BP2](http://www.ncbi.nlm.nih.gov/entrez/query.fcgi?cmd=search&db=gene&term=ITGB1BP2) |  |
| 5.44E-05 | 0.355801 | 152.5805762 | 193.794469 | 0.7873319 | 1010367 | [CSTF3](http://www.ncbi.nlm.nih.gov/entrez/query.fcgi?cmd=search&db=gene&term=CSTF3) | Polyadenylation of mRNA, gene_regulation, transcription |
| 0.0044045 | 0.488743 | 765.3253367 | 994.4263788 | 0.7696149 | 7380653 | [DDX56](http://www.ncbi.nlm.nih.gov/entrez/query.fcgi?cmd=search&db=gene&term=DDX56) | Folate biosynthesis, Starch and sucrose metabolism |
| 0.0017348 | 0.484726 | 122.6174475 | 159.4473702 | 0.7690152 | 4540543 | [SUPT3H](http://www.ncbi.nlm.nih.gov/entrez/query.fcgi?cmd=search&db=gene&term=SUPT3H) | tsonc |
| 0.000514 | 0.407673 | 155.9752613 | 203.1757168 | 0.7676865 | 4280767 | [LARS2](http://www.ncbi.nlm.nih.gov/entrez/query.fcgi?cmd=search&db=gene&term=LARS2) | Aminoacyl-tRNA biosynthesis, Valine, leucine and isoleucine biosynthesis |
| 0.0001008 | 0.355801 | 114.5332548 | 149.4361286 | 0.7664362 | 110349 | [SYMPK](http://www.ncbi.nlm.nih.gov/entrez/query.fcgi?cmd=search&db=gene&term=SYMPK) | Tight junction |
| 0.0023233 | 0.488743 | 789.3420037 | 1039.027317 | 0.7596932 | 6420180 | [TJAP1](http://www.ncbi.nlm.nih.gov/entrez/query.fcgi?cmd=search&db=gene&term=TJAP1) | Tight junction |
| 0.000972 | 0.427352 | 121.8052162 | 160.7686351 | 0.7576429 | 2570307 | [TTLL7](http://www.ncbi.nlm.nih.gov/entrez/query.fcgi?cmd=search&db=gene&term=TTLL7) |  |
| 0.0042086 | 0.488743 | 126.6072816 | 167.4380952 | 0.7561438 | 6220615 | [PSMD11](http://www.ncbi.nlm.nih.gov/entrez/query.fcgi?cmd=search&db=gene&term=PSMD11) | Proteasome |
| 0.0044088 | 0.488743 | 283.5670655 | 375.2266319 | 0.7557221 | 7160025 | [ING2](http://www.ncbi.nlm.nih.gov/entrez/query.fcgi?cmd=search&db=gene&term=ING2) |  |
| 0.0009659 | 0.427352 | 216.0178605 | 286.3103318 | 0.7544885 | 6620402 | [NUDT16](http://www.ncbi.nlm.nih.gov/entrez/query.fcgi?cmd=search&db=gene&term=NUDT16) |  |
| 0.0011226 | 0.45286 | 380.5068919 | 505.7152764 | 0.7524133 | 6660047 | [KIAA0090](http://www.ncbi.nlm.nih.gov/entrez/query.fcgi?cmd=search&db=gene&term=KIAA0090) |  |
| 0.0021115 | 0.488743 | 183.1950346 | 244.114975 | 0.7504457 | 6620176 | [GTPBP8](http://www.ncbi.nlm.nih.gov/entrez/query.fcgi?cmd=search&db=gene&term=GTPBP8) |  |
| 0.0002809 | 0.373332 | 99.8642312 | 133.1206192 | 0.7501785 | 2690228 | [LOC440934](http://www.ncbi.nlm.nih.gov/entrez/query.fcgi?cmd=search&db=gene&term=LOC440934) |  |
| 0.0034275 | 0.488743 | 98.6559947 | 132.3123026 | 0.7456298 | 3180372 | [FAM40B](http://www.ncbi.nlm.nih.gov/entrez/query.fcgi?cmd=search&db=gene&term=FAM40B) |  |
| 0.0041205 | 0.488743 | 543.9140844 | 732.0474344 | 0.7430039 | 2190709 | [DDEFL1](http://www.ncbi.nlm.nih.gov/entrez/query.fcgi?cmd=search&db=gene&term=DDEFL1) |  |
| 0.0038972 | 0.488743 | 1056.273423 | 1426.34943 | 0.7405432 | 20491 | [UBE2F](http://www.ncbi.nlm.nih.gov/entrez/query.fcgi?cmd=search&db=gene&term=UBE2F) |  |
| 0.0039106 | 0.488743 | 1981.05054 | 2677.684757 | 0.7398371 | 7320181 | [COPS7A](http://www.ncbi.nlm.nih.gov/entrez/query.fcgi?cmd=search&db=gene&term=COPS7A) |  |
| 3.67E-05 | 0.355801 | 150.3760183 | 205.689818 | 0.7310815 | 1410470 | [FAM71E1](http://www.ncbi.nlm.nih.gov/entrez/query.fcgi?cmd=search&db=gene&term=FAM71E1) |  |
| 0.0011585 | 0.46094 | 340.1350446 | 465.5241571 | 0.7306496 | 20707 | [COMMD5](http://www.ncbi.nlm.nih.gov/entrez/query.fcgi?cmd=search&db=gene&term=COMMD5) |  |
| 0.0022281 | 0.488743 | 290.792497 | 402.7572618 | 0.7220044 | 2480044 | [MTX2](http://www.ncbi.nlm.nih.gov/entrez/query.fcgi?cmd=search&db=gene&term=MTX2) |  |
| 0.0028139 | 0.488743 | 3645.517089 | 5072.583729 | 0.7186707 | 6020669 | [LOC391811](http://www.ncbi.nlm.nih.gov/entrez/query.fcgi?cmd=search&db=gene&term=LOC391811) |  |
| 0.0006505 | 0.427352 | 771.0561122 | 1078.305777 | 0.7150626 | 520196 | [GNL2](http://www.ncbi.nlm.nih.gov/entrez/query.fcgi?cmd=search&db=gene&term=GNL2) |  |
| 0.0026616 | 0.488743 | 853.0878875 | 1202.445982 | 0.7094605 | 3370487 | [HNRNPAB](http://www.ncbi.nlm.nih.gov/entrez/query.fcgi?cmd=search&db=gene&term=HNRNPAB) |  |
| 0.004152 | 0.488743 | 118.0905414 | 167.6143856 | 0.704537 | 2940095 | [SLC36A1](http://www.ncbi.nlm.nih.gov/entrez/query.fcgi?cmd=search&db=gene&term=SLC36A1) |  |
| 0.0021492 | 0.488743 | 139.2980001 | 199.7064314 | 0.6975138 | 4590347 | [ZNF784](http://www.ncbi.nlm.nih.gov/entrez/query.fcgi?cmd=search&db=gene&term=ZNF784) |  |
| 0.0024991 | 0.488743 | 274.0957246 | 393.4080702 | 0.6967212 | 240487 | [SH3BP5L](http://www.ncbi.nlm.nih.gov/entrez/query.fcgi?cmd=search&db=gene&term=SH3BP5L) |  |
| 0.0028228 | 0.488743 | 958.6614856 | 1390.638133 | 0.689368 | 1850482 | [TNK2](http://www.ncbi.nlm.nih.gov/entrez/query.fcgi?cmd=search&db=gene&term=TNK2) |  |
| 0.0047888 | 0.488743 | 150.9549782 | 219.4740526 | 0.6878033 | 6590324 | [PTDSS2](http://www.ncbi.nlm.nih.gov/entrez/query.fcgi?cmd=search&db=gene&term=PTDSS2) |  |
| 0.0011951 | 0.466249 | 189.898634 | 279.3651082 | 0.6797507 | 1010068 | [FKBP2](http://www.ncbi.nlm.nih.gov/entrez/query.fcgi?cmd=search&db=gene&term=FKBP2) |  |
| 0.0049101 | 0.488743 | 148.3446131 | 218.2746857 | 0.6796235 | 1770243 | [IGF2BP3](http://www.ncbi.nlm.nih.gov/entrez/query.fcgi?cmd=search&db=gene&term=IGF2BP3) |  |
| 0.0041798 | 0.488743 | 285.2610607 | 422.3596449 | 0.6753985 | 4890056 | [LOC283951](http://www.ncbi.nlm.nih.gov/entrez/query.fcgi?cmd=search&db=gene&term=LOC283951) |  |
| 0.0031629 | 0.488743 | 209.2524873 | 318.7831759 | 0.6564101 | 6380048 | [RNF217](http://www.ncbi.nlm.nih.gov/entrez/query.fcgi?cmd=search&db=gene&term=RNF217) |  |
| 0.0012968 | 0.468254 | 214.9870579 | 329.0147047 | 0.6534269 | 3060543 | [MORC2](http://www.ncbi.nlm.nih.gov/entrez/query.fcgi?cmd=search&db=gene&term=MORC2) |  |
| 0.0002656 | 0.373332 | 142.9540433 | 218.8259098 | 0.6532775 | 780475 | [PFDN6](http://www.ncbi.nlm.nih.gov/entrez/query.fcgi?cmd=search&db=gene&term=PFDN6) |  |
| 0.0046179 | 0.488743 | 231.8275481 | 356.5030592 | 0.6502821 | 6270450 | [GCN1L1](http://www.ncbi.nlm.nih.gov/entrez/query.fcgi?cmd=search&db=gene&term=GCN1L1) |  |
| 0.0013341 | 0.468254 | 122.1941022 | 192.9347563 | 0.6333442 | 2260253 | [USP16](http://www.ncbi.nlm.nih.gov/entrez/query.fcgi?cmd=search&db=gene&term=USP16) |  |
| 0.0001103 | 0.355801 | 721.7491528 | 1142.123393 | 0.6319362 | 70019 | [SFRS14](http://www.ncbi.nlm.nih.gov/entrez/query.fcgi?cmd=search&db=gene&term=SFRS14) |  |
| 0.0023187 | 0.488743 | 162.9066921 | 257.9397815 | 0.6315687 | 4040722 | [JPH2](http://www.ncbi.nlm.nih.gov/entrez/query.fcgi?cmd=search&db=gene&term=JPH2) |  |
| 0.0040436 | 0.488743 | 414.9839737 | 670.9714045 | 0.6184824 | 6330647 | [RPS7](http://www.ncbi.nlm.nih.gov/entrez/query.fcgi?cmd=search&db=gene&term=RPS7) | Ribosome |
| 0.003547 | 0.488743 | 146.8371197 | 237.9371802 | 0.6171256 | 4850184 | [CASZ1](http://www.ncbi.nlm.nih.gov/entrez/query.fcgi?cmd=search&db=gene&term=CASZ1) |  |
| 0.0040187 | 0.488743 | 618.3582269 | 1017.94515 | 0.6074573 | 4050156 | [ERBB2](http://www.ncbi.nlm.nih.gov/entrez/query.fcgi?cmd=search&db=gene&term=ERBB2) | immunology, signal_transduction, tsonc |
| 0.0008556 | 0.427352 | 222.5887099 | 367.5596356 | 0.6055853 | 3460068 | [TRIM54](http://www.ncbi.nlm.nih.gov/entrez/query.fcgi?cmd=search&db=gene&term=TRIM54) |  |
| 0.003961 | 0.488743 | 129.7497433 | 216.867185 | 0.5982913 | 6580386 | [OPA3](http://www.ncbi.nlm.nih.gov/entrez/query.fcgi?cmd=search&db=gene&term=OPA3) | immunology |
| 0.0043074 | 0.488743 | 804.2914714 | 1351.887644 | 0.5949396 | 1450377 | [BLOC1S1](http://www.ncbi.nlm.nih.gov/entrez/query.fcgi?cmd=search&db=gene&term=BLOC1S1) |  |
| 0.0019593 | 0.486392 | 114.9840693 | 194.443242 | 0.5913503 | 270520 | [DMD](http://www.ncbi.nlm.nih.gov/entrez/query.fcgi?cmd=search&db=gene&term=DMD) | Agrin in Postsynaptic Differentiation, immunology |
| 0.0043014 | 0.488743 | 229.0568566 | 388.3126816 | 0.5898774 | 1240309 | [FOXRED1](http://www.ncbi.nlm.nih.gov/entrez/query.fcgi?cmd=search&db=gene&term=FOXRED1) |  |
| 0.002801 | 0.488743 | 120.3725122 | 204.9694521 | 0.5872705 | 780326 |  |  |
| 0.003378 | 0.488743 | 1320.832752 | 2251.313449 | 0.5866943 | 4180577 | [FXR1](http://www.ncbi.nlm.nih.gov/entrez/query.fcgi?cmd=search&db=gene&term=FXR1) | immunology |
| 0.0007338 | 0.427352 | 211.1584302 | 362.5329274 | 0.5824531 | 4480288 | [ISG20L1](http://www.ncbi.nlm.nih.gov/entrez/query.fcgi?cmd=search&db=gene&term=ISG20L1) |  |
| 0.0019177 | 0.484726 | 558.0987231 | 962.660913 | 0.5797459 | 5090368 | [HSPA4](http://www.ncbi.nlm.nih.gov/entrez/query.fcgi?cmd=search&db=gene&term=HSPA4) |  |
| 0.0004648 | 0.407673 | 174.1482762 | 301.5317272 | 0.5775454 | 2070669 | [CDC42EP3](http://www.ncbi.nlm.nih.gov/entrez/query.fcgi?cmd=search&db=gene&term=CDC42EP3) |  |
| 0.0029189 | 0.488743 | 402.9669339 | 717.0618771 | 0.5619695 | 1070053 | [SFRS14](http://www.ncbi.nlm.nih.gov/entrez/query.fcgi?cmd=search&db=gene&term=SFRS14) |  |
| 0.0024365 | 0.488743 | 163.3587568 | 292.9198362 | 0.557691 | 3440356 | [TAF15](http://www.ncbi.nlm.nih.gov/entrez/query.fcgi?cmd=search&db=gene&term=TAF15) |  |
| 0.0014891 | 0.474223 | 182.540503 | 328.0182963 | 0.5564949 | 2600288 | [GOSR2](http://www.ncbi.nlm.nih.gov/entrez/query.fcgi?cmd=search&db=gene&term=GOSR2) | SNARE interactions in vesicular transport |
| 0.0024679 | 0.488743 | 225.5805208 | 408.4939697 | 0.5522248 | 7040170 | [NEURL2](http://www.ncbi.nlm.nih.gov/entrez/query.fcgi?cmd=search&db=gene&term=NEURL2) |  |
| 0.0028878 | 0.488743 | 501.8642115 | 912.1242111 | 0.5502148 | 290468 | [EIF2C2](http://www.ncbi.nlm.nih.gov/entrez/query.fcgi?cmd=search&db=gene&term=EIF2C2) | Dicer Pathway |
| 0.0026558 | 0.488743 | 271.5497334 | 497.4108178 | 0.5459265 | 1990491 | [AK2](http://www.ncbi.nlm.nih.gov/entrez/query.fcgi?cmd=search&db=gene&term=AK2) | Purine metabolism, cell_cycle, cell_signaling, signal_transduction |
| 0.0013903 | 0.47141 | 232.126514 | 435.9133106 | 0.5325061 | 2450167 | [RPL29](http://www.ncbi.nlm.nih.gov/entrez/query.fcgi?cmd=search&db=gene&term=RPL29) | Ribosome |
| 0.0042911 | 0.488743 | 239.7836271 | 451.7158989 | 0.5308284 | 4780433 | [RPL29](http://www.ncbi.nlm.nih.gov/entrez/query.fcgi?cmd=search&db=gene&term=RPL29) | Ribosome |
| 0.0036568 | 0.488743 | 215.3771244 | 415.2171652 | 0.5187096 | 5360020 | [PPP2R3B](http://www.ncbi.nlm.nih.gov/entrez/query.fcgi?cmd=search&db=gene&term=PPP2R3B) | Tight junction |
| 0.0022436 | 0.488743 | 170.9287597 | 336.3066196 | 0.5082527 | 5720192 | [FANCE](http://www.ncbi.nlm.nih.gov/entrez/query.fcgi?cmd=search&db=gene&term=FANCE) | BRCA1-dependent Ub-ligase activity, Role of BRCA1, BRCA2 and ATR in Cancer Susceptibility, immunology |
| 0.0007593 | 0.427352 | 259.1442376 | 528.7220114 | 0.4901332 | 3940066 | [KLHL30](http://www.ncbi.nlm.nih.gov/entrez/query.fcgi?cmd=search&db=gene&term=KLHL30) |  |
| 0.0002816 | 0.373332 | 143.0477332 | 294.2125192 | 0.4862055 | 2630053 | [COL4A3BP](http://www.ncbi.nlm.nih.gov/entrez/query.fcgi?cmd=search&db=gene&term=COL4A3BP) |  |
| 0.0021749 | 0.488743 | 218.0540073 | 449.3020133 | 0.4853172 | 2060037 | [ZBTB43](http://www.ncbi.nlm.nih.gov/entrez/query.fcgi?cmd=search&db=gene&term=ZBTB43) |  |
| 0.0018625 | 0.484726 | 764.0866125 | 1613.965122 | 0.473422 | 7650639 | [CHRNB1](http://www.ncbi.nlm.nih.gov/entrez/query.fcgi?cmd=search&db=gene&term=CHRNB1) | Role of nicotinic acetylcholine receptors in the regulation of apoptosis |
| 0.0005619 | 0.40801 | 164.8893102 | 350.388911 | 0.4705894 | 380279 | [SPTB](http://www.ncbi.nlm.nih.gov/entrez/query.fcgi?cmd=search&db=gene&term=SPTB) | cell_signaling, metastasis |
| 0.0036521 | 0.488743 | 233.2215149 | 510.797995 | 0.4565827 | 5360382 | [SPTB](http://www.ncbi.nlm.nih.gov/entrez/query.fcgi?cmd=search&db=gene&term=SPTB) | cell_signaling, metastasis |
| 0.0021595 | 0.488743 | 224.0136053 | 498.1373533 | 0.4497025 | 1580181 | [A2BP1](http://www.ncbi.nlm.nih.gov/entrez/query.fcgi?cmd=search&db=gene&term=A2BP1) |  |
| 0.0003534 | 0.373332 | 223.6060498 | 504.1952911 | 0.443491 | 5220554 | [A2BP1](http://www.ncbi.nlm.nih.gov/entrez/query.fcgi?cmd=search&db=gene&term=A2BP1) |  |
| 0.0044255 | 0.488743 | 115.4681301 | 288.8118002 | 0.3998041 | 6110017 | [C20orf26](http://www.ncbi.nlm.nih.gov/entrez/query.fcgi?cmd=search&db=gene&term=C20orf26) |  |
| 0.000175 | 0.355801 | 218.3722158 | 588.5756217 | 0.3710181 | 7050064 | [RAMP1](http://www.ncbi.nlm.nih.gov/entrez/query.fcgi?cmd=search&db=gene&term=RAMP1) |  |
| 0.0025407 | 0.488743 | 728.5980484 | 2089.60438 | 0.3486775 | 2940746 | [NNMT](http://www.ncbi.nlm.nih.gov/entrez/query.fcgi?cmd=search&db=gene&term=NNMT) | Nicotinate and nicotinamide metabolism, immunology |
| 0.0047472 | 0.488743 | 219.6068716 | 666.1400654 | 0.3296707 | 6510377 | [TNFRSF12A](http://www.ncbi.nlm.nih.gov/entrez/query.fcgi?cmd=search&db=gene&term=TNFRSF12A) | Cytokine-cytokine receptor interaction |
|  |  |  |  |  |  |  |  |
|  |  |  |  |  |  |  |  |
| **Genes which did not meet the filtering criteria but are cited in text, figure or tables** | | | |  |  |  |  |
| 0.0056009 | 0.488743 | 1332.148328 | 587.5810919 | 2.2671736 | 5220767 | [FLNB](http://www.ncbi.nlm.nih.gov/entrez/query.fcgi?cmd=search&db=gene&term=FLNB) | Focal adhesion, MAPK signaling pathway, immunology |
| 0.0331401 | 0.571303 | 11612.75757 | 5923.052487 | 1.9606035 | 5870138 | [VWF](http://www.ncbi.nlm.nih.gov/entrez/query.fcgi?cmd=search&db=gene&term=VWF) | Cell Communication, Complement and coagulation cascades, ECM-receptor interaction, Focal adhesion, angiogenesis, immunology, misc |
| 0.0050555 | 0.488743 | 3222.874126 | 1492.756855 | 2.1590081 | 620255 | [FZD4](http://www.ncbi.nlm.nih.gov/entrez/query.fcgi?cmd=search&db=gene&term=FZD4) | Colorectal cancer, Wnt signaling pathway, development |
| 0.054386 | 0.606814 | 645.0211782 | 164.7895891 | 3.9142107 | 3840458 | [LEP](http://www.ncbi.nlm.nih.gov/entrez/query.fcgi?cmd=search&db=gene&term=LEP) | Reversal of Insulin Resistance by Leptin, Adipocytokine signaling pathway, Cytokine-cytokine receptor interaction, Jak-STAT signaling pathway, Neuroactive ligand-receptor interaction, immunology |
| 0.0081035 | 0.489538 | 610.6256701 | 358.5856704 | 1.7028725 | 6290725 | [FYN](http://www.ncbi.nlm.nih.gov/entrez/query.fcgi?cmd=search&db=gene&term=FYN) | Bioactive Peptide Induced Signaling Pathway, Eph Kinases and ephrins support platelet aggregation, Erk and PI-3 Kinase Are Necessary for Collagen Binding in Corneal Epithelia, IL-7 Signal Transduction, Integrin Signaling Pathway, Lck and Fyn tyrosine kinases in initiation of TCR Activation, Reelin Signaling Pathway, T Cell Receptor Signaling Pathway, TSP-1 Induced Apoptosis in Microvascular Endothelial Cell , Adherens junction, Axon guidance, Fc epsilon RI signaling pathway, Focal adhesion, NA, Natural killer cell mediated cytotoxicity, T cell receptor signaling pathway, signal_transduction, tsonc |
| 0.0327355 | 0.571303 | 483.3657913 | 211.3823709 | 2.2866892 | 1170671 | [CD3D](http://www.ncbi.nlm.nih.gov/entrez/query.fcgi?cmd=search&db=gene&term=CD3D) | Activation of Csk by cAMP-dependent Protein Kinase Inhibits Signaling through the T Cell Receptor, CTL mediated immune response against target cells , HIV Induced T Cell Apoptosis, IL 17 Signaling Pathway, IL12 and Stat4 Dependent Signaling Pathway in Th1 Development, Lck and Fyn tyrosine kinases in initiation of TCR Activation, Role of Tob in T-cell activation, T Cell Receptor and CD3 Complex, T Cell Receptor Signaling Pathway, T Cytotoxic Cell Surface Molecules, T Helper Cell Surface Molecules, The Co-Stimulatory Signal During T-cell Activation, Hematopoietic cell lineage, T cell receptor signaling pathway |
| 0.1015613 | 0.657766 | 160.7012316 | 107.8380914 | 1.4902084 | 1780600 | [CD3E](http://www.ncbi.nlm.nih.gov/entrez/query.fcgi?cmd=search&db=gene&term=CD3E) | Activation of Csk by cAMP-dependent Protein Kinase Inhibits Signaling through the T Cell Receptor, CTL mediated immune response against target cells , HIV Induced T Cell Apoptosis, IL 17 Signaling Pathway, IL12 and Stat4 Dependent Signaling Pathway in Th1 Development, Lck and Fyn tyrosine kinases in initiation of TCR Activation, Role of Tob in T-cell activation, T Cell Receptor and CD3 Complex, T Cell Receptor Signaling Pathway, T Cytotoxic Cell Surface Molecules, T Helper Cell Surface Molecules, The Co-Stimulatory Signal During T-cell Activation, Hematopoietic cell lineage, T cell receptor signaling pathway, immunology |
| 0.089578 | 0.644376 | 2482.092844 | 2047.109166 | 1.2124868 | 7400626 | [NFKB1](http://www.ncbi.nlm.nih.gov/entrez/query.fcgi?cmd=search&db=gene&term=NFKB1) | Acetylation and Deacetylation of RelA in The Nucleus, Activation of PKC through G protein coupled receptor, AKT Signaling Pathway, ATM Signaling Pathway, Bone Remodelling, Cadmium induces DNA synthesis and proliferation in macrophages, CD40L Signaling Pathway, Ceramide Signaling Pathway, Chaperones modulate interferon Signaling Pathway, Corticosteroids and cardioprotection, CXCR4 Signaling Pathway, Double Stranded RNA Induced Gene Expression, Erythropoietin mediated neuroprotection through NF-kB, fMLP induced chemokine gene expression in HMC-1 cells, Free Radical Induced Apoptosis, HIV-I Nef: negative effector of Fas and TNF, Human Cytomegalovirus and Map Kinase Pathways, Inactivation of Gsk3 by AKT causes accumulation of b-catenin in Alveolar Macrophages, Induction of apoptosis through DR3 and DR4/5 Death Receptors , Influence of Ras and Rho proteins on G1 to S Transition, Keratinocyte Differentiation, MAPKinase Signaling Pathway, Neuropeptides VIP and PACAP inhibit the apoptosis of a ... |
| 0.2193556 | 0.746636 | 147.8948887 | 126.8861538 | 1.1655715 | 4780678 | [ZAP70](http://www.ncbi.nlm.nih.gov/entrez/query.fcgi?cmd=search&db=gene&term=ZAP70) | Activation of Csk by cAMP-dependent Protein Kinase Inhibits Signaling through the T Cell Receptor, Lck and Fyn tyrosine kinases in initiation of TCR Activation, T Cell Receptor Signaling Pathway, NA, Natural killer cell mediated cytotoxicity, T cell receptor signaling pathway, cell_cycle, cell_signaling, signal_transduction |
