# Supplementary material for: Different Molecular Signatures in Magnetic Resonance Imaging-Staged Facioscapulohumeral Muscular Dystrophy Muscles
Source: PLoS One. 2012 Jun 13;7(6):e38779. doi: 10.1371/journal.pone.0038779 (PMC3374833; doi:10.1371/journal.pone.0038779)
Supplement: Table S4 — BioCarta, Gene Ontology, and KEGG gene set expression comparison of T2-STIR + FSHD vs. Inflammatory myopathy muscles. (DOC) [file pone.0038779.s008.doc]

**Table S4: BioCarta, Gene Ontology, KEGG Gene Set Expression Comparison FSHD T2-STIR + vs. Inflammatory myopathies**

| BioCarta Gene Set Expression Comparison FSHD T2-STIR + vs. Inflammatory myopathies | | | | | | | |
| --- | --- | --- | --- | --- | --- | --- | --- |
|  | **Biocarta Pathway** | **Pathway description** | **Number of genes** | **LS permutation p-value** | **KS permutation p-value** | **Efron-Tibshirani's GSA test p-value** | **Goeman's global test p-value** |
| 1 | h_argininecPathway | [Catabolic Pathways for Arginine , Histidine, Glutamate, Glutamine, and Proline](http://cgap.nci.nih.gov/Pathways/BioCarta/h_argininecPathway) | [5](../../../../C:%5CDocuments%20and%20Settings%5CAdministrator%5CDesktop%5CDystro2009_all%20-Project%5COutput%5Cbuona%20fshd%20inf%20VS%20inf%20biocarta%20PathwayClassComparison%5CGeneSetGenesTable1.html#h_argininecPathway) | 0.0003 | 0.00536 | 0.005 (-) | 0.0028041 |
| 2 | h_reckPathway | [Inhibition of Matrix Metalloproteinases](http://cgap.nci.nih.gov/Pathways/BioCarta/h_reckPathway) | [8](../../../../C:%5CDocuments%20and%20Settings%5CAdministrator%5CDesktop%5CDystro2009_all%20-Project%5COutput%5Cbuona%20fshd%20inf%20VS%20inf%20biocarta%20PathwayClassComparison%5CGeneSetGenesTable1.html#h_reckPathway) | 0.00037 | 0.00642 | 0.01 (-) | 0.0013876 |
| 3 | h_tcrPathway | [T Cell Receptor Signaling Pathway](http://cgap.nci.nih.gov/Pathways/BioCarta/h_tcrPathway) | [29](../../../../C:%5CDocuments%20and%20Settings%5CAdministrator%5CDesktop%5CDystro2009_all%20-Project%5COutput%5Cbuona%20fshd%20inf%20VS%20inf%20biocarta%20PathwayClassComparison%5CGeneSetGenesTable1.html#h_tcrPathway) | 0.00064 | 0.00192 | 0.065 (-) | 0.0055856 |
| 4 | h_agrPathway | [Agrin in Postsynaptic Differentiation](http://cgap.nci.nih.gov/Pathways/BioCarta/h_agrPathway) | [24](../../../../C:%5CDocuments%20and%20Settings%5CAdministrator%5CDesktop%5CDystro2009_all%20-Project%5COutput%5Cbuona%20fshd%20inf%20VS%20inf%20biocarta%20PathwayClassComparison%5CGeneSetGenesTable1.html#h_agrPathway) | 0.00191 | 0.00782 | 0.11 (+) | 0.0110449 |
| 5 | h_cblPathway | [CBL mediated ligand-induced downregulation of EGF receptors](http://cgap.nci.nih.gov/Pathways/BioCarta/h_cblPathway) | [10](../../../../C:%5CDocuments%20and%20Settings%5CAdministrator%5CDesktop%5CDystro2009_all%20-Project%5COutput%5Cbuona%20fshd%20inf%20VS%20inf%20biocarta%20PathwayClassComparison%5CGeneSetGenesTable1.html#h_cblPathway) | 0.00227 | 0.0001 | < 0.005 (-) | 0.0086916 |
| 6 | h_igf1Pathway | [IGF-1 Signaling Pathway](http://cgap.nci.nih.gov/Pathways/BioCarta/h_igf1Pathway) | [15](../../../../C:%5CDocuments%20and%20Settings%5CAdministrator%5CDesktop%5CDystro2009_all%20-Project%5COutput%5Cbuona%20fshd%20inf%20VS%20inf%20biocarta%20PathwayClassComparison%5CGeneSetGenesTable1.html#h_igf1Pathway) | 0.00315 | 0.04727 | 0.02 (+) | 0.0032192 |
| 7 | h_vobesityPathway | [Visceral Fat Deposits and the Metabolic Syndrome](http://cgap.nci.nih.gov/Pathways/BioCarta/h_vobesityPathway) | [7](../../../../C:%5CDocuments%20and%20Settings%5CAdministrator%5CDesktop%5CDystro2009_all%20-Project%5COutput%5Cbuona%20fshd%20inf%20VS%20inf%20biocarta%20PathwayClassComparison%5CGeneSetGenesTable1.html#h_vobesityPathway) | 0.0064 | 0.03726 | 0.04 (-) | 0.0168046 |
| 8 | h_longevityPathway | [The IGF-1 Receptor and Longevity](http://cgap.nci.nih.gov/Pathways/BioCarta/h_longevityPathway) | [10](../../../../C:%5CDocuments%20and%20Settings%5CAdministrator%5CDesktop%5CDystro2009_all%20-Project%5COutput%5Cbuona%20fshd%20inf%20VS%20inf%20biocarta%20PathwayClassComparison%5CGeneSetGenesTable1.html#h_longevityPathway) | 0.00732 | 0.15027 | 0.15 (-) | 0.0124746 |
| 9 | h_dicerPathway | [Dicer Pathway](http://cgap.nci.nih.gov/Pathways/BioCarta/h_dicerPathway) | [5](../../../../C:%5CDocuments%20and%20Settings%5CAdministrator%5CDesktop%5CDystro2009_all%20-Project%5COutput%5Cbuona%20fshd%20inf%20VS%20inf%20biocarta%20PathwayClassComparison%5CGeneSetGenesTable1.html#h_dicerPathway) | 0.00851 | 0.15409 | 0.035 (+) | 0.0019499 |
| 10 | h_spryPathway | [Sprouty regulation of tyrosine kinase signals](http://cgap.nci.nih.gov/Pathways/BioCarta/h_spryPathway) | [15](../../../../C:%5CDocuments%20and%20Settings%5CAdministrator%5CDesktop%5CDystro2009_all%20-Project%5COutput%5Cbuona%20fshd%20inf%20VS%20inf%20biocarta%20PathwayClassComparison%5CGeneSetGenesTable1.html#h_spryPathway) | 0.0094 | 0.02249 | 0.095 (-) | 0.0072711 |
| 11 | h_At1rPathway | [Angiotensin II mediated activation of JNK Pathway via Pyk2 dependent signaling](http://cgap.nci.nih.gov/Pathways/BioCarta/h_At1rPathway) | [24](../../../../C:%5CDocuments%20and%20Settings%5CAdministrator%5CDesktop%5CDystro2009_all%20-Project%5COutput%5Cbuona%20fshd%20inf%20VS%20inf%20biocarta%20PathwayClassComparison%5CGeneSetGenesTable1.html#h_At1rPathway) | 0.01431 | 0.14143 | 0.075 (+) | 0.003844 |
| 12 | h_p53Pathway | [p53 Signaling Pathway](http://cgap.nci.nih.gov/Pathways/BioCarta/h_p53Pathway) | [14](../../../../C:%5CDocuments%20and%20Settings%5CAdministrator%5CDesktop%5CDystro2009_all%20-Project%5COutput%5Cbuona%20fshd%20inf%20VS%20inf%20biocarta%20PathwayClassComparison%5CGeneSetGenesTable1.html#h_p53Pathway) | 0.01647 | 0.05775 | < 0.005 (-) | 0.0013659 |
| 13 | h_keratinocytePathway | [Keratinocyte Differentiation](http://cgap.nci.nih.gov/Pathways/BioCarta/h_keratinocytePathway) | [32](../../../../C:%5CDocuments%20and%20Settings%5CAdministrator%5CDesktop%5CDystro2009_all%20-Project%5COutput%5Cbuona%20fshd%20inf%20VS%20inf%20biocarta%20PathwayClassComparison%5CGeneSetGenesTable1.html#h_keratinocytePathway) | 0.01809 | 0.17508 | 0.08 (-) | 0.0034776 |
| 14 | h_achPathway | [Role of nicotinic acetylcholine receptors in the regulation of apoptosis](http://cgap.nci.nih.gov/Pathways/BioCarta/h_achPathway) | [13](../../../../C:%5CDocuments%20and%20Settings%5CAdministrator%5CDesktop%5CDystro2009_all%20-Project%5COutput%5Cbuona%20fshd%20inf%20VS%20inf%20biocarta%20PathwayClassComparison%5CGeneSetGenesTable1.html#h_achPathway) | 0.02008 | 0.25661 | 0.005 (+) | 0.0159907 |
| 15 | h_tcraPathway | [Lck and Fyn tyrosine kinases in initiation of TCR Activation](http://cgap.nci.nih.gov/Pathways/BioCarta/h_tcraPathway) | [10](../../../../C:%5CDocuments%20and%20Settings%5CAdministrator%5CDesktop%5CDystro2009_all%20-Project%5COutput%5Cbuona%20fshd%20inf%20VS%20inf%20biocarta%20PathwayClassComparison%5CGeneSetGenesTable1.html#h_tcraPathway) | 0.02138 | 0.00517 | 0.145 (-) | 0.0550127 |
| 16 | h_il2Pathway | [IL 2 signaling pathway](http://cgap.nci.nih.gov/Pathways/BioCarta/h_il2Pathway) | [16](../../../../C:%5CDocuments%20and%20Settings%5CAdministrator%5CDesktop%5CDystro2009_all%20-Project%5COutput%5Cbuona%20fshd%20inf%20VS%20inf%20biocarta%20PathwayClassComparison%5CGeneSetGenesTable1.html#h_il2Pathway) | 0.02268 | 0.06169 | 0.015 (+) | 0.00373 |
| 17 | h_thelperPathway | [T Helper Cell Surface Molecules](http://cgap.nci.nih.gov/Pathways/BioCarta/h_thelperPathway) | [9](../../../../C:%5CDocuments%20and%20Settings%5CAdministrator%5CDesktop%5CDystro2009_all%20-Project%5COutput%5Cbuona%20fshd%20inf%20VS%20inf%20biocarta%20PathwayClassComparison%5CGeneSetGenesTable1.html#h_thelperPathway) | 0.02421 | 0.00019 | 0.145 (-) | 0.0231081 |
| 18 | h_etsPathway | [METS affect on Macrophage Differentiation](http://cgap.nci.nih.gov/Pathways/BioCarta/h_etsPathway) | [12](../../../../C:%5CDocuments%20and%20Settings%5CAdministrator%5CDesktop%5CDystro2009_all%20-Project%5COutput%5Cbuona%20fshd%20inf%20VS%20inf%20biocarta%20PathwayClassComparison%5CGeneSetGenesTable1.html#h_etsPathway) | 0.02457 | 0.16562 | 0.095 (-) | 0.0052349 |
| 19 | h_bcrPathway | [BCR Signaling Pathway](http://cgap.nci.nih.gov/Pathways/BioCarta/h_bcrPathway) | [21](../../../../C:%5CDocuments%20and%20Settings%5CAdministrator%5CDesktop%5CDystro2009_all%20-Project%5COutput%5Cbuona%20fshd%20inf%20VS%20inf%20biocarta%20PathwayClassComparison%5CGeneSetGenesTable1.html#h_bcrPathway) | 0.02463 | 0.12331 | 0.04 (+) | 0.0099563 |
| 20 | h_tcapoptosisPathway | [HIV Induced T Cell Apoptosis](http://cgap.nci.nih.gov/Pathways/BioCarta/h_tcapoptosisPathway) | [7](../../../../C:%5CDocuments%20and%20Settings%5CAdministrator%5CDesktop%5CDystro2009_all%20-Project%5COutput%5Cbuona%20fshd%20inf%20VS%20inf%20biocarta%20PathwayClassComparison%5CGeneSetGenesTable1.html#h_tcapoptosisPathway) | 0.02821 | 0.00207 | 0.125 (-) | 0.0417213 |
| 21 | h_biopeptidesPathway | [Bioactive Peptide Induced Signaling Pathway](http://cgap.nci.nih.gov/Pathways/BioCarta/h_biopeptidesPathway) | [26](../../../../C:%5CDocuments%20and%20Settings%5CAdministrator%5CDesktop%5CDystro2009_all%20-Project%5COutput%5Cbuona%20fshd%20inf%20VS%20inf%20biocarta%20PathwayClassComparison%5CGeneSetGenesTable1.html#h_biopeptidesPathway) | 0.03176 | 0.07459 | 0.225 (+) | 0.0050277 |
| 22 | h_ghrelinPathway | [Ghrelin: Regulation of Food Intake and Energy Homeostasis](http://cgap.nci.nih.gov/Pathways/BioCarta/h_ghrelinPathway) | [8](../../../../C:%5CDocuments%20and%20Settings%5CAdministrator%5CDesktop%5CDystro2009_all%20-Project%5COutput%5Cbuona%20fshd%20inf%20VS%20inf%20biocarta%20PathwayClassComparison%5CGeneSetGenesTable1.html#h_ghrelinPathway) | 0.03345 | 0.07987 | 0.005 (-) | 0.0035137 |
| 23 | h_insulinPathway | [Insulin Signaling Pathway](http://cgap.nci.nih.gov/Pathways/BioCarta/h_insulinPathway) | [14](../../../../C:%5CDocuments%20and%20Settings%5CAdministrator%5CDesktop%5CDystro2009_all%20-Project%5COutput%5Cbuona%20fshd%20inf%20VS%20inf%20biocarta%20PathwayClassComparison%5CGeneSetGenesTable1.html#h_insulinPathway) | 0.03385 | 0.08739 | 0.025 (+) | 0.0046151 |
| 24 | h_tcytotoxicPathway | [T Cytotoxic Cell Surface Molecules](http://cgap.nci.nih.gov/Pathways/BioCarta/h_tcytotoxicPathway) | [9](../../../../C:%5CDocuments%20and%20Settings%5CAdministrator%5CDesktop%5CDystro2009_all%20-Project%5COutput%5Cbuona%20fshd%20inf%20VS%20inf%20biocarta%20PathwayClassComparison%5CGeneSetGenesTable1.html#h_tcytotoxicPathway) | 0.03564 | 0.00224 | 0.125 (-) | 0.0406861 |
| 25 | h_lymphocytePathway | [Adhesion Molecules on Lymphocyte](http://cgap.nci.nih.gov/Pathways/BioCarta/h_lymphocytePathway) | [7](../../../../C:%5CDocuments%20and%20Settings%5CAdministrator%5CDesktop%5CDystro2009_all%20-Project%5COutput%5Cbuona%20fshd%20inf%20VS%20inf%20biocarta%20PathwayClassComparison%5CGeneSetGenesTable1.html#h_lymphocytePathway) | 0.03892 | 0.21511 | 0.015 (-) | 0.0063339 |
| 26 | h_epoPathway | [EPO Signaling Pathway](http://cgap.nci.nih.gov/Pathways/BioCarta/h_epoPathway) | [15](../../../../C:%5CDocuments%20and%20Settings%5CAdministrator%5CDesktop%5CDystro2009_all%20-Project%5COutput%5Cbuona%20fshd%20inf%20VS%20inf%20biocarta%20PathwayClassComparison%5CGeneSetGenesTable1.html#h_epoPathway) | 0.03893 | 0.11136 | 0.02 (+) | 0.00744 |
| 27 | h_hdacPathway | [Control of skeletal myogenesis by HDAC & calcium/calmodulin-dependent kinase (CaMK)](http://cgap.nci.nih.gov/Pathways/BioCarta/h_hdacPathway) | [15](../../../../C:%5CDocuments%20and%20Settings%5CAdministrator%5CDesktop%5CDystro2009_all%20-Project%5COutput%5Cbuona%20fshd%20inf%20VS%20inf%20biocarta%20PathwayClassComparison%5CGeneSetGenesTable1.html#h_hdacPathway) | 0.04052 | 0.2147 | 0.085 (+) | 0.0030131 |
| 28 | h_integrinPathway | [Integrin Signaling Pathway](http://cgap.nci.nih.gov/Pathways/BioCarta/h_integrinPathway) | [32](../../../../C:%5CDocuments%20and%20Settings%5CAdministrator%5CDesktop%5CDystro2009_all%20-Project%5COutput%5Cbuona%20fshd%20inf%20VS%20inf%20biocarta%20PathwayClassComparison%5CGeneSetGenesTable1.html#h_integrinPathway) | 0.04054 | 0.12974 | 0.04 (+) | 0.0029957 |
| 29 | h_nfatPathway | [NFAT and Hypertrophy of the heart (Transcription in the broken heart)](http://cgap.nci.nih.gov/Pathways/BioCarta/h_nfatPathway) | [28](../../../../C:%5CDocuments%20and%20Settings%5CAdministrator%5CDesktop%5CDystro2009_all%20-Project%5COutput%5Cbuona%20fshd%20inf%20VS%20inf%20biocarta%20PathwayClassComparison%5CGeneSetGenesTable1.html#h_nfatPathway) | 0.05173 | 0.03774 | 0.03 (+) | 0.0073224 |
| 30 | h_MITRPathway | [Signal Dependent Regulation of Myogenesis by Corepressor MITR](http://cgap.nci.nih.gov/Pathways/BioCarta/h_MITRPathway) | [5](../../../../C:%5CDocuments%20and%20Settings%5CAdministrator%5CDesktop%5CDystro2009_all%20-Project%5COutput%5Cbuona%20fshd%20inf%20VS%20inf%20biocarta%20PathwayClassComparison%5CGeneSetGenesTable1.html#h_MITRPathway) | 0.0551 | 0.16708 | 0.185 (+) | 0.0032562 |
| 31 | h_her2Pathway | [Role of ERBB2 in Signal Transduction and Oncology](http://cgap.nci.nih.gov/Pathways/BioCarta/h_her2Pathway) | [19](../../../../C:%5CDocuments%20and%20Settings%5CAdministrator%5CDesktop%5CDystro2009_all%20-Project%5COutput%5Cbuona%20fshd%20inf%20VS%20inf%20biocarta%20PathwayClassComparison%5CGeneSetGenesTable1.html#h_her2Pathway) | 0.05939 | 0.15383 | 0.14 (+) | 0.0069471 |
| 32 | h_reelinPathway | [Reelin Signaling Pathway](http://cgap.nci.nih.gov/Pathways/BioCarta/h_reelinPathway) | [5](../../../../C:%5CDocuments%20and%20Settings%5CAdministrator%5CDesktop%5CDystro2009_all%20-Project%5COutput%5Cbuona%20fshd%20inf%20VS%20inf%20biocarta%20PathwayClassComparison%5CGeneSetGenesTable1.html#h_reelinPathway) | 0.06148 | 0.31858 | 0.26 (+) | 0.003442 |
| 33 | h_metPathway | [Signaling of Hepatocyte Growth Factor Receptor](http://cgap.nci.nih.gov/Pathways/BioCarta/h_metPathway) | [31](../../../../C:%5CDocuments%20and%20Settings%5CAdministrator%5CDesktop%5CDystro2009_all%20-Project%5COutput%5Cbuona%20fshd%20inf%20VS%20inf%20biocarta%20PathwayClassComparison%5CGeneSetGenesTable1.html#h_metPathway) | 0.06652 | 0.08447 | 0.135 (+) | 0.003796 |
| 34 | h_monocytePathway | [Monocyte and its Surface Molecules](http://cgap.nci.nih.gov/Pathways/BioCarta/h_monocytePathway) | [9](../../../../C:%5CDocuments%20and%20Settings%5CAdministrator%5CDesktop%5CDystro2009_all%20-Project%5COutput%5Cbuona%20fshd%20inf%20VS%20inf%20biocarta%20PathwayClassComparison%5CGeneSetGenesTable1.html#h_monocytePathway) | 0.08073 | 0.38231 | 0.05 (-) | 0.0093807 |
| 35 | h_ck1Pathway | [Regulation of ck1/cdk5 by type 1 glutamate receptors](http://cgap.nci.nih.gov/Pathways/BioCarta/h_ck1Pathway) | [9](../../../../C:%5CDocuments%20and%20Settings%5CAdministrator%5CDesktop%5CDystro2009_all%20-Project%5COutput%5Cbuona%20fshd%20inf%20VS%20inf%20biocarta%20PathwayClassComparison%5CGeneSetGenesTable1.html#h_ck1Pathway) | 0.09342 | 0.49593 | 0.12 (+) | 0.0023642 |
| 36 | h_eea1Pathway | [The role of FYVE-finger proteins in vesicle transport](http://cgap.nci.nih.gov/Pathways/BioCarta/h_eea1Pathway) | [7](../../../../C:%5CDocuments%20and%20Settings%5CAdministrator%5CDesktop%5CDystro2009_all%20-Project%5COutput%5Cbuona%20fshd%20inf%20VS%20inf%20biocarta%20PathwayClassComparison%5CGeneSetGenesTable1.html#h_eea1Pathway) | 0.0974 | 0.32043 | 0.085 (-) | 0.0095916 |
| 37 | h_ngfPathway | [Nerve growth factor pathway (NGF)](http://cgap.nci.nih.gov/Pathways/BioCarta/h_ngfPathway) | [14](../../../../C:%5CDocuments%20and%20Settings%5CAdministrator%5CDesktop%5CDystro2009_all%20-Project%5COutput%5Cbuona%20fshd%20inf%20VS%20inf%20biocarta%20PathwayClassComparison%5CGeneSetGenesTable1.html#h_ngfPathway) | 0.11133 | 0.2899 | 0.02 (+) | 0.0056768 |
| 38 | h_bard1Pathway | [BRCA1-dependent Ub-ligase activity](http://cgap.nci.nih.gov/Pathways/BioCarta/h_bard1Pathway) | [6](../../../../C:%5CDocuments%20and%20Settings%5CAdministrator%5CDesktop%5CDystro2009_all%20-Project%5COutput%5Cbuona%20fshd%20inf%20VS%20inf%20biocarta%20PathwayClassComparison%5CGeneSetGenesTable1.html#h_bard1Pathway) | 0.21823 | 0.60991 | 0.04 (+) | 0.005818 |
| 39 | h_carm-erPathway | [CARM1 and Regulation of the Estrogen Receptor](http://cgap.nci.nih.gov/Pathways/BioCarta/h_carm-erPathway) | [21](../../../../C:%5CDocuments%20and%20Settings%5CAdministrator%5CDesktop%5CDystro2009_all%20-Project%5COutput%5Cbuona%20fshd%20inf%20VS%20inf%20biocarta%20PathwayClassComparison%5CGeneSetGenesTable1.html#h_carm-erPathway) | 0.22666 | 0.39558 | 0.215 (+) | 0.0059645 |
| 40 | h_sppaPathway | [Aspirin Blocks Signaling Pathway Involved in Platelet Activation](http://cgap.nci.nih.gov/Pathways/BioCarta/h_sppaPathway) | [17](../../../../C:%5CDocuments%20and%20Settings%5CAdministrator%5CDesktop%5CDystro2009_all%20-Project%5COutput%5Cbuona%20fshd%20inf%20VS%20inf%20biocarta%20PathwayClassComparison%5CGeneSetGenesTable1.html#h_sppaPathway) | 0.23267 | 0.00859 | 0.16 (-) | 0.0406723 |
| 41 | h_cellcyclePathway | [Cyclins and Cell Cycle Regulation](http://cgap.nci.nih.gov/Pathways/BioCarta/h_cellcyclePathway) | [21](../../../../C:%5CDocuments%20and%20Settings%5CAdministrator%5CDesktop%5CDystro2009_all%20-Project%5COutput%5Cbuona%20fshd%20inf%20VS%20inf%20biocarta%20PathwayClassComparison%5CGeneSetGenesTable1.html#h_cellcyclePathway) | 0.28227 | 0.2653 | 0.165 (-) | 0.0054272 |

| Gene Ontology Gene Set Expression Comparison FSHD T2-STIR + vs. Inflammatory myopathies | | | | | | | | |
| --- | --- | --- | --- | --- | --- | --- | --- | --- |
|  | **GO category** | **GO ontology** | **GO term** | **Number of genes** | **LS permutation p-value** | **KS permutation p-value** | **Efron-Tibshirani's GSA test p-value** | **Goeman's global test p-value** |
| 1 | GO:0016614 | MF | oxidoreductase activity, acting on CH-OH group of donors | [5](../../../../C:%5CDocuments%20and%20Settings%5CAdministrator%5CDesktop%5CDystro2009_all%20-Project%5COutput%5CBUONA%20fshd%20INF%20VS%20INFGOClassComparison%5CGeneSetGenesTable1.html#GO:0016614) | 0.00823 | 0.01448 | < 0.005 (-) | 0.0118829 |
| 2 | GO:0016616 | MF | oxidoreductase activity, acting on the CH-OH group of donors, NAD or NADP as acceptor | [5](../../../../C:%5CDocuments%20and%20Settings%5CAdministrator%5CDesktop%5CDystro2009_all%20-Project%5COutput%5CBUONA%20fshd%20INF%20VS%20INFGOClassComparison%5CGeneSetGenesTable1.html#GO:0016616) | 0.00823 | 0.01448 | < 0.005 (-) | 0.0118829 |
| 3 | GO:0016740 | MF | transferase activity | [24](../../../../C:%5CDocuments%20and%20Settings%5CAdministrator%5CDesktop%5CDystro2009_all%20-Project%5COutput%5CBUONA%20fshd%20INF%20VS%20INFGOClassComparison%5CGeneSetGenesTable1.html#GO:0016740) | 0.0532 | 0.08494 | 0.025 (+) | 0.0046111 |
| 4 | GO:0008194 | MF | UDP-glycosyltransferase activity | [7](../../../../C:%5CDocuments%20and%20Settings%5CAdministrator%5CDesktop%5CDystro2009_all%20-Project%5COutput%5CBUONA%20fshd%20INF%20VS%20INFGOClassComparison%5CGeneSetGenesTable1.html#GO:0008194) | 0.13516 | 0.01271 | 0.005 (-) | 0.2000147 |
| 5 | GO:0016757 | MF | transferase activity, transferring glycosyl groups | [7](../../../../C:%5CDocuments%20and%20Settings%5CAdministrator%5CDesktop%5CDystro2009_all%20-Project%5COutput%5CBUONA%20fshd%20INF%20VS%20INFGOClassComparison%5CGeneSetGenesTable1.html#GO:0016757) | 0.13516 | 0.01271 | 0.005 (-) | 0.2000147 |
| 6 | GO:0016758 | MF | transferase activity, transferring hexosyl groups | [7](../../../../C:%5CDocuments%20and%20Settings%5CAdministrator%5CDesktop%5CDystro2009_all%20-Project%5COutput%5CBUONA%20fshd%20INF%20VS%20INFGOClassComparison%5CGeneSetGenesTable1.html#GO:0016758) | 0.13516 | 0.01271 | 0.005 (-) | 0.2000147 |
| 7 | GO:0016491 | MF | oxidoreductase activity | [23](../../../../C:%5CDocuments%20and%20Settings%5CAdministrator%5CDesktop%5CDystro2009_all%20-Project%5COutput%5CBUONA%20fshd%20INF%20VS%20INFGOClassComparison%5CGeneSetGenesTable1.html#GO:0016491) | 0.15557 | 0.25748 | 0.005 (-) | 0.0117034 |
| 8 | GO:0051336 | BP | regulation of hydrolase activity | [7](../../../../C:%5CDocuments%20and%20Settings%5CAdministrator%5CDesktop%5CDystro2009_all%20-Project%5COutput%5CBUONA%20fshd%20INF%20VS%20INFGOClassComparison%5CGeneSetGenesTable1.html#GO:0051336) | 0.24597 | 0.27144 | 0.005 (+) | 0.0261375 |
| 9 | GO:0050790 | BP | regulation of catalytic activity | [10](../../../../C:%5CDocuments%20and%20Settings%5CAdministrator%5CDesktop%5CDystro2009_all%20-Project%5COutput%5CBUONA%20fshd%20INF%20VS%20INFGOClassComparison%5CGeneSetGenesTable1.html#GO:0050790) | 0.40118 | 0.44749 | 0.005 (+) | 0.0567995 |
| 10 | GO:0065009 | BP | regulation of molecular function | [10](../../../../C:%5CDocuments%20and%20Settings%5CAdministrator%5CDesktop%5CDystro2009_all%20-Project%5COutput%5CBUONA%20fshd%20INF%20VS%20INFGOClassComparison%5CGeneSetGenesTable1.html#GO:0065009) | 0.40118 | 0.44749 | 0.005 (+) | 0.0567995 |
| 11 | GO:0030528 | MF | transcription regulator activity | [59](../../../../C:%5CDocuments%20and%20Settings%5CAdministrator%5CDesktop%5CDystro2009_all%20-Project%5COutput%5CBUONA%20fshd%20INF%20VS%20INFGOClassComparison%5CGeneSetGenesTable1.html#GO:0030528) | 0.72786 | 0.63032 | 0.4 (+) | 0.004314 |

| KEGG Gene Set Expression Comparison FSHD T2-STIR + vs. Inflammatory myopathies | | | | | | | |
| --- | --- | --- | --- | --- | --- | --- | --- |
|  | **Kegg Pathway** | **Pathway description** | **Number of genes** | **LS permutation p-value** | **KS permutation p-value** | **Efron-Tibshirani's GSA test p-value** | **Goeman's global test p-value** |
| **1** | hsa04510 | [Focal adhesion](http://cgap.nci.nih.gov/Pathways/Kegg/hsa04510) | [174](../../../../C:%5CDocuments%20and%20Settings%5CAdministrator%5CDesktop%5CDystro2009_all%20-Project%5COutput%5Cbuona%20fshd%20INF%20VS%20INF%20%20KEGGPathwayClassComparison%5CGeneSetGenesTable1.html#hsa04510) | 0.00019 | 0.0042 | 0.09 (-) | 0.0653735 |
| **2** | hsa00750 | [Vitamin B6 metabolism](http://cgap.nci.nih.gov/Pathways/Kegg/hsa00750) | [8](../../../../C:%5CDocuments%20and%20Settings%5CAdministrator%5CDesktop%5CDystro2009_all%20-Project%5COutput%5Cbuona%20fshd%20INF%20VS%20INF%20%20KEGGPathwayClassComparison%5CGeneSetGenesTable1.html#hsa00750) | 0.00058 | 0.00008 | 0.01 (+) | 0.024161 |
| **3** | hsa03050 | [Proteasome](http://cgap.nci.nih.gov/Pathways/Kegg/hsa03050) | [30](../../../../C:%5CDocuments%20and%20Settings%5CAdministrator%5CDesktop%5CDystro2009_all%20-Project%5COutput%5Cbuona%20fshd%20INF%20VS%20INF%20%20KEGGPathwayClassComparison%5CGeneSetGenesTable1.html#hsa03050) | 0.00069 | 0.00039 | 0.005 (+) | 0.0123071 |
| **4** | hsa00561 | [Glycerolipid metabolism](http://cgap.nci.nih.gov/Pathways/Kegg/hsa00561) | [45](../../../../C:%5CDocuments%20and%20Settings%5CAdministrator%5CDesktop%5CDystro2009_all%20-Project%5COutput%5Cbuona%20fshd%20INF%20VS%20INF%20%20KEGGPathwayClassComparison%5CGeneSetGenesTable1.html#hsa00561) | 0.00072 | 0.02361 | < 0.005 (-) | 0.0033364 |
| **5** | hsa00350 | [Tyrosine metabolism](http://cgap.nci.nih.gov/Pathways/Kegg/hsa00350) | [44](../../../../C:%5CDocuments%20and%20Settings%5CAdministrator%5CDesktop%5CDystro2009_all%20-Project%5COutput%5Cbuona%20fshd%20INF%20VS%20INF%20%20KEGGPathwayClassComparison%5CGeneSetGenesTable1.html#hsa00350) | 0.00391 | 0.0112 | < 0.005 (+) | 0.0052766 |
| **6** | hsa00290 | [Valine, leucine and isoleucine biosynthesis](http://cgap.nci.nih.gov/Pathways/Kegg/hsa00290) | [8](../../../../C:%5CDocuments%20and%20Settings%5CAdministrator%5CDesktop%5CDystro2009_all%20-Project%5COutput%5Cbuona%20fshd%20INF%20VS%20INF%20%20KEGGPathwayClassComparison%5CGeneSetGenesTable1.html#hsa00290) | 0.00552 | 0.0561 | 0.005 (+) | 0.0247333 |
| **7** | hsa00730 | [Thiamine metabolism](http://cgap.nci.nih.gov/Pathways/Kegg/hsa00730) | [6](../../../../C:%5CDocuments%20and%20Settings%5CAdministrator%5CDesktop%5CDystro2009_all%20-Project%5COutput%5Cbuona%20fshd%20INF%20VS%20INF%20%20KEGGPathwayClassComparison%5CGeneSetGenesTable1.html#hsa00730) | 0.00614 | 0.00454 | 0.015 (+) | 0.0274185 |
| **8** | hsa04130 | [SNARE interactions in vesicular transport](http://cgap.nci.nih.gov/Pathways/Kegg/hsa04130) | [31](../../../../C:%5CDocuments%20and%20Settings%5CAdministrator%5CDesktop%5CDystro2009_all%20-Project%5COutput%5Cbuona%20fshd%20INF%20VS%20INF%20%20KEGGPathwayClassComparison%5CGeneSetGenesTable1.html#hsa04130) | 0.00703 | 0.20003 | 0.035 (-) | 0.0139324 |
| **9** | hsa04710 | [Circadian rhythm](http://cgap.nci.nih.gov/Pathways/Kegg/hsa04710) | [14](../../../../C:%5CDocuments%20and%20Settings%5CAdministrator%5CDesktop%5CDystro2009_all%20-Project%5COutput%5Cbuona%20fshd%20INF%20VS%20INF%20%20KEGGPathwayClassComparison%5CGeneSetGenesTable1.html#hsa04710) | 0.00829 | 0.02419 | 0.085 (-) | 0.0172586 |
| **10** | hsa00624 | [1- and 2-Methylnaphthalene degradation](http://cgap.nci.nih.gov/Pathways/Kegg/hsa00624) | [20](../../../../C:%5CDocuments%20and%20Settings%5CAdministrator%5CDesktop%5CDystro2009_all%20-Project%5COutput%5Cbuona%20fshd%20INF%20VS%20INF%20%20KEGGPathwayClassComparison%5CGeneSetGenesTable1.html#hsa00624) | 0.01025 | 0.01981 | 0.125 (-) | 0.0022353 |
| **11** | hsa00760 | [Nicotinate and nicotinamide metabolism](http://cgap.nci.nih.gov/Pathways/Kegg/hsa00760) | [40](../../../../C:%5CDocuments%20and%20Settings%5CAdministrator%5CDesktop%5CDystro2009_all%20-Project%5COutput%5Cbuona%20fshd%20INF%20VS%20INF%20%20KEGGPathwayClassComparison%5CGeneSetGenesTable1.html#hsa00760) | 0.01086 | 0.00187 | 0.075 (+) | 0.0038056 |
| **12** | hsa05210 | [Colorectal cancer](http://cgap.nci.nih.gov/Pathways/Kegg/hsa05210) | [68](../../../../C:%5CDocuments%20and%20Settings%5CAdministrator%5CDesktop%5CDystro2009_all%20-Project%5COutput%5Cbuona%20fshd%20INF%20VS%20INF%20%20KEGGPathwayClassComparison%5CGeneSetGenesTable1.html#hsa05210) | 0.0203 | 0.03219 | 0.12 (-) | 0.0053576 |
| **13** | hsa04630 | [Jak-STAT signaling pathway](http://cgap.nci.nih.gov/Pathways/Kegg/hsa04630) | [111](../../../../C:%5CDocuments%20and%20Settings%5CAdministrator%5CDesktop%5CDystro2009_all%20-Project%5COutput%5Cbuona%20fshd%20INF%20VS%20INF%20%20KEGGPathwayClassComparison%5CGeneSetGenesTable1.html#hsa04630) | 0.02437 | 0.0167 | < 0.005 (-) | 0.0158177 |
| **14** | hsa00600 | [Sphingolipid metabolism](http://cgap.nci.nih.gov/Pathways/Kegg/hsa00600) | [26](../../../../C:%5CDocuments%20and%20Settings%5CAdministrator%5CDesktop%5CDystro2009_all%20-Project%5COutput%5Cbuona%20fshd%20INF%20VS%20INF%20%20KEGGPathwayClassComparison%5CGeneSetGenesTable1.html#hsa00600) | 0.02788 | 0.01889 | 0.11 (-) | 0.002088 |
| **15** | hsa00960 | [Alkaloid biosynthesis II](http://cgap.nci.nih.gov/Pathways/Kegg/hsa00960) | [14](../../../../C:%5CDocuments%20and%20Settings%5CAdministrator%5CDesktop%5CDystro2009_all%20-Project%5COutput%5Cbuona%20fshd%20INF%20VS%20INF%20%20KEGGPathwayClassComparison%5CGeneSetGenesTable1.html#hsa00960) | 0.02854 | 0.00879 | 0.095 (-) | 0.0112003 |
| **16** | hsa00632 | [Benzoate degradation via CoA ligation](http://cgap.nci.nih.gov/Pathways/Kegg/hsa00632) | [25](../../../../C:%5CDocuments%20and%20Settings%5CAdministrator%5CDesktop%5CDystro2009_all%20-Project%5COutput%5Cbuona%20fshd%20INF%20VS%20INF%20%20KEGGPathwayClassComparison%5CGeneSetGenesTable1.html#hsa00632) | 0.02935 | 0.00963 | 0.005 (+) | 0.0166418 |
| **17** | hsa00450 | [Selenoamino acid metabolism](http://cgap.nci.nih.gov/Pathways/Kegg/hsa00450) | [30](../../../../C:%5CDocuments%20and%20Settings%5CAdministrator%5CDesktop%5CDystro2009_all%20-Project%5COutput%5Cbuona%20fshd%20INF%20VS%20INF%20%20KEGGPathwayClassComparison%5CGeneSetGenesTable1.html#hsa00450) | 0.04349 | 0.0034 | 0.005 (+) | 0.0085922 |
| **18** | hsa00562 | [Inositol phosphate metabolism](http://cgap.nci.nih.gov/Pathways/Kegg/hsa00562) | [45](../../../../C:%5CDocuments%20and%20Settings%5CAdministrator%5CDesktop%5CDystro2009_all%20-Project%5COutput%5Cbuona%20fshd%20INF%20VS%20INF%20%20KEGGPathwayClassComparison%5CGeneSetGenesTable1.html#hsa00562) | 0.04891 | 0.04337 | 0.035 (+) | 0.0037088 |
| **19** | hsa04070 | [Phosphatidylinositol signaling system](http://cgap.nci.nih.gov/Pathways/Kegg/hsa04070) | [64](../../../../C:%5CDocuments%20and%20Settings%5CAdministrator%5CDesktop%5CDystro2009_all%20-Project%5COutput%5Cbuona%20fshd%20INF%20VS%20INF%20%20KEGGPathwayClassComparison%5CGeneSetGenesTable1.html#hsa04070) | 0.05472 | 0.20822 | 0.045 (+) | 0.0033879 |
| **20** | hsa00740 | [Riboflavin metabolism](http://cgap.nci.nih.gov/Pathways/Kegg/hsa00740) | [14](../../../../C:%5CDocuments%20and%20Settings%5CAdministrator%5CDesktop%5CDystro2009_all%20-Project%5COutput%5Cbuona%20fshd%20INF%20VS%20INF%20%20KEGGPathwayClassComparison%5CGeneSetGenesTable1.html#hsa00740) | 0.05593 | 0.02135 | 0.07 (+) | 0.0082256 |
| **21** | hsa00120 | [Bile acid biosynthesis](http://cgap.nci.nih.gov/Pathways/Kegg/hsa00120) | [34](../../../../C:%5CDocuments%20and%20Settings%5CAdministrator%5CDesktop%5CDystro2009_all%20-Project%5COutput%5Cbuona%20fshd%20INF%20VS%20INF%20%20KEGGPathwayClassComparison%5CGeneSetGenesTable1.html#hsa00120) | 0.06841 | 0.16612 | 0.025 (-) | 0.0026362 |
| **22** | hsa04360 | [Axon guidance](http://cgap.nci.nih.gov/Pathways/Kegg/hsa04360) | [112](../../../../C:%5CDocuments%20and%20Settings%5CAdministrator%5CDesktop%5CDystro2009_all%20-Project%5COutput%5Cbuona%20fshd%20INF%20VS%20INF%20%20KEGGPathwayClassComparison%5CGeneSetGenesTable1.html#hsa04360) | 0.08008 | 0.06443 | 0.19 (-) | 0.0046045 |
| **23** | hsa00980 | [Metabolism of xenobiotics by cytochrome P450](http://cgap.nci.nih.gov/Pathways/Kegg/hsa00980) | [50](../../../../C:%5CDocuments%20and%20Settings%5CAdministrator%5CDesktop%5CDystro2009_all%20-Project%5COutput%5Cbuona%20fshd%20INF%20VS%20INF%20%20KEGGPathwayClassComparison%5CGeneSetGenesTable1.html#hsa00980) | 0.08099 | 0.14014 | 0.02 (-) | 0.0038653 |
| **24** | hsa00903 | [Limonene and pinene degradation](http://cgap.nci.nih.gov/Pathways/Kegg/hsa00903) | [23](../../../../C:%5CDocuments%20and%20Settings%5CAdministrator%5CDesktop%5CDystro2009_all%20-Project%5COutput%5Cbuona%20fshd%20INF%20VS%20INF%20%20KEGGPathwayClassComparison%5CGeneSetGenesTable1.html#hsa00903) | 0.08173 | 0.10215 | 0.19 (+) | 0.0095368 |
| **25** | hsa00626 | [Nitrobenzene degradation](http://cgap.nci.nih.gov/Pathways/Kegg/hsa00626) | [11](../../../../C:%5CDocuments%20and%20Settings%5CAdministrator%5CDesktop%5CDystro2009_all%20-Project%5COutput%5Cbuona%20fshd%20INF%20VS%20INF%20%20KEGGPathwayClassComparison%5CGeneSetGenesTable1.html#hsa00626) | 0.11181 | 0.12203 | 0.005 (+) | 0.0143432 |
| **26** | hsa00071 | [Fatty acid metabolism](http://cgap.nci.nih.gov/Pathways/Kegg/hsa00071) | [46](../../../../C:%5CDocuments%20and%20Settings%5CAdministrator%5CDesktop%5CDystro2009_all%20-Project%5COutput%5Cbuona%20fshd%20INF%20VS%20INF%20%20KEGGPathwayClassComparison%5CGeneSetGenesTable1.html#hsa00071) | 0.13657 | 0.09844 | 0.205 (-) | 0.0030278 |
| **27** | hsa03022 | [Basal transcription factors](http://cgap.nci.nih.gov/Pathways/Kegg/hsa03022) | [28](../../../../C:%5CDocuments%20and%20Settings%5CAdministrator%5CDesktop%5CDystro2009_all%20-Project%5COutput%5Cbuona%20fshd%20INF%20VS%20INF%20%20KEGGPathwayClassComparison%5CGeneSetGenesTable1.html#hsa03022) | 0.18928 | 0.17473 | 0.165 (+) | 0.005322 |
| **28** | hsa04110 | [Cell cycle](http://cgap.nci.nih.gov/Pathways/Kegg/hsa04110) | [103](../../../../C:%5CDocuments%20and%20Settings%5CAdministrator%5CDesktop%5CDystro2009_all%20-Project%5COutput%5Cbuona%20fshd%20INF%20VS%20INF%20%20KEGGPathwayClassComparison%5CGeneSetGenesTable1.html#hsa04110) | 0.27749 | 0.54502 | 0.21 (+) | 0.000201 |
| **29** | hsa00010 | [Glycolysis / Gluconeogenesis](http://cgap.nci.nih.gov/Pathways/Kegg/hsa00010) | [50](../../../../C:%5CDocuments%20and%20Settings%5CAdministrator%5CDesktop%5CDystro2009_all%20-Project%5COutput%5Cbuona%20fshd%20INF%20VS%20INF%20%20KEGGPathwayClassComparison%5CGeneSetGenesTable1.html#hsa00010) | 0.34027 | 0.30908 | 0.405 (-) | 0.0062137 |
| **30** | hsa00272 | [Cysteine metabolism](http://cgap.nci.nih.gov/Pathways/Kegg/hsa00272) | [18](../../../../C:%5CDocuments%20and%20Settings%5CAdministrator%5CDesktop%5CDystro2009_all%20-Project%5COutput%5Cbuona%20fshd%20INF%20VS%20INF%20%20KEGGPathwayClassComparison%5CGeneSetGenesTable1.html#hsa00272) | 0.35922 | 0.65315 | 0.485 (-) | 0.0095574 |
| **31** | hsa00564 | [Glycerophospholipid metabolism](http://cgap.nci.nih.gov/Pathways/Kegg/hsa00564) | [60](../../../../C:%5CDocuments%20and%20Settings%5CAdministrator%5CDesktop%5CDystro2009_all%20-Project%5COutput%5Cbuona%20fshd%20INF%20VS%20INF%20%20KEGGPathwayClassComparison%5CGeneSetGenesTable1.html#hsa00564) | 0.36197 | 0.65764 | 0.235 (-) | 0.0069964 |
| **32** | hsa04010 | [MAPK signaling pathway](http://cgap.nci.nih.gov/Pathways/Kegg/hsa04010) | [222](../../../../C:%5CDocuments%20and%20Settings%5CAdministrator%5CDesktop%5CDystro2009_all%20-Project%5COutput%5Cbuona%20fshd%20INF%20VS%20INF%20%20KEGGPathwayClassComparison%5CGeneSetGenesTable1.html#hsa04010) | 0.42064 | 0.23139 | 0.175 (+) | 0.008301 |
| **33** | hsa03320 | [PPAR signaling pathway](http://cgap.nci.nih.gov/Pathways/Kegg/hsa03320) | [56](../../../../C:%5CDocuments%20and%20Settings%5CAdministrator%5CDesktop%5CDystro2009_all%20-Project%5COutput%5Cbuona%20fshd%20INF%20VS%20INF%20%20KEGGPathwayClassComparison%5CGeneSetGenesTable1.html#hsa03320) | 0.55265 | 0.86039 | 0.125 (-) | 0.0045961 |
| **34** | hsa01030 | [Glycan structures - biosynthesis 1](http://cgap.nci.nih.gov/Pathways/Kegg/hsa01030) | [92](../../../../C:%5CDocuments%20and%20Settings%5CAdministrator%5CDesktop%5CDystro2009_all%20-Project%5COutput%5Cbuona%20fshd%20INF%20VS%20INF%20%20KEGGPathwayClassComparison%5CGeneSetGenesTable1.html#hsa01030) | 0.57823 | 0.70155 | 0.56 (+) | 0.0011524 |
| **35** | hsa00512 | [O-Glycan biosynthesis](http://cgap.nci.nih.gov/Pathways/Kegg/hsa00512) | [21](../../../../C:%5CDocuments%20and%20Settings%5CAdministrator%5CDesktop%5CDystro2009_all%20-Project%5COutput%5Cbuona%20fshd%20INF%20VS%20INF%20%20KEGGPathwayClassComparison%5CGeneSetGenesTable1.html#hsa00512) | 0.7993 | 0.90785 | 0.395 (+) | 0.0009958 |
