# Supplementary material for: Different Molecular Signatures in Magnetic Resonance Imaging-Staged Facioscapulohumeral Muscular Dystrophy Muscles
Source: PLoS One. 2012 Jun 13;7(6):e38779. doi: 10.1371/journal.pone.0038779 (PMC3374833; doi:10.1371/journal.pone.0038779)
Supplement: Text S1 — Supplementary methods: gene set expression comparison and real time PCR. (DOC) [file pone.0038779.s009.doc]

**Text S1**

**Supplementary Methods**

**Gene Set Expression Comparison**

We identified gene sets (Gene Ontology, BioCarta, KEGG) whose expression was differentially regulated among the classes. By analyzing functional categories, rather than individual genes, we were able to reduce the number of tests conducted, and to enable findings among biologically related genes to reinforce each other. This analysis is different than annotating a gene list using categories. For each gene set we computed the number n of genes represented on the microarray in that group, and the statistical significance p_i_ value for each gene i in the group. These p values reflect differential expression among classes and were computed based on random variance t-tests or F-tests [1]. For any given group, two statistics are computed that summarize the p values for genes in the group; the Fisher (LS) statistic and the Kolmogorov-Smirnov (KS) statistic as described in [2]. Samples of n genes are randomly selected from genes represented on the array and the summary statistic computed for those random samples. The significance level associated with the category is the proportion of the random samples giving as large a value of the summary statistic as in the actual n genes of the category. For each category, two significance levels are computed, corresponding to the two summary statistics. We considered a category significantly differentially regulated if either significance level was less than 0.01. We considered all categories with between 5 and 100 genes represented on the array.

**Real time PCR**

Real time polymerase chain reaction (PCR) reactions were performed on skeletal muscle RNA from all the samples that underwent gene expression analysis. First-strand cDNA was synthesized from 1 μg total RNA using Superscript VILO cDNA synthesis kit (Applied Biosystems, Foster City, CA, USA), following the manufacturer’s instructions. Real time PCR was performed on an ABI Prism 7000 Sequence Detection System using the Applied Biosystems TaqMan Universal Master

Mix II (no UNG) and the following gene-specific TaqMan primer sets: CIDEA Hs00154455m_1, SFRP1 Hs00610060_m1, C1S Hs01043795_m1, C3 Hs01100879_m1, ADIPOQ Hs00605917_m1, PRF1 Hs00169473_m1. GAPDH (Hs99999905_m1) and HPRT1 (Hs99999909_m1) were used as endogenous control genes.

**References**

**1.** Wright GW, Simon RM. A random variance model for detection of differential gene expression in small microarray experiments. *Bioinformatics.* 2003;19(18):2448-2455.

**2.** Simon R LA. BRB-ArrayTools User Guide, version 3.2. Biometric Research Branch, NationalCancerInstitute.
